# Genomic insights and evaluation of *Bacillus spizizenii* Endophyte from *Solanum elaeagnifolium Cav* against multidrug-resistant pathogens

**DOI:** 10.3389/fcimb.2026.1929148

**Published:** 2026-09-03

**Authors:** Merihan S. Sabra, Lina Jamil M. Abdel-Hafez, Amr S. Bishr, Hesham Ali El Enshasy, Mohamed H Al-Agamy, Khalid Alyahya, Khaled M. Aboshanab

**Affiliations:** 1 Department of Microbiology and Immunology, Faculty of Pharmacy, October 6 University, Giza, Egypt; 2 Department of Microbiology and Immunology, Faculty of Pharmacy, Ain Shams University, Cairo, Egypt; 3 Innovation Centre in Agritechnology in Advanced Bioprocessing (ICA), Universiti Teknologi Malaysia, Higher Education Hup, Pagoh, Johor, Malaysia; 4 Department of Pharmaceutics, College of Pharmacy, King Saud University, Riyadh, Saudi Arabia

**Keywords:** antiviral, *Bacillus spizizenii*, endophyte, *Solanum elaeagnifolium*, whole genome sequencing

## Abstract

Secondary metabolites are promising alternatives due to their diverse biological activities, including antimicrobial and antiviral effects. Wild plants, particularly *Solanum elaeagnifolium* Cav (Silverleaf Nightshade), harbor endophytic microorganisms that represent an untapped source of such bioactive compounds. In the present study, four bacterial endophytes were isolated from the leaves of *Solanum elaeagnifolium* Cav (LS1, LS2, LS3, and LS4), and their extracts (S1, S2, S3, and S4) were prepared. Extract S4 showed both qualitative and quantitative inhibitory activity against a broad range of standard and multidrug-resistant (MDR) pathogens, including Gram-positive and Gram-negative pathogens. Moreover, it showed significant antiviral effects against Adenovirus serotype 7 and Herpes Simplex Viruses 1 and 2. The endophytic bacterium LS4 was identified microscopically, biochemically, and via whole-genome sequencing as *Bacillus spizizenii* CCASU-2025-89. Cytotoxic assessment of S4 revealed a CC_50_ of 80.71 μg/mL and a safe concentration of 6.23 μg/mL. Whole-genome analysis revealed conserved biosynthetic gene clusters of antimicrobial peptides including bacilysin, subtilin, mycosubtilin, bacillaene, bacillibactin, subtilosin A, and Pulcherriminic acid. Overall, this study demonstrates that endophytic bacterium *Bacillus spizizenii* from *Solanum elaeagnifolium* represents a promising resource for developing new antimicrobial and antiviral therapeutics.

## 1 Introduction

Antimicrobial resistance (AMR) is considered a significant risk to human health and a developmental barrier. AMR primarily contributes to the erroneous and excessive consumption of antimicrobials across many sectors, such as human, animal, and agriculture (McEwen and Collignon, 2018). The inappropriate use of antibiotics contributes to widespread infection-related morbidity and mortality worldwide (Founou et al., 2021). According to documented studies on the social effects of Antimicrobial Resistance, the mortality rate is expected to increase by up to 10 million deaths in 2050 (Naghavi et al., 2024). These challenges necessitate the development of new bioactive compounds from natural sources, particularly from plants, animals, and microorganisms (Narayanan and Glick, 2022). Medicinal plants constitute an important source of bioactive compounds to treat numerous health conditions. Interestingly, in recent years, microbes linked to plants rather than plants themselves have demonstrated the ability to provide materials and products with significant medicinal promise (Gouda et al., 2016). In particular, wild plants are a valuable reservoir of unique endophytic microorganisms. Endophytes comprise bacteria and fungi that inhabit the internal tissues of plants for at least a portion of their lives without creating obvious disease signs.

Endophytic bacteria are thought to be a useful source of a variety of secondary metabolites, including peptides with medicinal potential. Furthermore, endophytic microbes have a variety of biological functions, including antiviral, antifungal, anticancer, and antibacterial activities (Najjar, 2025). Among the various bioactive metabolites produced by endophytic microorganisms, antimicrobial peptides represent a largely unexplored and promising source of novel therapeutic agents (Tejesvi et al., 2013). Antimicrobial peptides (AMPs) are bioactive compounds that serve as natural host defense mechanisms in many species. They have broad-spectrum antibacterial action, disrupting microbial membranes and targeting intracellular processes, making them promising alternatives to traditional antibiotics (Kim et al., 2025). Accordingly, the present study was designed to isolate and characterize endophytic bacteria from the wild plant *Solanum elaeagnifolium*, with particular emphasis on the isolate identified as *Bacillus spizizenii*, and to evaluate its antimicrobial potential.

## 2 Materials and methods

### 2.1 Plant sampling and taxonomic identification

A symptom-free wild plant specimen was obtained on 14 August 2024 using a sterile cutting tool while wearing sterile gloves, then placed in a sterile sampling bag (Srivastava et al., 2024). The sample was obtained from the rocky desert area along Old Borg El-Arab Road, near Borg El-Arab City, Alexandria Governorate, Egypt (approximate location: 30.918° N, 29.573° E), where the plant grows naturally in a dry habitat characterized by sandy, low-fertility soil. Plant collection was carried out in compliance with the applicable national regulations to ensure accurate identification. The plant was taxonomically identified by Dr. Walaa Amer, Professor of Plant Taxonomy and Flora, at the Cairo University Herbarium (CAI), following the Engler classification system, as *Solanum elaeagnifolium* Cav. The plant sample was deposited in the Culture Collection of Ain Shams University under the deposition code CCASU-SE-2025-89.

### 2.2 Isolation and preliminary characterization of endophytic bacteria

The plant specimen, including leaves, was subjected to surface sterilization before the isolation of endophytes to eliminate epiphytic microorganisms (Alkahtani et al., 2020). Leaves were first rinsed under running water to remove dust and debris, and then cut into smaller sections. For surface sterilization, the leaf segments were immersed in a 5% sodium hypochlorite solution for 5 minutes, then transferred to 70% ethanol for 1 minute. Finally, the segments were washed three times with sterile distilled water before further processing. To confirm the effectiveness of the sterilization process, the final rinse water was plated on Mueller-Hinton agar and monitored for microbial growth (Sahu et al., 2022). Approximately 2g of sterilized tissue was aseptically macerated using a sterile pestle and mortar in 18 mL of sterile saline. One mL of the resulting suspension was spread onto Mueller-Hinton agar (Hagaggi and Mohamed, 2020). The inoculated plates were maintained at 37 °C for 72 hours, and colony morphology was examined daily. Distinct colonies were selected based on differences in morphology, size, and elevation, and were repeatedly streaked (2–3 times) onto Mueller-Hinton agar at 37 °C for 24–48 hours to obtain pure bacterial isolates. The bacterial isolates were initially identified according to their phenotypic characteristics, including colony morphology (shape, size, and pigmentation) and Gram stain (Mahlangu et al., 2024). Selected bacterial isolates were maintained in 30% (v/v) glycerol and kept at −20 °C until further use.

### 2.3 Preparation of crude extracts from endophytic bacteria

The isolates were revived by subculturing on Mueller-Hinton agar. For preculturing, a single bacterial colony was inoculated into 30 mL of tryptic soy broth (TSB) and incubated overnight at 37 °C on a shaker at 150 rpm. Subsequently, 1.0 mL of this preculture (1.0 × 10^8^ CFU/mL) was transferred to 100 mL of fresh TSB as the main culture and incubated for 72 hours at 37 °C with shaking at 150 rpm (Hagaggi and Mohamed, 2020). After incubation, the culture was centrifuged at 10,000 rpm for 10 minutes at 4 °C to separate the supernatant from the cells. The supernatant was first filtered through sterile Whatman No.1 filter paper and then subjected to membrane filtration using a 0.2 μm PTFE membrane (Merck Co., Darmstadt, Germany). An equal volume of ethyl acetate was added to the cell-free supernatant (1:1, v/v) and then shaken at 5 °C at 150 rpm. The extraction was repeated three times to maximize compound recovery. The organic phases were collected and concentrated to dryness using a rotary evaporator (BUCHI, Germany) under reduced pressure using a rotary evaporator at 45 °C and 240 Pa. The resulting residue was dissolved in pure dimethyl sulfoxide (DMSO) (Abdel-Nasser et al., 2023). The yield of the crude extract varied between 70 and 100 mg per 100 mL of culture broth.

### 2.4 Antimicrobial screening and selection of the most active isolate

The antimicrobial activity of crude extracts prepared from the four bacterial endophytic isolates was evaluated against 3 standard strains (*S. aureus* ATCC 25923, *P. aeruginosa* ATCC 9027, and *C. albicans* ATCC 10231) and 9 clinical multidrug-resistant (MDR) isolates (*P. aeruginosa* (CCASU-28; CCASU-45, CCASU-47); *S. aureus* (CCASU-4 and CCASU-9); *Klebsiella pneumoniae* (CCASU-22 and CCASU-36); and MRSA (CCASU-5 and CCASU-8) as previously tested (Magiorakos et al., 2012). The antimicrobial susceptibility patterns of the clinical MDR isolates were previously determined according to the Clinical and Laboratory Standards Institute (CLSI) guidelines, as reported in our previous study (Sabra et al., 2026). The MDR clinical isolates were obtained from the Culture Collection Ain Shams University (CCASU), Cairo, Egypt. All bacterial isolates used in this study were preserved at −20 °C in Mueller–Hinton broth supplemented with 20% glycerol until use, as previously described (Soliman et al., 2024). The 4 extracts (S1, S2, S3, and S4) were evaluated following the same protocol (Soliman et al., 2024). Ciprofloxacin (8 μg/mL), gentamicin (5 mg/mL), and fluconazole (1 mg/mL) were used as positive controls. The inoculated plates were incubated at 37 °C for 24 hours, after which the inhibition zones were measured. All experiments were performed in triplicate, and data are presented as mean ± standard deviation (SD). Statistical analysis was carried out using GraphPad Prism version 10.3.1 (GraphPad Software, San Diego, CA, USA). Differences among groups were analyzed using one-way analysis of variance (ANOVA) followed by Dunnett’s multiple comparisons test, using the corresponding positive control (ciprofloxacin, gentamicin, and fluconazole) as the reference group. A p-value < 0.05 was considered statistically significant. The extract exhibiting the broadest antimicrobial activity was chosen for further evaluation of minimum inhibitory concentration (MIC), cytotoxic and antiviral activities. The endophyte exhibiting promising antimicrobial activity was subjected to whole-genome sequencing to facilitate accurate taxonomic identification and genome mining for biosynthetic gene clusters using antiSMASH (Sabra et al., 2026).

### 2.5 Cytotoxicity evaluation

The cytotoxic effect of the organic extract, showing promising antimicrobial effects, was evaluated on the Vero cell line to assess the non-cytotoxic concentration using the MTT assay (Van Meerloo et al., 2011; Bahuguna et al., 2017). The extract was prepared at a concentration of 25500 μg/mL, followed by successive two-fold serial dilutions on pre-grown cell lines. After removing the growth medium, the cells were exposed to the extract and incubated for 24 hours at 37 °C. The treated cell cultures were then examined microscopically to observe any morphological alterations or cell detachment. Subsequently, each well received 25 µL of 0.5% MTT solution, followed by incubation at 37 °C for 3–4 h. The formed intracellular formazan crystals were solubilized using 50 µL of dimethyl sulfoxide (DMSO) in each well, followed by shaking for 30 minutes. The optical density (OD) was calculated using a BioTek 800 ELISA plate reader (USA), and the percentage of cell viability was determined. The 50% cytotoxic concentration (CC_50_) of the extract was calculated using the MasterPlex-2010 software. A non-cytotoxic concentration of the extract was subsequently used for the antiviral evaluation (Keshavarz et al., 2021). The percentage of viable cells was calculated as previously described (Zakzouk et al., 2024).

### 2.6 Antiviral assay

The bioactive extract with strong antimicrobial properties was tested for antiviral effects as previously reported by Petricevich and Mendonça, 2003. The antiviral activity was assessed against Human Adenovirus serotype 7, Herpes Simplex Virus 1, and Herpes Simplex Virus 2. Viral titers were measured both in the presence and absence of the extract to determine its inhibitory effect. Vero cells were plated into 96-well culture plates at a density of 1 × 10^5^ cells/mL and incubated until a confluent monolayer was formed. Non-cytotoxic concentrations of the extract (6.23 μg/mL) were applied to the cells and incubated for 24 hours at 37 °C before viral inoculation for the assessment of the impact during the early stages of viral replication. For viral controls, untreated wells were maintained. The viruses were diluted serially at tenfold steps using Eagle’s Minimum Essential Medium (E-MEM), and 0.1 mL of each dilution was inoculated into the wells, regardless of extract treatment. Negative controls consisted of untreated cell wells, while acyclovir (10 µg/mL) was designated as the positive control, and the plates were incubated at 37 °C with daily monitoring using an inverted microscope. After three days, viral infectivity was determined by evaluating the cytopathic effect (CPE), and the 50% cell culture infectious dose (CCID50) was estimated from the number of wells exhibiting CPE at each viral dilution, using the method of Reed and Muench (1938). All titrations were carried out three times, followed by statistical analysis to establish significance.

### 2.7 Whole-genome sequencing of the selected isolate

Genomic DNA was isolated using the QIAamp^®^ DNA Mini Kit (catalog number 51304, Qiagen, Hilden, Germany) following the protocol provided by the manufacturer. DNA quantity and purity were evaluated using the Qubit 4 Fluorometer (Thermo Fisher Scientific, Massachusetts, USA). Approximately 400 ng of the purified DNA was employed to construct a sequencing library with the Rapid Sequencing Kit (SQK-RAD004; Oxford Nanopore Technologies, Oxford, UK), as specified by the manufacturer. Whole-genome sequencing was performed using the MinION™ device with an R10.4.1 flow cell (FLO-MIN114; Oxford Nanopore Technologies) and MinKNOW software ver. 24.11.10 (Oxford Nanopore Technologies) was used for data acquisition, as previously described (Sabra et al., 2026). The Fastq files were classified using kraken2 v 2.17.1 (Wood et al., 2019) with a custom database containing sequences for archaea, bacteria, fungi, and viral and then Bracken v 3.1 (Lu et al., 2017) was used to estimate species abundance using those classifications, then visualized using Krona v 2.8.1 (Ondov et al., 2011) to obtain taxonomic classification. medaka (github.com/nanoporetech/medaka, v 2.2.2) was used to align fastq reads to the reference genome of *Bacillus spizizenii* (NC_016047.1), call variants, and generate consensus. Consensus FASTA was used for gene annotation using Bakta v1.12 (Schwengers et al., 2021) using the full database; antimicrobial resistance genes were identified using ResFinder v 4.0 (Bortolaia et al., 2020).

The summary statistics of the raw nanopore sequencing quality control metrics (read N50, coverage depth, Q-score distribution) of *Bacillus spizizenii* CCASU-2025–89 genomic analysis after filtering are shown in **Table 1**.

**Table 1:** Summary statistics of genomic analysis of *Bacillus spizizenii* CCASU-2025–89 after filtering.

General summary	
Mean read length	460.1
Mean read quality	12.3
Median read length	226.0
Median read quality	13.4
Number of reads	132,135.0
Read length N50	891.0
STDEV read length	788.9
Total bases	60,789,037.0
Number, percentage and megabases of reads above quality cutoffs	
>Q10	115418 (87.3%) 57.0Mb
>Q15	37847 (28.6%) 21.0Mb
>Q20	3248 (2.5%) 0.7Mb
>Q25	328 (0.2%) 0.0Mb
>Q30	34 (0.0%) 0.0Mb
Top 5 highest mean basecall quality scores and their read lengths	
1	34.3 (181)
2	32.9 (192)
3	32.8 (114)
4	32.8 (43)
5	32.6 (27)
Top 5 longest reads and their mean basecall quality score	
1	25440 (11.6)
2	21555 (12.7)
3	21530 (16.3)
4	20617 (9.5)
5	19594 (15.5)

### 2.8 Bioinformatic analysis of sequencing data

Raw sequencing reads generated by MinION™ (POD5 data) were processed into FASTQ files using the Dorado basecall server version 7.3.9 (Oxford Nanopore Technologies) on an AWS EC2 g4dn.xlarge instance. Taxonomic assignment of sequencing reads was carried out using Kraken2 (Wood et al., 2019). Species abundance was estimated with Bracken (Lu et al., 2017) based on a custom-built reference database containing sequences from archaea, bacteria, fungi, and viruses, and visualized with Krona (Ondov et al., 2011). For consensus sequence generation, Medaka was employed to map reads against the reference genome, call variants, and generate a polished consensus. The resulting consensus sequence was annotated using Prokka (Seemann, 2014) with default parameters to predict gene content.

### 2.9 Identification of biosynthetic gene clusters

The detection and analysis of potential secondary metabolite biosynthetic gene clusters were performed using antiSMASH version 7 following the guidelines described by Blin et al., 2021 and Sabra et al., 2026.

## 3 Results

### 3.1 Isolation and identification of endophytic bacteria

The isolation procedure yielded four endophytic bacterial isolates, designated LS1, LS2, LS3, and LS4, obtained from the leaves of *Solanum elaeagnifolium* Cav. The recovered isolates differed in their phenotypic traits, including Gram staining and colony morphology. LS1 and LS2 were identified as Gram-negative coccobacilli producing creamy, circular colonies. In contrast, LS3 and LS4 were Gram-positive rods; LS3 formed off-white, circular colonies, whereas LS4 exhibited creamy colonies with irregular margins.

### 3.2 Extraction and screening of antimicrobial activity

The inhibition zones (mm) produced by the four crude organic extracts (S1, S2, S3, and S4) against the tested microbial isolates (n = 12) are presented in **Table 2** and **Supplementary Figure S1**. Statistical analysis of the inhibition zone diameters using one-way ANOVA followed by Dunnett’s multiple comparisons test demonstrated significant differences between most crude extract fractions and their corresponding positive control antibiotics (P < 0.05). However, the inhibition zone produced by fraction S4 against MRSA CCASU-8 and MDR *P. aeruginosa* CCASU-45 did not differ significantly from that of ciprofloxacin (P > 0.05), indicating comparable antibacterial activity. The statistical significance annotations are presented in **Table 2**. Among the tested extracts, S4 exhibited antimicrobial activity against all isolates tested. Based on these findings, the endophytic bacterial isolate responsible for producing S4, designated LS4, was selected for subsequent genomic profiling, while its crude extract was further investigated for cytotoxic and antiviral activities. The minimum inhibitory concentration (MIC) of the S4 extract is displayed in **Table 3** as the endpoint determinations from the broth microdilution assay, rather than mean ± SD values from quantitative measurements. Results showed promising antimicrobial activities against the tested MDR and standard bacterial pathogens.

**Table 2:** Results of zone inhibition of the four extracts (S1, S2, S3 and S4).

Microbial isolates	Inhibition zone (mm) ± SD							
	Endophytic extracts				Positive control			DMSO (negative control)
	S1	S2	S3	S4	Ciprofloxacin	Gentamicin	Fluconazole	
MRSA CCASU-5	14.3 ± 0.5^**^	14.3 ± 0.5^**^	15 ± 0.0^**^	18.6 ± 0.5^**^	26.3 ± 0.5	-	-	-
MRSA CCASU-8	11 ± 0.0^**^	9.6 ± 0.5^**^	10 ± 0.0^**^	24.3 ± 0.5^ns^	24.6± 0.33	-	-	-
MDR PA CCASU-28	-	-	-	19.6 ± 0.5^**^	34.4 ± 1.00	-	-	-
MDR PA CCASU-45	15.6 ± 0.5^**^	14.3 ± 0.0^**^	16.3 ± 0.5^**^	17 ± 0.0^ns^	33.3 ± 0.66	-	-	-
MDR PA CCASU-47	-	25 ± 0.0^**^	25 ± 0.0^**^	22.7 ± 0.5^**^	30.3 ± 0.33	-	-	-
MDR SA CCASU-4	-	12 ± 0.0^**^	12.3 ± 0.5^**^	23.6 ± 0.5^**^	28.0 ± 1.00	-	-	-
MDR SA CCASU-9	-	14 ± 0.0^**^	13.6 ± 0.5^**^	23.3 ± 0.5^**^	27.0 ± 0.00	-	-	-
MDR, KP CCASU-22	-	-	-	19.6 ± 0.5^**^		2.00 ± 0.00	-	-
MDR, KP CCASU-36	-	25 ± 0.0^**^	24.7 ± .05^**^	21.3 ± 0.5^**^		26.6 ± 0.33	-	-
*S. aureus* ATCC 25923	14 ± 0.5^**^	15 ± 0.5^**^	14 ± 0.5^**^	22.6 ± 0.5^**^	25.0 ± 0.00		-	-
*P. aeruginosa* ATCC 9027	12.2 ± 0.5^**^	17 ± 0.0^**^	19.6 ± 1.0^**^	15 ± 0.0^**^	32.6± 0.33		-	-
*C.albicans* ATCC 10231	13 ± 0.0^**^	16 ± 0.5^**^	14 ± .05^**^	11.6 ± 0.5^**^			37.0 ± 0.00	-

SD, Standard deviation. -, No inhibition zone was recorded; MDR, multidrug-resistant; MDR PA, MDR *Pseudomonas aeruginosa*; MDR SA, MDR *Staphylococcus aureus*; MDR KP, MDR *Klebsiella pneumoniae*; MRSA, methicillin-resistant *Staphylococcus aureus*; -, No MIC measurement was recorded; C. *albicans* ATCC 1023, *Candida albicans* ATCC 10231. Data are presented as mean ± SD (n = 3). Statistical significance was ^**^determined by one-way ANOVA followed by Dunnett’s multiple comparisons test using the corresponding positive control as the reference group. **P < 0.0001; ns, not significant.

**Table 3:** MIC measurement of the S4 extract against the tested pathogens.

Microbial isolates	MIC (µg/mL)			
	Endophytic extract S4	Positive control		
		Ciprofloxacin	Gentamicin	Fluconazole
MRSA CCASU-5	32	1.0	-	-
MRSA CCASU-8	16	1.0	-	-
MDR PA CCASU-28	32	0.5	-	-
MDR PA CCASU-45	64	0.5	-	-
MDR PA CCASU-47	8	0.25	-	-
MDR SA CCASU-4	16	1.0	-	-
MDR SA CCASU-9	32	1.0	-	-
MDR KP CCASU-22	64		2.0	-
MDR KP CCASU-36	32		2.0	-
*S. aureus* ATCC 25923	16	0.5		-
*P. aeruginosa* ATCC 9027	8	0.25		-
*C. albicans* ATCC 10231	16			1.0

MDR, multidrug-resistant; MDR PA, MDR *Pseudomonas aeruginosa*; MDR SA, MDR *Staphylococcus aureus*; MDR KP, MDR *Klebsiella pneumoniae*; MRSA, methicillin-resistant *Staphylococcus aureus*; -, No MIC measurement was recorded; *C. albicans* ATCC 1023, *Candida albicans* ATCC 10231.

### 3.3 Cytotoxicity evaluation

**Figure 1:**
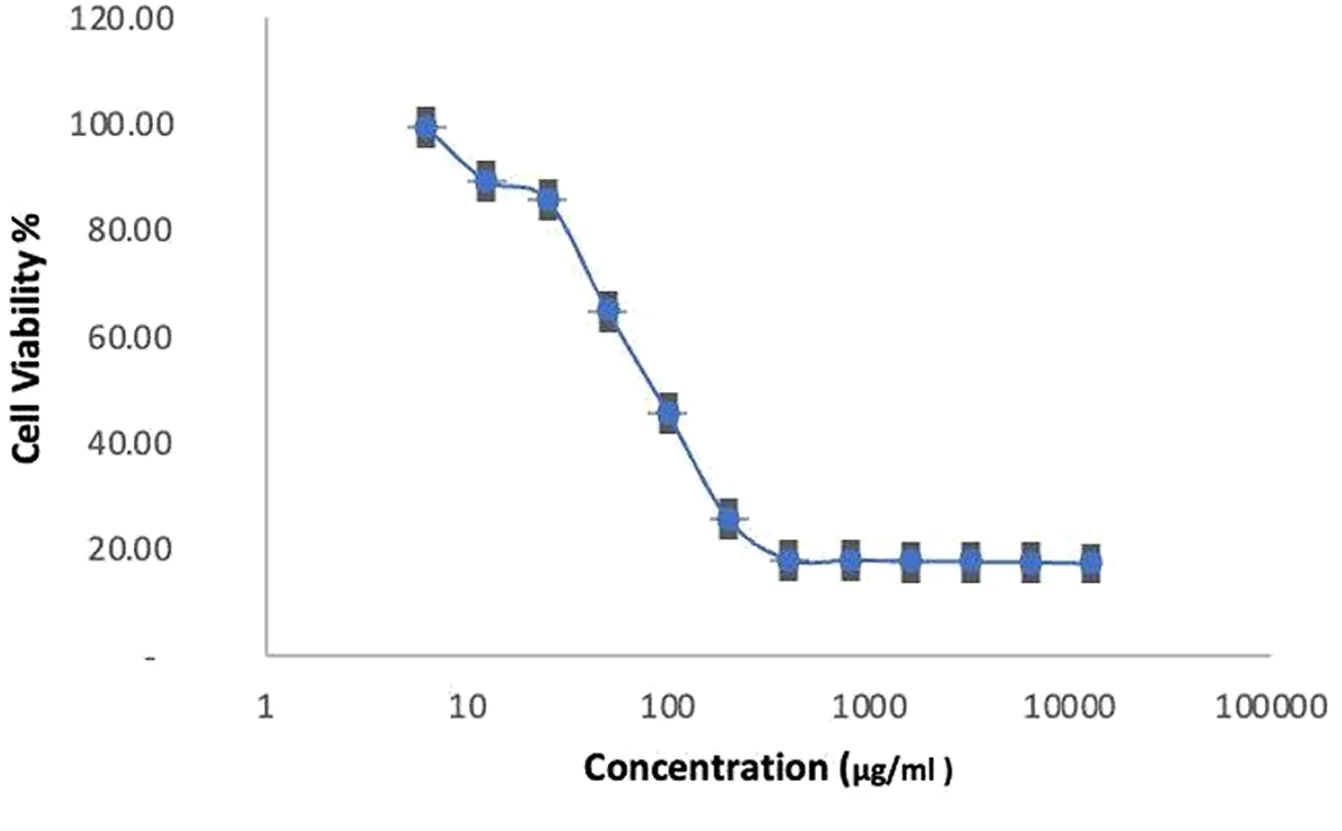
Percentage of viability for evaluating the cytotoxic activity of S4 organic extract.

The cytotoxic effect of the ethyl acetate extract S4 was evaluated on normal Vero cells using the MTT assay. The results indicated that the extract exhibited a CC50 of 80.71 μg/mL, whereas the non-cytotoxic concentration was 6.23 μg/mL (**Figure 1**). Representative morphological micrographs of untreated and extract-treated Vero cells are provided in **Supplementary Figure S2** to corroborate the MTT cytotoxicity findings.

### 3.4 Antiviral activity

Antiviral efficacy of the crude extract S4 was evaluated against three viruses, namely Adenovirus 7, HSV-1, and HSV-2. For Adenovirus 7, the untreated control exhibited a viral titer of 5.33 log_10_, whereas incubation with the S4 extract reduced the titer to 4.5 log_10_. This corresponded to a log reduction of.83, a viral reduction of 15.6%, and a reduction factor (RF) of 10^0.83^, suggesting slight antiviral activity in comparison with acyclovir, which produced a log reduction of 2.5 **Table 4a**. Against HSV-1, the untreated control demonstrated a viral titer of 7 log_10_. Treatment with S4 lowered the viral titer to 5 log_10_, corresponding to a log reduction of 2, a viral reduction of 28.6%, and an RF of 10^2^. These findings indicate moderate antiviral activity relative to acyclovir, which demonstrated a log reduction of 3.5 **Table 4b**.

**Table 4:** Antiviral assesment of the S4 extract against (a) HAdV-7, (b) HSV-1, and (c) HSV-2.

	Titre log (10)	TPT ^a^	Log diff ^b^	%red ^c^	RF ^d^
a. HAdV-7					
Acyclovir		2.83	2.5	46.9	10^2.5^
Adeno7	5.33				
S4 extract		4.5	0.83	15.6	10^0.83^
b. HSV-1					
Acyclovir		3.5	3.5	50	10^3.5^
HSV1	7				
S4 extract		5	2	28.6	10^2^
c. HSV-2					
Acyclovir		3.29	2.37	41.8	10^2.37^
HSV2	5.66				
S4 extract		4.66	1	17.7	10^1^

^a^TPT: titre post treatment, ^b^log diff: log difference between virus titre before and titre post treatment(log virus reduction factor), ^c^%red: reduction percentage, ^d^RF: reduction factor.

For HSV-2, the untreated control recorded a viral titer of 5.66 log_10_, while treatment with the crude extract S4 reduced the titer to 4.66 log_10_. This represented a log reduction of 1, a viral reduction of 17.7%, and an RF of 10^1^, reflecting slight antiviral activity compared with acyclovir, which showed a log reduction of 2.37 **Table 4c**.

### 3.5 Genomic analysis of LS4 isolate

**Figure 2:**
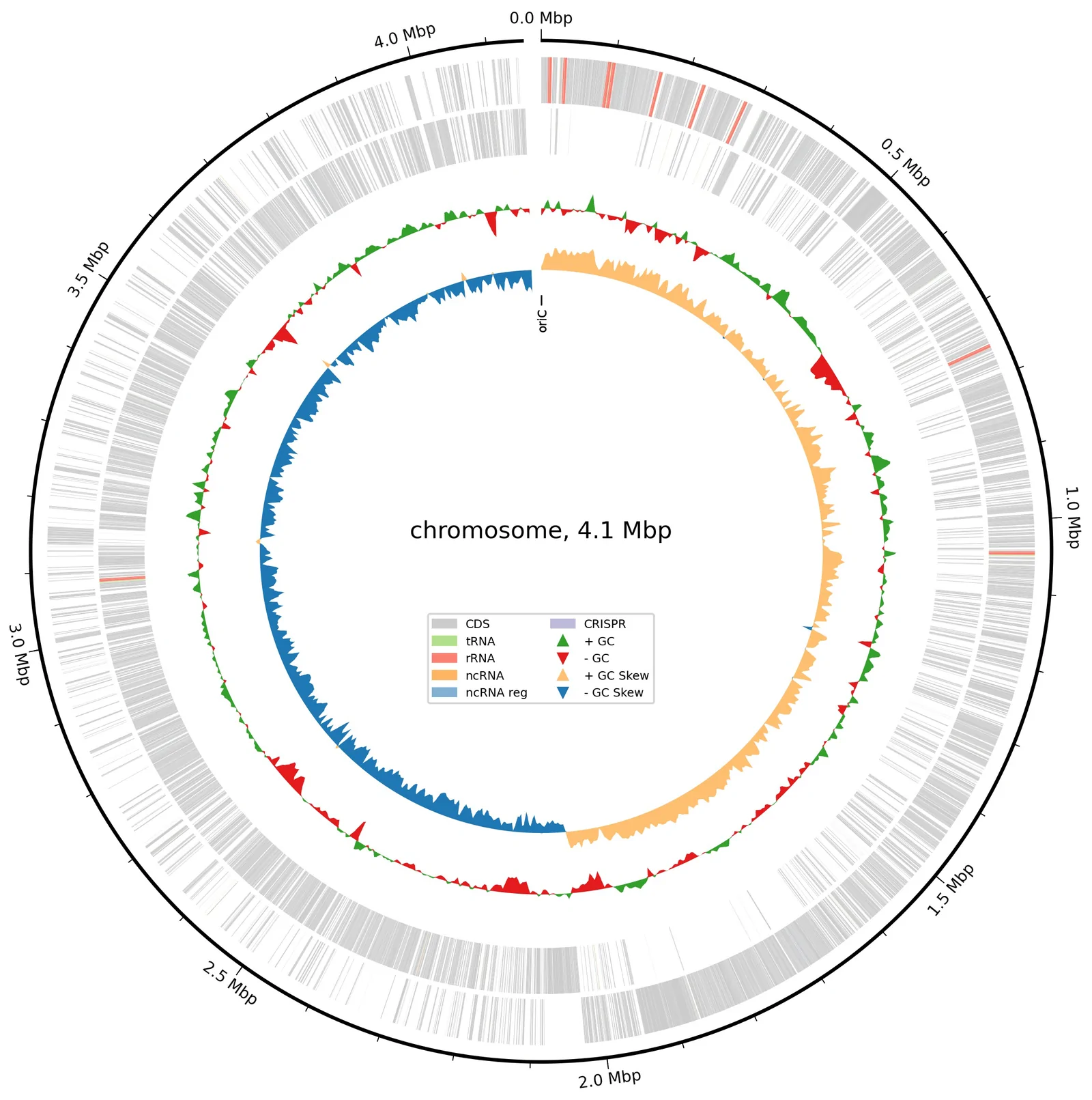
The genomic map of *Bacillus spizizenii* CCASU-2025–89 shows genomic segments of the coding sequences (CDS), various ribosomal RNAs (rRNA), and GC content.

The LS4 isolate was identified as *Bacillus spizizenii*. Its whole-genome sequence was deposited in the NCBI GenBank database under the BioProject accession number PRJNA1327104. In addition, the strain was preserved in the Culture Collection of Ain Shams University (CCASU) and assigned the accession number *Bacillus spizizenii* CCASU-2025-89. The genomic map of *Bacillus spizizenii* CCASU-2025–89 shows genomic segments of the coding sequences (CDS), various ribosomal RNAs (rRNA), and GC content is displayed in **Figure 2**. Genomic analysis of *Bacillus spizizenii* CCASU-2025–89 showed conserved biosynthetic gene clusters of the antimicrobial peptides including bacilysin (loci; 3806845-3816548), subtilin (loci; 3381216-3385629), mycosubtilin (loci; 2433630-2438118), bacillaene (loci; 1879418-1926411), bacillibactin (loci; 3201266-3208314), subtilosin A (loci; 3774824-3781761), and Pulcherriminic acid (loci; 3520532-3524691). The respective biosynthetic gene clusters are outlined in red in the additional **Supplementary File** (MS_assembly.tsv, **Supplementary 2**).

The full genome assembly statistics of *Bacillus spizizenii* CCASU-2025-89 (total contig number, genome size, GC content) and its comparison to the pan-genome sequences of *B. spizizenii* genome (Reference strain: *Bacillus spizizenii* (NCBI accession code, NC_016047.1)) are depicted in **Supplementary Figure 3**.

### 3.6 AntiSMASH analysis of the biosynthetic gene clusters in *Bacillus spizizenii* S4

**Figure 3:**
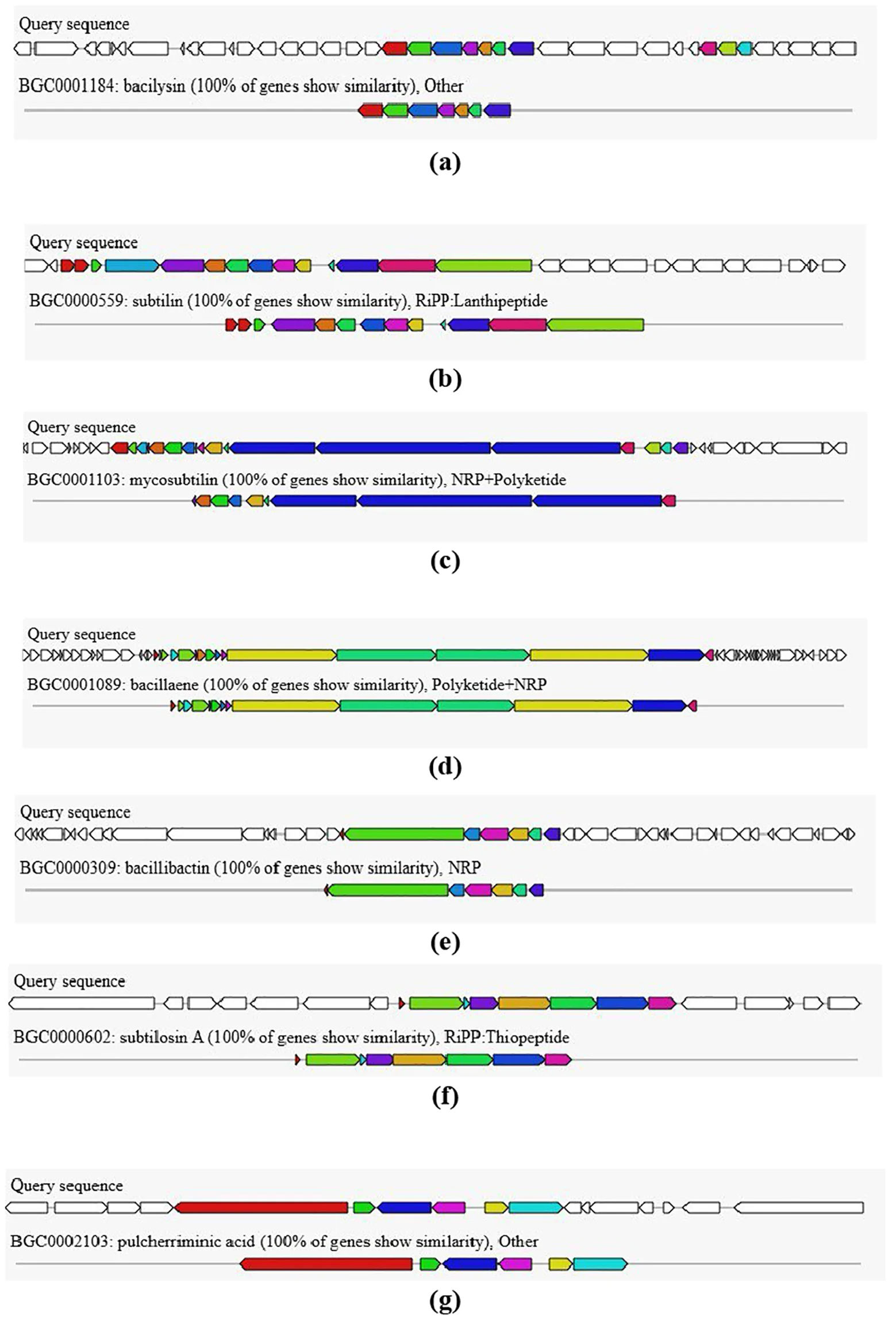
The biosynthetic gene clusters of *Bacillus spizizenii* CCASU-2025–89 identified using AntiSMASH for Bacilysin **(a)**, Subtilin **(b)**, Mycosubtilin **(c)**, Bacillaene **(d)**, Bacillibactin **(e)**, Subtilosin A **(f)**, and Pulcherriminic acid **(g)**. The conserved biosynthetic gene(s) are highlighted in color.

Whole-genome alignment and analysis of *Bacillus spizizenii* CCASU-2025–89 revealed gene clusters involved in secondary metabolite biosynthesis. Several biosynthetic gene clusters encoding significant secondary metabolites with possible antibacterial properties were identified by AntiSMASH investigation of the LS4 isolate, including bacilysin, subtilin, mycosubtilin, bacillaene, bacillibactin, subtilosin A, and pulcherriminic acid **Figure 3**. The bacilysin biosynthetic gene cluster exhibited 100% sequence identity with the bacilysin biosynthetic gene cluster of *Bacillus velezensis* FZB42 **Figure 3a**. The subtilin exhibited 100% sequence identity with the corresponding biosynthetic gene cluster identified in *Bacillus* spp. **Figure 3b**. The mycosubtilin biosynthetic gene cluster showed complete (100%) sequence identity with the corresponding gene cluster in *Bacillus subtilis* subsp. *spizizenii* ATCC 6633 **Figure 3c**. The bacillaene gene cluster showed 100% similarity to *Bacillus velezensis* FZB42 **Figure 3d**. The bacillibactin gene cluster showed 100% similarity to *Bacillus subtilis* subsp. *subtilis* str. 168 **Figure 3e**. The subtilosin A gene cluster showed 100% similarity to *Bacillus subtilis* subsp. *spizizenii* ATCC 6633 **Figure 3f**. The pulcherriminic acid gene cluster showed 100% similarity to *Bacillus subtilis* subsp. *subtilis* str. 168 **Figure 3g**. These findings suggest that the LS4 isolate has many biosynthetic gene clusters for secondary metabolites.

## 4 Discussion

Antimicrobial resistance represents a major global health challenge and necessitates the urgent need for novel therapeutic agents (Taati Moghadam et al., 2021). Consequently, natural sources, particularly plant-associated endophytic bacteria, have garnered significant attention as reservoirs of bioactive compounds (Mahdi et al., 2022). Endophytic bacteria are widely distributed within plant tissues and contribute to plant survival by mitigating various abiotic and biotic stresses, making them valuable sources of biologically active secondary metabolites (Afzal et al., 2019). This study was designed to evaluate the bioactive potential of endophytic bacteria isolated from *Solanum elaeagnifolium* Cav, focusing on their antimicrobial, antiviral, and genomic characteristics. The selected isolate, identified as *Bacillus spizizenii* CCASU-2025-89, exhibited notable biological activities, which were further supported by whole-genome analysis and biosynthetic gene cluster prediction. Among the tested extracts, the S4 extract demonstrated the broadest spectrum of antibacterial and antiviral activity. These results are in agreement with previous reports showing that *Bacillus spizizenii* and other *Bacillus* species isolated from environmental sources exhibit strong inhibitory effects against pathogens, highlighting the antimicrobial potential of *Bacillus*-derived metabolites (Geraldi et al., 2022).

The findings of the current study align with previous reports highlighting the antimicrobial potential of *Bacillus spizizenii*. Taggar et al. (2021) reported that *B. spizizenii* exhibits inhibitory activity against *S. aureus*. Similarly, *Bacillus subtilis* subsp. *spizizenii* isolated from marine environments has been shown to possess antifungal, biosurfactant, and bioemulsifier activities (Guillén-Navarro et al., 2023). Moreover, studies on *Bacillus subtilis* subsp. *spizizenii* DSM 15029T revealed the production of substantial amounts of unsuccinylated entianin, a metabolite with strong antibacterial activity (Fuchs et al., 2011). These observations align with our findings, where the selected isolate, *B. spizizenii* CCASU-2025-89, demonstrated broad-spectrum antibacterial activity as shown in **Table 2**. Collectively, these findings underscore the potential of *B. spizizenii* as a valuable source of bioactive secondary metabolites with pharmaceutical applications.

The cytotoxicity assessment of the crude extract obtained from *B. spizizenii* CCASU-2025–89 revealed a CC_50_ value of 80.71 μg/mL, indicating relatively low cytotoxicity toward normal Vero cells and suggesting that the extract is safe for further biological investigations. In antiviral activity, the S4 crude extract demonstrated inhibitory effects against HSV-1 and HSV-2. According to the European Standard EN 14476, a log reduction value of ≥1 is considered indicative of significant antiviral activity. Based on this criterion, the S4 extract exhibited antiviral effects against HSV-1 and HSV-2, with log reduction values of 2 and 1, respectively. In contrast, the activity against Adenovirus type 7 was lower (log reduction = 0.83), as shown in **Table 2**, which does not meet the threshold for significant antiviral efficacy. Genome mining showed the presence of many biosynthetic gene clusters that produce a variety of secondary metabolites with known biological functions. These metabolites may have contributed to the antiviral effects seen in this investigation, either individually or synergistically. Among these metabolites, subtilosin A has been reported to have antiviral activity against HSV-1 by inactivating viral particles and inhibiting viral replication. These findings are consistent with the antiviral effects observed in the present study (Torres et al., 2013). Furthermore, the discovery of the mycosubtilin biosynthetic gene cluster implies the potential production of membrane-active lipopeptides, which have been identified as promising candidates for antiviral drug development due to their ability to disrupt viral envelopes and modulate host responses (Isaia et al., 2025). Overall, the results indicate that the S4 crude extract possesses promising antiviral activity, particularly against herpes simplex viruses, and could serve as a potential candidate for further studies aimed at isolating and characterizing its active antiviral constituents.

The genomic analysis of *B. spizizenii* CCASU-2025–89 revealed that, the antimicrobial peptides bacilysin (loci 3806845-3816548), subtilin (loci 3381216-3385629), mycosubtilin (loci 2433630-2438118), bacillaene (loci 1879418-1926411), bacillibactin (loci 3201266-3208314), subtilosin A (loci 3774824-35) and pulcherriminic acid (loci 3520532-35) were found to have conserved biosynthesis gene clusters. The present genomic analysis revealed seven conserved (100% identity) antimicrobial biosynthetic gene clusters in the genomes of *B. Spizizenii*, CCASU-2025-89, as shown in **Figure 2**. The first gene cluster exhibited complete (100%) sequence identity with the biosynthetic gene cluster of bacilysin. Bacilysin is a dipeptide antibiotic, biosynthesized by aerobic spore-forming bacteria belonging to the genus *Bacillus* and exhibits lytic activity against both bacterial and fungal cells (Islam et al., 2022). *Bacillus amyloliquefaciens* FZB42 produces bacilysin, which is the most potent antibacterial agent. Bacilysin inhibits glucosamine 6-phosphate (GlcN6P) synthase, which facilitates the production of GlcN6P from fructose-6-phosphate and glutamine, a key component of the bacterial cell wall (Khan et al., 2016). These findings are consistent with previous reports indicating that compounds such as bacilysin contribute to the antimicrobial activity of *Bacillus* species through membrane disruption and iron competition mechanisms (Xiang et al., 2025). Therefore, bacilysin production by *B. spizizenii* CCASU-2025–89 has been confirmed by genomic analysis.

The second biosynthetic gene cluster exhibited complete (100%) sequence identity with the subtilin biosynthetic gene cluster, a lantibiotic naturally produced by *Bacillus subtilis* (Zhang et al., 2022). The first lantibiotic generated by a *Bacillus* strain to be mentioned (Entian and de Vos, 1996; Bierbaum and Sahl, 2009). Subtilin has nanomolar antibacterial activity against a variety of Gram-positive bacteria, such as *Micrococcus luteus, Lactococcus* spp., and *Bacillus spp* (Zhang et al., 2022). Subtilin interacts with the lipid II molecule to permeabilize the cell membrane and kill pathogens. This subtilin especially interacts with the bactoprenol hydrocarbon chain of the lipid II molecule, creating complexes that compromise the integrity of the cell membrane and cell wall by obstructing peptidoglycan formation in bacteria (Mercado and Olmos, 2022). Subtilin has demonstrated antibacterial activity against pathogens (Sutyak et al., 2008; Compaoreacute, 2013; Kumariya et al., 2019). Additionally, novel variants such as Subtilin L-Q11 have shown promising biophysical properties (Qin et al., 2019), and their production has been reported in multiple *Bacillus* strains (Helfrich et al., 2022; Zhang et al., 2022).

The third biosynthetic gene cluster exhibited 100% similarity to mycosubtilin. Mycosubtilin, which belongs to the iturin family, is considered the most powerful antifungal compound (Lin et al., 2022). Either by mycosubtilin clustering or lipopeptide aggregation, elevated mycosubtilin concentrations lyse the phospholipid layer. The release of metabolites and subsequent cell lysis is caused by this binding’s increase in membrane permeability. Additionally, mycosubtilin can trigger the signaling pathways for salicylic acid and jasmonic acid, which are implicated in the immune system’s reaction to infectious microbes (Tran et al., 2022). Mycosubtilin exhibits antifungal activity (Deravel et al., 2014; Mihalache et al., 2018; Besson et al., 1979) and has demonstrated strong inhibitory effects against *F. graminearum* and *F. verticillioides* (Yu et al., 2021). Importantly, lipopeptides such as mycosubtilin are known to interact with lipid membranes, which may explain the antiviral activity observed in the S4 extract, particularly against enveloped viruses such as HSV-1 and HSV-2 (Hoste et al., 2024).

The fourth biosynthetic gene cluster showing 100% similarity is bacillaene. *Bacillus subtilis* NCD-2 had the potential to produce secondary metabolites, including bacillaene (Su et al., 2020). Bacillaene is a bacteriostatic antibiotic that inhibits, rather than kills, its target. It is effective against a wide range of bacteria (Harwood et al., 2018) and suppresses bacterial growth by preventing prokaryotic protein synthesis (Su et al., 2020). *In vitro* studies showed that Bacillaene suppresses protein synthesis in bacteria. Cell survival experiments with *E. coli* strains reveal that the antibiotic is bacteriostatic (Patel et al., 1995). The fifth biosynthetic gene cluster showing 100% similarity is bacillibactin. Bacillibactin is a catecholamide siderophore produced by many *Bacillus* and other species. It exhibits antifungal and broad antibacterial activity. A study documented that bacillibactin produced by *B. subtilis* JSHY-K3 inhibited the growth of *Vp_AHPND_* (Xiang et al., 2025). Bacillibactin showed considerable antibacterial activity against drug-resistant test pathogens (Chakraborty et al., 2022). According to the study, the activity of bacillibactin is caused by direct antibiosis (Dimopoulou et al., 2021). Bacillibactin has the potential to directly inhibit several key enzymes in aquaculture pathogens, thereby affecting multiple bacterial pathways while providing a molecular basis for its potent antibacterial properties (Prazdnova et al., 2025).

The sixth biosynthetic gene cluster revealed 100% similarity to subtilosin A. Subtilosin A is a peptide antibiotic produced by *Bacillus* strains with documented antibacterial and probiotic-related properties (Alajlani, 2022; Parveen Rani et al., 2016). Its mode of action is that subtilosin A electrostatically binds to the plasma membrane and anchors to a membrane receptor. The transmembrane pH gradient is dissipated by this electrostatic interaction, which results in an intracellular ATP outflow that starves the cell and ultimately causes its death. Additionally, it has been demonstrated that subtilosin A prevents the production of biofilms, most likely by preventing quorum sensing between cells (Tran et al., 2022).

The last biosynthetic gene cluster identified in *B. spizizenii* showed 100% similarity to pulcherriminic acid. Pulcherriminic acid is a cyclic dipeptide found primarily in *Bacillus* and yeast (Yuan et al., 2020) and is classified as a natural biocontrol molecule belonging to the diketopiperazines (Gondry et al., 2009). Pulcherriminic acid prevents microbes from obtaining essential inorganic salts for growth, particularly iron, demonstrating its potential antimicrobial activity (Kántor et al., 2015). This compound contributes to microbial antagonism by altering iron availability in the surrounding environment (Lyng et al., 2024; Wang et al., 2023; Lyng et al., 2024). Therefore, its presence may play a role in the overall antimicrobial potential of B. spizizenii observed in this study.

## 5 Conclusion

The current study concludes by highlighting *Bacillus spizizenii* CCASU-2025-89’s strong biological potential as a source of bioactive metabolites. With comparatively low cytotoxicity, the S4 crude extract showed significant antiviral efficacy, especially against enveloped viruses like HSV-1 and HSV-2. Several antimicrobial biosynthesis gene clusters were also identified by genomic analysis, which may help explain the reported antiviral and antimicrobial effects. Thus, *Bacillus spizizenii* CCASU-2025–89 is a promising candidate for the creation of new antiviral and antimicrobial medicines, and more research is necessary to isolate, purify, and characterize its active chemicals.

## Data Availability

All data generated or analyzed during this study are included in this main article and **Supplementary File**. The whole-genome sequence was deposited in NCBI GenBank and assigned a BioProject accession code (PRJNA1327104) https://www.ncbi.nlm.nih.gov/bioproject/PRJNA1327104 and genome accession, SUB15626945. https://submit.ncbi.nlm.nih.gov/subs/wgs/SUB15626945/overview. The LS4 strain was maintained in the Culture Collection Ain Shams University (https://www.doi.org/10.12210/ccinfo.CCASU) and was designated the accession code Bacillıs spizizeni CCASU-2025-89.
