# Supplementary material for: Genomic insights and evaluation of Bacillus spizizenii Endophyte from Solanum elaeagnifolium Cav against multidrug-resistant pathogens
Source: Front Cell Infect Microbiol. 2026 Sep 3;16:1929148. doi: 10.3389/fcimb.2026.1929148 (PMC13581695; doi:10.3389/fcimb.2026.1929148)
Supplement: Supplementary file 1 [file DataSheet1.pdf]

MS_assembly.tsv Supplementray

# Annotated with Bakta							
# Software: v1.12.0							
# Database: v6.0, full							
# DOI: 10.1099/mgen.0.000685							
# URL: github.com/oschwengers/bakta							
#Sequence Id	Type	Start	Stop	Strand	Locus Tag	Gene	Product
contig_1	oriC	3	909	?			origin of replication
contig_1	cds	409	1749	+	GLNHAC_00001	dnaA	chromosomal replication initiator protein DnaA
contig_1	oriC	1770	1933	?			origin of replication
contig_1	cds	1938	3074	+	GLNHAC_00002	dnaN	DNA polymerase III subunit beta
contig_1	cds	3206	3421	+	GLNHAC_00003	rlbA	ribosome maturation protein RlbA
contig_1	cds	3437	4549	+	GLNHAC_00004	recF	DNA replication/repair protein RecF
contig_1	cds	4567	4812	+	GLNHAC_00005	remB	extracellular matrix regulator RemB
contig_1	cds	4861	6783	+	GLNHAC_00006	gyrB	DNA gyrase subunit B
contig_1	cds	6994	9459	+	GLNHAC_00007	gyrA	DNA gyrase subunit A
contig_1	rRNA	9740	11283	+	GLNHAC_00008	rrs	16S ribosomal RNA
contig_1	tRNA	11385	11461	+	GLNHAC_00009	trnI	tRNA-Ile(gat)
contig_1	tRNA	11473	11548	+	GLNHAC_00010	trnA	tRNA-Ala(tgc)
contig_1	rRNA	11630	14551	+	GLNHAC_00011	rrl	23S ribosomal RNA
contig_1	ncRNA	14613	14679	+	GLNHAC_00012	Bacillaceae-1	Bacillaceae-1 RNA
contig_1	rRNA	14686	14801	+	GLNHAC_00013	rrf	5S ribosomal RNA
contig_1	cds	14841	15827	-	GLNHAC_00014	yaaC	Uncharacterized protein YaaC
contig_1	cds	15909	17375	+	GLNHAC_00015	guaB	inosine 5'-monophosphate dehydrogenase
contig_1	cds	17528	18859	+	GLNHAC_00016	dacA	D-alanyl-D-alanine carboxypeptidase
contig_1	cds	19056	19940	+	GLNHAC_00017	pdxS	pyridoxal 5'-phosphate synthase subunit PdxS
contig_1	cds	19962	20552	+	GLNHAC_00018	pdxT	pyridoxal 5'-phosphate synthase subunit PdxT
contig_1	ncRNA-region	20605	20817	+			T-box leader
contig_1	cds	20873	22150	+	GLNHAC_00019	serS	serine--tRNA ligase
contig_1	tRNA	22285	22377	+	GLNHAC_00020	trnS	tRNA-Ser(tga)
contig_1	cds	22489	23142	-	GLNHAC_00021	dck	deoxyadenosine/deoxycytidine kinase
contig_1	cds	23139	23762	-	GLNHAC_00022	dgk	deoxyguanosine kinase
contig_1	cds	23861	25144	-	GLNHAC_00023	sleL	Cortical fragment-lytic enzyme
contig_1	cds	25214	25759	-	GLNHAC_00024	yaaI	Uncharacterized isochorismatase family protein YaaI
contig_1	cds	25845	26330	+	GLNHAC_00025	tadA	tRNA adenosine(34) deaminase TadA
contig_1	ncRNA	26413	26677	+	GLNHAC_00026	Bacteria_large_SRP	Bacterial large signal recognition particle RNA
contig_1	ncRNA	26527	26626	+	GLNHAC_00027	Bacteria_small_SRP	Bacterial small signal recognition particle RNA
contig_1	cds	26807	28498	+	GLNHAC_00028	dnaX	DNA polymerase III subunit gamma/tau
contig_1	cds	28522	28845	+	GLNHAC_00029		YbaB/EbfC family nucleoid-associated protein
contig_1	cds	28860	29456	+	GLNHAC_00030	recR	recombination protein RecR
contig_1	cds	29474	29590	+	GLNHAC_00031		hypothetical protein
contig_1	cds	29765	29989	+	GLNHAC_00032	bofA	Sigma-K factor-processing regulatory protein BofA
contig_1	ncRNA	30272	30337	-	GLNHAC_00033	Bacillaceae-1	Bacillaceae-1 RNA
contig_1	rRNA	30332	31871	+	GLNHAC_00034	rrs	16S ribosomal RNA
contig_1	tRNA	31970	32043	+	GLNHAC_00035	trnI	tRNA-Ile(gat)
contig_1	tRNA	32054	32129	+	GLNHAC_00036	trnA	tRNA-Ala(tgc)
contig_1	rRNA	32211	35139	+	GLNHAC_00037	rrl	23S ribosomal RNA
contig_1	rRNA	35195	35310	+	GLNHAC_00038	rrf	5S ribosomal RNA
contig_1	cds	35489	35683	+	GLNHAC_00039	csfB	anti-sigma-G factor
contig_1	cds	35816	36430	+	GLNHAC_00040		5-bromo-4-chloroindolyl phosphate hydrolysis family protein
contig_1	cds	36449	37606	+	GLNHAC_00041	yaaN	Uncharacterized protein YaaN
contig_1	cds	37688	39130	+	GLNHAC_00042	yaaO	Uncharacterized protein YaaO
contig_1	cds	39127	39765	+	GLNHAC_00043	tmk	dTMP kinase
contig_1	cds	39840	40169	+	GLNHAC_00044	darA	cyclic di-AMP receptor DarA
contig_1	cds	40182	40622	+	GLNHAC_00045	yaaR	Uncharacterized protein YaaR
contig_1	cds	40655	41623	+	GLNHAC_00046	holB	DNA polymerase III subunit delta'
contig_1	cds	41626	42453	+	GLNHAC_00047	yaaT	competence/sporulation regulator complex protein RicT
contig_1	cds	42468	42827	+	GLNHAC_00048	yabA	replication initiation-control protein YabA
contig_1	cds	42886	43629	+	GLNHAC_00049	yabB	putative RNA methyltransferase YabB
contig_1	cds	43616	44767	+	GLNHAC_00050		Ribosomal RNA small subunit methyltransferase I
contig_1	cds	44817	45101	-	GLNHAC_00051	abrB	transition state genes transcriptional regulator AbrB
contig_1	cds	45602	46825	+	GLNHAC_00052		Methionine--tRNA ligase
contig_1	cds	46822	47595	+	GLNHAC_00053		Methionine--tRNA ligase subunit beta
contig_1	cds	47674	48441	+	GLNHAC_00054	yabD	Uncharacterized metal-dependent hydrolase YabD
contig_1	cds	48687	49910	+	GLNHAC_00055	yabE	Putative cell wall shaping protein YabE
contig_1	cds	50055	50615	+	GLNHAC_00056	rnmV	ribonuclease M5
contig_1	cds	50608	51486	+	GLNHAC_00057	rsmA	16S rRNA (adenine(1518)-N(6)/adenine(1519)-N(6))-dimethyltr
contig_1	cds	51629	52489	+	GLNHAC_00058	yabG	sporulation-specific protease PrtG
contig_1	cds	52729	52899	+	GLNHAC_00059		hypothetical protein
contig_1	cds	52994	53332	+	GLNHAC_00060		hypothetical protein
contig_1	cds	53479	54348	+	GLNHAC_00061	ispE	4-(cytidine 5'-diphospho)-2-C-methyl-D-erythritol kinase
contig_1	cds	54404	55261	+	GLNHAC_00062	purR	pur operon repressor
contig_1	cds	55258	55635	+	GLNHAC_00063		2-iminobutanoate/2-iminopropanoate deaminase
contig_1	cds	55831	56124	+	GLNHAC_00064	spoVG	septation regulator SpoVG
contig_1	cds	56317	57687	+	GLNHAC_00065	glmU	bifunctional UDP-N-acetylglucosamine diphosphorylase/gluc
contig_1	cds	57710	58663	+	GLNHAC_00066	prs	ribose-phosphate diphosphokinase
contig_1	cds	58748	59365	+	GLNHAC_00067	ctc	50S ribosomal protein L25/general stress protein Ctc
contig_1	cds	59473	60039	+	GLNHAC_00068	pth	aminoacyl-tRNA hydrolase
contig_1	cds	60099	60329	+	GLNHAC_00069	fin	anti-sigma-F factor Fin
contig_1	cds	60399	63932	+	GLNHAC_00070	mfd	transcription-repair coupling factor
contig_1	cds	64067	64171	+	GLNHAC_00071		(pseudo) AbrB/MazE/SpoVT family DNA-binding domain-cont
contig_1	cds	64203	64604	+	GLNHAC_00072		Stage V sporulation protein T
contig_1	cds	64787	66385	+	GLNHAC_00073	yabM	Uncharacterized membrane protein YabM
contig_1	cds	66375	67844	+	GLNHAC_00074	mazG	nucleoside triphosphate pyrophosphohydrolase
contig_1	cds	67847	68107	+	GLNHAC_00075		RNA-binding S4 domain-containing protein
contig_1	cds	68186	68488	+	GLNHAC_00076	yabP	sporulation protein YabP
contig_1	cds	68485	69120	+	GLNHAC_00077	yabQ	spore cortex biosynthesis protein YabQ
contig_1	cds	69138	69515	+	GLNHAC_00078	divIC	cell division protein DivIC
contig_1	cds	69596	69982	+	GLNHAC_00079	yabR	S1 domain-containing RNA-binding protein
contig_1	tRNA	70151	70227	+	GLNHAC_00080	trnM	tRNA-Met(cat)
contig_1	tRNA	70237	70308	+	GLNHAC_00081	trnE	tRNA-Glu(ttc)
contig_1	cds	70508	72991	+	GLNHAC_00082	spoIIE	stage II sporulation protein E
contig_1	cds	73076	73813	+	GLNHAC_00083	yabS	Uncharacterized protein YabS
contig_1	cds	73779	74798	+	GLNHAC_00084	yabT	serine/threonine protein kinase PrkT
contig_1	cds	74902	76320	+	GLNHAC_00085	tilS	tRNA lysidine(34) synthetase TilS
contig_1	cds	76317	76757	+	GLNHAC_00086		Hypoxanthine phosphoribosyltransferase
contig_1	cds	76958	78871	+	GLNHAC_00087	ftsH	ATP-dependent zinc metalloprotease FtsH
contig_1	cds	79065	79841	+	GLNHAC_00088	coaX	type III pantothenate kinase
contig_1	cds	79853	80728	+	GLNHAC_00089	hslO	redox-regulated molecular chaperone HslO
contig_1	cds	80776	81669	+	GLNHAC_00090	yacD	Putative peptidyl-prolyl cis-trans isomerase YacD
contig_1	cds	81745	82671	+	GLNHAC_00091	cysK	cysteine synthase A
contig_1	cds	82835	84535	+	GLNHAC_00092	pabB	Aminodeoxychorismate synthase component 1
contig_1	cds	84553	84840	+	GLNHAC_00093		Glutamine amidotransferase domain-containing protein
contig_1	cds	84840	85721	+	GLNHAC_00094	pabC	aminodeoxychorismate lyase
contig_1	cds	85703	86494	+	GLNHAC_00095	folP	dihydropteroate synthase
contig_1	cds	86443	86643	+	GLNHAC_00096		hypothetical protein
contig_1	cds	86606	86749	+	GLNHAC_00097		hypothetical protein
contig_1	cds	86761	86889	+	GLNHAC_00098		hypothetical protein
contig_1	cds	86984	87391	+	GLNHAC_00099	folK	2-amino-4-hydroxy-6-hydroxymethyldihydropteridine diphosp
contig_1	cds	87601	87987	+	GLNHAC_00100	dusB	(pseudo) tRNA dihydrouridine synthase DusB
contig_1	cds	87987	88598	+	GLNHAC_00101		tRNA-dihydrouridine synthase
contig_1	cds	88690	90189	+	GLNHAC_00102	lysS	lysine--tRNA ligase
contig_1	rRNA	90533	92075	+	GLNHAC_00103	rrs	16S ribosomal RNA
contig_1	rRNA	92248	95176	+	GLNHAC_00104	rrl	23S ribosomal RNA
contig_1	rRNA	95231	95346	+	GLNHAC_00105	rrf	5S ribosomal RNA
contig_1	tRNA	95368	95443	+	GLNHAC_00106	trnV	tRNA-Val(tac)
contig_1	tRNA	95448	95523	+	GLNHAC_00107	trnT	tRNA-Thr(tgt)
contig_1	tRNA	95561	95636	+	GLNHAC_00108	trnK	tRNA-Lys(ttt)
contig_1	tRNA	95643	95725	+	GLNHAC_00109	trnL	tRNA-Leu(tag)
contig_1	tRNA	95766	95840	+	GLNHAC_00110	trnG	tRNA-Gly(gcc)
contig_1	tRNA	95855	95940	+	GLNHAC_00111	trnL	tRNA-Leu(taa)
contig_1	tRNA	95950	96026	+	GLNHAC_00112	trnR	tRNA-Arg(acg)
contig_1	tRNA	96053	96128	+	GLNHAC_00113	trnP	tRNA-Pro(tgg)
contig_1	tRNA	96136	96207	+	GLNHAC_00114	trnA	tRNA-Ala(tgc)
contig_1	rRNA	96388	97926	+	GLNHAC_00115	rrs	16S ribosomal RNA
contig_1	rRNA	98096	101007	+	GLNHAC_00116	rrl	23S ribosomal RNA
contig_1	rRNA	101061	101175	+	GLNHAC_00117	rrf	5S ribosomal RNA
contig_1	cds	101421	101885	+	GLNHAC_00118	ctsR	transcriptional regulator CtsR
contig_1	cds	102109	102456	+	GLNHAC_00119		UvrB/UvrC motif-containing protein
contig_1	cds	102456	103547	+	GLNHAC_00120	mcsB	protein arginine kinase
contig_1	cds	103544	105976	+	GLNHAC_00121	clpC	ATP-dependent protease ATP-binding subunit ClpC
contig_1	cds	106011	107444	+	GLNHAC_00122	radA	DNA repair protein RadA
contig_1	cds	107448	108530	+	GLNHAC_00123	disA	DNA integrity scanning diadenylate cyclase DisA
contig_1	cds	108645	109745	+	GLNHAC_00124	yacL	Uncharacterized PIN and TRAM-domain containing protein Ya
contig_1	cds	109759	110457	+	GLNHAC_00125	ispD	2-C-methyl-D-erythritol 4-phosphate cytidylyltransferase
contig_1	cds	110450	110926	+	GLNHAC_00126	ispF	2-C-methyl-D-erythritol 2,4-cyclodiphosphate synthase
contig_1	cds	111017	112468	+	GLNHAC_00127	gltX	glutamate--tRNA ligase
contig_1	ncRNA-region	112471	112701	+			T-box leader

contig_1	cds	112772	113425	+	GLNHAC_00128	cysE	serine O-acetyltransferase
contig_1	cds	113422	114822	+	GLNHAC_00129	cysS	cysteine--tRNA ligase
contig_1	cds	114826	115257	+	GLNHAC_00130	mrnC	mini-ribonuclease 3
contig_1	cds	115241	115990	+	GLNHAC_00131	rlmB	23S rRNA (guanosine(2251)-2'-O)-methyltransferase RlmB
contig_1	cds	115997	116509	+	GLNHAC_00132	rae1	ribosome-dependent mRNA decay endonuclease Rae1/YacP
contig_1	cds	116572	117228	+	GLNHAC_00133	sigH	RNA polymerase sporulation sigma factor SigH
contig_1	cds	117504	117683	+	GLNHAC_00134	secE	preprotein translocase subunit SecE
contig_1	cds	117863	118396	+	GLNHAC_00135	nusG	transcription termination/antitermination protein NusG
contig_1	cds	118564	118989	+	GLNHAC_00136	rplK	50S ribosomal protein L11
contig_1	cds	119084	119782	+	GLNHAC_00137	rplA	50S ribosomal protein L1
contig_1	ncRNA-region	119828	119968	+			Ribosomal protein L10 leader
contig_1	cds	120034	120534	+	GLNHAC_00138	rplJ	50S ribosomal protein L10
contig_1	cds	120580	120951	+	GLNHAC_00139	rplL	50S ribosomal protein L7/L12
contig_1	cds	121041	121646	+	GLNHAC_00140	ybxB	Uncharacterized protein YbxB
contig_1	cds	121893	124223	+	GLNHAC_00141		DNA-directed RNA polymerase subunit beta
contig_1	cds	124220	125473	+	GLNHAC_00142	rpoB	(pseudo) DNA-directed RNA polymerase subunit beta
contig_1	cds	125535	129134	+	GLNHAC_00143	rpoC	DNA-directed RNA polymerase subunit beta'
contig_1	cds	129310	129558	+	GLNHAC_00144	rulS	50S ribosomal protein L7ae-like protein
contig_1	cds	129672	129830	+	GLNHAC_00145		hypothetical protein
contig_1	cds	129827	130087	+	GLNHAC_00146	rpsL	30S ribosomal protein S12
contig_1	cds	130129	130599	+	GLNHAC_00147	rpsG	30S ribosomal protein S7
contig_1	cds	130652	132730	+	GLNHAC_00148	fusA	elongation factor G
contig_1	cds	132850	133071	+	GLNHAC_00149		Elongation factor Tu
contig_1	cds	133055	134041	+	GLNHAC_00150	tuf	elongation factor Tu
contig_1	cds	134142	135098	+	GLNHAC_00151		Proline iminopeptidase
contig_1	cds	135336	135644	+	GLNHAC_00152	rpsJ	30S ribosomal protein S10
contig_1	cds	135684	136313	+	GLNHAC_00153	rplC	50S ribosomal protein L3
contig_1	cds	136341	136964	+	GLNHAC_00154	rplD	50S ribosomal protein L4
contig_1	cds	136964	137251	+	GLNHAC_00155	rplW	50S ribosomal protein L23
contig_1	cds	137283	138116	+	GLNHAC_00156	rplB	50S ribosomal protein L2
contig_1	cds	138174	138452	+	GLNHAC_00157	rpsS	30S ribosomal protein S19
contig_1	cds	138469	138810	+	GLNHAC_00158	rplV	50S ribosomal protein L22
contig_1	cds	138814	139470	+	GLNHAC_00159	rpsC	30S ribosomal protein S3
contig_1	cds	139472	139906	+	GLNHAC_00160	rplP	50S ribosomal protein L16
contig_1	cds	139896	140096	+	GLNHAC_00161	rpmC	50S ribosomal protein L29
contig_1	cds	140119	140382	+	GLNHAC_00162	rpsQ	30S ribosomal protein S17
contig_1	cds	140423	140791	+	GLNHAC_00163	rplN	50S ribosomal protein L14
contig_1	cds	140828	141139	+	GLNHAC_00164	rplX	50S ribosomal protein L24
contig_1	cds	141166	141705	+	GLNHAC_00165	rplE	50S ribosomal protein L5
contig_1	cds	141728	141913	+	GLNHAC_00166	rpsN	30S ribosomal protein S14
contig_1	cds	141945	142343	+	GLNHAC_00167	rpsH	30S ribosomal protein S8
contig_1	cds	142373	142912	+	GLNHAC_00168	rplF	50S ribosomal protein L6
contig_1	cds	142945	143307	+	GLNHAC_00169	rplR	50S ribosomal protein L18
contig_1	cds	143326	143832	+	GLNHAC_00170	rpsE	30S ribosomal protein S5
contig_1	cds	143846	144025	+	GLNHAC_00171	rpmD	50S ribosomal protein L30
contig_1	cds	144056	144496	+	GLNHAC_00172	rplO	50S ribosomal protein L15
contig_1	cds	144498	145793	+	GLNHAC_00173	secY	preprotein translocase subunit SecY
contig_1	cds	145848	146501	+	GLNHAC_00174	adk	adenylate kinase
contig_1	cds	146498	147244	+	GLNHAC_00175	map	type I methionyl aminopeptidase
contig_1	cds	147555	147773	+	GLNHAC_00176	infA	translation initiation factor IF-1
contig_1	cds	147807	147920	+	GLNHAC_00177	rpmJ	50S ribosomal protein L36
contig_1	cds	147943	148308	+	GLNHAC_00178	rpsM	30S ribosomal protein S13
contig_1	cds	148329	148724	+	GLNHAC_00179	rpsK	30S ribosomal protein S11
contig_1	cds	148901	149845	+	GLNHAC_00180	rpoA	DNA-directed RNA polymerase subunit alpha
contig_1	cds	149923	150285	+	GLNHAC_00181	rplQ	50S ribosomal protein L17
contig_1	cds	150415	151260	+	GLNHAC_00182	ecfA	energy-coupling factor ABC transporter ATP-binding protein
contig_1	cds	151236	152105	+	GLNHAC_00183	ecfAB	energy-coupling factor ABC transporter ATP-binding protein
contig_1	cds	152102	152899	+	GLNHAC_00184	ecfT	Energy-coupling factor transporter transmembrane protein Ec
contig_1	cds	152909	153652	+	GLNHAC_00185	truA	tRNA pseudouridine(38-40) synthase TruA
contig_1	ncRNA-region	153709	153765	+			Ribosomal protein L13 leader
contig_1	cds	153814	154251	+	GLNHAC_00186	rplM	50S ribosomal protein L13
contig_1	cds	154272	154664	+	GLNHAC_00187	rpsI	30S ribosomal protein S9
contig_1	cds	155140	155907	+	GLNHAC_00188	ybaJ	Uncharacterized methyltransferase YbaJ
contig_1	cds	156103	156546	+	GLNHAC_00189	ybaK	Uncharacterized protein YbaK
contig_1	cds	156606	157319	+	GLNHAC_00190	cwlD	N-acetylmuramoyl-L-alanine amidase CwlD
contig_1	cds	157415	158473	+	GLNHAC_00191	salA	iron-sulfur carrier protein/transcriptional regulator SalA
contig_1	cds	158511	159068	-	GLNHAC_00192	gerD	spore germination protein GerD
contig_1	cds	159344	159772	+	GLNHAC_00193	kbaA	(pseudo) KinB-signaling pathway activation protein
contig_1	cds	159773	160192	-	GLNHAC_00194		polysaccharide deacetylase family protein
contig_1	rRNA	160885	162430	+	GLNHAC_00195	rrs	16S ribosomal RNA
contig_1	rRNA	162600	165527	+	GLNHAC_00196	rrl	23S ribosomal RNA
contig_1	rRNA	165582	165697	+	GLNHAC_00197	rrf	5S ribosomal RNA
contig_1	tRNA	165742	165816	+	GLNHAC_00198	trnN	tRNA-Asn(gtt)
contig_1	tRNA	165819	165891	+	GLNHAC_00199	trnT	tRNA-Thr(ggt)
contig_1	cds	165998	167182	-	GLNHAC_00200		Site-specific recombinase, phage integrase family protein
contig_1	cds	167169	167702	-	GLNHAC_00201	irrE	IrrE N-terminal-like domain-containing protein
contig_1	cds	167789	168454	-	GLNHAC_00202		DUF4352 domain-containing protein
contig_1	cds	168700	169029	-	GLNHAC_00203		helix-turn-helix transcriptional regulator
contig_1	cds	169190	169423	+	GLNHAC_00204		Helix-turn-helix, putative
contig_1	cds	169436	169627	+	GLNHAC_00205		helix-turn-helix domain-containing protein
contig_1	cds	169692	170225	+	GLNHAC_00206		Bacteriophage-related protein
contig_1	cds	170215	170475	+	GLNHAC_00207		Prophage MuSo1, F protein, putative
contig_1	cds	170476	170640	+	GLNHAC_00208		hypothetical protein
contig_1	cds	171060	171245	+	GLNHAC_00209		hypothetical protein
contig_1	cds	171242	173215	+	GLNHAC_00210		Nuclease SbcCD subunit C
contig_1	cds	173258	174016	+	GLNHAC_00211	bet	phage recombination protein Bet
contig_1	cds	174013	174714	+	GLNHAC_00212		Metallo-beta-lactamase domain-containing protein
contig_1	cds	174711	174890	+	GLNHAC_00213		hypothetical protein
contig_1	cds	174913	175668	+	GLNHAC_00214		Replication protein
contig_1	cds	175677	175967	+	GLNHAC_00215		Replicative helicase inhibitor G39P N-terminal domain-contai
contig_1	cds	175942	177243	+	GLNHAC_00216	dnaB	replicative DNA helicase
contig_1	cds	177262	177456	+	GLNHAC_00217	fbpA	Fur-regulated basic protein FbpA
contig_1	cds	177456	177551	+	GLNHAC_00218		hypothetical protein
contig_1	cds	177557	178012	+	GLNHAC_00219	rusA	Endodeoxyribonuclease RusA
contig_1	cds	178220	178399	+	GLNHAC_00220		hypothetical protein
contig_1	cds	178469	178672	+	GLNHAC_00221		Phage protein
contig_1	cds	178714	179001	+	GLNHAC_00222		VWA domain-containing protein
contig_1	cds	178998	179219	+	GLNHAC_00223		hypothetical protein
contig_1	cds	179296	179751	+	GLNHAC_00224		hypothetical protein
contig_1	cds	179824	180210	+	GLNHAC_00225	arpU	Phage transcriptional regulator, ArpU family subfamily
contig_1	cds	180450	181496	+	GLNHAC_00226		Oxidoreductase, FAD/FMN-binding protein
contig_1	cds	181574	182107	-	GLNHAC_00227		Glyoxalase family protein
contig_1	cds	182139	182534	-	GLNHAC_00228	doxX	DoxX
contig_1	cds	182848	183261	+	GLNHAC_00229	marR	MarR family winged helix-turn-helix transcriptional regulator
contig_1	cds	183869	184036	+	GLNHAC_00230		Phage protein
contig_1	cds	184131	184475	+	GLNHAC_00231		Structural protein
contig_1	cds	184543	185001	+	GLNHAC_00232		SMODS and SLOG-associating 2TM effector domain-contain
contig_1	cds	185057	185578	+	GLNHAC_00233		DNA methylase
contig_1	cds	185589	186608	+	GLNHAC_00234		DNA (cytosine-5-)-methyltransferase
contig_1	cds	186659	187426	+	GLNHAC_00235		DUF4145 domain-containing protein
contig_1	cds	187581	187829	+	GLNHAC_00236	copG	CopG family transcriptional regulator
contig_1	cds	187877	188755	+	GLNHAC_00237	terS	phage terminase small subunit
contig_1	cds	188752	190077	+	GLNHAC_00238		PBSX family phage terminase large subunit
contig_1	cds	190070	191602	+	GLNHAC_00239		phage portal protein
contig_1	cds	191599	192516	+	GLNHAC_00240		Phage Mu protein F like protein
contig_1	cds	192560	193204	+	GLNHAC_00241		DUF4145 domain-containing protein
contig_1	cds	193233	194414	+	GLNHAC_00242	xkdF	Phage-like element PBSX protein XkdF domain-containing pr
contig_1	cds	194446	195381	+	GLNHAC_00243		Phage major capsid protein
contig_1	cds	195392	195709	+	GLNHAC_00244	yqbF	Uncharacterized protein YqbF
contig_1	cds	195715	196107	+	GLNHAC_00245	yqbG	Protein YqbG
contig_1	cds	196104	196466	+	GLNHAC_00246		DUF3599 domain-containing protein
contig_1	cds	196463	196966	+	GLNHAC_00247	yqbI	Uncharacterized protein YqbI
contig_1	cds	196979	197416	+	GLNHAC_00248		Phage portal protein
contig_1	cds	197413	197604	+	GLNHAC_00249		Phage protein
contig_1	cds	197605	199005	+	GLNHAC_00250	yqbK	Uncharacterized protein YqbK
contig_1	cds	199007	199450	+	GLNHAC_00251	xkdM	Phage-like element PBSX protein XkdM
contig_1	ncRNA	199629	199904	-	GLNHAC_00252	bsrG	BsrG
contig_1	cds	200073	200522	+	GLNHAC_00253	yqbN	Uncharacterized protein YqbN
contig_1	cds	200546	200701	+	GLNHAC_00254	xkdN	XkdN
contig_1	cds	200704	206046	+	GLNHAC_00255		TMP repeat family
contig_1	cds	206039	206698	+	GLNHAC_00256	yqbP	Uncharacterized protein YqbP
contig_1	cds	206711	207691	+	GLNHAC_00257	yqbQ	Uncharacterized protein YqbQ
contig_1	cds	207688	207954	+	GLNHAC_00258		DUF2577 domain-containing protein
contig_1	cds	207967	208392	+	GLNHAC_00259	yqbS	Uncharacterized protein YqbS
contig_1	cds	208385	209431	+	GLNHAC_00260	yqbT	Uncharacterized protein YqbT
contig_1	cds	209415	209993	+	GLNHAC_00261	yqcA	Uncharacterized protein YqcA
contig_1	cds	209990	210262	+	GLNHAC_00262	yqcB	Uncharacterized protein YqcB
contig_1	cds	210266	212020	+	GLNHAC_00263		Terminase
contig_1	cds	212032	212361	+	GLNHAC_00264	yorD	Bacteriophage SP-beta YorD domain-containing protein

contig_1	cds	212358	212522	+	GLNHAC_00265	yqcE	XkdX family protein
contig_1	cds	212614	213507	+	GLNHAC_00266		Phage portal protein
contig_1	cds	213553	213975	+	GLNHAC_00267		Holin family
contig_1	cds	214020	214994	+	GLNHAC_00268		N-acetylmuramoyl-L-alanine amidase
contig_1	cds	215033	215317	-	GLNHAC_00269		Group-specific protein
contig_1	cds	215323	217035	-	GLNHAC_00270	ywqJ	YwqJ
contig_1	cds	217245	218378	+	GLNHAC_00271		Response regulator aspartate phosphatase B
contig_1	cds	218368	218526	+	GLNHAC_00272		hypothetical protein
contig_1	cds	218558	218731	-	GLNHAC_00273		YolD-like family protein
contig_1	cds	218953	220008	-	GLNHAC_00274		Abortive infection protein
contig_1	tRNA	220815	220889	+	GLNHAC_00275	trnG	tRNA-Gly(gcc)
contig_1	tRNA	220922	220998	+	GLNHAC_00276	trnR	tRNA-Arg(acg)
contig_1	tRNA	221025	221101	+	GLNHAC_00277	trnP	tRNA-Pro(tgg)
contig_1	tRNA	221109	221184	+	GLNHAC_00278	trnA	tRNA-Ala(tgc)
contig_1	rRNA	221358	222907	+	GLNHAC_00279	rrs	16S ribosomal RNA
contig_1	rRNA	223077	226004	+	GLNHAC_00280	rrl	23S ribosomal RNA
contig_1	rRNA	226059	226174	+	GLNHAC_00281	rrf	5S ribosomal RNA
contig_1	tRNA	226219	226293	+	GLNHAC_00282	trnN	tRNA-Asn(gtt)
contig_1	tRNA	226296	226368	+	GLNHAC_00283	trnT	tRNA-Thr(ggt)
contig_1	cds	226475	227659	-	GLNHAC_00284		Site-specific recombinase, phage integrase family protein
contig_1	cds	227646	228179	-	GLNHAC_00285	irrE	IrrE N-terminal-like domain-containing protein
contig_1	cds	228266	228931	-	GLNHAC_00286		DUF4352 domain-containing protein
contig_1	cds	229177	229506	-	GLNHAC_00287		helix-turn-helix transcriptional regulator
contig_1	cds	229667	229900	+	GLNHAC_00288		Helix-turn-helix, putative
contig_1	cds	229913	230104	+	GLNHAC_00289		helix-turn-helix domain-containing protein
contig_1	cds	230169	230702	+	GLNHAC_00290		Bacteriophage-related protein
contig_1	cds	230692	230952	+	GLNHAC_00291		Prophage MuSo1, F protein, putative
contig_1	cds	230953	231117	+	GLNHAC_00292		hypothetical protein
contig_1	cds	231537	231722	+	GLNHAC_00293		hypothetical protein
contig_1	cds	231719	233692	+	GLNHAC_00294		Nuclease SbcCD subunit C
contig_1	cds	233735	234493	+	GLNHAC_00295	bet	phage recombination protein Bet
contig_1	cds	234490	235191	+	GLNHAC_00296		Metallo-beta-lactamase domain-containing protein
contig_1	cds	235188	235367	+	GLNHAC_00297		hypothetical protein
contig_1	cds	235390	236145	+	GLNHAC_00298		Replication protein
contig_1	cds	236154	236444	+	GLNHAC_00299		Replicative helicase inhibitor G39P N-terminal domain-contai
contig_1	cds	236419	237720	+	GLNHAC_00300	dnaB	replicative DNA helicase
contig_1	cds	237739	237933	+	GLNHAC_00301	fbpA	Fur-regulated basic protein FbpA
contig_1	cds	237933	238028	+	GLNHAC_00302		hypothetical protein
contig_1	cds	238034	238489	+	GLNHAC_00303	rusA	Endodeoxyribonuclease RusA
contig_1	cds	238697	238876	+	GLNHAC_00304		hypothetical protein
contig_1	cds	238946	239149	+	GLNHAC_00305		Phage protein
contig_1	cds	239191	239478	+	GLNHAC_00306		VWA domain-containing protein
contig_1	cds	239475	239696	+	GLNHAC_00307		hypothetical protein
contig_1	cds	239773	240228	+	GLNHAC_00308		hypothetical protein
contig_1	cds	240301	240687	+	GLNHAC_00309	arpU	Phage transcriptional regulator, ArpU family subfamily
contig_1	cds	240927	241973	+	GLNHAC_00310		Oxidoreductase, FAD/FMN-binding protein
contig_1	cds	242051	242584	-	GLNHAC_00311		Glyoxalase family protein
contig_1	cds	242616	243011	-	GLNHAC_00312	doxX	DoxX
contig_1	cds	243325	243738	+	GLNHAC_00313	marR	MarR family winged helix-turn-helix transcriptional regulator
contig_1	cds	244346	244513	+	GLNHAC_00314		Phage protein
contig_1	cds	244608	244952	+	GLNHAC_00315		Structural protein
contig_1	cds	245020	245478	+	GLNHAC_00316		SMODS and SLOG-associating 2TM effector domain-contain
contig_1	cds	245534	246055	+	GLNHAC_00317		DNA methylase
contig_1	cds	246066	247085	+	GLNHAC_00318		DNA (cytosine-5-)-methyltransferase
contig_1	cds	247136	247903	+	GLNHAC_00319		DUF4145 domain-containing protein
contig_1	cds	248058	248306	+	GLNHAC_00320	copG	CopG family transcriptional regulator
contig_1	cds	248354	249232	+	GLNHAC_00321	terS	phage terminase small subunit
contig_1	cds	249229	250554	+	GLNHAC_00322		PBSX family phage terminase large subunit
contig_1	cds	250547	252079	+	GLNHAC_00323		phage portal protein
contig_1	cds	252076	252993	+	GLNHAC_00324		Phage Mu protein F like protein
contig_1	cds	253037	253681	+	GLNHAC_00325		DUF4145 domain-containing protein
contig_1	cds	253710	254891	+	GLNHAC_00326	xkdF	Phage-like element PBSX protein XkdF domain-containing pr
contig_1	cds	254923	255858	+	GLNHAC_00327		Phage major capsid protein
contig_1	cds	255869	256186	+	GLNHAC_00328	yqbF	Uncharacterized protein YqbF
contig_1	cds	256192	256584	+	GLNHAC_00329	yqbG	Protein YqbG
contig_1	cds	256581	256943	+	GLNHAC_00330		DUF3599 domain-containing protein
contig_1	cds	256940	257443	+	GLNHAC_00331	yqbI	Uncharacterized protein YqbI
contig_1	cds	257456	257893	+	GLNHAC_00332		Phage portal protein
contig_1	cds	257890	258081	+	GLNHAC_00333		Phage protein
contig_1	cds	258082	259482	+	GLNHAC_00334	yqbK	Uncharacterized protein YqbK
contig_1	cds	259484	259927	+	GLNHAC_00335	xkdM	Phage-like element PBSX protein XkdM
contig_1	ncRNA	260106	260381	-	GLNHAC_00336	bsrG	BsrG
contig_1	cds	260550	260999	+	GLNHAC_00337	yqbN	Uncharacterized protein YqbN
contig_1	cds	261023	261178	+	GLNHAC_00338	xkdN	XkdN
contig_1	cds	261181	266523	+	GLNHAC_00339		TMP repeat family
contig_1	cds	266516	267175	+	GLNHAC_00340	yqbP	Uncharacterized protein YqbP
contig_1	cds	267188	268168	+	GLNHAC_00341	yqbQ	Uncharacterized protein YqbQ
contig_1	cds	268165	268431	+	GLNHAC_00342		DUF2577 domain-containing protein
contig_1	cds	268444	268869	+	GLNHAC_00343	yqbS	Uncharacterized protein YqbS
contig_1	cds	268862	269908	+	GLNHAC_00344	yqbT	Uncharacterized protein YqbT
contig_1	cds	269892	270470	+	GLNHAC_00345	yqcA	Uncharacterized protein YqcA
contig_1	cds	270467	270739	+	GLNHAC_00346	yqcB	Uncharacterized protein YqcB
contig_1	cds	270743	272497	+	GLNHAC_00347		Terminase
contig_1	cds	272509	272838	+	GLNHAC_00348	yorD	Bacteriophage SP-beta YorD domain-containing protein
contig_1	cds	272835	272999	+	GLNHAC_00349	yqcE	XkdX family protein
contig_1	cds	273091	273984	+	GLNHAC_00350		Phage portal protein
contig_1	cds	274030	274452	+	GLNHAC_00351		Holin family
contig_1	cds	274497	275471	+	GLNHAC_00352		N-acetylmuramoyl-L-alanine amidase
contig_1	cds	275510	275794	-	GLNHAC_00353		Group-specific protein
contig_1	cds	275800	277512	-	GLNHAC_00354	ywqJ	YwqJ
contig_1	cds	277722	278855	+	GLNHAC_00355		Response regulator aspartate phosphatase B
contig_1	cds	278845	279003	+	GLNHAC_00356		hypothetical protein
contig_1	cds	279035	279208	-	GLNHAC_00357		YolD-like family protein
contig_1	cds	279430	280485	-	GLNHAC_00358		Abortive infection protein
contig_1	tRNA	281292	281366	+	GLNHAC_00359	trnG	tRNA-Gly(gcc)
contig_1	tRNA	281399	281475	+	GLNHAC_00360	trnR	tRNA-Arg(acg)
contig_1	tRNA	281502	281578	+	GLNHAC_00361	trnP	tRNA-Pro(tgg)
contig_1	tRNA	281586	281661	+	GLNHAC_00362	trnA	tRNA-Ala(tgc)
contig_1	rRNA	281835	283384	+	GLNHAC_00363	rrs	16S ribosomal RNA
contig_1	rRNA	283554	286480	+	GLNHAC_00364	rrl	23S ribosomal RNA
contig_1	rRNA	286596	286711	+	GLNHAC_00365	rrf	5S ribosomal RNA
contig_1	cds	286850	287971	-	GLNHAC_00366		Transporter, major facilitator family
contig_1	cds	288243	289511	+	GLNHAC_00367		4-aminobutyrate aminotransferase
contig_1	cds	289508	290458	+	GLNHAC_00368		Inorganic polyphosphate kinase
contig_1	cds	290433	291479	+	GLNHAC_00369		Oxidoreductase, zinc-binding protein
contig_1	cds	291718	293154	+	GLNHAC_00370	ybaR	Putative sulfate transporter YbaR
contig_1	cds	293259	294218	+	GLNHAC_00371	ybaS	Uncharacterized protein YbaS
contig_1	cds	294234	294980	-	GLNHAC_00372	ybbA	YbbA
contig_1	cds	294977	295570	-	GLNHAC_00373	feuC	iron ABC transporter permease FeuC
contig_1	cds	295552	295812	-	GLNHAC_00374		hypothetical protein
contig_1	cds	295979	296629	-	GLNHAC_00375		iron ABC transporter permease
contig_1	cds	296626	296982	-	GLNHAC_00376		iron chelate uptake ABC transporter family permease subunit
contig_1	cds	297001	297954	-	GLNHAC_00377	feuA	iron ABC transporter substrate-binding protein FeuA
contig_1	cds	298045	299637	-	GLNHAC_00378		ABC transporter substrate-binding protein
contig_1	cds	299827	301071	-	GLNHAC_00379	namZ	Peptidoglycan beta-N-acetylmuramidase NamZ
contig_1	cds	301085	303013	-	GLNHAC_00380		beta-N-acetylhexosaminidase
contig_1	cds	303041	303274	-	GLNHAC_00381		hypothetical protein
contig_1	cds	303383	303817	-	GLNHAC_00382		Beta-lactamase-related domain-containing protein
contig_1	cds	303775	304365	-	GLNHAC_00383		hypothetical protein
contig_1	cds	304421	305209	-	GLNHAC_00384		PTS system, N-acetylmuramic acid-specific IIB component
contig_1	cds	305206	305787	-	GLNHAC_00385	ybbF	(pseudo) YbbF
contig_1	cds	305813	306664	-	GLNHAC_00386	ybbH	Uncharacterized HTH-type transcriptional regulator YbbH
contig_1	cds	306678	307595	-	GLNHAC_00387	murQ	N-acetylmuramic acid 6-phosphate etherase
contig_1	cds	307705	308079	-	GLNHAC_00388		N-acetyltransferase domain-containing protein
contig_1	cds	308073	308186	-	GLNHAC_00389		hypothetical protein
contig_1	cds	308200	308655	-	GLNHAC_00390	ybbK	Uncharacterized protein YbbK
contig_1	tRNA	308836	308910	+	GLNHAC_00391	trnE	tRNA-Glu(ttc)
contig_1	tRNA	308914	308989	+	GLNHAC_00392	trnV	tRNA-Val(tac)
contig_1	tRNA	308994	309066	+	GLNHAC_00393	trnT	tRNA-Thr(tgt)
contig_1	tRNA	309087	309171	+	GLNHAC_00394	trnY	tRNA-Tyr(gta)
contig_1	tRNA	309176	309247	+	GLNHAC_00395	trnQ	tRNA-Gln(ttg)
contig_1	cds	309476	309787	+	GLNHAC_00396		RNA polymerase sigma factor
contig_1	cds	309790	310038	+	GLNHAC_00397		sigma-70 family RNA polymerase sigma factor
contig_1	cds	310052	310678	+	GLNHAC_00398	rsiW	anti-sigma-W factor RsiW
contig_1	cds	310846	311667	+	GLNHAC_00399	cdaA	diadenylate cyclase CdaA
contig_1	cds	311660	313114	+	GLNHAC_00400	cdaR	CdaA regulatory protein CdaR
contig_1	cds	313133	314479	+	GLNHAC_00401	glmM	phosphoglucosamine mutase
contig_1	cds	314525	314791	+	GLNHAC_00402		hypothetical protein
contig_1	ncRNA-region	314652	314822	+			glmS glucosamine-6-phosphate activated ribozyme aptamer

contig_1	cds	314911	316713	+	GLNHAC_00403	glmS	glutamine--fructose-6-phosphate transaminase (isomerizing)
contig_1	cds	317267	317425	+	GLNHAC_00404		hypothetical protein
contig_1	cds	317476	317667	+	GLNHAC_00405		hypothetical protein
contig_1	cds	318195	318401	+	GLNHAC_00406		hypothetical protein
contig_1	cds	318691	318792	+	GLNHAC_00407		hypothetical protein
contig_1	cds	320632	322149	+	GLNHAC_00408		NADH dehydrogenase subunit 5
contig_1	cds	322164	324788	+	GLNHAC_00409	dabA	putative inorganic carbon transporter subunit DabA
contig_1	cds	324861	325379	+	GLNHAC_00410		Carbonic anhydrase
contig_1	cds	325450	325740	+	GLNHAC_00411		DUF6407 domain-containing protein
contig_1	cds	325796	326170	+	GLNHAC_00412	ybcI	Uncharacterized protein YbcI
contig_1	cds	326741	327043	+	GLNHAC_00413	ybzH	Uncharacterized HTH-type transcriptional regulator YbzH
contig_1	cds	327169	328341	+	GLNHAC_00414	ybcL	Uncharacterized MFS-type transporter YbcL
contig_1	cds	328972	329487	+	GLNHAC_00415	skfG	sporulation killing factor biosynthesis/export protein SkfG
contig_1	cds	329698	330705	-	GLNHAC_00416		Beta-lactamase
contig_1	cds	330711	331541	-	GLNHAC_00417		Transcriptional regulator, putative
contig_1	cds	331677	332654	+	GLNHAC_00418	ybdG	Uncharacterized protein YbdG
contig_1	cds	332656	333327	+	GLNHAC_00419	ybdJ	two-component system response regulator YbdJ
contig_1	cds	333324	334310	+	GLNHAC_00420	ybdK	Sensor histidine kinase YbdK
contig_1	cds	334369	334623	+	GLNHAC_00421	ybzI	Uncharacterized protein YbzI
contig_1	cds	334616	335386	-	GLNHAC_00422		non-specific serine/threonine protein kinase
contig_1	cds	335495	336352	-	GLNHAC_00423	ybdN	Uncharacterized protein YbdN
contig_1	cds	336484	337668	+	GLNHAC_00424	ybdO	Uncharacterized protein YbdO
contig_1	cds	337992	339380	+	GLNHAC_00425	ybxG	putative threonine/serine transporter YbxG
contig_1	cds	339484	339741	+	GLNHAC_00426	csgA	sigma-G-dependent sporulation-specific acid-soluble spore p
contig_1	cds	339756	339947	+	GLNHAC_00427	ybxH	YbxH protein
contig_1	cds	339971	340414	-	GLNHAC_00428		Beta-lactamase class D
contig_1	cds	340423	340773	-	GLNHAC_00429	blaOXA	(pseudo) class D beta-lactamase
contig_1	cds	340945	342198	+	GLNHAC_00430		Cytochrome P450
contig_1	cds	342238	342498	-	GLNHAC_00431	ybyB	Uncharacterized protein YbyB
contig_1	cds	342868	344499	+	GLNHAC_00432	phoU	PhoU domain-containing protein
contig_1	cds	344525	345415	-	GLNHAC_00433	glpQ	Glycerophosphodiester phosphodiesterase
contig_1	cds	345514	346848	-	GLNHAC_00434	glpT	glycerol-3-phosphate transporter
contig_1	cds	347142	347390	+	GLNHAC_00435		DUF2651 domain-containing protein
contig_1	cds	347482	348399	+	GLNHAC_00436	ybfA	Putative HTH-type DNA-binding domain-containing acetyltrar
contig_1	cds	348396	349649	+	GLNHAC_00437	ybfB	Uncharacterized MFS-type transporter YbfB
contig_1	cds	350257	352356	-	GLNHAC_00438		glycoside hydrolase domain-containing protein
contig_1	cds	352429	353349	-	GLNHAC_00439	ybfH	Uncharacterized transporter YbhF
contig_1	cds	353423	353764	-	GLNHAC_00440		(pseudo) Transcriptional regulator, AraC/XylS family
contig_1	cds	353815	354171	-	GLNHAC_00441	araC	AraC family ligand binding domain-containing protein
contig_1	cds	354408	355562	+	GLNHAC_00442	purT	phosphoribosylglycinamide formyltransferase 2
contig_1	cds	355705	356643	+	GLNHAC_00443	mpr	extracellular metalloprotease Mpr
contig_1	cds	356606	357004	+	GLNHAC_00444	ybfJ	Uncharacterized protein YbfJ
contig_1	cds	357176	358120	+	GLNHAC_00445		Carboxylesterase NA
contig_1	cds	358257	358790	+	GLNHAC_00446	pssA	CDP-diacylglycerol--serine O-phosphatidyltransferase
contig_1	cds	358781	359269	+	GLNHAC_00447	ybfM	Uncharacterized membrane protein YbfM
contig_1	cds	359262	360053	+	GLNHAC_00448	psd	phosphatidylserine decarboxylase
contig_1	cds	360109	360387	+	GLNHAC_00449	ybfN	Uncharacterized protein YbfN
contig_1	cds	360492	361832	+	GLNHAC_00450		Hydrolase
contig_1	cds	362083	363051	+	GLNHAC_00451	trhO	rhodanese-related sulfurtransferase
contig_1	cds	363089	364333	-	GLNHAC_00452	gltP	glutamate-aspartate/proton symporter GltP
contig_1	cds	364476	364886	-	GLNHAC_00453		hypothetical protein
contig_1	cds	364828	366372	-	GLNHAC_00454	gamP	PTS system glucosamine-specific EIICBA component
contig_1	cds	366393	367142	-	GLNHAC_00455	nagB	glucosamine-6-phosphate deaminase
contig_1	cds	367360	368067	+	GLNHAC_00456	gamR	transcriptional regulator GamR
contig_1	cds	368101	368376	+	GLNHAC_00457		DUF1648 domain-containing protein
contig_1	cds	368569	369657	+	GLNHAC_00458	ilvE	branched-chain amino acid aminotransferase
contig_1	cds	369694	371106	-	GLNHAC_00459	mmuP	S-methylmethionine permease
contig_1	cds	371227	372174	-	GLNHAC_00460	mmuM	homocysteine S-methyltransferase
contig_1	cds	372302	373738	-	GLNHAC_00461	glnT	putative sodium/glutamine symporter GlnT
contig_1	cds	373760	374743	-	GLNHAC_00462	glsA1	glutaminase
contig_1	cds	374987	376276	+	GLNHAC_00463	glnK	two-component system sensor histidine kinase GlnK
contig_1	cds	376409	377098	+	GLNHAC_00464		Stage 0 sporulation A-like protein
contig_1	cds	377460	378386	+	GLNHAC_00465	kdgD	5-dehydro-4-deoxyglucarate dehydratase
contig_1	cds	378386	379885	+	GLNHAC_00466	gucD	alpha-ketoglutaric semialdehyde dehydrogenase GucD
contig_1	cds	379970	381337	+	GLNHAC_00467	gudP	glucarate transporter GudP
contig_1	cds	381374	382741	+	GLNHAC_00468	gudD	glucarate dehydratase
contig_1	cds	382811	383512	+	GLNHAC_00469	ycbG	Uncharacterized HTH-type transcriptional regulator YcbG
contig_1	cds	383604	385136	+	GLNHAC_00470	garD	galactarate dehydratase
contig_1	cds	385429	386349	+	GLNHAC_00471	mphK	macrolide 2'-phosphotransferase MphK
contig_1	ncRNA-region	386406	386653	+			T-box leader
contig_1	cds	386749	386910	+	GLNHAC_00472		Conserved domain protein
contig_1	cds	386931	387869	+	GLNHAC_00473	ycbK	YcbK
contig_1	cds	387954	388646	+	GLNHAC_00474	ycbL	two-component system response regulator YcbL
contig_1	cds	388648	389583	+	GLNHAC_00475		histidine kinase
contig_1	cds	389678	390601	+	GLNHAC_00476	ycbN	Uncharacterized ABC transporter ATP-binding protein YcbN
contig_1	cds	390620	391306	+	GLNHAC_00477	ycbO	Uncharacterized protein YcbO
contig_1	cds	391361	391747	-	GLNHAC_00478	ycbP	Uncharacterized protein YcbP
contig_1	cds	391860	393095	+	GLNHAC_00479		Proteinase inhibitor I4, serpin
contig_1	cds	393179	393607	+	GLNHAC_00480	cwlJ	cell wall hydrolase CwlJ
contig_1	cds	393715	394446	+	GLNHAC_00481	ycbR	Uncharacterized protein YcbR
contig_1	cds	394721	396472	+	GLNHAC_00482	phoD	alkaline phosphatase PhoD
contig_1	cds	396486	396698	+	GLNHAC_00483	tatAD	sec-independent protein translocase protein TatAD
contig_1	cds	396758	397483	+	GLNHAC_00484	tatC	twin-arginine translocase subunit TatC
contig_1	cds	397480	398127	-	GLNHAC_00485	pcp	pyroglutamyl-peptidase I
contig_1	cds	398207	399319	+	GLNHAC_00486	ycbU	YcbU
contig_1	cds	399362	400795	-	GLNHAC_00487	lmr(B)	lincomycin efflux MFS transporter Lmr(B)
contig_1	cds	400839	401405	-	GLNHAC_00488	lmrA	transcriptional regulator LmrA
contig_1	cds	401618	402754	+	GLNHAC_00489	ansZ	L-asparaginase 2
contig_1	cds	402926	403564	+	GLNHAC_00490		Lipase
contig_1	cds	403602	403988	-	GLNHAC_00491	yczC	Uncharacterized protein YczC
contig_1	cds	404220	405296	+	GLNHAC_00492	yccF	Uncharacterized protein YccF
contig_1	cds	405336	406292	-	GLNHAC_00493		Sensor-like histidine kinase
contig_1	cds	406305	407006	-	GLNHAC_00494	natR	two-component system response regulator NatR
contig_1	cds	407150	407890	+	GLNHAC_00495	natA	sodium ABC transporter ATP-binding protein NatA
contig_1	cds	407891	409051	+	GLNHAC_00496	natB	sodium ABC transporter permease NatB
contig_1	cds	409187	410119	+	GLNHAC_00497	yccK	Uncharacterized oxidoreductase YccK
contig_1	cds	410158	411222	-	GLNHAC_00498	ycdA	Uncharacterized lipoprotein YcdA
contig_1	cds	411550	412965	+	GLNHAC_00499		Putative hydrolase
contig_1	cds	413151	414512	+	GLNHAC_00500		YcdB/YcdC repeated domain-containing protein
contig_1	cds	414519	415022	-	GLNHAC_00501	cwlK	peptidoglycan L-alanyl-D-glutamate endopeptidase CwlK
contig_1	cds	415143	416264	+	GLNHAC_00502	rapJ	response regulator aspartate phosphatase RapJ
contig_1	cds	416374	417150	+	GLNHAC_00503	ycdF	Glucose 1-dehydrogenase 2
contig_1	cds	417175	418866	+	GLNHAC_00504	ycdG	putative oligo-1,6-glucosidase 2
contig_1	cds	419048	420025	+	GLNHAC_00505	znuA	High-affinity zinc uptake system protein ZnuA
contig_1	cds	420080	420775	+	GLNHAC_00506	znuC	zinc ABC transporter ATP-binding protein ZnuC
contig_1	cds	420733	421575	+	GLNHAC_00507	znuB	zinc ABC transporter permease ZnuB
contig_1	cds	421612	422607	-	GLNHAC_00508	yceB	Uncharacterized protein YceB
contig_1	cds	422891	423490	+	GLNHAC_00509	yceC	Stress response protein SCP2
contig_1	cds	423512	424093	+	GLNHAC_00510	yceD	General stress protein 16U
contig_1	cds	424128	424706	+	GLNHAC_00511	yceE	Uncharacterized protein YceE
contig_1	cds	424758	425531	+	GLNHAC_00512	yceF	Uncharacterized membrane protein YceF
contig_1	cds	425615	425962	+	GLNHAC_00513		hypothetical protein
contig_1	cds	425985	426764	+	GLNHAC_00514	yceG	(pseudo) Uncharacterized protein YceG
contig_1	cds	426764	427225	+	GLNHAC_00515		Putative component of 'biosynthetic module' domain-contain
contig_1	cds	427241	428143	+	GLNHAC_00516	yceH	Uncharacterized protein YceH
contig_1	cds	428115	428330	+	GLNHAC_00517		hypothetical protein
contig_1	cds	428442	429644	+	GLNHAC_00518	niaP	niacin permease NiaP
contig_1	cds	429920	430207	-	GLNHAC_00519	yceK	Uncharacterized HTH-type transcriptional regulator YceK
contig_1	cds	430512	431120	+	GLNHAC_00520		Betaine/proline/choline family ABC transporter ATP-binding p
contig_1	cds	431132	431767	+	GLNHAC_00521		CBS domain-containing protein
contig_1	cds	431769	432617	+	GLNHAC_00522	opuAB	glycine/proline betaine ABC transporter permease subunit Op
contig_1	cds	432617	433498	+	GLNHAC_00523	opuAC	glycine/proline betaine ABC transporter substrate-binding pro
contig_1	cds	433536	434687	-	GLNHAC_00524	amhX	Amidohydrolase AmhX
contig_1	cds	434836	436269	+	GLNHAC_00525	ycgA	Uncharacterized protein YcgA
contig_1	cds	436385	436873	+	GLNHAC_00526	ycgB	Uncharacterized protein YcgB
contig_1	cds	437124	439103	+	GLNHAC_00527		Alpha-amylase
contig_1	cds	439280	440242	+	GLNHAC_00528	ldh	L-lactate dehydrogenase
contig_1	cds	440274	441899	+	GLNHAC_00529	lctP	L-lactate permease
contig_1	cds	441943	443481	-	GLNHAC_00530	bmr3	multidrug efflux MFS transporter Bmr3
contig_1	cds	443594	444058	+	GLNHAC_00531	ycgE	Uncharacterized HTH-type transcriptional regulator YcgE
contig_1	cds	444133	444762	+	GLNHAC_00532	ycgF	Putative amino acid efflux protein YcgF
contig_1	cds	444832	445593	+	GLNHAC_00533	ycgG	Uncharacterized protein YcgG
contig_1	cds	445595	446941	-	GLNHAC_00534	ycgH	Uncharacterized transporter YcgH
contig_1	cds	447065	447661	+	GLNHAC_00535	ycgI	Uncharacterized protein YcgI
contig_1	cds	447794	448612	+	GLNHAC_00536	nadE	ammonia-dependent NAD(+) synthetase
contig_1	ncRNA	448705	448771	+	GLNHAC_00537	Bacillaceae-1	Bacillaceae-1 RNA
contig_1	cds	448886	449485	-	GLNHAC_00538	tmrB	tunicamycin resistance ATP-binding TmrB
contig_1	cds	449786	450346	+	GLNHAC_00539		Shikimate kinase
contig_1	cds	450373	451134	-	GLNHAC_00540		Methyltransferase type 11 domain-containing protein

contig_1	cds	451252	452226	+	GLNHAC_00541	ycgK	Uncharacterized HTH-type transcriptional regulator YcgK
contig_1	cds	452297	453253	+	GLNHAC_00542	cah	cephalosporin C deacetylase
contig_1	cds	453337	454119	+	GLNHAC_00543	ycgL	Uncharacterized protein YcgL
contig_1	cds	454307	454831	+	GLNHAC_00544	putB	(pseudo) Proline dehydrogenase 2
contig_1	cds	454819	455217	+	GLNHAC_00545		proline dehydrogenase family protein
contig_1	cds	455234	455890	+	GLNHAC_00546		hypothetical protein
contig_1	cds	455910	456782	+	GLNHAC_00547		aldehyde dehydrogenase family protein
contig_1	cds	456898	458328	+	GLNHAC_00548	putP	sodium/proline symporter PutP
contig_1	cds	458481	459716	+	GLNHAC_00549	putR	proline utilization transcriptional regulator PutR
contig_1	cds	459753	460610	-	GLNHAC_00550	ycgQ	TIGR03943 family putative permease subunit
contig_1	cds	460615	461517	-	GLNHAC_00551	ycgR	UPF0718 protein YcgR
contig_1	cds	461599	462453	-	GLNHAC_00552		Aromatic hydrocarbon catabolism protein
contig_1	cds	462616	463626	+	GLNHAC_00553	ycgT	ferredoxin--NADP(+) reductase
contig_1	cds	463654	464907	-	GLNHAC_00554		uroporphyrinogen-III C-methyltransferase
contig_1	cds	465252	465482	-	GLNHAC_00555		hypothetical protein
contig_1	cds	465514	467931	-	GLNHAC_00556	nasD	NADPH-nitrite reductase
contig_1	cds	468052	469863	-	GLNHAC_00557	nasC	assimilatory nitrate reductase catalytic subunit NasC
contig_1	cds	469887	470183	-	GLNHAC_00558		(pseudo) molybdopterin-dependent oxidoreductase
contig_1	cds	470190	472505	-	GLNHAC_00559		Nitrite reductase [NAD(P)H], large subunit
contig_1	cds	472671	473876	+	GLNHAC_00560	nasA	Nitrate transporter
contig_1	cds	473992	474906	+	GLNHAC_00561	folE2	GTP cyclohydrolase FolE2
contig_1	cds	474903	475028	+	GLNHAC_00562		hypothetical protein
contig_1	cds	475124	475495	+	GLNHAC_00563		L,D-TPase catalytic domain-containing protein
contig_1	cds	475578	476750	+	GLNHAC_00564		Flavoprotein
contig_1	cds	476788	477981	+	GLNHAC_00565	zinU	zinc metallochaperone ZinU
contig_1	cds	478028	478708	-	GLNHAC_00566	yckA	putative amino-acid ABC transporter permease protein YckA
contig_1	cds	478718	479581	-	GLNHAC_00567	yckB	putative ABC transporter extracellular-binding protein YckB
contig_1	cds	479749	479862	+	GLNHAC_00568		hypothetical protein
contig_1	cds	479995	480450	+	GLNHAC_00569	yckC	Uncharacterized protein YckC
contig_1	cds	480533	480865	+	GLNHAC_00570	yckD	Uncharacterized protein YckD
contig_1	cds	481000	481422	+	GLNHAC_00571	sarZ	HTH-type transcriptional regulator SarZ
contig_1	cds	481410	481751	+	GLNHAC_00572		DUF3147 domain-containing protein
contig_1	cds	481771	482499	+	GLNHAC_00573		ABC transporter domain-containing protein
contig_1	cds	482572	484005	+	GLNHAC_00574	bglC	6-phospho-beta-glucosidase
contig_1	cds	484041	484439	-	GLNHAC_00575	nin	DNA-entry nuclease inhibitor
contig_1	cds	484462	484899	-	GLNHAC_00576	nucA	DNA-entry nuclease
contig_1	cds	485085	486809	-	GLNHAC_00577	tlpC	Methyl-accepting chemotaxis protein TlpC
contig_1	cds	486921	487478	-	GLNHAC_00578	hxlB	3-hexulose-6-phosphate isomerase
contig_1	cds	487483	488115	-	GLNHAC_00579	hxlA	3-hexulose-6-phosphate synthase
contig_1	cds	488347	488709	+	GLNHAC_00580	hxlR	transcriptional activator HxlR
contig_1	cds	489280	496473	+	GLNHAC_00581	dltA	D-alanine--poly(phosphoribitol) ligase subunit DltA
contig_1	cds	496470	500042	+	GLNHAC_00582		Amino acid adenylation domain-containing protein
contig_1	cds	500055	510806	+	GLNHAC_00583	dltA	surfactin non-ribosomal peptide synthetase SrfAB
contig_1	cds	510842	513295	+	GLNHAC_00584		surfactin non-ribosomal peptide synthetase SrfAC
contig_1	cds	513271	514668	+	GLNHAC_00585		hypothetical protein
contig_1	cds	514696	515424	+	GLNHAC_00586	srfAD	surfactin biosynthesis thioesterase SrfAD
contig_1	cds	515525	516751	+	GLNHAC_00587	ycxA	Uncharacterized MFS-type transporter YcxA
contig_1	cds	516766	517323	-	GLNHAC_00588		YcxB-like C-terminal domain-containing protein
contig_1	cds	517377	518315	-	GLNHAC_00589	ycxC	Uncharacterized transporter YcxC
contig_1	cds	518439	519482	+	GLNHAC_00590	ycxD	Uncharacterized HTH-type transcriptional regulator YcxD
contig_1	cds	519477	519821	-	GLNHAC_00591	sfp	(pseudo) 4'-phosphopantetheinyl transferase Sfp
contig_1	cds	519818	520150	-	GLNHAC_00592		hypothetical protein
contig_1	cds	520255	520902	-	GLNHAC_00593	yczE	Uncharacterized membrane protein YczE
contig_1	cds	521024	521146	-	GLNHAC_00594		hypothetical protein
contig_1	cds	521146	521865	-	GLNHAC_00595		tetratricopeptide repeat protein
contig_1	cds	522148	522891	-	GLNHAC_00596	tcyC	cystine ABC transporter ATP-binding protein TcyC
contig_1	cds	522905	523609	-	GLNHAC_00597	tcyB	cystine ABC transporter permease TcyB
contig_1	cds	523596	524402	-	GLNHAC_00598	tcyA	cystine ABC transporter substrate-binding lipoprotein TcyA
contig_1	cds	524518	525390	-	GLNHAC_00599	bsdA	transcriptional regulator BsdA
contig_1	cds	525480	526094	+	GLNHAC_00600	bsdB	non-oxidative hydroxyarylic acid decarboxylases subunit B
contig_1	cds	526097	527515	+	GLNHAC_00601	bsdC	non-oxidative hydroxyarylic acid decarboxylases subunit C
contig_1	cds	527532	527759	+	GLNHAC_00602	bsdD	phenolic acid decarboxylase subunit BsdD
contig_1	cds	527756	528220	+	GLNHAC_00603		Pyridoxamine 5'-phosphate oxidase N-terminal domain-conta
contig_1	cds	528288	529133	+	GLNHAC_00604		Alpha/beta hydrolase
contig_1	cds	529173	530651	-	GLNHAC_00605	yclF	Uncharacterized transporter YclF
contig_1	cds	530766	531692	+	GLNHAC_00606		hypothetical protein
contig_1	cds	531844	532680	+	GLNHAC_00607	yclG	(pseudo) Uncharacterized protein YclG
contig_1	cds	532588	532917	-	GLNHAC_00608	yczF	YczF
contig_1	cds	533036	534631	+	GLNHAC_00609	gerKA	Spore germination protein KA
contig_1	cds	534659	535216	+	GLNHAC_00610		Ger(X)C family germination protein
contig_1	cds	535186	535374	+	GLNHAC_00611		hypothetical protein
contig_1	cds	535419	535760	+	GLNHAC_00612		hypothetical protein
contig_1	cds	535757	535879	+	GLNHAC_00613		hypothetical protein
contig_1	cds	535904	537028	+	GLNHAC_00614	gerKB	Spore germination protein KB
contig_1	cds	537131	537811	-	GLNHAC_00615	yclH	Uncharacterized ABC transporter ATP-binding protein YclH
contig_1	cds	537827	538849	-	GLNHAC_00616		ABC transporter permease
contig_1	cds	538824	539282	-	GLNHAC_00617		hypothetical protein
contig_1	cds	539381	540178	+	GLNHAC_00618	yclJ	Uncharacterized transcriptional regulatory protein YclJ
contig_1	cds	540168	541586	+	GLNHAC_00619	yclK	two-component system sensor histidine kinase YclK
contig_1	cds	541748	542896	+	GLNHAC_00620	rapC	Regulatory protein RapC
contig_1	cds	542880	543002	+	GLNHAC_00621	phrC	phosphatase RapC inhibitor PhrC
contig_1	cds	543101	543208	-	GLNHAC_00622	yjcZ	YjcZ family sporulation protein
contig_1	cds	543361	544725	-	GLNHAC_00623	yclM	aspartate kinase
contig_1	cds	545111	546061	+	GLNHAC_00624	yclN	petrobactin ABC transporter permease YclN
contig_1	cds	546054	547001	+	GLNHAC_00625	yclO	petrobactin ABC transporter permease YclO
contig_1	cds	546995	547657	+	GLNHAC_00626	yclP	Petrobactin import ATP-binding protein YclP
contig_1	cds	547776	548732	+	GLNHAC_00627	yclQ	petrobactin ABC transporter substrate-binding protein YclQ
contig_1	cds	548776	548943	-	GLNHAC_00628		(pseudo) Major facilitator superfamily (MFS) profile domain-cc
contig_1	cds	548940	549659	-	GLNHAC_00629		MFS transporter
contig_1	cds	549656	550192	-	GLNHAC_00630		Major facilitator superfamily (MFS) profile domain-containing p
contig_1	cds	550212	551090	-	GLNHAC_00631	ycnC	Uncharacterized HTH-type transcriptional regulator YcnC
contig_1	cds	551253	552002	-	GLNHAC_00632	nfsA	oxygen-insensitive NADPH nitroreductase
contig_1	cds	552019	552306	-	GLNHAC_00633	ycnE	Putative monooxygenase YcnE
contig_1	cds	552447	552761	+	GLNHAC_00634	yczG	Uncharacterized HTH-type transcriptional regulator YczG
contig_1	cds	552763	554202	-	GLNHAC_00635	gabR	HTH-type transcriptional regulatory protein GabR
contig_1	cds	554308	555618	+	GLNHAC_00636	gabT	4-aminobutyrate--2-oxoglutarate transaminase
contig_1	cds	555687	556031	+	GLNHAC_00637		hypothetical protein
contig_1	cds	555991	557076	+	GLNHAC_00638		succinate-semialdehyde dehydrogenase
contig_1	cds	557201	558064	+	GLNHAC_00639	glcU	glucose uptake protein GlcU
contig_1	cds	558083	558868	+	GLNHAC_00640	gdh	glucose 1-dehydrogenase
contig_1	cds	558912	559526	-	GLNHAC_00641	ycnI	Uncharacterized protein YcnI
contig_1	cds	559540	561165	-	GLNHAC_00642	ycnJ	Copper transport protein YcnJ
contig_1	cds	561200	561772	-	GLNHAC_00643	ycnK	copper uptake transcriptional regulator YcnK
contig_1	cds	561938	562291	+	GLNHAC_00644	ycnL	Uncharacterized protein YcnL
contig_1	cds	562463	563896	+	GLNHAC_00645		PTS system mannitol-specific EIICB component
contig_1	cds	563921	564352	+	GLNHAC_00646	mtlF	Mannitol-specific phosphotransferase enzyme IIA component
contig_1	cds	564354	565475	+	GLNHAC_00647	mtlD	mannitol-1-phosphate 5-dehydrogenase
contig_1	cds	565572	566630	+	GLNHAC_00648	ycsA	tartrate dehydrogenase
contig_1	cds	566821	567225	-	GLNHAC_00649		(pseudo) Dienelactone hydrolase family
contig_1	cds	567310	567702	+	GLNHAC_00650	fabZ	3-hydroxyacyl-ACP dehydratase FabZ family protein
contig_1	cds	567737	567901	-	GLNHAC_00651	yczO	Uncharacterized protein YczO
contig_1	cds	568034	568489	+	GLNHAC_00652	ycsE	(pseudo) phosphoglycolate phosphatase
contig_1	cds	568474	568782	+	GLNHAC_00653	ycsE	(pseudo) phosphoglycolate phosphatase
contig_1	cds	568988	569761	+	GLNHAC_00654	pxpA	5-oxoprolinase subunit PpxA
contig_1	cds	569776	570990	+	GLNHAC_00655	ycsG	Uncharacterized membrane protein YcsG
contig_1	cds	570996	571787	+	GLNHAC_00656	ycsI	putative hydro-lyase
contig_1	cds	571833	572555	+	GLNHAC_00657	pxpB	5-oxoprolinase subunit B
contig_1	cds	572558	573565	+	GLNHAC_00658	pxpC	5-oxoprolinase subunit C
contig_1	cds	573581	574333	+	GLNHAC_00659	kipR	HTH-type transcriptional regulator KipR
contig_1	cds	574491	575036	+	GLNHAC_00660	lipC	Spore germination lipase LipC
contig_1	cds	575210	575455	+	GLNHAC_00661	yczI	Uncharacterized protein YczI
contig_1	cds	575461	575748	-	GLNHAC_00662		Antibiotic biosynthesis monooxygenase domain protein
contig_1	cds	575900	577906	+	GLNHAC_00663	pbpC	penicillin-binding protein 3
contig_1	cds	578007	578909	+	GLNHAC_00664	ycsN	Uncharacterized oxidoreductase YcsN
contig_1	cds	579099	581189	+	GLNHAC_00665	mtlR	mannitol operon transcriptional activator MtlR
contig_1	cds	581401	582912	+	GLNHAC_00666	ydaB	Putative acyl--CoA ligase YdaB
contig_1	cds	582931	583527	-	GLNHAC_00667		Methyltransferase
contig_1	cds	583678	584538	+	GLNHAC_00668	ydaD	General stress protein 39
contig_1	cds	584554	585057	+	GLNHAC_00669	ydaE	D-lyxose ketol-isomerase
contig_1	cds	585145	585696	+	GLNHAC_00670		Ribosomal-protein-serine acetyltransferase
contig_1	cds	585774	586196	+	GLNHAC_00671		Pyridoxamine 5'-phosphate oxidase family protein
contig_1	ncRNA	586386	586472	-	GLNHAC_00672	bsrC	BsrC
contig_1	cds	586704	587513	+	GLNHAC_00673	amj	lipid II flippase Amj
contig_1	cds	587554	587847	-	GLNHAC_00674	ydzA	Uncharacterized membrane protein YdzA
contig_1	cds	588033	588467	+	GLNHAC_00675	lrpC	transcriptional regulator LrpC
contig_1	cds	588532	590718	+	GLNHAC_00676		DNA topoisomerase III
contig_1	cds	590921	592009	+	GLNHAC_00677	ydaJ	Putative lipoprotein YdaJ
contig_1	cds	591990	592841	+	GLNHAC_00678	ydaK	cyclic-di-GMP receptor EpsK
contig_1	cds	592831	594561	+	GLNHAC_00679	ydaL	Uncharacterized protein YdaL

**contig_1**	cds	594554	595459	+	GLNHAC_00680		Glycosyltransferase 2-like domain-containing protein
**contig_1**	cds	595461	595817	+	GLNHAC_00681		hypothetical protein
**contig_1**	cds	595814	597871	+	GLNHAC_00682	ydaN	Uncharacterized protein YdaN
**contig_1**	ncRNA-region	598069	598212	+			c-di-AMP riboswitch aptamer (ydaO/yuaA leader)
**contig_1**	cds	598406	600193	+	GLNHAC_00683	kimA	potassium transporter KimA
**contig_1**	cds	600286	600756	+	GLNHAC_00684		hypothetical protein
**contig_1**	cds	600803	602527	+	GLNHAC_00685	ydaP	pyruvate oxidase
**contig_1**	cds	602624	602821	+	GLNHAC_00686		hypothetical protein
**contig_1**	cds	602824	603090	-	GLNHAC_00687	ydzK	Uncharacterized membrane protein YdzK
**contig_1**	cds	603193	604470	-	GLNHAC_00688	mntH	Nramp family divalent metal transporter
**contig_1**	cds	604698	604955	-	GLNHAC_00689	ydaS	UPF0410 protein YdaS
**contig_1**	cds	605033	605485	-	GLNHAC_00690		DUF2188 domain-containing protein
**contig_1**	cds	605603	606421	+	GLNHAC_00691	ydbA	Uncharacterized protein YdbA
**contig_1**	cds	606550	606921	+	GLNHAC_00692	gsiB	Glucose starvation-inducible protein GsiB
**contig_1**	cds	607057	607396	+	GLNHAC_00693	ydbB	YdbB
**contig_1**	cds	607789	608610	-	GLNHAC_00694	ydbD	Manganese catalase
**contig_1**	cds	608696	609748	-	GLNHAC_00695	dctB	C4-dicarboxylate TRAP transporter substrate-binding protein
**contig_1**	cds	609818	611425	+	GLNHAC_00696	dctS	two-component system sensor histidine kinase DctS
**contig_1**	cds	611415	612095	+	GLNHAC_00697	dctR	two-component system response regulator DctR
**contig_1**	cds	612216	613481	+	GLNHAC_00698	dctP	C4-dicarboxylate transporter DctP
**contig_1**	cds	613632	614684	+	GLNHAC_00699	ydbI	Putative transport protein YdbI
**contig_1**	ncRNA-region	614733	614862	+			c-di-AMP riboswitch aptamer (ydaO/yuaA leader)
**contig_1**	cds	614936	616174	+	GLNHAC_00700		Proton/sodium-glutamate symport protein
**contig_1**	cds	616266	617192	+	GLNHAC_00701	ydbJ	Uncharacterized ABC transporter ATP-binding protein YdbJ
**contig_1**	cds	617185	617952	+	GLNHAC_00702	ydbK	Uncharacterized membrane protein YdbK
**contig_1**	cds	618047	618382	+	GLNHAC_00703	ydbL	Uncharacterized protein YdbL
**contig_1**	cds	618512	619657	+	GLNHAC_00704	ydbM	Putative acyl-CoA dehydrogenase YdbM
**contig_1**	cds	619682	619826	-	GLNHAC_00705	fbpB	Fur-regulated basic protein FbpB
**contig_1**	cds	619815	619979	-	GLNHAC_00706	fbpA	Fur-regulated basic protein FbpA
**contig_1**	cds	620225	621097	+	GLNHAC_00707	ydbO	Uncharacterized transporter YdbO
**contig_1**	cds	621112	621432	-	GLNHAC_00708	ydbP	Thioredoxin-like protein YdbP
**contig_1**	cds	621586	622671	+	GLNHAC_00709	ddl	D-alanine--D-alanine ligase
**contig_1**	cds	622746	623078	+	GLNHAC_00710		hypothetical protein
**contig_1**	cds	623111	624118	+	GLNHAC_00711		UDP-N-acetylmuramoyl-tripeptide--D-alanyl-D-alanine ligase
**contig_1**	cds	624519	626003	+	GLNHAC_00712	cshA	ATP-dependent RNA helicase CshA
**contig_1**	cds	626175	626654	+	GLNHAC_00713	ydbS	UPF0699 transmembrane protein YdbS
**contig_1**	cds	626644	628125	+	GLNHAC_00714	ydbT	UPF0699 transmembrane protein YdbT
**contig_1**	cds	628122	628721	-	GLNHAC_00715	ydcA	Putative rhomboid protease YdcA
**contig_1**	cds	628816	629181	+	GLNHAC_00716	acpS	Holo-[acyl-carrier-protein] synthase
**contig_1**	cds	629346	630362	+	GLNHAC_00717	ydcC	Sporulation protein YdcC
**contig_1**	cds	630477	631646	+	GLNHAC_00718	alr	alanine racemase
**contig_1**	cds	631762	632043	+	GLNHAC_00719	ndoAI	type II toxin-antitoxin system antitoxin EndoAI
**contig_1**	cds	632046	632396	+	GLNHAC_00720	ndoA	type II toxin-antitoxin system endoribonuclease NdoA
**contig_1**	cds	632518	633339	+	GLNHAC_00721	rsbRA	RsbT co-antagonist protein RsbRA
**contig_1**	cds	633344	633709	+	GLNHAC_00722	rsbS	RsbT antagonist protein RsbS
**contig_1**	cds	633713	634114	+	GLNHAC_00723	rsbT	serine/threonine-protein kinase RsbT
**contig_1**	cds	634126	635133	+	GLNHAC_00724	rsbU	phosphoserine phosphatase RsbU
**contig_1**	cds	635195	635524	+	GLNHAC_00725	rsbV	Anti-sigma-B factor antagonist
**contig_1**	cds	635521	636003	+	GLNHAC_00726	rsbW	anti-sigma B factor RsbW
**contig_1**	cds	635969	636757	+	GLNHAC_00727	sigB	RNA polymerase sigma factor SigB
**contig_1**	cds	636757	637356	+	GLNHAC_00728	rsbX	phosphoserine phosphatase RsbX
**contig_1**	cds	637541	638119	+	GLNHAC_00729		Tex-like protein N-terminal domain-containing protein
**contig_1**	cds	638160	639701	+	GLNHAC_00730		S1 motif domain-containing protein
**contig_1**	cds	639711	639824	-	GLNHAC_00731	cmpA	cortex morphogenetic protein CmpA
**contig_1**	cds	639928	640380	+	GLNHAC_00732	ydcK	SprT family protein
**contig_1**	tRNA	640503	640577	+	GLNHAC_00733	trnN	tRNA-Asn(gtt)
**contig_1**	tRNA	640582	640672	+	GLNHAC_00734	trnS	tRNA-Ser(gct)
**contig_1**	tRNA	640702	640773	+	GLNHAC_00735	trnE	tRNA-Glu(ttc)
**contig_1**	tRNA	640786	640860	+	GLNHAC_00736	trnQ	tRNA-Gln(ttg)
**contig_1**	tRNA	640888	640963	+	GLNHAC_00737	trnK	tRNA-Lys(ttt)
**contig_1**	tRNA	640975	641056	+	GLNHAC_00738	trnL	tRNA-Leu(tag)
**contig_1**	tRNA	641138	641221	+	GLNHAC_00739	trnL	tRNA-Leu(gag)
**contig_1**	cds	641481	642650	-	GLNHAC_00740		Major facilitator superfamily
**contig_1**	cds	642788	643534	-	GLNHAC_00741		Oxidoreductase
**contig_1**	cds	643781	644056	-	GLNHAC_00742		Phosphocarrier protein HPr
**contig_1**	cds	644226	645353	-	GLNHAC_00743		Dienelactone hydrolase domain-containing protein
**contig_1**	cds	645578	646123	+	GLNHAC_00744		HTH tetR-type domain-containing protein
**contig_1**	cds	646165	646386	-	GLNHAC_00745		(pseudo) Spore coat protein
**contig_1**	cds	646419	646559	-	GLNHAC_00746		SMI1/KNR4 family protein
**contig_1**	cds	646779	648065	-	GLNHAC_00747		Amino acid permease family protein
**contig_1**	cds	648219	648662	+	GLNHAC_00748		Transcriptional regulator, Lrp/AsnC family
**contig_1**	cds	649529	650104	+	GLNHAC_00749		TetR-family regulatory protein
**contig_1**	cds	650164	650913	+	GLNHAC_00750		Oxidoreductase, short-chain dehydrogenase/reductase family
**contig_1**	cds	651172	652185	+	GLNHAC_00751		Oxidoreductase, zinc-binding protein
**contig_1**	cds	653030	654031	-	GLNHAC_00752	yddN	Uncharacterized protein YddN
**contig_1**	cds	654105	654704	+	GLNHAC_00753	lrpA	HTH-type transcriptional regulator LrpA
**contig_1**	cds	655054	655818	+	GLNHAC_00754	yddR	UPF0173 protein YddR
**contig_1**	cds	656011	657321	+	GLNHAC_00755	yddS	Uncharacterized MFS-type transporter YddS
**contig_1**	cds	658075	658872	+	GLNHAC_00756		Dihydrodipicolinate synthase family protein, putative
**contig_1**	cds	659021	660274	+	GLNHAC_00757	yeaN	Inner membrane transport protein YeaN
**contig_1**	cds	660371	661261	+	GLNHAC_00758	lysR	Transcriptional regulator, LysR family
**contig_1**	cds	661337	661969	+	GLNHAC_00759	yflK	YflK
**contig_1**	cds	662065	662691	+	GLNHAC_00760	lysE	Transporter, LysE family
**contig_1**	cds	662811	663182	+	GLNHAC_00761		DUF1304 domain-containing protein
**contig_1**	cds	663548	664576	-	GLNHAC_00762	desA	Fatty acid desaturase
**contig_1**	ncRNA-region	665200	665572	+			cspA thermoregulator
**contig_1**	cds	665324	665524	+	GLNHAC_00763	cspC	cold shock protein CspC
**contig_1**	ncRNA	666003	666089	+	GLNHAC_00764	bsrC	BsrC
**contig_1**	cds	666303	666764	-	GLNHAC_00765	ydeB	Putative transcription factor YdeB
**contig_1**	cds	667264	668136	-	GLNHAC_00766	araC	AraC family transcriptional regulator
**contig_1**	cds	668176	668340	+	GLNHAC_00767		hypothetical protein
**contig_1**	cds	668607	668981	+	GLNHAC_00768	ydeH	Uncharacterized membrane protein YdeH
**contig_1**	cds	669368	669961	+	GLNHAC_00769	ydeI	YdeI family protein
**contig_1**	cds	670156	670764	-	GLNHAC_00770		Caax amino protease family
**contig_1**	cds	670896	671069	-	GLNHAC_00771		hypothetical protein
**contig_1**	cds	671370	671555	-	GLNHAC_00772		hypothetical protein
**contig_1**	cds	671678	672391	-	GLNHAC_00773	ydeJ	Uncharacterized lipoprotein YdeJ
**contig_1**	cds	672688	673395	-	GLNHAC_00774		Carbonyl reductase [NADPH] 1
**contig_1**	cds	673645	673920	-	GLNHAC_00775		HPr domain-containing protein
**contig_1**	cds	674254	674670	+	GLNHAC_00776		TetR/AcrR family transcriptional regulator
**contig_1**	cds	674657	674800	+	GLNHAC_00777		TetR-like C-terminal domain-containing protein
**contig_1**	cds	675219	675980	-	GLNHAC_00778		Metallo-beta-lactamase domain-containing protein
**contig_1**	cds	676250	676660	+	GLNHAC_00779	lrpA	HTH-type transcriptional regulator LrpA
**contig_1**	cds	676814	677677	-	GLNHAC_00780	ydeK	Uncharacterized transporter YdeK
**contig_1**	cds	677839	679230	+	GLNHAC_00781		PLP-dependent aminotransferase family protein
**contig_1**	cds	679507	679932	+	GLNHAC_00782	ydeM	Uncharacterized protein YdeM
**contig_1**	cds	679985	680557	-	GLNHAC_00783	ydeN	Putative hydrolase YdeN
**contig_1**	cds	680641	680970	-	GLNHAC_00784	ydzF	Uncharacterized HTH-type transcriptional regulator YdzF
**contig_1**	cds	681225	682097	+	GLNHAC_00785	ydeO	UPF0750 membrane protein YdeO
**contig_1**	cds	682276	682599	-	GLNHAC_00786		Cinnamoyl ester hydrolase
**contig_1**	cds	682667	683083	+	GLNHAC_00787	ydeQ	(pseudo) Uncharacterized NAD(P)H oxidoreductase YdeQ
**contig_1**	cds	683398	683745	+	GLNHAC_00788		Transcriptional regulator (Metals sensing ArsR-SmtB represso
**contig_1**	cds	683758	685065	+	GLNHAC_00789	ydfA	Putative arsenical pump membrane protein YdfA
**contig_1**	cds	685081	685500	+	GLNHAC_00790	arsC	arsenate reductase (thioredoxin)
**contig_1**	cds	685718	686497	+	GLNHAC_00791	ydfB	YdfB
**contig_1**	cds	686576	687463	-	GLNHAC_00792	lytR	Membrane-bound protein LytR
**contig_1**	cds	687676	688506	-	GLNHAC_00793	yheN	YheN
**contig_1**	cds	688755	689888	-	GLNHAC_00794		WD40 repeat domain-containing protein
**contig_1**	cds	689885	691270	-	GLNHAC_00795		histidine kinase
**contig_1**	cds	691339	692043	-	GLNHAC_00796	yclJ	Two-component response regulator YclJ
**contig_1**	cds	692274	693176	-	GLNHAC_00797		Putative permease
**contig_1**	cds	693311	694759	+	GLNHAC_00798		PLP-dependent aminotransferase family protein
**contig_1**	cds	694878	695501	-	GLNHAC_00799		Flavin reductase like domain-containing protein
**contig_1**	cds	695591	696004	+	GLNHAC_00800		HTH arsR-type domain-containing protein
**contig_1**	cds	696334	696777	-	GLNHAC_00801		Carboxymuconolactone decarboxylase-like domain-containin
**contig_1**	cds	696866	697036	-	GLNHAC_00802	ydzP	Uncharacterized membrane protein YdzP
**contig_1**	cds	697541	698704	+	GLNHAC_00803	comP	histidine kinase
**contig_1**	cds	698697	699338	+	GLNHAC_00804	ydfI	two-component system response regulator YdfI
**contig_1**	cds	699460	701631	+	GLNHAC_00805	ydfJ	Membrane protein YdfJ
**contig_1**	cds	701848	701985	+	GLNHAC_00806		MTA/SAH nucleosidase, C-region
**contig_1**	cds	702513	702725	+	GLNHAC_00807		(pseudo) Branched-chain amino acid transporter
**contig_1**	cds	703007	704329	+	GLNHAC_00808	brnQ	branched-chain amino acid transport system II carrier protein
**contig_1**	cds	704455	704928	+	GLNHAC_00809		Rok
**contig_1**	cds	705351	706040	-	GLNHAC_00810	ydfK	Uncharacterized membrane protein YdfK
**contig_1**	cds	706132	706617	-	GLNHAC_00811	merR	(pseudo) MerR family transcriptional regulator
**contig_1**	cds	706641	706943	-	GLNHAC_00812		helix-turn-helix domain-containing protein
**contig_1**	cds	707091	707984	-	GLNHAC_00813	mneP	manganese transporter MneP
**contig_1**	cds	708468	709088	+	GLNHAC_00814	mhqN	Putative NAD(P)H nitroreductase MhqN
**contig_1**	cds	709115	710053	+	GLNHAC_00815	mhqO	Putative ring-cleaving dioxygenase MhqO

contig_1	cds	710190	710579	+	GLNHAC_00816	mhqP	Putative oxidoreductase MhqP
contig_1	cds	710766	711104	+	GLNHAC_00817	ydfQ	Thioredoxin-like protein YdfQ
contig_1	cds	711462	711818	+	GLNHAC_00818		O-antigen polymerase
contig_1	cds	711793	712431	+	GLNHAC_00819		O-methyltransferase
contig_1	cds	712516	712746	-	GLNHAC_00820		Spore germination protein
contig_1	cds	713421	713852	-	GLNHAC_00821	cotP	Spore coat protein P
contig_1	cds	713865	714107	-	GLNHAC_00822		Spore germination protein
contig_1	cds	714121	714393	-	GLNHAC_00823		spore germination protein
contig_1	cds	714634	715233	+	GLNHAC_00824	ydgC	YdgC
contig_1	cds	715230	715574	+	GLNHAC_00825	doxX	DoxX family protein
contig_1	cds	715735	716208	+	GLNHAC_00826	yycN	YycN
contig_1	cds	716219	716548	-	GLNHAC_00827		HIT family protein
contig_1	cds	716904	717929	+	GLNHAC_00828	yisS	YisS
contig_1	cds	718023	719027	+	GLNHAC_00829	degA	HTH-type transcriptional regulator DegA
contig_1	cds	719273	719878	+	GLNHAC_00830		(pseudo) Sugar transporter family protein
contig_1	cds	719875	720456	+	GLNHAC_00831		MFS transporter
contig_1	cds	720472	721293	+	GLNHAC_00832		D-Tagatose 3-epimerase, putative
contig_1	cds	721545	722357	+	GLNHAC_00833	mdtG	Multidrug resistance protein MdtG
contig_1	cds	722748	723314	+	GLNHAC_00834	tetR	Transcriptional regulator, TetR family, putative
contig_1	cds	723354	724982	-	GLNHAC_00835	vmlR	ABC-F type ribosomal protection protein VmlR
contig_1	ncRNA	725010	725211	-	GLNHAC_00836	ncr1575	Bacillus sRNA ncr1575
contig_1	cds	725314	726690	-	GLNHAC_00837	ydgF	Uncharacterized transporter YdgF
contig_1	cds	726865	727389	-	GLNHAC_00838	dinB	damage-inducible protein DinB
contig_1	cds	727547	728008	+	GLNHAC_00839	ydgG	Uncharacterized HTH-type transcriptional regulator YdgG
contig_1	cds	728005	730104	+	GLNHAC_00840	ydgH	(pseudo) Putative membrane protein YdgH
contig_1	cds	730101	730436	+	GLNHAC_00841		hypothetical protein
contig_1	cds	730433	730660	+	GLNHAC_00842		Membrane transport protein MMPL domain-containing protei
contig_1	cds	730825	731454	-	GLNHAC_00843	ydgI	Putative NAD(P)H nitroreductase YdgI
contig_1	cds	731470	731904	-	GLNHAC_00844	ydgJ	Uncharacterized HTH-type transcriptional regulator YdgJ
contig_1	cds	732049	732606	-	GLNHAC_00845		Transcriptional regulator
contig_1	cds	732725	733450	+	GLNHAC_00846	azlC	AzlC family protein
contig_1	cds	733447	733770	+	GLNHAC_00847		Integral membrane protein
contig_1	cds	733964	734845	+	GLNHAC_00848		hypothetical protein
contig_1	cds	734824	735171	+	GLNHAC_00849		hypothetical protein
contig_1	cds	735208	735945	-	GLNHAC_00850	ydhB	putative membrane transporter protein YdhB
contig_1	cds	736194	736868	+	GLNHAC_00851	ydhC	Uncharacterized HTH-type transcriptional regulator YdhC
contig_1	cds	736990	738252	+	GLNHAC_00852	ydhD	Putative sporulation-specific glycosylase YdhD
contig_1	cds	738412	739599	+	GLNHAC_00853	ydhE	Uncharacterized UDP-glucosyltransferase YdhE
contig_1	cds	739637	740347	-	GLNHAC_00854	ydhF	Uncharacterized protein YdhF
contig_1	cds	740413	741801	-	GLNHAC_00855	phoB	alkaline phosphatase PhoB
contig_1	cds	741946	742554	+	GLNHAC_00856		Transglutaminase-like domain-containing protein
contig_1	cds	742590	743075	-	GLNHAC_00857		DUF4944 domain-containing protein
contig_1	cds	743093	743479	-	GLNHAC_00858	yxbD	YxbD
contig_1	cds	743622	744599	+	GLNHAC_00859		HD domain-containing protein
contig_1	cds	744808	745374	+	GLNHAC_00860	ydhK	YdhK family protein
contig_1	cds	745390	746556	-	GLNHAC_00861	pbuE	purine transporter PbuE
contig_1	ncRNA-region	746611	746710	-			Purine riboswitch aptamer
contig_1	cds	746887	747198	+	GLNHAC_00862	gmuB	PTS system oligo-beta-mannoside-specific EIIB component
contig_1	cds	747198	747530	+	GLNHAC_00863	gmuA	PTS system oligo-beta-mannoside-specific EIIA component
contig_1	cds	747551	748528	+	GLNHAC_00864	celB	PTS cellobiose transporter subunit IIC
contig_1	cds	748561	748878	+	GLNHAC_00865		(pseudo) PTS EIIC type-3 domain-containing protein
contig_1	cds	748896	750293	+	GLNHAC_00866	gmuD	6-phospho-beta-glucosidase GmuD
contig_1	cds	750441	750563	+	GLNHAC_00867		hypothetical protein
contig_1	cds	750694	751152	+	GLNHAC_00868	gntR	GntR family transcriptional regulator
contig_1	cds	751181	751294	+	GLNHAC_00869		hypothetical protein
contig_1	cds	751276	752079	+	GLNHAC_00870		fructokinase
contig_1	cds	752076	753047	+	GLNHAC_00871	manA	mannose-6-phosphate isomerase, class I
contig_1	cds	753040	754128	+	GLNHAC_00872	gmuG	Mannan endo-1,4-beta-mannosidase
contig_1	cds	754179	754634	-	GLNHAC_00873		Putative manganese catalase
contig_1	cds	754613	755032	-	GLNHAC_00874		hypothetical protein
contig_1	tRNA	755367	755443	+	GLNHAC_00875	trnR	tRNA-Arg(acg)
contig_1	tRNA	755458	755531	+	GLNHAC_00876	trnG	tRNA-Gly(tcc)
contig_1	ncRNA	755629	755695	-	GLNHAC_00877	Bacillaceae-1	Bacillaceae-1 RNA
contig_1	rRNA	755692	757232	+	GLNHAC_00878	rrs	16S ribosomal RNA
contig_1	rRNA	757402	760323	+	GLNHAC_00879	rrl	23S ribosomal RNA
contig_1	rRNA	760456	760571	+	GLNHAC_00880	rrf	5S ribosomal RNA
contig_1	tRNA	760594	760670	+	GLNHAC_00881	trnfM	tRNA-fMet(cat)
contig_1	tRNA	760732	760808	+	GLNHAC_00882	trnD	tRNA-Asp(gtc)
contig_1	cds	760989	761966	+	GLNHAC_00883	thiL	thiamine-phosphate kinase
contig_1	cds	761981	762457	+	GLNHAC_00884	tsaE	tRNA (adenosine(37)-N6)-threonylcarbamoyltransferase comp
contig_1	cds	762438	763127	+	GLNHAC_00885	tsaB	tRNA (adenosine(37)-N6)-threonylcarbamoyltransferase comp
contig_1	cds	763137	763592	+	GLNHAC_00886	rimI	ribosomal protein S18-alanine N-acetyltransferase
contig_1	cds	763585	764625	+	GLNHAC_00887	tsaD	tRNA (adenosine(37)-N6)-threonylcarbamoyltransferase comp
contig_1	cds	764852	766780	-	GLNHAC_00888	abc-f	ribosomal protection-like ABC-F family protein
contig_1	cds	766906	767418	+	GLNHAC_00889	moaC	cyclic pyranopterin monophosphate synthase MoaC
contig_1	cds	767415	768062	+	GLNHAC_00890	rex	redox-sensing transcriptional repressor Rex
contig_1	cds	768084	768257	+	GLNHAC_00891	tatAY	sec-independent protein translocase protein TatAY
contig_1	cds	768273	769028	+	GLNHAC_00892	tatC	twin-arginine translocase subunit TatC
contig_1	cds	769066	769257	-	GLNHAC_00893	ydiK	Uncharacterized lipoprotein YdiK
contig_1	cds	769254	769988	-	GLNHAC_00894	ydiL	Putative membrane peptidase YdiL
contig_1	cds	770227	770511	+	GLNHAC_00895	groES	co-chaperone GroES
contig_1	cds	770558	772192	+	GLNHAC_00896	groL	chaperonin GroEL
contig_1	cds	773000	773938	+	GLNHAC_00897		DUF4747 domain-containing protein
contig_1	cds	774003	774809	+	GLNHAC_00898		hypothetical protein
contig_1	cds	775034	776998	-	GLNHAC_00899		Tetratricopeptide repeat protein
contig_1	cds	777115	777540	-	GLNHAC_00900		(pseudo) ATP-binding protein
contig_1	cds	777744	778805	+	GLNHAC_00901	gutB	sorbitol dehydrogenase
contig_1	cds	778879	780255	+	GLNHAC_00902	gutA	putative glucitol transport protein GutA
contig_1	cds	780350	781312	+	GLNHAC_00903	ydjE	Uncharacterized sugar kinase YdjE
contig_1	cds	781508	782191	+	GLNHAC_00904	ydjF	Phage shock A-like protein
contig_1	cds	782257	783282	+	GLNHAC_00905	ydjG	Uncharacterized protein YdjG
contig_1	cds	783282	784046	+	GLNHAC_00906	ygcG	YgcG family protein
contig_1	cds	784077	785033	+	GLNHAC_00907	ydjI	Uncharacterized protein YdjI
contig_1	cds	785081	786094	-	GLNHAC_00908	ydjJ	Uncharacterized membrane protein YdjJ
contig_1	cds	786707	788128	+	GLNHAC_00909	iolT	myo-inositol transporter IolT
contig_1	cds	788177	789217	-	GLNHAC_00910	bdhA	(R,R)-butanediol dehydrogenase
contig_1	cds	789661	790032	+	GLNHAC_00911	ydjM	cell wall metabolism protein WalM
contig_1	cds	790098	791144	+	GLNHAC_00912	ydjN	Uncharacterized protein YdjN
contig_1	cds	791177	791335	-	GLNHAC_00913		Conserved domain protein
contig_1	cds	791522	791767	-	GLNHAC_00914	ydjO	Uncharacterized protein YdjO
contig_1	cds	791814	792629	-	GLNHAC_00915	ydjP	AB hydrolase superfamily protein YdjP
contig_1	cds	792645	793634	-	GLNHAC_00916	yeaA	Uncharacterized protein YeaA
contig_1	cds	793732	795273	-	GLNHAC_00917	cotA	outer spore coat copper-dependent laccase CotA
contig_1	cds	795426	796832	-	GLNHAC_00918	gabP	GABA permease
contig_1	cds	797246	798118	+	GLNHAC_00919	mneS	manganese transporter MneS
contig_1	cds	798269	799231	+	GLNHAC_00920	yeaC	Uncharacterized protein YeaC
contig_1	cds	799231	799482	+	GLNHAC_00921		hypothetical protein
contig_1	cds	799924	800427	+	GLNHAC_00922		(pseudo) DUF58 domain-containing protein
contig_1	cds	800449	801960	+	GLNHAC_00923	yebA	(pseudo) Uncharacterized protein YebA
contig_1	cds	802082	802654	+	GLNHAC_00924		hypothetical protein
contig_1	cds	802808	804358	+	GLNHAC_00925	guaA	glutamine-hydrolyzing GMP synthase
contig_1	ncRNA-region	804502	804603	+			Purine riboswitch aptamer
contig_1	cds	804739	806061	+	GLNHAC_00926	pbuG	hypoxanthine/guanine permease PbuG
contig_1	cds	806271	807074	+	GLNHAC_00927	yebC	Uncharacterized protein YebC
contig_1	cds	807233	807400	+	GLNHAC_00928	yebD	Uncharacterized protein YebD
contig_1	cds	807615	808169	+	GLNHAC_00929	yebE	UPF0316 protein RBAM_006820
contig_1	cds	808169	808366	+	GLNHAC_00930	yebG	Uncharacterized protein YebG
contig_1	ncRNA-region	808446	808547	+			Purine riboswitch aptamer
contig_1	cds	808688	809176	+	GLNHAC_00931	purE	N5-carboxyaminoimidazole ribonucleotide mutase
contig_1	cds	809160	810311	+	GLNHAC_00932	purK	5-(carboxyamino)imidazole ribonucleotide synthase
contig_1	cds	810308	811603	+	GLNHAC_00933	purB	adenylosuccinate lyase
contig_1	cds	811678	812403	+	GLNHAC_00934	purC	phosphoribosylaminoimidazolesuccinocarboxamide synthase
contig_1	cds	812396	812650	+	GLNHAC_00935	purS	phosphoribosylformylglycinamidine synthase subunit PurS
contig_1	cds	812647	813330	+	GLNHAC_00936	purQ	phosphoribosylformylglycinamidine synthase subunit PurQ
contig_1	cds	813314	815542	+	GLNHAC_00937	purL	phosphoribosylformylglycinamidine synthase subunit PurL
contig_1	cds	815518	816948	+	GLNHAC_00938	purF	amidophosphoribosyltransferase
contig_1	cds	817049	818089	+	GLNHAC_00939	purM	Phosphoribosylformylglycinamidine cyclo-ligase
contig_1	cds	818086	818673	+	GLNHAC_00940	purN	phosphoribosylglycinamide formyltransferase
contig_1	cds	818670	820208	+	GLNHAC_00941	purH	bifunctional phosphoribosylaminoimidazolecarboxamide form
contig_1	cds	820224	821492	+	GLNHAC_00942	purD	Phosphoribosylamine--glycine ligase
contig_1	cds	821531	821953	-	GLNHAC_00943	yezC	Uncharacterized HTH-type transcriptional regulator YezC
contig_1	cds	822097	823371	+	GLNHAC_00944		APC family permease
contig_1	cds	823386	823619	-	GLNHAC_00945	yezF	Uncharacterized membrane protein YezF
contig_1	cds	823749	825491	+	GLNHAC_00946	yerA	Putative adenine deaminase YerA
contig_1	cds	825652	826512	+	GLNHAC_00947	yerB	Putative lipoprotein YerB
contig_1	cds	826515	826829	+	GLNHAC_00948	yerC	Uncharacterized protein YerC
contig_1	cds	826867	828444	-	GLNHAC_00949	yerD	Glutamate synthase large subunit-like protein YerD
contig_1	cds	828690	829376	+	GLNHAC_00950	pcrB	heptaprenylglyceryl phosphate synthase
contig_1	cds	829438	831657	+	GLNHAC_00951	pcrA	DNA helicase PcrA

contig_1	cds	831681	833687	+	GLNHAC_00952	ligA	NAD-dependent DNA ligase LigA
contig_1	cds	833770	834078	+	GLNHAC_00953		hypothetical protein
contig_1	cds	834120	834893	+	GLNHAC_00954	yerH	(pseudo) Uncharacterized lipoprotein YerH
contig_1	cds	835078	836079	+	GLNHAC_00955	amiN	Amicoumacin kinase
contig_1	cds	836117	836809	-	GLNHAC_00956	sapB	Protein SapB
contig_1	cds	836921	838399	-	GLNHAC_00957	putP	sodium/proline symporter PutP
contig_1	cds	838814	839104	+	GLNHAC_00958	gatC	Asp-tRNA(Asn)/Glu-tRNA(Gln) amidotransferase subunit GatC
contig_1	cds	839120	840577	+	GLNHAC_00959	gatA	Asp-tRNA(Asn)/Glu-tRNA(Gln) amidotransferase subunit GatA
contig_1	cds	840591	842021	+	GLNHAC_00960	gatB	Asp-tRNA(Asn)/Glu-tRNA(Gln) amidotransferase subunit GatB
contig_1	cds	842164	842901	-	GLNHAC_00961	yerO	Uncharacterized HTH-type transcriptional regulator YerO
contig_1	cds	843033	844910	+	GLNHAC_00962		efflux RND transporter permease subunit
contig_1	cds	844886	846172	+	GLNHAC_00963	swrC	Swarming motility protein SwrC
contig_1	cds	846324	846965	+	GLNHAC_00964	dagK	(pseudo) diacylglycerol kinase
contig_1	cds	847034	847234	+	GLNHAC_00965		hypothetical protein
contig_1	cds	847292	848674	+	GLNHAC_00966	rlmD	23S rRNA (uracil(1939)-C(5))-methyltransferase RlmD
contig_1	cds	848774	849934	+	GLNHAC_00967	mcrA	McrA
contig_1	cds	850476	850871	-	GLNHAC_00968		Immunity 22 family protein
contig_1	cds	850884	852020	-	GLNHAC_00969		(pseudo) LXG domain-containing protein
contig_1	cds	852025	852774	-	GLNHAC_00970	yeeF	ribonuclease YeeF family protein
contig_1	cds	853219	854007	+	GLNHAC_00971	yeeG	Uncharacterized protein YeeG
contig_1	cds	854171	855313	+	GLNHAC_00972	rapH	Response regulator aspartate phosphatase H
contig_1	cds	855303	855482	+	GLNHAC_00973	rapH	Phosphatase RapH inhibitor
contig_1	cds	855586	856362	+	GLNHAC_00974	yeeI	YebC/PmpR family DNA-binding transcriptional regulator
contig_1	cds	856498	856755	+	GLNHAC_00975		hypothetical protein
contig_1	cds	856752	856940	+	GLNHAC_00976		hypothetical protein
contig_1	cds	857053	857637	+	GLNHAC_00977	yezE	Uncharacterized HTH-type transcriptional regulator YezE
contig_1	cds	857715	858158	+	GLNHAC_00978	yesE	Uncharacterized protein YesE
contig_1	cds	858155	859003	+	GLNHAC_00979	nmrA	NmrA family NAD(P)-binding protein
contig_1	cds	859163	859411	+	GLNHAC_00980	cotJA	spore coat-associated protein CotJA
contig_1	cds	859395	859658	+	GLNHAC_00981	cotJB	spore coat protein CotJB
contig_1	cds	859673	860242	+	GLNHAC_00982	cotJC	spore coat protein CotJC
contig_1	cds	860367	860909	+	GLNHAC_00983	yesJ	Uncharacterized N-acetyltransferase YesJ
contig_1	cds	861129	861752	+	GLNHAC_00984	yesL	Uncharacterized protein YesL
contig_1	cds	861749	863482	+	GLNHAC_00985	yesM	two-component system sensor histidine kinase YesM
contig_1	cds	863482	864588	+	GLNHAC_00986	yesN	two-component system response regulator YesN
contig_1	cds	864692	865975	+	GLNHAC_00987	yesO	Putative ABC transporter substrate-binding protein YesO
contig_1	cds	865996	866286	+	GLNHAC_00988		(pseudo) carbohydrate ABC transporter permease
contig_1	cds	866361	866900	+	GLNHAC_00989		Sugar ABC transporter permease
contig_1	cds	866904	867794	+	GLNHAC_00990	yesQ	putative ABC transporter permease protein YesQ
contig_1	cds	867811	868803	+	GLNHAC_00991	yesR	Unsaturated rhamnogalacturonyl hydrolase YesR
contig_1	cds	868859	869089	+	GLNHAC_00992		hypothetical protein
contig_1	cds	869038	870561	+	GLNHAC_00993	yesS	(pseudo) HTH-type transcriptional regulator YesS
contig_1	cds	870558	871142	+	GLNHAC_00994		hypothetical protein
contig_1	cds	871158	871400	+	GLNHAC_00995		hypothetical protein
contig_1	cds	871842	872048	+	GLNHAC_00996		hypothetical protein
contig_1	cds	872492	873118	+	GLNHAC_00997	yesV	Uncharacterized protein YesV
contig_1	cds	873257	874432	+	GLNHAC_00998		Rhamnogalacturonan lyase family 11 C-terminal domain-cont
contig_1	cds	874387	874770	+	GLNHAC_00999		hypothetical protein
contig_1	cds	874893	875096	+	GLNHAC_01000		putative rhamnogalacturonan lyase in rhamnose utilization clu
contig_1	cds	875515	876978	+	GLNHAC_01001	yesX	(pseudo) Rhamnogalacturonan exolyase YesX
contig_1	cds	877134	877787	+	GLNHAC_01002	yesY	putative rhamnogalacturonan acetylesterase YesY
contig_1	cds	877793	879787	+	GLNHAC_01003	yesZ	Beta-galactosidase YesZ
contig_1	cds	879832	882405	+	GLNHAC_01004	yetA	Uncharacterized protein YetA
contig_1	cds	882527	884035	+	GLNHAC_01005	lipO	Lipoprotein LipO
contig_1	cds	884090	884440	+	GLNHAC_01006		hypothetical protein
contig_1	cds	884632	885048	+	GLNHAC_01007	lplB	(pseudo) Protein LplB
contig_1	cds	885062	885949	+	GLNHAC_01008	lplC	Protein LplC
contig_1	cds	885958	886827	+	GLNHAC_01009		Alpha-glucosidase/alpha-galactosidase
contig_1	cds	886775	887299	+	GLNHAC_01010		hypothetical protein
contig_1	cds	887376	888071	+	GLNHAC_01011	yetF	YetF
contig_1	cds	888110	888433	-	GLNHAC_01012	hmoA	heme-degrading oxygenase HmoA
contig_1	cds	888534	888896	-	GLNHAC_01013	yetH	Uncharacterized protein YetH
contig_1	cds	888992	889519	-	GLNHAC_01014		hypothetical protein
contig_1	cds	889696	889887	+	GLNHAC_01015		hypothetical protein
contig_1	cds	889913	890569	+	GLNHAC_01016	yetI	YetI
contig_1	cds	890677	890916	-	GLNHAC_01017		hypothetical protein
contig_1	cds	891001	891645	+	GLNHAC_01018	yetJ	Uncharacterized protein YetJ
contig_1	cds	891710	892636	+	GLNHAC_01019	yetK	Uncharacterized transporter YetK
contig_1	cds	892660	893163	-	GLNHAC_01020	yetL	HTH-type transcriptional repressor YetL
contig_1	cds	893323	894438	+	GLNHAC_01021	yetM	Putative FAD-dependent monooxygenase YetM
contig_1	cds	894759	895865	+	GLNHAC_01022		Restriction endonuclease
contig_1	cds	896102	897172	-	GLNHAC_01023	yetN	Uncharacterized protein YetN
contig_1	cds	897322	900507	+	GLNHAC_01024	cypD	bifunctional P-450/NADPH--P450 reductase
contig_1	cds	900943	902862	+	GLNHAC_01025	ltaS1	lipoteichoic acid synthase LtaS1
contig_1	cds	903099	903863	+	GLNHAC_01026	yfnH	putative glucose-1-phosphate cytidylyltransferase
contig_1	cds	903936	904838	+	GLNHAC_01027	yfnG	Putative sugar dehydratase/epimerase YfnG
contig_1	cds	904856	905773	+	GLNHAC_01028	yfnF	Uncharacterized protein YfnF
contig_1	cds	905802	905975	+	GLNHAC_01029		(pseudo) glycosyltransferase family 2 protein
contig_1	cds	905942	906964	+	GLNHAC_01030	yfnE	Uncharacterized glycosyltransferase YfnE
contig_1	cds	906979	907914	+	GLNHAC_01031	yfnD	Uncharacterized protein YfnD
contig_1	cds	907943	909172	-	GLNHAC_01032	yfnC	Uncharacterized MFS-type transporter YfnC
contig_1	cds	909283	909990	-	GLNHAC_01033	yfnB	YjjG family noncanonical pyrimidine nucleotidase
contig_1	cds	910083	911468	-	GLNHAC_01034	mtrA	Methylthioribose transporter
contig_1	cds	911717	913174	+	GLNHAC_01035	yfmT	benzaldehyde dehydrogenase
contig_1	cds	913194	914048	+	GLNHAC_01036	yfmS	Putative sensory transducer protein YfmS
contig_1	cds	914183	915907	+	GLNHAC_01037	abc-f	ribosomal protection-like ABC-F family protein
contig_1	cds	916195	916641	+	GLNHAC_01038	yfmQ	YfmQ family protein
contig_1	cds	916765	917187	+	GLNHAC_01039	yfmP	HTH-type transcriptional regulator YfmP
contig_1	cds	917344	918441	+	GLNHAC_01040	yfmO	Multidrug efflux protein YfmO
contig_1	cds	918476	919021	-	GLNHAC_01041		Acetyltransferase, gnat family
contig_1	cds	919463	921019	-	GLNHAC_01042	abc-f	ribosomal protection-like ABC-F family protein
contig_1	cds	921191	922321	+	GLNHAC_01043	yfmL	putative ATP-dependent RNA helicase YfmL
contig_1	cds	922389	922829	+	GLNHAC_01044	yfmK	YfmK
contig_1	cds	922882	923901	-	GLNHAC_01045		Putative NADP-dependent oxidoreductase yncb
contig_1	cds	924384	925604	-	GLNHAC_01046	yfmI	Uncharacterized MFS-type transporter YfmI
contig_1	cds	925941	926933	+	GLNHAC_01047		SUMF1/EgtB/PvdO family nonheme iron enzyme
contig_1	cds	927983	928429	-	GLNHAC_01048		ABC transporter domain-containing protein
contig_1	cds	928423	928782	-	GLNHAC_01049	fepC	Ferric enterobactin transport ATP-binding protein fepC
contig_1	cds	928795	929796	-	GLNHAC_01050	yfmE	Fe(3+)-citrate ABC transporter permease YfmE
contig_1	cds	929793	930794	-	GLNHAC_01051	yfmD	Fe(3+) dicitrate ABC transporter permease FecD
contig_1	cds	930867	931814	-	GLNHAC_01052	yfmC	Fe(3+)-citrate ABC transporter substrate-binding protein YfmC
contig_1	cds	931924	932292	-	GLNHAC_01053		DUF3212 domain-containing protein
contig_1	cds	932533	932880	+	GLNHAC_01054	yflT	General stress protein 17M
contig_1	cds	933074	934336	+	GLNHAC_01055	pel	pectate lyase
contig_1	cds	934463	935899	+	GLNHAC_01056	yflS	Putative malate transporter YflS
contig_1	cds	936079	937665	+	GLNHAC_01057	citS	Sensor protein CitS
contig_1	cds	937679	938359	+	GLNHAC_01058	citT	two-component response regulator CitT
contig_1	cds	938362	939321	+	GLNHAC_01059	yflP	UPF0065 protein YflP
contig_1	cds	939511	940812	+	GLNHAC_01060	citM	citrate transporter CitM
contig_1	cds	940868	941662	+	GLNHAC_01061	yflN	putative metallo-hydrolase YflN
contig_1	cds	941783	942874	+	GLNHAC_01062	nos	Nitric oxide synthase oxygenase
contig_1	cds	942960	943139	-	GLNHAC_01063		hypothetical protein
contig_1	cds	943207	943872	+	GLNHAC_01064	yflK	Uncharacterized protein YflK
contig_1	cds	943919	944050	-	GLNHAC_01065	yflJ	Uncharacterized protein YflJ
contig_1	cds	944223	944468	-	GLNHAC_01066		hypothetical protein
contig_1	cds	944488	944802	-	GLNHAC_01067		DUF3243 domain-containing protein
contig_1	cds	944883	945632	-	GLNHAC_01068	map	type I methionyl aminopeptidase
contig_1	cds	945803	946414	+	GLNHAC_01069		hypothetical protein
contig_1	cds	946466	947158	+	GLNHAC_01070		PTS sugar transporter
contig_1	cds	947192	949141	-	GLNHAC_01071	ltaS	lipoteichoic acid synthase
contig_1	cds	949401	949793	+	GLNHAC_01072	yflB	Uncharacterized protein YflB
contig_1	cds	949921	951336	+	GLNHAC_01073	yflA	Uncharacterized transporter YflA
contig_1	cds	951333	951755	-	GLNHAC_01074		hypothetical protein
contig_1	cds	952517	952621	+	GLNHAC_01075		hypothetical protein
contig_1	cds	952895	953926	+	GLNHAC_01076		PTS system trehalose-specific transporter subunit IIBC
contig_1	cds	954000	955352	+	GLNHAC_01077		alpha,alpha-phosphotrehalase
contig_1	cds	955709	956425	+	GLNHAC_01078	treR	trehalose operon repressor
contig_1	cds	956566	957231	+	GLNHAC_01079	yfkO	Putative NAD(P)H nitroreductase YfkO
contig_1	cds	957269	961657	-	GLNHAC_01080	yfkN	multifunctional 2',3'-cyclic-nucleotide 2'-phosphodiesterase/3
contig_1	cds	961901	962419	+	GLNHAC_01081	yfkM	general stress protein 18
contig_1	cds	962456	963643	-	GLNHAC_01082	yfkL	Uncharacterized MFS-type transporter YfkL
contig_1	cds	963858	964073	-	GLNHAC_01083	yfkK	UPF0435 protein YfkK
contig_1	cds	964277	964747	+	GLNHAC_01084	yfkJ	Low molecular weight protein-tyrosine-phosphatase YfkJ
contig_1	cds	964765	965085	+	GLNHAC_01085	yfkI	Uncharacterized protein YfkI
contig_1	cds	965109	965936	+	GLNHAC_01086	yihY	YihY family inner membrane protein
contig_1	cds	965989	966096	+	GLNHAC_01087	yuzL	YuzL family protein
contig_1	cds	966136	967311	-	GLNHAC_01088	yfkF	Uncharacterized MFS-type transporter YfkF
contig_1	cds	967479	968534	+	GLNHAC_01089	cax	calcium/proton exchanger
contig_1	cds	968608	969399	+	GLNHAC_01090	yfkD	Uncharacterized protein YfkD

contig_1	cds	969438	970274	-	GLNHAC_01091	yfkC	Uncharacterized MscS family protein YfkC
contig_1	cds	970281	971402	-	GLNHAC_01092	yfkAB	radical SAM/CxCxxxxC motif protein YfkAB
contig_1	cds	971548	971733	+	GLNHAC_01093	yfjT	Uncharacterized protein YfjT
contig_1	cds	971834	972643	+	GLNHAC_01094	pdaA	delta-lactam-biosynthetic de-N-acetylase
contig_1	cds	972662	973609	-	GLNHAC_01095	yfjR	Uncharacterized oxidoreductase YfjR
contig_1	cds	973617	974390	-	GLNHAC_01096	yfjQ	Putative metal ion transporter YfjQ
contig_1	cds	974342	974575	-	GLNHAC_01097		hypothetical protein
contig_1	cds	974692	975555	+	GLNHAC_01098	yfjP	Putative DNA-3-methyladenine glycosylase YfjP
contig_1	cds	975668	977068	+	GLNHAC_01099	rlmD	23S rRNA (uracil(1939)-C(5))-methyltransferase RlmD
contig_1	cds	977787	978071	+	GLNHAC_01100	yfzA	Uncharacterized membrane protein YfzA
contig_1	cds	978811	979794	+	GLNHAC_01101	dus2	putative tRNA-dihydrouridine synthase 2
contig_1	cds	980024	980575	+	GLNHAC_01102		Ribosomal-protein-alanine N-acetyltransferase
contig_1	cds	980671	981123	+	GLNHAC_01103	yfjM	Uncharacterized protein YfjM
contig_1	cds	981177	981839	+	GLNHAC_01104	yfjL	Uncharacterized protein YfjL
contig_1	cds	982067	983068	+	GLNHAC_01105	acoA	acetoin:2,6-dichlorophenolindophenol oxidoreductase subuni
contig_1	cds	983070	984098	+	GLNHAC_01106	acoB	acetoin:2,6-dichlorophenolindophenol oxidoreductase subuni
contig_1	cds	984112	985308	+	GLNHAC_01107	acoC	Dihydrolipoyllysine-residue acetyltransferase component of a
contig_1	cds	985329	986705	+	GLNHAC_01108	acoL	acetoin dehydrogenase complex dihydrolipoyl dehydrogenase
contig_1	cds	986821	987150	+	GLNHAC_01109		hypothetical protein
contig_1	cds	987137	987598	+	GLNHAC_01110		hypothetical protein
contig_1	cds	987595	988638	+	GLNHAC_01111		Acetoin operon expression regulatory protein
contig_1	cds	988691	988870	+	GLNHAC_01112		small acid-soluble spore protein H
contig_1	cds	988929	989231	-	GLNHAC_01113		UPF0060 membrane protein BH2744
contig_1	cds	989284	989841	-	GLNHAC_01114		Integral inner membrane protein
contig_1	cds	989865	990632	-	GLNHAC_01115	yfjC	Uncharacterized protein YfjC
contig_1	cds	990644	991867	-	GLNHAC_01116	yfjB	Uncharacterized protein YfjB
contig_1	cds	991873	992187	-	GLNHAC_01117	yfjA	Uncharacterized protein YfjA
contig_1	cds	992523	993872	+	GLNHAC_01118	glvA	maltose-6'-phosphate glucosidase
contig_1	cds	994179	994700	+	GLNHAC_01119		MurR/RpiR family transcriptional regulator
contig_1	cds	994715	995389	+	GLNHAC_01120		PTS transporter subunit EIIC
contig_1	cds	995386	996297	+	GLNHAC_01121	malP	(pseudo) PTS system maltose-specific EIICB component
contig_1	cds	996403	998124	+	GLNHAC_01122	yfiB	Uncharacterized ABC transporter ATP-binding protein YfiB
contig_1	cds	998118	999932	+	GLNHAC_01123	yfiC	Uncharacterized ABC transporter ATP-binding protein YfiC
contig_1	cds	100008	100049	+	GLNHAC_01124	catD	DoxX family oxidoreductase CatD
contig_1	cds	100050	100136	+	GLNHAC_01125	catE	Catechol-2,3-dioxygenase
contig_1	cds	100161	100269	+	GLNHAC_01126		sensor histidine kinase
contig_1	cds	100269	100335	+	GLNHAC_01127	lnrK	two-component system response regulator LnrK
contig_1	cds	100349	100443	+	GLNHAC_01128	lnrL	linearmycin resistance ATP-binding protein LnrL
contig_1	cds	100444	100563	+	GLNHAC_01129		ABC-2 type transporter family
contig_1	cds	100565	100662	+	GLNHAC_01130		Putative ABC transporter (Permease)
contig_1	cds	100684	100743	-	GLNHAC_01131		Acetyltransferase, gnat family
contig_1	cds	100748	100803	-	GLNHAC_01132	padR	transcriptional regulator PadR
contig_1	cds	100831	100894	+	GLNHAC_01133	estB	Extracellular esterase EstB
contig_1	cds	100916	101025	+	GLNHAC_01134	yfiQ	Putative membrane-bound acyltransferase YfiQ
contig_1	cds	101038	101097	+	GLNHAC_01135	yfiT	YfiT family bacillithiol transferase
contig_1	cds	101125	101153	-	GLNHAC_01136		hypothetical protein
contig_1	cds	101153	101248	-	GLNHAC_01137		Major facilitator superfamily (MFS) profile domain-containing p
contig_1	cds	101260	101307	-	GLNHAC_01138	yfiV	Uncharacterized HTH-type transcriptional regulator YfiV
contig_1	cds	101324	101486	+	GLNHAC_01139		phosphatidylglycerol lysyltransferase domain-containing prote
contig_1	cds	101509	101526	+	GLNHAC_01140		hypothetical protein
contig_1	cds	101527	101577	+	GLNHAC_01141		hypothetical protein
contig_1	cds	101594	101680	-	GLNHAC_01142	yfiY	(pseudo) putative siderophore-binding lipoprotein YfiY
contig_1	cds	101693	101793	+	GLNHAC_01143	yfiZ	putative siderophore transport system permease protein YfiZ
contig_1	cds	101793	101896	+	GLNHAC_01144	yfhA	putative siderophore transport system permease protein YfhA
contig_1	cds	101907	101995	+	GLNHAC_01145	yfhB	YfhB
contig_1	cds	102004	102062	+	GLNHAC_01146	yfhC	Putative NAD(P)H nitroreductase YfhC
contig_1	cds	102066	102085	-	GLNHAC_01147	yfhD	Uncharacterized protein YfhD
contig_1	cds	102092	102103	-	GLNHAC_01148	yfhE	Uncharacterized protein YfhE
contig_1	cds	102109	102200	-	GLNHAC_01149	yfhF	Epimerase family protein YfhF
contig_1	cds	102209	102237	+	GLNHAC_01150		hypothetical protein
contig_1	cds	102247	102288	+	GLNHAC_01151	recX	(pseudo) recombination regulator RecX
contig_1	cds	102289	102320	+	GLNHAC_01152	yfhH	Uncharacterized protein YfhH
contig_1	cds	102334	102454	+	GLNHAC_01153	yfhI	Uncharacterized MFS-type transporter YfhI
contig_1	cds	102457	102472	-	GLNHAC_01154	sspK	small, acid-soluble spore protein K
contig_1	cds	102485	102511	+	GLNHAC_01155	yfhJ	Uncharacterized protein YfhJ
contig_1	cds	102526	102578	+	GLNHAC_01156		SH3b domain-containing protein
contig_1	cds	102580	102620	+	GLNHAC_01157	yfhL	Uncharacterized protein YfhL
contig_1	cds	102618	102704	+	GLNHAC_01158	yfhM	AB hydrolase superfamily protein YfhM
contig_1	cds	102727	102826	+	GLNHAC_01159	csbB	Putative glycosyltransferase CsbB
contig_1	cds	102833	103091	+	GLNHAC_01160		Membrane protein
contig_1	cds	103091	103189	-	GLNHAC_01161	yfhP	Uncharacterized protein YfhP
contig_1	cds	103194	103322	+	GLNHAC_01162	mutY	A/G-specific adenine glycosylase
contig_1	cds	103322	103345	-	GLNHAC_01163	yfhS	Uncharacterized protein YfhS
contig_1	cds	103353	103428	+	GLNHAC_01164	fabL	enoyl-[acyl-carrier-protein] reductase FabL
contig_1	cds	103435	103461	+	GLNHAC_01165	sspE	Small, acid-soluble spore protein gamma-type
contig_1	cds	103478	103504	+	GLNHAC_01166		YgaB-like protein
contig_1	cds	103518	103571	+	GLNHAC_01167	ygaC	Nucleoside triphosphate/diphosphate phosphatase
contig_1	cds	103580	103754	+	GLNHAC_01168	ygaD	Putative multidrug export ATP-binding/permease protein Ygal
contig_1	cds	103762	103873	-	GLNHAC_01169		Aromatic acid exporter family protein
contig_1	cds	103890	103982	-	GLNHAC_01170		Glutamate-1-semialdehyde aminotransferase
contig_1	cds	103977	104019	-	GLNHAC_01171		(pseudo) aminotransferase class III-fold pyridoxal phosphate-
contig_1	cds	104034	104082	+	GLNHAC_01172	bcp	thioredoxin-dependent thiol peroxidase
contig_1	cds	104095	104139	+	GLNHAC_01173	perR	peroxide-responsive transcriptional repressor PerR
contig_1	cds	104142	104177	-	GLNHAC_01174	ygzB	UPF0295 protein YgzB
contig_1	cds	104198	104287	+	GLNHAC_01175	ygxA	Uncharacterized protein YgxA
contig_1	ncRNA	104310	104316	-	GLNHAC_01176	Bacillaceae-1	Bacillaceae-1 RNA
contig_1	rRNA	104316	104471	+	GLNHAC_01177	rrs	16S ribosomal RNA
contig_1	rRNA	104488	104780	+	GLNHAC_01178	rrl	23S ribosomal RNA
contig_1	rRNA	104791	104803	+	GLNHAC_01179	rrf	5S ribosomal RNA
contig_1	tRNA	104804	104811	+	GLNHAC_01180	trnN	tRNA-Asn(gtt)
contig_1	tRNA	104812	104821	+	GLNHAC_01181	trnS	tRNA-Ser(gga)
contig_1	tRNA	104824	104831	+	GLNHAC_01182	trnE	tRNA-Glu(ttc)
contig_1	tRNA	104832	104840	+	GLNHAC_01183	trnV	tRNA-Val(tac)
contig_1	tRNA	104841	104848	+	GLNHAC_01184	trnfM	tRNA-fMet(cat)
contig_1	tRNA	104855	104862	+	GLNHAC_01185	trnD	tRNA-Asp(gtc)
contig_1	tRNA	104863	104871	+	GLNHAC_01186	trnF	tRNA-Phe(gaa)
contig_1	tRNA	104872	104879	+	GLNHAC_01187	trnT	tRNA-Thr(tgt)
contig_1	tRNA	104881	104889	+	GLNHAC_01188	trnY	tRNA-Tyr(gta)
contig_1	tRNA	104890	104897	+	GLNHAC_01189	trnW	tRNA-Trp(cca)
contig_1	tRNA	104900	104907	+	GLNHAC_01190	trnH	tRNA-His(gtg)
contig_1	tRNA	104908	104915	+	GLNHAC_01191	trnQ	tRNA-Gln(ttg)
contig_1	tRNA	104920	104928	+	GLNHAC_01192	trnG	tRNA-Gly(gcc)
contig_1	tRNA	104928	104935	+	GLNHAC_01193	trnC	tRNA-Cys(gca)
contig_1	tRNA	104936	104945	+	GLNHAC_01194	trnL	tRNA-Leu(taa)
contig_1	tRNA	104973	104981	+	GLNHAC_01195	trnL	tRNA-Leu(caa)
contig_1	cds	104990	105068	-	GLNHAC_01196	spo0M	sporulation control protein Spo0M
contig_1	cds	105083	105102	+	GLNHAC_01197	ygzA	Uncharacterized protein YgzA
contig_1	cds	105108	105133	-	GLNHAC_01198		Spore coat protein
contig_1	cds	105145	105214	+	GLNHAC_01199		Peptidase E
contig_1	ncRNA-region	105221	105232	+			TPP riboswitch aptamer (THI element)
contig_1	cds	105245	105346	+	GLNHAC_01200	thiC	(pseudo) phosphomethylpyrimidine synthase ThiC
contig_1	cds	105340	105402	+	GLNHAC_01201		Phosphomethylpyrimidine synthase
contig_1	cds	105436	105454	+	GLNHAC_01202		Transcriptional regulator (Activator)
contig_1	cds	105458	105603	-	GLNHAC_01203	katA	catalase KatA
contig_1	cds	105631	105721	+	GLNHAC_01204	ssuB	Aliphatic sulfonates import ATP-binding protein SsuB
contig_1	cds	105722	105822	+	GLNHAC_01205	ssuA	Putative aliphatic sulfonates-binding protein
contig_1	cds	105822	105905	+	GLNHAC_01206		ABC transporter permease
contig_1	cds	105907	106021	+	GLNHAC_01207	ssuD	FMNH2-dependent alkanesulfonate monooxygenase
contig_1	cds	106034	106084	+	GLNHAC_01208	ygaN	Uncharacterized protein YgaN
contig_1	cds	106096	106122	+	GLNHAC_01209	rpsN	30S ribosomal protein S14
contig_1	cds	106124	106172	-	GLNHAC_01210	ygaO	Uncharacterized lipoprotein YgaO
contig_1	cds	106172	106192	-	GLNHAC_01211	ygzD	Uncharacterized HTH-type transcriptional regulator YgzD
contig_1	tRNA	106211	106218	+	GLNHAC_01212	trnG	tRNA-Gly(tcc)
contig_1	cds	106227	106290	-	GLNHAC_01213	yhzB	Uncharacterized protein YhzB
contig_1	cds	106298	106334	+	GLNHAC_01214		(pseudo) DUF1730 domain-containing protein
contig_1	cds	106332	106406	+	GLNHAC_01215		(Fe-S)-binding protein
contig_1	cds	106422	106514	+	GLNHAC_01216	yhbB	Uncharacterized protein YhbB
contig_1	cds	106518	106566	+	GLNHAC_01217	trmL	tRNA (uridine(34)/cytosine(34)/5-carboxymethylaminomethylu
contig_1	cds	106578	106642	+	GLNHAC_01218	yhbD	YhbD family protein
contig_1	cds	106642	106713	+	GLNHAC_01219	yhbE	Uncharacterized protein YhbE
contig_1	cds	106714	106785	+	GLNHAC_01220		Cytoplasmic protein
contig_1	cds	106820	107009	+	GLNHAC_01221	prkA	serine/threonine protein kinase PrkA
contig_1	cds	107027	107145	+	GLNHAC_01222	yhbH	sporulation protein YhbH
contig_1	cds	107161	107208	+	GLNHAC_01223	yhbI	Uncharacterized HTH-type transcriptional regulator YhbI
contig_1	cds	107215	107278	+	GLNHAC_01224	yhbJ	Putative efflux system component YhbJ
contig_1	cds	107282	107442	+	GLNHAC_01225		MFS transporter
contig_1	cds	107444	107497	+	GLNHAC_01226	yhcB	Uncharacterized protein YhcB
contig_1	cds	107498	107536	+	GLNHAC_01227	yhcC	Uncharacterized protein YhcC
contig_1	cds	107552	107629	+	GLNHAC_01228		ABC transporter permease

contig_1	cds	107629	107665	+	GLNHAC_01229	yhcF	Uncharacterized HTH-type transcriptional regulator YhcF
contig_1	cds	107666	107735	+	GLNHAC_01230	yhcG	Uncharacterized ABC transporter ATP-binding protein YhcG
contig_1	cds	107737	107809	+	GLNHAC_01231		ABC transporter domain-containing protein
contig_1	cds	107811	107828	+	GLNHAC_01232		hypothetical protein
contig_1	cds	107828	107921	+	GLNHAC_01233	yhcI	Uncharacterized protein YhcI
contig_1	cds	107930	107951	-	GLNHAC_01234	cspB	cold shock-like protein CspB
contig_1	cds	107994	108077	+	GLNHAC_01235	yhcJ	Uncharacterized lipoprotein YhcJ
contig_1	cds	108078	108186	-	GLNHAC_01236	yhcK	Uncharacterized protein YhcK
contig_1	cds	108203	108342	+	GLNHAC_01237	tcyP	L-cystine uptake protein TcyP
contig_1	cds	108346	108390	-	GLNHAC_01238		Spore coat protein
contig_1	cds	108405	108464	+	GLNHAC_01239	yhcN	Sporulation lipoprotein YhcN
contig_1	cds	108476	108573	+	GLNHAC_01240		Sporulation protein YhcO/YhcP
contig_1	cds	108566	108631	-	GLNHAC_01241	yhcQ	Spore coat protein F-like protein YhcQ
contig_1	cds	108646	108956	+	GLNHAC_01242	yhcR	Endonuclease YhcR
contig_1	cds	108956	109005	+	GLNHAC_01243		hypothetical protein
contig_1	cds	109004	109064	+	GLNHAC_01244	srtD	Sortase D
contig_1	cds	109075	109158	-	GLNHAC_01245		Pseudouridine synthase
contig_1	cds	109169	109208	+	GLNHAC_01246	yhcU	Uncharacterized protein YhcU
contig_1	cds	109222	109264	+	GLNHAC_01247	yhcV	CBS domain-containing protein YhcV
contig_1	cds	109277	109343	+	GLNHAC_01248	yhcW	Putative phosphatase YhcW
contig_1	cds	109345	109494	+	GLNHAC_01249	yhcX	putative hydrolase YhcX
contig_1	cds	109510	109538	+	GLNHAC_01250		hypothetical protein
contig_1	cds	109537	109601	+	GLNHAC_01251		Transporter
contig_1	cds	109617	109751	+	GLNHAC_01252	yhxA	Uncharacterized aminotransferase YhxA
contig_1	cds	109754	109811	+	GLNHAC_01253	glpP	glycerol uptake operon antiterminator GlpP
contig_1	cds	109829	109912	+	GLNHAC_01254	glpF	glycerol uptake facilitator protein GlpF
contig_1	cds	109914	110063	+	GLNHAC_01255	glpK	glycerol kinase GlpK
contig_1	cds	110077	110244	+	GLNHAC_01256	glpD	glycerol-3-phosphate dehydrogenase
contig_1	cds	110257	110394	+	GLNHAC_01257		Phosphoglucomutase
contig_1	cds	110389	110431	+	GLNHAC_01258		phosphoglucomutase (alpha-D-glucose-1,6-bisphosphate-de
contig_1	cds	110446	110561	+	GLNHAC_01259	yhcY	Sensor histidine kinase YhcY
contig_1	cds	110560	110625	+	GLNHAC_01260	yhcZ	Uncharacterized transcriptional regulatory protein YhcZ
contig_1	cds	110625	110677	+	GLNHAC_01261	azr	FMN-dependent NADPH-azoreductase
contig_1	cds	110679	110703	-	GLNHAC_01262	yhdB	Uncharacterized protein YhdB
contig_1	cds	110723	110755	+	GLNHAC_01263	yhdC	Uncharacterized protein YhdC
contig_1	cds	110759	110902	-	GLNHAC_01264		SAG1386/EF1546 family surface-associated protein
contig_1	cds	110917	110961	-	GLNHAC_01265	nsrR	nitric oxide-sensing transcriptional repressor NsrR
contig_1	cds	110973	111125	-	GLNHAC_01266	ygxB	Uncharacterized protein YgxB
contig_1	cds	111142	111282	+	GLNHAC_01267	spoVR	stage V sporulation protein SpoVR
contig_1	cds	111286	111423	-	GLNHAC_01268	phoA	alkaline phosphatase PhoA
contig_1	cds	111477	111578	+	GLNHAC_01269	lytF	Endopeptidase LytF
contig_1	cds	111585	111673	-	GLNHAC_01270	citR	transcriptional regulator CitR
contig_1	cds	111684	111794	+	GLNHAC_01271	citA	citrate synthase CitA
contig_1	cds	111801	111888	+	GLNHAC_01272	yhdF	Uncharacterized oxidoreductase YhdF
contig_1	cds	111913	112053	+	GLNHAC_01273	bcaP	branched-chain amino acid transporter BcaP
contig_1	cds	112067	112200	+	GLNHAC_01274	yhdH	Uncharacterized sodium-dependent transporter YhdH
contig_1	cds	112204	112345	-	GLNHAC_01275	ydeL	YdeL
contig_1	cds	112352	112398	+	GLNHAC_01276	yhdJ	Uncharacterized N-acetyltransferase YhdJ
contig_1	cds	112402	112431	-	GLNHAC_01277	yhdK	putative anti-sigma-M factor YhdK
contig_1	cds	112430	112537	-	GLNHAC_01278	yhdL	putative anti-sigma-M factor YhdL
contig_1	cds	112536	112585	-	GLNHAC_01279	sigM	RNA polymerase sigma factor SigM
contig_1	cds	112605	112620	+	GLNHAC_01280		hypothetical protein
contig_1	cds	112628	112705	+	GLNHAC_01281		General stress protein 69
contig_1	cds	112718	112778	+	GLNHAC_01282	plsC	1-acyl-sn-glycerol-3-phosphate acyltransferase
contig_1	cds	112788	112901	+	GLNHAC_01283		Iminodiacetate oxidase
contig_1	cds	112903	112993	-	GLNHAC_01284		NAD(P)-dependent oxidoreductase
contig_1	cds	113009	113141	-	GLNHAC_01285	yhdP	UPF0053 protein YhdP
contig_1	cds	113148	113201	-	GLNHAC_01286	cueR	HTH-type transcriptional regulator CueR
contig_1	cds	113207	113259	+	GLNHAC_01287		Aminotransferase class I/classII large domain-containing prot
contig_1	cds	113354	113492	+	GLNHAC_01288	yhdT	UPF0053 protein YhdT
contig_1	cds	113494	113529	-	GLNHAC_01289	crcB	fluoride efflux transporter CrcB
contig_1	cds	113529	113569	-	GLNHAC_01290	fluC	Fluoride-specific ion channel FluC
contig_1	cds	113567	113640	-	GLNHAC_01291	yhdW	Putative glycerophosphodiester phosphodiesterase YhdW
contig_1	cds	113664	113675	+	GLNHAC_01292	yhdX	YhdX family protein
contig_1	cds	113690	113801	+	GLNHAC_01293	yhdY	small-conductance mechanosensitive channel protein MscY
contig_1	cds	113809	113889	+	GLNHAC_01294	cobB	NAD-dependent protein deacylase
contig_1	cds	113886	113970	-	GLNHAC_01295	yheN	Putative polysaccharide deacetylase YheN
contig_1	cds	113999	114084	+	GLNHAC_01296	dat	D-amino-acid transaminase
contig_1	cds	114088	114224	-	GLNHAC_01297	nhaC	Na+/H+ antiporter NhaC
contig_1	cds	114237	114287	-	GLNHAC_01298	nhaX	Stress response protein NhaX
contig_1	cds	114332	114507	+	GLNHAC_01299	yheI	multidrug ABC transporter ATP-binding protein BmrC
contig_1	cds	114507	114561	+	GLNHAC_01300		hypothetical protein
contig_1	cds	114562	114709	+	GLNHAC_01301	yheH	Putative multidrug resistance ABC transporter ATP-binding/pe
contig_1	cds	114714	114776	-	GLNHAC_01302		NAD(P)-dependent oxidoreductase
contig_1	cds	114803	114823	-	GLNHAC_01303	sspB	Small, acid-soluble spore protein B
contig_1	cds	114844	114865	-	GLNHAC_01304	yheE	Uncharacterized protein YheE
contig_1	cds	114881	115017	-	GLNHAC_01305	yheD	Endospore coat-associated protein YheD
contig_1	cds	115016	115078	-	GLNHAC_01306	yheC	(pseudo) Endospore coat-associated protein YheC
contig_1	cds	115081	115106	-	GLNHAC_01307		hypothetical protein
contig_1	cds	115152	115171	+	GLNHAC_01308		hypothetical protein
contig_1	cds	115171	115253	+	GLNHAC_01309		(pseudo) DUF445 domain-containing protein
contig_1	cds	115274	115309	+	GLNHAC_01310	yheA	UPF0342 protein YheA
contig_1	cds	115314	115438	-	GLNHAC_01311	yhaZ	Uncharacterized protein YhaZ
contig_1	cds	115470	115556	+	GLNHAC_01312	yhaX	Stress response protein YhaX
contig_1	cds	115567	115718	+	GLNHAC_01313	hemZ	coproporphyrinogen III oxidase
contig_1	cds	115720	115841	-	GLNHAC_01314	khtU	K(+)/H(+) antiporter subunit KhtU
contig_1	cds	115842	115892	-	GLNHAC_01315	khtT	K(+)/H(+) antiporter subunit KhtT
contig_1	cds	115898	115930	-	GLNHAC_01316	khtS	K(+)/H(+) antiporter modulator KhtS
contig_1	cds	115948	116025	+	GLNHAC_01317	yhaR	enoyl-CoA hydratase
contig_1	cds	116027	116046	-	GLNHAC_01318		YhzD-like protein
contig_1	cds	116058	116148	+	GLNHAC_01319	yhaQ	Uncharacterized ABC transporter ATP-binding protein YhaQ
contig_1	cds	116147	116273	+	GLNHAC_01320	yhaP	Uncharacterized protein YhaP
contig_1	cds	116284	116406	+	GLNHAC_01321		DNA repair exonuclease
contig_1	cds	116408	116697	+	GLNHAC_01322	yhaN	Uncharacterized protein YhaN
contig_1	cds	116704	116798	+	GLNHAC_01323	yhaM	3'-5' exoribonuclease YhaM
contig_1	cds	116811	116832	+	GLNHAC_01324	yhaL	sporulation protein YhaL
contig_1	cds	116836	116922	-	GLNHAC_01325	prsA	Foldase protein PrsA
contig_1	sorf	116938	116947	+	GLNHAC_01326	sscA	Small spore coat assembly protein A
contig_1	sorf	116959	116968	+	GLNHAC_01327	sscB	putative small spore coat assembly protein B
contig_1	cds	117002	117054	-	GLNHAC_01328	yhaJ	Uncharacterized membrane protein YhaJ
contig_1	cds	117076	117109	+	GLNHAC_01329	yhaI	Uncharacterized protein YhaI
contig_1	cds	117108	117170	-	GLNHAC_01330	hpr	HTH-type transcriptional regulator Hpr
contig_1	cds	117187	117212	-	GLNHAC_01331	yhaH	(pseudo) Uncharacterized membrane protein YhaH
contig_1	cds	117237	117255	+	GLNHAC_01332	yhzF	Uncharacterized membrane protein YhzF
contig_1	cds	117262	117314	-	GLNHAC_01333	trpP	tryptophan transporter TrpP
contig_1	cds	117327	117435	-	GLNHAC_01334	serC	3-phosphoserine/phosphohydroxythreonine transaminase
contig_1	cds	117449	117493	-	GLNHAC_01335	hit	Protein hit
contig_1	cds	117542	117616	+	GLNHAC_01336	ecsA	ABC transporter ATP-binding protein EcsA
contig_1	cds	117615	117634	+	GLNHAC_01337		ABC transporter permease
contig_1	cds	117634	117738	+	GLNHAC_01338	ecsB	Protein EcsB
contig_1	cds	117740	117811	+	GLNHAC_01339	ecsC	Protein EcsC
contig_1	cds	117813	117932	-	GLNHAC_01340	sndC	N-acetyl amino acid acetylase SndC
contig_1	cds	117939	118077	-	GLNHAC_01341	yhfA	Uncharacterized membrane protein YhfA
contig_1	cds	118114	118164	-	GLNHAC_01342	hmoB	heme-degrading oxygenase HmoB
contig_1	cds	118176	118390	+	GLNHAC_01343	pbpF	Penicillin-binding protein 1F
contig_1	cds	118403	118509	+	GLNHAC_01344	hemE	uroporphyrinogen decarboxylase
contig_1	cds	118516	118609	+	GLNHAC_01345	hemH	ferrochelatase
contig_1	cds	118611	118752	+	GLNHAC_01346	hemY	protoporphyrinogen oxidase
contig_1	cds	118769	118824	+	GLNHAC_01347	yhgD	Uncharacterized HTH-type transcriptional regulator YhgD
contig_1	cds	118831	119064	+	GLNHAC_01348	yhgE	Uncharacterized protein YhgE
contig_1	cds	119074	119165	-	GLNHAC_01349	fabHB	beta-ketoacyl-ACP synthase III FabHB
contig_1	cds	119178	119256	+	GLNHAC_01350	yhfC	Uncharacterized membrane protein YhfC
contig_1	cds	119268	119287	-	GLNHAC_01351		hypothetical protein
contig_1	cds	119299	119403	+	GLNHAC_01352	yhfE	Putative aminopeptidase YhfE
contig_1	cds	119404	119445	+	GLNHAC_01353	yhfF	Uncharacterized protein YhfF
contig_1	cds	119448	119577	-	GLNHAC_01354	gltT	glutamate/proton symporter GltT
contig_1	cds	119634	119707	+	GLNHAC_01355	yhfI	putative metallo-hydrolase YhfI
contig_1	cds	119708	119808	+	GLNHAC_01356	lplJ	lipoate--protein ligase LplJ
contig_1	cds	119814	119879	+	GLNHAC_01357	yhfK	Uncharacterized sugar epimerase YhfK
contig_1	cds	119886	120045	+	GLNHAC_01358	lcfB	long-chain-fatty-acid--CoA ligase LcfB
contig_1	cds	120048	120088	-	GLNHAC_01359	yhfM	Uncharacterized protein YhfM
contig_1	cds	120103	120231	+	GLNHAC_01360	yhfN	Uncharacterized metalloprotease YhfN
contig_1	cds	120235	120320	-	GLNHAC_01361	apr	Subtilisin
contig_1	cds	120331	120349	-	GLNHAC_01362		hypothetical protein
contig_1	cds	120393	120439	+	GLNHAC_01363	yhfO	Uncharacterized N-acetyltransferase YhfO
contig_1	cds	120445	120544	+	GLNHAC_01364	yhfP	Putative quinone oxidoreductase YhfP
contig_1	cds	120577	120674	+	GLNHAC_01365	yhfQ	Putative ABC transporter substrate-binding lipoprotein YhfQ
contig_1	cds	120677	120700	-	GLNHAC_01366		hypothetical protein
contig_1	cds	120700	120735	-	GLNHAC_01367		hypothetical protein

**contig_1**	cds	120757	120851	-	GLNHAC_01368	yhfS	acetyl-CoA C-acyltransferase
**contig_1**	cds	120851	120908	-	GLNHAC_01369		(pseudo) Acyl-CoA synthetase
**contig_1**	cds	120913	120958	-	GLNHAC_01370		hypothetical protein
**contig_1**	cds	120962	120996	-	GLNHAC_01371		hypothetical protein
**contig_1**	cds	120996	121052	-	GLNHAC_01372	bioY	putative biotin transporter BioY
**contig_1**	cds	121066	121204	-	GLNHAC_01373	hemAT	heme-based aerotactic transducer HemAT
**contig_1**	cds	121210	121362	-	GLNHAC_01374	yhfW	Putative Rieske 2Fe-2S iron-sulfur protein YhfW
**contig_1**	cds	121374	121459	+	GLNHAC_01375	yhxC	Uncharacterized oxidoreductase YhxC
**contig_1**	cds	121462	121485	-	GLNHAC_01376	yhzC	Uncharacterized protein YhzC
**contig_1**	cds	121515	121572	+	GLNHAC_01377	comK	competence transcription factor ComK
**contig_1**	cds	121577	121656	-	GLNHAC_01378		SDR family oxidoreductase
**contig_1**	cds	121689	121715	+	GLNHAC_01379	yhjA	Uncharacterized protein YhjA
**contig_1**	cds	121720	121803	-	GLNHAC_01380		Sodium:solute symporter
**contig_1**	cds	121802	121867	-	GLNHAC_01381	yhjB	(pseudo) Uncharacterized symporter YhjB
**contig_1**	cds	121866	121886	-	GLNHAC_01382	yhjC	Uncharacterized membrane protein YhjC
**contig_1**	cds	121909	121945	-	GLNHAC_01383	yhjD	Uncharacterized protein YhjD
**contig_1**	cds	121961	122023	+	GLNHAC_01384	yhjE	Uncharacterized membrane protein YhjE
**contig_1**	cds	122023	122075	+	GLNHAC_01385	lepB	signal peptidase I
**contig_1**	cds	122092	122154	+	GLNHAC_01386		hypothetical protein
**contig_1**	cds	122153	122241	+	GLNHAC_01387		FAD-dependent monooxygenase
**contig_1**	cds	122249	122302	+	GLNHAC_01388	yhjH	Uncharacterized HTH-type transcriptional regulator YhjH
**contig_1**	cds	122304	122324	-	GLNHAC_01389		hypothetical protein
**contig_1**	cds	122391	122513	-	GLNHAC_01390	glcP	glucose/mannose transporter GlcP
**contig_1**	cds	122520	122625	-	GLNHAC_01391	ntdC	glucose-6-phosphate 3-dehydrogenase NdtC
**contig_1**	cds	122627	122711	-	GLNHAC_01392	ntdB	kanosamine-6-phosphate phosphatase
**contig_1**	cds	122709	122753	-	GLNHAC_01393		hypothetical protein
**contig_1**	cds	122756	122841	-	GLNHAC_01394		aminotransferase class I/II-fold pyridoxal phosphate-depende
**contig_1**	cds	122852	122951	+	GLNHAC_01395	ntdR	NTD biosynthesis operon transcriptional regulator NtdR
**contig_1**	cds	123007	123121	-	GLNHAC_01396	yhjN	Uncharacterized protein YhjN
**contig_1**	cds	123131	123252	-	GLNHAC_01397		MFS transporter
**contig_1**	cds	123263	123436	+	GLNHAC_01398	sgrR	SgrR family transcriptional regulator
**contig_1**	cds	123437	123473	+	GLNHAC_01399		hypothetical protein
**contig_1**	cds	123484	123528	-	GLNHAC_01400	yhjR	Spore coat protein YhjR
**contig_1**	cds	123546	123790	+	GLNHAC_01401	addB	helicase-exonuclease AddAB subunit AddB
**contig_1**	cds	123809	123854	+	GLNHAC_01402		PD-(D/E)XK nuclease family protein
**contig_1**	cds	123857	123896	+	GLNHAC_01403		hypothetical protein
**contig_1**	cds	123895	124118	+	GLNHAC_01404		DNA 3'-5' helicase
**contig_1**	cds	124117	124266	+	GLNHAC_01405	addA	(pseudo) helicase-exonuclease AddAB subunit AddA
**contig_1**	cds	124273	124390	+	GLNHAC_01406	sbcD	exonuclease subunit SbcD
**contig_1**	cds	124390	124538	+	GLNHAC_01407		AAA family ATPase
**contig_1**	cds	124540	124633	+	GLNHAC_01408		hypothetical protein
**contig_1**	cds	124635	124729	+	GLNHAC_01409		Nuclease SbcCD subunit C
**contig_1**	cds	124731	124761	+	GLNHAC_01410	hlpB	HNH nuclease-like protein HlpB
**contig_1**	cds	124765	124786	-	GLNHAC_01411	gerPF	putative spore germination protein GerPF
**contig_1**	cds	124790	124830	-	GLNHAC_01412		Spore germination protein PE
**contig_1**	cds	124830	124847	-	GLNHAC_01413	gerPD	spore germination protein GerPD
**contig_1**	cds	124847	124909	-	GLNHAC_01414	gerPC	putative spore germination protein GerPC
**contig_1**	cds	124911	124934	-	GLNHAC_01415		Spore germination protein GerPB
**contig_1**	cds	124936	124958	-	GLNHAC_01416	gerPA	putative spore germination protein GerPA
**contig_1**	cds	124999	125016	-	GLNHAC_01417		Conserved domain protein
**contig_1**	cds	125030	125123	-	GLNHAC_01418		Spore coat protein
**contig_1**	cds	125138	125229	+	GLNHAC_01419	yisK	Oxaloacetate tautomerase YisK
**contig_1**	cds	125240	125276	+	GLNHAC_01420	yisL	UPF0344 protein YisL
**contig_1**	cds	125292	125486	+	GLNHAC_01421		S8 family serine peptidase
**contig_1**	cds	125486	125506	+	GLNHAC_01422		hypothetical protein
**contig_1**	cds	125509	125560	+	GLNHAC_01423		hypothetical protein
**contig_1**	cds	125563	125619	-	GLNHAC_01424	yisN	Uncharacterized protein YisN
**contig_1**	cds	125636	125821	+	GLNHAC_01425	asnB	asparagine synthase (glutamine-hydrolyzing)
**contig_1**	cds	125831	125912	+	GLNHAC_01426		Putative squalene/phytoene synthase
**contig_1**	cds	125915	126052	-	GLNHAC_01427	yisQ	Uncharacterized transporter YisQ
**contig_1**	cds	126064	126151	+	GLNHAC_01428	yisR	Uncharacterized HTH-type transcriptional regulator YisR
**contig_1**	cds	126152	126255	+	GLNHAC_01429	degA	transcriptional regulator DegA
**contig_1**	cds	126275	126377	+	GLNHAC_01430	iolX	scyllo-inositol 2-dehydrogenase (NAD(+))
**contig_1**	cds	126384	126435	-	GLNHAC_01431	dinB	Damage-inducible protein DinB
**contig_1**	cds	126440	126501	-	GLNHAC_01432		Putative aminoacid related metabolite efflux transporter
**contig_1**	cds	126514	126651	+	GLNHAC_01433	yisV	Uncharacterized HTH-type transcriptional regulator YisV
**contig_1**	cds	126685	126723	-	GLNHAC_01434		Pentapeptide repeat-containing protein
**contig_1**	cds	126743	126824	+	GLNHAC_01435	yisY	AB hydrolase superfamily protein YisY
**contig_1**	cds	126826	126886	-	GLNHAC_01436	yisZ	putative adenylyl-sulfate kinase
**contig_1**	cds	126886	127003	-	GLNHAC_01437	yitA	putative sulfate adenylyltransferase
**contig_1**	cds	127014	127086	-	GLNHAC_01438	cysH1	phosphoadenylyl-sulfate reductase
**contig_1**	cds	127104	127173	+	GLNHAC_01439		putative 2-phosphosulfolactate phosphatase
**contig_1**	cds	127173	127183	+	GLNHAC_01440		hypothetical protein
**contig_1**	cds	127186	127248	+	GLNHAC_01441		Phosphosulfolactate synthase
**contig_1**	cds	127253	127320	-	GLNHAC_01442	yitE	UPF0750 membrane protein YitE
**contig_1**	cds	127325	127437	-	GLNHAC_01443		Putative enolase superfamily enzyme
**contig_1**	cds	127438	127565	-	GLNHAC_01444	yitG	Uncharacterized MFS-type transporter YitG
**contig_1**	cds	127576	127662	-	GLNHAC_01445		Acetyltransferase, gnat family
**contig_1**	cds	127662	127707	-	GLNHAC_01446		N-acetyltransferase domain-containing protein
**contig_1**	cds	127716	127801	-	GLNHAC_01447		Methylenetetrahydrofolate reductase
**contig_1**	cds	127807	127897	-	GLNHAC_01448		5,10-methylenetetrahydrofolate reductase
**contig_1**	ncRNA-region	127909	127921	-			SAM riboswitch aptamer (S box leader)
**contig_1**	cds	127932	127981	-	GLNHAC_01449	yitK	YajQ family cyclic di-GMP-binding protein
**contig_1**	cds	127995	128080	+	GLNHAC_01450	yitL	Uncharacterized protein YitL
**contig_1**	cds	128092	128121	+	GLNHAC_01451	yitR	YitR
**contig_1**	cds	128125	128210	-	GLNHAC_01452	yitS	DegV domain-containing protein YitS
**contig_1**	cds	128224	128308	+	GLNHAC_01453	yitT	UPF0750 membrane protein YitT
**contig_1**	cds	128310	128355	+	GLNHAC_01454	ipi	Intracellular proteinase inhibitor
**contig_1**	cds	128358	128378	-	GLNHAC_01455	yizC	Uncharacterized protein YizC
**contig_1**	cds	128378	128393	-	GLNHAC_01456		Competence protein
**contig_1**	cds	128404	128485	-	GLNHAC_01457	yitU	5-amino-6-(5-phospho-D-ribitylamino)uracil phosphatase YitU
**contig_1**	cds	128497	128574	+	GLNHAC_01458	yitV	Putative esterase YitV
**contig_1**	cds	128580	128611	+	GLNHAC_01459	yitW	Fe-S protein maturation auxiliary factor YitW
**contig_1**	cds	128618	128635	+	GLNHAC_01460		YqaE/Pmp3 family membrane protein
**contig_1**	cds	128641	128784	+	GLNHAC_01461	yitY	Uncharacterized FAD-linked oxidoreductase YitY
**contig_1**	cds	128784	128838	+	GLNHAC_01462	yitZ	Uncharacterized MFS-type transporter YitZ
**contig_1**	cds	128858	128962	+	GLNHAC_01463	argC	N-acetyl-gamma-glutamyl-phosphate reductase
**contig_1**	cds	128964	129086	+	GLNHAC_01464	argJ	bifunctional ornithine acetyltransferase/N-acetylglutamate syr
**contig_1**	cds	129087	129165	+	GLNHAC_01465	argB	Acetylglutamate kinase
**contig_1**	cds	129165	129280	+	GLNHAC_01466	argD	acetylornithine transaminase
**contig_1**	cds	129287	129393	+	GLNHAC_01467	carA	carbamoyl phosphate synthase small subunit
**contig_1**	cds	129393	129702	+	GLNHAC_01468	carB	carbamoyl phosphate synthase large subunit
**contig_1**	cds	129701	129797	+	GLNHAC_01469	argF	ornithine carbamoyltransferase
**contig_1**	cds	129805	129823	+	GLNHAC_01470	yjzC	Uncharacterized protein YjzC
**contig_1**	cds	129828	129846	-	GLNHAC_01471	yjzD	Uncharacterized membrane protein YjzD
**contig_1**	cds	129871	129945	+	GLNHAC_01472	yjaU	Uncharacterized protein YjaU
**contig_1**	cds	129953	130009	+	GLNHAC_01473	yjaV	Uncharacterized protein YjaV
**contig_1**	cds	130018	130113	+	GLNHAC_01474	med	transcriptional regulator Med
**contig_1**	cds	130115	130134	+	GLNHAC_01475	comZ	ComG operon transcriptional repressor ComZ
**contig_1**	cds	130137	130159	-	GLNHAC_01476		Spore coat protein
**contig_1**	cds	130176	130270	+	GLNHAC_01477	fabH	beta-ketoacyl-ACP synthase III
**contig_1**	cds	130272	130372	+	GLNHAC_01478		hypothetical protein
**contig_1**	cds	130367	130396	+	GLNHAC_01479		hypothetical protein
**contig_1**	cds	130404	130482	+	GLNHAC_01480	yjaZ	Uncharacterized protein YjaZ
**contig_1**	cds	130501	130600	+	GLNHAC_01481	appD	oligopeptide ABC transporter ATP-binding protein AppD
**contig_1**	cds	130600	130698	+	GLNHAC_01482	appF	oligopeptide ABC transporter ATP-binding protein AppF
**contig_1**	cds	130707	130870	+	GLNHAC_01483		Peptide-binding protein
**contig_1**	cds	130878	130973	+	GLNHAC_01484	gsiC	Glutathione transport system permease protein GsiC
**contig_1**	cds	130974	131066	+	GLNHAC_01485	opp4C	oligopeptide ABC transporter permease
**contig_1**	cds	131086	131161	+	GLNHAC_01486		UPF0736 protein RBAM_011410
**contig_1**	cds	131165	131264	-	GLNHAC_01487	trpS	tryptophan--tRNA ligase
**contig_1**	ncRNA-region	131270	131291	-			T-box leader
**contig_1**	cds	131338	131412	+	GLNHAC_01488		Solute-binding protein family 5 domain-containing protein
**contig_1**	cds	131414	131502	+	GLNHAC_01489	oppA	(pseudo) Oligopeptide-binding protein OppA
**contig_1**	cds	131513	131593	+	GLNHAC_01490	oppB	Oligopeptide transport system permease protein OppB
**contig_1**	cds	131607	131699	+	GLNHAC_01491	oppC	oligopeptide ABC transporter permease OppC
**contig_1**	cds	131700	131807	+	GLNHAC_01492	oppD	oligopeptide ABC transporter ATP-binding protein OppD
**contig_1**	cds	131807	131899	+	GLNHAC_01493	oppF	oligopeptide ABC transporter ATP-binding protein OppF
**contig_1**	cds	131920	132031	+	GLNHAC_01494		Putative exporter
**contig_1**	cds	132048	132106	+	GLNHAC_01495	yjbC	Putative acetyltransferase YjbC
**contig_1**	cds	132124	132164	+	GLNHAC_01496	spx	transcriptional regulator Spx
**contig_1**	cds	132189	132255	-	GLNHAC_01497	yjbE	Uncharacterized membrane protein YjbE
**contig_1**	cds	132282	132348	+	GLNHAC_01498	mecA	adaptor protein MecA
**contig_1**	cds	132364	132479	+	GLNHAC_01499	coiA	Competence protein CoiA
**contig_1**	cds	132484	132507	+	GLNHAC_01500		hypothetical protein
**contig_1**	cds	132524	132685	+	GLNHAC_01501	pepF	oligoendopeptidase F
**contig_1**	cds	132689	132705	-	GLNHAC_01502	yizD	Uncharacterized membrane protein YizD
**contig_1**	ncRNA	132718	132730	+	GLNHAC_01503	rsaE	RNA Staph. aureus E (RoxS)
**contig_1**	cds	132737	132827	-	GLNHAC_01504	spxH	protease adaptor protein SpxH

contig_1	cds	132826	132866	-	GLNHAC_01505	yjbI	Group 2 truncated hemoglobin YjbI
contig_1	cds	132892	132965	-	GLNHAC_01506	cwlQ	Bifunctional muramidase/lytic transglycosylase CwlQ
contig_1	cds	132967	133024	-	GLNHAC_01507	yjbK	Putative triphosphatase YjbK
contig_1	cds	133036	133073	+	GLNHAC_01508	yjbL	UPF0738 protein YjbL
contig_1	cds	133076	133139	+	GLNHAC_01509	yjbM	GTP diphosphokinase
contig_1	cds	133141	133221	+	GLNHAC_01510	ppnKA	NAD kinase
contig_1	cds	133223	133313	+	GLNHAC_01511	yjbO	Uncharacterized RNA pseudouridine synthase YjbO
contig_1	cds	133314	133387	-	GLNHAC_01512	prpE	bis(5'-nucleosyl)-tetraphosphatase PrpE
contig_1	cds	133411	133595	+	GLNHAC_01513	yjbQ	Putative Na(+)/H(+) antiporter YjbQ
contig_1	ncRNA-region	133601	133612	+			TPP riboswitch aptamer (THI element)
contig_1	cds	133620	133691	+	GLNHAC_01514	tenA	thiaminase II
contig_1	cds	133689	133751	+	GLNHAC_01515	tenI	thiazole tautomerase TenI
contig_1	cds	133749	133860	+	GLNHAC_01516	thiO	glycine oxidase ThiO
contig_1	cds	133860	133880	+	GLNHAC_01517	thiS	sulfur carrier protein ThiS
contig_1	cds	133880	133957	+	GLNHAC_01518	thiG	thiazole synthase
contig_1	cds	133956	134057	+	GLNHAC_01519	thiF	Sulfur carrier protein ThiS adenylyltransferase
contig_1	cds	134059	134141	+	GLNHAC_01520	thiD	Hydroxymethylpyrimidine/phosphomethylpyrimidine kinase
contig_1	cds	134154	134232	+	GLNHAC_01521	fabI	enoyl-ACP reductase FabI
contig_1	cds	134243	134310	+	GLNHAC_01522		Spore coat protein O
contig_1	cds	134319	134364	-	GLNHAC_01523	cotZ	Spore coat protein Z
contig_1	cds	134404	134426	-	GLNHAC_01524		(pseudo) Spore coat protein
contig_1	cds	134441	134493	-	GLNHAC_01525	cotX	spore coat protein CotX
contig_1	cds	134507	134535	-	GLNHAC_01526		hypothetical protein
contig_1	cds	134539	134577	-	GLNHAC_01527	cotV	spore coat protein CotV
contig_1	cds	134594	134629	+	GLNHAC_01528	yjcA	sporulation protein YjcA
contig_1	cds	134652	134678	+	GLNHAC_01529		hypothetical protein
contig_1	cds	134686	134702	+	GLNHAC_01530	yjcZ	YjcZ family sporulation protein
contig_1	cds	134715	134737	+	GLNHAC_01531	spoVIF	Sporulation-specific transcription factor SpoVIF
contig_1	cds	134748	134780	-	GLNHAC_01532		hypothetical protein
contig_1	cds	134780	134975	-	GLNHAC_01533	yjcD	Putative ATP-dependent DNA helicase YjcD
contig_1	cds	134987	135013	+	GLNHAC_01534	yjzE	Uncharacterized protein YjzE
contig_1	cds	135028	135062	-	GLNHAC_01535		N-acetyltransferase domain-containing protein
contig_1	cds	135074	135114	-	GLNHAC_01536	yjcG	(pseudo) Putative phosphoesterase YjcG
contig_1	cds	135118	135149	-	GLNHAC_01537	yjcH	(pseudo) Uncharacterized protein YjcH
contig_1	cds	135170	135190	-	GLNHAC_01538		hypothetical protein
contig_1	ncRNA-region	135207	135219	+			SAM riboswitch aptamer (S box leader)
contig_1	cds	135248	135324	+	GLNHAC_01539		Cystathionine gamma-synthase
contig_1	cds	135337	135454	+	GLNHAC_01540	metC	cystathionine beta-lyase
contig_1	cds	135457	135512	-	GLNHAC_01541		GNAT family N-acetyltransferase
contig_1	cds	135519	135636	+	GLNHAC_01542	yjcL	Uncharacterized membrane protein YjcL
contig_1	tRNA	135655	135662	+	GLNHAC_01543	trnV	tRNA-Val(gac)
contig_1	cds	135679	135691	-	GLNHAC_01544		Phr peptide
contig_1	cds	135690	135806	-	GLNHAC_01545		Rap phosphatase
contig_1	cds	135835	135863	+	GLNHAC_01546	yjcS	Uncharacterized protein YjcS
contig_1	cds	135914	136046	+	GLNHAC_01547	manR	(pseudo) Transcriptional regulator ManR
contig_1	cds	136046	136100	+	GLNHAC_01548		PTS sugar transporter subunit IIA
contig_1	cds	136124	136319	+	GLNHAC_01549	manP	PTS mannose transporter subunit IIABC
contig_1	cds	136321	136415	+	GLNHAC_01550	manA	mannose-6-phosphate isomerase, class I
contig_1	cds	136463	136491	+	GLNHAC_01551		Lipoprotein
contig_1	cds	136500	136523	-	GLNHAC_01552		(pseudo) GNAT family N-acetyltransferase
contig_1	cds	136533	136550	-	GLNHAC_01553		hypothetical protein
contig_1	cds	136577	136617	-	GLNHAC_01554	yjdH	Uncharacterized protein YjdH
contig_1	cds	136637	136685	+	GLNHAC_01555	ybaK	Cys-tRNA(Pro) deacylase
contig_1	cds	136689	136709	-	GLNHAC_01556	yjzH	Uncharacterized protein YjzH
contig_1	cds	136788	136807	+	GLNHAC_01557		hypothetical protein
contig_1	cds	136810	136906	-	GLNHAC_01558	cyoE	heme o synthase
contig_1	cds	136921	136944	-	GLNHAC_01559		Spore coat protein
contig_1	cds	136969	137060	+	GLNHAC_01560	pdaC	(pseudo) Peptidoglycan-N-acetylmuramic acid deacetylase P
contig_1	cds	137063	137080	+	GLNHAC_01561		hypothetical protein
contig_1	cds	137085	137109	+	GLNHAC_01562		hypothetical protein
contig_1	cds	137113	137161	-	GLNHAC_01563	yjfA	Uncharacterized protein YjfA
contig_1	cds	137174	137190	-	GLNHAC_01564	yjfB	Uncharacterized protein YjfB
contig_1	cds	137203	137293	+	GLNHAC_01565		DUF2268 domain-containing protein
contig_1	cds	137294	137337	-	GLNHAC_01566	yjgA	YjgA
contig_1	cds	137344	137401	-	GLNHAC_01567	yjgB	YjgB family protein
contig_1	cds	137416	137712	+	GLNHAC_01568	fdhF	formate dehydrogenase subunit alpha
contig_1	cds	137711	137767	+	GLNHAC_01569	yjgD	Uncharacterized protein YjgD
contig_1	cds	137796	137855	+	GLNHAC_01570		DUF4352 domain-containing protein
contig_1	cds	137865	137926	+	GLNHAC_01571		Nudix hydrolase
contig_1	cds	137930	137957	-	GLNHAC_01572	yjiA	Uncharacterized protein YjiA
contig_1	cds	137997	138041	+	GLNHAC_01573		hypothetical protein
contig_1	cds	138053	138171	+	GLNHAC_01574		UDP-glucosyltransferase
contig_1	cds	138175	138194	-	GLNHAC_01575	yjzI	Uncharacterized protein YjzI
contig_1	cds	138211	138293	+	GLNHAC_01576	yjjA	uroporphyrinogen-III synthase
contig_1	cds	138297	138372	-	GLNHAC_01577	yjkA	UPF0014 membrane protein YjkA
contig_1	cds	138372	138448	-	GLNHAC_01578	yjkB	Putative ABC transporter ATP-binding protein YjkB
contig_1	cds	138460	138557	-	GLNHAC_01579	yjlA	Uncharacterized protein YjlA
contig_1	cds	138570	138620	+	GLNHAC_01580		Cupin type-1 domain-containing protein
contig_1	cds	138659	138701	+	GLNHAC_01581	forE	DUF1641 domain-containing protein
contig_1	cds	138705	138773	+	GLNHAC_01582		NAD(P)/FAD-dependent oxidoreductase
contig_1	cds	138776	138823	+	GLNHAC_01583		(pseudo) NAD(P)/FAD-dependent oxidoreductase
contig_1	cds	138841	138877	+	GLNHAC_01584		(pseudo) Uronate isomerase
contig_1	cds	138930	138949	+	GLNHAC_01585		hypothetical protein
contig_1	cds	138950	138985	+	GLNHAC_01586		Uronate isomerase
contig_1	cds	138993	139008	+	GLNHAC_01587		hypothetical protein
contig_1	cds	139018	139131	+	GLNHAC_01588	yjmB	Uncharacterized symporter YjmB
contig_1	cds	139142	139166	+	GLNHAC_01589		hypothetical protein
contig_1	cds	139165	139244	+	GLNHAC_01590		Ldh family oxidoreductase
contig_1	cds	139244	139346	+	GLNHAC_01591	yjmD	Uncharacterized zinc-type alcohol dehydrogenase-like protein
contig_1	cds	139349	139367	+	GLNHAC_01592		hypothetical protein
contig_1	cds	139368	139397	+	GLNHAC_01593	uxuA	(pseudo) mannonate dehydratase
contig_1	cds	139396	139456	+	GLNHAC_01594		Mannonate dehydratase
contig_1	cds	139456	139489	+	GLNHAC_01595		SDR family NAD(P)-dependent oxidoreductase
contig_1	cds	139492	139518	+	GLNHAC_01596	yjmF	(pseudo) YjmF protein
contig_1	cds	139526	139539	+	GLNHAC_01597		hypothetical protein
contig_1	cds	139544	139581	+	GLNHAC_01598		hypothetical protein
contig_1	cds	139576	139671	+	GLNHAC_01599	exuT	(pseudo) Hexuronate transporter
contig_1	cds	139680	139780	+	GLNHAC_01600	exuR	putative HTH-type transcriptional repressor ExuR
contig_1	cds	139787	139932	+	GLNHAC_01601	uxaB	Altronate oxidoreductase
contig_1	cds	139931	140081	+	GLNHAC_01602	uxaA	Altronate dehydratase
contig_1	cds	140084	140161	-	GLNHAC_01603	yjnA	putative membrane transporter protein YjnA
contig_1	cds	140183	140230	-	GLNHAC_01604	yjoA	Uncharacterized protein YjoA
contig_1	cds	140244	140372	+	GLNHAC_01605	yjoB	ATPase YjoB
contig_1	cds	140387	140500	+	GLNHAC_01606	rapA	response regulator aspartate phosphatase RapA
contig_1	cds	140499	140512	+	GLNHAC_01607	phrA	phosphatase RapA inhibitor PhrA
contig_1	cds	140515	140541	-	GLNHAC_01608	yjpA	Uncharacterized protein YjpA
contig_1	cds	140553	140648	+	GLNHAC_01609	xlyB	N-acetylmuramoyl-L-alanine amidase XlyB
contig_1	cds	140652	140690	-	GLNHAC_01610	yjqA	Uncharacterized protein YjqA
contig_1	cds	140701	140761	+	GLNHAC_01611	yjqB	UPF0714 protein YjqB
contig_1	cds	140768	140852	+	GLNHAC_01612	yjqC	Uncharacterized protein YjqC
contig_1	cds	140856	140916	-	GLNHAC_01613	xkdA	Phage-like element PBSX protein XkdA
contig_1	cds	140932	140966	-	GLNHAC_01614	xre	HTH-type transcriptional regulator Xre
contig_1	cds	140984	141002	+	GLNHAC_01615	yjzJ	Uncharacterized protein YjzJ
contig_1	cds	141001	141084	+	GLNHAC_01616		Phage portal protein
contig_1	cds	141091	141154	+	GLNHAC_01617	dnaC	DNA replication protein DnaC
contig_1	cds	141154	141171	+	GLNHAC_01618	ykzK	YkzK
contig_1	cds	141170	141180	+	GLNHAC_01619		hypothetical protein
contig_1	cds	141180	141215	+	GLNHAC_01620	xkdD	phage-like element PBSX protein XkdD
contig_1	cds	141214	141235	+	GLNHAC_01621	xtrA	phage-like element PBSX protein XtrA
contig_1	cds	141246	141297	+	GLNHAC_01622	xpf	Positive control factor
contig_1	cds	141309	141389	+	GLNHAC_01623	xtmA	phage terminase small subunit
contig_1	cds	141388	141518	+	GLNHAC_01624	xtmB	PBSX family phage terminase large subunit
contig_1	cds	141519	141668	+	GLNHAC_01625	xkdE	phage portal protein
contig_1	cds	141670	141752	+	GLNHAC_01626	xkdF	Phage-like element PBSX protein XkdF
contig_1	cds	141755	141848	+	GLNHAC_01627	xkdG	Putative prophage capsid protein XkdG
contig_1	cds	141850	141889	+	GLNHAC_01628	ykzL	Uncharacterized protein YkzL
contig_1	cds	141888	141924	+	GLNHAC_01629	xkdH	Phage-like element PBSX protein XkdH
contig_1	cds	141924	141972	+	GLNHAC_01630	xkdI	Phage-like element PBSX protein XkdI
contig_1	cds	141974	142018	+	GLNHAC_01631	xkdJ	Phage-like element PBSX protein XkdJ
contig_1	cds	142039	142179	+	GLNHAC_01632	xkdK	Phage-like element PBSX protein XkdK
contig_1	cds	142180	142224	+	GLNHAC_01633	xkdM	Phage-like element PBSX protein XkdM
contig_1	cds	142233	142255	+	GLNHAC_01634		(pseudo) Phage portal protein
contig_1	cds	142297	142728	+	GLNHAC_01635		Transglycosylase SLT domain-containing protein
contig_1	cds	142762	142800	+	GLNHAC_01636		(pseudo) transglycosylase SLT domain-containing protein
contig_1	cds	142812	142833	+	GLNHAC_01637		hypothetical protein
contig_1	cds	142838	142878	+	GLNHAC_01638	lysM	LysM peptidoglycan-binding domain-containing protein
contig_1	cds	142880	142977	+	GLNHAC_01639	xkdQ	Phage-like element PBSX protein XkdQ
contig_1	cds	142977	143003	+	GLNHAC_01640		hypothetical protein
contig_1	cds	143010	143052	+	GLNHAC_01641	xkdS	Phage-like element PBSX protein XkdS

**contig_1**	cds	143051	143156	+	GLNHAC_01642	xkdT	Phage-like element PBSX protein XkdT
**contig_1**	cds	143154	143212	+	GLNHAC_01643	xkdU	Phage-like element PBSX protein XkdU
**contig_1**	cds	143212	143239	+	GLNHAC_01644		hypothetical protein
**contig_1**	cds	143239	143424	+	GLNHAC_01645		(pseudo) Terminase
**contig_1**	cds	143418	143477	+	GLNHAC_01646		hypothetical protein
**contig_1**	cds	143485	143518	+	GLNHAC_01647	yorD	Bacteriophage SP-beta YorD domain-containing protein
**contig_1**	cds	143518	143534	+	GLNHAC_01648	xkdX	XkdX family protein
**contig_1**	cds	143538	143622	+	GLNHAC_01649	xepA	Phage-like element PBSX protein XepA
**contig_1**	cds	143628	143655	+	GLNHAC_01650	xhlA	Hemolysin XhlA family protein
**contig_1**	cds	143656	143682	+	GLNHAC_01651	xhlB	phage holin
**contig_1**	cds	143683	143773	+	GLNHAC_01652	xlyA	N-acetylmuramoyl-L-alanine amidase XlyA
**contig_1**	cds	143799	143816	-	GLNHAC_01653	spoIISB	three component toxin-antitoxin-antitoxin system antitoxin Sp
**contig_1**	cds	143816	143890	-	GLNHAC_01654	spoIISA	toxin-antitoxin-antitoxin system toxin SpoIISA
**contig_1**	cds	143901	144001	-	GLNHAC_01655	pit	putative low-affinity inorganic phosphate transporter
**contig_1**	cds	144003	144064	-	GLNHAC_01656	ykaA	UPF0111 protein YkaA
**contig_1**	cds	144092	144224	-	GLNHAC_01657	steT	serine/threonine exchanger
**contig_1**	cds	144262	144357	+	GLNHAC_01658	mhqA	Putative ring-cleaving dioxygenase MhqA
**contig_1**	cds	144367	144382	+	GLNHAC_01659		hypothetical protein
**contig_1**	cds	144383	144476	+	GLNHAC_01660		Mannosyltransferase
**contig_1**	cds	144476	144601	+	GLNHAC_01661	ykcB	(pseudo) YkcB
**contig_1**	cds	144602	144700	+	GLNHAC_01662	ykcC	Uncharacterized glycosyltransferase YkcC
**contig_1**	cds	144703	144746	+	GLNHAC_01663		hypothetical protein
**contig_1**	cds	144750	144887	-	GLNHAC_01664	htrA	serine protease HtrA
**contig_1**	cds	144903	144985	+	GLNHAC_01665	proG	pyrroline-5-carboxylate reductase ProG
**contig_1**	cds	145018	145100	+	GLNHAC_01666	dppA	D-aminopeptidase DppA
**contig_1**	cds	145103	145194	+	GLNHAC_01667	dppB	dipeptide ABC transporter permease DppB
**contig_1**	cds	145194	145291	+	GLNHAC_01668	dppC	dipeptide ABC transporter permease DppC
**contig_1**	cds	145292	145392	+	GLNHAC_01669	dppD	dipeptide ABC transporter ATP-binding subunit DppD
**contig_1**	cds	145394	145439	+	GLNHAC_01670		hypothetical protein
**contig_1**	cds	145434	145558	+	GLNHAC_01671	dppE	dipeptide ABC transporter substrate-binding protein DppE
**contig_1**	cds	145568	145662	+	GLNHAC_01672	ykfA	putative murein peptide carboxypeptidase
**contig_1**	cds	145662	145772	+	GLNHAC_01673	ykfB	L-Ala-D/L-Glu epimerase
**contig_1**	cds	145772	145861	+	GLNHAC_01674		C40 family peptidase
**contig_1**	cds	145862	145885	+	GLNHAC_01675		hypothetical protein
**contig_1**	cds	145889	145961	+	GLNHAC_01676	ykfD	(pseudo) Putative oligopeptide transport ATP-binding protein
**contig_1**	cds	145965	146069	-	GLNHAC_01677	pgl	6-phosphogluconolactonase
**contig_1**	cds	146078	146164	-	GLNHAC_01678	ykgA	Uncharacterized protein YkgA
**contig_1**	cds	146179	146235	+	GLNHAC_01679	ykhA	Uncharacterized acyl-CoA thioester hydrolase YkhA
**contig_1**	cds	146259	146379	+	GLNHAC_01680	hmpA	NO-inducible flavohemoprotein
**contig_1**	cds	146387	146410	-	GLNHAC_01681	ykzH	YkzH
**contig_1**	cds	146425	146499	+	GLNHAC_01682	ykjA	UPF0702 transmembrane protein YkjA
**contig_1**	cds	146508	146561	+	GLNHAC_01683	ykkA	Uncharacterized protein YkkA
**contig_1**	cds	146560	146611	+	GLNHAC_01684		N-acetyltransferase domain-containing protein
**contig_1**	ncRNA-region	146614	146625	+			Guanidine-I riboswitch aptamer
**contig_1**	cds	146633	146667	+	GLNHAC_01685	ykkC	multidrug efflux SMR transporter subunit YkkC
**contig_1**	cds	146667	146699	+	GLNHAC_01686	ykkD	multidrug efflux SMR transporter subunit YkkD
**contig_1**	cds	146706	146796	+	GLNHAC_01687	purU	formyltetrahydrofolate deformylase
**contig_1**	cds	146832	146942	+	GLNHAC_01688	proB	Glutamate 5-kinase 1
**contig_1**	cds	146943	147001	+	GLNHAC_01689		aldehyde dehydrogenase family protein
**contig_1**	cds	147005	147068	+	GLNHAC_01690		glutamate-5-semialdehyde dehydrogenase
**contig_1**	cds	147081	147123	+	GLNHAC_01691	ohrA	Organic hydroperoxide resistance protein OhrA
**contig_1**	cds	147127	147171	-	GLNHAC_01692	ohrR	organic hydroperoxide resistance transcriptional regulator Oh
**contig_1**	cds	147185	147226	+	GLNHAC_01693	ohrB	Organic hydroperoxide resistance protein OhrB
**contig_1**	cds	147246	147349	+	GLNHAC_01694		Glycosyl transferase
**contig_1**	cds	147350	147397	-	GLNHAC_01695		nucleoside deaminase
**contig_1**	cds	147410	147473	+	GLNHAC_01696		Phosphoglycerate mutase family protein
**contig_1**	cds	147477	147706	-	GLNHAC_01697	metE	5-methyltetrahydropteroyltriglutamate--homocysteine S-meth
**contig_1**	ncRNA-region	147718	147734	-			SAM riboswitch aptamer (S box leader)
**contig_1**	cds	147747	147843	-	GLNHAC_01698	isp	serine protease Isp
**contig_1**	cds	147865	147949	+	GLNHAC_01699	rsbRB	RsbT co-antagonist RsbRB
**contig_1**	cds	147952	148028	-	GLNHAC_01700	thiX	HMP/thiamine ABC transporter permease ThiX
**contig_1**	cds	148026	148143	-	GLNHAC_01701	ykoD	(pseudo) energy-coupling factor transporter ATPase
**contig_1**	cds	148155	148190	-	GLNHAC_01702		hypothetical protein
**contig_1**	cds	148188	148248	-	GLNHAC_01703	ykoE	Putative HMP/thiamine permease protein YkoE
**contig_1**	cds	148249	148309	-	GLNHAC_01704	ykoF	HMP/thiamine ABC transporter substrate-binding protein ThiU
**contig_1**	ncRNA-region	148318	148329	-			TPP riboswitch aptamer (THI element)
**contig_1**	cds	148340	148408	+	GLNHAC_01705	ykoG	two-component system response regulator YkoG
**contig_1**	cds	148409	148490	+	GLNHAC_01706	ykoH	(pseudo) Sensor histidine kinase YkoH
**contig_1**	cds	148486	148545	+	GLNHAC_01707		(pseudo) histidine kinase
**contig_1**	cds	148545	148613	+	GLNHAC_01708		PepSY domain-containing protein
**contig_1**	cds	148622	148673	+	GLNHAC_01709		PepSY domain-containing protein
**contig_1**	cds	148681	148695	+	GLNHAC_01710	ykzD	Uncharacterized protein YkzD
**contig_1**	ncRNA-region	148706	148723	+			M-box riboswitch aptamer (ykoK leader)
**contig_1**	cds	148732	148744	+	GLNHAC_01711		hypothetical protein
**contig_1**	cds	148746	148881	+	GLNHAC_01712	mgtE	magnesium transporter
**contig_1**	cds	148886	148919	-	GLNHAC_01713	tnrA	MerR family transcriptional regulator TnrA
**contig_1**	cds	148938	148954	+	GLNHAC_01714		hypothetical protein
**contig_1**	cds	148963	148981	+	GLNHAC_01715	ykoL	stress response protein YkoL
**contig_1**	cds	148994	149041	+	GLNHAC_01716	ykoM	Uncharacterized HTH-type transcriptional regulator YkoM
**contig_1**	cds	149045	149154	-	GLNHAC_01717		Diacylglycerol glucosyltransferase N-terminal domain-contain
**contig_1**	cds	149163	149222	+	GLNHAC_01718	ykoP	Uncharacterized protein YkoP
**contig_1**	cds	149221	149302	-	GLNHAC_01719	ykoQ	Uncharacterized metallophosphoesterase YkoQ
**contig_1**	cds	149322	149384	+	GLNHAC_01720		Dolichyl-phosphate-mannose-mannosyltransferase family pro
**contig_1**	cds	149389	149491	+	GLNHAC_01721	ykoS	(pseudo) Uncharacterized membrane protein YkoS
**contig_1**	cds	149493	149594	+	GLNHAC_01722	ykoT	Uncharacterized glycosyltransferase YkoT
**contig_1**	cds	149607	149791	-	GLNHAC_01723		DNA ligase D
**contig_1**	cds	149788	149829	-	GLNHAC_01724		hypothetical protein
**contig_1**	cds	149824	149858	-	GLNHAC_01725		Ku domain-containing protein
**contig_1**	cds	149888	150128	-	GLNHAC_01726	ykoW	Signaling protein YkoW
**contig_1**	cds	150149	150212	+	GLNHAC_01727		VTT domain-containing protein
**contig_1**	cds	150220	150318	+	GLNHAC_01728	ykoY	Uncharacterized membrane protein YkoY
**contig_1**	cds	150344	150420	+	GLNHAC_01729	sigI	RNA polymerase sigma factor SigI
**contig_1**	cds	150420	150534	+	GLNHAC_01730	rsgI	Anti-sigma-I factor RsgI
**contig_1**	cds	150535	150555	-	GLNHAC_01731	sspD	Small, acid-soluble spore protein D
**contig_1**	cds	150568	150638	-	GLNHAC_01732	htpK	transcriptional regulator HtpK
**contig_1**	cds	150655	150745	+	GLNHAC_01733	htpX	protease HtpX
**contig_1**	cds	150762	150897	+	GLNHAC_01734	ktrD	Ktr system potassium uptake protein D
**contig_1**	cds	150913	150928	+	GLNHAC_01735	ykzP	Uncharacterized protein YkzP
**contig_1**	cds	150929	150946	+	GLNHAC_01736	ykzE	YkzE
**contig_1**	cds	150950	151052	-	GLNHAC_01737	ykrP	Putative membrane-bound acyltransferase YkrP
**contig_1**	cds	151077	151280	+	GLNHAC_01738	kinE	Sporulation kinase E
**contig_1**	cds	151278	151299	+	GLNHAC_01739		ATP-binding protein
**contig_1**	cds	151299	151348	+	GLNHAC_01740	ogt	Methylated-DNA--protein-cysteine methyltransferase, constitu
**contig_1**	cds	151357	151370	+	GLNHAC_01741		hypothetical protein
**contig_1**	cds	151373	151407	-	GLNHAC_01742		hypothetical protein
**contig_1**	cds	151405	151479	-	GLNHAC_01743		Methylthioribose-1-phosphate isomerase 2
**contig_1**	cds	151480	151600	-	GLNHAC_01744	mtnK	S-methyl-5-thioribose kinase
**contig_1**	ncRNA-region	151611	151626	-			SAM riboswitch aptamer (S box leader)
**contig_1**	cds	151634	151712	-	GLNHAC_01745	mtnU	2-oxoglutaramate amidase
**contig_1**	cds	151722	151841	+	GLNHAC_01746	mtnE	methionine-glutamine aminotransferase
**contig_1**	ncRNA-region	151845	151855	+			SAM riboswitch aptamer (S box leader)
**contig_1**	cds	151864	151975	+	GLNHAC_01747	mtnW	2,3-diketo-5-methylthiopentyl-1-phosphate enolase
**contig_1**	cds	151985	152056	+	GLNHAC_01748	mtnX	2-hydroxy-3-keto-5-methylthiopentenyl-1-phosphate phosph
**contig_1**	cds	152052	152114	+	GLNHAC_01749	mtnB	methylthioribulose 1-phosphate dehydratase
**contig_1**	cds	152116	152170	+	GLNHAC_01750	mtnD	acireductone dioxygenase
**contig_1**	cds	152174	152206	-	GLNHAC_01751	ykvA	Uncharacterized membrane protein YkvA
**contig_1**	cds	152226	152252	+	GLNHAC_01752	spo0E	aspartyl-phosphate phosphatase Spo0E
**contig_1**	cds	152260	152303	+	GLNHAC_01753		Uncharacterized protein eag
**contig_1**	cds	152306	152456	-	GLNHAC_01754	kinD	sporulation kinase KinD
**contig_1**	cds	152477	152521	+	GLNHAC_01755	mhqR	MarR family transcriptional regulator MhqR
**contig_1**	cds	152525	152603	-	GLNHAC_01756	motB	flagellar motor protein MotB
**contig_1**	cds	152601	152683	-	GLNHAC_01757	motA	flagellar motor stator protein MotA
**contig_1**	cds	152721	152930	-	GLNHAC_01758	clpE	ATP-dependent protease ATP-binding subunit ClpE
**contig_1**	cds	152967	153071	+	GLNHAC_01759	ykvI	Uncharacterized membrane protein YkvI
**contig_1**	ncRNA-region	153086	153090	+			PreQ1 (pre queuosine) riboswitch aptamer
**contig_1**	cds	153103	153169	+	GLNHAC_01760	queC	7-cyano-7-deazaguanine synthase QueC
**contig_1**	cds	153169	153213	+	GLNHAC_01761	queD	6-carboxytetrahydropterin synthase QueD
**contig_1**	cds	153212	153285	+	GLNHAC_01762	queE	7-carboxy-7-deazaguanine synthase QueE
**contig_1**	cds	153287	153337	+	GLNHAC_01763	queF	preQ(1) synthase
**contig_1**	cds	153384	153496	+	GLNHAC_01764	ykvP	spore protein YkvP
**contig_1**	cds	153497	153519	+	GLNHAC_01765	ykzQ	Uncharacterized protein YkzQ
**contig_1**	cds	153528	153621	+	GLNHAC_01766		Glycosyl hydrolase, family 18
**contig_1**	cds	153676	153705	+	GLNHAC_01767		DUF3219 domain-containing protein
**contig_1**	cds	153717	153735	-	GLNHAC_01768	ykvS	Uncharacterized protein YkvS
**contig_1**	cds	153752	153771	+	GLNHAC_01769	ykzS	Uncharacterized protein YkzS
**contig_1**	cds	153801	153864	+	GLNHAC_01770		cell wall hydrolase
**contig_1**	cds	153876	154010	+	GLNHAC_01771	ykvU	sporulation protein YkvU
**contig_1**	cds	154015	154064	+	GLNHAC_01772	stoA	endospore biogenesis thiol-disulfide oxidoreductase StoA
**contig_1**	cds	154088	154104	+	GLNHAC_01773		hypothetical protein

contig_1	cds	154098	154148	+	GLNHAC_01774		hypothetical protein
contig_1	cds	154150	154279	+	GLNHAC_01775		heavy metal translocating P-type ATPase
contig_1	cds	154283	154303	-	GLNHAC_01776		MFS transporter
contig_1	cds	154320	154429	+	GLNHAC_01777	ykvY	di/tri-peptidase
contig_1	cds	154434	154447	-	GLNHAC_01778		Lipoprotein
contig_1	cds	154457	154554	+	GLNHAC_01779	ykvZ	Uncharacterized HTH-type transcriptional regulator YkvZ
contig_1	cds	154562	154613	+	GLNHAC_01780		PtsGHI operon antiterminator
contig_1	cds	154634	154646	-	GLNHAC_01781		hypothetical protein
contig_1	cds	154669	154879	+	GLNHAC_01782	ptsG	PTS glucose transporter subunit IIABC
contig_1	cds	154889	154916	+	GLNHAC_01783	ptsH	phosphocarrier protein HPr
contig_1	cds	154916	154980	+	GLNHAC_01784		phosphoenolpyruvate-utilizing N-terminal domain-containing
contig_1	cds	154980	155052	+	GLNHAC_01785		phosphoenolpyruvate--protein phosphotransferase
contig_1	cds	155052	155087	+	GLNHAC_01786		putative PEP-binding protein
contig_1	cds	155096	155120	+	GLNHAC_01787	splA	splAB operon transcriptional regulator SplA
contig_1	cds	155127	155230	+	GLNHAC_01788	splB	spore photoproduct lyase
contig_1	cds	155232	155300	-	GLNHAC_01789		Acetyltransferase, gnat family
contig_1	cds	155313	155510	+	GLNHAC_01790	mcpC	Methyl-accepting chemotaxis protein McpC
contig_1	cds	155525	155612	+	GLNHAC_01791	ykwC	Uncharacterized oxidoreductase YkwC
contig_1	cds	155616	155696	-	GLNHAC_01792		CAP domain-containing protein
contig_1	cds	155730	155942	+	GLNHAC_01793	pbpH	Penicillin-binding protein H
contig_1	cds	155958	156140	+	GLNHAC_01794	kinA	sporulation kinase KinA
contig_1	cds	156141	156260	-	GLNHAC_01795	dapX	aminotransferase A
contig_1	cds	156280	156296	-	GLNHAC_01796	ykzT	Uncharacterized protein YkzT
contig_1	cds	156316	156408	+	GLNHAC_01797	cheV	chemotaxis protein CheV
contig_1	cds	156412	156458	-	GLNHAC_01798	ykyB	Uncharacterized protein YkyB
contig_1	cds	156471	156527	-	GLNHAC_01799		MFS transporter
contig_1	cds	156545	156600	-	GLNHAC_01800		hypothetical protein
contig_1	cds	156608	156658	-	GLNHAC_01801	ykuD	Putative L,D-transpeptidase YkuD
contig_1	cds	156663	156749	-	GLNHAC_01802	ykuE	Uncharacterized metallophosphoesterase YkuE
contig_1	cds	156763	156832	+	GLNHAC_01803		Peroxisomal trans-2-enoyl-CoA reductase
contig_1	cds	156846	156925	+	GLNHAC_01804	ykuI	(pseudo) Uncharacterized EAL-domain containing protein Yku
contig_1	cds	156924	156968	+	GLNHAC_01805		EAL-associated domain-containing protein
contig_1	ncRNA	156976	156985	+	GLNHAC_01806	fsrA	FsrA
contig_1	cds	157030	157054	+	GLNHAC_01807	ykuJ	Uncharacterized protein YkuJ
contig_1	cds	157065	157116	+	GLNHAC_01808	ykuK	Uncharacterized protein YkuK
contig_1	cds	157130	157150	+	GLNHAC_01809	abbA	AbrB antirepressor AbbA
contig_1	cds	157163	157208	+	GLNHAC_01810	cbpB	cyclic-di-AMP-binding protein CbpB
contig_1	cds	157222	157311	+	GLNHAC_01811	ykuM	transcriptional regulator CcpC
contig_1	cds	157322	157369	+	GLNHAC_01812	ykuN	flavodoxin
contig_1	cds	157368	157458	+	GLNHAC_01813	ykuO	Uncharacterized protein YkuO
contig_1	cds	157459	157505	+	GLNHAC_01814	ykuP	flavodoxin
contig_1	cds	157515	157586	+	GLNHAC_01815	dapD	2,3,4,5-tetrahydropyridine-2,6-dicarboxylate N-acetyltransfera
contig_1	cds	157587	157663	+	GLNHAC_01816		N-acetyl-L,L-diaminopimelate deacetylase
contig_1	cds	157666	157698	+	GLNHAC_01817		hypothetical protein
contig_1	cds	157712	157736	+	GLNHAC_01818	ykuS	UPF0180 protein YkuS
contig_1	cds	157740	157820	-	GLNHAC_01819	ykuT	small-conductance mechanosensitive channel protein MscT
contig_1	cds	157826	157846	+	GLNHAC_01820		hypothetical protein
contig_1	cds	157844	157899	+	GLNHAC_01821	ykuU	biofilm-specific peroxidase AhpA
contig_1	cds	157913	157951	+	GLNHAC_01822	ykuV	Thiol-disulfide oxidoreductase YkuV
contig_1	cds	157996	158055	+	GLNHAC_01823	rok	transcriptional regulator Rok
contig_1	cds	158059	158155	-	GLNHAC_01824	cse15	Sporulation protein cse15
contig_1	cds	158169	158229	+	GLNHAC_01825		putative molybdenum cofactor guanylyltransferase
contig_1	cds	158234	158336	+	GLNHAC_01826	moeB	Molybdopterin-synthase adenylyltransferase
contig_1	cds	158338	158467	+	GLNHAC_01827	glp	gephyrin-like molybdotransferase Glp
contig_1	cds	158463	158506	+	GLNHAC_01828	mobB	molybdopterin-guanine dinucleotide biosynthesis protein B
contig_1	cds	158506	158516	+	GLNHAC_01829		hypothetical protein
contig_1	cds	158515	158562	+	GLNHAC_01830	moaE	Molybdopterin synthase catalytic subunit
contig_1	cds	158562	158585	+	GLNHAC_01831	moaD	molybdopterin converting factor subunit 1
contig_1	cds	158607	158783	+	GLNHAC_01832	yknU	Uncharacterized ABC transporter ATP-binding protein YknU
contig_1	cds	158784	158965	+	GLNHAC_01833	yknV	Uncharacterized ABC transporter ATP-binding protein YknV
contig_1	cds	158977	159046	+	GLNHAC_01834	yknW	toxin SDP protection protein YknW
contig_1	cds	159047	159076	+	GLNHAC_01835		efflux RND transporter periplasmic adaptor subunit
contig_1	cds	159074	159160	+	GLNHAC_01836	yknX	(pseudo) Putative efflux system component YknX
contig_1	cds	159160	159193	+	GLNHAC_01837	yknY	(pseudo) Uncharacterized ABC transporter ATP-binding prote
contig_1	cds	159192	159230	+	GLNHAC_01838		ABC transporter, ATP-binding domain protein
contig_1	cds	159229	159349	+	GLNHAC_01839	yknZ	sporulation-delaying-protein transporter subunit SkiZ
contig_1	cds	159377	159452	+	GLNHAC_01840	fruR	Uncharacterized HTH-type transcriptional regulator FruR
contig_1	cds	159452	159543	+	GLNHAC_01841	pfkB	1-phosphofructokinase
contig_1	cds	159544	159663	+	GLNHAC_01842		PTS fructose transporter subunit IIC
contig_1	cds	159667	159734	+	GLNHAC_01843		PTS fructose transporter subunit IIABC
contig_1	cds	159748	159776	+	GLNHAC_01844	lepB	(pseudo) signal peptidase I
contig_1	cds	159780	159806	+	GLNHAC_01845	sipU	signal peptidase I sipU
contig_1	cds	159810	159837	-	GLNHAC_01846	ykoA	Uncharacterized protein YkoA
contig_1	cds	159855	159953	+	GLNHAC_01847		(pseudo) ABC-F family ATP-binding cassette domain-containi
contig_1	cds	159951	160017	+	GLNHAC_01848		(pseudo) ABC-F family ATP-binding cassette domain-containi
contig_1	cds	160023	160099	+	GLNHAC_01849		ketopantoate reductase family protein
contig_1	cds	160098	160114	+	GLNHAC_01850		2-dehydropantoate 2-reductase
contig_1	cds	160117	160180	-	GLNHAC_01851	pepS	Aminopeptidase PepS
contig_1	cds	160197	160240	-	GLNHAC_01852		Aminopeptidase
contig_1	cds	160252	160265	-	GLNHAC_01853	ykpC	Uncharacterized protein YkpC
contig_1	cds	160275	160377	-	GLNHAC_01854	mreBH	cell shape-determining protein MreBH
contig_1	cds	160404	160432	+	GLNHAC_01855	abhA	transcriptional regulator AbhA
contig_1	cds	160443	160580	+	GLNHAC_01856	kinC	two-component sensor histidine kinase KinC
contig_1	cds	160581	160641	+	GLNHAC_01857		hypothetical protein
contig_1	cds	160671	160738	+	GLNHAC_01858	ktrC	Ktr system potassium transporter KtrC
contig_1	cds	160753	160927	+	GLNHAC_01859	ade	adenine deaminase
contig_1	cds	160930	161097	-	GLNHAC_01860	rnjA	ribonuclease J1
contig_1	cds	161097	161118	-	GLNHAC_01861	rnpZA	RNA polymerase subunit omega 1
contig_1	cds	161152	161234	+	GLNHAC_01862	ykrA	Putative phosphatase YkrA
contig_1	cds	161238	161293	-	GLNHAC_01863	def	peptide deformylase
contig_1	cds	161304	161319	-	GLNHAC_01864	ykzV	Uncharacterized membrane protein YkzV
contig_1	cds	161344	161408	+	GLNHAC_01865	ykyA	YkyA family protein
contig_1	cds	161450	161562	+	GLNHAC_01866	pdhA	pyruvate dehydrogenase (acetyl-transferring) E1 component s
contig_1	cds	161562	161660	+	GLNHAC_01867	pdhB	pyruvate dehydrogenase complex E1 component subunit bet
contig_1	cds	161671	161804	+	GLNHAC_01868	pdhC	pyruvate dehydrogenase complex dihydrolipoyllysine-residue
contig_1	cds	161804	161946	+	GLNHAC_01869	lpdA	dihydrolipoyl dehydrogenase
contig_1	cds	161950	161988	-	GLNHAC_01870	slp	peptidoglycan-associated lipoprotein Slp
contig_1	ncRNA	162025	162045	+	GLNHAC_01871	antisense_SR1	SR1 sRNA
contig_1	cds	162045	162192	-	GLNHAC_01872	speA	arginine decarboxylase
contig_1	cds	162211	162238	+	GLNHAC_01873	yktA	UPF0223 protein YktA
contig_1	cds	162241	162305	-	GLNHAC_01874	yktB	UPF0637 protein YktB
contig_1	cds	162323	162347	+	GLNHAC_01875	ykzI	Uncharacterized protein YkzI
contig_1	cds	162361	162441	+	GLNHAC_01876	suhB	Inositol-1-monophosphatase
contig_1	cds	162440	162486	+	GLNHAC_01877	ykzC	Uncharacterized protein YkzC
contig_1	cds	162494	162585	-	GLNHAC_01878	yktD	Putative S-adenosyl-L-methionine-dependent methyltransfera
contig_1	cds	162629	162786	-	GLNHAC_01879	nprE	neutral metalloprotease NprE
contig_1	cds	162816	162965	+	GLNHAC_01880	ylaA	(pseudo) YlaA
contig_1	cds	163008	163035	+	GLNHAC_01881		Anti-sigma factor
contig_1	cds	163035	163066	+	GLNHAC_01882	ylaC	RNA polymerase ECF-type sigma factor YlaC
contig_1	cds	163077	163098	+	GLNHAC_01883		hypothetical protein
contig_1	cds	163099	163116	+	GLNHAC_01884	ylaD	(pseudo) YlaD
contig_1	cds	163120	163181	-	GLNHAC_01885	ylaE	Uncharacterized protein YlaE
contig_1	cds	163208	163227	-	GLNHAC_01886	ylaF	Uncharacterized protein YlaF
contig_1	cds	163238	163410	+	GLNHAC_01887	typA	translational GTPase TypA
contig_1	cds	163428	163459	+	GLNHAC_01888	ylaH	Uncharacterized membrane protein YlaH
contig_1	cds	163465	163486	-	GLNHAC_01889	ylaI	Uncharacterized protein YlaI
contig_1	cds	163494	163557	-	GLNHAC_01890	ylaJ	putative spore germination lipoprotein YlaJ
contig_1	cds	163573	163639	+	GLNHAC_01891		PIN domain-containing protein
contig_1	cds	163645	163705	+	GLNHAC_01892		PhoH-like protein domain-containing protein
contig_1	cds	163706	163754	-	GLNHAC_01893	ylaL	Uncharacterized protein YlaL
contig_1	cds	163764	163836	+	GLNHAC_01894		glutaminase
contig_1	cds	163836	163852	+	GLNHAC_01895		(pseudo) glutaminase
contig_1	cds	163867	163895	+	GLNHAC_01896	ylaN	UPF0358 protein YlaN
contig_1	cds	163915	164036	+	GLNHAC_01897	ftsW	cell division protein FtsW
contig_1	cds	164044	164388	+	GLNHAC_01898	pyc	pyruvate carboxylase
contig_1	cds	164433	164525	-	GLNHAC_01899	ctaA	heme A synthase CtaA
contig_1	cds	164561	164644	+	GLNHAC_01900	cyoE	heme o synthase
contig_1	cds	164676	164783	+	GLNHAC_01901	coxB	cytochrome c oxidase subunit II
contig_1	cds	164786	164973	+	GLNHAC_01902	ctaD	cytochrome c oxidase subunit I
contig_1	cds	164973	164990	+	GLNHAC_01903		hypothetical protein
contig_1	cds	164985	165035	+	GLNHAC_01904	ctaE	Cytochrome c oxidase subunit 3
contig_1	cds	165035	165069	+	GLNHAC_01905	ctaF	cytochrome c oxidase subunit IVB
contig_1	cds	165071	165161	+	GLNHAC_01906	ctaG	cytochrome c oxidase assembly factor CtaG
contig_1	cds	165164	165200	-	GLNHAC_01907	ylbA	Uncharacterized protein YlbA
contig_1	cds	165214	165259	+	GLNHAC_01908	ylbB	Uncharacterized protein YlbB
contig_1	cds	165265	165371	+	GLNHAC_01909	ylbC	Uncharacterized membrane protein YlbC
contig_1	cds	165400	165440	+	GLNHAC_01910	ylbD	Uncharacterized protein YlbD
contig_1	cds	165442	165465	+	GLNHAC_01911	ylbE	Uncharacterized protein YlbE
contig_1	cds	165477	165522	+	GLNHAC_01912	ylbF	competence/sporulation regulator complex protein RicF

contig_1	cds	165530	165555	+	GLNHAC_01913	ylbG	YlbG family protein
contig_1	ncRNA-region	165556	165568	+			ylbH leader
contig_1	cds	165588	165643	+	GLNHAC_01914	rsmD	16S rRNA (guanine(966)-N(2))-methyltransferase RsmD
contig_1	cds	165643	165692	+	GLNHAC_01915	coaD	pantetheine-phosphate adenylyltransferase
contig_1	cds	165693	165816	-	GLNHAC_01916	ylbJ	dipicolinic acid transporter SpoVV
contig_1	cds	165834	165912	+	GLNHAC_01917	ylbK	Uncharacterized NTE family protein YlbK
contig_1	cds	165912	166015	+	GLNHAC_01918	ylbL	protease DdcP
contig_1	cds	166016	166141	-	GLNHAC_01919	tmcAL	nucleotidyltransferase
contig_1	cds	166162	166214	+	GLNHAC_01920	ylbN	Uncharacterized protein YlbN
contig_1	cds	166216	166234	+	GLNHAC_01921	rpmF	50S ribosomal protein L32
contig_1	cds	166248	166306	+	GLNHAC_01922	gerR	sporulation specific transcriptional regulator GerR
contig_1	cds	166312	166360	-	GLNHAC_01923	ylbP	N-acetyltransferase
contig_1	cds	166376	166466	+	GLNHAC_01924	panE	2-dehydropantoate 2-reductase
contig_1	cds	166473	166635	+	GLNHAC_01925	bshC	bacillithiol biosynthesis cysteine-adding enzyme BshC
contig_1	cds	166646	166691	+	GLNHAC_01926	mraZ	division/cell wall cluster transcriptional repressor MraZ
contig_1	cds	166698	166791	+	GLNHAC_01927	rsmH	16S rRNA (cytosine(1402)-N(4))-methyltransferase RsmH
contig_1	cds	166795	166830	+	GLNHAC_01928	ftsL	cell division protein FtsL
contig_1	cds	166831	166865	+	GLNHAC_01929		hypothetical protein
contig_1	cds	166890	167045	+	GLNHAC_01930		Penicillin-binding protein
contig_1	cds	167058	167252	+	GLNHAC_01931	spoVD	stage V sporulation protein D
contig_1	cds	167270	167350	+	GLNHAC_01932	murE	(pseudo) UDP-N-acetylmuramoyl-L-alanyl-D-glutamate--2,6-(
contig_1	cds	167350	167418	+	GLNHAC_01933		UDP-N-acetylmuramoyl-L-alanyl-D-glutamate--2, 6-diaminop
contig_1	cds	167429	167527	+	GLNHAC_01934	mraY	phospho-N-acetylmuramoyl-pentapeptide-transferase
contig_1	cds	167527	167662	+	GLNHAC_01935	murD	UDP-N-acetylmuramoyl-L-alanine--D-glutamate ligase
contig_1	cds	167676	167776	+	GLNHAC_01936	spoVE	stage V sporulation protein E
contig_1	cds	167790	167899	+	GLNHAC_01937	murG	undecaprenyldiphospho-muramoylpentapeptide beta-N-acety
contig_1	cds	167911	167994	+	GLNHAC_01938	murB	UDP-N-acetylmuramate dehydrogenase
contig_1	cds	168007	168086	+	GLNHAC_01939	divIB	cell division protein DivIB
contig_1	cds	168086	168155	+	GLNHAC_01940	ylxW	UPF0749 protein YlxW
contig_1	cds	168161	168228	+	GLNHAC_01941	ylxX	UPF0749 protein YlxX
contig_1	cds	168230	168266	+	GLNHAC_01942	sbp	Small basic protein
contig_1	cds	168284	168416	+	GLNHAC_01943	ftsA	cell division protein FtsA
contig_1	cds	168420	168466	+	GLNHAC_01944	ftsZ	(pseudo) Cell division protein FtsZ
contig_1	cds	168466	168534	+	GLNHAC_01945		Tubulin/FtsZ 2-layer sandwich domain-containing protein
contig_1	cds	168565	168995	+	GLNHAC_01946	bpr	Bacillopeptidase F
contig_1	cds	169012	169107	+	GLNHAC_01947	spoIIGA	sigma-E processing peptidase SpoIIGA
contig_1	cds	169113	169185	+	GLNHAC_01948	sigE	RNA polymerase sporulation sigma factor SigE
contig_1	cds	169199	169277	+	GLNHAC_01949	sigG	RNA polymerase sporulation sigma factor SigG
contig_1	cds	169293	169372	+	GLNHAC_01950	ylmA	Uncharacterized ABC transporter ATP-binding protein YlmA
contig_1	ncRNA-region	169373	169383	+			TPP riboswitch aptamer (THI element)
contig_1	cds	169392	169520	+	GLNHAC_01951	ylmB	acetylornithine deacetylase
contig_1	cds	169528	169553	+	GLNHAC_01952	ylmC	putative sporulation protein YlmC
contig_1	cds	169569	169652	+	GLNHAC_01953	pgeF	peptidoglycan editing factor PgeF
contig_1	cds	169653	169722	+	GLNHAC_01954	ylmE	Pyridoxal phosphate homeostasis protein
contig_1	cds	169722	169768	+	GLNHAC_01955	sepF	Cell division protein SepF
contig_1	cds	169768	169795	+	GLNHAC_01956	ylmG	Uncharacterized membrane protein YlmG
contig_1	cds	169802	169879	+	GLNHAC_01957	rqcP2	Ribosome-associated protein quality control protein P2
contig_1	cds	169888	169938	+	GLNHAC_01958	divIVA	septum site-determining protein DivIVA
contig_1	ncRNA-region	169944	169967	+			T-box leader
contig_1	cds	169972	170248	+	GLNHAC_01959	ileS	isoleucine--tRNA ligase
contig_1	cds	170263	170300	+	GLNHAC_01960	ylyA	sporulation-related RNA polymerase-binding protein YlyA
contig_1	cds	170311	170359	+	GLNHAC_01961	lspA	signal peptidase II
contig_1	cds	170357	170438	+	GLNHAC_01962	ylyB	Uncharacterized RNA pseudouridine synthase YlyB
contig_1	ncRNA-region	170452	170464	+			PyrR binding site
contig_1	cds	170492	170521	+	GLNHAC_01963	pyrR	Bifunctional pyr operon transcriptional regulator/uracil phosph
contig_1	ncRNA-region	170521	170533	+			PyrR binding site
contig_1	cds	170547	170669	+	GLNHAC_01964	pyrP	uracil permease PyrP
contig_1	ncRNA-region	170669	170681	+			PyrR binding site
contig_1	cds	170684	170775	+	GLNHAC_01965	pyrB	aspartate carbamoyltransferase catalytic subunit
contig_1	cds	170774	170902	+	GLNHAC_01966	pyrC	dihydroorotase
contig_1	cds	170902	170917	+	GLNHAC_01967		hypothetical protein
contig_1	cds	170924	171011	+	GLNHAC_01968		carbamoyl-phosphate synthase (glutamine-hydrolyzing)
contig_1	cds	171021	171233	+	GLNHAC_01969		carbamoyl-phosphate synthase (ammonia)
contig_1	cds	171236	171331	+	GLNHAC_01970		Carbamoyl-phosphate synthase large chain
contig_1	cds	171331	171381	+	GLNHAC_01971	pyrK	(pseudo) dihydroorotate dehydrogenase electron transfer sub
contig_1	cds	171408	171464	+	GLNHAC_01972	pyrD	(pseudo) dihydroorotate dehydrogenase
contig_1	cds	171464	171501	+	GLNHAC_01973		nitronate monooxygenase
contig_1	cds	171498	171570	+	GLNHAC_01974	pyrF	orotidine-5'-phosphate decarboxylase
contig_1	cds	171570	171633	+	GLNHAC_01975	pyrE	Orotate phosphoribosyltransferase
contig_1	ncRNA-region	171648	171658	+			SAM riboswitch aptamer (S box leader)
contig_1	cds	171675	171745	+	GLNHAC_01976	cysH	phosphoadenylyl-sulfate reductase
contig_1	cds	171746	171758	+	GLNHAC_01977		(pseudo) inorganic phosphate transporter
contig_1	cds	171758	171852	+	GLNHAC_01978	cysP	Sulfate permease CysP
contig_1	cds	171857	171972	+	GLNHAC_01979	sat	Sulfate adenylyltransferase
contig_1	cds	171973	172033	+	GLNHAC_01980	cysC	putative adenylyl-sulfate kinase
contig_1	cds	172042	172120	+	GLNHAC_01981	sumT	Uroporphyrinogen-III C-methyltransferase
contig_1	cds	172120	172175	+	GLNHAC_01982	sirB	(pseudo) Sirohydrochlorin ferrochelatase
contig_1	cds	172176	172199	+	GLNHAC_01983		CbiX/SirB N-terminal domain-containing protein
contig_1	cds	172197	172246	+	GLNHAC_01984		precorrin-2 dehydrogenase
contig_1	cds	172250	172421	-	GLNHAC_01985	rqcH	tRNA modification protein RqcH
contig_1	cds	172433	172544	+	GLNHAC_01986		HAD-IC family P-type ATPase
contig_1	cds	172539	172700	+	GLNHAC_01987	yloB	(pseudo) calcium-translocating P-type ATPase, SERCA-type
contig_1	cds	172708	172796	+	GLNHAC_01988	yloC	Endoribonuclease YloC
contig_1	cds	172803	172830	+	GLNHAC_01989	remA	extracellular matrix/biofilm regulator RemA
contig_1	cds	172831	172893	+	GLNHAC_01990	gmk	guanylate kinase
contig_1	cds	172893	172913	+	GLNHAC_01991	rpoZ	DNA-directed RNA polymerase subunit omega
contig_1	cds	172921	173043	+	GLNHAC_01992	coaBC	bifunctional phosphopantothenoylcysteine decarboxylase/pho
contig_1	cds	173043	173285	+	GLNHAC_01993	priA	primosomal protein N'
contig_1	cds	173288	173336	+	GLNHAC_01994	def	peptide deformylase
contig_1	cds	173336	173432	+	GLNHAC_01995	fmt	methionyl-tRNA formyltransferase
contig_1	cds	173430	173477	+	GLNHAC_01996		hypothetical protein
contig_1	cds	173502	173564	+	GLNHAC_01997		SAM-dependent MTase RsmB/NOP-type domain-containing
contig_1	cds	173565	173674	+	GLNHAC_01998	rlmN	23S rRNA (adenine(2503)-C(2))-methyltransferase RlmN
contig_1	cds	173674	173710	+	GLNHAC_01999		(pseudo) protein phosphatase 2C domain-containing protein
contig_1	cds	173704	173751	+	GLNHAC_02000		PPM-type phosphatase domain-containing protein
contig_1	cds	173750	173945	+	GLNHAC_02001	prkC	serine/threonine protein kinase PrkC
contig_1	cds	173946	174023	+	GLNHAC_02002	rsgA	ribosome small subunit-dependent GTPase A
contig_1	cds	174017	174036	+	GLNHAC_02003	rsgA	(pseudo) ribosome small subunit-dependent GTPase A
contig_1	cds	174036	174102	+	GLNHAC_02004	rpe	ribulose-phosphate 3-epimerase
contig_1	cds	174109	174173	+	GLNHAC_02005	thiN	thiamine diphosphokinase
contig_1	sorf	174180	174188	+	GLNHAC_02006	spoVM	stage V sporulation protein SpoVM
contig_1	cds	174195	174214	-	GLNHAC_02007	rpmB	50S ribosomal protein L28
contig_1	cds	174242	174278	+	GLNHAC_02008	yloU	Uncharacterized protein YloU
contig_1	cds	174280	174446	+	GLNHAC_02009	yloV	Protein YloV
contig_1	cds	174460	174526	+	GLNHAC_02010	sdaAB	L-serine ammonia-lyase, iron-sulfur-dependent subunit beta
contig_1	cds	174529	174619	+	GLNHAC_02011	sdaAA	L-serine ammonia-lyase, iron-sulfur-dependent, subunit alpha
contig_1	cds	174617	174632	+	GLNHAC_02012		hypothetical protein
contig_1	cds	174647	174790	+	GLNHAC_02013	recG	(pseudo) ATP-dependent DNA helicase RecG
contig_1	cds	174790	174821	+	GLNHAC_02014		(pseudo) Helicase C-terminal domain-containing protein
contig_1	cds	174832	174869	+	GLNHAC_02015	fapR	(pseudo) transcription factor FapR
contig_1	cds	174867	174889	+	GLNHAC_02016	fapR	(pseudo) transcription factor FapR
contig_1	cds	174890	174976	+	GLNHAC_02017	plsX	phosphate acyltransferase PlsX
contig_1	cds	174992	175087	+	GLNHAC_02018	fabD	ACP S-malonyltransferase
contig_1	cds	175087	175161	+	GLNHAC_02019	fabG	3-oxoacyl-[acyl-carrier-protein] reductase
contig_1	cds	175169	175192	+	GLNHAC_02020	acpP	acyl carrier protein
contig_1	cds	175206	175281	+	GLNHAC_02021	rncS	ribonuclease III
contig_1	cds	175285	175295	+	GLNHAC_02022		hypothetical protein
contig_1	cds	175292	175360	+	GLNHAC_02023		Chromosome partition protein Smc
contig_1	cds	175366	175647	+	GLNHAC_02024	smc	chromosome segregation protein SMC
contig_1	cds	175649	175748	+	GLNHAC_02025	ftsY	signal recognition particle-docking protein FtsY
contig_1	cds	175753	175801	-	GLNHAC_02026		hypothetical protein
contig_1	cds	175819	175852	+	GLNHAC_02027	ylxM	putative DNA-binding protein
contig_1	cds	175853	175987	+	GLNHAC_02028	ffh	signal recognition particle protein
contig_1	cds	175998	176025	+	GLNHAC_02029	rpsP	30S ribosomal protein S16
contig_1	cds	176062	176100	+	GLNHAC_02030	ylqD	Uncharacterized protein YlqD
contig_1	cds	176101	176153	+	GLNHAC_02031	rimM	ribosome maturation factor RimM
contig_1	cds	176153	176183	+	GLNHAC_02032	trmD	(pseudo) tRNA (guanosine(37)-N1)-methyltransferase TrmD
contig_1	cds	176185	176226	+	GLNHAC_02033		tRNA (guanine-N(1)-)-methyltransferase
contig_1	ncRNA-region	176234	176238	+			Ribosomal protein L19 leader
contig_1	cds	176240	176274	+	GLNHAC_02034	rplS	50S ribosomal protein L19
contig_1	cds	176289	176374	+	GLNHAC_02035	ylqF	ribosome biogenesis GTPase YlqF
contig_1	cds	176381	176437	+	GLNHAC_02036		Ribonuclease HII
contig_1	cds	176443	176461	+	GLNHAC_02037		hypothetical protein
contig_1	cds	176461	176472	+	GLNHAC_02038		hypothetical protein
contig_1	cds	176483	176633	+	GLNHAC_02039	ylqG	Uncharacterized protein YlqG
contig_1	cds	176633	176661	+	GLNHAC_02040	ylqH	Uncharacterized protein YlqH
contig_1	cds	176678	176794	+	GLNHAC_02041	sucC	ADP-forming succinate--CoA ligase subunit beta
contig_1	cds	176797	176887	+	GLNHAC_02042	sucD	succinate--CoA ligase subunit alpha
contig_1	cds	176893	176983	+	GLNHAC_02043	dprA	DNA-processing protein DprA

contig_1	cds	177001	177209	+	GLNHAC_02044	topA	type I DNA topoisomerase
contig_1	cds	177216	177347	+	GLNHAC_02045	trmFO	FADH(2)-oxidizing methylenetetrahydrofolate--tRNA-(uracil(54
contig_1	cds	177354	177445	+	GLNHAC_02046	xerC	tyrosine recombinase XerC
contig_1	cds	177447	177501	+	GLNHAC_02047	hslV	ATP-dependent protease subunit ClpQ
contig_1	cds	177503	177559	+	GLNHAC_02048	hslU	(pseudo) HslU--HslV peptidase ATPase subunit
contig_1	cds	177557	177643	+	GLNHAC_02049	hslU	(pseudo) HslU--HslV peptidase ATPase subunit
contig_1	cds	177647	177725	+	GLNHAC_02050	codY	GTP-sensing pleiotropic transcriptional regulator CodY
contig_1	cds	177763	177802	+	GLNHAC_02051	flgB	flagellar basal body rod protein FlgB
contig_1	cds	177802	177847	+	GLNHAC_02052	flgC	flagellar basal body rod protein FlgC
contig_1	cds	177848	177880	+	GLNHAC_02053	fliE	flagellar hook-basal body complex protein FliE
contig_1	cds	177885	178046	+	GLNHAC_02054	fliF	flagellar basal-body MS-ring/collar protein FliF
contig_1	cds	178047	178149	+	GLNHAC_02055	fliG	flagellar motor switch protein FliG
contig_1	cds	178148	178223	+	GLNHAC_02056	fliH	flagellar assembly protein FliH
contig_1	cds	178223	178248	+	GLNHAC_02057		hypothetical protein
contig_1	cds	178254	178278	+	GLNHAC_02058		hypothetical protein
contig_1	cds	178273	178355	+	GLNHAC_02059	fliI	(pseudo) flagellar protein export ATPase FliI
contig_1	cds	178355	178399	+	GLNHAC_02060	fliJ	flagellar export protein FliJ
contig_1	cds	178401	178462	+	GLNHAC_02061	ylxF	FlaA locus protein
contig_1	cds	178463	178610	+	GLNHAC_02062	fliK	flagellar hook-length control protein FliK
contig_1	cds	178610	178649	+	GLNHAC_02063	flgD	flagellar hook assembly protein FlgD
contig_1	cds	178654	178733	+	GLNHAC_02064	flgG	flagellar basal body rod protein FlgG
contig_1	cds	178758	178801	+	GLNHAC_02065	fliL	flagellar basal body-associated protein FliL
contig_1	cds	178811	178904	+	GLNHAC_02066	fliM	flagellar motor switch protein FliM
contig_1	cds	178903	179017	+	GLNHAC_02067	fliY	flagellar motor switch phosphatase FliY
contig_1	cds	179019	179056	+	GLNHAC_02068	cheY	chemotaxis protein CheY
contig_1	cds	179057	179123	+	GLNHAC_02069	fliZ	flagella biosynthesis regulatory protein FliZ
contig_1	cds	179122	179189	+	GLNHAC_02070	fliP	flagellar type III secretion system pore protein FliP
contig_1	cds	179190	179217	+	GLNHAC_02071	fliQ	flagellar biosynthesis protein FliQ
contig_1	cds	179218	179296	+	GLNHAC_02072	fliR	flagellar biosynthetic protein FliR
contig_1	cds	179296	179404	+	GLNHAC_02073	flhB	flagellar biosynthesis protein FlhB
contig_1	cds	179407	179611	+	GLNHAC_02074	flhA	flagellar biosynthesis protein FlhA
contig_1	cds	179611	179721	+	GLNHAC_02075	flhF	flagellar biosynthesis protein FlhF
contig_1	cds	179720	179809	+	GLNHAC_02076	flhG	flagellum location/number ATPase FlhG
contig_1	cds	179810	179846	+	GLNHAC_02077		Stage 0 sporulation A-like protein
contig_1	cds	179917	180119	+	GLNHAC_02078	cheA	chemotaxis protein CheA
contig_1	cds	180121	180168	+	GLNHAC_02079	cheW	chemotaxis protein CheW
contig_1	cds	180170	180233	+	GLNHAC_02080	cheC	CheY-P phosphatase CheC
contig_1	cds	180232	180282	+	GLNHAC_02081	cheD	chemoreceptor glutamine deamidase CheD
contig_1	cds	180285	180361	+	GLNHAC_02082	sigD	RNA polymerase sigma-28 factor SigD
contig_1	cds	180364	180402	+	GLNHAC_02083	swrB	(pseudo) Swarming motility protein SwrB
contig_1	cds	180429	180503	+	GLNHAC_02084	rpsB	30S ribosomal protein S2
contig_1	cds	180513	180601	+	GLNHAC_02085	tsf	translation elongation factor Ts
contig_1	cds	180616	180640	+	GLNHAC_02086		hypothetical protein
contig_1	cds	180636	180688	+	GLNHAC_02087		Uridylate kinase
contig_1	cds	180688	180744	+	GLNHAC_02088	frr	ribosome recycling factor
contig_1	cds	180757	180835	+	GLNHAC_02089	uppS	isoprenyl transferase
contig_1	cds	180836	180916	+	GLNHAC_02090	cdsA	phosphatidate cytidylyltransferase
contig_1	cds	180922	181037	+	GLNHAC_02091	dxr	1-deoxy-D-xylulose-5-phosphate reductoisomerase
contig_1	cds	181039	181165	+	GLNHAC_02092	rseP	RIP metalloprotease RseP
contig_1	cds	181168	181338	+	GLNHAC_02093	proS	proline--tRNA ligase
contig_1	cds	181343	181451	+	GLNHAC_02094		hypothetical protein
contig_1	cds	181449	181780	+	GLNHAC_02095	polC	(pseudo) PolC-type DNA polymerase III
contig_1	cds	181813	181860	+	GLNHAC_02096	rimP	ribosome maturation factor RimP
contig_1	cds	181863	181976	+	GLNHAC_02097	nusA	Transcription termination/antitermination protein NusA
contig_1	cds	181976	182004	+	GLNHAC_02098	rulR	glucose-induced regulator RulR
contig_1	cds	182004	182034	+	GLNHAC_02099	rulQ	YlxQ family RNA-binding protein
contig_1	cds	182036	182251	+	GLNHAC_02100	infB	translation initiation factor IF-2
contig_1	cds	182251	182278	+	GLNHAC_02101	ylxP	Uncharacterized protein YlxP
contig_1	cds	182280	182315	+	GLNHAC_02102	rbfA	30S ribosome-binding factor RbfA
contig_1	cds	182324	182365	+	GLNHAC_02103	truB	tRNA pseudouridine(55) synthase TruB
contig_1	cds	182367	182417	+	GLNHAC_02104	truB	(pseudo) tRNA pseudouridine(55) synthase TruB
contig_1	cds	182418	182513	+	GLNHAC_02105	ribF	bifunctional riboflavin kinase/FAD synthetase
contig_1	cds	182529	182556	+	GLNHAC_02106	rpsO	30S ribosomal protein S15
contig_1	cds	182573	182785	+	GLNHAC_02107	pnp	polyribonucleotide nucleotidyltransferase
contig_1	cds	182797	182893	+	GLNHAC_02108	ylxY	Uncharacterized protein YlxY
contig_1	cds	182897	182973	+	GLNHAC_02109		pitrilysin family protein
contig_1	cds	182976	182999	+	GLNHAC_02110		(pseudo) pitrilysin family protein
contig_1	cds	182999	183020	+	GLNHAC_02111		(pseudo) pitrilysin family protein
contig_1	cds	183028	183053	+	GLNHAC_02112	ymxH	Uncharacterized protein YmxH
contig_1	cds	183073	183104	+	GLNHAC_02113	dpsA	(pseudo) dipicolinate synthase subunit DpsA
contig_1	cds	183123	183162	+	GLNHAC_02114	dpaA	(pseudo) dipicolinic acid synthetase subunit A
contig_1	cds	183162	183223	+	GLNHAC_02115	dpaB	dipicolinate synthase subunit B
contig_1	cds	183235	183339	+	GLNHAC_02116	asd	aspartate-semialdehyde dehydrogenase
contig_1	cds	183348	183470	+	GLNHAC_02117	dapG	aspartate kinase
contig_1	cds	183473	183560	+	GLNHAC_02118	dapA	4-hydroxy-tetrahydrodipicolinate synthase
contig_1	cds	183578	183731	+	GLNHAC_02119	rnjB	Ribonuclease J2
contig_1	cds	183731	183745	+	GLNHAC_02120		hypothetical protein
contig_1	cds	183757	183830	+	GLNHAC_02121	tepA	translocation-enhancing protein TepA
contig_1	cds	183830	183851	+	GLNHAC_02122	tepJ	TepA modulator TepJ
contig_1	cds	183864	184100	+	GLNHAC_02123	spoIIIE	DNA translocase SpoIIIE
contig_1	cds	184121	184187	+	GLNHAC_02124	ymfC	Uncharacterized HTH-type transcriptional regulator YmfC
contig_1	cds	184201	184321	+	GLNHAC_02125	ymfD	bacillibactin exporter BcbE
contig_1	cds	184347	184475	+	GLNHAC_02126	yfmF	EF-P 5-aminopentanol modification-associated protein YfmF
contig_1	cds	184475	184580	+	GLNHAC_02127		Insulinase family protein
contig_1	cds	184601	184681	+	GLNHAC_02128	ymfI	EF-P-5 aminopentanone reductase
contig_1	cds	184689	184715	+	GLNHAC_02129		DUF3243 domain-containing protein
contig_1	cds	184728	184807	+	GLNHAC_02130	ymfK	Uncharacterized protein YmfK
contig_1	cds	184809	184892	+	GLNHAC_02131	ymfM	Uncharacterized membrane protein YmfM
contig_1	cds	184936	184962	+	GLNHAC_02132	pgsA	(pseudo) CDP-diacylglycerol--glycerol-3-phosphate 3-phosph
contig_1	cds	184964	185089	+	GLNHAC_02133	cinA	competence/damage-inducible protein A
contig_1	cds	185106	185211	+	GLNHAC_02134	recA	recombinase RecA
contig_1	cds	185228	185345	+	GLNHAC_02135	pbpX	Putative penicillin-binding protein PbpX
contig_1	cds	185373	185529	+	GLNHAC_02136	rny	ribonuclease Y
contig_1	cds	185536	185616	+	GLNHAC_02137	ymdB	2',3'-cyclic-nucleotide 2'-phosphodiesterase
contig_1	cds	185636	185662	+	GLNHAC_02138	spoVS	stage V sporulation protein SpoVS
contig_1	cds	185688	185792	+	GLNHAC_02139	tdh	L-threonine 3-dehydrogenase
contig_1	cds	185794	185911	+	GLNHAC_02140	kbl	glycine C-acetyltransferase
contig_1	cds	185926	186079	+	GLNHAC_02141	miaB	tRNA (N6-isopentenyl adenosine(37)-C2)-methylthiotransferas
contig_1	cds	186079	186122	+	GLNHAC_02142	ricA	RicAFT regulatory complex protein RicA
contig_1	cds	186148	186202	+	GLNHAC_02143	cotE	outer spore coat protein CotE
contig_1	cds	186216	186473	+	GLNHAC_02144	mutS	DNA mismatch repair protein MutS
contig_1	cds	186474	186664	+	GLNHAC_02145	mutL	DNA mismatch repair endonuclease MutL
contig_1	ncRNA	186684	186699	+	GLNHAC_02146	ncr952	Bacillus sRNA ncr952
contig_1	cds	186720	186767	-	GLNHAC_02147	yrvL	regulatory YrvL family protein
contig_1	cds	186771	186781	-	GLNHAC_02148		hypothetical protein
contig_1	cds	186781	186837	-	GLNHAC_02149	ymcC	Uncharacterized membrane protein YmcC
contig_1	cds	186849	186911	+	GLNHAC_02150		Putative transcriptional regulator
contig_1	cds	186930	186997	+	GLNHAC_02151	pksB	putative polyketide biosynthesis zinc-dependent hydrolase Pk
contig_1	cds	187035	187122	+	GLNHAC_02152	fabD	ACP S-malonyltransferase
contig_1	cds	187173	187270	+	GLNHAC_02153		Polyketide synthesis protein
contig_1	cds	187270	187443	+	GLNHAC_02154	fabD	(pseudo) ACP S-malonyltransferase
contig_1	cds	187450	187498	+	GLNHAC_02155		hypothetical protein
contig_1	cds	187507	187532	+	GLNHAC_02156	acpK	acyl carrier protein
contig_1	cds	187530	187655	+	GLNHAC_02157	pksF	Polyketide biosynthesis malonyl-ACP decarboxylase PksF
contig_1	cds	187655	187781	+	GLNHAC_02158	pksG	Polyketide biosynthesis 3-hydroxy-3-methylglutaryl-ACP synt
contig_1	cds	187781	187858	+	GLNHAC_02159	pksH	enoyl-CoA hydratase/isomerase
contig_1	cds	187862	187937	+	GLNHAC_02160	pksI	Putative polyketide biosynthesis enoyl-CoA isomerase PksI
contig_1	cds	187941	189188	+	GLNHAC_02161		Polyketide synthase of type I
contig_1	cds	189186	189295	+	GLNHAC_02162		hypothetical protein
contig_1	cds	189310	189455	+	GLNHAC_02163		beta-ketoacyl synthase N-terminal-like domain-containing pr
contig_1	cds	189456	189531	+	GLNHAC_02164		PKS/mFAS DH domain-containing protein
contig_1	cds	189534	189811	+	GLNHAC_02165		(pseudo) beta-ketoacyl synthase N-terminal-like domain-cont
contig_1	cds	189808	190044	+	GLNHAC_02166		hypothetical protein
contig_1	cds	190042	190275	+	GLNHAC_02167		Polyketide synthase of type I
contig_1	cds	190276	190531	+	GLNHAC_02168	pksL	Polyketide synthase PksL
contig_1	cds	190555	190824	+	GLNHAC_02169		beta-ketoacyl synthase N-terminal-like domain-containing pr
contig_1	cds	190826	190973	+	GLNHAC_02170		hypothetical protein
contig_1	cds	190990	191064	+	GLNHAC_02171		hypothetical protein
contig_1	cds	191056	191072	-	GLNHAC_02172		hypothetical protein
contig_1	cds	191087	191135	+	GLNHAC_02173		hypothetical protein
contig_1	cds	191138	192104	+	GLNHAC_02174		SDR family NAD(P)-dependent oxidoreductase
contig_1	cds	192103	192199	+	GLNHAC_02175		condensation domain-containing protein
contig_1	cds	192199	192641	+	GLNHAC_02176		non-ribosomal peptide synthetase
contig_1	cds	192638	192683	+	GLNHAC_02177		hypothetical protein
contig_1	cds	192680	193221	+	GLNHAC_02178		(pseudo) non-ribosomal peptide synthetase
contig_1	cds	193219	193272	+	GLNHAC_02179		hypothetical protein
contig_1	cds	193277	193324	+	GLNHAC_02180		hypothetical protein
contig_1	cds	193326	193348	+	GLNHAC_02181		hypothetical protein

**contig_1**	cds	193342	193756	+	GLNHAC_02182		hypothetical protein
**contig_1**	cds	193757	193777	+	GLNHAC_02183		hypothetical protein
**contig_1**	cds	193794	194256	+	GLNHAC_02184		Polyketide synthase
**contig_1**	cds	194250	194425	+	GLNHAC_02185		(pseudo) beta-ketoacyl synthase N-terminal-like domain-cont
**contig_1**	cds	194425	194520	+	GLNHAC_02186		hypothetical protein
**contig_1**	cds	194511	194586	-	GLNHAC_02187		Cytochrome P450
**contig_1**	cds	194582	194619	-	GLNHAC_02188		hypothetical protein
**contig_1**	cds	194615	194646	-	GLNHAC_02189		hypothetical protein
**contig_1**	cds	194667	194705	-	GLNHAC_02190	ymzB	Uncharacterized protein YmzB
**contig_1**	cds	194713	194795	-	GLNHAC_02191	ymaE	Uncharacterized protein YmaE
**contig_1**	cds	194806	194856	-	GLNHAC_02192		hypothetical protein
**contig_1**	cds	194850	194939	-	GLNHAC_02193	aprX	(pseudo) Serine protease AprX
**contig_1**	cds	194961	194984	+	GLNHAC_02194		WYL domain-containing protein
**contig_1**	cds	195012	195040	+	GLNHAC_02195		hypothetical protein
**contig_1**	cds	195036	195082	+	GLNHAC_02196		(pseudo) Poly-gamma-glutamate hydrolase family protein
**contig_1**	cds	195090	195135	+	GLNHAC_02197	osmC	OsmC family protein
**contig_1**	cds	195136	195172	-	GLNHAC_02198	ebrB	multidrug efflux SMR transporter subunit EbrB
**contig_1**	cds	195173	195205	-	GLNHAC_02199	ebrA	multidrug efflux SMR transporter subunit EbrA
**contig_1**	cds	195219	195244	-	GLNHAC_02200		Spore coat protein
**contig_1**	cds	195254	195294	+	GLNHAC_02201	ymaF	Uncharacterized protein YmaF
**contig_1**	cds	195304	195398	+	GLNHAC_02202	miaA	tRNA (adenosine(37)-N6)-dimethylallyltransferase MiaA
**contig_1**	cds	195402	195424	+	GLNHAC_02203	hfq	RNA chaperone Hfq
**contig_1**	cds	195444	195471	+	GLNHAC_02204	ymzC	Uncharacterized protein YmzC
**contig_1**	cds	195479	195502	+	GLNHAC_02205	ymzA	Uncharacterized protein YmzA
**contig_1**	cds	195562	195580	+	GLNHAC_02206		(pseudo) Ribonucleotide reductase N-terminal
**contig_1**	cds	195579	195772	+	GLNHAC_02207		Ribonucleoside-diphosphate reductase subunit alpha
**contig_1**	cds	195774	195873	+	GLNHAC_02208	nrdF	class 1b ribonucleoside-diphosphate reductase subunit beta
**contig_1**	cds	195878	195940	+	GLNHAC_02209	ymaB	Protein YmaB
**contig_1**	cds	195946	196023	-	GLNHAC_02210	cwlC	Sporulation-specific N-acetylmuramoyl-L-alanine amidase
**contig_1**	cds	196105	196202	+	GLNHAC_02211	spoVK	stage V sporulation protein K
**contig_1**	cds	196215	196341	+	GLNHAC_02212	hflX	GTPase HflX
**contig_1**	cds	196343	196431	+	GLNHAC_02213		methionine gamma-lyase family protein
**contig_1**	cds	196428	196469	+	GLNHAC_02214		methionine gamma-lyase family protein
**contig_1**	cds	196480	196521	+	GLNHAC_02215	glnR	transcriptional repressor GlnR
**contig_1**	cds	196527	196660	+	GLNHAC_02216	glnA	type I glutamate--ammonia ligase
**contig_1**	cds	196687	196775	+	GLNHAC_02217		Knr4/Smi1-like domain-containing protein
**contig_1**	cds	196877	196896	+	GLNHAC_02218		hypothetical protein
**contig_1**	cds	196907	196945	+	GLNHAC_02219		Uncharacterised protein family (UPF0715)
**contig_1**	cds	197038	197177	+	GLNHAC_02220	ynaJ	Uncharacterized symporter YnaJ
**contig_1**	cds	197181	197341	+	GLNHAC_02221	xynB	Beta-xylosidase
**contig_1**	cds	197359	197474	-	GLNHAC_02222	xylR	Xylose repressor
**contig_1**	cds	197500	197634	+	GLNHAC_02223	xylA	xylose isomerase
**contig_1**	cds	197651	197801	+	GLNHAC_02224	xylB	xylulokinase
**contig_1**	cds	197827	197897	+	GLNHAC_02225		Endo-1,4-beta-xylanase
**contig_1**	cds	197919	197989	-	GLNHAC_02226		Thermonuclease
**contig_1**	cds	198034	198175	+	GLNHAC_02227	yncC	Putative metabolite transport protein YncC
**contig_1**	cds	198190	198308	-	GLNHAC_02228	alr	alanine racemase
**contig_1**	cds	198323	198341	+	GLNHAC_02229		hypothetical protein
**contig_1**	cds	198347	198395	+	GLNHAC_02230	yncE	YncE
**contig_1**	cds	198398	198441	+	GLNHAC_02231	yncF	dUTP diphosphatase DutA
**contig_1**	cds	198454	198471	-	GLNHAC_02232		DUF1146 domain-containing protein
**contig_1**	cds	198486	198509	-	GLNHAC_02233		hypothetical protein
**contig_1**	cds	198509	198521	-	GLNHAC_02234		hypothetical protein
**contig_1**	cds	198573	198591	+	GLNHAC_02235		hypothetical protein
**contig_1**	cds	198605	198689	+	GLNHAC_02236	thyA	thymidylate synthase
**contig_1**	cds	198704	198725	-	GLNHAC_02237		tRNA processing exoribonuclease BN, putative
**contig_1**	cds	198741	198813	-	GLNHAC_02238	yncM	Uncharacterized protein YncM
**contig_1**	cds	198831	198867	-	GLNHAC_02239	yoqO	YoqO family protein
**contig_1**	cds	198891	198913	-	GLNHAC_02240		Spore coat protein C
**contig_1**	cds	198980	199020	+	GLNHAC_02241	yndA	Uncharacterized protein YndA
**contig_1**	cds	199026	199069	-	GLNHAC_02242		Activator of Hsp90 ATPase-like ue 1/2-like C-terminal domain
**contig_1**	cds	199085	199111	+	GLNHAC_02243		Histidine kinase
**contig_1**	cds	199113	199132	+	GLNHAC_02244	ynzB	Uncharacterized protein YnzB
**contig_1**	cds	199148	199268	+	GLNHAC_02245		Chromate efflux transporter
**contig_1**	cds	199282	199310	+	GLNHAC_02246		hypothetical protein
**contig_1**	cds	199325	199406	+	GLNHAC_02247		DoxX-like family protein
**contig_1**	cds	199400	199630	+	GLNHAC_02248	yndJ	Uncharacterized membrane protein YndJ
**contig_1**	cds	199641	199672	+	GLNHAC_02249		Phage/plasmid replication protein
**contig_1**	ncRNA	199672	199700	-	GLNHAC_02250	surA	SurA sRNA
**contig_1**	cds	199695	199771	+	GLNHAC_02251	yndL	UPF0714 protein YndL
**contig_1**	cds	199774	199819	-	GLNHAC_02252	yndM	Uncharacterized membrane protein YndM
**contig_1**	cds	199838	199881	+	GLNHAC_02253	fosM	FosM family fosfomycin resistance protein
**contig_1**	cds	199888	199931	+	GLNHAC_02254	yoaW	YoaW
**contig_1**	ncRNA	199957	199965	+	GLNHAC_02255	ncr982	Bacillus sRNA ncr982
**contig_1**	cds	199971	200032	-	GLNHAC_02256	lexA	transcriptional repressor LexA
**contig_1**	cds	200048	200079	+	GLNHAC_02257	yneA	cell division suppressor protein YneA
**contig_1**	cds	200081	200146	+	GLNHAC_02258	yneB	Resolvase-like protein YneB
**contig_1**	cds	200153	200169	+	GLNHAC_02259	ynzC	(pseudo) UPF0291 protein ynzC
**contig_1**	cds	200193	200393	+	GLNHAC_02260	tkt	transketolase
**contig_1**	cds	200409	200454	+	GLNHAC_02261	sirA	Sporulation inhibitor of replication protein SirA
**contig_1**	cds	200462	200484	+	GLNHAC_02262	yneF	YneF family protein
**contig_1**	cds	200491	200509	-	GLNHAC_02263	ynzD	aspartyl-phosphate phosphatase YnzD
**contig_1**	cds	200531	200601	+	GLNHAC_02264	ccdA	cytochrome c-type biogenesis protein CcdA
**contig_1**	cds	200610	200646	+	GLNHAC_02265	ccdB	Protein CcdB
**contig_1**	cds	200655	200703	+	GLNHAC_02266	ccdC	Protein CcdC
**contig_1**	cds	200707	200749	-	GLNHAC_02267	yneK	Membrane protein YneK
**contig_1**	cds	200773	200810	-	GLNHAC_02268	cotM	outer spore coat protein CotM
**contig_1**	cds	200820	200835	-	GLNHAC_02269	sspP	small acid-soluble spore protein P
**contig_1**	cds	200838	200854	-	GLNHAC_02270	sspO	small acid-soluble spore protein O
**contig_1**	cds	200875	200958	+	GLNHAC_02271		Aconitate hydratase
**contig_1**	cds	200957	201148	+	GLNHAC_02272	acnA	(pseudo) aconitate hydratase AcnA
**contig_1**	cds	201155	201184	+	GLNHAC_02273		hypothetical protein
**contig_1**	cds	201179	201206	+	GLNHAC_02274		redoxin domain-containing protein
**contig_1**	cds	201208	201227	+	GLNHAC_02275	fbpB	FbpB family small basic protein
**contig_1**	cds	201233	201248	+	GLNHAC_02276	sspN	acid-soluble spore protein SspN
**contig_1**	cds	201252	201277	+	GLNHAC_02277	tlp	small acid-soluble spore protein Tlp
**contig_1**	cds	201285	201327	+	GLNHAC_02278	yneP	Putative acyl-CoA thioesterase YneP
**contig_1**	cds	201328	201358	+	GLNHAC_02279	yneQ	Uncharacterized protein YneQ
**contig_1**	cds	201361	201390	-	GLNHAC_02280	pdhI	Pyruvate dehydrogenase inhibitor
**contig_1**	cds	201398	201456	-	GLNHAC_02281	plsY	glycerol-3-phosphate 1-O-acyltransferase PlsY
**contig_1**	cds	201473	201514	+	GLNHAC_02282	yneT	Uncharacterized protein YneT
**contig_1**	cds	201553	201750	+	GLNHAC_02283	parE	DNA topoisomerase IV subunit B
**contig_1**	cds	201750	201992	+	GLNHAC_02284	parC	DNA topoisomerase IV subunit A
**contig_1**	cds	201997	202010	-	GLNHAC_02285		hypothetical protein
**contig_1**	cds	202012	202048	-	GLNHAC_02286	ynfC	Uncharacterized protein YnfC
**contig_1**	cds	202097	202236	+	GLNHAC_02287	alsT	Amino-acid carrier protein AlsT
**contig_1**	cds	202268	202418	+	GLNHAC_02288		Endoglucanase
**contig_1**	cds	202426	202452	+	GLNHAC_02289	ynfE	YnfE family protein
**contig_1**	cds	202482	202522	+	GLNHAC_02290	yngA	YngA protein
**contig_1**	cds	202525	202614	+	GLNHAC_02291	galU	UTP--glucose-1-phosphate uridylyltransferase GalU
**contig_1**	cds	202620	202680	+	GLNHAC_02292	uptA	Undecaprenyl phosphate transporter A
**contig_1**	cds	202685	202805	-	GLNHAC_02293	nrnB	oligoribonuclease NrnB
**contig_1**	cds	202822	202975	-	GLNHAC_02294	yngE	Uncharacterized carboxylase YngE
**contig_1**	cds	202977	203055	-	GLNHAC_02295	yngF	enoyl-CoA hydratase
**contig_1**	cds	203057	203147	-	GLNHAC_02296	yngG	hydroxymethylglutaryl-CoA lyase
**contig_1**	cds	203148	203171	-	GLNHAC_02297	yngHB	acetyl-CoA carboxylase biotin carboxyl carrier protein subuni
**contig_1**	cds	203172	203290	-	GLNHAC_02298	accC2	Biotin carboxylase 2
**contig_1**	cds	203290	203305	-	GLNHAC_02299		hypothetical protein
**contig_1**	cds	203311	203355	-	GLNHAC_02300		hypothetical protein
**contig_1**	cds	203359	203403	-	GLNHAC_02301		hypothetical protein
**contig_1**	cds	203425	203451	-	GLNHAC_02302		(pseudo) AMP-binding protein
**contig_1**	cds	203458	203470	-	GLNHAC_02303		hypothetical protein
**contig_1**	cds	203470	203580	-	GLNHAC_02304	yngJ	putative acyl-CoA dehydrogenase YngJ
**contig_1**	cds	203605	203651	-	GLNHAC_02305		GNAT family protein
**contig_1**	cds	203662	203816	-	GLNHAC_02306	yngK	Glycosyl hydrolase YngK
**contig_1**	cds	203831	203870	-	GLNHAC_02307	yngL	UPF0713 protein YngL
**contig_1**	cds	203886	204237	-	GLNHAC_02308		amino acid adenylation domain-containing protein
**contig_1**	cds	204237	204634	-	GLNHAC_02309		non-ribosomal peptide synthetase
**contig_1**	cds	204656	204768	-	GLNHAC_02310		hypothetical protein
**contig_1**	cds	204768	204800	-	GLNHAC_02311		condensation domain-containing protein
**contig_1**	cds	204823	204989	-	GLNHAC_02312		hypothetical protein
**contig_1**	cds	205032	205146	-	GLNHAC_02313		hypothetical protein
**contig_1**	cds	205155	205724	-	GLNHAC_02314		Non-ribosomal peptide synthetase
**contig_1**	cds	205724	205908	-	GLNHAC_02315		Non-ribosomal peptide synthetase
**contig_1**	cds	205912	206286	-	GLNHAC_02316		non-ribosomal peptide synthetase
**contig_1**	cds	206292	206727	-	GLNHAC_02317		amino acid adenylation domain-containing protein
**contig_1**	cds	206785	206974	-	GLNHAC_02318		hypothetical protein
**contig_1**	cds	206989	207483	-	GLNHAC_02319		aminotransferase class III-fold pyridoxal phosphate-dependen
**contig_1**	cds	207485	207605	-	GLNHAC_02320	fabD	ACP S-malonyltransferase

contig_1	cds	207702	207849	-		GLNHAC_02321	dacB	D-alanyl-D-alanine carboxypeptidase/D-alanyl-D-alanine-end
contig_1	cds	207874	207899	-		GLNHAC_02322		hypothetical protein
contig_1	cds	207893	207950	-		GLNHAC_02323		(pseudo) Aldose 1-epimerase
contig_1	cds	207963	208103	-		GLNHAC_02324	yoeA	putative multidrug resistance protein YoeA
contig_1	cds	208131	208186	+		GLNHAC_02325	yoeB	DL-endopeptidase inhibitor IseA
contig_1	tRNA	208195	208202	-		GLNHAC_02326	trnR	tRNA-Arg(cct)
contig_1	cds	208207	208262	-		GLNHAC_02327	yoeC	putative integrase/recombinase YoeC
contig_1	cds	208293	208316	-		GLNHAC_02328	yoeD	Uncharacterized protein YoeD
contig_1	cds	208335	208511	+		GLNHAC_02329	ggt	gamma-glutamyltransferase
contig_1	cds	208544	208615	-		GLNHAC_02330	lysR	LysR family transcriptional regulator
contig_1	cds	208628	208727	+		GLNHAC_02331	yogA	YogA
contig_1	cds	208733	208769	-		GLNHAC_02332		hypothetical protein
contig_1	cds	208767	208881	-		GLNHAC_02333		Glutamate synthase
contig_1	cds	208882	209308	-		GLNHAC_02334	gltB	glutamate synthase large subunit
contig_1	cds	209304	209339	-		GLNHAC_02335		Glutamine amidotransferase type-2 domain-containing protei
contig_1	cds	209353	209443	+		GLNHAC_02336	gltC	glutamate biosynthesis transcriptional regulator GltC
contig_1	cds	209449	209560	-		GLNHAC_02337	proJ	Glutamate 5-kinase 2
contig_1	cds	209560	209641	-		GLNHAC_02338	proC	pyrroline-5-carboxylate reductase
contig_1	cds	209664	209701	-		GLNHAC_02339	rtp	replication termination protein
contig_1	cds	209731	209853	+		GLNHAC_02340	cyp109	Cytochrome P450 109
contig_1	cds	209856	209877	-		GLNHAC_02341		(pseudo) SDR family NAD(P)-dependent oxidoreductase
contig_1	cds	209905	209927	-		GLNHAC_02342		SDR family NAD(P)-dependent oxidoreductase
contig_1	cds	209942	209973	+		GLNHAC_02343		DUF948 domain-containing protein
contig_1	cds	209980	210030	+		GLNHAC_02344		(pseudo) Uracil DNA glycosylase superfamily protein
contig_1	cds	210061	210115	+		GLNHAC_02345		GNAT family N-acetyltransferase
contig_1	cds	210133	210232	-		GLNHAC_02346		ABC-2 type transporter transmembrane domain-containing p
contig_1	cds	210232	210306	-		GLNHAC_02347		ABC transporter permease
contig_1	cds	210307	210401	-		GLNHAC_02348		ABC-type MDR transporter, ATPase component
contig_1	cds	210402	210457	-		GLNHAC_02349		Transcriptional regulator, MarR/EmrR family
contig_1	cds	210502	210626	-		GLNHAC_02350	yoaB	Putative transporter YoaB
contig_1	cds	210635	210782	-		GLNHAC_02351	yoaC	Putative sugar kinase YoaC
contig_1	cds	210784	210884	-		GLNHAC_02352	yoaD	Putative 2-hydroxyacid dehydrogenase YoaD
contig_1	ncRNA-region	210895	210905	-				SAM riboswitch aptamer (S box leader)
contig_1	cds	210919	211124	+		GLNHAC_02353	yoaE	putative oxidoreductase YoaE
contig_1	cds	211134	211164	+		GLNHAC_02354	yoaF	YoaF
contig_1	cds	211182	211194	-		GLNHAC_02355		(pseudo) ABC transporter ATP-binding protein
contig_1	cds	211201	211232	-		GLNHAC_02356		UPF0715 family protein
contig_1	cds	211271	211283	+		GLNHAC_02357		Biofilm-forming protein
contig_1	cds	211289	211318	+		GLNHAC_02358		DUF4025 domain-containing protein
contig_1	cds	211333	211502	-		GLNHAC_02359	yoaH	Putative methyl-accepting chemotaxis protein YoaH
contig_1	cds	211533	211618	+		GLNHAC_02360		4-hydroxyphenylacetate 3-monooxygenase
contig_1	cds	211618	211674	+		GLNHAC_02361		hypothetical protein
contig_1	cds	211685	211754	-		GLNHAC_02362	yoaJ	expansin ExlX
contig_1	cds	211781	211848	-		GLNHAC_02363	yoaK	UPF0700 transmembrane protein YoaK
contig_1	cds	211866	211969	+		GLNHAC_02364	pelB	Pectin lyase
contig_1	cds	211994	212071	-		GLNHAC_02365		Diacetyl reductase [(S)-acetoin forming]
contig_1	cds	212096	212139	-		GLNHAC_02366	yoaW	YoaW
contig_1	cds	212154	212226	-		GLNHAC_02367		Necrosis and ethylene inducing protein
contig_1	cds	212252	212320	+		GLNHAC_02368		Abasic site processing protein
contig_1	cds	212344	212354	-		GLNHAC_02369		hypothetical protein
contig_1	cds	212359	212389	-		GLNHAC_02370		Permease
contig_1	cds	212417	212534	-		GLNHAC_02371	oxdD	Oxalate decarboxylase OxdD
contig_1	cds	212547	212596	-		GLNHAC_02372		DUF4944 domain-containing protein
contig_1	cds	212629	212648	-		GLNHAC_02373		Transcriptional regulator
contig_1	cds	212662	212711	-		GLNHAC_02374	yozF	Uncharacterized protein YozF
contig_1	cds	212716	212808	-		GLNHAC_02375	yoaR	Uncharacterized protein YoaR
contig_1	cds	212846	212894	+		GLNHAC_02376		DUF2975 domain-containing protein
contig_1	cds	212895	212917	+		GLNHAC_02377		HTH cro/C1-type domain-containing protein
contig_1	cds	212930	213009	+		GLNHAC_02378		Putative integral inner membrane protein
contig_1	cds	213033	213103	-		GLNHAC_02379	lysR	LysR substrate-binding domain-containing protein
contig_1	cds	213130	213218	+		GLNHAC_02380	yoaV	Uncharacterized transporter YoaV
contig_1	cds	213252	213269	-		GLNHAC_02381	yqzM	YqzM family protein
contig_1	cds	213277	213305	-		GLNHAC_02382		hypothetical protein
contig_1	cds	213374	213463	-		GLNHAC_02383		CPBP family intramembrane metalloprotease
contig_1	cds	213487	213550	-		GLNHAC_02384		Putative factor of the oxidative stress response
contig_1	cds	213585	213676	+		GLNHAC_02385	bla	class A beta-lactamase
contig_1	cds	213695	213709	-		GLNHAC_02386		(pseudo) Knr4/Smi1-like domain-containing protein
contig_1	cds	213758	213820	-		GLNHAC_02387		Chitin binding protein
contig_1	cds	213892	213942	+		GLNHAC_02388	yrdA	Uncharacterized protein YrdA
contig_1	cds	214035	214088	-		GLNHAC_02389		GNAT family N-acetyltransferase
contig_1	cds	214097	214161	-		GLNHAC_02390	xynA	endo-1,4-beta-xylanase XynA
contig_1	cds	214214	214244	+		GLNHAC_02391		Response regulator aspartate phosphatase C
contig_1	cds	214241	214254	+		GLNHAC_02392		hypothetical protein
contig_1	cds	214265	214338	+		GLNHAC_02393	bglS	beta-glucanase
contig_1	cds	214419	214517	-		GLNHAC_02394		cytochrome P450
contig_1	cds	214524	214543	-		GLNHAC_02395		hypothetical protein
contig_1	cds	214556	214588	-		GLNHAC_02396		N-acetyltransferase
contig_1	cds	214646	214663	+		GLNHAC_02397		TetR-like C-terminal domain-containing protein
contig_1	cds	214728	214787	-		GLNHAC_02398	pdxT	pyridoxal 5'-phosphate synthase glutaminase subunit PdxT
contig_1	cds	214788	214876	-		GLNHAC_02399	pdxS	pyridoxal 5'-phosphate synthase lyase subunit PdxS
contig_1	cds	214889	215029	+		GLNHAC_02400	gntR	Transcriptional regulator, GntR family
contig_1	cds	215039	215061	-		GLNHAC_02401		cupin domain-containing protein
contig_1	cds	215079	215112	+		GLNHAC_02402		hypothetical protein
contig_1	cds	215159	215195	+		GLNHAC_02403		GH32 C-terminal domain-containing protein
contig_1	cds	215191	215292	+		GLNHAC_02404		Fructokinase
contig_1	cds	215317	215373	+		GLNHAC_02405		Acetyltransferase
contig_1	cds	215421	215444	+		GLNHAC_02406		DUF3953 domain-containing protein
contig_1	cds	215445	215484	-		GLNHAC_02407	yopD	SPbeta prophage-derived UPF0715 membrane protein YopD
contig_1	cds	215613	215631	-		GLNHAC_02408		hypothetical protein
contig_1	cds	215681	215824	+		GLNHAC_02409		Amine oxidase, flavin-containing protein
contig_1	ncRNA	215838	215849	+		GLNHAC_02410	bsrF	BsrF
contig_1	cds	215850	215884	-		GLNHAC_02411	csaA	chaperone CsaA
contig_1	cds	215890	215962	-		GLNHAC_02412	yobQ	Uncharacterized HTH-type transcriptional regulator YobQ
contig_1	cds	215964	216038	-		GLNHAC_02413		GNAT family N-acetyltransferase
contig_1	cds	216046	216104	-		GLNHAC_02414		HTH tetR-type domain-containing protein
contig_1	cds	216104	216174	-		GLNHAC_02415	yobT	Uncharacterized protein YobT
contig_1	cds	216182	216230	-		GLNHAC_02416		Regulatory protein
contig_1	cds	216235	216329	-		GLNHAC_02417	yobV	Uncharacterized HTH-type transcriptional regulator YobV
contig_1	cds	216349	216393	+		GLNHAC_02418		Protein csk22
contig_1	cds	216406	216433	-		GLNHAC_02419	czrA	HTH-type transcriptional repressor CzrA
contig_1	cds	216457	216502	+		GLNHAC_02420		hypothetical protein
contig_1	cds	216512	216525	+		GLNHAC_02421		(pseudo) lysozyme family protein
contig_1	cds	216534	216588	-		GLNHAC_02422	yozB	Uncharacterized membrane protein YozB
contig_1	cds	216602	216680	-		GLNHAC_02423	yocB	Uncharacterized protein YocB
contig_1	cds	216697	216747	+		GLNHAC_02424	yocC	Uncharacterized protein YocC
contig_1	cds	216749	216846	+		GLNHAC_02425	yocD	Putative carboxypeptidase YocD
contig_1	cds	216868	216973	+		GLNHAC_02426	desE	fatty acid desaturase DesE
contig_1	cds	216986	217097	+		GLNHAC_02427	desK	two-component sensor histidine kinase DesK
contig_1	cds	217099	217159	+		GLNHAC_02428	desR	two-component system response regulator DesR
contig_1	cds	217162	217321	-		GLNHAC_02429		solute:sodium symporter family transporter
contig_1	cds	217340	217426	-		GLNHAC_02430	yocH	Cell wall-binding protein YocH
contig_1	cds	217451	217628	-		GLNHAC_02431	recQ	DNA helicase RecQ
contig_1	ncRNA	217642	217661	-		GLNHAC_02432	6S	6S / SsrS RNA
contig_1	cds	217684	217707	-		GLNHAC_02433		Flavodoxin-like fold domain-containing protein
contig_1	cds	217722	217747	-		GLNHAC_02434		Flavodoxin-like fold domain-containing protein
contig_1	cds	217762	217811	-		GLNHAC_02435	yocK	General stress protein 16O
contig_1	cds	217836	217871	-		GLNHAC_02436	yozO	Uncharacterized protein YozO
contig_1	cds	217904	217924	-		GLNHAC_02437	yozC	Uncharacterized protein YozC
contig_1	cds	217947	218096	+		GLNHAC_02438	dhaS	aldehyde dehydrogenase DhaS
contig_1	cds	218106	218296	+		GLNHAC_02439	sqhC	Sporulenol synthase
contig_1	cds	218295	218379	+		GLNHAC_02440	sodF	putative superoxide dismutase [Fe]
contig_1	cds	218383	218516	-		GLNHAC_02441	yocR	Uncharacterized sodium-dependent transporter YocR
contig_1	cds	218544	218609	+		GLNHAC_02442		Sodium bile acid symporter family protein
contig_1	cds	218641	218766	-		GLNHAC_02443	odhB	2-oxoglutarate dehydrogenase complex dihydrolipoyllysine-re
contig_1	cds	218767	219051	-		GLNHAC_02444	sucA	2-oxoglutarate dehydrogenase E1 component
contig_1	cds	219074	219265	-		GLNHAC_02445	yojO	Uncharacterized protein YojO
contig_1	cds	219266	219355	-		GLNHAC_02446	yojN	Uncharacterized protein YojN
contig_1	cds	219364	219423	-		GLNHAC_02447	yojM	Superoxide dismutase-like protein YojM
contig_1	cds	219433	219556	-		GLNHAC_02448	cwlS	SAG1386/EF1546 family surface-associated protein
contig_1	cds	219594	219716	-		GLNHAC_02449	yojK	Uncharacterized UDP-glucosyltransferase YojK
contig_1	cds	219740	219801	-		GLNHAC_02450	cdaS	cyclic di-AMP synthase CdaS
contig_1	cds	219810	219821	+		GLNHAC_02451		Phage protein
contig_1	cds	219828	219964	+		GLNHAC_02452	norM	putative multidrug resistance protein NorM
contig_1	cds	219966	220051	+		GLNHAC_02453	rsbRC	RsbT co-antagonist RsbRC
contig_1	cds	220053	220120	-		GLNHAC_02454	bshB2	bacillithiol biosynthesis deacetylase BshB2
contig_1	cds	220122	220157	-		GLNHAC_02455	yojF	Uncharacterized protein YojF
contig_1	cds	220156	220184	-		GLNHAC_02456	yoyC	Uncharacterized protein YoyC
contig_1	cds	220192	220281	-		GLNHAC_02457	rarD	EamA family transporter RarD
contig_1	cds	220291	220337	+		GLNHAC_02458	gerT	Spore gernimation protein GerT

contig_1	cds	220341	220364	-	GLNHAC_02459		Spore coat protein
contig_1	cds	220373	220447	-	GLNHAC_02460		Gluconate permease
contig_1	cds	220447	220506	-	GLNHAC_02461		Gluconate permease
contig_1	cds	220542	220581	+	GLNHAC_02462		Tautomerase family protein
contig_1	cds	220613	220646	-	GLNHAC_02463	yodB	transcriptional regulator YodB
contig_1	cds	220660	220721	+	GLNHAC_02464	yodC	Putative NAD(P)H nitroreductase YodC
contig_1	cds	220725	220786	-	GLNHAC_02465	mhqD	Putative hydrolase MhqD
contig_1	cds	220787	220833	-	GLNHAC_02466	mhqE	(pseudo) Putative ring-cleaving dioxygenase MhqE
contig_1	cds	220833	220879	-	GLNHAC_02467	mhqE	(pseudo) Putative ring-cleaving dioxygenase MhqE
contig_1	cds	220898	220918	+	GLNHAC_02468	yoyD	Uncharacterized membrane protein YoyD
contig_1	cds	220957	221066	+	GLNHAC_02469	yodF	(pseudo) Uncharacterized symporter YodF
contig_1	cds	221070	221210	-	GLNHAC_02470	ctpA	carboxy-terminal processing protease CtpA
contig_1	cds	221230	221295	+	GLNHAC_02471	yodH	Uncharacterized methyltransferase YodH
contig_1	cds	221304	221329	+	GLNHAC_02472	yodI	Uncharacterized protein YodI
contig_1	cds	221337	221419	-	GLNHAC_02473	yodJ	LD-carboxypeptidase LdcB
contig_1	cds	221428	221498	-	GLNHAC_02474	deoD	purine-nucleoside phosphorylase
contig_1	cds	221518	221530	+	GLNHAC_02475	yoyE	Uncharacterized protein YoyE
contig_1	cds	221534	221566	-	GLNHAC_02476	yodL	shape determination protein YodL
contig_1	cds	221572	221633	-	GLNHAC_02477	pgpB	Phosphatidylglycerophosphatase PgpB
contig_1	cds	221641	221658	-	GLNHAC_02478	yozD	Uncharacterized protein YozD
contig_1	cds	221671	221685	+	GLNHAC_02479	yoyF	Uncharacterized membrane protein YoyF
contig_1	cds	221722	221753	-	GLNHAC_02480	yodN	(pseudo) Uncharacterized protein YodN
contig_1	cds	221768	221790	-	GLNHAC_02481	yozE	UPF0346 protein YozE
contig_1	cds	221799	221825	-	GLNHAC_02482	yokU	Uncharacterized protein YokU
contig_1	cds	221833	221967	-	GLNHAC_02483	ablA	lysine 2,3-aminomutase
contig_1	cds	221970	222053	-	GLNHAC_02484	ablB	putative beta-lysine N-acetyltransferase
contig_1	cds	222051	222181	-	GLNHAC_02485	yodQ	peptidase
contig_1	cds	222182	222241	-	GLNHAC_02486	yodR	putative coenzyme A transferase subunit beta
contig_1	cds	222254	222304	-	GLNHAC_02487		Acetate CoA-transferase subunit alpha
contig_1	cds	222304	222315	-	GLNHAC_02488		hypothetical protein
contig_1	cds	222316	222449	-	GLNHAC_02489	yodT	aspartate aminotransferase family protein
contig_1	cds	222481	222559	-	GLNHAC_02490	cgeE	spore maturation N-acetyltransferase CgeE
contig_1	cds	222562	222690	-	GLNHAC_02491	cgeD	Sporulation putative glycosyl transferase CgeD
contig_1	cds	222697	222727	-	GLNHAC_02492	cgeC	spore maturation protein CgeC
contig_1	cds	222748	222787	+	GLNHAC_02493	cgeA	Sporulation protein CgeA
contig_1	cds	222788	222883	+	GLNHAC_02494	cgeB	Sporulation protein CgeB
contig_1	cds	222890	223005	-	GLNHAC_02495	phy	3-phytase
contig_1	cds	223042	223144	+	GLNHAC_02496		Capsular polysaccharide biosynthesis protein D
contig_1	cds	223149	223192	-	GLNHAC_02497	msrB	Peptide methionine sulfoxide reductase MsrB
contig_1	cds	223191	223245	-	GLNHAC_02498	msrA	Peptide methionine sulfoxide reductase MsrA
contig_1	cds	223259	223308	+	GLNHAC_02499	ypoP	Uncharacterized HTH-type transcriptional regulator YpoP
contig_1	cds	223306	223334	-	GLNHAC_02500		hypothetical protein
contig_1	cds	223330	223369	-	GLNHAC_02501		hypothetical protein
contig_1	cds	223370	223440	-	GLNHAC_02502		MATE family efflux transporter
contig_1	cds	223447	223467	-	GLNHAC_02503	ypmT	Protein YpmT
contig_1	cds	223473	223524	-	GLNHAC_02504	ypmS	Uncharacterized protein YpmS
contig_1	cds	223525	223602	-	GLNHAC_02505	ypmR	Uncharacterized protein YpmR
contig_1	cds	223610	223668	-	GLNHAC_02506	ypmQ	cytochrome c oxidase assembly accessory protein ScuA
contig_1	cds	223683	223708	-	GLNHAC_02507	ypmP	Uncharacterized protein YpmP
contig_1	cds	223717	223844	-	GLNHAC_02508	ilvA	threonine ammonia-lyase IlvA
contig_1	cds	223869	223968	+	GLNHAC_02509		Sigma 54-interacting transcriptional regulator
contig_1	cds	223970	224034	+	GLNHAC_02510	yplQ	Hemolysin-3-like protein
contig_1	cds	224038	224100	-	GLNHAC_02511	ypkP	Uncharacterized protein YpkP
contig_1	cds	224100	224150	-	GLNHAC_02512	dfrA	dihydrofolate reductase DfrA
contig_1	cds	224150	224229	-	GLNHAC_02513	thyA	thymidylate synthase
contig_1	cds	224238	224291	-	GLNHAC_02514	ypjQ	Uncharacterized protein YpjQ
contig_1	cds	224293	224354	-	GLNHAC_02515	ypjP	Uncharacterized protein YpjP
contig_1	cds	224380	224459	-	GLNHAC_02516	ypiP	Uncharacterized protein YpiP
contig_1	cds	224463	224506	-	GLNHAC_02517	brxA	bacilliredoxin BrxA
contig_1	cds	224517	224645	-	GLNHAC_02518		Dihydroxy-acid dehydratase
contig_1	cds	224713	224827	-	GLNHAC_02519	ypgR	Uncharacterized protein YpgR
contig_1	cds	224833	224894	-	GLNHAC_02520	ypgQ	YpgQ
contig_1	cds	224896	224944	-	GLNHAC_02521	bsaA	Glutathione peroxidase-like protein BsaA
contig_1	cds	224980	225070	+	GLNHAC_02522	metA	homoserine O-succinyltransferase
contig_1	cds	225093	225208	+	GLNHAC_02523	ugtP	diglucosyl diacylglycerol synthase
contig_1	cds	225232	225252	+	GLNHAC_02524	cspD	cold-shock protein CspD
contig_1	cds	225257	225276	-	GLNHAC_02525	degR	transcriptional regulator DegR
contig_1	cds	225291	225318	+	GLNHAC_02526	ypzA	Uncharacterized protein YpzA
contig_1	cds	225321	225338	-	GLNHAC_02527	ypeQ	Uncharacterized protein YpeQ
contig_1	cds	225339	225419	-	GLNHAC_02528	ypeP	Uncharacterized protein YpeP
contig_1	cds	225423	225484	+	GLNHAC_02529	ypdP	queuosine precursor transporter
contig_1	cds	225484	225529	+	GLNHAC_02530	rnhA	14-7 kDa ribonuclease H-like protein
contig_1	cds	225528	225540	+	GLNHAC_02531	sspL	small, acid-soluble spore protein L
contig_1	cds	225564	225598	-	GLNHAC_02532		(pseudo) 5'-3' exonuclease
contig_1	cds	225600	225630	-	GLNHAC_02533		5'-3' exonuclease
contig_1	cds	225640	225655	-	GLNHAC_02534	ypzF	Uncharacterized protein YpzF
contig_1	cds	225662	225688	-	GLNHAC_02535	ypbS	Uncharacterized protein YpbS
contig_1	cds	225694	226053	-	GLNHAC_02536		dynamin family protein
contig_1	sorf	226070	226079	-	GLNHAC_02537	fbpC	Fur-regulated basic protein FbpC
contig_1	cds	226086	226137	-	GLNHAC_02538	ypbQ	Uncharacterized protein YpbQ
contig_1	cds	226137	226247	-	GLNHAC_02539		Putative chalcone synthase
contig_1	cds	226254	226303	-	GLNHAC_02540		(pseudo) solute carrier family 23 protein
contig_1	cds	226304	226335	-	GLNHAC_02541		hypothetical protein
contig_1	cds	226338	226386	-	GLNHAC_02542		Xanthine permease
contig_1	cds	226385	226444	-	GLNHAC_02543	xpt	xanthine phosphoribosyltransferase
contig_1	ncRNA-region	226453	226463	-			Purine riboswitch aptamer
contig_1	cds	226477	226628	-	GLNHAC_02544	ypwA	carboxypeptidase
contig_1	cds	226674	226738	-	GLNHAC_02545	kdgT	(pseudo) 2-keto-3-deoxygluconate transporter
contig_1	cds	226742	226801	-	GLNHAC_02546	kdgA	2-dehydro-3-deoxy-phosphogluconate aldolase
contig_1	cds	226807	226899	-	GLNHAC_02547	kdgK	2-dehydro-3-deoxygluconokinase
contig_1	cds	226903	226970	-	GLNHAC_02548	kdgR	(pseudo) HTH-type transcriptional regulator KdgR
contig_1	cds	226969	227004	-	GLNHAC_02549		hypothetical protein
contig_1	cds	227027	227109	+	GLNHAC_02550	kduI	5-dehydro-4-deoxy-D-glucuronate isomerase
contig_1	cds	227109	227186	+	GLNHAC_02551	kduD	2-dehydro-3-deoxy-D-gluconate 5-dehydrogenase KduD
contig_1	cds	227190	227382	-	GLNHAC_02552	ypvA	putative ATP-dependent helicase YpvA
contig_1	cds	227393	227412	-	GLNHAC_02553	yptA	YptA
contig_1	cds	227428	227443	+	GLNHAC_02554	ypzG	Uncharacterized protein YpzG
contig_1	cds	227449	227565	-	GLNHAC_02555	ypsC	YpsC
contig_1	ncRNA	227575	227613	-	GLNHAC_02556	RNaseP_bact_b	Bacterial RNase P class B
contig_1	cds	227619	227645	-	GLNHAC_02557	gpsB	cell division regulator GpsB
contig_1	cds	227656	227711	-	GLNHAC_02558	ypsA	UPF0398 protein YpsA
contig_1	cds	227773	227898	-	GLNHAC_02559	yprB	Uncharacterized protein YprB
contig_1	cds	227900	228125	-	GLNHAC_02560	yprA	ATP-dependent helicase MrfA
contig_1	cds	228135	228191	-	GLNHAC_02561	ptsA	Phosphotransferase enzyme IIA component PtsA
contig_1	cds	228200	228242	+	GLNHAC_02562		Hsp20/alpha crystallin family protein
contig_1	cds	228244	228280	-	GLNHAC_02563	yppG	Uncharacterized protein YppG
contig_1	cds	228299	228318	+	GLNHAC_02564	yppF	Uncharacterized protein YppF
contig_1	cds	228322	228359	-	GLNHAC_02565	yppE	Uncharacterized protein YppE
contig_1	cds	228364	228388	-	GLNHAC_02566	yppD	Uncharacterized protein YppD
contig_1	cds	228408	228418	+	GLNHAC_02567	sspM	acid-soluble spore protein SspM
contig_1	cds	228421	228480	-	GLNHAC_02568		DUF2515 domain-containing protein
contig_1	cds	228479	228517	-	GLNHAC_02569		DUF2515 domain-containing protein
contig_1	cds	228521	228583	+	GLNHAC_02570	recU	Holliday junction resolvase RecU
contig_1	cds	228585	228861	+	GLNHAC_02571	ponA	Penicillin-binding protein 1A/1B
contig_1	cds	228868	228917	-	GLNHAC_02572	ypoC	Uncharacterized protein YpoC
contig_1	cds	228917	228983	-	GLNHAC_02573	nth	endonuclease III
contig_1	cds	228985	229055	-	GLNHAC_02574	dnaD	DNA replication protein DnaD
contig_1	cds	229064	229194	-	GLNHAC_02575	asnS	asparagine--tRNA ligase
contig_1	cds	229208	229228	-	GLNHAC_02576		aminotransferase class I/II-fold pyridoxal phosphate-depende
contig_1	cds	229223	229326	-	GLNHAC_02577	aspB	Aspartate aminotransferase
contig_1	cds	229328	229377	-	GLNHAC_02578	tseB	cell wall elongation/penicillin-binding protein regulator TseB
contig_1	cds	229378	229395	-	GLNHAC_02579	ypmA	Uncharacterized protein YpmA
contig_1	cds	229409	229695	-	GLNHAC_02580	dinG	ATP-dependent DNA helicase DinG
contig_1	cds	229701	229739	-	GLNHAC_02581	panD	aspartate 1-decarboxylase
contig_1	cds	229739	229825	-	GLNHAC_02582	panC	pantoate--beta-alanine ligase
contig_1	cds	229826	229909	-	GLNHAC_02583	panB	3-methyl-2-oxobutanoate hydroxymethyltransferase
contig_1	ncRNA-region	229920	229929	-			pan motif
contig_1	cds	229933	229990	-	GLNHAC_02584		biotin--[acetyl-CoA-carboxylase] ligase
contig_1	cds	229989	230149	-	GLNHAC_02585		hypothetical protein
contig_1	cds	230149	230263	-	GLNHAC_02586	bshA	N-acetyl-alpha-D-glucosaminyl L-malate synthase BshA
contig_1	cds	230262	230328	-	GLNHAC_02587	bshB1	bacillithiol biosynthesis deacetylase BshB1
contig_1	cds	230323	230374	-	GLNHAC_02588		Methylglyoxal synthase
contig_1	cds	230376	230456	-	GLNHAC_02589	dapB	4-hydroxy-tetrahydrodipicolinate reductase
contig_1	cds	230457	230491	-	GLNHAC_02590		NTP pyrophosphohydrolase MazG-like domain-containing pro
contig_1	cds	230505	230592	+	GLNHAC_02591	ypjC	UPF0750 membrane protein YpjC
contig_1	cds	230596	230675	-	GLNHAC_02592	ypjB	sporulation protein YpjB
contig_1	cds	230682	230738	-	GLNHAC_02593	ypjA	Uncharacterized protein YpjA
contig_1	cds	230752	230776	-	GLNHAC_02594		Cytochrome c
contig_1	cds	230776	230829	-	GLNHAC_02595	qcrC	(pseudo) Menaquinol:cytochrome c reductase cytochrome c s

contig_1	cds	230833	230869	-	GLNHAC_02596		Menaquinol-cytochrome c reductase cytochrome b subunit
contig_1	cds	230865	230900	-	GLNHAC_02597		Menaquinol-cytochrome c reductase cytochrome b subunit
contig_1	cds	230900	230951	-	GLNHAC_02598	qcrA	Menaquinol:cytochrome c reductase iron-sulfur subunit
contig_1	cds	230965	231009	-	GLNHAC_02599	ypiF	Uncharacterized protein YpiF
contig_1	cds	231015	231069	-	GLNHAC_02600	ypiB	ReoY family proteolytic degradation factor
contig_1	cds	231076	231109	-	GLNHAC_02601	lapB	(pseudo) lipopolysaccharide assembly protein LapB
contig_1	cds	231113	231203	-	GLNHAC_02602	lapB	lipopolysaccharide assembly protein LapB
contig_1	cds	231316	231366	-	GLNHAC_02603		3-phosphoshikimate 1-carboxyvinyltransferase
contig_1	cds	231367	231479	-	GLNHAC_02604	tyrA	prephenate dehydrogenase
contig_1	cds	231484	231549	-	GLNHAC_02605		aminotransferase class I/II-fold pyridoxal phosphate-depende
contig_1	cds	231561	231592	-	GLNHAC_02606		aminotransferase class I/II-fold pyridoxal phosphate-depende
contig_1	cds	231593	231673	-	GLNHAC_02607	trpA	tryptophan synthase subunit alpha
contig_1	cds	231672	231793	-	GLNHAC_02608	trpB	tryptophan synthase subunit beta
contig_1	cds	231791	231931	-	GLNHAC_02609		hypothetical protein
contig_1	cds	231930	232032	-	GLNHAC_02610	trpD	Anthranilate phosphoribosyltransferase
contig_1	cds	232029	232184	-	GLNHAC_02611	trpE	anthranilate synthase component I
contig_1	ncRNA-region	232190	232204	-			Bacillus tryptophan operon leader
contig_1	cds	232205	232244	-	GLNHAC_02612	aroH	chorismate mutase
contig_1	cds	232243	232352	-	GLNHAC_02613	aroB	3-dehydroquinate synthase
contig_1	cds	232352	232469	-	GLNHAC_02614	aroC	chorismate synthase
contig_1	cds	232477	232554	-	GLNHAC_02615	cheR	protein-glutamate O-methyltransferase CheR
contig_1	cds	232578	232622	-	GLNHAC_02616	ndk	nucleoside-diphosphate kinase
contig_1	cds	232634	232739	-	GLNHAC_02617	hepT	heptaprenyl diphosphate synthase component II
contig_1	cds	232733	232803	-	GLNHAC_02618	menG	demethylmenaquinone methyltransferase
contig_1	cds	232804	232880	-	GLNHAC_02619	hepS	heptaprenyl diphosphate synthase component 1
contig_1	cds	232896	232919	-	GLNHAC_02620	mtrB	trp RNA-binding attenuation protein MtrB
contig_1	cds	232921	232978	-	GLNHAC_02621	folE	GTP cyclohydrolase I FolE
contig_1	cds	232997	233025	-	GLNHAC_02622	hbs	non-specific DNA-binding protein Hbs
contig_1	cds	233062	233210	-	GLNHAC_02623	spoIVA	stage IV sporulation protein A
contig_1	cds	233229	233302	-	GLNHAC_02624	yphF	Uncharacterized protein YphF
contig_1	cds	233304	233324	-	GLNHAC_02625	yphE	Uncharacterized protein YphE
contig_1	cds	233358	233460	-	GLNHAC_02626	gpsA	NAD(P)H-dependent glycerol-3-phosphate dehydrogenase
contig_1	cds	233463	233594	-	GLNHAC_02627	engA	ribosome-associated GTPase EngA
contig_1	cds	233610	233629	-	GLNHAC_02628	ypzH	Uncharacterized protein YpzH
contig_1	cds	233629	233718	-	GLNHAC_02629	yphB	Uncharacterized protein YphB
contig_1	cds	233718	233778	-	GLNHAC_02630	yphA	Uncharacterized protein YphA
contig_1	cds	233786	233799	+	GLNHAC_02631	ypzI	Uncharacterized protein YpzI
contig_1	cds	233803	233908	-	GLNHAC_02632	fni	type 2 isopentenyl-diphosphate Delta-isomerase
contig_1	cds	233909	234024	-	GLNHAC_02633	rpsA	30S ribosomal protein S1
contig_1	cds	234048	234115	-	GLNHAC_02634	cmk	(d)CMP kinase
contig_1	cds	234120	234140	-	GLNHAC_02635	ypfB	Uncharacterized protein YpfB
contig_1	cds	234145	234210	-	GLNHAC_02636		Flagellar brake protein
contig_1	cds	234220	234355	-	GLNHAC_02637	ypeB	germination protein YpeB
contig_1	cds	234358	234449	-	GLNHAC_02638	sleB	spore cortex-lytic enzyme
contig_1	cds	234465	234530	-	GLNHAC_02639	prsW	glutamic-type intramembrane protease PrsW
contig_1	cds	234542	234640	-	GLNHAC_02640	bdr	Bacilliredoxin reductase Bdr
contig_1	cds	234651	234748	-	GLNHAC_02641	rocG	Catabolic NAD-specific glutamate dehydrogenase RocG
contig_1	cds	234751	234778	-	GLNHAC_02642		Glutamate dehydrogenase
contig_1	cds	234794	234852	-	GLNHAC_02643		genetic competence negative regulator
contig_1	cds	234868	234946	-	GLNHAC_02644	ypbG	Uncharacterized protein YpbG
contig_1	cds	234955	234997	-	GLNHAC_02645	ypbF	YpbF
contig_1	cds	235005	235080	-	GLNHAC_02646	lysM	LysM domain-containing protein
contig_1	cds	235075	235136	-	GLNHAC_02647	ypbD	Uncharacterized protein YpbD
contig_1	cds	235139	235287	-	GLNHAC_02648	recS	putative ATP-dependent DNA helicase RecS
contig_1	cds	235286	235393	-	GLNHAC_02649	ypbB	Protein YpbB
contig_1	cds	235419	235444	+	GLNHAC_02650	fer	Ferredoxin
contig_1	cds	235447	235504	-	GLNHAC_02651	fmnP	riboflavin transporter FmnP
contig_1	cds	235507	235533	-	GLNHAC_02652		hypothetical protein
contig_1	ncRNA-region	235519	235533	-			FMN riboswitch aptamer (RFN element)
contig_1	cds	235554	235654	+	GLNHAC_02653		D-3-phosphoglycerate dehydrogenase
contig_1	cds	235677	235701	+	GLNHAC_02654		hypothetical protein
contig_1	cds	235719	235791	-	GLNHAC_02655		3-dehydroquinate dehydratase
contig_1	cds	235802	235913	-	GLNHAC_02656		sigma-X negative effector
contig_1	cds	235907	235965	-	GLNHAC_02657	sigX	RNA polymerase sigma factor SigX
contig_1	cds	235985	236107	-	GLNHAC_02658	resE	sensor histidine kinase ResE
contig_1	cds	236107	236162	-	GLNHAC_02659		hypothetical protein
contig_1	cds	236162	236234	-	GLNHAC_02660	resD	DNA-binding response regulator ResD
contig_1	cds	236242	236306	-	GLNHAC_02661	ccsA	cytochrome c biogenesis protein CcsA
contig_1	cds	236322	236360	-	GLNHAC_02662		hypothetical protein
contig_1	cds	236362	236525	-	GLNHAC_02663	resB	cytochrome c biogenesis protein ResB
contig_1	cds	236524	236578	-	GLNHAC_02664	resA	thiol-disulfide oxidoreductase ResA
contig_1	cds	236588	236603	-	GLNHAC_02665		hypothetical protein
contig_1	cds	236606	236661	-	GLNHAC_02666		Ribosomal large subunit pseudouridine synthase B
contig_1	cds	236670	236724	-	GLNHAC_02667	spmB	spore maturation protein SpmB
contig_1	cds	236724	236783	-	GLNHAC_02668	spmA	spore maturation protein SpmA
contig_1	cds	236782	236897	-	GLNHAC_02669	dacB	D-alanyl-D-alanine carboxypeptidase DacB
contig_1	cds	236909	236963	-	GLNHAC_02670	ypuI	Uncharacterized protein YpuI
contig_1	cds	236969	237028	-	GLNHAC_02671	scpB	SMC-Scp complex subunit ScpB
contig_1	cds	237027	237105	-	GLNHAC_02672	scpA	segregation/condensation protein A
contig_1	cds	237133	237185	+	GLNHAC_02673		DUF309 domain-containing protein
contig_1	cds	237187	237224	-	GLNHAC_02674	ribT	GNAT family N-acetyltransferase RibT
contig_1	cds	237235	237282	-	GLNHAC_02675	ribH	6,7-dimethyl-8-ribityllumazine synthase
contig_1	cds	237285	237405	-	GLNHAC_02676	ribBA	bifunctional 3,4-dihydroxy-2-butanone-4-phosphate synthase
contig_1	cds	237406	237471	-	GLNHAC_02677	ribE	riboflavin synthase subunit alpha
contig_1	cds	237472	237580	-	GLNHAC_02678	ribD	bifunctional diaminohydroxyphosphoribosylaminopyrimidine d
contig_1	ncRNA-region	237593	237608	-			FMN riboswitch aptamer (RFN element)
contig_1	cds	237620	237654	-	GLNHAC_02679	ypuD	Uncharacterized protein YpuD
contig_1	cds	237678	237733	-	GLNHAC_02680	lepB	signal peptidase I
contig_1	cds	237740	237788	-	GLNHAC_02681		hypothetical protein
contig_1	cds	237788	237809	-	GLNHAC_02682	ypzJ	Uncharacterized protein YpzJ
contig_1	cds	237839	237860	+	GLNHAC_02683		Spore germination protein
contig_1	cds	237876	237919	-	GLNHAC_02684	ppiB	Peptidyl-prolyl cis-trans isomerase B
contig_1	cds	237944	238031	+	GLNHAC_02685	ypuA	Uncharacterized protein YpuA
contig_1	cds	238034	238167	-	GLNHAC_02686	lysA	diaminopimelate decarboxylase
contig_1	cds	238177	238325	-	GLNHAC_02687	spoVAF	Stage V sporulation protein AF
contig_1	cds	238320	238381	-	GLNHAC_02688	spoVAEA	stage V sporulation protein SpoVABEA
contig_1	cds	238382	238417	-	GLNHAC_02689	spoVAE	stage V sporulation protein AE
contig_1	cds	238417	238519	-	GLNHAC_02690	spoVAD	stage V sporulation protein AD
contig_1	cds	238520	238565	-	GLNHAC_02691	spoVAC	stage V sporulation protein AC
contig_1	cds	238566	238609	-	GLNHAC_02692	spoVAB	stage V sporulation protein SpoVAB
contig_1	cds	238608	238658	-	GLNHAC_02693		Stage V sporulation protein AA
contig_1	cds	238683	238759	-	GLNHAC_02694	sigF	RNA polymerase sporulation sigma factor SigF
contig_1	cds	238761	238805	-	GLNHAC_02695	spoIIAB	anti-sigma F factor
contig_1	cds	238804	238840	-	GLNHAC_02696	spoIIAA	anti-sigma F factor antagonist
contig_1	cds	238849	238966	-	GLNHAC_02697	dacF	D-alanyl-D-alanine carboxypeptidase DacF
contig_1	cds	238982	239064	-	GLNHAC_02698	punA	purine nucleoside phosphorylase I, inosine and guanosine-sp
contig_1	cds	239065	239183	-	GLNHAC_02699	deoB	phosphopentomutase
contig_1	cds	239200	239289	-	GLNHAC_02700	xerD	site-specific tyrosine recombinase XerD
contig_1	cds	239289	239312	-	GLNHAC_02701	yqzK	Uncharacterized membrane protein YqzK
contig_1	cds	239325	239369	-	GLNHAC_02702	fur	ferric iron uptake transcriptional regulator
contig_1	cds	239381	239445	-	GLNHAC_02703	spoIIM	stage II sporulation protein M
contig_1	cds	239455	239477	-	GLNHAC_02704	yqkK	YqkK
contig_1	cds	239487	239619	-	GLNHAC_02705	mleA	NAD-dependent malic enzyme 1
contig_1	cds	239621	239761	-	GLNHAC_02706	nhaC	Na+/H+ antiporter NhaC
contig_1	cds	239776	239882	-	GLNHAC_02707		Aspartate ammonia-lyase
contig_1	cds	239879	239899	-	GLNHAC_02708		hypothetical protein
contig_1	cds	239898	239918	-	GLNHAC_02709		hypothetical protein
contig_1	cds	239923	240022	-	GLNHAC_02710	ansA	asparaginase
contig_1	cds	240040	240075	+	GLNHAC_02711	ansR	HTH-type transcriptional regulator AnsR
contig_1	cds	240076	240192	-	GLNHAC_02712	yqxK	Uncharacterized protein YqxK
contig_1	cds	240192	240247	-	GLNHAC_02713	nudF	ADP-ribose pyrophosphatase
contig_1	cds	240255	240267	+	GLNHAC_02714	mciZ	Z-ring formation inhibitor MciZ
contig_1	cds	240273	240365	+	GLNHAC_02715	yqkF	Uncharacterized oxidoreductase YqkF
contig_1	cds	240369	240403	-	GLNHAC_02716		Conserved domain protein
contig_1	cds	240407	240499	+	GLNHAC_02717		Putative hydrolase
contig_1	cds	240503	240617	-	GLNHAC_02718	ftsW	putative lipid II flippase FtsW
contig_1	cds	240617	240733	-	GLNHAC_02719	rodA	rod shape-determining protein RodA
contig_1	cds	240734	240799	-	GLNHAC_02720		Helix-turn-helix domain protein
contig_1	cds	240845	240869	-	GLNHAC_02721	yqkC	Uncharacterized protein YqkC
contig_1	cds	240871	240903	-	GLNHAC_02722	yqkB	Uncharacterized protein YqkB
contig_1	cds	240903	241006	-	GLNHAC_02723		N-acetyltransferase domain-containing protein
contig_1	cds	241006	241039	-	GLNHAC_02724		Antibiotic biosynthesis monooxygenase family protein
contig_1	cds	241041	241088	-	GLNHAC_02725	yqjY	Uncharacterized protein YqjY
contig_1	cds	241107	241141	-	GLNHAC_02726	yqjX	Uncharacterized protein YqjX
contig_1	cds	241140	241264	-	GLNHAC_02727	dinB2	DNA polymerase IV 2
contig_1	cds	241281	241301	+	GLNHAC_02728	yqzH	Uncharacterized protein YqzH
contig_1	cds	241320	241333	-	GLNHAC_02729		MBL fold metallo-hydrolase
contig_1	cds	241329	241348	-	GLNHAC_02730		hypothetical protein
contig_1	cds	241354	241477	+	GLNHAC_02731	yqjV	Uncharacterized MFS-type transporter YqjV

contig_1	cds	241483	241498	+	GLNHAC_02732		DUF4083 domain-containing protein
contig_1	cds	241498	241537	-	GLNHAC_02733	yqjT	Uncharacterized protein YqjT
contig_1	cds	241537	241633	-	GLNHAC_02734	coaA	type I pantothenate kinase
contig_1	cds	241642	241697	-	GLNHAC_02735		D-serine dehydratase
contig_1	cds	241701	241775	-	GLNHAC_02736		D-serine dehydratase
contig_1	cds	241783	241830	-	GLNHAC_02737		SDR family NAD(P)-dependent oxidoreductase
contig_1	cds	241845	241860	-	GLNHAC_02738		hypothetical protein
contig_1	cds	241861	241957	-	GLNHAC_02739	yqjP	putative metallo-hydrolase YqjP
contig_1	ncRNA-region	241972	241993	+			T-box leader
contig_1	cds	241997	242081	+	GLNHAC_02740	proC	pyrroline-5-carboxylate reductase
contig_1	cds	242180	242249	-	GLNHAC_02741	yqjN	(pseudo) Uncharacterized protein YqjN
contig_1	cds	242266	242368	+	GLNHAC_02742	namA	NADPH dehydrogenase NamA
contig_1	cds	242379	242455	+	GLNHAC_02743	yqjL	Uncharacterized protein YqjL
contig_1	cds	242459	242474	-	GLNHAC_02744	rpmG	50S ribosomal protein L33
contig_1	cds	242482	242574	-	GLNHAC_02745	rnz	ribonuclease Z
contig_1	cds	242597	242744	+	GLNHAC_02746	zwf	glucose-6-phosphate dehydrogenase
contig_1	cds	242757	242898	-	GLNHAC_02747	gndA	NADP-dependent phosphogluconate dehydrogenase
contig_1	cds	242935	243034	-	GLNHAC_02748		DNA polymerase IV
contig_1	cds	243040	243070	+	GLNHAC_02749	mifM	membrane protein insertion/folding monitor MifM
contig_1	cds	243073	243155	+	GLNHAC_02750	misCB	Membrane protein insertase MisCB
contig_1	cds	243163	243235	+	GLNHAC_02751		DUF2071 domain-containing protein
contig_1	cds	243249	243361	-	GLNHAC_02752	yqjE	Uncharacterized protein YqjE
contig_1	cds	243363	243509	-	GLNHAC_02753	yqjD	Putative propionyl-CoA carboxylase beta chain
contig_1	cds	243514	243556	-	GLNHAC_02754	mce	methylmalonyl-CoA epimerase
contig_1	cds	243565	243575	+	GLNHAC_02755	prli42	stressosome-associated protein Prli42
contig_1	cds	243577	243630	-	GLNHAC_02756	yqjB	Putative L,D-transpeptidase YqjB
contig_1	cds	243635	243732	-	GLNHAC_02757	yqjA	Uncharacterized protein YqjA
contig_1	cds	243739	243811	-	GLNHAC_02758	artR	arginine ABC transporter ATP-binding protein ArtR
contig_1	cds	243811	243870	-	GLNHAC_02759	artQ	Arginine transport system permease protein ArtQ
contig_1	cds	243885	243961	-	GLNHAC_02760	artP	arginine ABC transporter substrate-binding protein ArtP
contig_1	cds	243987	244031	-	GLNHAC_02761	brxB	bacilliredoxin BrxB
contig_1	cds	244047	244135	+	GLNHAC_02762	bmrU	Putative lipid kinase BmrU
contig_1	cds	244143	244260	+	GLNHAC_02763	bmr	multidrug efflux MFS transporter Bmr
contig_1	cds	244267	244351	+	GLNHAC_02764	bmrR	multidrug efflux transcriptional regulator BmrR
contig_1	cds	244357	244485	-	GLNHAC_02765	bfmBB	Lipoamide acyltransferase component of branched-chain alpl
contig_1	cds	244488	244586	-	GLNHAC_02766	bfmBAB	3-methyl-2-oxobutanoate dehydrogenase subunit beta
contig_1	cds	244587	244686	-	GLNHAC_02767	bfmBAA	3-methyl-2-oxobutanoate dehydrogenase subunit alpha
contig_1	cds	244689	244724	-	GLNHAC_02768		Dihydrolipoyl dehydrogenase
contig_1	cds	244720	244831	-	GLNHAC_02769		Dihydrolipoyl dehydrogenase
contig_1	cds	244833	244942	-	GLNHAC_02770	buk	butyrate kinase
contig_1	cds	244944	245054	-	GLNHAC_02771	bcd	branched-chain amino acid dehydrogenase
contig_1	cds	245055	245145	-	GLNHAC_02772	yqiS	phosphate butyryltransferase
contig_1	cds	245157	245365	-	GLNHAC_02773	yqiR	Putative sigma L-dependent transcriptional regulator YqiR
contig_1	cds	245380	245404	+	GLNHAC_02774	yqzF	Uncharacterized protein YqzF
contig_1	cds	245408	245498	-	GLNHAC_02775	prpB	methylisocitrate lyase
contig_1	cds	245500	245642	-	GLNHAC_02776	mmgE	bifunctional 2-methylcitrate dehydratase/aconitate hydratase
contig_1	cds	245644	245756	-	GLNHAC_02777	mmgD	citrate synthase
contig_1	cds	245760	245873	-	GLNHAC_02778	mmgC	Acyl-CoA dehydrogenase
contig_1	cds	245876	245954	-	GLNHAC_02779		3-hydroxybutyryl-CoA dehydrogenase
contig_1	cds	245952	245962	-	GLNHAC_02780		hypothetical protein
contig_1	cds	245966	245999	-	GLNHAC_02781		hypothetical protein
contig_1	cds	245996	246060	-	GLNHAC_02782	mmgA	(pseudo) acetyl-CoA C-acetyltransferase
contig_1	cds	246058	246083	-	GLNHAC_02783		hypothetical protein
contig_1	cds	246176	246219	-	GLNHAC_02784	yqiI	(pseudo) Uncharacterized protein YqiI
contig_1	cds	246228	246237	-	GLNHAC_02785		hypothetical protein
contig_1	cds	246240	246269	-	GLNHAC_02786		Lipoprotein
contig_1	cds	246320	246434	+	GLNHAC_02787	yqiG	putative NADH-dependent flavin oxidoreductase YqiG
contig_1	cds	246439	246450	-	GLNHAC_02788		hypothetical protein
contig_1	cds	246464	246481	-	GLNHAC_02789		hypothetical protein
contig_1	cds	246502	246569	-	GLNHAC_02790		Zinc metalloprotease
contig_1	cds	246569	246888	-	GLNHAC_02791		Type I restriction enzyme endonuclease subunit
contig_1	cds	246888	247015	-	GLNHAC_02792		Type I restriction-modification enzyme, S subunit
contig_1	cds	247016	247304	-	GLNHAC_02793		type I restriction-modification system subunit M
contig_1	cds	247328	247342	-	GLNHAC_02794		hypothetical protein
contig_1	cds	247345	247371	-	GLNHAC_02795		hypothetical protein
contig_1	cds	247424	247437	-	GLNHAC_02796		hypothetical protein
contig_1	cds	247446	247512	+	GLNHAC_02797		hypothetical protein
contig_1	cds	247506	247524	-	GLNHAC_02798		hypothetical protein
contig_1	cds	247541	247638	-	GLNHAC_02799		DNA integration/recombination protein
contig_1	cds	247653	247733	-	GLNHAC_02800	spo0A	sporulation transcription factor Spo0A
contig_1	cds	247761	247782	-	GLNHAC_02801		SpoIVB peptidase S55 domain-containing protein
contig_1	cds	247791	247841	-	GLNHAC_02802		hypothetical protein
contig_1	cds	247840	247889	-	GLNHAC_02803		hypothetical protein
contig_1	cds	247906	248079	-	GLNHAC_02804	recN	DNA repair protein RecN
contig_1	cds	248083	248128	-	GLNHAC_02805	argR	arginine repressor ArgR
contig_1	cds	248137	248222	-	GLNHAC_02806	yqxC	Putative rRNA methyltransferase YqxC
contig_1	cds	248222	248412	-	GLNHAC_02807	dxs	1-deoxy-D-xylulose-5-phosphate synthase
contig_1	cds	248430	248519	-	GLNHAC_02808	ispA	Farnesyl diphosphate synthase
contig_1	cds	248518	248543	-	GLNHAC_02809	xseB	exodeoxyribonuclease VII small subunit
contig_1	cds	248543	248566	-	GLNHAC_02810		hypothetical protein
contig_1	cds	248565	248677	-	GLNHAC_02811	xseA	exodeoxyribonuclease VII large subunit
contig_1	cds	248691	248776	-	GLNHAC_02812	folD	bifunctional methylenetetrahydrofolate dehydrogenase/methe
contig_1	cds	248777	248817	-	GLNHAC_02813	nusB	transcription antitermination factor NusB
contig_1	cds	248843	248884	-	GLNHAC_02814	yqhY	fatty acid biosynthesis protein YqhY
contig_1	cds	248886	249021	-	GLNHAC_02815	accC	acetyl-CoA carboxylase biotin carboxylase subunit
contig_1	cds	249022	249071	-	GLNHAC_02816	accB	acetyl-CoA carboxylase biotin carboxyl carrier protein
contig_1	cds	249086	249152	-	GLNHAC_02817	spoIIIAH	stage III sporulation ratchet engulfment protein SpoIIIAH
contig_1	cds	249152	249221	-	GLNHAC_02818	spoIIIAG	stage III sporulation protein AG
contig_1	cds	249220	249282	-	GLNHAC_02819	spoIIIAF	stage III sporulation protein AF
contig_1	cds	249284	249403	-	GLNHAC_02820	spoIIIAE	stage III sporulation protein AE
contig_1	cds	249405	249445	-	GLNHAC_02821	spoIIIAD	stage III sporulation protein AD
contig_1	cds	249446	249467	-	GLNHAC_02822	spoIIIAC	stage III sporulation protein AC
contig_1	cds	249469	249521	-	GLNHAC_02823	spoIIIAB	stage III sporulation protein SpoIIIAB
contig_1	cds	249520	249612	-	GLNHAC_02824	spoIIIAA	stage III sporulation protein AA
contig_1	cds	249620	249648	-	GLNHAC_02825	yqhV	Uncharacterized protein YqhV
contig_1	cds	249662	249718	-	GLNHAC_02826	efp	elongation factor P
contig_1	cds	249721	249827	-	GLNHAC_02827	yqhT	aminopeptidase PapA
contig_1	cds	249827	249871	-	GLNHAC_02828	aroQ	type II 3-dehydroquinate dehydratase
contig_1	cds	249880	249922	-	GLNHAC_02829	yqhR	YqhR
contig_1	cds	249920	249933	-	GLNHAC_02830		hypothetical protein
contig_1	cds	249952	250012	+	GLNHAC_02831		DUF1385 domain-containing protein
contig_1	cds	250055	250095	+	GLNHAC_02832	yqhP	Uncharacterized protein YqhP
contig_1	cds	250094	250182	-	GLNHAC_02833	yqhO	Uncharacterized protein YqhO
contig_1	cds	250194	250237	-	GLNHAC_02834	mntR	transcriptional regulator MntR
contig_1	cds	250247	250331	-	GLNHAC_02835	lipM	octanoyltransferase LipM
contig_1	cds	250350	250388	+	GLNHAC_02836	yqhL	Uncharacterized protein YqhL
contig_1	cds	250392	250538	-	GLNHAC_02837	gcvPB	aminomethyl-transferring glycine dehydrogenase subunit Gcv
contig_1	cds	250538	250653	-	GLNHAC_02838	gcvPA	aminomethyl-transferring glycine dehydrogenase subunit Gcv
contig_1	cds	250648	250672	-	GLNHAC_02839		hypothetical protein
contig_1	cds	250675	250784	-	GLNHAC_02840	gcvT	glycine cleavage system aminomethyltransferase GcvT
contig_1	ncRNA-region	250801	250811	-			Glycine riboswitch aptamer
contig_1	cds	250828	250996	+	GLNHAC_02841	yqhH	Uncharacterized ATP-dependent helicase YqhH
contig_1	cds	250998	251069	+	GLNHAC_02842	yqhG	Uncharacterized protein YqhG
contig_1	cds	251096	251113	+	GLNHAC_02843	sinI	anti-repressor SinI
contig_1	cds	251116	251150	+	GLNHAC_02844	sinR	transcriptional regulator SinR
contig_1	cds	251159	251238	-	GLNHAC_02845	tasA	biofilm matrix protein TasA
contig_1	cds	251244	251301	-	GLNHAC_02846	sipW	signal peptidase I SipW
contig_1	cds	251300	251376	-	GLNHAC_02847	tapA	amyloid fiber anchoring/assembly protein TapA
contig_1	cds	251403	251435	+	GLNHAC_02848	yqzG	Uncharacterized protein YqzG
contig_1	cds	251440	251458	-	GLNHAC_02849	yqzE	Uncharacterized protein YqzE
contig_1	cds	251465	251502	-	GLNHAC_02850		Component of the DNA transport platform
contig_1	cds	251502	251538	-	GLNHAC_02851	comGF	competence type IV pilus minor pilin ComGF
contig_1	cds	251543	251578	-	GLNHAC_02852	comGE	competence type IV pilus minor pilin ComGE
contig_1	cds	251583	251619	-	GLNHAC_02853	comGD	competence type IV pilus minor pilin ComGD
contig_1	cds	251618	251648	-	GLNHAC_02854	comGC	comG operon protein ComGC
contig_1	cds	251649	251753	-	GLNHAC_02855	comGB	competence type IV pilus assembly protein ComGB
contig_1	cds	251752	251859	-	GLNHAC_02856	comGA	competence protein ComGA
contig_1	cds	251880	251921	-	GLNHAC_02857		CBS domain-containing protein
contig_1	cds	251927	252023	-	GLNHAC_02858	corA	Magnesium transport protein CorA
contig_1	cds	252038	252170	+	GLNHAC_02859	yqhB	UPF0053 protein YqhB
contig_1	cds	252174	252258	-	GLNHAC_02860		Component of the piezosome (Stressosome)
contig_1	tRNA	252266	252273	+	GLNHAC_02861	trnQ	tRNA-Gln(ttg)
contig_1	cds	252280	252318	-	GLNHAC_02862	mgsR	transcriptional regulator MgsR
contig_1	cds	252341	252366	+	GLNHAC_02863	yqgY	Uncharacterized protein YqgY
contig_1	cds	252370	252433	-	GLNHAC_02864	yqgX	putative metallo-hydrolase YqgX
contig_1	cds	252449	252466	+	GLNHAC_02865	yqgW	Uncharacterized protein YqgW
contig_1	cds	252469	252501	-	GLNHAC_02866	yqgV	UPF0045 protein YqgV
contig_1	cds	252501	252607	-	GLNHAC_02867		YqgU-like 6-bladed beta-propeller domain-containing protein

**contig_1**	cds	252613	252726	-	GLNHAC_02868	yqgT	Uncharacterized protein YqgT
**contig_1**	cds	252735	252926	-	GLNHAC_02869	yqgS	Lipoteichoic acid synthase-like YqgS
**contig_1**	cds	252938	253012	-	GLNHAC_02870		Glucokinase
**contig_1**	cds	253012	253034	-	GLNHAC_02871		hypothetical protein
**contig_1**	cds	253036	253058	-	GLNHAC_02872		DUF910 domain-containing protein
**contig_1**	cds	253071	253163	-	GLNHAC_02873	yqgP	(pseudo) rhomboid protease YqgP
**contig_1**	cds	253163	253196	-	GLNHAC_02874		hypothetical protein
**contig_1**	cds	253196	253220	-	GLNHAC_02875		hypothetical protein
**contig_1**	cds	253233	253246	-	GLNHAC_02876	yqgO	(pseudo) Uncharacterized protein YqgO
**contig_1**	cds	253253	253309	-	GLNHAC_02877		5-formyltetrahydrofolate cyclo-ligase
**contig_1**	cds	253317	253332	-	GLNHAC_02878	rpmG	50S ribosomal protein L33
**contig_1**	cds	253341	253449	-	GLNHAC_02879	yqgM	YqgM
**contig_1**	cds	253448	253495	-	GLNHAC_02880	yqgL	YqgL
**contig_1**	cds	253513	253549	+	GLNHAC_02881	yqzD	Uncharacterized protein YqzD
**contig_1**	cds	253548	253595	+	GLNHAC_02882	yqzC	Uncharacterized protein YqzC
**contig_1**	cds	253598	253676	-	GLNHAC_02883	pstB	phosphate ABC transporter ATP-binding protein PstB
**contig_1**	cds	253677	253758	-	GLNHAC_02884	pstB	phosphate ABC transporter ATP-binding protein PstB
**contig_1**	cds	253760	253849	-	GLNHAC_02885	pstA	phosphate ABC transporter permease PstA
**contig_1**	cds	253849	253941	-	GLNHAC_02886	pstC	phosphate ABC transporter permease subunit PstC
**contig_1**	cds	253948	253985	-	GLNHAC_02887		hypothetical protein
**contig_1**	cds	253989	254057	-	GLNHAC_02888	pstS	(pseudo) Phosphate-binding protein PstS
**contig_1**	cds	254054	254269	-	GLNHAC_02889	pbpA	Penicillin-binding protein 2A
**contig_1**	cds	254280	254410	-	GLNHAC_02890	yqgE	Uncharacterized protein YqgE
**contig_1**	cds	254420	254481	-	GLNHAC_02891	sodA	Superoxide dismutase [Mn]
**contig_1**	cds	254499	254547	-	GLNHAC_02892	yqgC	Uncharacterized protein YqgC
**contig_1**	cds	254558	254676	+	GLNHAC_02893		hypothetical protein
**contig_1**	cds	254688	254801	+	GLNHAC_02894	ispG	flavodoxin-dependent (E)-4-hydroxy-3-methylbut-2-enyl-diph
**contig_1**	cds	254804	254842	-	GLNHAC_02895	yqfX	Uncharacterized protein YqfX
**contig_1**	cds	254856	254914	+	GLNHAC_02896	yqfW	Putative nucleotidase YqfW
**contig_1**	cds	254918	254962	-	GLNHAC_02897	zur	zinc uptake transcriptional repressor Zur
**contig_1**	cds	254976	255064	-	GLNHAC_02898	yqfU	UPF0750 membrane protein YqfU
**contig_1**	cds	255076	255101	+	GLNHAC_02899	yqfT	Uncharacterized protein YqfT
**contig_1**	cds	255104	255193	-	GLNHAC_02900	nfo	deoxyribonuclease IV
**contig_1**	cds	255194	255326	-	GLNHAC_02901	cshB	DEAD-box ATP-dependent RNA helicase CshB
**contig_1**	cds	255343	255415	+	GLNHAC_02902		YqfQ-like protein
**contig_1**	cds	255427	255521	+	GLNHAC_02903	ispH	4-hydroxy-3-methylbut-2-enyl diphosphate reductase
**contig_1**	cds	255524	255636	-	GLNHAC_02904	yqfO	Nif3-like dinuclear metal center hexameric protein
**contig_1**	cds	255635	255700	-	GLNHAC_02905	trmK	tRNA (adenine(22)-N(1))-methyltransferase
**contig_1**	cds	255726	255762	-	GLNHAC_02906	cccA	cytochrome c-550
**contig_1**	cds	255795	255906	-	GLNHAC_02907	rpoD	RNA polymerase sigma factor RpoD
**contig_1**	cds	255926	256107	-	GLNHAC_02908	dnaG	DNA primase
**contig_1**	cds	256118	256160	-	GLNHAC_02909	yqxD	UPF0178 protein YqxD
**contig_1**	cds	256185	256266	-	GLNHAC_02910	yqfL	Putative pyruvate, phosphate dikinase regulatory protein
**contig_1**	cds	256269	256333	-	GLNHAC_02911	ccpN	transcriptional regulator CcpN
**contig_1**	cds	256346	256404	-	GLNHAC_02912		DALR anticodon-binding domain-containing protein
**contig_1**	cds	256400	256546	-	GLNHAC_02913		glycine--tRNA ligase
**contig_1**	cds	256549	256642	-	GLNHAC_02914	glyQ	glycine--tRNA ligase subunit alpha
**contig_1**	cds	256668	256744	-	GLNHAC_02915	recO	DNA repair protein RecO
**contig_1**	cds	256748	256762	-	GLNHAC_02916	yqzL	Uncharacterized protein YqzL
**contig_1**	cds	256777	256867	-	GLNHAC_02917	era	GTPase Era
**contig_1**	cds	256865	256906	-	GLNHAC_02918	cdd	cytidine deaminase
**contig_1**	cds	256919	256956	-	GLNHAC_02919	dgkA	Undecaprenol kinase
**contig_1**	cds	256954	257001	-	GLNHAC_02920	ybeY	rRNA maturation RNase YbeY
**contig_1**	cds	257001	257215	-	GLNHAC_02921	pgpH	cyclic-di-AMP phosphodiesterase PgpH
**contig_1**	cds	257209	257223	-	GLNHAC_02922		hypothetical protein
**contig_1**	cds	257223	257318	-	GLNHAC_02923	phoH	PhoH-like protein
**contig_1**	cds	257318	257438	-	GLNHAC_02924	yqfD	sporulation protein YqfD
**contig_1**	cds	257440	257468	-	GLNHAC_02925	yqfC	sporulation protein YqfC
**contig_1**	cds	257473	257515	-	GLNHAC_02926	yqfB	YqfB
**contig_1**	cds	257518	257617	-	GLNHAC_02927	floA	flotillin-like protein FloA
**contig_1**	cds	257619	257751	-	GLNHAC_02928	nfeD1	Membrane protein NfeD1b
**contig_1**	cds	257764	257808	-	GLNHAC_02929	yqeY	GatB/YqeY domain-containing protein
**contig_1**	cds	257810	257827	-	GLNHAC_02930	rpsU	30S ribosomal protein S21
**contig_1**	cds	257843	257937	+	GLNHAC_02931	yqeW	Uncharacterized protein YqeW
**contig_1**	cds	257940	258076	-	GLNHAC_02932	mtaB	tRNA (N(6)-L-threonylcarbamoyladenosine(37)-C(2))-methylthi
**contig_1**	cds	258076	258153	-	GLNHAC_02933	rsmE	16S rRNA (uracil(1498)-N(3))-methyltransferase
**contig_1**	cds	258155	258249	-	GLNHAC_02934	prmA	50S ribosomal protein L11 methyltransferase
**contig_1**	cds	258251	258364	-	GLNHAC_02935	dnaJ	molecular chaperone DnaJ
**contig_1**	ncRNA	258368	258384	+	GLNHAC_02936	surC	SurC sRNA
**contig_1**	cds	258384	258567	-	GLNHAC_02937	dnaK	molecular chaperone DnaK
**contig_1**	cds	258570	258626	-	GLNHAC_02938	grpE	nucleotide exchange factor GrpE
**contig_1**	cds	258633	258736	-	GLNHAC_02939	hrcA	heat-inducible transcriptional repressor HrcA
**contig_1**	cds	258744	258858	-	GLNHAC_02940	hemW	radical SAM family heme chaperone HemW
**contig_1**	cds	258864	259047	-	GLNHAC_02941	lepA	translation elongation factor 4
**contig_1**	cds	259061	259094	-	GLNHAC_02942	yqxA	Uncharacterized protein YqxA
**contig_1**	cds	259096	259217	-	GLNHAC_02943	spoIIP	Stage II sporulation protein P
**contig_1**	cds	259223	259334	-	GLNHAC_02944	gpr	GPR endopeptidase
**contig_1**	cds	259354	259381	+	GLNHAC_02945	rpsT	30S ribosomal protein S20
**contig_1**	cds	259382	259486	-	GLNHAC_02946	holA	DNA polymerase III subunit delta
**contig_1**	cds	259534	259760	-	GLNHAC_02947	comE	ComE operon protein 3
**contig_1**	cds	259760	259817	-	GLNHAC_02948	comEB	ComE operon protein 2
**contig_1**	cds	259824	259886	-	GLNHAC_02949		Membrane bound high-affinity DNA-binding receptor
**contig_1**	cds	259894	259976	+	GLNHAC_02950	comER	late competence protein ComER
**contig_1**	cds	259981	260057	-	GLNHAC_02951	yqeM	Putative methyltransferase YqeM
**contig_1**	cds	260057	260092	-	GLNHAC_02952	rsfS	ribosome silencing factor
**contig_1**	cds	260094	260150	-	GLNHAC_02953	yqeK	bis(5'-nucleosyl)-tetraphosphatase (symmetrical) YqeK
**contig_1**	cds	260149	260189	-	GLNHAC_02954		nicotinate-nucleotide adenylyltransferase
**contig_1**	cds	260194	260206	-	GLNHAC_02955		(pseudo) nicotinate-nucleotide adenylyltransferase
**contig_1**	cds	260207	260236	-	GLNHAC_02956	yhbY	ribosome assembly RNA-binding protein YhbY
**contig_1**	cds	260236	260283	-	GLNHAC_02957	aroE	(pseudo) shikimate dehydrogenase
**contig_1**	cds	260283	260320	-	GLNHAC_02958	aroE	(pseudo) shikimate dehydrogenase
**contig_1**	cds	260322	260432	-	GLNHAC_02959	yqeH	ribosome biogenesis GTPase YqeH
**contig_1**	cds	260432	260485	-	GLNHAC_02960	yqeG	putative phosphatase YqeG
**contig_1**	cds	260548	260557	-	GLNHAC_02961	yjcZ	Sporulation protein YjcZ
**contig_1**	cds	260564	260638	-	GLNHAC_02962	yqeF	Uncharacterized lipoprotein YqeF
**contig_1**	cds	260662	260737	-	GLNHAC_02963	cwlH	N-acetylmuramoyl-L-alanine amidase CwlH
**contig_1**	cds	260756	260819	+	GLNHAC_02964		TVP38/TMEM64 family membrane protein
**contig_1**	cds	260821	260910	-	GLNHAC_02965	gnd	phosphogluconate dehydrogenase (NAD(+)-dependent, deca
**contig_1**	cds	260943	261014	+	GLNHAC_02966		DUF308 domain-containing protein
**contig_1**	cds	261018	261059	-	GLNHAC_02967	nucB	sporulation-specific Dnase NucB
**contig_1**	cds	261078	261151	+	GLNHAC_02968	sigK	RNA polymerase sporulation sigma factor SigK
**contig_1**	cds	261162	261187	-	GLNHAC_02969		Site-specific DNA recombinase
**contig_1**	cds	261253	261378	-	GLNHAC_02970		Alanine--tRNA ligase
**contig_1**	cds	261386	261496	-	GLNHAC_02971	dsd1	Putative alanine racemase
**contig_1**	cds	261496	261535	-	GLNHAC_02972	ridA	Putative translation initiation inhibitor
**contig_1**	cds	261537	261634	-	GLNHAC_02973		L-asparaginase
**contig_1**	cds	261639	261749	-	GLNHAC_02974	thrC	Threonine synthase
**contig_1**	cds	261757	261794	-	GLNHAC_02975	ridA	Putative Endoribonuclease L-PSP
**contig_1**	cds	261795	261861	-	GLNHAC_02976	yheO	DNA-binding protein
**contig_1**	cds	261924	261975	+	GLNHAC_02977	rapE	(pseudo) response regulator aspartate phosphatase RapE
**contig_1**	cds	261994	262021	+	GLNHAC_02978		hypothetical protein
**contig_1**	cds	262036	262049	+	GLNHAC_02979	phrE	phosphatase RapE inhibitor PhrE
**contig_1**	cds	262072	262233	+	GLNHAC_02980	yqcG	Toxin YqcG
**contig_1**	cds	262235	262274	+	GLNHAC_02981		Immunity protein 50
**contig_1**	cds	262275	262319	+	GLNHAC_02982		Knr4/Smi1-like domain-containing protein
**contig_1**	cds	262346	262376	+	GLNHAC_02983		hypothetical protein
**contig_1**	cds	262375	262409	+	GLNHAC_02984		Rhoptry protein
**contig_1**	cds	262415	262427	+	GLNHAC_02985		hypothetical protein
**contig_1**	cds	262446	262532	+	GLNHAC_02986		Sialate O-acetylesterase domain-containing protein
**contig_1**	cds	262534	262585	+	GLNHAC_02987	padR	Transcription regulator PadR N-terminal domain-containing p
**contig_1**	cds	262633	262710	-	GLNHAC_02988		putative membrane transporter protein
**contig_1**	cds	262717	262739	-	GLNHAC_02989	tusA	sulfurtransferase TusA family protein
**contig_1**	cds	262743	262855	-	GLNHAC_02990	yrkH	Uncharacterized protein YrkH
**contig_1**	cds	262890	262945	-	GLNHAC_02991	yrkF	Putative sulfur carrier protein YrkF
**contig_1**	cds	262952	262987	-	GLNHAC_02992		Rhodanese-like domain-containing protein
**contig_1**	cds	262993	263041	-	GLNHAC_02993	yrkE	Uncharacterized protein YrkE
**contig_1**	cds	263060	263082	-	GLNHAC_02994	yrkD	Uncharacterized protein YrkD
**contig_1**	cds	263084	263107	-	GLNHAC_02995		sulfurtransferase
**contig_1**	cds	263186	263200	-	GLNHAC_02996		hypothetical protein
**contig_1**	cds	263309	263450	-	GLNHAC_02997		Hydroxyacylglutathione hydrolase
**contig_1**	cds	263457	263576	-	GLNHAC_02998	fadH2	Sulfide:quinone oxidoreductase
**contig_1**	cds	263591	263656	-	GLNHAC_02999	citB	Two-component response regulator
**contig_1**	cds	263653	263762	-	GLNHAC_03000	comP	Sensor histidine kinase
**contig_1**	cds	263794	263872	-	GLNHAC_03001		putative membrane transporter protein
**contig_1**	cds	263925	263966	-	GLNHAC_03002		Ester cyclase
**contig_1**	cds	263971	264047	-	GLNHAC_03003		Metallo-beta-lactamase domain-containing protein
**contig_1**	cds	264070	264085	-	GLNHAC_03004		hypothetical protein
**contig_1**	cds	264203	264286	-	GLNHAC_03005		Transcription antiterminator
**contig_1**	cds	264294	264333	-	GLNHAC_03006	yidH	YidH family protein

contig_1	cds	264359	264499	-	GLNHAC_03007		6-phospho-beta-glucosidase
contig_1	cds	264501	264686	-	GLNHAC_03008		Pts system beta-glucoside-specific eiibca component
contig_1	cds	264737	264981	-	GLNHAC_03009	copZ	copper chaperone CopZ
contig_1	cds	264988	265009	-	GLNHAC_03010	copZ	copper chaperone CopZ
contig_1	cds	265015	265049	-	GLNHAC_03011	csoR	CsoR family transcriptional regulator
contig_1	cds	265085	265131	-	GLNHAC_03012		DinB-like domain-containing protein
contig_1	cds	265147	265203	-	GLNHAC_03013		Cysteine dioxygenase type I
contig_1	cds	265241	265270	+	GLNHAC_03014	irrE	IrrE N-terminal-like domain-containing protein
contig_1	cds	265304	265332	+	GLNHAC_03015		RNA polymerase sigma-K factor
contig_1	cds	265407	265481	-	GLNHAC_03016		NADP oxidoreductase, coenzyme f420-dependent
contig_1	cds	265504	265540	+	GLNHAC_03017		Cinnamoyl ester hydrolase
contig_1	cds	265578	265652	+	GLNHAC_03018	nfsA	oxygen-insensitive NADPH nitroreductase
contig_1	cds	265673	265700	-	GLNHAC_03019		Barstar (barnase inhibitor) domain-containing protein
contig_1	cds	265723	265761	+	GLNHAC_03020		DUF3139 domain-containing protein
contig_1	cds	265813	265895	-	GLNHAC_03021	bltR	multidrug efflux transcriptional regulator BltR
contig_1	cds	265908	266028	+	GLNHAC_03022	blt	multidrug efflux MFS transporter Blt
contig_1	cds	266063	266109	+	GLNHAC_03023	bltD	spermine/spermidine acetyltransferase
contig_1	sorf	266202	266210	+	GLNHAC_03024	yjcZ	YjcZ family sporulation protein
contig_1	cds	266280	266336	-	GLNHAC_03025	rutF	Putative Flavoredoxin
contig_1	cds	266339	266355	-	GLNHAC_03026		Arginase/agmatinase/formiminoglutamase
contig_1	cds	266368	266382	-	GLNHAC_03027		hypothetical protein
contig_1	cds	266387	266448	-	GLNHAC_03028		helix-turn-helix domain-containing protein
contig_1	cds	266462	266474	+	GLNHAC_03029		Dioxygenase
contig_1	cds	266496	266530	-	GLNHAC_03030		MmcQ/YjbR family DNA-binding protein
contig_1	cds	266573	266733	-	GLNHAC_03031		Phosphatidic acid phosphatase type 2/haloperoxidase domai
contig_1	cds	266785	266860	-	GLNHAC_03032	yrpE	putative metal-binding protein YrpE
contig_1	cds	266867	266884	-	GLNHAC_03033		hypothetical protein
contig_1	cds	266903	266938	-	GLNHAC_03034	yraJ	Uncharacterized protein YraJ
contig_1	cds	266943	266979	-	GLNHAC_03035		SH3b domain-containing protein
contig_1	cds	267022	267047	+	GLNHAC_03036	yraG	Spore coat protein F-like protein YraG
contig_1	cds	267049	267086	+	GLNHAC_03037	yraF	Spore coat protein F-like protein YraF
contig_1	cds	267087	267201	+	GLNHAC_03038	adhB	Uncharacterized zinc-type alcohol dehydrogenase-like protei
contig_1	cds	267203	267223	+	GLNHAC_03039	yraE	Uncharacterized protein YraE
contig_1	cds	267224	267254	+	GLNHAC_03040		Coat F domain family
contig_1	cds	267318	267345	-	GLNHAC_03041	merR	MerR family transcriptional regulator
contig_1	cds	267351	267392	-	GLNHAC_03042	adhR	aldehyde stress transcriptional regulator AdhR
contig_1	cds	267411	267484	+	GLNHAC_03043		4-carboxymuconolactone decarboxylase domain protein
contig_1	cds	267506	267611	+	GLNHAC_03044	adhA	formaldehyde dehydrogenase AdhA
contig_1	cds	267626	267658	+	GLNHAC_03045		DJ-1/PfpI domain-containing protein
contig_1	cds	267658	267677	+	GLNHAC_03046		DJ-1/PfpI family protein
contig_1	cds	267705	267884	-	GLNHAC_03047		GH32 C-terminal domain-containing protein
contig_1	cds	267900	267983	-	GLNHAC_03048	levG	PTS fructose transporter subunit IID
contig_1	cds	267985	268066	-	GLNHAC_03049	levF	PTS fructose transporter subunit IIC
contig_1	cds	268068	268117	-	GLNHAC_03050	levE	PTS fructose transporter subunit IIB
contig_1	cds	268117	268161	-	GLNHAC_03051	levD	PTS fructose transporter subunit IIA
contig_1	cds	268180	268461	-	GLNHAC_03052	levR	transcriptional regulator LevR
contig_1	cds	268500	268530	-	GLNHAC_03053		hypothetical protein
contig_1	cds	268554	268693	+	GLNHAC_03054	aapA	Amino-acid permease AapA
contig_1	cds	268706	268770	-	GLNHAC_03055	yrhP	Uncharacterized membrane protein YrhP
contig_1	cds	268785	268868	+	GLNHAC_03056	yrhO	Uncharacterized protein YrhO
contig_1	cds	268888	268938	+	GLNHAC_03057	sigV	RNA polymerase sigma factor SigV
contig_1	cds	268938	269024	+	GLNHAC_03058	rsiV	Anti-sigma-V factor RsiV
contig_1	cds	269035	269226	+	GLNHAC_03059	oatA	O-acetyltransferase OatA
contig_1	cds	269239	269268	+	GLNHAC_03060	yrhK	YrhK domain-containing protein
contig_1	ncRNA	269281	269292	+	GLNHAC_03061	ncr1175	Bacillus sRNA ncr1175
contig_1	cds	269292	269388	-	GLNHAC_03062		NADPH--hemoprotein reductase
contig_1	cds	269409	269591	-	GLNHAC_03063		cytochrome P450
contig_1	cds	269585	269609	-	GLNHAC_03064		hypothetical protein
contig_1	cds	269611	269669	-	GLNHAC_03065	fatR	transcriptional regulator FatR
contig_1	cds	269691	269745	-	GLNHAC_03066		class I SAM-dependent methyltransferase
contig_1	cds	269796	269811	-	GLNHAC_03067	yrzI	Uncharacterized protein YrzI
contig_1	cds	269850	269930	-	GLNHAC_03068	yrhG	Uncharacterized transporter YrhG
contig_1	cds	269956	269999	-	GLNHAC_03069	yrhF	Uncharacterized protein YrhF
contig_1	cds	270024	270319	+	GLNHAC_03070	fdhF	formate dehydrogenase subunit alpha
contig_1	cds	270321	270369	+	GLNHAC_03071	yrhD	Uncharacterized protein YrhD
contig_1	cds	270404	270511	-	GLNHAC_03072	mccB	bifunctional cystathionine gamma-lyase/homocysteine desulf
contig_1	cds	270518	270610	-	GLNHAC_03073	mccA	O-acetylserine dependent cystathionine beta-synthase
contig_1	cds	270617	270686	-	GLNHAC_03074	mtnN	5'-methylthioadenosine/S-adenosylhomocysteine nucleosida
contig_1	cds	270688	270752	-	GLNHAC_03075	yrrT	Uncharacterized methyltransferase YrrT
contig_1	cds	270772	270792	+	GLNHAC_03076	yrzA	Uncharacterized protein YrzA
contig_1	cds	270796	270815	-	GLNHAC_03077		DUF1510 domain-containing protein
contig_1	cds	270835	270865	-	GLNHAC_03078		hypothetical protein
contig_1	cds	270871	271047	-	GLNHAC_03079	pbpI	Penicillin-binding protein 4B
contig_1	cds	271052	271099	-	GLNHAC_03080	greA	transcription elongation factor GreA
contig_1	cds	271125	271188	-	GLNHAC_03081	udk	uridine kinase
contig_1	cds	271189	271315	-	GLNHAC_03082	trhP1	tRNA hydroxylation protein P1
contig_1	cds	271317	271410	-	GLNHAC_03083	trhP2	tRNA hydroxylation protein P2
contig_1	cds	271411	271476	-	GLNHAC_03084	trmR	tRNA 5-hydroxyuridine methyltransferase
contig_1	cds	271491	271593	-	GLNHAC_03085	mltG	Endolytic murein transglycosylase
contig_1	cds	271613	271642	-	GLNHAC_03086	yrzB	UPF0473 protein YrzB
contig_1	cds	271647	271685	-	GLNHAC_03087	ruvX	Holliday junction resolvase RuvX
contig_1	cds	271686	271712	-	GLNHAC_03088	yrzL	IreB family regulatory phosphoprotein
contig_1	cds	271721	271792	-	GLNHAC_03089		DHHA1 domain-containing protein
contig_1	cds	271815	271828	-	GLNHAC_03090		hypothetical protein
contig_1	cds	271828	271984	-	GLNHAC_03091		Alanine--tRNA ligase
contig_1	cds	272017	272124	-	GLNHAC_03092	yrrI	Putative transport protein YrrI
contig_1	cds	272227	272467	-	GLNHAC_03093	recD2	ATP-dependent RecD2 DNA helicase
contig_1	cds	272469	272534	-	GLNHAC_03094		hypothetical protein
contig_1	cds	272540	272651	-	GLNHAC_03095	mnmA	tRNA 2-thiouridine(34) synthase MnmA
contig_1	cds	272668	272768	-	GLNHAC_03096	iscS1	Putative cysteine desulfurase IscS 1
contig_1	cds	272770	272812	-	GLNHAC_03097	cymR	cysteine metabolism transcriptional regulator CymR
contig_1	cds	272832	272958	+	GLNHAC_03098	yrvN	Uncharacterized AAA domain-containing protein YrvN
contig_1	cds	272963	273039	-	GLNHAC_03099	tcdA	tRNA threonylcarbamoyladenosine dehydratase
contig_1	ncRNA	273048	273067	-	GLNHAC_03100	6S	6S / SsrS RNA
contig_1	cds	273073	273189	-	GLNHAC_03101	aspS	(pseudo) aspartate--tRNA ligase
contig_1	cds	273189	273251	-	GLNHAC_03102	aspS	(pseudo) aspartate--tRNA ligase
contig_1	cds	273252	273379	-	GLNHAC_03103	hisS	histidine--tRNA ligase
contig_1	ncRNA-region	273389	273412	-			T-box leader
contig_1	cds	273418	273435	-	GLNHAC_03104	yrzK	Uncharacterized protein YrzK
contig_1	cds	273448	273516	+	GLNHAC_03105		SH3 domain-containing protein
contig_1	cds	273564	273603	+	GLNHAC_03106		hypothetical protein
contig_1	cds	273606	273650	-	GLNHAC_03107	dtd	D-aminoacyl-tRNA deacylase
contig_1	cds	273651	273720	-	GLNHAC_03108		(pseudo) bifunctional (p)ppGpp synthetase/guanosine-3',5'-bi
contig_1	cds	273719	273813	-	GLNHAC_03109		(pseudo) GTP pyrophosphokinase
contig_1	cds	273813	273871	-	GLNHAC_03110		HD domain-containing protein
contig_1	cds	273888	273939	-	GLNHAC_03111	apt	adenine phosphoribosyltransferase
contig_1	cds	273940	274176	-	GLNHAC_03112	recJ	single-stranded-DNA-specific exonuclease RecJ
contig_1	cds	274183	274216	-	GLNHAC_03113		DUF1049 domain-containing protein
contig_1	cds	274224	274273	-	GLNHAC_03114	trkA	TrkA domain protein
contig_1	cds	274289	274510	-	GLNHAC_03115	secDF	protein translocase subunit SecDF
contig_1	cds	274514	274544	-	GLNHAC_03116	comN	post-transcriptional regulator ComN
contig_1	cds	274555	274711	+	GLNHAC_03117	spoVB	stage V sporulation protein B
contig_1	cds	274711	274777	-	GLNHAC_03118	yrbG	UPF0702 transmembrane protein YrbG
contig_1	cds	274790	274836	+	GLNHAC_03119	yrzE	Uncharacterized membrane protein YrzE
contig_1	cds	274841	274868	-	GLNHAC_03120	yajC	preprotein translocase subunit YajC
contig_1	cds	274872	274986	-	GLNHAC_03121	tgt	tRNA guanosine(34) transglycosylase Tgt
contig_1	cds	274989	275053	-	GLNHAC_03122		S-adenosylmethionine:tRNA ribosyltransferase-isomerase
contig_1	cds	275095	275115	-	GLNHAC_03123	yrzS	Uncharacterized membrane protein YrzS
contig_1	cds	275114	275214	-	GLNHAC_03124	ruvB	Holliday junction branch migration DNA helicase RuvB
contig_1	cds	275215	275269	-	GLNHAC_03125	ruvA	Holliday junction branch migration protein RuvA
contig_1	cds	275290	275341	-	GLNHAC_03126	bofC	sporulation cell-cell signaling protein BofC
contig_1	cds	275346	275477	-	GLNHAC_03127	csbX	Alpha-ketoglutarate permease
contig_1	cds	275485	275506	-	GLNHAC_03128		(pseudo) Gfo/Idh/MocA family oxidoreductase
contig_1	cds	275506	275535	-	GLNHAC_03129		hypothetical protein
contig_1	cds	275537	275587	-	GLNHAC_03130		Oxidoreductase, NAD-binding, GFO/IDH/MOCA
contig_1	cds	275611	275675	+	GLNHAC_03131	yrzF	Putative serine/threonine-protein kinase YrzF
contig_1	cds	275680	275692	-	GLNHAC_03132		Helix-turn-helix domain-containing protein
contig_1	cds	275719	275865	+	GLNHAC_03133	yrbD	Putative sodium/proton-dependent alanine carrier protein Yrb
contig_1	cds	275869	275941	-	GLNHAC_03134	yrbC	YebC/PmpR family DNA-binding transcriptional regulator
contig_1	cds	275951	276011	-	GLNHAC_03135	coxA	Sporulation cortex protein CoxA
contig_1	cds	276026	276142	-	GLNHAC_03136	safA	spore coat assembly protein SafA
contig_1	cds	276153	276264	-	GLNHAC_03137	nadA	quinolinate synthase NadA
contig_1	cds	276263	276350	-	GLNHAC_03138	nadC	carboxylating nicotinate-nucleotide diphosphorylase
contig_1	cds	276345	276504	-	GLNHAC_03139	nadB	L-aspartate oxidase
contig_1	cds	276515	276636	+	GLNHAC_03140	nifS	Putative cysteine desulfurase NifS
contig_1	cds	276632	276684	+	GLNHAC_03141	nadR	Transcription repressor NadR
contig_1	cds	276686	276703	-	GLNHAC_03142		ACT domain-containing protein
contig_1	cds	276710	276774	-	GLNHAC_03143		prephenate dehydratase
contig_1	cds	276773	276817	-	GLNHAC_03144	thrR	transcriptional regulator ThrR

contig_1	cds	276823	276952	-	GLNHAC_03145	obgE	GTPase ObgE
contig_1	cds	276955	277013	-	GLNHAC_03146	spo0B	sporulation initiation phosphotransferase Sop0B
contig_1	cds	277045	277074	-	GLNHAC_03147	rpmA	50S ribosomal protein L27
contig_1	cds	277075	277109	-	GLNHAC_03148	prp	ribosomal-processing cysteine protease Prp
contig_1	cds	277109	277140	-	GLNHAC_03149	rplU	50S ribosomal protein L21
contig_1	ncRNA-region	277141	277149	-			Ribosomal protein L21 leader
contig_1	cds	277154	277241	-	GLNHAC_03150	spoIVFB	stage IV sporulation intramembrane metalloprotease SpoIVFE
contig_1	cds	277240	277320	-	GLNHAC_03151	spoIVFA	stage IV sporulation protein SpoIVFA
contig_1	cds	277335	277415	-	GLNHAC_03152	minD	septum site-determining protein MinD
contig_1	cds	277415	277483	-	GLNHAC_03153	minC	septum site-determining protein MinC
contig_1	cds	277489	277540	-	GLNHAC_03154	mreD	rod shape-determining protein MreD
contig_1	cds	277540	277627	-	GLNHAC_03155	mreC	rod shape-determining protein MreC
contig_1	cds	277630	277732	-	GLNHAC_03156	mreB	rod shape-determining protein MreB
contig_1	cds	277741	277810	-	GLNHAC_03157	radC	DNA repair protein RadC
contig_1	cds	277814	277871	-	GLNHAC_03158	maf	dTTP/UTP pyrophosphatase
contig_1	cds	277886	277985	-	GLNHAC_03159		SPOR domain-containing protein
contig_1	cds	277999	278073	-	GLNHAC_03160	comC	Prepilin leader peptidase/N-methyltransferase
contig_1	cds	278087	278135	-	GLNHAC_03161		hypothetical protein
contig_1	cds	278139	278216	-	GLNHAC_03162		hypothetical protein
contig_1	cds	278222	278269	-	GLNHAC_03163		valine--tRNA ligase
contig_1	cds	278271	278427	-	GLNHAC_03164		valine--tRNA ligase
contig_1	cds	278425	278449	-	GLNHAC_03165		valine--tRNA ligase
contig_1	cds	278463	278487	-	GLNHAC_03166		hypothetical protein
contig_1	ncRNA-region	278493	278515	-			T-box leader
contig_1	cds	278552	278595	-	GLNHAC_03167	ysxE	(pseudo) spore coat protein YsxE
contig_1	cds	278595	278656	-	GLNHAC_03168	ysxE	(pseudo) spore coat protein YsxE
contig_1	cds	278658	278674	-	GLNHAC_03169	spoVID	stage VI sporulation protein D
contig_1	cds	278678	278714	-	GLNHAC_03170		hypothetical protein
contig_1	cds	278730	278883	-	GLNHAC_03171		hypothetical protein
contig_1	cds	278896	278919	-	GLNHAC_03172		hypothetical protein
contig_1	cds	278919	279025	-	GLNHAC_03173		Glutamate-1-semialdehyde 2,1-aminomutase
contig_1	cds	279028	279126	-	GLNHAC_03174	hemB	porphobilinogen synthase
contig_1	cds	279125	279204	-	GLNHAC_03175	hemD	uroporphyrinogen-III synthase
contig_1	cds	279203	279297	-	GLNHAC_03176	hemC	hydroxymethylbilane synthase
contig_1	cds	279301	279384	-	GLNHAC_03177	hemX	Protein HemX
contig_1	cds	279385	279526	-	GLNHAC_03178	hemA	glutamyl-tRNA reductase
contig_1	cds	279545	279594	+	GLNHAC_03179	ysxD	Uncharacterized membrane protein YsxD
contig_1	cds	279597	279655	-	GLNHAC_03180	yihA	ribosome biogenesis GTP-binding protein YihA/YsxC
contig_1	cds	279655	279888	-	GLNHAC_03181	lon	endopeptidase La
contig_1	cds	279906	280071	-	GLNHAC_03182	lonB	ATP-dependent protease LonB
contig_1	cds	280087	280213	-	GLNHAC_03183	clpX	ATP-dependent protease ATP-binding subunit ClpX
contig_1	cds	280240	280368	-	GLNHAC_03184	tig	trigger factor
contig_1	cds	280390	280491	-	GLNHAC_03185	ddcA	DNA damage checkpoint antagonist DdcA
contig_1	cds	280503	280528	-	GLNHAC_03186		(pseudo) 3-isopropylmalate dehydratase small subunit
contig_1	cds	280545	280562	-	GLNHAC_03187		hypothetical protein
contig_1	cds	280564	280608	-	GLNHAC_03188		hypothetical protein
contig_1	cds	280607	280648	-	GLNHAC_03189		hypothetical protein
contig_1	cds	280648	280665	-	GLNHAC_03190		hypothetical protein
contig_1	cds	280661	280690	-	GLNHAC_03191		aconitase family protein
contig_1	cds	280710	280820	-	GLNHAC_03192	leuB	3-isopropylmalate dehydrogenase
contig_1	cds	280822	280859	-	GLNHAC_03193	leuA	2-isopropylmalate synthase LeuA allosteric (dimerisation) dom
contig_1	cds	280864	280890	-	GLNHAC_03194		hypothetical protein
contig_1	cds	280886	280916	-	GLNHAC_03195		Pyruvate carboxyltransferase domain-containing protein
contig_1	cds	280989	281078	-	GLNHAC_03196	ilvC	ketol-acid reductoisomerase
contig_1	cds	281080	281132	-	GLNHAC_03197	ilvN	acetolactate synthase small subunit
contig_1	cds	281132	281294	-	GLNHAC_03198	ilvB	acetolactate synthase large subunit
contig_1	cds	281294	281304	-	GLNHAC_03199		hypothetical protein
contig_1	ncRNA-region	281316	281341	-			T-box leader
contig_1	cds	281386	281418	+	GLNHAC_03200		DNA helicase
contig_1	cds	281437	281483	+	GLNHAC_03201	ysnE	Uncharacterized N-acetyltransferase YsnE
contig_1	cds	281502	281584	+	GLNHAC_03202		YsnF/AvaK domain-containing protein
contig_1	tRNA	281591	281598	-	GLNHAC_03203	trnR	tRNA-Arg(tct)
contig_1	cds	281611	281662	-	GLNHAC_03204	ysnB	putative metallophosphoesterase YsnB
contig_1	cds	281663	281722	-	GLNHAC_03205	ysnA	XTP/dITP diphosphatase
contig_1	cds	281724	281798	-	GLNHAC_03206	rph	ribonuclease PH
contig_1	cds	281809	281919	-	GLNHAC_03207	gerM	spore germination protein GerM
contig_1	cds	281931	282012	-	GLNHAC_03208	racE	glutamate racemase
contig_1	cds	282013	282057	-	GLNHAC_03209	ysmB	Uncharacterized HTH-type transcriptional regulator YsmB
contig_1	cds	282082	282105	-	GLNHAC_03210	gerE	spore germination transcription factor GerE
contig_1	cds	282116	282160	-	GLNHAC_03211		Acyl-CoA thioesterase
contig_1	cds	282166	282243	-	GLNHAC_03212	sdhB	succinate dehydrogenase iron-sulfur subunit
contig_1	cds	282243	282419	-	GLNHAC_03213	sdhA	succinate dehydrogenase flavoprotein subunit
contig_1	cds	282422	282483	-	GLNHAC_03214	sdhC	succinate dehydrogenase cytochrome B558
contig_1	cds	282512	282557	+	GLNHAC_03215	yslB	Uncharacterized protein YslB
contig_1	cds	282561	282684	-	GLNHAC_03216	lysC	aspartate kinase
contig_1	ncRNA-region	282697	282715	-			Lysine riboswitch aptamer
contig_1	cds	282721	282898	-	GLNHAC_03217	uvrC	excinuclease ABC subunit UvrC
contig_1	cds	282912	282943	-	GLNHAC_03218	trxA	thioredoxin
contig_1	cds	282976	283114	-	GLNHAC_03219	abfB	alpha-L-arabinofuranosidase AbfB
contig_1	cds	283132	283148	+	GLNHAC_03220		hypothetical protein
contig_1	cds	283146	283205	-	GLNHAC_03221		hypothetical protein
contig_1	cds	283209	283243	-	GLNHAC_03222		electron transfer flavoprotein subunit alpha/FixB family protei
contig_1	cds	283247	283324	-	GLNHAC_03223	etfB	electron transfer flavoprotein subunit beta
contig_1	cds	283326	283403	-	GLNHAC_03224	fadB	enoyl-CoA hydratase
contig_1	cds	283405	283463	-	GLNHAC_03225	fadR	fatty acid metabolism transcriptional regulator FadR
contig_1	cds	283476	283643	-	GLNHAC_03226	lcfA	long-chain-fatty-acid--CoA ligase LcfA
contig_1	cds	283662	283688	-	GLNHAC_03227		DUF350 domain-containing protein
contig_1	cds	283685	283703	-	GLNHAC_03228		hypothetical protein
contig_1	cds	283704	283940	-	GLNHAC_03229	mutS2	endonuclease MutS2
contig_1	cds	283942	284113	-	GLNHAC_03230	polX	DNA polymerase/3'-5' exonuclease PolX
contig_1	cds	284120	284174	-	GLNHAC_03231	yshB	Uncharacterized transmembrane protein YshB
contig_1	cds	284174	284200	-	GLNHAC_03232	zapA	cell division protein ZapA
contig_1	cds	284214	284308	+	GLNHAC_03233	rnhC	ribonuclease HIII
contig_1	cds	284311	284326	-	GLNHAC_03234		hypothetical protein
contig_1	cds	284338	284373	-	GLNHAC_03235		hypothetical protein
contig_1	cds	284388	284553	-	GLNHAC_03236	pheT	(pseudo) phenylalanine--tRNA ligase subunit beta
contig_1	cds	284554	284658	-	GLNHAC_03237	pheS	phenylalanine--tRNA ligase subunit alpha
contig_1	cds	284693	284766	-	GLNHAC_03238	rlmP	23S rRNA (guanosine(2553)-2'-O)-methyltransferase RlmP
contig_1	cds	284779	284801	+	GLNHAC_03239	sspI	small acid-soluble spore protein SspI
contig_1	cds	284808	284820	+	GLNHAC_03240		(pseudo) sugar diacid recognition domain-containing protein
contig_1	cds	284819	284857	+	GLNHAC_03241		hypothetical protein
contig_1	cds	284857	284903	+	GLNHAC_03242		hypothetical protein
contig_1	cds	284903	284919	+	GLNHAC_03243		hypothetical protein
contig_1	cds	284929	284997	+	GLNHAC_03244		hypothetical protein
contig_1	cds	285006	285070	+	GLNHAC_03245		FAD-linked oxidase C-terminal domain-containing protein
contig_1	cds	285069	285203	+	GLNHAC_03246	glcF	putative glycolate oxidase iron-sulfur subunit
contig_1	cds	285207	285245	-	GLNHAC_03247		Glyoxalase family protein
contig_1	cds	285246	285426	-	GLNHAC_03248	cstA	carbon starvation protein CstA
contig_1	cds	285439	285526	-	GLNHAC_03249		DUF4097 domain-containing protein
contig_1	cds	285528	285583	-	GLNHAC_03250		DUF1700 domain-containing protein
contig_1	cds	285583	285616	-	GLNHAC_03251	padR	Transcriptional regulator, PadR family
contig_1	cds	285631	285781	-	GLNHAC_03252	abfA	alpha-L-arabinofuranosidase AbfA
contig_1	cds	285783	285867	-	GLNHAC_03253	araQ	arabinose ABC transporter permease AraQ
contig_1	cds	285867	285961	-	GLNHAC_03254	araP	Arabinooligosaccharides transport system permease protein A
contig_1	cds	285965	286097	-	GLNHAC_03255	araN	Arabinooligosaccharide-binding protein
contig_1	cds	286098	286217	-	GLNHAC_03256	egsA	sn-glycerol-1-phosphate dehydrogenase
contig_1	cds	286216	286297	-	GLNHAC_03257	araL	sugar-phosphatase AraL
contig_1	cds	286296	286365	-	GLNHAC_03258	araD	L-ribulose-5-phosphate 4-epimerase
contig_1	cds	286366	286535	-	GLNHAC_03259	araB	ribulokinase
contig_1	cds	286538	286685	-	GLNHAC_03260	araA	L-arabinose isomerase
contig_1	cds	286717	286815	-	GLNHAC_03261		Endo-alpha-(1-->5)-L-arabinanase
contig_1	cds	286830	286938	-	GLNHAC_03262	ysdC	Putative aminopeptidase YsdC
contig_1	cds	286958	286996	+	GLNHAC_03263	ysdB	sigma W pathway protein YsdB
contig_1	cds	286997	287024	-	GLNHAC_03264	ysdA	Uncharacterized protein YsdA
contig_1	cds	287030	287066	-	GLNHAC_03265	rplT	50S ribosomal protein L20
contig_1	cds	287090	287137	-	GLNHAC_03266	infC	translation initiation factor IF-3
contig_1	ncRNA-region	287148	287161	-			Ribosomal protein L20 leader
contig_1	cds	287206	287270	+	GLNHAC_03267		Lipoprotein
contig_1	cds	287273	287292	-	GLNHAC_03268		hypothetical protein
contig_1	cds	287295	287342	-	GLNHAC_03269	lrgB	(pseudo) antiholin-like protein LrgB
contig_1	cds	287344	287388	-	GLNHAC_03270	lrgA	antiholin-like murein hydrolase modulator LrgA
contig_1	cds	287402	287474	-	GLNHAC_03271	lytT	two-component system response regulator LytT
contig_1	cds	287472	287650	-	GLNHAC_03272	lytS	two-component system sensor histidine kinase LytS
contig_1	cds	287667	287745	+	GLNHAC_03273	serB	Phosphoserine phosphatase
contig_1	cds	287749	287806	-	GLNHAC_03274		threonine--tRNA ligase
contig_1	cds	287807	287942	-	GLNHAC_03275		Threonine--tRNA ligase
contig_1	cds	287982	288066	-	GLNHAC_03276	ytxC	putative sporulation protein YtxC
contig_1	cds	288074	288136	-	GLNHAC_03277	ytxB	TVP38/TMEM64 family membrane protein YtxB
contig_1	cds	288141	288235	-	GLNHAC_03278	dnaI	primosomal protein DnaI

contig_1	cds	288238	288380	-	GLNHAC_03279	dnaB	replication initiation membrane attachment protein DnaB
contig_1	cds	288391	288437	-	GLNHAC_03280	nrdR	transcriptional regulator NrdR
contig_1	cds	288464	288502	-	GLNHAC_03281	speD	adenosylmethionine decarboxylase
contig_1	cds	288526	288629	-	GLNHAC_03282	gapB	glyceraldehyde-3-phosphate dehydrogenase
contig_1	cds	288649	288687	-	GLNHAC_03283	ytcD	Uncharacterized HTH-type transcriptional regulator YtcD
contig_1	cds	288705	288824	+	GLNHAC_03284	ytbD	Uncharacterized MFS-type transporter YtbD
contig_1	cds	288827	288903	+	GLNHAC_03285	ytbE	Uncharacterized oxidoreductase YtbE
contig_1	cds	288915	288974	-	GLNHAC_03286	coaE	dephospho-CoA kinase
contig_1	cds	288976	289039	-	GLNHAC_03287	ytaF	sporulation membrane protein YtaF
contig_1	cds	289056	289139	-	GLNHAC_03288	mutM	DNA-formamidopyrimidine glycosylase
contig_1	cds	289141	289405	-	GLNHAC_03289	polA	DNA polymerase I
contig_1	cds	289429	289603	-	GLNHAC_03290	phoR	sensory box histidine kinase PhoR
contig_1	cds	289603	289675	-	GLNHAC_03291	phoP	two-component system response regulator PhoP
contig_1	cds	289696	289790	-	GLNHAC_03292	mdh	malate dehydrogenase
contig_1	cds	289794	289921	-	GLNHAC_03293	icd	NADP-dependent isocitrate dehydrogenase
contig_1	cds	289938	290049	-	GLNHAC_03294	citZ	citrate synthase
contig_1	cds	290083	290129	-	GLNHAC_03295	ytwI	UPF0756 membrane protein YtwI
contig_1	cds	290139	290250	+	GLNHAC_03296	ytvI	sporulation integral membrane protein YtvI
contig_1	cds	290254	290292	-	GLNHAC_03297	ytzA	UPF0716 protein YtzA
contig_1	cds	290301	290477	-	GLNHAC_03298	pyk	pyruvate kinase
contig_1	cds	290481	290577	-	GLNHAC_03299	pfkA	6-phosphofructokinase
contig_1	cds	290596	290693	-	GLNHAC_03300	accA	acetyl-CoA carboxylase carboxyl transferase subunit alpha
contig_1	cds	290692	290779	-	GLNHAC_03301	accD	acetyl-CoA carboxylase, carboxyltransferase subunit beta
contig_1	cds	290813	290936	-	GLNHAC_03302	maeB	NADP-dependent malic enzyme
contig_1	cds	290949	290992	-	GLNHAC_03303		hypothetical protein
contig_1	cds	290990	291284	-	GLNHAC_03304	dnaE	DNA polymerase III subunit alpha
contig_1	cds	291298	291332	+	GLNHAC_03305	ytrH	sporulation membrane protein YtrH
contig_1	cds	291332	291382	+	GLNHAC_03306	ytrI	sporulation membrane protein YtrI
contig_1	cds	291392	291406	+	GLNHAC_03307	ytzJ	(pseudo) Uncharacterized protein YtzJ
contig_1	cds	291443	291507	-	GLNHAC_03308		3'-to-5' oligoribonuclease A
contig_1	cds	291520	291550	+	GLNHAC_03309	ytpI	Uncharacterized membrane protein YtpI
contig_1	cds	291552	291684	-	GLNHAC_03310	ytoI	Uncharacterized protein YtoI
contig_1	cds	291696	291764	-	GLNHAC_03311	ytkL	metal-dependent hydrolase
contig_1	cds	291770	291847	-	GLNHAC_03312	ytkK	Putative oxidoreductase YtkK
contig_1	cds	291860	291873	-	GLNHAC_03313		DUF4025 domain-containing protein
contig_1	cds	291879	292004	-	GLNHAC_03314	argH	argininosuccinate lyase
contig_1	cds	292001	292018	-	GLNHAC_03315		(pseudo) Argininosuccinate lyase
contig_1	cds	292017	292036	-	GLNHAC_03316		hypothetical protein
contig_1	cds	292036	292138	-	GLNHAC_03317		Argininosuccinate synthase
contig_1	cds	292155	292201	-	GLNHAC_03318	moaB	Molybdenum cofactor biosynthesis protein B
contig_1	cds	292215	292238	-	GLNHAC_03319		Acetate kinase
contig_1	cds	292288	292333	-	GLNHAC_03320		Propionate kinase, propanediol utilization
contig_1	cds	292367	292466	-	GLNHAC_03321	ytxK	Uncharacterized protein YtxK
contig_1	cds	292472	292492	-	GLNHAC_03322		hypothetical protein
contig_1	cds	292493	292522	-	GLNHAC_03323	tpx	(pseudo) thiol peroxidase
contig_1	cds	292547	292579	-	GLNHAC_03324		hypothetical protein
contig_1	cds	292581	292649	-	GLNHAC_03325	ytfI	Uncharacterized protein YtfI
contig_1	cds	292656	292706	-	GLNHAC_03326	yteJ	Uncharacterized membrane protein YteJ
contig_1	cds	292707	292808	-	GLNHAC_03327	sppA	Putative signal peptide peptidase SppA
contig_1	cds	292829	292855	+	GLNHAC_03328		hypothetical protein
contig_1	cds	292854	292907	+	GLNHAC_03329		Inorganic polyphosphate/ATP-NAD kinase
contig_1	cds	292931	293069	-	GLNHAC_03330	ytcJ	Putative amidohydrolase YtcJ
contig_1	cds	293071	293230	-	GLNHAC_03331	mbcS	acyl-CoA synthetase MbcS
contig_1	cds	293248	293269	-	GLNHAC_03332	sspA	Small, acid-soluble spore protein A
contig_1	cds	293278	293396	-	GLNHAC_03333	thiI	putative tRNA sulfurtransferase
contig_1	cds	293399	293513	-	GLNHAC_03334	iscS2	Putative cysteine desulfurase IscS 2
contig_1	cds	293534	293666	+	GLNHAC_03335	brnQ	branched-chain amino acid transport system II carrier protein
contig_1	cds	293676	293848	-	GLNHAC_03336	ezrA	septation ring formation regulator EzrA
contig_1	cds	293866	293948	+	GLNHAC_03337	hisJ	histidinol-phosphatase HisJ
contig_1	cds	293948	294010	-	GLNHAC_03338	refZ	transcriptional regulator RefZ
contig_1	cds	294023	294072	+	GLNHAC_03339	ytsP	Protein YtsP
contig_1	cds	294076	294250	-	GLNHAC_03340	ytrP	Uncharacterized protein YtrP
contig_1	cds	294297	294326	+	GLNHAC_03341		30S ribosomal protein S4
contig_1	cds	294326	294339	+	GLNHAC_03342		(pseudo) S4 domain-containing protein
contig_1	cds	294341	294354	-	GLNHAC_03343	sr7p	small protein SR7P
contig_1	cds	294367	294414	-	GLNHAC_03344		hypothetical protein
contig_1	cds	294421	294494	-	GLNHAC_03345		Tyrosine--tRNA ligase
contig_1	ncRNA-region	294499	294522	-			T-box leader
contig_1	cds	294528	294700	-	GLNHAC_03346	acsA	acetate--CoA ligase
contig_1	cds	294716	294779	+	GLNHAC_03347	acuA	acetoin utilization protein acetyltransferase AcuA
contig_1	cds	294782	294846	+	GLNHAC_03348	acuB	Acetoin utilization protein AcuB
contig_1	cds	294846	294962	+	GLNHAC_03349	acuC	Acetoin utilization protein AcuC
contig_1	cds	294965	295036	-	GLNHAC_03350	motS	flagellar motor protein MotS
contig_1	cds	295035	295117	-	GLNHAC_03351	motP	flagellar motor protein MotP
contig_1	cds	295123	295224	-	GLNHAC_03352	ccpA	catabolite control protein A
contig_1	cds	295251	295318	-	GLNHAC_03353		Chorismate mutase I / 2-keto-3-deoxy-D-arabino-heptuloson
contig_1	cds	295321	295359	-	GLNHAC_03354		Chorismate mutase domain-containing protein
contig_1	cds	295383	295415	-	GLNHAC_03355	brxC	Monothiol bacilliredoxin BrxC
contig_1	cds	295418	295426	-	GLNHAC_03356		hypothetical protein
contig_1	cds	295431	295463	-	GLNHAC_03357	ytxH	(pseudo) YtxH domain-containing protein
contig_1	cds	295466	295508	-	GLNHAC_03358	ytxG	UPF0478 protein YtxG
contig_1	cds	295525	295654	-	GLNHAC_03359	murC	UDP-N-acetylmuramate--L-alanine ligase
contig_1	cds	295679	295909	-	GLNHAC_03360	ftsK	FtsK domain-containing protein
contig_1	cds	295908	295985	-	GLNHAC_03361		hypothetical protein
contig_1	cds	296001	296062	-	GLNHAC_03362	ytpR	YtpR family tRNA-binding protein
contig_1	cds	296063	296144	-	GLNHAC_03363	ytpQ	UPF0354 protein YtpQ
contig_1	cds	296146	296178	-	GLNHAC_03364	ytpP	Thioredoxin-like protein YtpP
contig_1	cds	296201	296246	+	GLNHAC_03365	ytoQ	Uncharacterized protein YtoQ
contig_1	cds	296252	296359	-	GLNHAC_03366	ytoP	Putative aminopeptidase YtoP
contig_1	cds	296375	296407	+	GLNHAC_03367	ytzB	Uncharacterized protein YtzB
contig_1	cds	296412	296582	-	GLNHAC_03368	malS	NAD-dependent malic enzyme 3
contig_1	cds	296591	296650	-	GLNHAC_03369		Metallo-beta-lactamase domain-containing protein
contig_1	cds	296656	296675	-	GLNHAC_03370		Metal-dependent hydrolase
contig_1	cds	296682	296746	-	GLNHAC_03371	trmB	tRNA (guanosine(46)-N7)-methyltransferase TrmB
contig_1	cds	296767	296794	+	GLNHAC_03372	ytzH	Uncharacterized protein YtzH
contig_1	cds	296795	296874	-	GLNHAC_03373	ccrZ	Cell cycle regulator CcrZ
contig_1	cds	296892	297108	-	GLNHAC_03374	pulA	type I pullulanase
contig_1	cds	297110	297182	-	GLNHAC_03375	ytlR	(pseudo) Putative lipid kinase YtlR
contig_1	cds	297181	297203	-	GLNHAC_03376		acylglycerol kinase family protein
contig_1	cds	297208	297300	-	GLNHAC_03377	ytlQ	Uncharacterized protein YtlQ
contig_1	cds	297302	297357	-	GLNHAC_03378	thpR	RNA 2',3'-cyclic phosphodiesterase
contig_1	cds	297396	297466	+	GLNHAC_03379		Cysteine synthase
contig_1	cds	297469	297494	-	GLNHAC_03380		Acetylornithine deacetylase/succinyl-diaminopimelate desucc
contig_1	cds	297492	297608	-	GLNHAC_03381	ytjP	Putative dipeptidase YtjP
contig_1	cds	297618	297747	+	GLNHAC_03382	pbuO	hypoxanthine/guanine permease PbuO
contig_1	cds	297764	297866	-	GLNHAC_03383		ABC transporter permease
contig_1	cds	297866	297937	-	GLNHAC_03384	ythP	Uncharacterized ABC transporter ATP-binding protein YthP
contig_1	cds	297966	297988	+	GLNHAC_03385	ytzE	Uncharacterized HTH-type transcriptional regulator YtzE
contig_1	cds	298000	298072	-	GLNHAC_03386	ytzG	Uncharacterized RNA pseudouridine synthase YtzG
contig_1	cds	298079	298242	-	GLNHAC_03387	murJ	lipid II flippase MurJ
contig_1	cds	298267	298364	+	GLNHAC_03388		BaiN-like insert domain-containing protein
contig_1	cds	298408	298562	+	GLNHAC_03389	opuD	Glycine betaine transporter OpuD
contig_1	cds	298565	298584	-	GLNHAC_03390	cse60	Sporulation protein cse60
contig_1	cds	298590	298621	-	GLNHAC_03391		(pseudo) DUF624 domain-containing protein
contig_1	cds	298615	298657	-	GLNHAC_03392		Integral inner membrane protein
contig_1	cds	298674	298701	-	GLNHAC_03393		hypothetical protein
contig_1	cds	298697	298737	-	GLNHAC_03394		hypothetical protein
contig_1	cds	298737	298781	-	GLNHAC_03395		(pseudo) Gfo/Idh/MocA family oxidoreductase
contig_1	cds	298800	298831	-	GLNHAC_03396		hypothetical protein
contig_1	cds	298839	298950	-	GLNHAC_03397	yteR	unsaturated rhamnogalacturonyl hydrolase
contig_1	cds	298952	299048	-	GLNHAC_03398	yteP	Polygalacturonan/rhamnogalacturonan transport system perm
contig_1	cds	299071	299174	+	GLNHAC_03399		Cache domain-containing protein
contig_1	cds	299169	299301	+	GLNHAC_03400		helix-turn-helix domain-containing protein
contig_1	cds	299306	299455	-	GLNHAC_03401	ytcQ	Polygalacturonan/rhamnogalacturonan-binding protein YtcQ
contig_1	cds	299458	299563	-	GLNHAC_03402	ytcP	Polygalacturonan/rhamnogalacturonan transport system perm
contig_1	cds	299564	299640	-	GLNHAC_03403	ytbQ	Uncharacterized protein YtbQ
contig_1	cds	299648	299743	-	GLNHAC_03404		Cytochrome P450
contig_1	cds	299743	299767	-	GLNHAC_03405		hypothetical protein
contig_1	cds	299780	299812	-	GLNHAC_03406		Biotin and thiamin synthesis-associated domain-containing p
contig_1	cds	299812	299874	-	GLNHAC_03407	bioB	(pseudo) biotin synthase BioB
contig_1	cds	299875	299944	-	GLNHAC_03408	bioD	dethiobiotin synthase
contig_1	cds	299944	300061	-	GLNHAC_03409	bioF	8-amino-7-oxononanoate synthase
contig_1	cds	300060	300194	-	GLNHAC_03410	bioA	adenosylmethionine--8-amino-7-oxononanoate transaminase
contig_1	cds	300193	300267	-	GLNHAC_03411	bioW	6-carboxyhexanoate--CoA ligase
contig_1	cds	300291	300341	-	GLNHAC_03412		DUF2127 domain-containing protein
contig_1	cds	300346	300436	-	GLNHAC_03413		Dienelactone hydrolase family protein
contig_1	cds	300458	300561	+	GLNHAC_03414	lacI	LacI family DNA-binding transcriptional regulator
contig_1	cds	300565	300693	+	GLNHAC_03415	melE	Melibiose/raffinose/stachyose-binding protein MelE
contig_1	cds	300692	300783	+	GLNHAC_03416	melD	Melibiose/raffinose/stachyose import permease protein MelD

**contig_1**	cds	300783	300866	+	GLNHAC_03417	melC	Melibiose/raffinose/stachyose import permease protein MelC
**contig_1**	cds	300868	300998	+	GLNHAC_03418	melA	alpha-galactosidase MelA
**contig_1**	cds	301009	301042	+	GLNHAC_03419	tnp	IS3 family ISBsu1 transposase ORF A
**contig_1**	cds	301044	301124	+	GLNHAC_03420	tnp	IS3 family ISBsu1 transposase ORF B
**contig_1**	ncRNA	301104	301114	-	GLNHAC_03421	rli23	Listeria snRNA rli23
**contig_1**	cds	301129	301160	-	GLNHAC_03422	ytwF	Putative sulfurtransferase YtwF
**contig_1**	cds	301172	301413	-	GLNHAC_03423	leuS	leucine--tRNA ligase
**contig_1**	ncRNA-region	301424	301446	-			T-box leader
**contig_1**	cds	301456	301490	-	GLNHAC_03424	ytvB	Uncharacterized protein YtvB
**contig_1**	cds	301529	301607	+	GLNHAC_03425	pfyP	blue-light photoreceptor
**contig_1**	cds	301610	301730	-	GLNHAC_03426	yttB	Uncharacterized MFS-type transporter YttB
**contig_1**	cds	301749	301823	+	GLNHAC_03427	yttA	Uncharacterized membrane protein YttA
**contig_1**	cds	301827	301875	-	GLNHAC_03428	bceB	Bacitracin export permease protein BceB
**contig_1**	cds	301883	302021	-	GLNHAC_03429	bceB	Bacitracin export permease protein BceB
**contig_1**	cds	302020	302096	-	GLNHAC_03430	bceA	bacitracin ABC transporter ATP-binding protein BceA
**contig_1**	cds	302106	302200	-	GLNHAC_03431	bceS	two-component sensor histidine kinase BceS
**contig_1**	cds	302206	302276	-	GLNHAC_03432	bceR	two-component response regulator BceR
**contig_1**	cds	302285	302416	-	GLNHAC_03433	ytrF	ABC transporter permease YtrF
**contig_1**	cds	302415	302485	-	GLNHAC_03434	ytrE	ABC transporter ATP-binding protein YtrE
**contig_1**	cds	302486	302584	-	GLNHAC_03435		ABC transporter permease
**contig_1**	cds	302587	302685	-	GLNHAC_03436	ytrC	ABC transporter permease YtrC
**contig_1**	cds	302685	302773	-	GLNHAC_03437	ytrB	ABC transporter ATP-binding protein YtrB
**contig_1**	cds	302772	302811	-	GLNHAC_03438	ytrA	HTH-type transcriptional repressor YtrA
**contig_1**	cds	302814	302828	-	GLNHAC_03439		Lipoprotein
**contig_1**	cds	302844	302871	-	GLNHAC_03440	ytzC	Uncharacterized protein YtzC
**contig_1**	cds	302887	302984	+	GLNHAC_03441	ytqA	TIGR01212 family radical SAM protein
**contig_1**	cds	302984	303042	+	GLNHAC_03442	mnmM	tRNA (mnm(5)s(2)U34)-methyltransferase
**contig_1**	cds	303041	303152	-	GLNHAC_03443	ytpB	tetraprenyl-beta-curcumene synthase
**contig_1**	cds	303154	303232	-	GLNHAC_03444	ytpA	phospholipase YtpA
**contig_1**	cds	303236	303288	+	GLNHAC_03445	ytoA	Uncharacterized transferase YtoA
**contig_1**	cds	303314	303453	-	GLNHAC_03446	ytnA	Uncharacterized amino acid permease YtnA
**contig_1**	cds	303467	303539	-	GLNHAC_03447		asparagine synthase (glutamine-hydrolyzing)
**contig_1**	cds	303535	303620	-	GLNHAC_03448	asnB	(pseudo) asparagine synthase (glutamine-hydrolyzing)
**contig_1**	cds	303618	303656	-	GLNHAC_03449		asparagine synthase (glutamine-hydrolyzing)
**contig_1**	cds	303706	303791	-	GLNHAC_03450	metK	(pseudo) methionine adenosyltransferase
**contig_1**	ncRNA-region	303808	303822	-			SAM riboswitch aptamer (S box leader)
**contig_1**	cds	303842	303854	+	GLNHAC_03451		hypothetical protein
**contig_1**	cds	303860	304000	+	GLNHAC_03452	pckA	phosphoenolpyruvate carboxykinase (ATP)
**contig_1**	cds	304004	304028	-	GLNHAC_03453	ytmB	Uncharacterized protein YtmB
**contig_1**	cds	304033	304110	-	GLNHAC_03454	ytmA	Uncharacterized peptidase YtmA
**contig_1**	cds	304123	304226	+	GLNHAC_03455	ytlA	Putative binding protein YtlA
**contig_1**	cds	304227	304305	+	GLNHAC_03456	ytlC	Uncharacterized ABC transporter ATP-binding protein YtlC
**contig_1**	cds	304303	304363	+	GLNHAC_03457		ABC transmembrane type-1 domain-containing protein
**contig_1**	cds	304371	304384	+	GLNHAC_03458		hypothetical protein
**contig_1**	cds	304387	304434	-	GLNHAC_03459	ytkD	RNA deprotection pyrophosphohydrolase
**contig_1**	cds	304455	304496	-	GLNHAC_03460	ytkC	Uncharacterized protein YtkC
**contig_1**	cds	304513	304556	-	GLNHAC_03461	dps	General stress protein 20U
**contig_1**	cds	304569	304583	+	GLNHAC_03462	ytzI	YtzI protein
**contig_1**	cds	304583	304626	-	GLNHAC_03463	fixH	FixH family protein
**contig_1**	cds	304639	304686	-	GLNHAC_03464	luxS	S-ribosylhomocysteine lyase
**contig_1**	cds	304721	304778	-	GLNHAC_03465	ytiB	Putative carbonic anhydrase YtiB
**contig_1**	cds	304790	304814	-	GLNHAC_03466	rpmE2	type B 50S ribosomal protein L31
**contig_1**	cds	304835	304968	+	GLNHAC_03467		Cytochrome d ubiquinol oxidase subunit I
**contig_1**	cds	304972	305076	+	GLNHAC_03468	ythB	Putative cytochrome bd menaquinol oxidase subunit II
**contig_1**	cds	305082	305098	+	GLNHAC_03469	ytzL	Uncharacterized protein YtzL
**contig_1**	cds	305104	305188	-	GLNHAC_03470	mntD	Manganese transport system membrane protein MntD
**contig_1**	cds	305187	305319	-	GLNHAC_03471	mntC	Manganese transport system membrane protein MntC
**contig_1**	cds	305318	305394	-	GLNHAC_03472	mntB	manganese ABC transporter ATP-binding protein MntB
**contig_1**	cds	305395	305487	-	GLNHAC_03473	mntA	manganese ABC transporter substrate-binding protein/adhes
**contig_1**	cds	305516	305627	-	GLNHAC_03474	menC	o-succinylbenzoate synthase
**contig_1**	cds	305627	305773	-	GLNHAC_03475	menE	o-succinylbenzoate--CoA ligase
**contig_1**	cds	305782	305864	-	GLNHAC_03476	menB	1,4-dihydroxy-2-naphthoyl-CoA synthase
**contig_1**	cds	305867	305949	-	GLNHAC_03477	menH	2-succinyl-6-hydroxy-2,4-cyclohexadiene-1-carboxylate synt
**contig_1**	cds	305948	306122	-	GLNHAC_03478	menD	2-succinyl-5-enolpyruvyl-6-hydroxy-3-cyclohexene-1-carbox
**contig_1**	cds	306122	306282	-	GLNHAC_03479	menF	Isochorismate synthase MenF
**contig_1**	cds	306293	306365	+	GLNHAC_03480	yteA	Uncharacterized protein YteA
**contig_1**	cds	306465	306593	+	GLNHAC_03481	ytcA	Putative UDP-glucose 6-dehydrogenase YtcA
**contig_1**	cds	306593	306688	+	GLNHAC_03482	ytcB	Uncharacterized UDP-glucose epimerase YtcB
**contig_1**	cds	306688	306810	+	GLNHAC_03483	ytcC	Putative glycosyltransferase YtcC
**contig_1**	cds	306818	306860	-	GLNHAC_03484		Spore coat protein
**contig_1**	cds	306860	306966	-	GLNHAC_03485	cotS	spore coat protein CotS
**contig_1**	cds	306967	307081	-	GLNHAC_03486	cotSA	spore coat protein CotSA
**contig_1**	cds	307101	307207	+	GLNHAC_03487	cotI	spore coat kinase CotI
**contig_1**	cds	307216	307263	+	GLNHAC_03488	ytaB	Uncharacterized membrane protein YtaB
**contig_1**	cds	307266	307505	-	GLNHAC_03489	glgP	glycogen phosphorylase GlgP
**contig_1**	cds	307504	307564	-	GLNHAC_03490	glgA	(pseudo) glycogen synthase GlgA
**contig_1**	cds	307571	307649	-	GLNHAC_03491		starch synthase
**contig_1**	cds	307649	307752	-	GLNHAC_03492	glgD	glucose-1-phosphate adenylyltransferase subunit GlgD
**contig_1**	cds	307754	307792	-	GLNHAC_03493		hypothetical protein
**contig_1**	cds	307791	307844	-	GLNHAC_03494	glgC	(pseudo) glucose-1-phosphate adenylyltransferase
**contig_1**	cds	307853	307868	-	GLNHAC_03495		Nucleotidyl transferase
**contig_1**	cds	307868	308056	-	GLNHAC_03496	glgB	1,4-alpha-glucan branching enzyme
**contig_1**	tRNA	308080	308087	-	GLNHAC_03497	trnE	tRNA-Glu(ttc)
**contig_1**	tRNA	308090	308099	-	GLNHAC_03498	trnS	tRNA-Ser(gct)
**contig_1**	tRNA	308099	308107	-	GLNHAC_03499	trnN	tRNA-Asn(gtt)
**contig_1**	tRNA	308108	308116	-	GLNHAC_03500	trnI	tRNA-Ile(gat)
**contig_1**	tRNA	308117	308125	-	GLNHAC_03501	trnG	tRNA-Gly(tcc)
**contig_1**	tRNA	308126	308133	-	GLNHAC_03502	trnH	tRNA-His(gtg)
**contig_1**	tRNA	308135	308143	-	GLNHAC_03503	trnF	tRNA-Phe(gaa)
**contig_1**	tRNA	308144	308151	-	GLNHAC_03504	trnD	tRNA-Asp(gtc)
**contig_1**	tRNA	308158	308166	-	GLNHAC_03505	trnfM	tRNA-fMet(cat)
**contig_1**	tRNA	308168	308177	-	GLNHAC_03506	trnS	tRNA-Ser(tga)
**contig_1**	tRNA	308178	308185	-	GLNHAC_03507	trnI	tRNA-Ile2(cat)
**contig_1**	tRNA	308186	308193	-	GLNHAC_03508	trnM	tRNA-Met(cat)
**contig_1**	tRNA	308195	308203	-	GLNHAC_03509	trnA	tRNA-Ala(tgc)
**contig_1**	tRNA	308203	308211	-	GLNHAC_03510	trnP	tRNA-Pro(tgg)
**contig_1**	tRNA	308212	308220	-	GLNHAC_03511	trnR	tRNA-Arg(acg)
**contig_1**	tRNA	308221	308229	-	GLNHAC_03512	trnL	tRNA-Leu(taa)
**contig_1**	tRNA	308231	308238	-	GLNHAC_03513	trnG	tRNA-Gly(gcc)
**contig_1**	tRNA	308239	308248	-	GLNHAC_03514	trnL	tRNA-Leu(cag)
**contig_1**	tRNA	308249	308256	-	GLNHAC_03515	trnK	tRNA-Lys(ttt)
**contig_1**	tRNA	308260	308267	-	GLNHAC_03516	trnT	tRNA-Thr(tgt)
**contig_1**	tRNA	308271	308278	-	GLNHAC_03517	trnV	tRNA-Val(tac)
**contig_1**	rRNA	308281	308292	-	GLNHAC_03518	rrf	5S ribosomal RNA
**contig_1**	rRNA	308297	308590	-	GLNHAC_03519	rrl	23S ribosomal RNA
**contig_1**	rRNA	308607	308762	-	GLNHAC_03520	rrs	16S ribosomal RNA
**contig_1**	ncRNA	308761	308768	+	GLNHAC_03521	Bacillaceae-1	Bacillaceae-1 RNA
**contig_1**	ncRNA-region	308808	308818	+			TPP riboswitch aptamer (THI element)
**contig_1**	cds	308828	308886	+	GLNHAC_03522	thiT	energy-coupled thiamine transporter ThiT
**contig_1**	cds	308890	308942	-	GLNHAC_03523	yuaI	Uncharacterized N-acetyltransferase YuaI
**contig_1**	cds	308945	309098	-	GLNHAC_03524	floT	flotillin lipid rafts scaffold protein FloT
**contig_1**	cds	309100	309152	-	GLNHAC_03525	nfeD2	Membrane protein NfeD2
**contig_1**	cds	309169	309218	+	GLNHAC_03526	yuaE	Uncharacterized protein YuaE
**contig_1**	cds	309240	309277	-	GLNHAC_03527		(pseudo) MOSC domain-containing protein
**contig_1**	cds	309285	309373	-	GLNHAC_03528		iron-containing alcohol dehydrogenase
**contig_1**	cds	309370	309406	-	GLNHAC_03529		hypothetical protein
**contig_1**	cds	309408	309555	-	GLNHAC_03530	betB	betaine-aldehyde dehydrogenase
**contig_1**	cds	309575	309629	+	GLNHAC_03531	gbsR	transcriptional regulator GbsR
**contig_1**	cds	309650	309679	+	GLNHAC_03532		hypothetical protein
**contig_1**	cds	309679	309704	+	GLNHAC_03533		BslA/BslB family hydrophobin
**contig_1**	ncRNA-region	309719	309733	+			c-di-AMP riboswitch aptamer (ydaO/yuaA leader)
**contig_1**	cds	309741	309807	+	GLNHAC_03534	ktrA	potassium uptake protein KtrA
**contig_1**	cds	309808	309942	+	GLNHAC_03535	ktrB	Ktr system potassium transporter KtrB
**contig_1**	cds	309945	309972	-	GLNHAC_03536	yubF	Uncharacterized membrane protein YubF
**contig_1**	cds	309983	310067	-	GLNHAC_03537	lytG	exo-glucosaminidase LytG
**contig_1**	cds	310083	310237	-	GLNHAC_03538	yubD	Uncharacterized MFS-type transporter YubD
**contig_1**	cds	310283	310331	+	GLNHAC_03539	cdoA	cysteine dioxygenase
**contig_1**	tRNA	310347	310354	-	GLNHAC_03540	trnA	tRNA-Ala(ggc)
**contig_1**	cds	310365	310448	-	GLNHAC_03541	uppP	undecaprenyl-diphosphate phosphatase
**contig_1**	cds	310457	310574	-	GLNHAC_03542	yubA	Putative transport protein YubA
**contig_1**	cds	310592	310690	+	GLNHAC_03543	iolU	scyllo-inositol 2-dehydrogenase
**contig_1**	cds	310695	310822	-	GLNHAC_03544	rhaA	L-rhamnose isomerase
**contig_1**	cds	310825	310856	-	GLNHAC_03545	rhaM	L-rhamnose mutarotase
**contig_1**	cds	310857	311003	-	GLNHAC_03546	rhaB	rhamnulokinase
**contig_1**	cds	311004	311082	-	GLNHAC_03547	rhaR	rhamnose catabolism operon transcriptional regulator RhaR
**contig_1**	cds	311087	311294	-	GLNHAC_03548	yuxG	bifunctional aldolase/short-chain dehydrogenase
**contig_1**	cds	311308	311507	-	GLNHAC_03549	tlpB	Methyl-accepting chemotaxis protein TlpB
**contig_1**	cds	311518	311716	-	GLNHAC_03550	mcpA	Methyl-accepting chemotaxis protein McpA
**contig_1**	cds	311729	311928	-	GLNHAC_03551	tlpA	Methyl-accepting chemotaxis protein TlpA

contig_1	cds	311945	312144	-	GLNHAC_03552	mcpB	methyl-accepting chemotaxis protein McpB
contig_1	cds	312160	312234	+	GLNHAC_03553	tgl	protein-glutamine gamma-glutamyltransferase
contig_1	cds	312235	312261	-	GLNHAC_03554	yuzH	Uncharacterized protein YuzH
contig_1	cds	312263	312366	-	GLNHAC_03555		putative nitronate monooxygenase
contig_1	cds	312368	312407	-	GLNHAC_03556	yugU	UPF0047 protein YugU
contig_1	cds	312419	312586	-	GLNHAC_03557	yugT	putative oligo-1,6-glucosidase 3
contig_1	cds	312598	312662	-	GLNHAC_03558		Hemolysins and related proteins containing CBS domains
contig_1	cds	312663	312727	-	GLNHAC_03559	yugS	(pseudo) UPF0053 protein YugS
contig_1	cds	312732	312800	-	GLNHAC_03560	yugP	Putative membrane protease YugP
contig_1	cds	312804	312830	-	GLNHAC_03561	yuzI	Uncharacterized membrane protein YuzI
contig_1	cds	312835	312861	+	GLNHAC_03562	mstX	(pseudo) Protein mistic
contig_1	cds	312859	312868	+	GLNHAC_03563		hypothetical protein
contig_1	cds	312868	312967	+	GLNHAC_03564	yugO	Putative potassium channel protein YugO
contig_1	cds	312966	313007	-	GLNHAC_03565		YugN-like family protein
contig_1	cds	313013	313055	-	GLNHAC_03566		Transporter
contig_1	cds	313056	313191	-	GLNHAC_03567	pgi	glucose-6-phosphate isomerase
contig_1	cds	313202	313242	-	GLNHAC_03568		NADH-dependent alcohol dehydrogenase
contig_1	cds	313282	313319	-	GLNHAC_03569	gldA	Alcohol dehydrogenase iron-type/glycerol dehydrogenase Glc
contig_1	cds	313330	313416	-	GLNHAC_03570	gldA	Alcohol dehydrogenase iron-type/glycerol dehydrogenase Glc
contig_1	cds	313412	313440	-	GLNHAC_03571		iron-containing alcohol dehydrogenase
contig_1	cds	313468	313492	+	GLNHAC_03572	yuzA	Uncharacterized membrane protein YuzA
contig_1	cds	313513	313552	-	GLNHAC_03573	yugI	S1 domain-containing post-transcriptional regulator GSP13
contig_1	ncRNA	313566	313572	+	GLNHAC_03574	ncr1241	Bacillus sRNA ncr1241
contig_1	cds	313572	313688	-	GLNHAC_03575	yugH	aminotransferase
contig_1	cds	313688	313738	-	GLNHAC_03576	yugG	Uncharacterized HTH-type transcriptional regulator YugG
contig_1	cds	313780	313835	+	GLNHAC_03577		Hydrolase
contig_1	cds	313838	313864	-	GLNHAC_03578	yugE	Uncharacterized protein YugE
contig_1	cds	313872	313897	+	GLNHAC_03579		hypothetical protein
contig_1	cds	313894	313927	+	GLNHAC_03580		hypothetical protein
contig_1	cds	313928	313988	+	GLNHAC_03581	patB	Cystathionine beta-lyase, Bsu PatB
contig_1	cds	314001	314130	+	GLNHAC_03582	kinB	sporulation sensor histidine kinase KinB
contig_1	cds	314135	314169	+	GLNHAC_03583	kapB	Kinase-associated lipoprotein B
contig_1	cds	314176	314228	-	GLNHAC_03584	kapD	3'-5' exonuclease KapD
contig_1	cds	314270	314376	+	GLNHAC_03585	yuxJ	Uncharacterized MFS-type transporter YuxJ
contig_1	cds	314385	314573	+	GLNHAC_03586	pbpD	Penicillin-binding protein 4
contig_1	cds	314575	314616	+	GLNHAC_03587	yuxK	Uncharacterized protein YuxK
contig_1	cds	314618	314673	-	GLNHAC_03588	yufK	Uncharacterized membrane protein YufK
contig_1	cds	314691	314851	+	GLNHAC_03589	maeL	two-component system sensor histidine kinase MaeL
contig_1	cds	314851	314921	+	GLNHAC_03590	maeM	two-component system response regulator MaeM
contig_1	cds	314971	315077	+	GLNHAC_03591	nupN	guanosine ABC transporter substrate-binding protein NupN
contig_1	cds	315084	315237	+	GLNHAC_03592	nupO	Guanosine import ATP-binding protein NupO
contig_1	cds	315237	315266	+	GLNHAC_03593		hypothetical protein
contig_1	cds	315266	315327	+	GLNHAC_03594		ABC transporter permease
contig_1	cds	315341	315437	+	GLNHAC_03595	nupQ	guanosine ABC transporter permease NupQ
contig_1	cds	315453	315587	+	GLNHAC_03596	maeN	sodium/malate symporter MaeN
contig_1	cds	315591	315613	-	GLNHAC_03597		Bacteriocin
contig_1	cds	315636	315876	+	GLNHAC_03598	mrpA	Na+/H+ antiporter subunit A
contig_1	cds	315875	315912	+	GLNHAC_03599		hypothetical protein
contig_1	cds	315919	315953	+	GLNHAC_03600	mrpC	Na(+)/H(+) antiporter subunit C
contig_1	cds	315951	315989	+	GLNHAC_03601		hypothetical protein
contig_1	cds	315986	316100	+	GLNHAC_03602	mrpD	(pseudo) Na+/H+ antiporter subunit D
contig_1	cds	316101	316148	+	GLNHAC_03603	mrpE	Na+/H+ antiporter subunit E
contig_1	cds	316148	316177	+	GLNHAC_03604	mrpF	Na(+)/H(+) antiporter subunit F1
contig_1	cds	316175	316212	+	GLNHAC_03605	mnhG	monovalent cation/H(+) antiporter subunit G
contig_1	cds	316216	316254	-	GLNHAC_03606	comA2	Putative esterase ComA2
contig_1	cds	316256	316320	-	GLNHAC_03607	comA	two-component system response regulator ComA
contig_1	cds	316328	316560	-	GLNHAC_03608	comP	Sensor histidine kinase ComP
contig_1	cds	316561	316583	-	GLNHAC_03609	comX	ComX pheromone
contig_1	cds	316583	316673	-	GLNHAC_03610	comQ	Competence regulatory protein ComQ
contig_1	cds	316688	316702	-	GLNHAC_03611	degQ	degradation enzyme regulation protein DegQ
contig_1	cds	316749	316785	+	GLNHAC_03612		Inner spore coat protein
contig_1	cds	316783	316841	-	GLNHAC_03613	yuxH	(pseudo) Uncharacterized protein YuxH
contig_1	cds	316856	316906	-	GLNHAC_03614		EAL domain-containing protein
contig_1	cds	316919	317067	-	GLNHAC_03615	pncB	nicotinate phosphoribosyltransferase
contig_1	cds	317068	317123	-	GLNHAC_03616	pncA	Uncharacterized isochorismatase family protein PncA
contig_1	cds	317133	317173	-	GLNHAC_03617		DUF1694 domain-containing protein
contig_1	cds	317180	317205	-	GLNHAC_03618	yueH	Uncharacterized protein YueH
contig_1	cds	317212	317234	-	GLNHAC_03619		Conserved domain protein
contig_1	cds	317240	317351	-	GLNHAC_03620	yueF	Putative transport protein YueF
contig_1	cds	317362	317401	+	GLNHAC_03621	yuzE	Uncharacterized protein YuzE
contig_1	cds	317405	317429	-	GLNHAC_03622	yuzF	Uncharacterized protein YuzF
contig_1	cds	317448	317501	-	GLNHAC_03623	yueE	Putative phosphohydrolase YueE
contig_1	cds	317531	317593	-	GLNHAC_03624	yueD	(pseudo) (S)-benzoin forming benzil reductase
contig_1	cds	317600	317646	-	GLNHAC_03625	essA	type VII secretion protein EssA
contig_1	cds	317648	317700	-	GLNHAC_03626		hypothetical protein
contig_1	cds	317697	317790	-	GLNHAC_03627		hypothetical protein
contig_1	cds	317792	317970	-	GLNHAC_03628		hypothetical protein
contig_1	cds	317969	318027	-	GLNHAC_03629	ftsK	FtsK domain-containing protein
contig_1	cds	318033	318096	-	GLNHAC_03630		hypothetical protein
contig_1	cds	318096	318183	-	GLNHAC_03631		hypothetical protein
contig_1	cds	318181	318418	-	GLNHAC_03632	essC	type VII secretion protein EssC
contig_1	cds	318445	318557	-	GLNHAC_03633	essB	type VII secretion protein EssB
contig_1	cds	318558	318582	-	GLNHAC_03634	yukD	ESX secretion system protein YukD
contig_1	cds	318589	318618	-	GLNHAC_03635	yukE	Protein YukE
contig_1	cds	318675	318725	+	GLNHAC_03636	pucR	PucR family transcriptional regulator
contig_1	cds	318727	318762	+	GLNHAC_03637		hypothetical protein
contig_1	cds	318807	318821	+	GLNHAC_03638		hypothetical protein
contig_1	cds	318827	318846	+	GLNHAC_03639		hypothetical protein
contig_1	cds	318848	318921	+	GLNHAC_03640	ald	alanine dehydrogenase
contig_1	cds	318932	319000	+	GLNHAC_03641	yukJ	Uncharacterized protein YukJ
contig_1	cds	319004	319025	-	GLNHAC_03642	ybdZ	Uncharacterized protein YbdZ
contig_1	cds	319027	319397	-	GLNHAC_03643		Amino acid adenylation domain-containing protein
contig_1	cds	319405	319440	-	GLNHAC_03644		acyl carrier protein
contig_1	cds	319440	319534	-	GLNHAC_03645		non-ribosomal peptide synthetase
contig_1	cds	319534	319740	-	GLNHAC_03646		condensation domain-containing protein
contig_1	cds	319742	319836	-	GLNHAC_03647	dhbB	Isochorismatase
contig_1	cds	319839	319902	-	GLNHAC_03648		2,3-dihydroxybenzoate-AMP ligase [enterobactin] siderophore
contig_1	cds	319898	319925	-	GLNHAC_03649		hypothetical protein
contig_1	cds	319930	319990	-	GLNHAC_03650		hypothetical protein
contig_1	cds	319984	320001	-	GLNHAC_03651		(pseudo) AMP-binding protein
contig_1	cds	320000	320034	-	GLNHAC_03652		hypothetical protein
contig_1	cds	320029	320124	-	GLNHAC_03653		isochorismate synthase
contig_1	cds	320126	320205	-	GLNHAC_03654	dhbA	2,3-dihydro-2,3-dihydroxybenzoate dehydrogenase
contig_1	cds	320225	320313	-	GLNHAC_03655	besA	ferri-bacillibactin esterase BesA
contig_1	cds	320333	320385	-	GLNHAC_03656	yuiH	Uncharacterized oxidoreductase YuiH
contig_1	cds	320403	320463	+	GLNHAC_03657	bioYB	Putative biotin transporter BioYB
contig_1	cds	320470	320603	-	GLNHAC_03658	yuiF	Putative amino acid transporter YuiF
contig_1	cds	320617	320768	-	GLNHAC_03659	pepA	leucyl aminopeptidase
contig_1	cds	320783	320831	+	GLNHAC_03660	yuiD	Uncharacterized membrane protein YuiD
contig_1	cds	320834	320899	-	GLNHAC_03661	yuiC	stationary phase survival protein SpsC
contig_1	cds	320910	320942	-	GLNHAC_03662	yuiB	Uncharacterized membrane protein YuiB
contig_1	cds	320979	321101	-	GLNHAC_03663	yumB	NADH dehydrogenase-like protein YumB
contig_1	cds	321134	321234	+	GLNHAC_03664	yumC	Ferredoxin--NADP reductase 2
contig_1	cds	321238	321252	-	GLNHAC_03665		DUF4044 domain-containing protein
contig_1	cds	321279	321377	+	GLNHAC_03666	guaC	GMP reductase
contig_1	cds	321385	321447	-	GLNHAC_03667	paiB	protease synthase/sporulation negative transcriptional regulat
contig_1	cds	321449	321501	-	GLNHAC_03668		Protease synthase and sporulation negative regulatory protein
contig_1	cds	321556	321592	-	GLNHAC_03669	sufA	Uncharacterized protein SufA
contig_1	cds	321600	321685	-	GLNHAC_03670	dapF	diaminopimelate epimerase
contig_1	cds	321717	321819	-	GLNHAC_03671		NupC/NupG family nucleoside CNT transporter
contig_1	cds	321833	321857	-	GLNHAC_03672	yuzB	UPF0349 protein RBAM_029300
contig_1	cds	321887	321990	+	GLNHAC_03673	yutJ	NADH dehydrogenase-like protein YutJ
contig_1	cds	321992	322025	-	GLNHAC_03674	yuzD	Putative disulfide oxidoreductase YuzD
contig_1	cds	322045	322068	+	GLNHAC_03675	yutI	Putative nitrogen fixation protein YutI
contig_1	cds	322072	322270	-	GLNHAC_03676		S9 family peptidase
contig_1	cds	322280	322373	-	GLNHAC_03677	thrB	homoserine kinase
contig_1	cds	322373	322480	-	GLNHAC_03678	thrC	threonine synthase
contig_1	cds	322479	322609	-	GLNHAC_03679	hom	Homoserine dehydrogenase
contig_1	cds	322626	322728	-	GLNHAC_03680	yutH	spore coat-associated protein CotNH
contig_1	cds	322744	322794	+	GLNHAC_03681	yutG	Uncharacterized protein YutG
contig_1	cds	322796	322873	-	GLNHAC_03682	yutF	5' nucleotidase NucF
contig_1	cds	322876	322920	-	GLNHAC_03683	yutE	Putative RNase YutE
contig_1	cds	322922	322950	-	GLNHAC_03684	yutD	Putative antitoxin YutD
contig_1	cds	322961	323025	+	GLNHAC_03685	yutC	Uncharacterized lipoprotein YutC
contig_1	cds	323026	323116	-	GLNHAC_03686	lipA	lipoyl synthase
contig_1	cds	323139	323237	+	GLNHAC_03687	lytH	L-Ala--D-Glu endopeptidase
contig_1	cds	323240	323317	-	GLNHAC_03688	yunB	sporulation protein YunB
contig_1	cds	323324	323355	-	GLNHAC_03689	yunC	Uncharacterized protein YunC

contig_1	cds	323361	323500	-	GLNHAC_03690	yunD	Uncharacterized metallophosphoesterase YunD
contig_1	cds	323502	323584	-	GLNHAC_03691	yunE	putative membrane transporter protein YunE
contig_1	cds	323586	323623	-	GLNHAC_03692		DUF72 domain-containing protein
contig_1	cds	323629	323663	-	GLNHAC_03693		DUF72 domain-containing protein
contig_1	cds	323674	323709	-	GLNHAC_03694	yunG	Uncharacterized protein YunG
contig_1	cds	323719	323750	-	GLNHAC_03695		(pseudo) amidohydrolase family protein
contig_1	cds	323764	323853	-	GLNHAC_03696		amidohydrolase family protein
contig_1	cds	323870	324030	+	GLNHAC_03697	pucR	purine catabolism transcriptional regulator PucR
contig_1	cds	324044	324179	+	GLNHAC_03698	pucJ	uric acid permease PucJ
contig_1	cds	324180	324309	+	GLNHAC_03699	pucK	uric acid permease PucK
contig_1	cds	324310	324459	+	GLNHAC_03700	pucL	factor-independent urate hydroxylase
contig_1	cds	324523	324535	+	GLNHAC_03701	yjcZ	YjcZ family sporulation protein
contig_1	cds	324570	324593	-	GLNHAC_03702		hypothetical protein
contig_1	cds	324615	324632	-	GLNHAC_03703		hypothetical protein
contig_1	cds	324708	324762	-	GLNHAC_03704	resA	Thiol-disulfide oxidoreductase ResA
contig_1	cds	324766	324837	+	GLNHAC_03705	ccdA	CytoChrome c-type biogenesis protein CcdA
contig_1	cds	324841	324879	-	GLNHAC_03706		hypothetical protein
contig_1	cds	324920	324933	+	GLNHAC_03707		hypothetical protein
contig_1	cds	325028	325080	-	GLNHAC_03708	pucE	xanthine dehydrogenase subunit E
contig_1	cds	325080	325303	-	GLNHAC_03709	pucD	xanthine dehydrogenase subunit D
contig_1	cds	325303	325387	-	GLNHAC_03710	pucC	xanthine dehydrogenase subunit C
contig_1	cds	325386	325448	-	GLNHAC_03711	pucB	xanthine dehydrogenase accessory protein PucB
contig_1	cds	325448	325547	-	GLNHAC_03712	xdhC	XdhC family protein
contig_1	cds	325570	325695	-	GLNHAC_03713	pucG	(S)-ureidoglycine--glyoxylate transaminase
contig_1	cds	325697	325770	-	GLNHAC_03714	allC	(pseudo) allantoate deiminase
contig_1	cds	325770	325820	-	GLNHAC_03715		hypothetical protein
contig_1	cds	325849	325883	+	GLNHAC_03716		(pseudo) Glycosyl transferase family, helical bundle domain p
contig_1	cds	325891	325996	+	GLNHAC_03717		hypothetical protein
contig_1	cds	325986	326091	-	GLNHAC_03718	yurJ	Uncharacterized ABC transporter ATP-binding protein YurJ
contig_1	cds	326109	326182	+	GLNHAC_03719	yurK	transcriptional regulator FrlR
contig_1	cds	326185	326270	-	GLNHAC_03720	frlD	Fructosamine kinase FrlD
contig_1	cds	326271	326351	-	GLNHAC_03721	yurM	putative ABC transporter permease protein YurM
contig_1	cds	326348	326361	-	GLNHAC_03722		hypothetical protein
contig_1	cds	326362	326450	-	GLNHAC_03723	yurN	putative ABC transporter permease protein YurN
contig_1	cds	326455	326532	-	GLNHAC_03724		extracellular solute-binding protein
contig_1	cds	326530	326582	-	GLNHAC_03725		ABC transporter substrate-binding protein
contig_1	cds	326590	326689	-	GLNHAC_03726	frlB	fructosamine deglycase FrlB
contig_1	cds	326710	326748	-	GLNHAC_03727	yurQ	Uncharacterized protein YurQ
contig_1	cds	326757	326870	-	GLNHAC_03728	yurR	Uncharacterized oxidoreductase YurR
contig_1	cds	326886	326901	+	GLNHAC_03729		Spore protein
contig_1	cds	326901	326928	+	GLNHAC_03730	yurS	Uncharacterized protein YurS
contig_1	cds	326944	326982	-	GLNHAC_03731		Glyoxalase family protein
contig_1	cds	327067	327207	-	GLNHAC_03732	sufB	Fe-S cluster assembly protein SufB
contig_1	cds	327209	327253	-	GLNHAC_03733	sufU	iron-sulfur cluster assembly scaffold protein SufU
contig_1	cds	327252	327374	-	GLNHAC_03734	sufS	cysteine desulfurase SufS
contig_1	cds	327374	327505	-	GLNHAC_03735	sufD	Fe-S cluster assembly protein SufD
contig_1	cds	327507	327537	-	GLNHAC_03736	sufC	Iron-sulfur cluster assembly ATPase protein SufC
contig_1	cds	327536	327586	-	GLNHAC_03737	sufC	Fe-S cluster assembly ATPase SufC
contig_1	cds	327605	327618	-	GLNHAC_03738	yuzK	Uncharacterized protein YuzK
contig_1	cds	327638	327674	-	GLNHAC_03739	yurZ	Uncharacterized protein YurZ
contig_1	cds	327684	327767	-	GLNHAC_03740	metQ	methionine ABC transporter substrate-binding lipoprotein Me
contig_1	cds	327768	327806	-	GLNHAC_03741		Putative ABC transporter permease
contig_1	cds	327807	327835	-	GLNHAC_03742		hypothetical protein
contig_1	cds	327834	327937	-	GLNHAC_03743	metN	methionine ABC transporter ATP-binding protein MetN
contig_1	ncRNA-region	327947	327958	-			SAM riboswitch aptamer (S box leader)
contig_1	cds	327969	328004	-	GLNHAC_03744	yusD	SCP2 domain-containing protein YusD
contig_1	cds	328014	328046	-	GLNHAC_03745	yusE	Thioredoxin-like protein YusE
contig_1	cds	328047	328091	-	GLNHAC_03746	yusF	Uncharacterized protein YusF
contig_1	cds	328091	328114	-	GLNHAC_03747	yusG	Uncharacterized protein YusG
contig_1	cds	328120	328158	-	GLNHAC_03748	gcvH	glycine cleavage system protein GcvH
contig_1	cds	328165	328200	-	GLNHAC_03749	yusI	Uncharacterized protein YusI
contig_1	cds	328211	328390	-	GLNHAC_03750	fadE	acyl-CoA dehydrogenase FadE
contig_1	cds	328391	328509	-	GLNHAC_03751	fadA	acetyl-CoA C-acetyltransferase
contig_1	cds	328510	328755	-	GLNHAC_03752	fadN	putative 3-hydroxyacyl-CoA dehydrogenase
contig_1	cds	328764	328779	+	GLNHAC_03753	yuzL	Uncharacterized protein YuzL
contig_1	cds	328781	328872	-	GLNHAC_03754	fadM	Proline dehydrogenase 1
contig_1	cds	328882	328906	+	GLNHAC_03755	yuzM	Uncharacterized protein YuzM
contig_1	cds	328907	328941	+	GLNHAC_03756	yusN	Uncharacterized protein YusN
contig_1	cds	328957	329004	+	GLNHAC_03757	mdtR	MarR family transcriptional regulator MdtR
contig_1	cds	329004	329065	+	GLNHAC_03758		MFS transporter
contig_1	cds	329065	329161	+	GLNHAC_03759	mdtP	(pseudo) Multidrug transporter protein MdtP
contig_1	cds	329169	329207	-	GLNHAC_03760	yusQ	putative tautomerase YusQ
contig_1	cds	329209	329283	-	GLNHAC_03761	yusS	Short-chain dehydrogenase/reductase-like protein YusS
contig_1	cds	329297	329385	+	GLNHAC_03762	yusT	Uncharacterized HTH-type transcriptional regulator YusT
contig_1	cds	329387	329416	-	GLNHAC_03763	yusU	Uncharacterized protein YusU
contig_1	cds	329419	329510	-	GLNHAC_03764	yusV	putative siderophore transport system ATP-binding protein Yu
contig_1	cds	329523	329566	-	GLNHAC_03765	yusW	YusW family protein
contig_1	cds	329581	329650	-	GLNHAC_03766		Peptidase M3A/M3B catalytic domain-containing protein
contig_1	cds	329650	329763	-	GLNHAC_03767		(pseudo) M3 family oligoendopeptidase
contig_1	cds	329775	329861	+	GLNHAC_03768	yusZ	Uncharacterized oxidoreductase YusZ
contig_1	cds	329868	329914	+	GLNHAC_03769	mrgA	metalloregulation DNA-binding stress protein MgrA
contig_1	cds	329919	330056	-	GLNHAC_03770	htrB	serine protease Do-like protein HtrB
contig_1	cds	330084	330152	+	GLNHAC_03771	cssR	secretion stress-responsive two-component system response
contig_1	cds	330151	330287	+	GLNHAC_03772	cssS	secretion stress-responsive two-component system sensor hi
contig_1	cds	330290	330306	-	GLNHAC_03773	spxO	anti-adapter protein SpxO
contig_1	cds	330323	330411	+	GLNHAC_03774	yuxN	Uncharacterized HTH-type transcriptional regulator YuxN
contig_1	cds	330415	330553	-	GLNHAC_03775	fumC	class II fumarate hydratase
contig_1	cds	330560	330579	-	GLNHAC_03776	yvzF	Uncharacterized protein YvzF
contig_1	cds	330590	330689	+	GLNHAC_03777	gerAA	(pseudo) Spore germination protein A1
contig_1	cds	330690	330735	+	GLNHAC_03778		Spore germination protein
contig_1	cds	330732	330842	+	GLNHAC_03779	gerAB	Spore germination protein A2
contig_1	cds	330841	330895	+	GLNHAC_03780		(pseudo) Spore germination protein AC
contig_1	cds	330895	330953	+	GLNHAC_03781		(pseudo) Spore germination protein AC
contig_1	cds	330954	331018	-	GLNHAC_03782	liaR	two-component system response regulator LiaR
contig_1	cds	331015	331124	-	GLNHAC_03783	liaS	two-component system sensor histidine kinase LiaS
contig_1	cds	331123	331196	-	GLNHAC_03784	liaF	LiaRS two-component regulatory system accessory protein L
contig_1	cds	331199	331286	-	GLNHAC_03785		DUF4097 domain-containing protein
contig_1	cds	331296	331364	-	GLNHAC_03786	liaH	Protein LiaH
contig_1	cds	331367	331405	-	GLNHAC_03787	liaI	cell envelope stress-induced membrane anchor protein LiaI
contig_1	cds	331442	331569	-	GLNHAC_03788	yvqJ	Uncharacterized MFS-type transporter YvqJ
contig_1	cds	331588	331644	-	GLNHAC_03789	yvqK	Corrinoid adenosyltransferase
contig_1	cds	331646	331779	-	GLNHAC_03790		Putative vitamin B12 transport system, ATPase component
contig_1	cds	331779	331883	-	GLNHAC_03791	yvrB	Uncharacterized ABC transporter permease protein YvrB
contig_1	cds	331905	331977	-	GLNHAC_03792		helical backbone metal receptor
contig_1	ncRNA-region	331982	332001	-			Cobalamin riboswitch aptamer
contig_1	cds	332017	332096	+	GLNHAC_03793	yvrD	Uncharacterized oxidoreductase YvrD
contig_1	cds	332100	332187	-	GLNHAC_03794	yvrE	Putative sugar lactone lactonase YvrE
contig_1	cds	332194	332307	-	GLNHAC_03795		HAMP domain-containing sensor histidine kinase
contig_1	cds	332310	332369	-	GLNHAC_03796		hypothetical protein
contig_1	cds	332368	332440	-	GLNHAC_03797	yvrH	two-component system response regulator YvrH
contig_1	cds	332455	332479	-	GLNHAC_03798	rsoA	sigma-O factor regulator RsoA
contig_1	cds	332478	332536	-	GLNHAC_03799	sigO	RNA polymerase sigma factor SigO
contig_1	cds	332552	332568	+	GLNHAC_03800	yvrJ	YvrJ family protein
contig_1	cds	332580	332696	+	GLNHAC_03801	oxdC	oxalate decarboxylase
contig_1	cds	332702	332743	+	GLNHAC_03802	yvrL	Membrane-bound negative regulator YvrL
contig_1	cds	332746	332871	-	GLNHAC_03803	yvrN	Uncharacterized ABC transporter permease YvrN
contig_1	cds	332868	332937	-	GLNHAC_03804	yvrO	Uncharacterized ABC transporter ATP-binding protein YvrO
contig_1	cds	332936	333055	-	GLNHAC_03805	yvrP	Putative efflux system protein YvrP
contig_1	cds	333072	333153	-	GLNHAC_03806	fhuC	Iron(3+)-hydroxamate import ATP-binding protein FhuC
contig_1	cds	333155	333256	-	GLNHAC_03807	fhuG	Iron(3+)-hydroxamate import system permease protein FhuG
contig_1	cds	333256	333378	-	GLNHAC_03808	fhuB	Iron(3+)-hydroxamate import system permease protein FhuB
contig_1	cds	333381	333475	+	GLNHAC_03809	fhuD	Iron(3+)-hydroxamate-binding protein FhuD
contig_1	cds	333511	333652	-	GLNHAC_03810	yvsH	Putative arginine/ornithine antiporter
contig_1	ncRNA-region	333662	333680	-			Lysine riboswitch aptamer
contig_1	cds	333692	333706	-	GLNHAC_03811	sspJ	small acid-soluble spore protein SspJ
contig_1	cds	333723	333771	+	GLNHAC_03812	yvsG	Uncharacterized protein YvsG
contig_1	cds	333781	333967	+	GLNHAC_03813	yvgJ	Lipoteichoic acid synthase-like YvgJ
contig_1	cds	333969	334062	-	GLNHAC_03814	yvgK	Uncharacterized protein YvgK
contig_1	cds	334073	334151	+	GLNHAC_03815	modA	molybdate ABC transporter substrate-binding protein
contig_1	cds	334149	334218	+	GLNHAC_03816	modB	molybdate ABC transporter permease subunit
contig_1	cds	334221	334304	-	GLNHAC_03817	yvgN	glyoxal/methylglyoxal reductase
contig_1	cds	334326	334374	+	GLNHAC_03818	yvgO	stress response protein YvgO
contig_1	cds	334379	334580	-	GLNHAC_03819	nhaK	Na+/H+ antiporter
contig_1	cds	334606	334719	-	GLNHAC_03820		Sulfite reductase [NADPH] hemoprotein beta-component (EC
contig_1	cds	334780	334961	-	GLNHAC_03821	cysJ	assimilatory sulfite reductase (NADPH) flavoprotein subunit
contig_1	cds	334978	335006	-	GLNHAC_03822		ATP-binding domain-containing protein
contig_1	cds	335011	335211	-	GLNHAC_03823	helD	RNA polymerase recycling motor HelD
contig_1	cds	335247	335291	-	GLNHAC_03824		Glycine transporter domain-containing protein
contig_1	cds	335309	335351	-	GLNHAC_03825	bdbC	Disulfide bond formation protein C

**contig_1**	cds	335351	335418	-	GLNHAC_03826	bdbD	Disulfide bond formation protein D
**contig_1**	cds	335430	335640	-	GLNHAC_03827	cadA	Cadmium, zinc and cobalt-transporting ATPase
**contig_1**	cds	335657	335715	-	GLNHAC_03828		P-type Cu(+) transporter
**contig_1**	cds	335711	335873	-	GLNHAC_03829	copZ	(pseudo) copper chaperone CopZ
**contig_1**	cds	335876	335898	-	GLNHAC_03830		hypothetical protein
**contig_1**	cds	335906	335927	-	GLNHAC_03831	copZ	copper chaperone CopZ
**contig_1**	cds	335934	335965	-	GLNHAC_03832	csoR	copper-sensing transcriptional repressor CsoR
**contig_1**	cds	335980	336085	+	GLNHAC_03833	iolW	scyllo-inositol 2-dehydrogenase (NADP(+)) IolW
**contig_1**	cds	336089	336152	-	GLNHAC_03834	azoRB	FMN-dependent NADH-azoreductase AzoRB
**contig_1**	cds	336169	336358	-	GLNHAC_03835		Putative integral inner membrane protein
**contig_1**	cds	336374	336434	-	GLNHAC_03836	gntR	Transcriptional regulator, GntR family
**contig_1**	cds	336435	336495	-	GLNHAC_03837		(pseudo) Transporter, Drug/Metabolite Exporter family
**contig_1**	cds	336498	336530	-	GLNHAC_03838		EamA-like transporter family protein
**contig_1**	cds	336540	336580	-	GLNHAC_03839	yvaD	Uncharacterized protein YvaD
**contig_1**	cds	336579	336615	-	GLNHAC_03840	yvaE	Uncharacterized membrane protein YvaE
**contig_1**	cds	336615	336672	-	GLNHAC_03841	yvaF	Uncharacterized HTH-type transcriptional regulator YvaF
**contig_1**	cds	336683	336763	-	GLNHAC_03842	yvaG	Uncharacterized oxidoreductase YvaG
**contig_1**	tmRNA	336781	336817	-	GLNHAC_03843	ssrA	transfer-messenger RNA, SsrA
**contig_1**	cds	336843	336882	-	GLNHAC_03844		SsrA-binding protein
**contig_1**	cds	336896	337130	-	GLNHAC_03845	rnr	ribonuclease R
**contig_1**	cds	337132	337206	-	GLNHAC_03846	est	Carboxylesterase
**contig_1**	cds	337219	337242	-	GLNHAC_03847	secG	preprotein translocase subunit SecG
**contig_1**	cds	337257	337334	+	GLNHAC_03848	yvaM	Protein YvaM
**contig_1**	cds	337338	337363	-	GLNHAC_03849		Transcriptional regulator
**contig_1**	cds	337376	337418	+	GLNHAC_03850	rghR	transcriptional repressor RghR
**contig_1**	cds	337420	337462	+	GLNHAC_03851	yvaO	YvaO
**contig_1**	cds	337472	337504	+	GLNHAC_03852	yvaP	catDE operon transcriptional regulator CatR
**contig_1**	cds	337518	337629	+	GLNHAC_03853	yvaQ	(pseudo) Putative sensory transducer protein YvaQ
**contig_1**	cds	337629	337688	+	GLNHAC_03854		(pseudo) methyl-accepting chemotaxis protein
**contig_1**	cds	337692	337829	-	GLNHAC_03855		histidine kinase
**contig_1**	cds	337828	337894	-	GLNHAC_03856	spaR	Transcriptional regulatory protein SpaR
**contig_1**	cds	337896	337973	-	GLNHAC_03857	etnG	EtnG
**contig_1**	cds	337973	338048	-	GLNHAC_03858	etnE	EtnE
**contig_1**	cds	338048	338120	-	GLNHAC_03859	rbbA	ABC transporter domain-containing protein
**contig_1**	cds	338121	338170	-	GLNHAC_03860		Lantibiotic immunity protein Spa1 C-terminal domain-containi
**contig_1**	cds	338219	338286	-	GLNHAC_03861	opuBD	choline ABC transporter permease OpuBD
**contig_1**	cds	338288	338380	-	GLNHAC_03862	opuBC	choline ABC transporter substrate-binding lipoprotein OpuBC
**contig_1**	cds	338381	338446	-	GLNHAC_03863	opuBB	choline ABC transporter permease OpuBB
**contig_1**	cds	338448	338562	-	GLNHAC_03864	opuBA	choline ABC transporter ATP-binding protein OpuBA
**contig_1**	cds	338591	338644	+	GLNHAC_03865	yvaV	Putative HTH-type transcriptional regulator YvaV
**contig_1**	cds	338648	338714	-	GLNHAC_03866	opuCD	glycine betaine/carnitine/choline/choline sulfate ABC transpo
**contig_1**	cds	338716	338808	-	GLNHAC_03867	opuCC	osmoprotectant ABC transporter substrate-binding lipoprotei
**contig_1**	cds	338809	338875	-	GLNHAC_03868	opuCB	glycine betaine/carnitine/choline/choline sulfate ABC transpo
**contig_1**	cds	338877	338991	-	GLNHAC_03869	opuCA	osmoprotectant ABC transporter ATP-binding protein OpuCA
**contig_1**	cds	339017	339073	+	GLNHAC_03870	opcR	opuC operon transcriptional regulator OpcR
**contig_1**	cds	339084	339117	+	GLNHAC_03871		YvbH-like oligomerisation domain-containing protein
**contig_1**	cds	339126	339195	+	GLNHAC_03872	yvbI	Uncharacterized protein YvbI
**contig_1**	cds	339199	339254	-	GLNHAC_03873		hypothetical protein
**contig_1**	cds	339254	339380	-	GLNHAC_03874		(pseudo) Zinc ribbon domain-containing protein
**contig_1**	cds	339394	339440	+	GLNHAC_03875		N-acetyltransferase domain-containing protein
**contig_1**	cds	339444	339573	-	GLNHAC_03876	eno	phosphopyruvate hydratase
**contig_1**	cds	339576	339730	-	GLNHAC_03877	gpmI	2,3-bisphosphoglycerate-independent phosphoglycerate mut
**contig_1**	cds	339767	339805	-	GLNHAC_03878	tpiA	(pseudo) triose-phosphate isomerase
**contig_1**	cds	339808	339817	-	GLNHAC_03879		phosphoglycerate kinase
**contig_1**	cds	339817	339926	-	GLNHAC_03880	pgk	Phosphoglycerate kinase
**contig_1**	cds	339963	340059	-	GLNHAC_03881	gap	type I glyceraldehyde-3-phosphate dehydrogenase
**contig_1**	cds	340063	340165	-	GLNHAC_03882	cggR	gapA transcriptional regulator CggR
**contig_1**	cds	340195	340335	-	GLNHAC_03883	araE	arabinose-proton symporter AraE
**contig_1**	cds	340355	340464	+	GLNHAC_03884	araR	arabinose utilization transcriptional regulator AraR
**contig_1**	cds	340469	340534	-	GLNHAC_03885		Luciferase-like domain-containing protein
**contig_1**	cds	340590	340607	-	GLNHAC_03886		hypothetical protein
**contig_1**	cds	340639	340655	-	GLNHAC_03887		hypothetical protein
**contig_1**	cds	340655	340664	-	GLNHAC_03888		hypothetical protein
**contig_1**	cds	340732	340773	+	GLNHAC_03889		(pseudo) DMT family transporter
**contig_1**	cds	340777	340912	-	GLNHAC_03890	yvbW	Uncharacterized amino acid permease YvbW
**contig_1**	ncRNA-region	340919	340942	-			T-box leader
**contig_1**	cds	340967	341008	-	GLNHAC_03891		(pseudo) Glycosyl hydrolase, family 18
**contig_1**	cds	341032	341075	-	GLNHAC_03892		IS3 family transposase
**contig_1**	ncRNA	341042	341052	+	GLNHAC_03893	rli23	Listeria snRNA rli23
**contig_1**	cds	341123	341147	-	GLNHAC_03894		Transposase
**contig_1**	cds	341159	341182	-	GLNHAC_03895		(pseudo) hypothetical protein
**contig_1**	cds	341193	341265	-	GLNHAC_03896	lutC	lactate utilization protein LutC
**contig_1**	cds	341265	341409	-	GLNHAC_03897	lutB	lactate utilization iron-sulfur protein LutB
**contig_1**	cds	341412	341483	-	GLNHAC_03898	lutA	lactate utilization iron-sulfur protein LutA
**contig_1**	cds	341501	341562	-	GLNHAC_03899		DUF2812 domain-containing protein
**contig_1**	cds	341562	341595	-	GLNHAC_03900	padR	Transcriptional regulator, PadR family
**contig_1**	cds	341607	341628	-	GLNHAC_03901	luxR	Bacterial regulatory s, luxR family protein
**contig_1**	cds	341628	341667	-	GLNHAC_03902		Response regulator transcription factor
**contig_1**	cds	341667	341781	-	GLNHAC_03903		histidine kinase
**contig_1**	cds	341781	341855	-	GLNHAC_03904	yvfS	Putative transport permease YvfS
**contig_1**	cds	341855	341945	-	GLNHAC_03905		ABC transporter ATP-binding protein
**contig_1**	cds	341974	342055	+	GLNHAC_03906		Alpha/beta hydrolase
**contig_1**	cds	342058	342179	+	GLNHAC_03907	rsbP	Phosphoserine phosphatase RsbP
**contig_1**	cds	342183	342312	-	GLNHAC_03908		arabinogalactan endo-1,4-beta-galactosidase
**contig_1**	cds	342320	342527	-	GLNHAC_03909	ganA	beta-galactosidase GanA
**contig_1**	cds	342529	342614	-	GLNHAC_03910	ganQ	arabinogalactan oligomer ABC transporter permease GanQ
**contig_1**	cds	342614	342740	-	GLNHAC_03911	ganP	arabinogalactan oligomer ABC transporter permease GanP
**contig_1**	cds	342744	342870	-	GLNHAC_03912	ganS	arabinogalactan oligomer ABC transporter substrate-binding
**contig_1**	cds	342884	342984	-	GLNHAC_03913	ganR	galactan degradation operon transcriptional regulator GanR
**contig_1**	cds	343001	343046	-	GLNHAC_03914		FCD domain-containing protein
**contig_1**	cds	343043	343074	-	GLNHAC_03915		hypothetical protein
**contig_1**	cds	343097	343266	+	GLNHAC_03916	lutP	L-lactate permease LutP
**contig_1**	cds	343269	343400	-	GLNHAC_03917	rpoN	RNA polymerase factor sigma-54
**contig_1**	cds	343408	343430	+	GLNHAC_03918	yvfG	Uncharacterized protein YvfG
**contig_1**	cds	343431	343527	-	GLNHAC_03919	epsO	Putative pyruvyl transferase EpsO
**contig_1**	cds	343525	343700	-	GLNHAC_03920	pglD	PglD N-terminal domain-containing protein
**contig_1**	cds	343707	343718	-	GLNHAC_03921		hypothetical protein
**contig_1**	cds	343718	343768	-	GLNHAC_03922	epsL	Uncharacterized sugar transferase EpsL
**contig_1**	cds	343768	343919	-	GLNHAC_03923	epsK	Uncharacterized membrane protein EpsK
**contig_1**	cds	343919	343984	-	GLNHAC_03924		(pseudo) Glycosyltransferase
**contig_1**	cds	343982	344022	-	GLNHAC_03925		glycosyltransferase family 2 protein
**contig_1**	cds	344022	344130	-	GLNHAC_03926		Pyruvyl transferase
**contig_1**	cds	344142	344156	-	GLNHAC_03927		hypothetical protein
**contig_1**	cds	344153	344233	-	GLNHAC_03928	epsH	(pseudo) Putative glycosyltransferase EpsH
**contig_1**	cds	344236	344324	-	GLNHAC_03929	epsG	(pseudo) Transmembrane protein EpsG
**contig_1**	cds	344367	344396	-	GLNHAC_03930		hypothetical protein
**contig_1**	cds	344396	344454	-	GLNHAC_03931	epsF	(pseudo) Putative glycosyltransferase EpsF
**contig_1**	cds	344460	344488	-	GLNHAC_03932		hypothetical protein
**contig_1**	cds	344504	344543	-	GLNHAC_03933		hypothetical protein
**contig_1**	cds	344543	344657	-	GLNHAC_03934	epsD	Putative glycosyltransferase EpsD
**contig_1**	cds	344701	344838	-	GLNHAC_03935	epsC	putative polysaccharide biosynthesis protein EpsC
**contig_1**	ncRNA-region	344851	344862	-			epsC RNA
**contig_1**	cds	344864	344932	-	GLNHAC_03936	epsB	protein tyrosine kinase EpsB
**contig_1**	cds	344933	345003	-	GLNHAC_03937	yveK	Uncharacterized protein YveK
**contig_1**	cds	345028	345073	+	GLNHAC_03938	slrR	transcriptional regulator SlrR
**contig_1**	cds	345081	345228	+	GLNHAC_03939	pnbA	Para-nitrobenzyl esterase
**contig_1**	cds	345228	345247	+	GLNHAC_03940		Lipoprotein
**contig_1**	cds	345250	345298	-	GLNHAC_03941	padC	phenolic acid decarboxylase
**contig_1**	cds	345301	345346	-	GLNHAC_03942	yveG	UPF0311 protein YveG
**contig_1**	cds	345359	345427	-	GLNHAC_03943	racX	broad specificity amino-acid racemase RacX
**contig_1**	cds	345429	345564	-	GLNHAC_03944		Beta-lactamase
**contig_1**	cds	345579	345635	-	GLNHAC_03945		C1q domain-containing protein
**contig_1**	cds	345695	345837	+	GLNHAC_03946	sacB	levansucrase
**contig_1**	cds	345844	345999	+	GLNHAC_03947	levB	Levanbiose-producing levanase
**contig_1**	cds	346010	346166	+	GLNHAC_03948	aspP	aspartate/proton symporter AspP
**contig_1**	cds	346176	346234	+	GLNHAC_03949	yvdT	Uncharacterized HTH-type transcriptional regulator YvdT
**contig_1**	cds	346242	346276	+	GLNHAC_03950	yvdS	Uncharacterized membrane protein YvdS
**contig_1**	cds	346276	346307	+	GLNHAC_03951	yvdR	Uncharacterized membrane protein YvdR
**contig_1**	cds	346312	346363	-	GLNHAC_03952	yvdQ	Uncharacterized protein YvdQ
**contig_1**	tRNA	346378	346386	-	GLNHAC_03953	trnR	tRNA-Arg(ccg)
**contig_1**	cds	346413	346472	+	GLNHAC_03954	clpP	ATP-dependent Clp endopeptidase proteolytic subunit ClpP
**contig_1**	cds	346479	346547	-	GLNHAC_03955	pgmB	beta-phosphoglucomutase
**contig_1**	cds	346547	346715	-	GLNHAC_03956	malL	Oligo-1,6-glucosidase 1
**contig_1**	cds	346727	346942	-	GLNHAC_03957		Glycosyl hydrolase, family 65
**contig_1**	cds	346939	347027	-	GLNHAC_03958	yvdJ	YvdJ
**contig_1**	cds	347027	347111	-	GLNHAC_03959	mdxG	maltodextrin ABC transporter permease MdxG
**contig_1**	cds	347111	347242	-	GLNHAC_03960	mdxF	maltodextrin ABC transporter permease MdxF
**contig_1**	cds	347246	347371	-	GLNHAC_03961	mdxE	maltodextrin ABC transporter substrate-binding protein MdxE

**contig_1**	cds	347381	347557	-	GLNHAC_03962		glycoside hydrolase family 13 protein
**contig_1**	cds	347569	347664	-	GLNHAC_03963	yvdE	Uncharacterized HTH-type transcriptional regulator YvdE
**contig_1**	cds	347686	347743	-	GLNHAC_03964	yvdD	LOG family protein YvdD
**contig_1**	cds	347755	347787	+	GLNHAC_03965	yvdC	Uncharacterized protein YvdC
**contig_1**	cds	347790	347949	-	GLNHAC_03966	yvdB	Putative sulfate transporter YvdB
**contig_1**	cds	347951	348009	-	GLNHAC_03967	yvdA	Putative carbonic anhydrase YvdA
**contig_1**	cds	348050	348091	+	GLNHAC_03968		hypothetical protein
**contig_1**	cds	348096	348147	+	GLNHAC_03969		2-ketoaldonate reductase/glyoxylate reductase B
**contig_1**	cds	348151	348345	-	GLNHAC_03970	yvcS	Uncharacterized ABC transporter permease YvcS
**contig_1**	cds	348343	348420	-	GLNHAC_03971	yvcR	lantibiotic ABC transporter ATP-binding protein PsdA
**contig_1**	cds	348429	348536	-	GLNHAC_03972	yvcQ	Sensor histidine kinase YvcQ
**contig_1**	cds	348535	348606	-	GLNHAC_03973	yvcP	two-component system response regulator PsdR
**contig_1**	cds	348621	348642	+	GLNHAC_03974	yvzJ	Uncharacterized lipoprotein YvzJ
**contig_1**	cds	348646	348721	-	GLNHAC_03975	yvcN	YvcN
**contig_1**	cds	348722	348748	-	GLNHAC_03976	crh	HPr family phosphocarrier protein
**contig_1**	cds	348750	348845	-	GLNHAC_03977	whiA	DNA-binding protein WhiA
**contig_1**	cds	348848	348937	-	GLNHAC_03978	mgfK	gluconeogenesis morphogenetic factor
**contig_1**	cds	348936	349032	-	GLNHAC_03979	rapZ	RNase adapter RapZ
**contig_1**	cds	349052	349081	-	GLNHAC_03980	yvcI	(pseudo) Uncharacterized Nudix hydrolase YvcI
**contig_1**	cds	349114	349209	-	GLNHAC_03981	trxB	thioredoxin-disulfide reductase
**contig_1**	cds	349231	349372	-	GLNHAC_03982	cwlO	Peptidoglycan DL-endopeptidase CwlO
**contig_1**	cds	349410	349556	-	GLNHAC_03983	yvcD	TPR repeat-containing protein YvcD
**contig_1**	cds	349568	349745	-	GLNHAC_03984	bmrA	multidrug resistance ABC transporter ATP-binding protein/per
**contig_1**	cds	349762	349798	-	GLNHAC_03985	yvzA	Uncharacterized protein YvzA
**contig_1**	cds	349799	349896	-	GLNHAC_03986		hypothetical protein
**contig_1**	cds	350033	350062	-	GLNHAC_03987	yvcA	(pseudo) lipoprotein YvcA
**contig_1**	cds	350087	350150	-	GLNHAC_03988	hisIE	bifunctional phosphoribosyl-AMP cyclohydrolase/phosphorib
**contig_1**	cds	350149	350225	-	GLNHAC_03989	hisF	Imidazole glycerol phosphate synthase subunit HisF
**contig_1**	cds	350225	350299	-	GLNHAC_03990	hisA	1-(5-phosphoribosyl)-5-[(5-phosphoribosylamino)methylidene
**contig_1**	cds	350298	350362	-	GLNHAC_03991	hisH	imidazole glycerol phosphate synthase subunit HisH
**contig_1**	cds	350362	350421	-	GLNHAC_03992	hisB	imidazoleglycerol-phosphate dehydratase HisB
**contig_1**	cds	350420	350503	-	GLNHAC_03993		Histidinol dehydrogenase
**contig_1**	cds	350503	350516	-	GLNHAC_03994		Histidinol dehydrogenase
**contig_1**	cds	350532	350548	-	GLNHAC_03995		hypothetical protein
**contig_1**	cds	350548	350612	-	GLNHAC_03996	hisG	ATP phosphoribosyltransferase
**contig_1**	cds	350611	350658	-	GLNHAC_03997		hypothetical protein
**contig_1**	cds	350663	350729	-	GLNHAC_03998	hisZ	(pseudo) ATP phosphoribosyltransferase regulatory subunit
**contig_1**	cds	350754	350829	+	GLNHAC_03999	yvpB	Uncharacterized protein YvpB
**contig_1**	cds	350854	350921	+	GLNHAC_04000	pelC	Pectate lyase C
**contig_1**	cds	350923	350975	-	GLNHAC_04001	yvoF	Putative acetyltransferase YvoF
**contig_1**	cds	350975	351040	-	GLNHAC_04002	ppaX	pyrophosphatase PpaX
**contig_1**	cds	351040	351134	-	GLNHAC_04003	yvoD	Uncharacterized membrane protein YvoD
**contig_1**	cds	351136	351217	-	GLNHAC_04004	lgt	prolipoprotein diacylglyceryl transferase
**contig_1**	cds	351219	351312	-	GLNHAC_04005	hprK	HPr(Ser) kinase/phosphatase
**contig_1**	cds	351330	351449	+	GLNHAC_04006	nagA	N-acetylglucosamine-6-phosphate deacetylase
**contig_1**	cds	351449	351521	+	GLNHAC_04007	nagB	glucosamine-6-phosphate deaminase
**contig_1**	cds	351523	351596	+	GLNHAC_04008	nagR	transcriptional regulator NagR
**contig_1**	cds	351601	351624	-	GLNHAC_04009		hypothetical protein
**contig_1**	cds	351625	351985	-	GLNHAC_04010	yvnB	Uncharacterized protein YvnB
**contig_1**	cds	352001	352049	+	GLNHAC_04011		Putative transcriptional regulator
**contig_1**	cds	352053	352174	-	GLNHAC_04012	cypX	pulcherriminic acid synthase
**contig_1**	cds	352176	352251	-	GLNHAC_04013	yvmC	Cyclo(L-leucyl-L-leucyl) synthase
**contig_1**	cds	352294	352345	+	GLNHAC_04014	pchR	pulcherriminic acid biosynthesis transcriptional regulator Pch
**contig_1**	cds	352348	352469	+	GLNHAC_04015		MFS transporter
**contig_1**	cds	352472	352508	-	GLNHAC_04016	yvlD	Uncharacterized membrane protein YvlD
**contig_1**	cds	352507	352527	-	GLNHAC_04017	yvlC	Uncharacterized membrane protein YvlC
**contig_1**	cds	352528	352638	-	GLNHAC_04018	yvlB	Uncharacterized protein YvlB
**contig_1**	cds	352640	352673	-	GLNHAC_04019	yvlA	Uncharacterized protein YvlA
**contig_1**	cds	352695	352718	+	GLNHAC_04020	yvkN	Uncharacterized protein YvkN
**contig_1**	cds	352728	353015	-	GLNHAC_04021	uvrA	excinuclease ABC subunit UvrA
**contig_1**	cds	353016	353215	-	GLNHAC_04022	uvrB	excinuclease ABC subunit UvrB
**contig_1**	cds	353233	353256	-	GLNHAC_04023	csbA	Protein CsbA
**contig_1**	cds	353284	353311	+	GLNHAC_04024		(pseudo) hypothetical protein
**contig_1**	cds	353348	353576	+	GLNHAC_04025	yvkC	Uncharacterized phosphotransferase YvkC
**contig_1**	cds	353576	353597	+	GLNHAC_04026		PEP-utilizing enzyme
**contig_1**	cds	353605	353661	+	GLNHAC_04027		TetR/AcrR family transcriptional regulator
**contig_1**	cds	353666	353690	+	GLNHAC_04028		hypothetical protein
**contig_1**	cds	353688	353798	+	GLNHAC_04029	yvkA	Uncharacterized MFS-type transporter YvkA
**contig_1**	cds	353803	353922	-	GLNHAC_04030	minJ	cell division topological determinant MinJ
**contig_1**	cds	353930	353964	-	GLNHAC_04031	swrA	swarming motility protein SwrAA
**contig_1**	cds	354004	354148	-	GLNHAC_04032	ctpB	carboxy-terminal processing protease CtpB
**contig_1**	cds	354162	354252	-	GLNHAC_04033	ftsX	permease-like cell division protein FtsX
**contig_1**	cds	354250	354319	-	GLNHAC_04034	ftsE	cell division ATP-binding protein FtsE
**contig_1**	cds	354342	354376	-	GLNHAC_04035	cccB	cytochrome c-551
**contig_1**	cds	354381	354465	-	GLNHAC_04036	yvjA	UPF0750 membrane protein YvjA
**contig_1**	cds	354484	354564	-	GLNHAC_04037	tnp	IS3 family ISBsu1 transposase ORF B
**contig_1**	ncRNA	354494	354504	+	GLNHAC_04038	rli23	Listeria snRNA rli23
**contig_1**	cds	354566	354590	-	GLNHAC_04039	tnp	IS3 family transposase ORF A
**contig_1**	cds	354590	354599	-	GLNHAC_04040		(pseudo) helix-turn-helix domain-containing protein
**contig_1**	cds	354612	354635	-	GLNHAC_04041		hypothetical protein
**contig_1**	cds	354640	354710	-	GLNHAC_04042		hypothetical protein
**contig_1**	cds	354729	354981	-	GLNHAC_04043	secA	preprotein translocase subunit SecA
**contig_1**	cds	354998	355055	-	GLNHAC_04044	hpf	ribosome hibernation promotion factor
**contig_1**	cds	355074	355112	-	GLNHAC_04045	yvzG	Uncharacterized protein YvzG
**contig_1**	cds	355113	355147	-	GLNHAC_04046	fliT	Flagellar protein FliT
**contig_1**	cds	355147	355187	-	GLNHAC_04047	fliS	flagellar export chaperone FliS
**contig_1**	cds	355189	355339	-	GLNHAC_04048	fliD	flagellar hook-associated protein 2
**contig_1**	cds	355340	355373	-	GLNHAC_04049	flaG	flagellar protein FlaG
**contig_1**	cds	355441	355483	-	GLNHAC_04050		Flagellin
**contig_1**	cds	355497	355520	-	GLNHAC_04051	csrA	carbon storage regulator CsrA
**contig_1**	cds	355519	355562	-	GLNHAC_04052	fliW	flagellar assembly protein FliW
**contig_1**	cds	355564	355622	-	GLNHAC_04053	yviE	Uncharacterized protein YviE
**contig_1**	cds	355627	355716	-	GLNHAC_04054	flgL	flagellar hook-associated protein FlgL
**contig_1**	cds	355717	355870	-	GLNHAC_04055	flgK	flagellar hook-associated protein FlgK
**contig_1**	cds	355871	355920	-	GLNHAC_04056	flgN	flagellar protein FlgN
**contig_1**	cds	355921	355948	-	GLNHAC_04057	flgM	flagellar biosynthesis anti-sigma factor FlgM
**contig_1**	cds	356005	356077	-	GLNHAC_04058	comFC	ComF operon protein 3
**contig_1**	cds	356073	356103	-	GLNHAC_04059	comFB	ComF operon protein 2
**contig_1**	cds	356110	356248	-	GLNHAC_04060	comF	ComF operon protein 1
**contig_1**	cds	356259	356343	-	GLNHAC_04061	degV	Protein DegV
**contig_1**	cds	356353	356422	-	GLNHAC_04062	degU	two-component system response regulator DegU
**contig_1**	cds	356430	356546	-	GLNHAC_04063	degS	two-component sensor histidine kinase DegS
**contig_1**	cds	356568	356623	+	GLNHAC_04064	yvyE	IMPACT family member YvyE
**contig_1**	cds	356617	356628	+	GLNHAC_04065		hypothetical protein
**contig_1**	cds	356633	356665	+	GLNHAC_04066		hypothetical protein
**contig_1**	cds	356665	356693	+	GLNHAC_04067		hypothetical protein
**contig_1**	cds	356775	356883	-	GLNHAC_04068	tagO	putative undecaprenyl-phosphate N-acetylglucosaminyl 1-ph
**contig_1**	cds	356898	356952	-	GLNHAC_04069	tuaH	(pseudo) teichuronic acid biosynthesis protein TuaH
**contig_1**	cds	356988	357018	-	GLNHAC_04070		hypothetical protein
**contig_1**	cds	357020	357096	-	GLNHAC_04071	tuaG	teichuronic acid biosynthesis protein TuaG
**contig_1**	cds	357098	357166	-	GLNHAC_04072	tuaF	teichuronic acid biosynthesis protein TuaF
**contig_1**	cds	357169	357316	-	GLNHAC_04073	tuaE	teichuronic acid biosynthesis protein TuaE
**contig_1**	cds	357324	357464	-	GLNHAC_04074	tuaD	UDP-glucose 6-dehydrogenase TuaD
**contig_1**	cds	357469	357586	-	GLNHAC_04075	tuaC	teichuronic acid biosynthesis protein TuaC
**contig_1**	cds	357585	357731	-	GLNHAC_04076	tuaB	teichuronic acid biosynthesis protein TuaB
**contig_1**	cds	357736	357802	-	GLNHAC_04077		Putative undecaprenyl-phosphate N-acetylgalactosaminyl 1-p
**contig_1**	cds	357834	357925	-	GLNHAC_04078		(pseudo) glycosyltransferase
**contig_1**	cds	357929	358016	-	GLNHAC_04079		glycosyltransferase family A protein
**contig_1**	cds	358035	358102	-	GLNHAC_04080	yqiI	Uncharacterized protein YqiI
**contig_1**	cds	358114	358183	-	GLNHAC_04081		(pseudo) N-acetylmuramoyl-L-alanine amidase
**contig_1**	cds	358192	358408	-	GLNHAC_04082	lytC	Modifier protein of major autolysin LytC
**contig_1**	cds	358410	358443	-	GLNHAC_04083	lytA	Membrane-bound protein LytA
**contig_1**	cds	358463	358473	+	GLNHAC_04084		hypothetical protein
**contig_1**	cds	358471	358535	+	GLNHAC_04085	lytR	Membrane-bound protein LytR
**contig_1**	cds	358539	358653	-	GLNHAC_04086	wecB	non-hydrolyzing UDP-N-acetylglucosamine 2-epimerase
**contig_1**	cds	358655	358845	-	GLNHAC_04087		Putative glycosyltransferase
**contig_1**	cds	358862	358892	-	GLNHAC_04088		hypothetical protein
**contig_1**	cds	358897	358946	-	GLNHAC_04089		hypothetical protein
**contig_1**	cds	358977	359005	-	GLNHAC_04090		hypothetical protein
**contig_1**	cds	359195	359283	+	GLNHAC_04091	galU	UTP--glucose-1-phosphate uridylyltransferase GalU
**contig_1**	cds	359287	359445	-	GLNHAC_04092	tagH	teichoic acids export ABC transporter ATP-binding subunit Ta
**contig_1**	cds	359447	359530	-	GLNHAC_04093	tagG	teichoic acids export ABC transporter permease subunit Tag(
**contig_1**	cds	359822	359868	-	GLNHAC_04094		CDP-glycerol glycerophosphotransferase family protein
**contig_1**	cds	359866	359903	-	GLNHAC_04095		hypothetical protein
**contig_1**	cds	359907	359931	-	GLNHAC_04096		hypothetical protein
**contig_1**	cds	359939	359979	-	GLNHAC_04097	tagD	glycerol-3-phosphate cytidylyltransferase
**contig_1**	cds	360028	360106	+	GLNHAC_04098	tarA	N-acetylglucosaminyldiphosphoundecaprenol N-acetyl-beta-
**contig_1**	cds	360115	360230	+	GLNHAC_04099	tarB	Teichoic acid glycerol-phosphate primase

**contig_1**	cds	360239	360310	+	GLNHAC_04100	tarI	Ribitol-5-phosphate cytidylyltransferase
**contig_1**	cds	360310	360413	+	GLNHAC_04101	tarJ	Ribulose-5-phosphate reductase
**contig_1**	cds	360413	360530	+	GLNHAC_04102	tarK	Teichoic acid ribitol-phosphate primase
**contig_1**	cds	360532	360578	+	GLNHAC_04103		hypothetical protein
**contig_1**	cds	360602	360718	+	GLNHAC_04104		CDP-glycerol:glycerophosphate glycerophosphotransferase
**contig_1**	cds	360721	360810	-	GLNHAC_04105		SH3b domain-containing protein
**contig_1**	cds	360823	360985	-	GLNHAC_04106		hypothetical protein
**contig_1**	cds	360998	361093	-	GLNHAC_04107	manA	mannose-6-phosphate isomerase, class I
**contig_1**	cds	361120	361265	+	GLNHAC_04108		spore germination protein
**contig_1**	cds	361266	361376	+	GLNHAC_04109		spore germination protein
**contig_1**	cds	361376	361488	+	GLNHAC_04110		Lipoprotein component of the germination receptor B
**contig_1**	cds	361492	361629	-	GLNHAC_04111	ywtG	Putative metabolite transport protein YwtG
**contig_1**	cds	361663	361697	+	GLNHAC_04112		hypothetical protein
**contig_1**	cds	361696	361743	+	GLNHAC_04113	tagT	(pseudo) Polyisoprenyl-teichoic acid--peptidoglycan teichoic
**contig_1**	cds	361741	361759	+	GLNHAC_04114		hypothetical protein
**contig_1**	cds	361775	361861	+	GLNHAC_04115	ywtE	5-amino-6-(5-phospho-D-ribitylamino)uracil phosphatase Ywt
**contig_1**	cds	361865	361989	-	GLNHAC_04116	pgdS	gamma-DL-glutamyl hydrolase
**contig_1**	cds	362003	362020	-	GLNHAC_04117	ywtC	extrachromosomal elements maintenance protein EdmS
**contig_1**	cds	362021	362057	-	GLNHAC_04118		hypothetical protein
**contig_1**	cds	362052	362135	-	GLNHAC_04119	capA	(pseudo) PGA biosynthesis protein CapA
**contig_1**	cds	362137	362182	-	GLNHAC_04120	pgsC	capsular polyglutamate amide ligase/translocase PgsC
**contig_1**	cds	362183	362302	-	GLNHAC_04121	capB	PGA synthase CapB
**contig_1**	cds	362380	362471	+	GLNHAC_04122		Ribose operon repressor
**contig_1**	cds	362498	362565	+	GLNHAC_04123	pfkB	PfkB family carbohydrate kinase
**contig_1**	cds	362565	362604	+	GLNHAC_04124	rbsD	D-ribose pyranase
**contig_1**	cds	362606	362754	+	GLNHAC_04125	rbsA	ribose ABC transporter ATP-binding protein RbsA
**contig_1**	cds	362754	362851	+	GLNHAC_04126	rbsC	ribose ABC transporter permease RbsC
**contig_1**	cds	362860	362944	+	GLNHAC_04127	rbsB	ribose ABC transporter substrate-binding protein RbsB
**contig_1**	cds	362955	362979	+	GLNHAC_04128		hypothetical protein
**contig_1**	cds	362977	363009	+	GLNHAC_04129	ywsB	(pseudo) Cell wall-binding protein YwsB
**contig_1**	cds	363024	363054	+	GLNHAC_04130	ywsA	Uncharacterized protein YwsA
**contig_1**	cds	363058	363111	-	GLNHAC_04131	ywrO	General stress protein 14
**contig_1**	cds	363121	363197	-	GLNHAC_04132	budA	acetolactate decarboxylase
**contig_1**	cds	363204	363351	-	GLNHAC_04133	alsS	acetolactate synthase AlsS
**contig_1**	cds	363390	363479	+	GLNHAC_04134	alsR	acetoin biosynthesis transcriptional regulator AlsR
**contig_1**	cds	363483	363592	-	GLNHAC_04135	cotH	Inner spore coat protein H
**contig_1**	cds	363606	363666	+	GLNHAC_04136	cotG	spore coat protein CotG
**contig_1**	cds	363683	363745	+	GLNHAC_04137	ywrF	Uncharacterized protein YwrF
**contig_1**	cds	363762	363796	+	GLNHAC_04138		DUF4181 domain-containing protein
**contig_1**	cds	363797	363954	-	GLNHAC_04139	ggt	gamma-glutamyltransferase
**contig_1**	cds	363976	364023	+	GLNHAC_04140	ywrC	chromate efflux transcriptional regulator ChrS
**contig_1**	cds	364025	364084	+	GLNHAC_04141	chrB	chromate efflux transporter subunit ChrB
**contig_1**	cds	364084	364137	+	GLNHAC_04142	chrA	chromate resistance efflux protein ChrA
**contig_1**	cds	364140	364162	-	GLNHAC_04143	ywqO	Uncharacterized protein YwqO
**contig_1**	cds	364162	364216	-	GLNHAC_04144	ywqN	Putative NAD(P)H-dependent FMN-containing oxidoreductase
**contig_1**	cds	364229	364317	+	GLNHAC_04145	ywqM	Uncharacterized HTH-type transcriptional regulator YwqM
**contig_1**	cds	364323	364552	-	GLNHAC_04146		Glycoside hydrolase family 127 protein
**contig_1**	cds	364567	364654	+	GLNHAC_04147		Transcriptional regulator, putative
**contig_1**	cds	364657	364729	-	GLNHAC_04148		endonuclease V
**contig_1**	cds	364757	364790	-	GLNHAC_04149		hypothetical protein
**contig_1**	cds	364813	364854	-	GLNHAC_04150		(pseudo) SMI1/KNR4 family protein
**contig_1**	cds	364862	364917	-	GLNHAC_04151		DUF4375 domain-containing protein
**contig_1**	cds	364918	364931	-	GLNHAC_04152		hypothetical protein
**contig_1**	cds	364965	365108	-	GLNHAC_04153	yeeF	Ribonuclease YeeF family protein
**contig_1**	cds	365110	365136	-	GLNHAC_04154	ywqI	Protein YwqI
**contig_1**	cds	365137	365179	-	GLNHAC_04155	ywqH	YwqH
**contig_1**	cds	365216	365348	-	GLNHAC_04156	ywqF	UDP-glucose 6-dehydrogenase UglF
**contig_1**	cds	365367	365444	-	GLNHAC_04157		Tyrosine-protein phosphatase
**contig_1**	cds	365449	365520	-	GLNHAC_04158	ywqD	Tyrosine-protein kinase YwqD
**contig_1**	cds	365519	365594	-	GLNHAC_04159	ywqC	protein-tyrosine kinase activator TkmA
**contig_1**	cds	365617	365632	-	GLNHAC_04160	ywzD	Uncharacterized protein YwzD
**contig_1**	cds	365652	365813	+	GLNHAC_04161	ywqB	Uncharacterized protein YwqB
**contig_1**	cds	365812	366033	+	GLNHAC_04162	ywqA	Uncharacterized ATP-dependent helicase YwqA
**contig_1**	cds	366030	366062	+	GLNHAC_04163		hypothetical protein
**contig_1**	cds	366061	366089	+	GLNHAC_04164		(pseudo) Helicase C-terminal domain-containing protein
**contig_1**	cds	366101	366187	-	GLNHAC_04165	ywpJ	Phosphatase YwpJ
**contig_1**	cds	366188	366265	-	GLNHAC_04166	glcR	transcriptional regulator GlcR
**contig_1**	cds	366288	366322	-	GLNHAC_04167	ssbB	Single-stranded DNA-binding protein B
**contig_1**	cds	366330	366368	-	GLNHAC_04168	ywpG	Protein YwpG
**contig_1**	cds	366383	366427	+	GLNHAC_04169	ywpF	Uncharacterized protein YwpF
**contig_1**	cds	366441	366505	-	GLNHAC_04170		Class A sortase
**contig_1**	cds	366544	367031	-	GLNHAC_04171		Gram-positive cocci surface proteins LPxTG domain-containi
**contig_1**	cds	367027	367085	-	GLNHAC_04172		hypothetical protein
**contig_1**	cds	367142	367250	-	GLNHAC_04173		Two-component response regulator
**contig_1**	cds	367268	367549	+	GLNHAC_04174		histidine kinase
**contig_1**	cds	367580	367619	-	GLNHAC_04175	mscL	large conductance mechanosensitive channel protein MscL
**contig_1**	cds	367626	367673	-	GLNHAC_04176	fabZ	3-hydroxyacyl-ACP dehydratase FabZ
**contig_1**	cds	367688	367794	+	GLNHAC_04177	rapD	aspartate phosphatase RapD
**contig_1**	cds	367796	367877	-	GLNHAC_04178	flhP	flagellar hook-basal body complex protein FlhP
**contig_1**	cds	367881	367966	-	GLNHAC_04179	flhO	flagellar hook-basal body complex protein FlhO
**contig_1**	cds	367978	368078	-	GLNHAC_04180	mbl	cell shape-determining protein Mbl
**contig_1**	cds	368096	368124	-	GLNHAC_04181	spoIIID	sporulation transcriptional regulator SpoIIID
**contig_1**	cds	368159	368200	+	GLNHAC_04182	ywoH	Uncharacterized HTH-type transcriptional regulator YwoH
**contig_1**	cds	368202	368321	+	GLNHAC_04183	ywoG	Uncharacterized MFS-type transporter YwoG
**contig_1**	cds	368331	368472	-	GLNHAC_04184	ywoF	Uncharacterized protein YwoF
**contig_1**	cds	368482	368629	-	GLNHAC_04185	pucI	allantoin permease
**contig_1**	cds	368647	368783	-	GLNHAC_04186	ywoD	Uncharacterized MFS-type transporter YwoD
**contig_1**	cds	368783	368840	-	GLNHAC_04187	ywoC	hydrolase
**contig_1**	cds	368859	368905	-	GLNHAC_04188	ywoB	Uncharacterized protein YwoB
**contig_1**	cds	368932	369054	+	GLNHAC_04189	nrgA	ammonium transporter AmtB
**contig_1**	cds	369055	369090	+	GLNHAC_04190	nrgB	Nitrogen regulatory PII-like protein
**contig_1**	cds	369108	369166	+	GLNHAC_04191	bcrC	undecaprenyl-diphosphate phosphatase BcrC
**contig_1**	cds	369169	369212	-	GLNHAC_04192	ywnJ	Sporulation VanZ like family protein YwnJ
**contig_1**	cds	369224	369311	-	GLNHAC_04193	spoIIQ	stage II sporulation protein spoIIQ
**contig_1**	cds	369324	369374	+	GLNHAC_04194	ywnH	Putative phosphinothricin acetyltransferase YwnH
**contig_1**	cds	369374	369425	+	GLNHAC_04195		DUF5362 domain-containing protein
**contig_1**	cds	369451	369534	+	GLNHAC_04196		(pseudo) AFP-like domain-containing protein
**contig_1**	cds	369532	369630	+	GLNHAC_04197		SAF domain-containing protein
**contig_1**	cds	369643	369687	-	GLNHAC_04198	ywnF	Uncharacterized protein YwnF
**contig_1**	cds	369707	369856	+	GLNHAC_04199	cls	cardiolipin synthase
**contig_1**	cds	369858	369935	-	GLNHAC_04200		Transcriptional regulator
**contig_1**	cds	369950	369989	+	GLNHAC_04201	ywnC	Uncharacterized protein YwnC
**contig_1**	cds	370004	370056	-	GLNHAC_04202	ywnB	(pseudo) Uncharacterized protein YwnB
**contig_1**	cds	370063	370103	-	GLNHAC_04203	ywnA	Putative HTH-type transcriptional regulator YwnA
**contig_1**	cds	370117	370287	-	GLNHAC_04204	ureC	urease subunit alpha
**contig_1**	ncRNA	370243	370273	+	GLNHAC_04205	5_ureB_sRNA	5' ureB small RNA
**contig_1**	cds	370287	370325	-	GLNHAC_04206	ureB	urease subunit beta
**contig_1**	cds	370324	370356	-	GLNHAC_04207	ureA	urease subunit gamma
**contig_1**	cds	370366	370436	-	GLNHAC_04208	urtE	urea ABC transporter ATP-binding subunit UrtE
**contig_1**	cds	370436	370511	-	GLNHAC_04209	urtD	urea ABC transporter ATP-binding protein UrtD
**contig_1**	cds	370508	370615	-	GLNHAC_04210	urtC	urea ABC transporter permease subunit UrtC
**contig_1**	cds	370616	370707	-	GLNHAC_04211	urtB	urea ABC transporter permease subunit UrtB
**contig_1**	cds	370710	370821	-	GLNHAC_04212	urtA	urea ABC transporter substrate-binding protein
**contig_1**	cds	370820	370835	-	GLNHAC_04213		hypothetical protein
**contig_1**	cds	370856	370875	-	GLNHAC_04214	csbD	CsbD family protein
**contig_1**	cds	370882	370930	-	GLNHAC_04215		Integral inner membrane protein
**contig_1**	cds	370947	371060	-	GLNHAC_04216	rapB	response regulator aspartate phosphatase RapB
**contig_1**	cds	371080	371182	-	GLNHAC_04217	moaA	GTP 3',8-cyclase MoaA
**contig_1**	cds	371184	371263	-	GLNHAC_04218	fdhD	formate dehydrogenase accessory sulfurtransferase FdhD
**contig_1**	cds	371273	371289	-	GLNHAC_04219	ywmE	Uncharacterized protein YwmE
**contig_1**	cds	371298	371366	-	GLNHAC_04220	ywmD	Uncharacterized protein YwmD
**contig_1**	cds	371398	371466	-	GLNHAC_04221	ywmC	Uncharacterized protein YwmC
**contig_1**	cds	371505	371608	-	GLNHAC_04222	spoIID	stage II sporulation protein D
**contig_1**	cds	371628	371759	-	GLNHAC_04223	murA	UDP-N-acetylglucosamine 1-carboxyvinyltransferase
**contig_1**	cds	371762	371836	-	GLNHAC_04224	ywmB	Uncharacterized protein YwmB
**contig_1**	cds	371850	371873	-	GLNHAC_04225	ywzB	Uncharacterized membrane protein YwzB
**contig_1**	cds	371890	371938	+	GLNHAC_04226	ywmA	Uncharacterized protein YwmA
**contig_1**	cds	371941	371981	-	GLNHAC_04227	atpC	F0F1 ATP synthase subunit epsilon
**contig_1**	cds	371983	372126	-	GLNHAC_04228	atpD	F0F1 ATP synthase subunit beta
**contig_1**	cds	372128	372214	-	GLNHAC_04229	atpG	ATP synthase F1 subunit gamma
**contig_1**	cds	372222	372314	-	GLNHAC_04230	atpA	(pseudo) F0F1 ATP synthase subunit alpha
**contig_1**	cds	372309	372332	-	GLNHAC_04231		ATPase F1/V1/A1 complex alpha/beta subunit nucleotide-bind
**contig_1**	cds	372326	372345	-	GLNHAC_04232		hypothetical protein
**contig_1**	cds	372349	372373	-	GLNHAC_04233		(pseudo) HAS-barrel domain-containing protein
**contig_1**	cds	372375	372429	-	GLNHAC_04234	atpH	F0F1 ATP synthase subunit delta
**contig_1**	cds	372429	372480	-	GLNHAC_04235	atpF	F0F1 ATP synthase subunit B
**contig_1**	cds	372496	372518	-	GLNHAC_04236	atpE	F0F1 ATP synthase subunit C
**contig_1**	cds	372522	372596	-	GLNHAC_04237	atpB	F0F1 ATP synthase subunit A
**contig_1**	cds	372596	372635	-	GLNHAC_04238	atpI	ATP synthase subunit I

**contig_1**	cds	372677	372740	-	GLNHAC_04239	upp	uracil phosphoribosyltransferase
**contig_1**	cds	372753	372878	-	GLNHAC_04240	glyA	serine hydroxymethyltransferase
**contig_1**	cds	372898	372953	-	GLNHAC_04241	ywlG	UPF0340 protein YwlG
**contig_1**	cds	372954	372999	-	GLNHAC_04242	rpiB	ribose 5-phosphate isomerase B
**contig_1**	cds	373015	373060	-	GLNHAC_04243	ywlE	Protein-arginine-phosphatase
**contig_1**	cds	373067	373123	-	GLNHAC_04244	mntP	Putative manganese efflux pump MntP
**contig_1**	cds	373131	373235	-	GLNHAC_04245	ywlC	L-threonylcarbamoyladenylate synthase
**contig_1**	cds	373251	373297	-	GLNHAC_04246	ywlB	Uncharacterized protein YwlB
**contig_1**	cds	373302	373369	-	GLNHAC_04247	spoIIR	stage II sporulation protein R
**contig_1**	cds	373383	373419	+	GLNHAC_04248	ywlA	UPF0715 membrane protein YwlA
**contig_1**	cds	373421	373450	-	GLNHAC_04249	ywkF	Uncharacterized protein YwkF
**contig_1**	cds	373456	373543	-	GLNHAC_04250	prmC	peptide chain release factor N(5)-glutamine methyltransferase
**contig_1**	cds	373543	373650	-	GLNHAC_04251	prfA	peptide chain release factor 1
**contig_1**	cds	373663	373701	+	GLNHAC_04252	ywkD	VOC family protein
**contig_1**	cds	373713	373769	+	GLNHAC_04253	racA	chromosome-anchoring protein RacA
**contig_1**	cds	373772	373868	-	GLNHAC_04254	ywkB	Uncharacterized transporter YwkB
**contig_1**	cds	373876	374016	-	GLNHAC_04255		NAD-dependent malic enzyme 2
**contig_1**	cds	374086	374133	-	GLNHAC_04256	tdk	(pseudo) thymidine kinase
**contig_1**	cds	374142	374162	-	GLNHAC_04257	rpmE	50S ribosomal protein L31
**contig_1**	ncRNA-region	374163	374166	-			L31-Firmicutes ribosomal protein leader
**contig_1**	cds	374174	374302	-	GLNHAC_04258	rho	transcription termination factor Rho
**contig_1**	cds	374345	374439	-	GLNHAC_04259	glpX	class II fructose-bisphosphatase
**contig_1**	cds	374442	374497	-	GLNHAC_04260	murAB	(pseudo) UDP-N-acetylglucosamine 1-carboxyvinyltransferas
**contig_1**	cds	374495	374571	-	GLNHAC_04261	murAB	(pseudo) UDP-N-acetylglucosamine 1-carboxyvinyltransferas
**contig_1**	cds	374609	374672	-	GLNHAC_04262	fsa	fructose-6-phosphate aldolase
**contig_1**	cds	374684	374712	-	GLNHAC_04263		hypothetical protein
**contig_1**	cds	374730	374770	-	GLNHAC_04264		Fructose-1,6-bisphosphate aldolase
**contig_1**	cds	374788	374825	-	GLNHAC_04265	spo0F	sporulation initiation phosphotransferase Spo0F
**contig_1**	cds	374842	374894	+	GLNHAC_04266	ywjG	Uncharacterized protein YwjG
**contig_1**	cds	374902	375063	-	GLNHAC_04267	pyrG	glutamine hydrolyzing CTP synthase
**contig_1**	ncRNA-region	375073	375081	-			PyrG leader
**contig_1**	cds	375087	375139	-	GLNHAC_04268	rpoE	DNA-directed RNA polymerase subunit delta
**contig_1**	cds	375157	375271	-	GLNHAC_04269	acdA	acyl-CoA dehydrogenase AcdA
**contig_1**	cds	375271	375483	-	GLNHAC_04270	fadF	putative iron-sulfur-binding oxidoreductase FadF
**contig_1**	cds	375498	375543	+	GLNHAC_04271		hypothetical protein
**contig_1**	cds	375543	375618	+	GLNHAC_04272		cardiolipin synthase
**contig_1**	cds	375619	375716	+	GLNHAC_04273	uvsE	UV DNA damage repair endonuclease UvsE
**contig_1**	cds	375724	375751	+	GLNHAC_04274	ywjC	Uncharacterized protein YwjC
**contig_1**	cds	375755	375807	-	GLNHAC_04275		Dihydrofolate reductase
**contig_1**	cds	375815	375898	-	GLNHAC_04276		ATP-binding cassette domain-containing protein
**contig_1**	cds	375922	375957	-	GLNHAC_04277		hypothetical protein
**contig_1**	cds	375989	376139	-	GLNHAC_04278	cls	cardiolipin synthase
**contig_1**	cds	376150	376213	-	GLNHAC_04279	yitT	YitT family protein
**contig_1**	cds	376230	376297	-	GLNHAC_04280	narI	respiratory nitrate reductase subunit gamma
**contig_1**	cds	376297	376352	-	GLNHAC_04281	narJ	nitrate reductase molybdenum cofactor assembly chaperone
**contig_1**	cds	376355	376501	-	GLNHAC_04282	narH	nitrate reductase subunit beta
**contig_1**	cds	376500	376555	-	GLNHAC_04283		(pseudo) Nitrate reductase alpha subunit
**contig_1**	cds	376555	376869	-	GLNHAC_04284		nitrate reductase subunit alpha
**contig_1**	ncRNA	376882	376888	-	GLNHAC_04285	Bacillaceae-1	Bacillaceae-1 RNA
**contig_1**	cds	376890	376938	-	GLNHAC_04286	arfM	Crp/Fnr family transcriptional regulator ArfM
**contig_1**	cds	376952	377024	+	GLNHAC_04287	ywiC	Uncharacterized protein YwiC
**contig_1**	cds	377028	377099	-	GLNHAC_04288	fnr	fumarate/nitrate reduction transcriptional regulator Fnr
**contig_1**	cds	377109	377232	-	GLNHAC_04289	narK	nitrate transporter NarK
**contig_1**	cds	377241	377316	-	GLNHAC_04290		Arginine--tRNA ligase
**contig_1**	cds	377316	377408	-	GLNHAC_04291		Arginine--tRNA ligase
**contig_1**	cds	377408	377451	-	GLNHAC_04292	ywiB	Uncharacterized beta-barrel protein YwiB
**contig_1**	cds	377482	377495	+	GLNHAC_04293	sboA	Subtilosin-A
**contig_1**	cds	377508	377643	+	GLNHAC_04294	albA	subtilosin maturase AlbA
**contig_1**	cds	377644	377660	+	GLNHAC_04295	albB	antilisterial bacteriocin subtilosin biosynthesis protein AlbB
**contig_1**	cds	377660	377732	+	GLNHAC_04296	albC	Putative ABC transporter ATP-binding protein AlbC
**contig_1**	cds	377740	377862	+	GLNHAC_04297	albD	antilisterial bacteriocin subtilosin biosynthesis protein AlbD
**contig_1**	cds	377859	377977	+	GLNHAC_04298	albE	Antilisterial bacteriocin subtilosin biosynthesis protein AlbE
**contig_1**	cds	377978	378106	+	GLNHAC_04299	albF	Putative zinc protease AlbF
**contig_1**	cds	378106	378176	+	GLNHAC_04300	albG	Antilisterial bacteriocin subtilosin biosynthesis protein AlbG
**contig_1**	cds	378189	378325	-	GLNHAC_04301	yncE	YncE family protein
**contig_1**	cds	378348	378406	+	GLNHAC_04302		tetratricopeptide repeat protein
**contig_1**	cds	378497	378544	+	GLNHAC_04303		DUF2712 domain-containing protein
**contig_1**	cds	378559	378638	+	GLNHAC_04304		ABC transporter permease
**contig_1**	cds	378637	378704	+	GLNHAC_04305		ABC transporter permease
**contig_1**	cds	378704	378785	+	GLNHAC_04306		ABC transporter permease subunit
**contig_1**	cds	378785	378850	+	GLNHAC_04307		ABC transporter, ATP-binding protein
**contig_1**	cds	378849	378905	+	GLNHAC_04308		Lipoprotein
**contig_1**	cds	378910	378997	-	GLNHAC_04309	speB	agmatinase
**contig_1**	cds	379003	379086	-	GLNHAC_04310	speE	spermidine synthase
**contig_1**	cds	379107	379314	+	GLNHAC_04311	pbpG	Penicillin-binding protein 2D
**contig_1**	cds	379346	379398	-	GLNHAC_04312	ywhD	Uncharacterized protein YwhD
**contig_1**	cds	379399	379447	-	GLNHAC_04313	ywhC	(pseudo) Putative zinc metalloprotease YwhC
**contig_1**	cds	379446	379465	-	GLNHAC_04314		(pseudo) site-2 protease family protein
**contig_1**	cds	379476	379495	+	GLNHAC_04315	ywhB	2-hydroxymuconate tautomerase
**contig_1**	cds	379499	379541	-	GLNHAC_04316	ywhA	Uncharacterized HTH-type transcriptional regulator YwhA
**contig_1**	cds	379553	379745	-	GLNHAC_04317	thrS	threonine--tRNA ligase
**contig_1**	ncRNA-region	379750	379772	-			T-box leader
**contig_1**	ncRNA-region	379775	379797	-			T-box leader
**contig_1**	ncRNA-region	379801	379823	-			T-box leader
**contig_1**	cds	379805	379821	-	GLNHAC_04318		hypothetical protein
**contig_1**	cds	379835	379885	-	GLNHAC_04319	ywgA	Uncharacterized protein YwgA
**contig_1**	cds	379888	380018	-	GLNHAC_04320	ywfO	Uncharacterized protein YwfO
**contig_1**	cds	380034	380057	-	GLNHAC_04321	ywzC	UPF0741 protein RBAM_034720
**contig_1**	cds	380078	380156	+	GLNHAC_04322	rsfA	prespore-specific transcription regulator RsfA
**contig_1**	cds	380171	380246	-	GLNHAC_04323		carboxylate/amino acid/amine transporter
**contig_1**	cds	380247	380260	-	GLNHAC_04324		hypothetical protein
**contig_1**	cds	380277	380361	-	GLNHAC_04325	ywfL	octanoyl-[GcvH]:protein N-octanoyltransferase
**contig_1**	cds	380366	380456	-	GLNHAC_04326	cysL	cysJI operon transcriptional regulator CysL
**contig_1**	cds	380470	380567	-	GLNHAC_04327	pta	phosphate acetyltransferase
**contig_1**	cds	380594	380671	+	GLNHAC_04328	hemQ	hydrogen peroxide-dependent heme synthase
**contig_1**	cds	380684	380762	+	GLNHAC_04329	bacG	NADPH-dependent reductase BacG
**contig_1**	cds	380763	380883	-	GLNHAC_04330	bacF	transaminase BacF
**contig_1**	cds	380883	381002	-	GLNHAC_04331	bacE	bacilysin exporter BacE
**contig_1**	cds	381002	381143	-	GLNHAC_04332	bacD	alanine--anticapsin ligase
**contig_1**	cds	381145	381221	-	GLNHAC_04333	bacC	dihydroanticapsin 7-dehydrogenase
**contig_1**	cds	381222	381292	-	GLNHAC_04334	bacB	3-((4R)-4-hydroxycyclohexa-1,5-dien-1-yl)-2-oxopropanoate
**contig_1**	cds	381291	381353	-	GLNHAC_04335	bacA	prephenate decarboxylase
**contig_1**	cds	381368	381492	-	GLNHAC_04336	ywfA	Uncharacterized MFS-type transporter YwfA
**contig_1**	cds	381507	381654	-	GLNHAC_04337	rocC	Amino-acid permease RocC
**contig_1**	cds	381654	381692	-	GLNHAC_04338		hypothetical protein
**contig_1**	cds	381691	381824	-	GLNHAC_04339	rocB	Protein RocB
**contig_1**	cds	381832	381986	-	GLNHAC_04340	pruA	L-glutamate gamma-semialdehyde dehydrogenase
**contig_1**	cds	382010	382138	-	GLNHAC_04341	rocG	glutamate dehydrogenase
**contig_1**	cds	382156	382202	-	GLNHAC_04342	bslB	biofilm-surface layer protein BslB
**contig_1**	cds	382234	382280	-	GLNHAC_04343		Spore coat protein
**contig_1**	cds	382279	382364	-	GLNHAC_04344	rfbD	dTDP-4-dehydrorhamnose reductase
**contig_1**	cds	382366	382460	-	GLNHAC_04345	rfbB	dTDP-glucose 4,6-dehydratase
**contig_1**	cds	382460	382507	-	GLNHAC_04346		glucose-1-phosphate thymidylyltransferase
**contig_1**	cds	382503	382535	-	GLNHAC_04347		hypothetical protein
**contig_1**	cds	382572	382609	-	GLNHAC_04348		hypothetical protein
**contig_1**	cds	382605	382639	-	GLNHAC_04349		hypothetical protein
**contig_1**	cds	382661	382711	-	GLNHAC_04350		(pseudo) Putative glycosyltransferase
**contig_1**	cds	382711	382823	-	GLNHAC_04351	spsE	Spore coat polysaccharide biosynthesis protein SpsE
**contig_1**	cds	382823	382910	-	GLNHAC_04352	spsD	Spore coat polysaccharide biosynthesis protein SpsD
**contig_1**	cds	382910	383027	-	GLNHAC_04353	spsC	Spore coat polysaccharide biosynthesis protein SpsC
**contig_1**	cds	383029	383062	-	GLNHAC_04354		hypothetical protein
**contig_1**	cds	383060	383167	-	GLNHAC_04355	spsB	(pseudo) Spore coat polysaccharide biosynthesis protein Sps
**contig_1**	cds	383172	383249	-	GLNHAC_04356	spsA	spore coat dTDP-glycosyltransferase SpsA
**contig_1**	cds	383281	383334	+	GLNHAC_04357	gerQ	spore coat protein GerQ
**contig_1**	cds	383339	383376	-	GLNHAC_04358	ywdK	UPF0382 membrane protein YwdK
**contig_1**	cds	383382	383514	-	GLNHAC_04359	ywdJ	Putative purine permease YwdJ
**contig_1**	cds	383516	383548	-	GLNHAC_04360	ywdI	Uncharacterized protein YwdI
**contig_1**	cds	383565	383702	+	GLNHAC_04361	ywdH	Putative aldehyde dehydrogenase YwdH
**contig_1**	cds	383705	383772	-	GLNHAC_04362	ung	uracil-DNA glycosylase
**contig_1**	cds	383774	383854	-	GLNHAC_04363	ywdF	Uncharacterized glycosyltransferase YwdF
**contig_1**	cds	383863	383917	-	GLNHAC_04364	ywdE	Uncharacterized protein YwdE
**contig_1**	cds	383921	383971	-	GLNHAC_04365		permease prefix domain 1-containing protein
**contig_1**	cds	383967	383985	-	GLNHAC_04366		hypothetical protein
**contig_1**	cds	383984	384018	-	GLNHAC_04367	ywzG	Putative DNA-binding protein YwzG
**contig_1**	cds	384033	384114	+	GLNHAC_04368	pdxK	bifunctional hydroxymethylpyrimidine kinase/phosphomethylp
**contig_1**	cds	384123	384148	-	GLNHAC_04369	ywdA	Uncharacterized protein YwdA
**contig_1**	cds	384170	384301	-	GLNHAC_04370	sacA	Sucrose-6-phosphate hydrolase
**contig_1**	cds	384301	384439	-	GLNHAC_04371	sacP	PTS system sucrose transporter subunit IIBC

contig_1	cds	384455	384536	+	GLNHAC_04372	ywcJ	Uncharacterized transporter YwcJ
contig_1	cds	384550	384633	-	GLNHAC_04373	sacT	sac operon transcriptional antiterminator SacT
contig_1	cds	384637	384668	-	GLNHAC_04374	ywcI	Uncharacterized protein YwcI
contig_1	cds	384720	384962	+	GLNHAC_04375	vpr	serine protease Vpr
contig_1	cds	384966	385003	-	GLNHAC_04376		Luciferase-like domain-containing protein
contig_1	cds	385002	385066	-	GLNHAC_04377	msnO8	MsnO8 family LLM class oxidoreductase
contig_1	cds	385084	385159	-	GLNHAC_04378	nfsA	oxygen-insensitive NADPH nitroreductase
contig_1	cds	385169	385287	-	GLNHAC_04379	rodA	cell shape-determining peptidoglycan glycosyltransferase Ro
contig_1	cds	385309	385421	-	GLNHAC_04380		N-acetyltransferase domain-containing protein
contig_1	cds	385446	385472	+	GLNHAC_04381	ywcE	spore morphogenesis/germination protein YwcE
contig_1	cds	385477	385514	-	GLNHAC_04382	qoxD	cytochrome aa3 quinol oxidase subunit IV
contig_1	cds	385514	385576	-	GLNHAC_04383	qoxC	cytochrome aa3 quinol oxidase subunit III
contig_1	cds	385577	385772	-	GLNHAC_04384	qoxB	cytochrome aa3 quinol oxidase subunit I
contig_1	cds	385775	385875	-	GLNHAC_04385	qoxA	cytochrome aa3 quinol oxidase subunit II
contig_1	cds	385922	385947	+	GLNHAC_04386	ywzA	UPF0410 protein YwzA
contig_1	cds	385954	385985	-	GLNHAC_04387		hypothetical protein
contig_1	cds	386034	386108	-	GLNHAC_04388	galT	(pseudo) UDP-glucose--hexose-1-phosphate uridylyltransfera
contig_1	cds	386109	386226	-	GLNHAC_04389	galK	galactokinase
contig_1	cds	386234	386275	-	GLNHAC_04390	ywcD	Uncharacterized membrane protein YwcD
contig_1	cds	386274	386341	-	GLNHAC_04391	ywcC	Uncharacterized HTH-type transcriptional regulator YwcC
contig_1	cds	386377	386393	+	GLNHAC_04392	slrA	Transcriptional regulator SlrA
contig_1	cds	386437	386468	+	GLNHAC_04393	ywcB	Uncharacterized protein YwcB
contig_1	cds	386467	386522	+	GLNHAC_04394	actP	Cation/acetate symporter ActP
contig_1	cds	386516	386622	+	GLNHAC_04395		Truncated sodium-dependent symporter-like protein
contig_1	cds	386626	386686	-	GLNHAC_04396	ywbO	Uncharacterized protein YwbO
contig_1	cds	386707	386820	-	GLNHAC_04397		Deferrochelatase
contig_1	cds	386822	386937	-	GLNHAC_04398	efeM	iron uptake system lipoprotein EfeM
contig_1	cds	386937	387082	-	GLNHAC_04399	efeU	ferrous ion permease EfeU
contig_1	cds	387097	387164	-	GLNHAC_04400	thiE	Thiamine-phosphate synthase
contig_1	cds	387164	387245	-	GLNHAC_04401	thiM	hydroxyethylthiazole kinase
contig_1	cds	387246	387337	-	GLNHAC_04402	cidR	cidABC operon transcriptional activator CidR
contig_1	cds	387347	387386	+	GLNHAC_04403	cidA	Holin-like protein CidA
contig_1	cds	387384	387452	+	GLNHAC_04404	ywbG	CidB/LrgB family autolysis modulator
contig_1	cds	387463	387532	+	GLNHAC_04405		MFS transporter
contig_1	cds	387529	387572	+	GLNHAC_04406		MFS transporter
contig_1	cds	387575	387595	+	GLNHAC_04407	ywbE	Uncharacterized protein YwbE
contig_1	cds	387598	387717	-	GLNHAC_04408	ywbD	Putative ribosomal RNA large subunit methyltransferase Ywbl
contig_1	cds	387729	387767	+	GLNHAC_04409	glxA	glyoxalase GlxA
contig_1	cds	387771	387838	-	GLNHAC_04410		DUF2711 domain-containing protein
contig_1	cds	387849	387981	-	GLNHAC_04411	celB	PTS cellobiose transporter subunit IIC
contig_1	cds	388004	388197	+	GLNHAC_04412		Minor extracellular protease Epr
contig_1	cds	388242	388380	+	GLNHAC_04413	sacX	SacY negative regulator SacX
contig_1	cds	388385	388469	+	GLNHAC_04414	sacY	Levansucrase and sucrase synthesis operon antiterminator
contig_1	cds	388475	388561	-	GLNHAC_04415	gspA	General stress protein A
contig_1	cds	388572	388643	-	GLNHAC_04416		TIGR02206 family membrane protein
contig_1	cds	388658	388687	+	GLNHAC_04417	ywaE	(pseudo) Uncharacterized HTH-type transcriptional regulator
contig_1	cds	388687	388718	-	GLNHAC_04418		hypothetical protein
contig_1	ncRNA-region	388709	388730	+			T-box leader
contig_1	cds	388735	388859	+	GLNHAC_04419	tyrS	tyrosine--tRNA ligase
contig_1	cds	388874	389011	+	GLNHAC_04420	ywaD	Aminopeptidase YwaD
contig_1	cds	389014	389077	-	GLNHAC_04421	ywaC	GTP pyrophosphokinase YwaC
contig_1	cds	389091	389185	-	GLNHAC_04422	menA	1,4-dihydroxy-2-naphthoate polyprenyltransferase
contig_1	cds	389221	389243	+	GLNHAC_04423	ywzH	Uncharacterized membrane protein YwzH
contig_1	cds	389245	389396	+	GLNHAC_04424	dltA	D-alanine--poly(phosphoribitol) ligase subunit DltA
contig_1	cds	389396	389514	+	GLNHAC_04425	dltB	D-alanyl-lipoteichoic acid biosynthesis protein DltB
contig_1	cds	389516	389540	+	GLNHAC_04426	dltC	D-alanine--poly(phosphoribitol) ligase subunit DltC
contig_1	cds	389540	389658	+	GLNHAC_04427	dltD	D-alanyl-lipoteichoic acid biosynthesis protein DltD
contig_1	cds	389666	389742	+	GLNHAC_04428	dltE	Uncharacterized oxidoreductase DltE
contig_1	cds	389756	389865	+	GLNHAC_04429	ilvK	branched-chain amino acid aminotransferase
contig_1	cds	389869	389933	-	GLNHAC_04430		Glycosyl hydrolase family 4 C-terminal domain-containing pro
contig_1	cds	389933	390002	-	GLNHAC_04431		6-phospho-beta-glucosidase
contig_1	cds	390002	390028	-	GLNHAC_04432		PTS lactose/cellobiose transporter subunit IIA
contig_1	cds	390037	390100	-	GLNHAC_04433		PTS transporter subunit EIIC
contig_1	cds	390100	390172	-	GLNHAC_04434		PTS EIIC type-3 domain-containing protein
contig_1	cds	390174	390205	-	GLNHAC_04435	licB	PTS lichenan transporter subunit IIB
contig_1	cds	390218	390410	-	GLNHAC_04436	licR	transcriptional regulator LicR
contig_1	cds	390426	390442	-	GLNHAC_04437	yxzF	Uncharacterized protein YxzF
contig_1	cds	390445	390504	-	GLNHAC_04438	yxlJ	DNA-3-methyladenine glycosylase
contig_1	cds	390517	390681	+	GLNHAC_04439	katX	catalase KatX
contig_1	cds	390691	390812	+	GLNHAC_04440	yxlH	Uncharacterized MFS-type transporter YxlH
contig_1	cds	390811	390889	-	GLNHAC_04441	yxlG	Uncharacterized transmembrane protein YxlG
contig_1	cds	390890	390977	-	GLNHAC_04442	yxlF	Uncharacterized ABC transporter ATP-binding protein YxlF
contig_1	cds	390978	390997	-	GLNHAC_04443	yxlE	Negative regulatory protein YxlE
contig_1	cds	390996	391017	-	GLNHAC_04444		Conserved domain protein
contig_1	cds	391017	391049	-	GLNHAC_04445	yxlC	YxlC family protein
contig_1	cds	391058	391099	-	GLNHAC_04446		hypothetical protein
contig_1	cds	391120	391257	+	GLNHAC_04447	yxlA	Putative purine-cytosine permease YxlA
contig_1	cds	391259	391342	-	GLNHAC_04448	nnrD	ADP-dependent (S)-NAD(P)H-hydrate dehydratase
contig_1	cds	391350	391513	-	GLNHAC_04449	cydC	thiol reductant ABC exporter subunit CydC
contig_1	cds	391510	391624	-	GLNHAC_04450	cydC	Transport ATP-binding protein CydC
contig_1	cds	391632	391648	+	GLNHAC_04451		hypothetical protein
contig_1	cds	391642	391693	-	GLNHAC_04452		ABC transporter ATP-binding protein
contig_1	cds	391693	391794	-	GLNHAC_04453	cydB	cytochrome d ubiquinol oxidase subunit II
contig_1	cds	391793	391933	-	GLNHAC_04454	cydA	cytochrome ubiquinol oxidase subunit I
contig_1	cds	391997	392091	+	GLNHAC_04455	cimH	(pseudo) Citrate/malate-proton symporter
contig_1	cds	392091	392121	+	GLNHAC_04456		Citrate carrier protein
contig_1	cds	392136	392308	+	GLNHAC_04457		M56 family metallopeptidase
contig_1	cds	392311	392333	+	GLNHAC_04458	yxzE	Uncharacterized protein YxzE
contig_1	cds	392332	392416	-	GLNHAC_04459		Polysaccharide deacetylase family protein
contig_1	cds	392434	392471	+	GLNHAC_04460		BIG2 domain-containing protein
contig_1	cds	392477	392486	-	GLNHAC_04461		hypothetical protein
contig_1	cds	392486	392549	-	GLNHAC_04462		ZIP family metal transporter
contig_1	cds	392560	392670	-	GLNHAC_04463	msmX	maltodextrin ABC transporter ATP-binding protein MsmX
contig_1	cds	392682	392771	-	GLNHAC_04464	pucR	PucR C-terminal helix-turn-helix domain-containing protein
contig_1	cds	392790	392935	+	GLNHAC_04465	aldY	Putative aldehyde dehydrogenase AldY
contig_1	cds	392939	393023	-	GLNHAC_04466	yxkD	UPF0750 membrane protein YxkD
contig_1	ncRNA-region	393030	393040	-			Guanidine-I riboswitch aptamer
contig_1	cds	393068	393132	+	GLNHAC_04467	yxkC	Uncharacterized protein YxkC
contig_1	cds	393140	393242	-	GLNHAC_04468	galE	UDP-glucose 4-epimerase GalE
contig_1	cds	393268	393315	+	GLNHAC_04469	yxjN	Uncharacterized protein YxjN
contig_1	cds	393328	393439	+	GLNHAC_04470	yxjM	Sensor histidine kinase YxjM
contig_1	cds	393438	393503	+	GLNHAC_04471	yxjL	two-component system response regulator YxJL
contig_1	cds	393508	393631	+	GLNHAC_04472	pepT	peptidase T
contig_1	cds	393728	393777	+	GLNHAC_04473	yxjI	Uncharacterized protein YxjI
contig_1	ncRNA-region	393783	393794	+			SAM riboswitch aptamer (S box leader)
contig_1	cds	393795	393916	+	GLNHAC_04474	yxjH	Uncharacterized protein YxjH
contig_1	ncRNA-region	393923	393933	+			SAM riboswitch aptamer (S box leader)
contig_1	cds	393941	394004	+	GLNHAC_04475		hypothetical protein
contig_1	cds	394015	394041	+	GLNHAC_04476		hypothetical protein
contig_1	cds	394074	394137	-	GLNHAC_04477	yxjF	Uncharacterized oxidoreductase YxjF
contig_1	cds	394138	394204	-	GLNHAC_04478	scoB	putative succinyl-CoA:3-ketoacid coenzyme A transferase su
contig_1	cds	394204	394275	-	GLNHAC_04479	scoA	putative succinyl-CoA:3-ketoacid coenzyme A transferase su
contig_1	cds	394278	394374	-	GLNHAC_04480	gntP	GntP family permease
contig_1	cds	394379	394437	+	GLNHAC_04481		hypothetical protein
contig_1	cds	394435	394521	-	GLNHAC_04482		Methyltransferase domain-containing protein
contig_1	ncRNA-region	394560	394570	+			Purine riboswitch aptamer
contig_1	cds	394583	394655	+	GLNHAC_04483	nupC	Nucleoside transporter NupC family protein
contig_1	cds	394655	394702	+	GLNHAC_04484		(pseudo) nucleoside transporter C-terminal domain-containin
contig_1	cds	394706	394773	-	GLNHAC_04485		DUF3298 DUF4163 domains-containing protein
contig_1	cds	394788	394817	-	GLNHAC_04486	yxiS	Uncharacterized protein YxiS
contig_1	cds	394822	395028	-	GLNHAC_04487	katE	Catalase-2
contig_1	cds	395048	395176	+	GLNHAC_04488	citH	citrate transporter CitH
contig_1	cds	395186	395243	+	GLNHAC_04489	yetF	YetF C-terminal domain-containing protein
contig_1	cds	395247	395358	-	GLNHAC_04490		hypothetical protein
contig_1	cds	395368	395510	-	GLNHAC_04491		Glycoside hydrolase family 1 protein
contig_1	cds	395512	395695	-	GLNHAC_04492		PTS beta-glucoside transporter subunit IIABC
contig_1	cds	395726	395810	-	GLNHAC_04493	licT	transcriptional antiterminator LicT
contig_1	cds	395819	395841	-	GLNHAC_04494		hypothetical protein
contig_1	cds	395841	395887	-	GLNHAC_04495		(pseudo) Lipoprotein
contig_1	cds	395908	396036	+	GLNHAC_04496	yxiO	Uncharacterized MFS-type transporter YxiO
contig_1	cds	396038	396182	-	GLNHAC_04497	dbpA	ATP-dependent RNA helicase DbpA
contig_1	cds	396190	396305	-	GLNHAC_04498	yxiM	Uncharacterized esterase YxiM
contig_1	cds	396322	396355	+	GLNHAC_04499		Regulatory protein
contig_1	cds	396361	396411	+	GLNHAC_04500		DUF2812 domain-containing protein
contig_1	cds	396413	396456	-	GLNHAC_04501		hypothetical protein
contig_1	cds	396484	396510	-	GLNHAC_04502		Transporter, Drug/Metabolite Exporter family
contig_1	cds	396527	396555	-	GLNHAC_04503		(pseudo) hypothetical protein
contig_1	cds	396588	396656	-	GLNHAC_04504	wapI	Immunity protein WapI
contig_1	cds	396659	396821	-	GLNHAC_04505		Rhs family protein

**contig_1**	cds	396822	397359	-	GLNHAC_04506		Rhs family protein
**contig_1**	cds	397376	397449	-	GLNHAC_04507	yxxF	(pseudo) Uncharacterized transporter YxxF
**contig_1**	cds	397484	397529	-	GLNHAC_04508	yxiE	Universal stress protein YxiE
**contig_1**	cds	397539	397680	-	GLNHAC_04509	bglH	aryl-phospho-beta-d-glucosidase
**contig_1**	cds	397691	397866	-	GLNHAC_04510	bglP	PTS system beta-glucoside-specific EIIBCA component
**contig_1**	cds	397898	397924	-	GLNHAC_04511		hypothetical protein
**contig_1**	cds	397970	398003	-	GLNHAC_04512		Imm8 family immunity protein
**contig_1**	cds	398004	398166	-	GLNHAC_04513		LXG domain-containing protein
**contig_1**	cds	398168	398195	-	GLNHAC_04514	yxiC	Protein YxiC
**contig_1**	cds	398196	398233	-	GLNHAC_04515	yxiB	Protein YxiB
**contig_1**	cds	398263	398405	-	GLNHAC_04516	abnB	arabinan endo-1,5-alpha-L-arabinosidase AbnB
**contig_1**	cds	398412	398431	-	GLNHAC_04517		(pseudo) hypothetical protein
**contig_1**	cds	398427	398511	-	GLNHAC_04518		Gfo/Idh/MocA family oxidoreductase
**contig_1**	cds	398536	398580	+	GLNHAC_04519	hutP	hut operon transcriptional regulator HutP
**contig_1**	cds	398592	398744	+	GLNHAC_04520	hutH	histidine ammonia-lyase
**contig_1**	cds	398744	398873	+	GLNHAC_04521	hutU	(pseudo) urocanate hydratase
**contig_1**	cds	398869	398910	+	GLNHAC_04522	hutU	(pseudo) urocanate hydratase
**contig_1**	cds	398911	398939	+	GLNHAC_04523		amidohydrolase family protein
**contig_1**	cds	398939	399015	+	GLNHAC_04524		Imidazolonepropionase
**contig_1**	cds	399015	399037	+	GLNHAC_04525		hypothetical protein
**contig_1**	cds	399037	399133	+	GLNHAC_04526	hutG	formimidoylglutamase
**contig_1**	cds	399140	399283	+	GLNHAC_04527	hutM	Putative histidine permease
**contig_1**	cds	399287	399417	-	GLNHAC_04528	pdp	pyrimidine-nucleoside phosphorylase
**contig_1**	cds	399420	399538	-	GLNHAC_04529	nupC	nucleoside permease NupC
**contig_1**	cds	399548	399615	-	GLNHAC_04530	deoC	deoxyribose-phosphate aldolase
**contig_1**	cds	399625	399720	-	GLNHAC_04531	deoR	DNA-binding transcriptional repressor DeoR
**contig_1**	cds	399733	399816	-	GLNHAC_04532		DUF1206 domain-containing protein
**contig_1**	cds	399826	399900	-	GLNHAC_04533	eutH	ethanolamine utilization protein EutH
**contig_1**	cds	399901	399934	-	GLNHAC_04534		hypothetical protein
**contig_1**	cds	399941	400075	-	GLNHAC_04535	yxeQ	Uncharacterized protein YxeQ
**contig_1**	cds	400074	400189	-	GLNHAC_04536	sndB	N-acetyl-sulfur-metabolite deacetylase SndB
**contig_1**	cds	400190	400265	-	GLNHAC_04537	yxeO	putative amino-acid import ATP-binding protein YxeO
**contig_1**	cds	400267	400334	-	GLNHAC_04538	yxeN	putative amino-acid permease protein YxeN
**contig_1**	cds	400336	400416	-	GLNHAC_04539	yxeM	putative amino-acid-binding protein YxeM
**contig_1**	cds	400418	400468	-	GLNHAC_04540	scmL	S-(2-succino)cysteine N-acetyltransferase
**contig_1**	cds	400469	400602	-	GLNHAC_04541	scmK	N-acetyl-S-(2-succino)cysteine monooxygenase
**contig_1**	cds	400638	400736	-	GLNHAC_04542		Penicillin acylase
**contig_1**	cds	400776	400858	-	GLNHAC_04543	yidA	sugar-phosphatase
**contig_1**	cds	400860	400917	-	GLNHAC_04544	yxeG	Uncharacterized protein YxeG
**contig_1**	cds	400916	400958	-	GLNHAC_04545		Lipoprotein, putative
**contig_1**	cds	400969	401006	+	GLNHAC_04546	yxeE	Uncharacterized protein YxeE
**contig_1**	cds	401031	401066	+	GLNHAC_04547	yxeD	Uncharacterized protein YxeD
**contig_1**	cds	401071	401110	-	GLNHAC_04548	yxeC	Uncharacterized protein YxeC
**contig_1**	cds	401128	401225	+	GLNHAC_04549	yxeB	Iron(3+)-hydroxamate-binding protein YxeB
**contig_1**	cds	401229	401264	-	GLNHAC_04550	yxeA	Uncharacterized protein YxeA
**contig_1**	cds	401265	401452	-	GLNHAC_04551	yxdM	ABC transporter permease YxdM
**contig_1**	cds	401449	401527	-	GLNHAC_04552	yxdL	ABC transporter ATP-binding protein YxdL
**contig_1**	cds	401541	401639	-	GLNHAC_04553	yxdK	two-component system sensor histidine kinase YxdK
**contig_1**	cds	401645	401708	-	GLNHAC_04554	yxdJ	Transcriptional regulatory protein YxdJ
**contig_1**	cds	401707	401718	-	GLNHAC_04555		hypothetical protein
**contig_1**	cds	401718	401805	-	GLNHAC_04556	iolJ	6-phospho-5-dehydro-2-deoxy-D-gluconate aldolase
**contig_1**	cds	401808	401891	-	GLNHAC_04557	iolI	2-keto-myo-inositol isomerase
**contig_1**	cds	401900	401987	-	GLNHAC_04558	iolH	Protein IolH
**contig_1**	cds	401989	402092	-	GLNHAC_04559	iolG	bifunctional inositol 2-dehydrogenase/D-chiro-inositol 1-dehy
**contig_1**	cds	402095	402228	-	GLNHAC_04560	iolF	myo-inositol transporter IolF
**contig_1**	cds	402228	402317	-	GLNHAC_04561	iolE	myo-inosose-2 dehydratase
**contig_1**	cds	402319	402510	-	GLNHAC_04562	iolD	3D-(3,5/4)-trihydroxycyclohexane-1,2-dione acylhydrolase (de
**contig_1**	cds	402513	402611	-	GLNHAC_04563	iolC	5-dehydro-2-deoxygluconokinase
**contig_1**	cds	402613	402695	-	GLNHAC_04564	iolB	5-deoxy-glucuronate isomerase
**contig_1**	cds	402702	402849	-	GLNHAC_04565	iolA	methylmalonate-semialdehyde dehydrogenase
**contig_1**	cds	402890	402966	+	GLNHAC_04566	iolR	myo-inositol utilization transcriptional regulator IolR
**contig_1**	cds	402971	403065	+	GLNHAC_04567	iolS	Aldo-keto reductase IolS
**contig_1**	cds	403097	403162	+	GLNHAC_04568	yxcE	Uncharacterized protein YxcE
**contig_1**	cds	403163	403193	+	GLNHAC_04569	yxcD	Uncharacterized protein YxcD
**contig_1**	cds	403216	403355	+	GLNHAC_04570	csbC	putative metabolite transport protein CsbC
**contig_1**	cds	403358	403546	-	GLNHAC_04571	htpG	molecular chaperone HtpG
**contig_1**	cds	403564	403590	-	GLNHAC_04572	yxcA	Uncharacterized protein YxcA
**contig_1**	cds	403601	403683	+	GLNHAC_04573	yxbG	SDR family oxidoreductase
**contig_1**	cds	403708	403724	+	GLNHAC_04574		Lipoprotein
**contig_1**	cds	403768	403882	-	GLNHAC_04575		TetR/AcrR family transcriptional regulator
**contig_1**	cds	403897	404030	+	GLNHAC_04576	aldX	Putative aldehyde dehydrogenase AldX
**contig_1**	cds	404055	404099	+	GLNHAC_04577	yxaI	YxaI
**contig_1**	cds	404113	404156	-	GLNHAC_04578	yxaJ	YxaJ
**contig_1**	cds	404181	404227	+	GLNHAC_04579		RDD family protein
**contig_1**	cds	404230	404351	-	GLNHAC_04580	yxaH	Uncharacterized protein YxaH
**contig_1**	cds	404361	404463	-	GLNHAC_04581	qdoI	Quercetin 2,3-dioxygenase
**contig_1**	cds	404472	404529	-	GLNHAC_04582	yxaF	transcriptional regulator YxaF
**contig_1**	cds	404542	404649	+	GLNHAC_04583	yxnA	Uncharacterized oxidoreductase YxnA
**contig_1**	cds	404655	404698	-	GLNHAC_04584	yxaD	Uncharacterized HTH-type transcriptional regulator YxaD
**contig_1**	cds	404721	404761	+	GLNHAC_04585	yxzK	Putative integral membrane protein YxzK
**contig_1**	cds	404758	404827	+	GLNHAC_04586	yxaC	Uncharacterized protein YxaC
**contig_1**	cds	404848	405005	+	GLNHAC_04587	nhaG	Na+/H+ antiporter
**contig_1**	cds	405009	405112	-	GLNHAC_04588	yxaB	General stress protein 30
**contig_1**	cds	405122	405236	-	GLNHAC_04589		Glycerate kinase 2
**contig_1**	cds	405256	405329	+	GLNHAC_04590	gntR	gluconate operon transcriptional repressor GntR
**contig_1**	cds	405328	405483	+	GLNHAC_04591	gntK	gluconokinase
**contig_1**	cds	405485	405620	+	GLNHAC_04592	gntP	gluconate permease GntP
**contig_1**	cds	405622	405763	+	GLNHAC_04593	gnd	decarboxylating NADP(+)-dependent phosphogluconate dehy
**contig_1**	cds	405815	405871	+	GLNHAC_04594	ahpC	alkyl hydroperoxide reductase subunit C
**contig_1**	cds	405873	406025	+	GLNHAC_04595	ahpF	alkyl hydroperoxide reductase subunit F
**contig_1**	cds	406048	406185	-	GLNHAC_04596		Polysaccharide pyruvyl transferase domain-containing protein
**contig_1**	cds	406218	406362	-	GLNHAC_04597	bglA	6-phospho-beta-glucosidase BglA
**contig_1**	cds	406363	406550	-	GLNHAC_04598		Pts system beta-glucoside-specific eiibca component
**contig_1**	cds	406566	406637	+	GLNHAC_04599	yydK	Uncharacterized HTH-type transcriptional regulator YydK
**contig_1**	cds	406683	406755	-	GLNHAC_04600	yydJ	putative peptide export permease protein YydJ
**contig_1**	cds	406757	406820	-	GLNHAC_04601	yydI	putative peptide export ATP-binding protein YydI
**contig_1**	cds	406835	406911	-	GLNHAC_04602		Peptidase
**contig_1**	cds	406909	407005	-	GLNHAC_04603	yydG	YydG family radical SAM peptide epimerase
**contig_1**	cds	407011	407026	-	GLNHAC_04604	yydF	Putative exported peptide YydF
**contig_1**	cds	407062	407072	-	GLNHAC_04605		hypothetical protein
**contig_1**	cds	407088	407280	+	GLNHAC_04606	fbp	fructose-1,6-bisphosphatase
**contig_1**	cds	407308	407512	-	GLNHAC_04607		Endonuclease GajA/Old nuclease/RecF-like AAA domain-con
**contig_1**	cds	407542	408012	-	GLNHAC_04608	avs4	AVAST type 4 anti-phage nuclease Avs4
**contig_1**	cds	408034	408081	-	GLNHAC_04609	rlmH	23S rRNA (pseudouridine(1915)-N(3))-methyltransferase RlmH
**contig_1**	cds	408090	408107	-	GLNHAC_04610	yyzF	CxxH/CxxC protein
**contig_1**	cds	408151	408273	-	GLNHAC_04611	fdhA	formaldehyde dehydrogenase, glutathione-independent
**contig_1**	cds	408280	408297	-	GLNHAC_04612		Holin
**contig_1**	cds	408316	408334	-	GLNHAC_04613		(pseudo) DUF2651 domain-containing protein
**contig_1**	cds	408336	408452	-	GLNHAC_04614		DUF5050 domain-containing protein
**contig_1**	cds	408453	408527	-	GLNHAC_04615	yycO	Uncharacterized protein YycO
**contig_1**	cds	408542	408589	-	GLNHAC_04616	yycN	Uncharacterized N-acetyltransferase YycN
**contig_1**	cds	408601	408710	+	GLNHAC_04617	rapG	response regulator aspartate phosphatase RapG
**contig_1**	cds	408710	408722	+	GLNHAC_04618	rapG	RapG inhibitor
**contig_1**	cds	408726	408815	-	GLNHAC_04619	rocF	arginase
**contig_1**	cds	408822	408970	-	GLNHAC_04620	rocE	Amino-acid permease RocE
**contig_1**	cds	408985	409108	-	GLNHAC_04621	rocD	ornithine aminotransferase
**contig_1**	cds	409129	409268	+	GLNHAC_04622	rocR	arginine utilization regulatory protein RocR
**contig_1**	cds	409268	409301	-	GLNHAC_04623		histidine kinase
**contig_1**	cds	409339	409355	-	GLNHAC_04624		PDZ domain-containing protein
**contig_1**	cds	409369	409459	-	GLNHAC_04625	yyxA	(pseudo) Uncharacterized serine protease YyxA
**contig_1**	cds	409467	409547	-	GLNHAC_04626	yycJ	Exodeoxyribonuclease YycJ
**contig_1**	cds	409549	409633	-	GLNHAC_04627	yycI	WalRK two-component regulatory system regulator WalI
**contig_1**	cds	409632	409770	-	GLNHAC_04628	yycH	WalRK two-component regulatory system regulator WalH
**contig_1**	cds	409768	409874	-	GLNHAC_04629		histidine kinase
**contig_1**	cds	409877	409951	-	GLNHAC_04630		hypothetical protein
**contig_1**	cds	409952	410023	-	GLNHAC_04631	walR	cell wall metabolism DNA-binding response regulator WalR
**contig_1**	tRNA	410061	410068	-	GLNHAC_04632	trnF	tRNA-Phe(gaa)
**contig_1**	tRNA	410072	410079	-	GLNHAC_04633	trnD	tRNA-Asp(gtc)
**contig_1**	tRNA	410087	410095	-	GLNHAC_04634	trnE	tRNA-Glu(ttc)
**contig_1**	tRNA	410096	410103	-	GLNHAC_04635	trnK	tRNA-Lys(ttt)
**contig_1**	cds	410125	410255	-	GLNHAC_04636	purA	adenylosuccinate synthase
**contig_1**	cds	410275	410317	-	GLNHAC_04637		VOC domain-containing protein
**contig_1**	cds	410329	410465	-	GLNHAC_04638	dnaB	replicative DNA helicase
**contig_1**	cds	410482	410502	+	GLNHAC_04639	yycD	Uncharacterized protein YycD
**contig_1**	cds	410507	410527	-	GLNHAC_04640	yyzB	Uncharacterized protein YyzB
**contig_1**	cds	410539	410553	+	GLNHAC_04641	yycC	Uncharacterized protein YycC
**contig_1**	cds	410560	410581	+	GLNHAC_04642		hypothetical protein
**contig_1**	cds	410584	410681	+	GLNHAC_04643	yycB	Uncharacterized transporter YycB
**contig_1**	cds	410693	410799	+	GLNHAC_04644		Mannosyltransferase

**contig_1**	cds	410816	410883	+	GLNHAC_04645		hypothetical protein
**contig_1**	cds	410952	411150	-	GLNHAC_04646	gdpP	cyclic-di-AMP phosphodiesterase GdpP
**contig_1**	cds	411154	411247	-	GLNHAC_04647	yybS	Uncharacterized protein YybS
**contig_1**	cds	411270	411285	-	GLNHAC_04648	yyzH	Uncharacterized membrane protein YyzH
**contig_1**	cds	411299	411347	+	GLNHAC_04649	cotF	spore coat protein CotF
**contig_1**	cds	411350	411388	-	GLNHAC_04650	yybR	transcriptional regulator HypR
**contig_1**	cds	411409	411502	+	GLNHAC_04651	ppaC	manganese-dependent inorganic pyrophosphatase
**contig_1**	cds	411505	411550	-	GLNHAC_04652	yybP	Uncharacterized lipoprotein YybP
**contig_1**	cds	411551	411562	-	GLNHAC_04653		hypothetical protein
**contig_1**	cds	411593	411724	+	GLNHAC_04654	yybO	Uncharacterized transporter YybO
**contig_1**	cds	411748	411821	+	GLNHAC_04655		HTH merR-type domain-containing protein
**contig_1**	cds	411823	411875	-	GLNHAC_04656		Spore coat protein
**contig_1**	cds	411874	411904	-	GLNHAC_04657		hypothetical protein
**contig_1**	cds	411911	411938	-	GLNHAC_04658		DUF4440 domain-containing protein
**contig_1**	cds	411946	412012	-	GLNHAC_04659	yybB	putative metallo-hydrolase YybB
**contig_1**	cds	412022	412058	+	GLNHAC_04660		Cinnamoyl ester hydrolase
**contig_1**	cds	412074	412119	-	GLNHAC_04661	yybA	Uncharacterized HTH-type transcriptional regulator YybA
**contig_1**	cds	412131	412174	+	GLNHAC_04662		GNAT family N-acetyltransferase
**contig_1**	cds	412174	412235	+	GLNHAC_04663	yitT	YitT family protein
**contig_1**	cds	412251	412298	-	GLNHAC_04664		DMT family transporter
**contig_1**	cds	412300	412311	-	GLNHAC_04665		hypothetical protein
**contig_1**	cds	412311	412343	-	GLNHAC_04666		(pseudo) DMT family transporter
**contig_1**	cds	412351	412382	-	GLNHAC_04667	arsR	ArsR family transcriptional regulator
**contig_1**	cds	412410	412535	+	GLNHAC_04668		thioredoxin domain-containing protein
**contig_1**	cds	412537	412617	+	GLNHAC_04669		Thioredoxin domain-containing protein
**contig_1**	cds	412616	412706	-	GLNHAC_04670	yyaK	Uncharacterized protein YyaK
**contig_1**	cds	412729	412864	+	GLNHAC_04671	yyaJ	Putative metabolite transport protein YyaJ
**contig_1**	cds	412868	412924	-	GLNHAC_04672	maa	putative maltose O-acetyltransferase
**contig_1**	cds	412925	412963	-	GLNHAC_04673		VOC domain-containing protein
**contig_1**	cds	412969	413062	-	GLNHAC_04674	ccpB	Catabolite control protein B
**contig_1**	cds	413068	413144	-	GLNHAC_04675	xth	exodeoxyribonuclease III
**contig_1**	cds	413150	413174	-	GLNHAC_04676	rpsR	30S ribosomal protein S18
**contig_1**	cds	413179	413230	-	GLNHAC_04677	ssbA	Single-stranded DNA-binding protein A
**contig_1**	cds	413234	413263	-	GLNHAC_04678	rpsF	30S ribosomal protein S6
**contig_1**	cds	413274	413384	-	GLNHAC_04679	ychF	redox-regulated ATPase YchF
**contig_1**	cds	413397	413597	-	GLNHAC_04680	yyaE	putative oxidoreductase YyaE
**contig_1**	cds	413602	413623	-	GLNHAC_04681	yyzM	Uncharacterized protein YyzM
**contig_1**	cds	413632	413734	-	GLNHAC_04682	yyaD	Uncharacterized protein YyaD
**contig_1**	cds	413791	413853	+	GLNHAC_04683	yyaC	spore protease YyaC
**contig_1**	cds	413857	413941	-	GLNHAC_04684	spo0J	stage 0 sporulation protein Spo0J
**contig_1**	cds	413941	414017	-	GLNHAC_04685	soj	sporulation initiation inhibitor protein Soj
**contig_1**	cds	414038	414085	+	GLNHAC_04686		PH domain-containing protein
**contig_1**	cds	414090	414175	-	GLNHAC_04687	noc	nucleoid occlusion protein
**contig_1**	cds	414187	414259	-	GLNHAC_04688	rsmG	16S rRNA (guanine(527)-N(7))-methyltransferase RsmG
**contig_1**	cds	414261	414449	-	GLNHAC_04689	mnmG	tRNA uridine-5-carboxymethylaminomethyl(34) synthesis enz
**contig_1**	cds	414451	414589	-	GLNHAC_04690	mnmE	tRNA uridine-5-carboxymethylaminomethyl(34) synthesis GTF
**contig_1**	cds	414621	414683	-	GLNHAC_04691	jag	RNA-binding cell elongation regulator Jag/EloR
**contig_1**	cds	414683	414761	-	GLNHAC_04692	spoIIIJ	YidC family membrane integrase SpoIIIJ
**contig_1**	cds	414767	414811	-	GLNHAC_04693		Ribonuclease P protein component

DbXrefs
BlastRules:WP_003242674, COG:COG0593, COG:L, EC:3.6.4.-, GO:0003677, GO:0003688, GO:0005524, GO:0005737, GO:0005886, GO:0006270, GO:0006275, GO:0016887, KEGG:K02313, RefSeq:WP_003242674.1, SO:
BlastRules:WP_003242509, EC:2.7.7.7, GO:0003677, GO:0003887, GO:0006260, GO:0009360, KEGG:K02338, RefSeq:WP_088680205.1, SO:0001217, UniParc:UPI000B531732, UniRef:UniRef100_UPI000B531732, UniRef:
BlastRules:WP_003226810, GO:0003723, GO:0042254, RefSeq:WP_003219264.1, SO:0001217, UniParc:UPI0001D28CA2, UniRef:UniRef100_A0A9Q4E004, UniRef:UniRef50_P05650, UniRef:UniRef90_P05650
BlastRules:WP_003243515, GO:0000731, GO:0003697, GO:0005524, GO:0005737, GO:0006260, GO:0006281, GO:0006302, GO:0006310, GO:0009432, RefSeq:WP_014112268.1, SO:0001217, UniParc:UPI00022BAD23, U
BlastRules:WP_003219266, KEGG:K24648, RefSeq:WP_003219266.1, SO:0001217, UniParc:UPI000195C52B, UniRef:UniRef100_P37525, UniRef:UniRef50_P37525, UniRef:UniRef90_P37525
BlastRules:WP_003226808, EC:5.6.2.2, GO:0003677, GO:0003918, GO:0005524, GO:0005694, GO:0006265, KEGG:K02470, SO:0001217, UniRef:UniRef50_Q839Z1, UniRef:UniRef90_A0AAP3CQE7
BlastRules:WP_003244540, EC:5.6.2.2, GO:0003677, GO:0003916, GO:0003918, GO:0005524, GO:0005694, GO:0005737, GO:0006261, GO:0006265, GO:0009330, GO:0034335, KEGG:K02469, RefSeq:WP_061187096.1,
GO:0003735, GO:0005840, KEGG:K01977, RFAM:RF00177, SO:0001000
SO:0000263
SO:0000254
GO:0003735, GO:0005840, KEGG:K01980, RFAM:RF02541, SO:0001001
RFAM:RF01690, SO:0000655
GO:0003735, GO:0005840, KEGG:K01985, RFAM:RF00001, SO:0000652
SO:0001217, UniRef:UniRef50_P37526, UniRef:UniRef90_P37526
BlastRules:WP_003226803, COG:COG0517, COG:T, EC:1.1.1.205, GO:0000166, GO:0003938, GO:0006164, GO:0006177, GO:0006183, GO:0046872, KEGG:K00088, RefSeq:WP_014112273.1, SO:0001217, UniParc:UPI00
BlastRules:WP_009966224, EC:3.4.16.4, GO:0005576, GO:0005886, GO:0006508, GO:0008360, GO:0009002, GO:0009252, GO:0045121, GO:0071555, KEGG:K07258, RefSeq:WP_014112274.1, SO:0001217, UniParc:UPI0
BlastRules:WP_003247145, COG:COG0214, COG:H, EC:4.3.3.6, GO:0006520, GO:0008615, GO:0036381, GO:0042819, GO:0042823, KEGG:K06215, RefSeq:WP_003225444.1, SO:0001217, UniParc:UPI0001D2893D, UniF
BlastRules:WP_003226797, EC:3.5.1.2, EC:4.3.3.6, GO:0004359, GO:0005829, GO:0006543, GO:0008614, GO:0016884, GO:0036381, GO:0042802, GO:0042823, GO:1903600, RefSeq:WP_014112275.1, SO:0001217, UniP
RFAM:RF00230, SO:0000140
BlastRules:WP_003247131, COG:COG0172, COG:J, EC:6.1.1.11, GO:0000166, GO:0004828, GO:0005524, GO:0005737, GO:0006434, GO:0016260, GO:0016740, RefSeq:WP_061187062.1, SO:0001217, UniParc:UPI00077
SO:0000269
BlastRules:WP_003226792, EC:2.7.1.74, EC:2.7.1.76, GO:0004136, GO:0004137, GO:0005524, GO:0005737, GO:0019136, RefSeq:WP_013307891.1, SO:0001217, UniParc:UPI0001E14EA5, UniRef:UniRef100_A0A9Q4E6J
BlastRules:WP_003247138, EC:2.7.1.113, GO:0004138, GO:0005524, GO:0005737, GO:0019136, KEGG:K15518, RefSeq:WP_213408498.1, SO:0001217, UniParc:UPI001BA1DC94, UniRef:UniRef100_UPI001BA1DC94, Uni
EC:3.2.1.-, GO:0005975, GO:0008061, GO:0012505, GO:0016798, GO:0070492, SO:0001217, UniRef:UniRef50_P37531, UniRef:UniRef90_P37531
EC:3.-.-.-, GO:0016810, RefSeq:WP_061187059.1, SO:0001217, UniParc:UPI0006A925D8, UniRef:UniRef100_UPI0006A925D8, UniRef:UniRef50_P37532, UniRef:UniRef90_P37532
EC:3.5.4.33, GO:0002100, GO:0008251, GO:0008270, GO:0052717, KEGG:K11991, RefSeq:WP_014112280.1, SO:0001217, UniParc:UPI00022BAD2F, UniRef:UniRef100_G4NT14, UniRef:UniRef50_P21335, UniRef:UniRef9
RFAM:RF01854, SO:0000655
RFAM:RF00169, SO:0000655
BlastRules:WP_003247135, EC:2.7.7.7, GO:0003677, GO:0003887, GO:0005524, GO:0005737, GO:0006260, GO:0006261, GO:0009295, GO:0009360, GO:0016887, GO:0046872, KEGG:K02343, SO:0001217, UniRef:UniRe
GO:0003677, KEGG:K09747, RefSeq:WP_014112282.1, SO:0001217, UniParc:UPI00022BAEA4, UniRef:UniRef100_A0A9Q2LCM9, UniRef:UniRef50_Q2G0T4, UniRef:UniRef90_A0A9Q2LCM9
BlastRules:WP_000559171, BlastRules:WP_003225425, COG:COG0353, COG:L, GO:0003677, GO:0006281, GO:0006310, GO:0046872, KEGG:K06187, RefSeq:WP_003225425.1, SO:0001217, UniParc:UPI000005FDC5, Ur
GO:0016020, GO:0030435, GO:0046872, KEGG:K06317, SO:0001217, UniRef:UniRef50_P24282, UniRef:UniRef90_P24282
RFAM:RF01690, SO:0000655
GO:0003735, GO:0005840, KEGG:K01977, RFAM:RF00177, SO:0001000
SO:0000263
SO:0000254
GO:0003735, GO:0005840, KEGG:K01980, RFAM:RF02541, SO:0001001
GO:0003735, GO:0005840, KEGG:K01985, RFAM:RF00001, SO:0000652
BlastRules:WP_003243294, GO:0030435, GO:0046872, RefSeq:WP_014112284.1, SO:0001217, UniParc:UPI00022BAEA6, UniRef:UniRef100_G4NT92, UniRef:UniRef50_P37534, UniRef:UniRef90_P37534
SO:0001217, UniRef:UniRef50_P37467, UniRef:UniRef90_UPI000300CA13
RefSeq:WP_079288888.1, SO:0001217, UniParc:UPI0009A2EDEF, UniRef:UniRef100_UPI0009A2EDEF, UniRef:UniRef50_P37535, UniRef:UniRef90_P37535
GO:0016831, RefSeq:WP_327816544.1, SO:0001217, UniParc:UPI002E2009B0, UniRef:UniRef100_UPI002E2009B0, UniRef:UniRef50_P37536, UniRef:UniRef90_P37536
EC:2.7.4.9, GO:0004798, GO:0005524, GO:0005737, GO:0005829, GO:0006227, GO:0006233, GO:0006235, KEGG:K00943, RefSeq:WP_014112288.1, SO:0001217, UniParc:UPI00022BAEAA, UniRef:UniRef100_G4NT96, U
BlastRules:WP_003242755, GO:0005737, RefSeq:WP_003218295.1, SO:0001217, UniParc:UPI0001D28F95, UniRef:UniRef100_A0A9Q4HAG2, UniRef:UniRef50_P37538, UniRef:UniRef90_P37538
KEGG:K09770, RefSeq:WP_003218297.1, SO:0001217, UniParc:UPI0001D28F96, UniRef:UniRef100_G4NT98, UniRef:UniRef50_P37539, UniRef:UniRef90_P37539
BlastRules:WP_003244417, EC:2.7.7.7, GO:0003677, GO:0003887, GO:0005737, GO:0006260, GO:0006261, GO:0008408, GO:0009295, GO:0009360, SO:0001217, UniRef:UniRef50_P37540, UniRef:UniRef90_P37540
BlastRules:WP_003243571, COG:COG1774, COG:T, GO:0003729, GO:0005737, GO:0030435, RefSeq:WP_014112290.1, SO:0001217, UniParc:UPI00022BAEAC, UniRef:UniRef100_G4NTA0, UniRef:UniRef50_P37541, UniR
BlastRules:WP_003218308, GO:0006260, RefSeq:WP_003218308.1, SO:0001217, UniParc:UPI000005FDCF, UniRef:UniRef100_P37542, UniRef:UniRef50_P37542, UniRef:UniRef90_P37542
GO:0008168, RefSeq:WP_014112291.1, SO:0001217, UniParc:UPI00022BAEAD, UniRef:UniRef100_G4NTA2, UniRef:UniRef50_P37543, UniRef:UniRef90_P37543
SO:0001217, UniRef:UniRef50_Q81JB0
BlastRules:WP_003226760, COG:COG5845, COG:DK, GO:0003677, GO:0030435, GO:0042802, GO:0043937, GO:0045892, RefSeq:WP_003218316.1, SO:0001217, UniParc:UPI000016E7B5, UniRef:UniRef100_P08874, Un
SO:0001217, UniRef:UniRef50_Q7WSP6
SO:0001217, UniRef:UniRef50_A0A355S1N4
EC:3.1.-.-, GO:0004536, GO:0005829, GO:0046872, KEGG:K03424, RefSeq:WP_072565620.1, SO:0001217, UniParc:UPI00090941AC, UniRef:UniRef100_UPI00090941AC, UniRef:UniRef50_P37545, UniRef:UniRef90_P3754
GO:0004553, GO:0009254, GO:0019867, RefSeq:WP_240510062.1, SO:0001217, UniParc:UPI0006A93879, UniRef:UniRef100_UPI0006A93879, UniRef:UniRef50_P37546, UniRef:UniRef90_P37546
BlastRules:WP_003226752, EC:3.1.26.8, GO:0005737, GO:0006364, GO:0019843, GO:0042254, GO:0043822, GO:0046872, KEGG:K05985, RefSeq:WP_014112297.1, SO:0001217, UniParc:UPI00022BAEB3, UniRef:UniRef
EC:2.1.1.182, GO:0000179, GO:0003723, GO:0005829, GO:0006364, GO:0031167, GO:0052908, RefSeq:WP_014112298.1, SO:0001217, UniParc:UPI00022BAEB4, UniRef:UniRef100_G4NTB0, UniRef:UniRef50_P37468, Ur
BlastRules:WP_003243478, EC:3.4.-.-, GO:0008233, GO:0030436, KEGG:K06436, SO:0001217, UniRef:UniRef50_A0A5C0WFL3, UniRef:UniRef90_UPI00046348F4
EC:2.7.1.148, GO:0005524, GO:0016114, GO:0019288, GO:0050515, KEGG:K00919, RefSeq:WP_268360647.1, SO:0001217, UniParc:UPI0022822D24, UniRef:UniRef100_UPI0022822D24, UniRef:UniRef50_P37550, UniRef
GO:0003677, GO:0006355, GO:0009116, GO:0045892, GO:0045982, KEGG:K09685, RefSeq:WP_024713064.1, SO:0001217, UniParc:UPI000463BC3E, UniRef:UniRef100_A0A6H0WEL9, UniRef:UniRef50_P37551, UniRef:U
RefSeq:WP_003218344.1, SO:0001217, UniParc:UPI0001D28220, UniRef:UniRef100_A0A9Q4HJ46, UniRef:UniRef50_I3EC32, UniRef:UniRef90_A0AA96UNU6
COG:COG2088, COG:D, GO:0000917, GO:0030435, GO:0030436, KEGG:K06412, RefSeq:WP_003218346.1, SO:0001217, UniParc:UPI0001976D8A, UniRef:UniRef100_A0A9W5PB29, UniRef:UniRef50_Q81J99, UniRef:UniF
EC:2.3.1.157, EC:2.7.7.23, GO:0000287, GO:0000902, GO:0003977, GO:0005737, GO:0006048, GO:0008360, GO:0009245, GO:0009252, GO:0016020, GO:0019134, GO:0071555, KEGG:K04042, RefSeq:WP_053407025.1,
EC:2.7.6.1, GO:0000287, GO:0002189, GO:0004749, GO:0005524, GO:0005737, GO:0006015, GO:0006164, GO:0009156, GO:0016301, KEGG:K00948, RefSeq:WP_003218353.1, SO:0001217, UniParc:UPI000005FDE1, Ur
GO:0003735, GO:0005840, GO:0006412, GO:0008097, GO:0022625, RefSeq:WP_014112303.1, SO:0001217, UniParc:UPI00022BAEB9, UniRef:UniRef100_G4NTC0, UniRef:UniRef50_P14194, UniRef:UniRef90_P14194
EC:3.1.1.29, GO:0004045, GO:0005737, GO:0006515, GO:0030435, GO:0072344, RefSeq:WP_072183122.1, SO:0001217, UniParc:UPI0006A87F74, UniRef:UniRef100_UPI0006A87F74, UniRef:UniRef50_P37470, UniRef:Un
BlastRules:WP_003243417, GO:0005737, GO:0010468, GO:0030435, GO:0046872, RefSeq:WP_014112305.1, SO:0001217, UniParc:UPI00022BAEBB, UniRef:UniRef100_G4NTC2, UniRef:UniRef50_P37553, UniRef:UniRef9
EC:3.6.4.-, GO:0000716, GO:0003677, GO:0003684, GO:0004386, GO:0005524, GO:0005737, GO:0006281, GO:0006355, GO:0015616, GO:0016787, GO:0043175, SO:0001217, UniRef:UniRef50_P37474, UniRef:UniRef90_
SO:0001217, UniRef:UniRef50_W7UIH3, UniRef:UniRef90_A0A9D1PJB7
SO:0001217, UniRef:UniRef50_P37554
GO:0005886, SO:0001217, UniRef:UniRef50_P37555, UniRef:UniRef90_P37555
EC:3.6.1.9, GO:0006203, GO:0008168, GO:0046047, GO:0046052, GO:0046061, GO:0046076, GO:0046081, GO:0047429, KEGG:K02499, SO:0001217, UniRef:UniRef50_P37556, UniRef:UniRef90_P37556
RefSeq:WP_003218370.1, SO:0001217, UniParc:UPI0001D28229, UniRef:UniRef100_A0A9Q4HAF3, UniRef:UniRef50_P37557, UniRef:UniRef90_A0A150F7Z8
GO:0016020, GO:0030435, GO:0042601, RefSeq:WP_014112309.1, SO:0001217, UniParc:UPI00022BAEBF, UniRef:UniRef100_G4NTC8, UniRef:UniRef50_P37558, UniRef:UniRef90_P37558
GO:0016020, GO:0030435, GO:0034301, GO:0042601, RefSeq:WP_014112310.1, SO:0001217, UniParc:UPI00022BAEC0, UniRef:UniRef100_G4NTC9, UniRef:UniRef50_P37559, UniRef:UniRef90_P37559
BlastRules:WP_001208736, BlastRules:WP_003243211, GO:0051301, KEGG:K13052, RefSeq:WP_003242110.1, SO:0001217, UniParc:UPI00022BAEC1, UniRef:UniRef100_A0A9W5LEE7, UniRef:UniRef50_P37471, UniRef:U
GO:0003676, RefSeq:WP_003218379.1, SO:0001217, UniParc:UPI000005FDED, UniRef:UniRef100_P37560, UniRef:UniRef50_P37560, UniRef:UniRef90_P37560
SO:0000266
SO:0000259
EC:3.1.3.16, GO:0004722, GO:0005886, GO:0016020, GO:0017018, GO:0030435, GO:0030436, GO:0033192, GO:0042601, GO:0140791, GO:0180004, GO:0180005, GO:0180006, GO:0180007, GO:0180008, GO:1990439,
SO:0001217, UniRef:UniRef50_P37561, UniRef:UniRef90_P37561
BlastRules:WP_009966278, EC:2.7.11.1, GO:0004676, GO:0004677, GO:0004679, GO:0004711, GO:0005524, GO:0035175, GO:0035402, GO:0035403, GO:0035979, GO:0044022, GO:0044023, GO:0044024, GO:0044025,
BlastRules:WP_000655474, BlastRules:WP_003243704, EC:6.3.4.19, GO:0005524, GO:0005737, GO:0006400, GO:0032267, KEGG:K04075, SO:0001217, UniRef:UniRef50_P37563, UniRef:UniRef90_P37563
SO:0001217, UniRef:UniRef50_A0AA40IU15
BlastRules:WP_001079659, BlastRules:WP_002656600, BlastRules:WP_003243881, BlastRules:WP_012957092, COG:COG0465, COG:O, EC:3.4.24.-, GO:0004176, GO:0004222, GO:0005524, GO:0005886, GO:0006508, G
EC:2.7.1.33, GO:0004594, GO:0005524, GO:0005737, GO:0015937, GO:0046872, KEGG:K03525, RefSeq:WP_003218395.1, SO:0001217, UniParc:UPI0001D28233, UniRef:UniRef100_A0A9Q4DRH9, UniRef:UniRef50_P375
BlastRules:WP_000656366, BlastRules:WP_003226695, GO:0005737, GO:0006457, GO:0042026, GO:0044183, GO:0051082, KEGG:K04083, RefSeq:WP_041520828.1, SO:0001217, UniParc:UPI0003006E09, UniRef:UniRef
EC:5.2.1.8, GO:0140839, GO:0140840, RefSeq:WP_072181788.1, SO:0001217, UniParc:UPI00092F51F6, UniRef:UniRef100_UPI00092F51F6, UniRef:UniRef50_P37566, UniRef:UniRef90_P37566
EC:2.5.1.47, GO:0004122, GO:0004124, GO:0005737, GO:0006535, GO:0008284, GO:0009087, GO:0032991, GO:0032993, GO:0042803, GO:0080146, GO:0140297, GO:1904798, RefSeq:WP_014112318.1, SO:0001217, U
EC:2.6.1.85, GO:0000162, GO:0000287, GO:0046654, GO:0046656, GO:0046820, KEGG:K25578, SO:0001217, UniRef:UniRef50_P28820, UniRef:UniRef90_P28820
SO:0001217, UniRef:UniRef50_A0A920DJN6
EC:4.1.3.38, GO:0003824, RefSeq:WP_072181786.1, SO:0001217, UniParc:UPI0006A8A067, UniRef:UniRef100_UPI0006A8A067, UniRef:UniRef50_P28821, UniRef:UniRef90_E0TYK5
EC:2.5.1.15, GO:0004156, GO:0005829, GO:0009396, GO:0046654, GO:0046656, GO:0046872, KEGG:K00796, SO:0001217, UniRef:UniRef50_P28822, UniRef:UniRef90_P28822
PFAM:PF02152.23
PFAM:PF02152.23
EC:2.7.6.3, GO:0003848, GO:0005524, GO:0009396, GO:0016301, GO:0046654, GO:0046656, SO:0001217, UniRef:UniRef50_P29252, UniRef:UniRef90_P29252
EC:1.-.-.-, EC:1.3.1.-, GO:0008033, GO:0017150, GO:0050660, KEGG:K05540, SO:0001217, UniRef:UniRef50_A0A3M1RT76, UniRef:UniRef90_S7UC72
SO:0001217, UniRef:UniRef50_UPI002281E46F, UniRef:UniRef90_UPI002281E46F
COG:COG1190, COG:J, EC:6.1.1.6, GO:0000049, GO:0000287, GO:0004824, GO:0005524, GO:0005737, GO:0005829, GO:0006430, GO:0016740, SO:0001217, UniRef:UniRef50_P37477, UniRef:UniRef90_P37477
GO:0003735, GO:0005840, KEGG:K01977, RFAM:RF00177, SO:0001000
GO:0003735, GO:0005840, KEGG:K01980, RFAM:RF02541, SO:0001001
GO:0003735, GO:0005840, KEGG:K01985, RFAM:RF00001, SO:0000652
SO:0000273
SO:0000270
SO:0000265
SO:0000264
SO:0000260
SO:0000264
SO:0001036
SO:0000268
SO:0000254
GO:0003735, GO:0005840, KEGG:K01977, RFAM:RF00177, SO:0001000
GO:0003735, GO:0005840, KEGG:K01980, RFAM:RF02541, SO:0001001
GO:0003735, GO:0005840, KEGG:K01985, RFAM:RF00001, SO:0000652
BlastRules:WP_001244563, BlastRules:WP_003225724, BlastRules:WP_033017268, GO:0003677, GO:0006355, KEGG:K03708, RefSeq:WP_003225724.1, SO:0001217, UniParc:UPI000005FE00, UniRef:UniRef100_P37568,
SO:0001217, UniRef:UniRef50_UPI0034A064DA
EC:2.7.14.1, GO:0004111, GO:0004672, GO:0005524, GO:0005615, GO:0016301, GO:0046314, RefSeq:WP_061188087.1, SO:0001217, UniParc:UPI0006A83BD0, UniRef:UniRef100_UPI0006A83BD0, UniRef:UniRef50_P37
BlastRules:WP_003235011, COG:COG0542, COG:O, GO:0005524, GO:0016887, GO:0030420, SO:0001217, UniRef:UniRef50_Q2G0P5, UniRef:UniRef90_P37571
COG:COG1066, COG:L, EC:3.6.4.-, GO:0000725, GO:0003684, GO:0005524, GO:0006281, GO:0016887, GO:0046872, GO:0140664, SO:0001217, UniRef:UniRef50_P37572, UniRef:UniRef90_P37572
EC:2.7.7.85, GO:0000400, GO:0004016, GO:0005524, GO:0005737, GO:0006281, GO:0106408, KEGG:K07067, RefSeq:WP_014112331.1, SO:0001217, UniParc:UPI00022BB056, UniRef:UniRef100_G4NQC2, UniRef:UniRe
GO:0004518, GO:0046872, RefSeq:WP_014112332.1, SO:0001217, UniParc:UPI00022BB057, UniRef:UniRef100_G4NQC3, UniRef:UniRef50_Q06754, UniRef:UniRef90_Q06754
EC:2.7.7.60, GO:0019288, GO:0050518, SO:0001217, UniRef:UniRef50_Q06755, UniRef:UniRef90_Q06755
EC:4.6.1.12, GO:0008685, GO:0016114, GO:0019288, GO:0046872, RefSeq:WP_072182890.1, SO:0001217, UniParc:UPI0006A8482A, UniRef:UniRef100_A0A9Q4E4V5, UniRef:UniRef50_Q06756, UniRef:UniRef90_Q06756
EC:6.1.1.17, GO:0000049, GO:0000166, GO:0004818, GO:0005524, GO:0005829, GO:0006424, GO:0008270, KEGG:K09698, RefSeq:WP_061188090.1, SO:0001217, UniParc:UPI0006A8FA49, UniRef:UniRef100_A0A9Q4H
RFAM:RF00230, SO:0000140

EC:2.3.1.30, GO:0005737, GO:0005829, GO:0006535, GO:0009001, GO:0009087, GO:0019344, RefSeq:WP_014112338.1, SO:0001217, UniParc:UPI00022BB05D, UniRef:UniRef100_G4NQC9, UniRef:UniRef50_Q06750, Ur
EC:6.1.1.16, GO:0004817, GO:0005524, GO:0005737, GO:0005829, GO:0006423, GO:0008270, KEGG:K01883, SO:0001217, UniRef:UniRef50_Q06752, UniRef:UniRef90_Q06752
BlastRules:WP_004399685, EC:3.1.26.-, GO:0004525, GO:0004540, GO:0005737, GO:0006364, GO:0019843, RefSeq:WP_003242030.1, SO:0001217, UniParc:UPI00022BB05F, UniRef:UniRef100_A0A9W5PB55, UniRef:Uni
EC:2.1.1.-, GO:0003723, GO:0005829, GO:0006396, GO:0008173, GO:0032259, RefSeq:WP_003225755.1, SO:0001217, UniParc:UPI0001D28BA8, UniRef:UniRef100_A0A9Q4DS22, UniRef:UniRef50_Q06753, UniRef:UniRe
BlastRules:WP_003235032, KEGG:K06962, RefSeq:WP_003225756.1, SO:0001217, UniParc:UPI0001D28BA9, UniRef:UniRef100_A0A9Q4E3F2, UniRef:UniRef50_P37574, UniRef:UniRef90_P37574
COG:COG1595, COG:K, GO:0003677, GO:0003700, GO:0006352, GO:0006355, GO:0016987, GO:0030435, RefSeq:WP_003225758.1, SO:0001217, UniParc:UPI0001976C93, UniRef:UniRef100_A0A9Q4DUD1, UniRef:UniR
GO:0005886, GO:0006605, GO:0008320, GO:0009306, GO:0015450, GO:0043952, GO:0065002, RefSeq:WP_072182892.1, SO:0001217, UniParc:UPI0006A902D8, UniRef:UniRef100_UPI0006A902D8, UniRef:UniRef50_Q0
COG:COG0250, COG:K, GO:0005829, GO:0006353, GO:0031564, GO:0032784, GO:0140673, KEGG:K02601, RefSeq:WP_003225764.1, SO:0001217, UniParc:UPI000005FE11, UniRef:UniRef100_Q06795, UniRef:UniRef50_
COG:COG0080, COG:J, GO:0003735, GO:0005840, GO:0006412, GO:0022625, GO:0070180, KEGG:K02867, RefSeq:WP_003156430.1, SO:0001217, UniParc:UPI0001597116, UniRef:UniRef100_Q06796, UniRef:UniRef50_
GO:0000049, GO:0003723, GO:0003735, GO:0006412, GO:0006417, GO:0015934, GO:0019843, KEGG:K02863, RefSeq:WP_003235040.1, SO:0001217, UniParc:UPI000195C4DC, UniRef:UniRef100_Q06797, UniRef:UniRe
RFAM:RF00557, SO:0000140
COG:COG0244, COG:J, GO:0003735, GO:0006412, GO:0015934, GO:0070180, KEGG:K02864, RefSeq:WP_003242020.1, SO:0001217, UniParc:UPI0002AC3A1E, UniRef:UniRef100_A0A2M8SUW6, UniRef:UniRef50_P4292
COG:COG0222, COG:J, GO:0003729, GO:0003735, GO:0005840, GO:0006412, GO:0022625, RefSeq:WP_003156436.1, SO:0001217, UniParc:UPI000005FE15, UniRef:UniRef100_P02394, UniRef:UniRef50_P02394, UniRef
GO:0008757, GO:0032259, RefSeq:WP_072182893.1, SO:0001217, UniParc:UPI0006A92CCF, UniRef:UniRef100_UPI0006A92CCF, UniRef:UniRef50_P37872, UniRef:UniRef90_P37872
SO:0001217, UniRef:UniRef50_W4VLQ9
EC:2.7.7.6, GO:0003677, GO:0003899, GO:0006351, KEGG:K03043, SO:0001217, UniRef:UniRef50_W4VLQ9, UniRef:UniRef90_A0AAX3RPC9
COG:COG0086, COG:K, EC:2.7.7.6, GO:0000287, GO:0000428, GO:0001054, GO:0001055, GO:0001056, GO:0001057, GO:0001058, GO:0001066, GO:0003677, GO:0003899, GO:0006351, GO:0008270, KEGG:K03046, SO
GO:0003723, GO:0005737, GO:0005840, GO:1990904, KEGG:K07590, RefSeq:WP_003225778.1, SO:0001217, UniParc:UPI000005FE19, UniRef:UniRef100_P46350, UniRef:UniRef50_P46350, UniRef:UniRef90_P46350
COG:COG0048, COG:J, GO:0003735, GO:0006412, GO:0015935, KEGG:K02950, SO:0001217, UniParc:UPI00022D7AD3, UniRef:UniRef100_A0A060PNF2, UniRef:UniRef50_P0A7S3, UniRef:UniRef90_A0A0H4KEW2
COG:COG0049, COG:J, GO:0000049, GO:0003735, GO:0006412, GO:0015935, GO:0019843, KEGG:K02992, RefSeq:WP_003225784.1, SO:0001217, UniParc:UPI000195C4E4, UniRef:UniRef100_P21469, UniRef:UniRef50_
COG:COG0480, COG:J, GO:0003746, GO:0003924, GO:0005525, GO:0005737, GO:0006414, GO:0032790, KEGG:K02355, SO:0001217, UniRef:UniRef50_P80868, UniRef:UniRef90_P80868
SO:0001217, UniRef:UniRef50_A0A948GMB8
GO:0003746, GO:0003924, GO:0005525, GO:0005829, GO:0006414, SO:0001217, UniRef:UniRef50_A6GYU7, UniRef:UniRef90_A8F982
RefSeq:WP_072182895.1, SO:0001217, UniParc:UPI0006A80260, UniRef:UniRef100_UPI0006A80260, UniRef:UniRef50_P70981, UniRef:UniRef90_E0TZE6
BlastRules:WP_001040594, COG:COG0051, COG:J, GO:0000049, GO:0003723, GO:0003735, GO:0005840, GO:0006412, GO:1990904, RefSeq:WP_003156464.1, SO:0001217, UniParc:UPI000005FE1E, UniRef:UniRef100_
COG:COG0087, COG:J, GO:0003735, GO:0005840, GO:0006412, GO:0019843, GO:0022625, KEGG:K02906, RefSeq:WP_003225791.1, SO:0001217, UniParc:UPI0001D28C79, UniRef:UniRef100_A0A9Q4E2M4, UniRef:Uni
COG:COG0088, COG:J, GO:0003735, GO:0005840, GO:0006412, GO:0019843, GO:0022625, GO:1990904, RefSeq:WP_003225792.1, SO:0001217, UniParc:UPI0001D28C7A, UniRef:UniRef100_G4NQF2, UniRef:UniRef50_
GO:0003735, GO:0005840, GO:0006412, GO:0019843, GO:1990904, RefSeq:WP_003225793.1, SO:0001217, UniParc:UPI0001D28C7B, UniRef:UniRef100_A0A9Q4E3N9, UniRef:UniRef50_P42924, UniRef:UniRef90_P4292
COG:COG0090, COG:J, GO:0002181, GO:0003723, GO:0003735, GO:0006412, GO:0015934, GO:0016740, GO:0019843, KEGG:K02886, RefSeq:WP_003225795.1, SO:0001217, UniParc:UPI000195C4E7, UniRef:UniRef100
COG:COG0185, COG:J, GO:0000028, GO:0003735, GO:0005737, GO:0006412, GO:0015935, GO:0019843, RefSeq:WP_003156472.1, SO:0001217, UniParc:UPI000005FE23, UniRef:UniRef100_P21476, UniRef:UniRef50_P
COG:COG0091, COG:J, GO:0003735, GO:0006412, GO:0015934, GO:0019843, GO:0022625, RefSeq:WP_003156475.1, SO:0001217, UniParc:UPI000005FE24, UniRef:UniRef100_P42060, UniRef:UniRef50_P42060, UniRef
COG:COG0092, COG:J, GO:0003729, GO:0003735, GO:0006412, GO:0019843, GO:0022627, KEGG:K02982, RefSeq:WP_003242001.1, SO:0001217, UniParc:UPI00022BAAD6, UniRef:UniRef100_A0A3N6BZP3, UniRef:Uni
COG:COG0197, COG:J, GO:0000049, GO:0003735, GO:0006412, GO:0019843, GO:0022625, KEGG:K02878, RefSeq:WP_003225801.1, SO:0001217, UniParc:UPI000005FE26, UniRef:UniRef100_P14577, UniRef:UniRef50_
COG:COG0255, COG:J, GO:0003735, GO:0005840, GO:0006412, GO:0022625, RefSeq:WP_003156482.1, SO:0001217, UniParc:UPI000005FE27, UniRef:UniRef100_P12873, UniRef:UniRef50_P12873, UniRef:UniRef90_P1
GO:0003735, GO:0005840, GO:0006412, GO:0019843, GO:0022627, RefSeq:WP_003225804.1, SO:0001217, UniParc:UPI000005FE28, UniRef:UniRef100_P12874, UniRef:UniRef50_P12874, UniRef:UniRef90_P12874
COG:COG0093, COG:J, GO:0003735, GO:0006412, GO:0015934, GO:0022625, GO:0070180, KEGG:K02874, RefSeq:WP_003225805.1, SO:0001217, UniParc:UPI000005FE29, UniRef:UniRef100_P12875, UniRef:UniRef50_
COG:COG0198, COG:J, GO:0003677, GO:0003735, GO:0005840, GO:0006412, GO:0009295, GO:0019843, GO:0022625, RefSeq:WP_003156486.1, SO:0001217, UniParc:UPI000005FE2A, UniRef:UniRef100_P0CI78, UniRe
COG:COG0094, COG:J, GO:0000049, GO:0003735, GO:0005840, GO:0006412, GO:0019843, GO:1990904, KEGG:K02931, RefSeq:WP_003225809.1, SO:0001217, UniParc:UPI000005FE2B, UniRef:UniRef100_P12877, Uni
BlastRules:WP_003156488, COG:COG0199, COG:J, GO:0003735, GO:0005840, GO:0006412, GO:0008270, GO:0015935, GO:0019843, KEGG:K02954, RefSeq:WP_003156488.1, SO:0001217, UniParc:UPI000005FE2C, Un
COG:COG0096, COG:J, GO:0003735, GO:0005737, GO:0005840, GO:0006412, GO:0019843, GO:1990904, RefSeq:WP_003241998.1, SO:0001217, UniParc:UPI000016E944, UniRef:UniRef100_P12879, UniRef:UniRef50_P
COG:COG0097, COG:J, GO:0002181, GO:0003735, GO:0005840, GO:0006412, GO:0019843, GO:0022625, RefSeq:WP_003225814.1, SO:0001217, UniParc:UPI0001D28C80, UniRef:UniRef100_A0A9Q4E2L4, UniRef:UniRe
GO:0003735, GO:0005840, GO:0006412, GO:0008097, GO:0022625, RefSeq:WP_014112347.1, SO:0001217, UniParc:UPI00022BB20B, UniRef:UniRef100_G4NQG6, UniRef:UniRef50_P46899, UniRef:UniRef90_P46899
COG:COG0098, COG:J, GO:0003735, GO:0005737, GO:0006412, GO:0015935, GO:0019843, KEGG:K02988, SO:0001217, UniParc:UPI00015970A4, UniRef:UniRef100_A7Z0Q5, UniRef:UniRef50_P21467, UniRef:UniRef90_
COG:COG1841, COG:J, GO:0003735, GO:0006412, GO:0015934, GO:0022625, RefSeq:WP_003156497.1, SO:0001217, UniParc:UPI000005FE31, UniRef:UniRef100_P19947, UniRef:UniRef50_Q5SHQ6, UniRef:UniRef90_P
COG:COG0200, COG:J, GO:0003735, GO:0006412, GO:0015934, GO:0019843, GO:0022625, KEGG:K02876, RefSeq:WP_003225819.1, SO:0001217, UniParc:UPI000005FE32, UniRef:UniRef100_P19946, UniRef:UniRef50_
GO:0005048, GO:0005886, GO:0006616, GO:0008320, GO:0015031, GO:0016020, GO:0031522, GO:0043952, GO:0045121, RefSeq:WP_014112348.1, SO:0001217, UniParc:UPI00022BB20C, UniRef:UniRef100_G4NQH0,
EC:2.7.4.3, GO:0004017, GO:0005524, GO:0005737, GO:0006139, GO:0008270, GO:0016776, GO:0019205, GO:0044209, RefSeq:WP_003225823.1, SO:0001217, UniParc:UPI0001D28C83, UniRef:UniRef100_G4NQH1, Un
COG:COG0024, COG:J, EC:3.4.11.18, GO:0004239, GO:0005829, GO:0006508, GO:0046872, GO:0070006, KEGG:K01265, RefSeq:WP_003225828.1, SO:0001217, UniParc:UPI000005FE35, UniRef:UniRef100_P19994, Uni
COG:COG0361, COG:J, GO:0003743, GO:0005829, GO:0006413, GO:0019843, GO:0043022, KEGG:K02518, RefSeq:WP_003156508.1, SO:0001217, UniParc:UPI000005FE36, UniRef:UniRef100_P20458, UniRef:UniRef50_
BlastRules:WP_000868344, COG:COG0257, COG:J, GO:0003735, GO:0005737, GO:0005840, GO:0006412, GO:1990904, KEGG:K02919, RefSeq:WP_003156543.1, SO:0001217, UniParc:UPI000005FE37, UniRef:UniRef100
COG:COG0099, COG:J, GO:0000049, GO:0003723, GO:0003735, GO:0005829, GO:0005840, GO:0006412, GO:0015935, GO:0019843, KEGG:K02952, RefSeq:WP_003235095.1, SO:0001217, UniParc:UPI000016E945, Uni
COG:COG0100, COG:J, GO:0003735, GO:0005840, GO:0006412, GO:0019843, GO:1990904, KEGG:K02948, RefSeq:WP_009966385.1, SO:0001217, UniParc:UPI000005FE39, UniRef:UniRef100_P04969, UniRef:UniRef50_
COG:COG0202, COG:K, EC:2.7.7.6, GO:0000428, GO:0001054, GO:0001055, GO:0001056, GO:0001057, GO:0001058, GO:0001066, GO:0003677, GO:0003899, GO:0005737, GO:0006351, GO:0046983, RefSeq:WP_00322
COG:COG0203, COG:J, GO:0003735, GO:0005840, GO:0006412, GO:0022625, KEGG:K02879, RefSeq:WP_003225836.1, SO:0001217, UniParc:UPI000005FE3B, UniRef:UniRef100_P20277, UniRef:UniRef50_P20277, UniR
EC:7.-.-.-, GO:0005524, GO:0016020, GO:0016887, GO:0042626, GO:0043190, GO:0055085, RefSeq:WP_014112350.1, SO:0001217, UniParc:UPI00022BB20E, UniRef:UniRef100_G4NQH8, UniRef:UniRef50_A2RI01, UniRe
EC:7.-.-.-, GO:0005524, GO:0016020, GO:0016887, GO:0042626, GO:0043190, GO:0055085, SO:0001217, UniRef:UniRef50_A2RI02, UniRef:UniRef90_P70970
GO:0005886, GO:0022857, KEGG:K16785, RefSeq:WP_014112351.1, SO:0001217, UniParc:UPI00022BB20F, UniRef:UniRef100_G4NQH9, UniRef:UniRef50_P70972, UniRef:UniRef90_P70972
EC:5.4.99.12, GO:0001522, GO:0003723, GO:0009451, GO:0009982, GO:0031119, GO:0160147, KEGG:K06173, RefSeq:WP_061188096.1, SO:0001217, UniParc:UPI0007722B71, UniRef:UniRef100_UPI0007722B71, UniRe
RFAM:RF00555, SO:0000140
COG:COG0102, COG:J, GO:0003729, GO:0003735, GO:0005840, GO:0006412, GO:0017148, GO:0022625, KEGG:K02871, RefSeq:WP_003241966.1, SO:0001217, UniParc:UPI000195C4F9, UniRef:UniRef100_P70974, Uni
COG:COG0103, COG:J, GO:0003723, GO:0003735, GO:0005840, GO:0006412, GO:0022627, KEGG:K02996, RefSeq:WP_004399691.1, SO:0001217, UniParc:UPI000005FE41, UniRef:UniRef100_P21470, UniRef:UniRef50_
GO:0008168, GO:0008757, GO:0032259, RefSeq:WP_014112354.1, SO:0001217, UniParc:UPI00022BB212, UniRef:UniRef100_G4NQI4, UniRef:UniRef50_P70976, UniRef:UniRef90_P70976
SO:0001217, UniRef:UniRef50_P50862, UniRef:UniRef90_P50862
EC:3.5.1.28, GO:0005576, GO:0008745, GO:0009253, GO:0030436, GO:0071555, KEGG:K01448, SO:0001217, UniRef:UniRef50_P50864, UniRef:UniRef90_P50864
BlastRules:WP_003235124, GO:0005524, GO:0016226, GO:0016887, GO:0045892, GO:0046872, GO:0051539, GO:0140663, RefSeq:WP_061188098.1, SO:0001217, UniParc:UPI0007721E16, UniRef:UniRef100_UPI000772
BlastRules:WP_003235126, GO:0005886, GO:0009847, GO:0031160, KEGG:K06294, RefSeq:WP_014112358.1, SO:0001217, UniParc:UPI00022BB216, UniRef:UniRef100_G4NQI8, UniRef:UniRef50_P16450, UniRef:UniRef
GO:0005886, GO:0030435, GO:0045881, KEGG:K06349, SO:0001217, UniRef:UniRef50_P16449, UniRef:UniRef90_P16449
SO:0001217, UniRef:UniRef50_UPI002E1FF5A4, UniRef:UniRef90_UPI0033994F16
GO:0003735, GO:0005840, KEGG:K01977, RFAM:RF00177, SO:0001000
GO:0003735, GO:0005840, KEGG:K01980, RFAM:RF02541, SO:0001001
GO:0003735, GO:0005840, KEGG:K01985, RFAM:RF00001, SO:0000652
SO:0000257
SO:0000270
RefSeq:WP_014112362.1, SO:0001217, UniParc:UPI00022BB381, UniRef:UniRef100_G4NQJ2, UniRef:UniRef50_G4NQJ2, UniRef:UniRef90_G4NQJ2
RefSeq:WP_014112363.1, SO:0001217, UniParc:UPI00022BB382, UniRef:UniRef100_G4NQJ3, UniRef:UniRef50_G4NQJ3, UniRef:UniRef90_G4NQJ3
RefSeq:WP_014112364.1, SO:0001217, UniParc:UPI00022BB383, UniRef:UniRef100_G4NT20, UniRef:UniRef50_G4NT20, UniRef:UniRef90_G4NT20
RefSeq:WP_017696738.1, SO:0001217, UniParc:UPI00037CCC5F, UniRef:UniRef100_UPI00037CCC5F, UniRef:UniRef50_A0AAP5QTI9, UniRef:UniRef90_UPI0029552E9D
RefSeq:WP_014112366.1, SO:0001217, UniParc:UPI00022BB385, UniRef:UniRef100_G4NT91, UniRef:UniRef50_G4NT91, UniRef:UniRef90_G4NT91
RefSeq:WP_017696740.1, SO:0001217, UniParc:UPI00036EF6FE, UniRef:UniRef100_UPI00036EF6FE, UniRef:UniRef50_A0AAP5QRY4, UniRef:UniRef90_UPI00345BBDA9
RefSeq:WP_014112367.1, SO:0001217, UniParc:UPI00022BB386, UniRef:UniRef100_G4NT23, UniRef:UniRef50_G4NT23, UniRef:UniRef90_G4NT23
RefSeq:WP_014112368.1, SO:0001217, UniParc:UPI00022BB387, UniRef:UniRef100_G4NT24, UniRef:UniRef50_G4NT24, UniRef:UniRef90_G4NT24
RefSeq:WP_014112369.1, SO:0001217, UniParc:UPI00022BB388, UniRef:UniRef100_G4NT25, UniRef:UniRef50_G4NT25, UniRef:UniRef90_G4NT25
RefSeq:WP_041520831.1, SO:0001217, UniParc:UPI0005A1A432, UniRef:UniRef100_UPI0005A1A432, UniRef:UniRef50_A0A6I7FB31, UniRef:UniRef90_A0A8I1WIJ9
RefSeq:WP_014112370.1, SO:0001217, UniParc:UPI00022BB389, UniRef:UniRef100_G4NTF3, UniRef:UniRef50_A0AAP5V2F0, UniRef:UniRef90_G4NTF3
GO:0006310, RefSeq:WP_014112371.1, SO:0001217, UniParc:UPI00022BB38A, UniRef:UniRef100_G4NTF4, UniRef:UniRef50_A0A5R9C6P6, UniRef:UniRef90_G4NTF4
RefSeq:WP_041520832.1, SO:0001217, UniParc:UPI0005A22F93, UniRef:UniRef100_G4NT28, UniRef:UniRef50_G4HNS2, UniRef:UniRef90_G4NT28
SO:0001217, UniRef:UniRef50_A0A2G8IY16
RefSeq:WP_014112373.1, SO:0001217, UniParc:UPI00022BB38C, UniRef:UniRef100_G4NTF6, UniRef:UniRef50_G4NTF6, UniRef:UniRef90_G4NTF6
RefSeq:WP_374188608.1, SO:0001217, UniParc:UPI000ACEA172, UniRef:UniRef100_UPI000ACEA172, UniRef:UniRef50_G4NTF7, UniRef:UniRef90_G4NTF7
EC:5.6.2.3, GO:0003677, GO:0003678, GO:0005524, GO:0006260, KEGG:K02314, RefSeq:WP_014112375.1, SO:0001217, UniParc:UPI00022BB38E, UniRef:UniRef100_G4NTF8, UniRef:UniRef50_A0A5M8RPB5, UniRef:Un
SO:0001217, UniRef:UniRef50_A0A1R1QPW4, UniRef:UniRef90_UPI0030D9287C
EC:3.1.21.10, KEGG:K01160, RefSeq:WP_014112376.1, SO:0001217, UniParc:UPI00022BB38F, UniRef:UniRef100_G4NT32, UniRef:UniRef50_G4NT32, UniRef:UniRef90_G4NT32
RefSeq:WP_041520834.1, SO:0001217, UniParc:UPI0005A16297, UniRef:UniRef100_UPI0005A16297, UniRef:UniRef50_G4NTG0, UniRef:UniRef90_G4NTG0
RefSeq:WP_014112378.1, SO:0001217, UniParc:UPI00022BB391, UniRef:UniRef100_G4NTG1, UniRef:UniRef50_A0A8I1WJP5, UniRef:UniRef90_A0A8I1WJP5
RefSeq:WP_014112379.1, SO:0001217, UniParc:UPI00022BB392, UniRef:UniRef100_G4NTG2, UniRef:UniRef50_A0AAP5V1L8, UniRef:UniRef90_G4NTG2
RefSeq:WP_014112380.1, SO:0001217, UniParc:UPI00022BB393, UniRef:UniRef100_G4NT36, UniRef:UniRef50_G4NT36, UniRef:UniRef90_G4NT36
RefSeq:WP_014112381.1, SO:0001217, UniParc:UPI00022BB394, UniRef:UniRef100_G4NT37, UniRef:UniRef50_G4NT37, UniRef:UniRef90_G4NT37
RefSeq:WP_014112382.1, SO:0001217, UniParc:UPI00022BB395, UniRef:UniRef100_G4NT38, UniRef:UniRef50_A0A0M0GA20, UniRef:UniRef90_G4NT38
RefSeq:WP_014112383.1, SO:0001217, UniParc:UPI00022BB396, UniRef:UniRef100_G4NTG6, UniRef:UniRef50_A0A378Y054, UniRef:UniRef90_G4NTG6
RefSeq:WP_014112384.1, SO:0001217, UniParc:UPI00022BB397, UniRef:UniRef100_G4NT40, UniRef:UniRef50_G4NT40, UniRef:UniRef90_G4NT40
SO:0001217, UniRef:UniRef50_G4NTG8, UniRef:UniRef90_G4NTG8
RefSeq:WP_044059163.1, SO:0001217, UniParc:UPI0005C53350, UniRef:UniRef100_UPI0005C53350, UniRef:UniRef50_UPI000F546693, UniRef:UniRef90_UPI0005C53350
RefSeq:WP_014112388.1, SO:0001217, UniParc:UPI00022BB39B, UniRef:UniRef100_G4NT44, UniRef:UniRef50_A0A653VNJ1, UniRef:UniRef90_G4NT44
RefSeq:WP_014112389.1, SO:0001217, UniParc:UPI00022BB39C, UniRef:UniRef100_G4NT45, UniRef:UniRef50_Q38579, UniRef:UniRef90_G4NT45
RefSeq:WP_014112390.1, SO:0001217, UniParc:UPI00022BB39D, UniRef:UniRef100_G4NTH3, UniRef:UniRef50_A0A150F498, UniRef:UniRef90_G4NTH3
RefSeq:WP_014112391.1, SO:0001217, UniParc:UPI00022BB39E, UniRef:UniRef100_G4NTH4, UniRef:UniRef50_M8DL86, UniRef:UniRef90_G4NTH4
EC:2.1.1.37, KEGG:K00558, RefSeq:WP_014112392.1, SO:0001217, UniParc:UPI00022BB39F, UniRef:UniRef100_G4NTH5, UniRef:UniRef50_G4NTH5, UniRef:UniRef90_G4NTH5
SO:0001217, UniParc:UPI0002D332B1, UniRef:UniRef100_A0A8I2B7B8, UniRef:UniRef50_Q65GH4, UniRef:UniRef90_G4NT49
RefSeq:WP_019712754.1, SO:0001217, UniParc:UPI0003112168, UniRef:UniRef100_UPI0003112168, UniRef:UniRef50_A0A2S0JZZ8, UniRef:UniRef90_A0A8I1WGL3
RefSeq:WP_014112394.1, SO:0001217, UniParc:UPI00022BB3A1, UniRef:UniRef100_G4NTH7, UniRef:UniRef50_G4NTH7, UniRef:UniRef90_G4NTH7
KEGG:K06909, RefSeq:WP_014112395.1, SO:0001217, UniParc:UPI00022BB3A2, UniRef:UniRef100_G4NTH8, UniRef:UniRef50_P39786, UniRef:UniRef90_G4NTH8
RefSeq:WP_014112396.1, SO:0001217, UniParc:UPI00022BB3A3, UniRef:UniRef100_G4NT52, UniRef:UniRef50_P45917, UniRef:UniRef90_G4NT52
RefSeq:WP_014112397.1, SO:0001217, UniParc:UPI00022BB3A4, UniRef:UniRef100_G4NT53, UniRef:UniRef50_P45918, UniRef:UniRef90_G4NT53
RefSeq:WP_014112398.1, SO:0001217, UniParc:UPI00022BB3A5, UniRef:UniRef100_G4NTI1, UniRef:UniRef50_G4NTI1, UniRef:UniRef90_G4NTI1
RefSeq:WP_014112399.1, SO:0001217, UniParc:UPI00022BB3A6, UniRef:UniRef100_G4NTI2, UniRef:UniRef50_A0A4R6TUE8, UniRef:UniRef90_G4NTI2
RefSeq:WP_014112400.1, SO:0001217, UniParc:UPI00022BB3A7, UniRef:UniRef100_G4NT56, UniRef:UniRef50_P54327, UniRef:UniRef90_G4NT56
RefSeq:WP_010330132.1, SO:0001217, UniParc:UPI00022BB3A8, UniRef:UniRef100_G4NT57, UniRef:UniRef50_P45922, UniRef:UniRef90_G4NT57
RefSeq:WP_010330131.1, SO:0001217, UniParc:UPI00022BB3A9, UniRef:UniRef100_G4NT58, UniRef:UniRef50_P45923, UniRef:UniRef90_G4NT58
RefSeq:WP_014112401.1, SO:0001217, UniParc:UPI00022BB3AA, UniRef:UniRef100_G4NT59, UniRef:UniRef50_P54328, UniRef:UniRef90_G4NT59
RefSeq:WP_014112402.1, SO:0001217, UniParc:UPI00022BB3AB, UniRef:UniRef100_G4NTI7, UniRef:UniRef50_P45925, UniRef:UniRef90_P45925
RefSeq:WP_014112403.1, SO:0001217, UniParc:UPI00022BB3AC, UniRef:UniRef100_G4NT61, UniRef:UniRef50_P45926, UniRef:UniRef90_G4NT61
RefSeq:WP_080010045.1, SO:0001217, UniParc:UPI0002E14426, UniRef:UniRef100_A0A8I2BAQ1, UniRef:UniRef50_A0A8I2BAQ1, UniRef:UniRef90_A0A8I2BAQ1
RefSeq:WP_014112405.1, SO:0001217, UniParc:UPI00022BB3AE, UniRef:UniRef100_G4NTJ0, UniRef:UniRef50_P45927, UniRef:UniRef90_P45927
RefSeq:WP_014112406.1, SO:0001217, UniParc:UPI00022BB3AF, UniRef:UniRef100_G4NT64, UniRef:UniRef50_P54332, UniRef:UniRef90_P54332
RFAM:RF01412, SO:0000655
RefSeq:WP_014112407.1, SO:0001217, UniParc:UPI00022BB3B0, UniRef:UniRef100_G4NT65, UniRef:UniRef50_P54333, UniRef:UniRef90_P45930
SO:0001217, UniRef:UniRef50_C0H3Z8, UniRef:UniRef90_A0A0D1KVX4
RefSeq:WP_014112409.1, SO:0001217, UniParc:UPI00022BB524, UniRef:UniRef100_G4NTJ4, UniRef:UniRef50_G4NTJ4, UniRef:UniRef90_G4NTJ4
RefSeq:WP_014112410.1, SO:0001217, UniParc:UPI00022BB525, UniRef:UniRef100_G4NTJ5, UniRef:UniRef50_P54335, UniRef:UniRef90_P45932
RefSeq:WP_014112411.1, SO:0001217, UniParc:UPI00022BB526, UniRef:UniRef100_G4NTJ6, UniRef:UniRef50_P54336, UniRef:UniRef90_P45950
RefSeq:WP_014112412.1, SO:0001217, UniParc:UPI00022BB527, UniRef:UniRef100_G4NTJ7, UniRef:UniRef50_P45933, UniRef:UniRef90_G4NTJ7
RefSeq:WP_014112413.1, SO:0001217, UniParc:UPI00022BB528, UniRef:UniRef100_G4NT71, UniRef:UniRef50_P45934, UniRef:UniRef90_P45934
RefSeq:WP_014112414.1, SO:0001217, UniParc:UPI00022BB529, UniRef:UniRef100_G4NTJ9, UniRef:UniRef50_P54339, UniRef:UniRef90_P45935
RefSeq:WP_014112415.1, SO:0001217, UniParc:UPI00022BB52A, UniRef:UniRef100_G4NT73, UniRef:UniRef50_P45936, UniRef:UniRef90_P45936
RefSeq:WP_014112416.1, SO:0001217, UniParc:UPI00022BB52B, UniRef:UniRef100_G4NT74, UniRef:UniRef50_P45937, UniRef:UniRef90_P45937
RefSeq:WP_014112417.1, SO:0001217, UniParc:UPI00022BB52C, UniRef:UniRef100_G4NTK2, UniRef:UniRef50_G4NTK2, UniRef:UniRef90_G4NTK2
RefSeq:WP_014112418.1, SO:0001217, UniParc:UPI00022BB52D, UniRef:UniRef100_G4NTK3, UniRef:UniRef50_P54342, UniRef:UniRef90_G4NTK3

RefSeq:WP_010330111.1, SO:0001217, UniParc:UPI00022BB52E, UniRef:UniRef100_G4NT77, UniRef:UniRef50_P54343, UniRef:UniRef90_P45940
RefSeq:WP_014112419.1, SO:0001217, UniParc:UPI00022BB52F, UniRef:UniRef100_G4NT78, UniRef:UniRef50_P39797, UniRef:UniRef90_G4NT78
RefSeq:WP_041520837.1, SO:0001217, UniParc:UPI00059F6ABE, UniRef:UniRef100_UPI00059F6ABE, UniRef:UniRef50_Q9ZXD8, UniRef:UniRef90_A0A9W5LK67
RefSeq:WP_014112421.1, SO:0001217, UniParc:UPI00022BB531, UniRef:UniRef100_G4NTK7, UniRef:UniRef50_P24808, UniRef:UniRef90_G4NTK7
RefSeq:WP_014112422.1, SO:0001217, UniParc:UPI00022BB532, UniRef:UniRef100_G4NTK8, UniRef:UniRef50_A0A0D1X892, UniRef:UniRef90_G4NTK8
EC:3.1.-.-, KEGG:K21491, RefSeq:WP_014112423.1, SO:0001217, UniParc:UPI00022BB533, UniRef:UniRef100_G4NT82, UniRef:UniRef50_P45942, UniRef:UniRef90_G4NT82
EC:3.1.-.-, KEGG:K06366, SO:0001217, UniParc:UPI00022BB534, UniRef:UniRef100_G4NT83, UniRef:UniRef50_Q59HN8, UniRef:UniRef90_G4NT83
RefSeq:WP_014112425.1, SO:0001217, UniParc:UPI00022BB535, UniRef:UniRef100_G4NTL1, UniRef:UniRef50_A0A0D1L3M0, UniRef:UniRef90_A0A0D1L3M0
RefSeq:WP_014112426.1, SO:0001217, UniParc:UPI00022BB536, UniRef:UniRef100_G4NT85, UniRef:UniRef50_G4NT85, UniRef:UniRef90_G4NT85
RefSeq:WP_014112427.1, SO:0001217, UniParc:UPI00022BB537, UniRef:UniRef100_G4NT86, UniRef:UniRef50_A0A0M9WIP5, UniRef:UniRef90_G4NT86
SO:0000260
SO:0001036
SO:0000268
SO:0000254
GO:0003735, GO:0005840, KEGG:K01977, RFAM:RF00177, SO:0001000
GO:0003735, GO:0005840, KEGG:K01980, RFAM:RF02541, SO:0001001
GO:0003735, GO:0005840, KEGG:K01985, RFAM:RF00001, SO:0000652
SO:0000257
SO:0000270
RefSeq:WP_014112362.1, SO:0001217, UniParc:UPI00022BB381, UniRef:UniRef100_G4NQJ2, UniRef:UniRef50_G4NQJ2, UniRef:UniRef90_G4NQJ2
RefSeq:WP_014112363.1, SO:0001217, UniParc:UPI00022BB382, UniRef:UniRef100_G4NQJ3, UniRef:UniRef50_G4NQJ3, UniRef:UniRef90_G4NQJ3
RefSeq:WP_014112364.1, SO:0001217, UniParc:UPI00022BB383, UniRef:UniRef100_G4NT20, UniRef:UniRef50_G4NT20, UniRef:UniRef90_G4NT20
RefSeq:WP_017696738.1, SO:0001217, UniParc:UPI00037CCC5F, UniRef:UniRef100_UPI00037CCC5F, UniRef:UniRef50_A0AAP5QTI9, UniRef:UniRef90_UPI0029552E9D
RefSeq:WP_014112366.1, SO:0001217, UniParc:UPI00022BB385, UniRef:UniRef100_G4NT91, UniRef:UniRef50_G4NT91, UniRef:UniRef90_G4NT91
RefSeq:WP_017696740.1, SO:0001217, UniParc:UPI00036EF6FE, UniRef:UniRef100_UPI00036EF6FE, UniRef:UniRef50_A0AAP5QRY4, UniRef:UniRef90_UPI00345BBDA9
RefSeq:WP_014112367.1, SO:0001217, UniParc:UPI00022BB386, UniRef:UniRef100_G4NT23, UniRef:UniRef50_G4NT23, UniRef:UniRef90_G4NT23
RefSeq:WP_014112368.1, SO:0001217, UniParc:UPI00022BB387, UniRef:UniRef100_G4NT24, UniRef:UniRef50_G4NT24, UniRef:UniRef90_G4NT24
RefSeq:WP_014112369.1, SO:0001217, UniParc:UPI00022BB388, UniRef:UniRef100_G4NT25, UniRef:UniRef50_G4NT25, UniRef:UniRef90_G4NT25
RefSeq:WP_041520831.1, SO:0001217, UniParc:UPI0005A1A432, UniRef:UniRef100_UPI0005A1A432, UniRef:UniRef50_A0A6I7FB31, UniRef:UniRef90_A0A8I1WIJ9
RefSeq:WP_014112370.1, SO:0001217, UniParc:UPI00022BB389, UniRef:UniRef100_G4NTF3, UniRef:UniRef50_A0AAP5V2F0, UniRef:UniRef90_G4NTF3
GO:0006310, RefSeq:WP_014112371.1, SO:0001217, UniParc:UPI00022BB38A, UniRef:UniRef100_G4NTF4, UniRef:UniRef50_A0A5R9C6P6, UniRef:UniRef90_G4NTF4
RefSeq:WP_041520832.1, SO:0001217, UniParc:UPI0005A22F93, UniRef:UniRef100_G4NT28, UniRef:UniRef50_G4HNS2, UniRef:UniRef90_G4NT28
SO:0001217, UniRef:UniRef50_A0A2G8IY16
RefSeq:WP_014112373.1, SO:0001217, UniParc:UPI00022BB38C, UniRef:UniRef100_G4NTF6, UniRef:UniRef50_G4NTF6, UniRef:UniRef90_G4NTF6
RefSeq:WP_374188608.1, SO:0001217, UniParc:UPI000ACEA172, UniRef:UniRef100_UPI000ACEA172, UniRef:UniRef50_G4NTF7, UniRef:UniRef90_G4NTF7
EC:5.6.2.3, GO:0003677, GO:0003678, GO:0005524, GO:0006260, KEGG:K02314, RefSeq:WP_014112375.1, SO:0001217, UniParc:UPI00022BB38E, UniRef:UniRef100_G4NTF8, UniRef:UniRef50_A0A5M8RPB5, UniRef:Un
SO:0001217, UniRef:UniRef50_A0A1R1QPW4, UniRef:UniRef90_UPI0030D9287C
EC:3.1.21.10, KEGG:K01160, RefSeq:WP_014112376.1, SO:0001217, UniParc:UPI00022BB38F, UniRef:UniRef100_G4NT32, UniRef:UniRef50_G4NT32, UniRef:UniRef90_G4NT32
RefSeq:WP_041520834.1, SO:0001217, UniParc:UPI0005A16297, UniRef:UniRef100_UPI0005A16297, UniRef:UniRef50_G4NTG0, UniRef:UniRef90_G4NTG0
RefSeq:WP_014112378.1, SO:0001217, UniParc:UPI00022BB391, UniRef:UniRef100_G4NTG1, UniRef:UniRef50_A0A8I1WJP5, UniRef:UniRef90_A0A8I1WJP5
RefSeq:WP_014112379.1, SO:0001217, UniParc:UPI00022BB392, UniRef:UniRef100_G4NTG2, UniRef:UniRef50_A0AAP5V1L8, UniRef:UniRef90_G4NTG2
RefSeq:WP_014112380.1, SO:0001217, UniParc:UPI00022BB393, UniRef:UniRef100_G4NT36, UniRef:UniRef50_G4NT36, UniRef:UniRef90_G4NT36
RefSeq:WP_014112381.1, SO:0001217, UniParc:UPI00022BB394, UniRef:UniRef100_G4NT37, UniRef:UniRef50_G4NT37, UniRef:UniRef90_G4NT37
RefSeq:WP_014112382.1, SO:0001217, UniParc:UPI00022BB395, UniRef:UniRef100_G4NT38, UniRef:UniRef50_A0A0M0GA20, UniRef:UniRef90_G4NT38
RefSeq:WP_014112383.1, SO:0001217, UniParc:UPI00022BB396, UniRef:UniRef100_G4NTG6, UniRef:UniRef50_A0A378Y054, UniRef:UniRef90_G4NTG6
RefSeq:WP_014112384.1, SO:0001217, UniParc:UPI00022BB397, UniRef:UniRef100_G4NT40, UniRef:UniRef50_G4NT40, UniRef:UniRef90_G4NT40
SO:0001217, UniRef:UniRef50_G4NTG8, UniRef:UniRef90_G4NTG8
RefSeq:WP_044059163.1, SO:0001217, UniParc:UPI0005C53350, UniRef:UniRef100_UPI0005C53350, UniRef:UniRef50_UPI000F546693, UniRef:UniRef90_UPI0005C53350
RefSeq:WP_014112388.1, SO:0001217, UniParc:UPI00022BB39B, UniRef:UniRef100_G4NT44, UniRef:UniRef50_A0A653VNJ1, UniRef:UniRef90_G4NT44
RefSeq:WP_014112389.1, SO:0001217, UniParc:UPI00022BB39C, UniRef:UniRef100_G4NT45, UniRef:UniRef50_Q38579, UniRef:UniRef90_G4NT45
RefSeq:WP_014112390.1, SO:0001217, UniParc:UPI00022BB39D, UniRef:UniRef100_G4NTH3, UniRef:UniRef50_A0A150F498, UniRef:UniRef90_G4NTH3
RefSeq:WP_014112391.1, SO:0001217, UniParc:UPI00022BB39E, UniRef:UniRef100_G4NTH4, UniRef:UniRef50_M8DL86, UniRef:UniRef90_G4NTH4
EC:2.1.1.37, KEGG:K00558, RefSeq:WP_014112392.1, SO:0001217, UniParc:UPI00022BB39F, UniRef:UniRef100_G4NTH5, UniRef:UniRef50_G4NTH5, UniRef:UniRef90_G4NTH5
SO:0001217, UniParc:UPI0002D332B1, UniRef:UniRef100_A0A8I2B7B8, UniRef:UniRef50_Q65GH4, UniRef:UniRef90_G4NT49
RefSeq:WP_019712754.1, SO:0001217, UniParc:UPI0003112168, UniRef:UniRef100_UPI0003112168, UniRef:UniRef50_A0A2S0JZZ8, UniRef:UniRef90_A0A8I1WGL3
RefSeq:WP_014112394.1, SO:0001217, UniParc:UPI00022BB3A1, UniRef:UniRef100_G4NTH7, UniRef:UniRef50_G4NTH7, UniRef:UniRef90_G4NTH7
KEGG:K06909, RefSeq:WP_014112395.1, SO:0001217, UniParc:UPI00022BB3A2, UniRef:UniRef100_G4NTH8, UniRef:UniRef50_P39786, UniRef:UniRef90_G4NTH8
RefSeq:WP_014112396.1, SO:0001217, UniParc:UPI00022BB3A3, UniRef:UniRef100_G4NT52, UniRef:UniRef50_P45917, UniRef:UniRef90_G4NT52
RefSeq:WP_014112397.1, SO:0001217, UniParc:UPI00022BB3A4, UniRef:UniRef100_G4NT53, UniRef:UniRef50_P45918, UniRef:UniRef90_G4NT53
RefSeq:WP_014112398.1, SO:0001217, UniParc:UPI00022BB3A5, UniRef:UniRef100_G4NTI1, UniRef:UniRef50_G4NTI1, UniRef:UniRef90_G4NTI1
RefSeq:WP_014112399.1, SO:0001217, UniParc:UPI00022BB3A6, UniRef:UniRef100_G4NTI2, UniRef:UniRef50_A0A4R6TUE8, UniRef:UniRef90_G4NTI2
RefSeq:WP_014112400.1, SO:0001217, UniParc:UPI00022BB3A7, UniRef:UniRef100_G4NT56, UniRef:UniRef50_P54327, UniRef:UniRef90_G4NT56
RefSeq:WP_010330132.1, SO:0001217, UniParc:UPI00022BB3A8, UniRef:UniRef100_G4NT57, UniRef:UniRef50_P45922, UniRef:UniRef90_G4NT57
RefSeq:WP_010330131.1, SO:0001217, UniParc:UPI00022BB3A9, UniRef:UniRef100_G4NT58, UniRef:UniRef50_P45923, UniRef:UniRef90_G4NT58
RefSeq:WP_014112401.1, SO:0001217, UniParc:UPI00022BB3AA, UniRef:UniRef100_G4NT59, UniRef:UniRef50_P54328, UniRef:UniRef90_G4NT59
RefSeq:WP_014112402.1, SO:0001217, UniParc:UPI00022BB3AB, UniRef:UniRef100_G4NTI7, UniRef:UniRef50_P45925, UniRef:UniRef90_P45925
RefSeq:WP_014112403.1, SO:0001217, UniParc:UPI00022BB3AC, UniRef:UniRef100_G4NT61, UniRef:UniRef50_P45926, UniRef:UniRef90_G4NT61
RefSeq:WP_080010045.1, SO:0001217, UniParc:UPI0002E14426, UniRef:UniRef100_A0A8I2BAQ1, UniRef:UniRef50_A0A8I2BAQ1, UniRef:UniRef90_A0A8I2BAQ1
RefSeq:WP_014112405.1, SO:0001217, UniParc:UPI00022BB3AE, UniRef:UniRef100_G4NTJ0, UniRef:UniRef50_P45927, UniRef:UniRef90_P45927
RefSeq:WP_014112406.1, SO:0001217, UniParc:UPI00022BB3AF, UniRef:UniRef100_G4NT64, UniRef:UniRef50_P54332, UniRef:UniRef90_P54332
RFAM:RF01412, SO:0000655
RefSeq:WP_014112407.1, SO:0001217, UniParc:UPI00022BB3B0, UniRef:UniRef100_G4NT65, UniRef:UniRef50_P54333, UniRef:UniRef90_P45930
SO:0001217, UniRef:UniRef50_C0H3Z8, UniRef:UniRef90_A0A0D1KVX4
RefSeq:WP_014112409.1, SO:0001217, UniParc:UPI00022BB524, UniRef:UniRef100_G4NTJ4, UniRef:UniRef50_G4NTJ4, UniRef:UniRef90_G4NTJ4
RefSeq:WP_014112410.1, SO:0001217, UniParc:UPI00022BB525, UniRef:UniRef100_G4NTJ5, UniRef:UniRef50_P54335, UniRef:UniRef90_P45932
RefSeq:WP_014112411.1, SO:0001217, UniParc:UPI00022BB526, UniRef:UniRef100_G4NTJ6, UniRef:UniRef50_P54336, UniRef:UniRef90_P45950
RefSeq:WP_014112412.1, SO:0001217, UniParc:UPI00022BB527, UniRef:UniRef100_G4NTJ7, UniRef:UniRef50_P45933, UniRef:UniRef90_G4NTJ7
RefSeq:WP_014112413.1, SO:0001217, UniParc:UPI00022BB528, UniRef:UniRef100_G4NT71, UniRef:UniRef50_P45934, UniRef:UniRef90_P45934
RefSeq:WP_014112414.1, SO:0001217, UniParc:UPI00022BB529, UniRef:UniRef100_G4NTJ9, UniRef:UniRef50_P54339, UniRef:UniRef90_P45935
RefSeq:WP_014112415.1, SO:0001217, UniParc:UPI00022BB52A, UniRef:UniRef100_G4NT73, UniRef:UniRef50_P45936, UniRef:UniRef90_P45936
RefSeq:WP_014112416.1, SO:0001217, UniParc:UPI00022BB52B, UniRef:UniRef100_G4NT74, UniRef:UniRef50_P45937, UniRef:UniRef90_P45937
RefSeq:WP_014112417.1, SO:0001217, UniParc:UPI00022BB52C, UniRef:UniRef100_G4NTK2, UniRef:UniRef50_G4NTK2, UniRef:UniRef90_G4NTK2
RefSeq:WP_014112418.1, SO:0001217, UniParc:UPI00022BB52D, UniRef:UniRef100_G4NTK3, UniRef:UniRef50_P54342, UniRef:UniRef90_G4NTK3
RefSeq:WP_010330111.1, SO:0001217, UniParc:UPI00022BB52E, UniRef:UniRef100_G4NT77, UniRef:UniRef50_P54343, UniRef:UniRef90_P45940
RefSeq:WP_014112419.1, SO:0001217, UniParc:UPI00022BB52F, UniRef:UniRef100_G4NT78, UniRef:UniRef50_P39797, UniRef:UniRef90_G4NT78
RefSeq:WP_041520837.1, SO:0001217, UniParc:UPI00059F6ABE, UniRef:UniRef100_UPI00059F6ABE, UniRef:UniRef50_Q9ZXD8, UniRef:UniRef90_A0A9W5LK67
RefSeq:WP_014112421.1, SO:0001217, UniParc:UPI00022BB531, UniRef:UniRef100_G4NTK7, UniRef:UniRef50_P24808, UniRef:UniRef90_G4NTK7
RefSeq:WP_014112422.1, SO:0001217, UniParc:UPI00022BB532, UniRef:UniRef100_G4NTK8, UniRef:UniRef50_A0A0D1X892, UniRef:UniRef90_G4NTK8
EC:3.1.-.-, KEGG:K21491, RefSeq:WP_014112423.1, SO:0001217, UniParc:UPI00022BB533, UniRef:UniRef100_G4NT82, UniRef:UniRef50_P45942, UniRef:UniRef90_G4NT82
EC:3.1.-.-, KEGG:K06366, SO:0001217, UniParc:UPI00022BB534, UniRef:UniRef100_G4NT83, UniRef:UniRef50_Q59HN8, UniRef:UniRef90_G4NT83
RefSeq:WP_014112425.1, SO:0001217, UniParc:UPI00022BB535, UniRef:UniRef100_G4NTL1, UniRef:UniRef50_A0A0D1L3M0, UniRef:UniRef90_A0A0D1L3M0
RefSeq:WP_014112426.1, SO:0001217, UniParc:UPI00022BB536, UniRef:UniRef100_G4NT85, UniRef:UniRef50_G4NT85, UniRef:UniRef90_G4NT85
RefSeq:WP_014112427.1, SO:0001217, UniParc:UPI00022BB537, UniRef:UniRef100_G4NT86, UniRef:UniRef50_A0A0M9WIP5, UniRef:UniRef90_G4NT86
SO:0000260
SO:0001036
SO:0000268
SO:0000254
GO:0003735, GO:0005840, KEGG:K01977, RFAM:RF00177, SO:0001000
GO:0003735, GO:0005840, KEGG:K01980, RFAM:RF02541, SO:0001001
GO:0003735, GO:0005840, KEGG:K01985, RFAM:RF00001, SO:0000652
SO:0001217, UniRef:UniRef50_A0A9W5PJJ0, UniRef:UniRef90_G4NTL4
SO:0001217, UniRef:UniRef50_A0A3N6F8M2, UniRef:UniRef90_I2C0S5
RefSeq:WP_014112430.1, SO:0001217, UniParc:UPI00022BB82C, UniRef:UniRef100_G4NTL6, UniRef:UniRef50_A0A5P2UTJ9, UniRef:UniRef90_A0A2M8SXD5
SO:0001217, UniRef:UniRef50_A0A9W5PJI4, UniRef:UniRef90_G4NTL7
GO:0005886, GO:0008271, KEGG:K03321, RefSeq:WP_061186943.1, SO:0001217, UniParc:UPI0006A834D9, UniRef:UniRef100_A0A9Q4HIF9, UniRef:UniRef50_P55189, UniRef:UniRef90_P55189
GO:0005886, SO:0001217, UniParc:UPI00022BB82F, UniRef:UniRef100_G4NTL9, UniRef:UniRef50_P55190, UniRef:UniRef90_P55190
RefSeq:WP_087987868.1, SO:0001217, UniParc:UPI000B4526A4, UniRef:UniRef100_UPI000B4526A4, UniRef:UniRef50_P55192, UniRef:UniRef90_G4NTM0
SO:0001217, UniRef:UniRef50_UPI000261A256, UniRef:UniRef90_UPI000261A256
SO:0001217, UniRef:UniRef50_UPI002ACE0144
SO:0001217, UniRef:UniRef50_UPI00042142F9
BlastRules:WP_003234978, GO:0005737, GO:0005886, GO:0030288, GO:0045121, GO:1901678, RefSeq:WP_072181799.1, SO:0001217, UniParc:UPI0006A84E1A, UniRef:UniRef100_UPI0006A84E1A, UniRef:UniRef50_P4
SO:0001217, UniRef:UniRef50_P40408, UniRef:UniRef90_A0A5D4N7F6
EC:3.2.1.92, GO:0005576, GO:0033922, GO:0071555, SO:0001217, UniRef:UniRef50_P40407, UniRef:UniRef90_P40407
SO:0001217, UniRef:UniRef50_P40406, UniRef:UniRef90_E0U058
PFAM:PF00144.29
SO:0001217, UniRef:UniRef50_A0A5S9M455
PFAM:PF00144.29
SO:0001217, UniRef:UniRef50_A0A4Y1Z8F2
SO:0001217, UniRef:UniRef50_Q45579, UniRef:UniRef90_Q45579
GO:0003677, GO:0003700, GO:0006355, GO:0097367, GO:1901135, RefSeq:WP_268455850.1, SO:0001217, UniParc:UPI00227FFD4B, UniRef:UniRef100_UPI00227FFD4B, UniRef:UniRef50_Q45581, UniRef:UniRef90_Q45
EC:4.2.1.126, GO:0009254, GO:0016803, GO:0016835, GO:0046348, GO:0097173, GO:0097367, KEGG:K07106, SO:0001217, UniRef:UniRef50_Q45582, UniRef:UniRef90_Q45582
SO:0001217, UniRef:UniRef50_A0A1B1MZY1
RefSeq:WP_114151477.1, SO:0001217, UniParc:UPI000DEF894A, UniRef:UniRef100_UPI000DEF894A, UniRef:UniRef50_Q45584, UniRef:UniRef90_Q45584
SO:0000259
SO:0000273
SO:0000270
SO:0000272
SO:0000261
SO:0001217, UniRef:UniRef50_C0ZIM4
SO:0001217, UniRef:UniRef50_A0A5S9LZ76, UniRef:UniRef90_UPI0024AD8557
BlastRules:WP_003234951, BlastRules:WP_010896446, BlastRules:WP_011064630, BlastRules:WP_011229663, GO:0005886, GO:0046872, SO:0001217, UniRef:UniRef50_Q45588, UniRef:UniRef90_Q45588
BlastRules:WP_003223651, COG:COG1642, COG:T, EC:2.7.7.85, GO:0004016, GO:0005524, GO:0005886, GO:0006171, GO:0106408, KEGG:K18672, RefSeq:WP_003223651.1, SO:0001217, UniParc:UPI000195C502, UniF
GO:0005886, GO:0045762, SO:0001217, UniRef:UniRef50_O34659, UniRef:UniRef90_O34659
COG:COG1109, COG:G, EC:5.4.2.10, GO:0000287, GO:0004615, GO:0005829, GO:0005975, GO:0006048, GO:0008966, GO:0009252, RefSeq:WP_003223656.1, SO:0001217, UniParc:UPI0001CCAAEF, UniRef:UniRef100_
SO:0001217, UniRef:UniRef50_UPI00227DE055, UniRef:UniRef90_UPI00227DE055
RFAM:RF00234, SO:0005836

EC:2.6.1.16, GO:0004360, GO:0005829, GO:0005975, GO:0006002, GO:0006047, GO:0006487, GO:0097367, GO:1901137, KEGG:K00820, RefSeq:WP_003223659.1, SO:0001217, UniParc:UPI0001CCAAF0, UniRef:UniRef
EC:1.6.5.9, SO:0001217, UniRef:UniRef50_A0A6L8Q027, UniRef:UniRef90_E0U071
KEGG:K09822, SO:0001217, UniRef:UniRef50_Q5L2V2, UniRef:UniRef90_E0U072
SO:0001217, UniRef:UniRef50_O34823, UniRef:UniRef90_G4NQL2
RefSeq:WP_014112454.1, SO:0001217, UniParc:UPI00022BB843, UniRef:UniRef100_G4NQL3, UniRef:UniRef50_O34795, UniRef:UniRef90_G4NQL3
RefSeq:WP_014112455.1, SO:0001217, UniParc:UPI00022BB844, UniRef:UniRef100_G4NQL4, UniRef:UniRef50_O34380, UniRef:UniRef90_O34380
COG:COG0640, COG:K, GO:0003677, GO:0003700, GO:0006355, RefSeq:WP_014112457.1, SO:0001217, UniParc:UPI00022BB846, UniRef:UniRef100_G4NQL6, UniRef:UniRef50_C0H3S9, UniRef:UniRef90_C0H3S9
GO:0005886, GO:0022857, GO:0055085, KEGG:K08164, SO:0001217, UniRef:UniRef50_O34663, UniRef:UniRef90_O34663
BlastRules:WP_003234897, GO:0030152, RefSeq:WP_079288260.1, SO:0001217, UniParc:UPI0009A2D35C, UniRef:UniRef100_UPI0009A2D35C, UniRef:UniRef50_O31429, UniRef:UniRef90_O31429
RefSeq:WP_079288259.1, SO:0001217, UniParc:UPI0009A35985, UniRef:UniRef100_UPI0009A35985, UniRef:UniRef50_A0A5M9H3W9, UniRef:UniRef90_G4NQL9
RefSeq:WP_079288258.1, SO:0001217, UniParc:UPI0009A34332, UniRef:UniRef100_UPI0009A34332, UniRef:UniRef50_A0A0J1G5N5, UniRef:UniRef90_G4NQM0
GO:0005886, RefSeq:WP_079288257.1, SO:0001217, UniParc:UPI00086D9C1B, UniRef:UniRef100_UPI00086D9C1B, UniRef:UniRef50_O31431, UniRef:UniRef90_O31431
BlastRules:WP_003234895, GO:0000156, GO:0000976, GO:0005829, GO:0006355, GO:0032993, RefSeq:WP_090557451.1, SO:0001217, UniParc:UPI00086937AD, UniRef:UniRef100_UPI00086937AD, UniRef:UniRef50_O3
EC:2.7.13.3, GO:0000155, GO:0005524, GO:0005886, RefSeq:WP_205635571.1, SO:0001217, UniParc:UPI0006A8A5A7, UniRef:UniRef100_UPI0006A8A5A7, UniRef:UniRef50_O31433, UniRef:UniRef90_O31433
SO:0001217, UniRef:UniRef50_C0H3T1, UniRef:UniRef90_UPI0005A2AE22
RefSeq:WP_014112467.1, SO:0001217, UniParc:UPI00022BB9E0, UniRef:UniRef100_G4NQM6, UniRef:UniRef50_O31435, UniRef:UniRef90_A0A6H0WH21
RefSeq:WP_072182589.1, SO:0001217, UniParc:UPI0006A9177F, UniRef:UniRef100_UPI0006A9177F, UniRef:UniRef50_O31436, UniRef:UniRef90_O31436
RefSeq:WP_072182590.1, SO:0001217, UniParc:UPI0006A91A41, UniRef:UniRef100_UPI0006A91A41, UniRef:UniRef50_O31437, UniRef:UniRef90_O31437
COG:COG1113, COG:E, GO:0005886, GO:0006865, GO:0055085, RefSeq:WP_088680180.1, SO:0001217, UniParc:UPI0006A91A89, UniRef:UniRef100_UPI0006A91A89, UniRef:UniRef50_P27837, UniRef:UniRef90_P54425
BlastRules:WP_003234887, GO:0030435, SO:0001217, UniRef:UniRef50_P54379, UniRef:UniRef90_P54379
RefSeq:WP_041521120.1, SO:0001217, UniParc:UPI0005A1BFD7, UniRef:UniRef100_G4NQN1, UniRef:UniRef50_P54426, UniRef:UniRef90_A0A9W5LFM4
SO:0001217, UniRef:UniRef50_A0A839TW85
EC:3.5.2.6, GO:0008800, GO:0030655, SO:0001217, UniRef:UniRef50_A0A3S0V8J4, UniRef:UniRef90_A0AAP3CHA5
EC:1.11.2.4, KEGG:K15629, SO:0001217, UniRef:UniRef50_W6SD13, UniRef:UniRef90_A0A5D4N6A5
RefSeq:WP_014112476.1, SO:0001217, UniParc:UPI00022BB9E9, UniRef:UniRef100_G4NQN5, UniRef:UniRef50_O31441, UniRef:UniRef90_O31441
RefSeq:WP_061186969.1, SO:0001217, UniParc:UPI0006A88F2C, UniRef:UniRef100_UPI0006A88F2C, UniRef:UniRef50_A0A5P9HPA1, UniRef:UniRef90_A0A9W5PBK8
EC:3.1.4.46, GO:0005576, GO:0006071, GO:0006629, GO:0008889, GO:0046872, GO:0071555, KEGG:K01126, RefSeq:WP_090557442.1, SO:0001217, UniParc:UPI0008688055, UniRef:UniRef100_UPI0008688055, UniRef:
GO:0005886, GO:0006071, GO:0015169, GO:0015760, GO:0015794, GO:0016020, GO:0035435, GO:0061513, KEGG:K02445, SO:0001217, UniRef:UniRef50_P37948, UniRef:UniRef90_P37948
RefSeq:WP_087987937.1, SO:0001217, UniParc:UPI00086A847D, UniRef:UniRef100_UPI00086A847D, UniRef:UniRef50_O31442, UniRef:UniRef90_E0U0A6
EC:2.3.1.-, GO:0003677, GO:0003700, GO:0008080, RefSeq:WP_090557440.1, SO:0001217, UniParc:UPI000B88ABA8, UniRef:UniRef100_UPI000B88ABA8, UniRef:UniRef50_O31443, UniRef:UniRef90_O31443
GO:0005886, GO:0022857, RefSeq:WP_014112483.1, SO:0001217, UniParc:UPI00022BB9F0, UniRef:UniRef100_G4NQP2, UniRef:UniRef50_O31444, UniRef:UniRef90_O31444
SO:0001217, UniRef:UniRef50_O31447, UniRef:UniRef90_UPI000301110B
GO:0005886, SO:0001217, UniRef:UniRef50_O31448, UniRef:UniRef90_O31448
SO:0001217, UniRef:UniRef50_A0A0D1KY59, UniRef:UniRef90_A0A0D1KY59
SO:0001217, UniRef:UniRef50_UPI002E8E5EAA
EC:6.3.1.21, GO:0000287, GO:0004644, GO:0005524, GO:0005829, GO:0006189, GO:0009152, GO:0016742, GO:0043815, KEGG:K08289, RefSeq:WP_044444766.1, SO:0001217, UniParc:UPI0005CA0576, UniRef:UniRef1
BlastRules:WP_003246370, EC:3.4.21.-, GO:0004252, GO:0005576, GO:0006508, RefSeq:WP_072181575.1, SO:0001217, UniParc:UPI0006A884AF, UniRef:UniRef100_UPI0006A884AF, UniRef:UniRef50_P39790, UniRef:Un
SO:0001217, UniRef:UniRef50_O31451, UniRef:UniRef90_O31451
SO:0001217, UniRef:UniRef50_P96688, UniRef:UniRef90_G4NQQ7
EC:2.7.8.8, GO:0003882, GO:0008654, KEGG:K17103, SO:0001217, UniRef:UniRef50_P39823, UniRef:UniRef90_A0AAP3CH40
GO:0005886, KEGG:K03975, RefSeq:WP_041520844.1, SO:0001217, UniParc:UPI00030D1459, UniRef:UniRef100_A0A9Q4E9Y0, UniRef:UniRef50_O31453, UniRef:UniRef90_O31453
EC:4.1.1.65, GO:0004609, GO:0005886, GO:0006646, GO:0008654, KEGG:K01613, RefSeq:WP_072181577.1, SO:0001217, UniParc:UPI0006A81CFD, UniRef:UniRef100_UPI0006A81CFD, UniRef:UniRef50_P39822, UniRef
SO:0001217, UniRef:UniRef50_O31454, UniRef:UniRef90_A0A6H0WEN7
EC:3.1.1.-, KEGG:K06880, SO:0001217, UniRef:UniRef50_O31455, UniRef:UniRef90_A0A5D4N6W5
EC:1.14.-.-, GO:0006400, GO:0016705, KEGG:K07146, RefSeq:WP_003223769.1, SO:0001217, UniParc:UPI0001D2864B, UniRef:UniRef100_G4NQR2, UniRef:UniRef50_Q2NIJ6, UniRef:UniRef90_O31457
BlastRules:WP_003234847, GO:0005886, GO:0006835, GO:0006865, GO:0015293, GO:0022857, KEGG:K11102, RefSeq:WP_014112503.1, SO:0001217, UniParc:UPI00022BBA04, UniRef:UniRef100_G4NQR3, UniRef:UniR
PFAM:PF00358.26
EC:2.7.1.-, GO:0005886, GO:0008982, GO:0009401, GO:0016301, GO:0055056, GO:0090563, GO:0103111, GO:1904659, KEGG:K02765, SO:0001217, UniRef:UniRef50_P39816, UniRef:UniRef90_P39816
EC:3.5.99.6, GO:0004342, GO:0005737, GO:0005975, GO:0006043, GO:0006044, GO:0006046, GO:0019262, GO:0042802, SO:0001217, UniRef:UniRef50_O31458, UniRef:UniRef90_O31458
BlastRules:WP_003234841, GO:0003677, GO:0003700, GO:0045892, KEGG:K24966, SO:0001217, UniRef:UniRef50_O31459, UniRef:UniRef90_O31459
RefSeq:WP_061186986.1, SO:0001217, UniParc:UPI0006A8D4F4, UniRef:UniRef100_UPI0006A8D4F4, UniRef:UniRef50_O31460, UniRef:UniRef90_G4NQR7
EC:2.6.1.42, GO:0004084, GO:0009081, GO:0009097, GO:0009098, GO:0009099, GO:0052655, GO:0052656, KEGG:K00826, SO:0001217, UniRef:UniRef50_O31461, UniRef:UniRef90_O31461
GO:0003333, GO:0005886, GO:0015171, GO:0016020, GO:0055085, KEGG:K16235, RefSeq:WP_061186988.1, SO:0001217, UniParc:UPI0006A904DE, UniRef:UniRef100_A0A9Q4E601, UniRef:UniRef50_P42087, UniRef:Ur
EC:2.1.1.10, GO:0008270, GO:0008898, GO:0009086, GO:0032259, GO:0033528, GO:0047150, GO:0061627, KEGG:K00547, RefSeq:WP_326201829.1, SO:0001217, UniParc:UPI002DB756FF, UniRef:UniRef100_UPI002DB
GO:0005283, GO:0005886, KEGG:K11626, RefSeq:WP_072181582.1, SO:0001217, UniParc:UPI0006A88876, UniRef:UniRef100_UPI0006A88876, UniRef:UniRef50_O31464, UniRef:UniRef90_O31464
EC:3.5.1.2, GO:0004359, GO:0006537, GO:0006543, RefSeq:WP_072181583.1, SO:0001217, UniParc:UPI0006A8B4DE, UniRef:UniRef100_UPI0006A8B4DE, UniRef:UniRef50_O31465, UniRef:UniRef90_O31465
BlastRules:WP_010886394, EC:2.7.13.3, GO:0000155, GO:0005524, GO:0005886, KEGG:K07717, SO:0001217, UniRef:UniRef50_P40758, UniRef:UniRef90_P40758
SO:0001217, UniRef:UniRef50_A0A017RXA3
EC:4.2.1.41, GO:0008840, GO:0016052, GO:0042838, GO:0047448, SO:0001217, UniRef:UniRef50_P42235, UniRef:UniRef90_P42235
EC:1.2.1.26, GO:0047533, SO:0001217, UniRef:UniRef50_P42236, UniRef:UniRef90_P42236
BlastRules:WP_003234818, GO:0005886, GO:0022857, RefSeq:WP_014112517.1, SO:0001217, UniParc:UPI00022BBBA4, UniRef:UniRef100_G4NTN2, UniRef:UniRef50_P0AA80, UniRef:UniRef90_P42237
EC:4.2.1.40, GO:0000287, GO:0008872, GO:0019394, GO:0042838, KEGG:K01706, SO:0001217, UniRef:UniRef50_Q6FFQ2, UniRef:UniRef90_P42238
GO:0003677, GO:0003700, RefSeq:WP_003223808.1, SO:0001217, UniParc:UPI0001D2865F, UniRef:UniRef100_A0A9Q4DN87, UniRef:UniRef50_P42239, UniRef:UniRef90_P42239
EC:4.2.1.42, GO:0008867, GO:0019698, GO:0046392, GO:0046872, KEGG:K01708, SO:0001217, UniRef:UniRef50_P39829, UniRef:UniRef90_P42240
BlastRules:WP_003246254, KEGG:K06979, NCBIProtein:WP_003246254.1, SO:0001217, UniRef:UniRef50_V6LZM1, UniRef:UniRef90_A0A9Q4E6Z5
RFAM:RF00230, SO:0000140
RefSeq:WP_014112522.1, SO:0001217, UniParc:UPI00022BBBA9, UniRef:UniRef100_G4NTN7, UniRef:UniRef50_O31466, UniRef:UniRef90_G4NTN7
SO:0001217, UniRef:UniRef50_P42243, UniRef:UniRef90_G4NTN8
BlastRules:WP_009966453, GO:0000156, GO:0000976, GO:0005829, GO:0006355, GO:0032993, SO:0001217, UniRef:UniRef50_P42244, UniRef:UniRef90_P42244
SO:0001217, UniRef:UniRef50_A0A5D4N6A0, UniRef:UniRef90_A0A5D4N6A0
GO:0005524, GO:0016887, RefSeq:WP_014112526.1, SO:0001217, UniParc:UPI00022BBBAD, UniRef:UniRef100_G4NTP1, UniRef:UniRef50_P42246, UniRef:UniRef90_P42246
GO:0005886, SO:0001217, UniRef:UniRef50_P42247, UniRef:UniRef90_P42247
GO:0005886, RefSeq:WP_014112528.1, SO:0001217, UniParc:UPI00022BBBAF, UniRef:UniRef100_G4NTP3, UniRef:UniRef50_P42248, UniRef:UniRef90_P42248
SO:0001217, UniRef:UniRef50_E0U0W9, UniRef:UniRef90_G4NTP4
BlastRules:WP_003246500, GO:0005576, GO:0016787, GO:0030435, GO:0071555, RefSeq:WP_014112530.1, SO:0001217, UniParc:UPI00022BBBB1, UniRef:UniRef100_G4NTP5, UniRef:UniRef50_P42249, UniRef:UniRef9
SO:0001217, UniRef:UniRef50_P42250, UniRef:UniRef90_P42250
BlastRules:WP_009969242, EC:3.1.3.1, GO:0004035, SO:0001217, UniRef:UniRef50_P42251, UniRef:UniRef90_P42251
BlastRules:WP_003223835, GO:0005829, GO:0008320, GO:0033281, GO:0043953, RefSeq:WP_003223835.1, SO:0001217, UniParc:UPI000195C521, UniRef:UniRef100_O31467, UniRef:UniRef50_O31467, UniRef:UniRef90
GO:0005886, GO:0015628, KEGG:K03118, RefSeq:WP_014112534.1, SO:0001217, UniParc:UPI00022BBBB5, UniRef:UniRef100_G4NTQ0, UniRef:UniRef50_P42252, UniRef:UniRef90_G4NTQ0
EC:3.4.19.3, GO:0005829, GO:0006508, GO:0008234, GO:0016920, KEGG:K01304, RefSeq:WP_014112535.1, SO:0001217, UniParc:UPI00022BBBB6, UniRef:UniRef100_G4NTQ1, UniRef:UniRef50_P46107, UniRef:UniRef9
RefSeq:WP_326201786.1, SO:0001217, UniParc:UPI002DBCD6E5, UniRef:UniRef100_UPI002DBCD6E5, UniRef:UniRef50_P42253, UniRef:UniRef90_G4NTQ2
GO:0005886, GO:0016020, GO:0022857, GO:0046677, RefSeq:WP_014112537.1, SO:0001217, UniParc:UPI00022BBBB8, UniRef:UniRef100_G4NTQ3, UniRef:UniRef50_O35018, UniRef:UniRef90_O35018
BlastRules:WP_003246449, GO:0003677, GO:0006355, KEGG:K18939, RefSeq:WP_087987904.1, SO:0001217, UniParc:UPI000B43FA4B, UniRef:UniRef100_UPI000B43FA4B, UniRef:UniRef50_O34619, UniRef:UniRef90_O
EC:3.5.1.1, GO:0004067, GO:0006530, GO:0042597, SO:0001217, UniRef:UniRef50_P06608, UniRef:UniRef90_O34482
EC:3.1.1.3, KEGG:K01046, RefSeq:WP_014112540.1, SO:0001217, UniParc:UPI00022BBBBB, UniRef:UniRef100_G4NTQ6, UniRef:UniRef50_P37957, UniRef:UniRef90_G4NTQ6
GO:0005886, SO:0001217, UniParc:UPI00022BA3DA, UniRef:UniRef100_G4NTQ7, UniRef:UniRef50_O31469, UniRef:UniRef90_O31469
GO:0003677, GO:0005524, RefSeq:WP_072182363.1, SO:0001217, UniParc:UPI0006A8246C, UniRef:UniRef100_UPI0006A8246C, UniRef:UniRef50_O34478, UniRef:UniRef90_O34478
EC:2.7.13.3, KEGG:K11640, SO:0001217, UniRef:UniRef50_P70954, UniRef:UniRef90_G4NTQ9
BlastRules:WP_003234759, GO:0000156, GO:0000976, GO:0005829, GO:0006355, GO:0032993, KEGG:K11641, RefSeq:WP_072182365.1, SO:0001217, UniParc:UPI0006A8037E, UniRef:UniRef100_UPI0006A8037E, UniRe
BlastRules:WP_003234757, GO:0005524, GO:0008554, GO:0016887, GO:0140679, SO:0001217, UniRef:UniRef50_P46903, UniRef:UniRef90_P46903
BlastRules:WP_003246346, GO:0005886, GO:0008554, GO:0140679, KEGG:K09696, RefSeq:WP_014112546.1, SO:0001217, UniParc:UPI00022BA3DF, UniRef:UniRef100_G4NTR2, UniRef:UniRef50_P46904, UniRef:UniRef
EC:1.-.-.-, GO:0005829, GO:0016491, KEGG:K06607, RefSeq:WP_072182366.1, SO:0001217, UniParc:UPI0006A8F7B4, UniRef:UniRef100_UPI0006A8F7B4, UniRef:UniRef50_P46336, UniRef:UniRef90_P46905
GO:0005886, GO:0045121, RefSeq:WP_014112548.1, SO:0001217, UniParc:UPI00022BA3E1, UniRef:UniRef100_G4NTR4, UniRef:UniRef50_O34538, UniRef:UniRef90_O34538
SO:0001217, UniRef:UniRef50_O34621, UniRef:UniRef90_E0U1J3
SO:0001217, UniRef:UniRef50_O34772, UniRef:UniRef90_A0A9W5PBK5
BlastRules:WP_009966473, EC:3.4.-.-, GO:0005886, GO:0008233, GO:0071555, RefSeq:WP_014112552.1, SO:0001217, UniParc:UPI00022BA3E5, UniRef:UniRef100_G4NTR8, UniRef:UniRef50_O34360, UniRef:UniRef90_(
BlastRules:WP_003246478, EC:3.1.3.-, GO:0004721, GO:0005737, GO:0030435, KEGG:K06368, SO:0001217, UniRef:UniRef50_O34327, UniRef:UniRef90_O34327
EC:1.1.1.47, GO:0016616, GO:0047934, GO:0047935, RefSeq:WP_061187014.1, SO:0001217, UniParc:UPI0006A85813, UniRef:UniRef100_UPI0006A85813, UniRef:UniRef50_P40288, UniRef:UniRef90_P80869
EC:3.2.1.10, GO:0004556, GO:0004574, GO:0005737, GO:0009313, RefSeq:WP_087987908.1, SO:0001217, UniParc:UPI000B449399, UniRef:UniRef100_A0A9Q4E5T4, UniRef:UniRef50_O34364, UniRef:UniRef90_O34364
GO:0005886, GO:0006829, GO:0007155, GO:0045121, GO:0046872, RefSeq:WP_014112556.1, SO:0001217, UniParc:UPI00022BA3E9, UniRef:UniRef100_G4NTS2, UniRef:UniRef50_O34966, UniRef:UniRef90_O34966
BlastRules:WP_003234730, EC:7.2.2.20, GO:0005524, GO:0015633, GO:0016887, GO:0042626, GO:0043190, RefSeq:WP_072182371.1, SO:0001217, UniParc:UPI0006A7F7D9, UniRef:UniRef100_UPI0006A7F7D9, UniRef:U
BlastRules:WP_003246340, GO:0005886, GO:0006829, GO:0010043, GO:0043190, GO:0055085, RefSeq:WP_053407028.1, SO:0001217, UniParc:UPI0006A8A301, UniRef:UniRef100_A0A9Q4EA22, UniRef:UniRef50_O3461
GO:0005829, GO:0016705, RefSeq:WP_242734831.1, SO:0001217, UniParc:UPI001F626160, UniRef:UniRef100_UPI001F626160, UniRef:UniRef50_O34504, UniRef:UniRef90_O34504
GO:0005737, RefSeq:WP_014112559.1, SO:0001217, UniParc:UPI00022BA3EC, UniRef:UniRef100_G4NTS5, UniRef:UniRef50_P81100, UniRef:UniRef90_P81100
RefSeq:WP_003224762.1, SO:0001217, UniParc:UPI0001D2902C, UniRef:UniRef100_A0A9Q4DR03, UniRef:UniRef50_P80875, UniRef:UniRef90_P80875
SO:0001217, UniRef:UniRef50_O34384, UniRef:UniRef90_O34384
GO:0005886, RefSeq:WP_014112562.1, SO:0001217, UniParc:UPI00022BA3EF, UniRef:UniRef100_G4NTS8, UniRef:UniRef50_O34447, UniRef:UniRef90_O34447
PFAM:PF14266.11
SO:0001217, UniRef:UniRef50_O34809, UniRef:UniRef90_O34809
SO:0001217, UniRef:UniRef50_A0A5S9M185
SO:0001217, UniRef:UniRef50_O34833, UniRef:UniRef90_O34833
BlastRules:WP_003246397, RefSeq:WP_079288221.1, SO:0001217, UniParc:UPI00086BB88A, UniRef:UniRef100_A0A9Q4E720, UniRef:UniRef50_A0A2S0MBC2, UniRef:UniRef90_A0AAP3CIQ8
GO:0003677, GO:0003700, GO:0006355, SO:0001217, UniRef:UniRef50_O34464, UniRef:UniRef90_O34464
SO:0001217, UniRef:UniRef50_A0A849WPK5
SO:0001217, UniRef:UniRef50_UPI0034649AD4
BlastRules:WP_003234703, GO:0005275, GO:0006865, GO:0015226, GO:0015871, GO:0031460, GO:0043190, GO:1902603, KEGG:K02001, RefSeq:WP_014112569.1, SO:0001217, UniParc:UPI00022BA3F6, UniRef:UniRe
BlastRules:WP_003246462, GO:0006865, GO:0022857, GO:0043190, GO:0045121, RefSeq:WP_014112570.1, SO:0001217, UniParc:UPI00022BA3F7, UniRef:UniRef100_G4NTT6, UniRef:UniRef50_Q9RR44, UniRef:UniRef9
EC:3.5.1.-, GO:0016787, KEGG:K14665, SO:0001217, UniRef:UniRef50_P54983, UniRef:UniRef90_P54983
GO:0005886, RefSeq:WP_072182379.1, SO:0001217, UniParc:UPI0006A8C5BE, UniRef:UniRef100_UPI0006A8C5BE, UniRef:UniRef50_P55908, UniRef:UniRef90_P55908
SO:0001217, UniRef:UniRef50_P55909, UniRef:UniRef90_P55909
EC:3.2.1.1, KEGG:K01176, SO:0001217, UniRef:UniRef50_P00691, UniRef:UniRef90_A0A5D4N7S5
EC:1.1.1.27, GO:0004459, GO:0005737, GO:0006089, GO:0006090, GO:0006096, KEGG:K00016, SO:0001217, UniRef:UniRef50_P00343, UniRef:UniRef90_P13714
GO:0005886, GO:0015129, GO:0015295, SO:0001217, UniRef:UniRef50_P55910, UniRef:UniRef90_P55910
BlastRules:WP_003246377, RefSeq:WP_014112577.1, SO:0001217, UniParc:UPI00022BA565, UniRef:UniRef100_G4NTU3, UniRef:UniRef50_P96712, UniRef:UniRef90_I2C146
GO:0003677, GO:0003700, GO:0006355, GO:0006950, RefSeq:WP_061187021.1, SO:0001217, UniParc:UPI0006A7F506, UniRef:UniRef100_A0A9Q4HHH9, UniRef:UniRef50_O31472, UniRef:UniRef90_O31472
GO:0005886, GO:0006865, GO:0015171, KEGG:K06895, RefSeq:WP_014112579.1, SO:0001217, UniParc:UPI00022BA567, UniRef:UniRef100_A0A9Q4HMR9, UniRef:UniRef50_P94381, UniRef:UniRef90_P94381
KEGG:K09190, RefSeq:WP_014112580.1, SO:0001217, UniParc:UPI00022BA568, UniRef:UniRef100_G4NTU6, UniRef:UniRef50_P94382, UniRef:UniRef90_P94382
GO:0005886, GO:0006865, GO:0055085, SO:0001217, UniRef:UniRef50_P94383, UniRef:UniRef90_P94383
SO:0001217, UniRef:UniRef50_O31473, UniRef:UniRef90_O31473
BlastRules:WP_000174879, BlastRules:WP_003246440, EC:6.3.1.5, GO:0003952, GO:0004359, GO:0005524, GO:0005737, GO:0008795, GO:0009435, GO:0030435, GO:0046872, RefSeq:WP_014112583.1, SO:0001217, U
RFAM:RF01690, SO:0000655
BlastRules:WP_003246258, SO:0001217, UniRef:UniRef50_P12921, UniRef:UniRef90_A0A9Q4E4W9
RefSeq:WP_003224806.1, SO:0001217, UniParc:UPI0001D282C9, UniRef:UniRef100_A0A9Q4DQC4, UniRef:UniRef50_P37944, UniRef:UniRef90_A0A6H0WFA9
SO:0001217, UniRef:UniRef50_O31474, UniRef:UniRef90_D4G4X6

GO:0003677, GO:0003700, GO:0006355, GO:0032993, RefSeq:WP_327816577.1, SO:0001217, UniParc:UPI002E1FCFCA, UniRef:UniRef100_UPI002E1FCFCA, UniRef:UniRef50_P94387, UniRef:UniRef90_P94387
BlastRules:WP_003246400, EC:3.1.1.41, GO:0005737, GO:0005976, GO:0030245, GO:0046555, GO:0047739, GO:0052689, SO:0001217, UniRef:UniRef50_P94388, UniRef:UniRef90_P94388
KEGG:K07074, SO:0001217, UniRef:UniRef50_P94389, UniRef:UniRef90_P94389
EC:1.5.5.2, GO:0004657, GO:0006562, GO:0010133, GO:0071949, SO:0001217, UniRef:UniRef50_P94390, UniRef:UniRef90_P94390
SO:0001217, UniRef:UniRef50_UPI0020D212FA
SO:0001217, UniRef:UniRef50_A0A6L3ZWN9, UniRef:UniRef90_UPI001923A4D3
GO:0005298, GO:0006814, GO:0015824, GO:0016020, GO:0031402, SO:0001217, UniRef:UniRef50_P94392, UniRef:UniRef90_A0A5D4N6V2
BlastRules:WP_003234651, GO:0003677, RefSeq:WP_003224834.1, SO:0001217, UniParc:UPI0001D282D0, UniRef:UniRef100_A0A9Q4DRF2, UniRef:UniRef50_P94393, UniRef:UniRef90_P94393
GO:0005886, SO:0001217, UniRef:UniRef50_P94394, UniRef:UniRef90_P94394
GO:0005886, KEGG:K07089, SO:0001217, UniRef:UniRef50_P94395, UniRef:UniRef90_P94395
SO:0001217, UniRef:UniRef50_P94396, UniRef:UniRef90_G4NQS7
BlastRules:WP_003234643, EC:1.18.1.2, GO:0004324, GO:0004791, GO:0045454, GO:0050660, GO:0050661, GO:0071281, KEGG:K21567, RefSeq:WP_072182391.1, SO:0001217, UniParc:UPI0006A7FFFD, UniRef:UniRef
EC:2.1.1.107, EC:4.2.1.75, KEGG:K13542, SO:0001217, UniRef:UniRef50_P42437, UniRef:UniRef90_E0U1P4
BlastRules:WP_003234637, EC:1.7.1.4, GO:0020037, GO:0042128, GO:0046872, GO:0050660, GO:0050661, GO:0051537, GO:0051539, GO:0106316, RefSeq:WP_072182393.1, SO:0001217, UniParc:UPI0006A89D4F, UniF
EC:1.7.-.-, GO:0016491, GO:0019333, GO:0042128, GO:0043546, GO:0046872, GO:0051539, SO:0001217, UniRef:UniRef50_P42434, UniRef:UniRef90_P42434
SO:0001217, UniRef:UniRef50_W4QA72, UniRef:UniRef90_UPI0011A0236D
RefSeq:WP_072182395.1, SO:0001217, UniParc:UPI0006A886F5, UniRef:UniRef100_UPI0006A886F5, UniRef:UniRef50_P42433, UniRef:UniRef90_G4NQT2
GO:0005886, GO:0015112, GO:0015113, GO:0042128, KEGG:K02575, RefSeq:WP_014112599.1, SO:0001217, UniParc:UPI00022BA57C, UniRef:UniRef100_G4NQT3, UniRef:UniRef50_P42432, UniRef:UniRef90_P42432
EC:3.5.4.16, GO:0003933, GO:0003934, GO:0046654, KEGG:K09007, SO:0001217, UniRef:UniRef50_P94398, UniRef:UniRef90_P94398
SO:0001217, UniRef:UniRef50_D4G4Z5, UniRef:UniRef90_D4G4Z5
SO:0001217, UniRef:UniRef50_G4NQT6, UniRef:UniRef90_G4NQT6
BlastRules:WP_003234625, EC:3.6.5.-, GO:0005525, GO:0016787, RefSeq:WP_014112603.1, SO:0001217, UniParc:UPI00022BA580, UniRef:UniRef100_G4NQT7, UniRef:UniRef50_P94400, UniRef:UniRef90_P94400
GO:0005886, GO:0006865, GO:0022857, GO:0043190, RefSeq:WP_003224863.1, SO:0001217, UniParc:UPI0001D282DF, UniRef:UniRef100_G4NQT8, UniRef:UniRef50_P42399, UniRef:UniRef90_P42399
GO:0005886, RefSeq:WP_072182399.1, SO:0001217, UniParc:UPI0006A8EAF3, UniRef:UniRef100_G4NQT9, UniRef:UniRef50_P42400, UniRef:UniRef90_P42400
SO:0001217, UniParc:UPI00022BA582, UniRef:UniRef100_G4NQU0, UniRef:UniRef50_G4NQU0, UniRef:UniRef90_G4NQU0
GO:0005886, RefSeq:WP_014112607.1, SO:0001217, UniParc:UPI00022BA584, UniRef:UniRef100_G4NQU2, UniRef:UniRef50_P42401, UniRef:UniRef90_P42401
RefSeq:WP_090557354.1, SO:0001217, UniParc:UPI00086AF6A6, UniRef:UniRef100_UPI00086AF6A6, UniRef:UniRef50_P42402, UniRef:UniRef90_P42402
RefSeq:WP_088680288.1, SO:0001217, UniParc:UPI0006A8B374, UniRef:UniRef100_A0A9Q4E749, UniRef:UniRef50_A0A3M7TQJ0, UniRef:UniRef90_D4G503
RefSeq:WP_014112610.1, SO:0001217, UniParc:UPI00022BA587, UniRef:UniRef100_G4NQU5, UniRef:UniRef50_E0U2B0, UniRef:UniRef90_E0U2B0
RefSeq:WP_072182402.1, SO:0001217, UniParc:UPI0006A80DEF, UniRef:UniRef100_UPI0006A80DEF, UniRef:UniRef50_E0U2B1, UniRef:UniRef90_E0U2B1
BlastRules:WP_003246242, EC:3.2.1.86, GO:0005829, GO:0008422, GO:0008706, GO:0016052, RefSeq:WP_072182403.1, SO:0001217, UniParc:UPI0006A8FAC6, UniRef:UniRef100_UPI0006A8FAC6, UniRef:UniRef50_P42
BlastRules:WP_003246342, RefSeq:WP_072182404.1, SO:0001217, UniParc:UPI0006A8A99A, UniRef:UniRef100_UPI0006A8A99A, UniRef:UniRef50_P12669, UniRef:UniRef90_G4NQU8
BlastRules:WP_000811492, BlastRules:WP_010886401, EC:3.-.-.-, GO:0004518, GO:0005886, GO:0030420, SO:0001217, UniRef:UniRef50_P12667, UniRef:UniRef90_P12667
GO:0004888, GO:0005886, GO:0006935, GO:0007165, SO:0001217, UniRef:UniRef50_P39209, UniRef:UniRef90_P39209
EC:5.3.1.27, GO:0009636, GO:0019647, GO:0043800, GO:0097367, GO:1901135, KEGG:K08094, RefSeq:WP_072182406.1, SO:0001217, UniParc:UPI0006A84C67, UniRef:UniRef100_UPI0006A84C67, UniRef:UniRef50_P4
EC:4.1.2.43, GO:0004590, GO:0006207, GO:0006730, GO:0009636, GO:0019647, GO:0019854, GO:0033982, GO:0043801, RefSeq:WP_014112617.1, SO:0001217, UniParc:UPI00022BA6FA, UniRef:UniRef100_A0A9Q4E7K
BlastRules:WP_003246265, GO:0003677, RefSeq:WP_014112618.1, SO:0001217, UniParc:UPI00022BA6FB, UniRef:UniRef100_G4NQV3, UniRef:UniRef50_P42406, UniRef:UniRef90_P42406
EC:6.1.1.13, GO:0005524, GO:0019350, GO:0047473, SO:0001217, UniRef:UniRef50_A0A2W1N839, UniRef:UniRef90_UPI00119D3E92
SO:0001217, UniRef:UniRef50_A0A9Q4EJX2
BlastRules:WP_010886403, BlastRules:WP_057766253, EC:6.1.1.13, GO:0005524, GO:0005737, GO:0016874, GO:0017000, GO:0019350, GO:0030435, GO:0031177, GO:0043041, GO:0044550, GO:0047473, SO:0001217,
SO:0001217, UniRef:UniRef50_UPI00366B0B69, UniRef:UniRef90_UPI00366B0B69
PFAM:PF00550.31, PFAM:PF00975.26, PFAM:PF13193.12
BlastRules:WP_003234568, BlastRules:WP_007609310, EC:3.1.2.-, GO:0005737, GO:0008610, GO:0016787, GO:0017000, GO:0030435, KEGG:K15657, RefSeq:WP_072183602.1, SO:0001217, UniParc:UPI0006A859DA, U
GO:0005886, GO:0022857, RefSeq:WP_088680294.1, SO:0001217, UniParc:UPI000B5306C5, UniRef:UniRef100_UPI000B5306C5, UniRef:UniRef50_Q08791, UniRef:UniRef90_Q08791
RefSeq:WP_072183603.1, SO:0001217, UniParc:UPI0006A91502, UniRef:UniRef100_UPI0006A91502, UniRef:UniRef50_Q08793, UniRef:UniRef90_E0U2C5
GO:0005886, RefSeq:WP_072183604.1, SO:0001217, UniParc:UPI0006A89AB3, UniRef:UniRef100_UPI0006A89AB3, UniRef:UniRef50_Q08794, UniRef:UniRef90_Q08794
SO:0001217, UniRef:UniRef50_Q08792
EC:2.7.8.7, GO:0000287, GO:0005829, GO:0006633, GO:0008897, GO:0017000, GO:0019878, GO:1900192, SO:0001217, UniRef:UniRef50_P39135, UniRef:UniRef90_P39135
PFAM:PF22624.1
GO:0005886, KEGG:K07149, RefSeq:WP_014112627.1, SO:0001217, UniParc:UPI00022BA705, UniRef:UniRef100_G4NQW3, UniRef:UniRef50_O34927, UniRef:UniRef90_O34927
SO:0001217, UniRef:UniRef50_G4NQW4, UniRef:UniRef90_G4NQW4
SO:0001217, UniRef:UniRef50_UPI0035586684, UniRef:UniRef90_UPI0035586684
BlastRules:WP_003246629, EC:7.4.2.-, GO:0005524, GO:0005886, GO:0015424, GO:0016887, RefSeq:WP_014112630.1, SO:0001217, UniParc:UPI00022BA708, UniRef:UniRef100_G4NQW6, UniRef:UniRef50_P39456, UniI
BlastRules:WP_003234541, GO:0005886, GO:0015184, GO:0015811, GO:0043190, RefSeq:WP_014112631.1, SO:0001217, UniParc:UPI00022BA709, UniRef:UniRef100_A0A9Q4HHW8, UniRef:UniRef50_P42200, UniRef:Ur
BlastRules:WP_003246699, GO:0005886, GO:0006865, SO:0001217, UniRef:UniRef50_P42199, UniRef:UniRef90_P42199
BlastRules:WP_003246552, GO:0003677, GO:0003700, GO:0005829, GO:0006355, RefSeq:WP_014112633.1, SO:0001217, UniParc:UPI00022BA70B, UniRef:UniRef100_A0A2M8SXV0, UniRef:UniRef50_P94403, UniRef:Un
EC:2.5.1.129, GO:0003824, GO:0009056, GO:0009636, GO:0016831, GO:0106141, KEGG:K03186, RefSeq:WP_029316764.1, SO:0001217, UniParc:UPI000493E37D, UniRef:UniRef100_G4NQX0, UniRef:UniRef50_P94404,
COG:COG0043, COG:H, EC:4.1.1.-, EC:4.1.1.61, GO:0005737, GO:0005829, GO:0006744, GO:0008694, GO:0009056, GO:0009636, GO:0016831, GO:0018799, GO:0046872, KEGG:K01612, SO:0001217, UniRef:UniRef50_
BlastRules:WP_010886405, GO:0009056, GO:0009636, SO:0001217, UniRef:UniRef50_C0H3U9, UniRef:UniRef90_C0H3U9
RefSeq:WP_072183608.1, SO:0001217, UniParc:UPI0006A83299, UniRef:UniRef100_UPI0006A83299, UniRef:UniRef50_C0SPB5, UniRef:UniRef90_G4NQX3
SO:0001217, UniRef:UniRef50_A0A2X1B9U9, UniRef:UniRef90_A0A9Q4DNT9
GO:0005886, GO:0006857, GO:0022857, GO:0055085, GO:1904680, RefSeq:WP_087987708.1, SO:0001217, UniParc:UPI000B4426C1, UniRef:UniRef100_A0A9Q4E8K3, UniRef:UniRef50_P94408, UniRef:UniRef90_P94408
PFAM:PF12708.12, PFAM:PF13229.12
SO:0001217, UniRef:UniRef50_P94409, UniRef:UniRef90_P94409
SO:0001217, UniRef:UniRef50_G4NQX7
GO:0005886, GO:0009847, SO:0001217, UniRef:UniRef50_P49939, UniRef:UniRef90_P49939
SO:0001217, UniRef:UniRef50_A0A7V9YXX8
GO:0005886, GO:0009847, KEGG:K06296, SO:0001217, UniRef:UniRef50_P49940, UniRef:UniRef90_P49940
GO:0005524, GO:0005886, GO:0016887, GO:0022857, GO:0055085, RefSeq:WP_088680296.1, SO:0001217, UniParc:UPI000B533D9C, UniRef:UniRef100_UPI000B533D9C, UniRef:UniRef50_P94411, UniRef:UniRef90_P94
SO:0001217, UniRef:UniRef50_W7AR24
GO:0000156, GO:0000976, GO:0005829, GO:0006355, GO:0032993, SO:0001217, UniRef:UniRef50_P94413, UniRef:UniRef90_P94413
BlastRules:WP_009969263, EC:2.7.13.3, GO:0000155, GO:0000156, GO:0005524, GO:0005886, GO:0007234, GO:0030295, SO:0001217, UniParc:UPI00022BA88C, UniRef:UniRef100_G4NQY4, UniRef:UniRef50_P94414, U
EC:3.1.-.-, GO:0005737, GO:0030420, KEGG:K06361, RefSeq:WP_014112649.1, SO:0001217, UniParc:UPI00022BA88D, UniRef:UniRef100_G4NQY5, UniRef:UniRef50_P71002, UniRef:UniRef90_P94415
BlastRules:WP_003224994, GO:0005576, GO:0005737, GO:0009372, GO:0030420, GO:0030435, GO:0043657, KEGG:K06353, RefSeq:WP_003224994.1, SO:0001217, UniParc:UPI000005FF1E, UniRef:UniRef100_P94416,
RefSeq:WP_041520857.1, SO:0001217, UniParc:UPI0005A1F395, UniRef:UniRef100_A0A9Q4E4F6, UniRef:UniRef50_A0A2M8SXW7, UniRef:UniRef90_A0A9Q4DM64
EC:2.7.2.4, GO:0004072, GO:0005524, GO:0005829, GO:0009088, GO:0009089, GO:0009090, GO:0019877, SO:0001217, UniRef:UniRef50_P94417, UniRef:UniRef90_P94417
BlastRules:WP_003234491, GO:0005886, GO:0022857, GO:0033214, SO:0001217, UniRef:UniRef50_P94418, UniRef:UniRef90_P94418
BlastRules:WP_003246705, GO:0005886, GO:0022857, GO:0033214, SO:0001217, UniRef:UniRef50_P94419, UniRef:UniRef90_P94419
EC:7.2.2.-, GO:0005524, GO:0005886, GO:0006826, GO:0016887, SO:0001217, UniRef:UniRef50_P94420, UniRef:UniRef90_P94420
BlastRules:WP_003246619, GO:0005886, GO:0030288, GO:0045121, GO:1901678, RefSeq:WP_014112654.1, SO:0001217, UniParc:UPI00022BA892, UniRef:UniRef100_G4NQZ1, UniRef:UniRef50_P94421, UniRef:UniRef9
SO:0001217, UniRef:UniRef50_A0A150F3U8, UniRef:UniRef90_A0A150F3U8
SO:0001217, UniRef:UniRef50_UPI0020C98DA0
SO:0001217, UniRef:UniRef50_A0A1Y3QPF4
GO:0003677, RefSeq:WP_072181729.1, SO:0001217, UniParc:UPI0006A8490B, UniRef:UniRef100_UPI0006A8490B, UniRef:UniRef50_P94423, UniRef:UniRef90_P94423
EC:1.5.1.39, GO:0008752, GO:0009056, GO:0009636, GO:0016491, GO:0052873, GO:0052874, RefSeq:WP_072181728.1, SO:0001217, UniParc:UPI0006A8AC97, UniRef:UniRef100_UPI0006A8AC97, UniRef:UniRef50_P94
EC:1.-.-.-, GO:0003824, GO:0004497, GO:0009056, GO:0009636, RefSeq:WP_003240208.1, SO:0001217, UniParc:UPI00022BA896, UniRef:UniRef100_A0A9W5PBZ3, UniRef:UniRef50_P94425, UniRef:UniRef90_P94425
GO:0003677, GO:0003700, GO:0006355, RefSeq:WP_014112658.1, SO:0001217, UniParc:UPI00022BA897, UniRef:UniRef100_G4NQZ6, UniRef:UniRef50_O31480, UniRef:UniRef90_O31480
GO:0003677, GO:0003700, GO:0008483, GO:0009058, GO:0030170, KEGG:K00375, SO:0001217, UniRef:UniRef50_P94426, UniRef:UniRef90_P94426
EC:2.6.1.19, GO:0005829, GO:0009450, GO:0030170, GO:0034386, GO:0047298, SO:0001217, UniRef:UniRef50_P94427, UniRef:UniRef90_P94427
SO:0001217, UniRef:UniRef50_A0A380FP33, UniRef:UniRef90_UPI002E20666E
BlastRules:WP_000350855, BlastRules:WP_003234455, GO:0005886, GO:0015144, GO:0030435, KEGG:K05340, RefSeq:WP_014112663.1, SO:0001217, UniParc:UPI00022BA89C, UniRef:UniRef100_G4NTV6, UniRef:UniR
BlastRules:WP_003246720, EC:1.1.1.47, GO:0016616, GO:0030435, GO:0047934, GO:0047935, SO:0001217, UniRef:UniRef50_P39485, UniRef:UniRef90_P12310
GO:0005886, RefSeq:WP_014112665.1, SO:0001217, UniParc:UPI00022BA89E, UniRef:UniRef100_G4NTV8, UniRef:UniRef50_P94431, UniRef:UniRef90_P94431
GO:0005507, GO:0005886, GO:0006825, GO:0042597, GO:0046688, KEGG:K14166, SO:0001217, UniRef:UniRef50_C0SP95, UniRef:UniRef90_C0SP95
BlastRules:WP_003246724, GO:0003677, GO:0003700, GO:0005737, GO:0046872, RefSeq:WP_072181725.1, SO:0001217, UniParc:UPI0006A80FF9, UniRef:UniRef100_UPI0006A80FF9, UniRef:UniRef50_P94433, UniRef:U
GO:0005886, RefSeq:WP_088680032.1, SO:0001217, UniParc:UPI000B52EA1F, UniRef:UniRef100_A0A9Q4E651, UniRef:UniRef50_P94434, UniRef:UniRef90_P94434
EC:2.7.1.197, KEGG:K02800, SO:0001217, UniRef:UniRef50_P50852, UniRef:UniRef90_A0AAP3FUC9
GO:0005737, GO:0005886, GO:0009401, GO:0016301, GO:0090563, RefSeq:WP_014112670.1, SO:0001217, UniParc:UPI00022BA8A3, UniRef:UniRef100_G4NTW3, UniRef:UniRef50_C0H3V2, UniRef:UniRef90_C0H3V2
EC:1.1.1.17, GO:0005829, GO:0008926, GO:0019592, GO:0019594, KEGG:K00009, RefSeq:WP_014112671.1, SO:0001217, UniParc:UPI00022BA8A4, UniRef:UniRef100_G4NTW4, UniRef:UniRef50_P42957, UniRef:UniRef9
EC:1.1.1.93, EC:4.1.1.73, GO:0000287, GO:0009027, GO:0046553, GO:0050319, GO:0051287, KEGG:K07246, SO:0001217, UniRef:UniRef50_P42958, UniRef:UniRef90_P42958
SO:0001217, UniRef:UniRef50_O31482, UniRef:UniRef90_G4NTW6
EC:4.2.1.59, KEGG:K02372, SO:0001217, UniRef:UniRef50_P42961, UniRef:UniRef90_A0AAP2M1U8
RefSeq:WP_061186203.1, SO:0001217, UniParc:UPI0006A846CA, UniRef:UniRef100_UPI0006A846CA, UniRef:UniRef50_C0H3V3, UniRef:UniRef90_C0H3V3
EC:3.1.3.104, EC:3.1.3.18, GO:0000287, GO:0005829, GO:0008967, GO:0009231, GO:0016791, GO:0043726, KEGG:K21064, SO:0001217, UniRef:UniRef50_P42962, UniRef:UniRef90_P42962
EC:3.1.3.104, EC:3.1.3.18, GO:0000287, GO:0005829, GO:0008967, GO:0009231, GO:0016791, GO:0043726, KEGG:K21064, SO:0001217, UniRef:UniRef50_P42962, UniRef:UniRef90_P42962
BlastRules:WP_003234423, EC:3.5.2.9, GO:0005524, GO:0005975, GO:0017168, KEGG:K07160, RefSeq:WP_014112676.1, SO:0001217, UniParc:UPI00022BAA08, UniRef:UniRef100_G4NTX0, UniRef:UniRef50_P42963, Ur
GO:0005384, GO:0005886, GO:0006828, GO:0015086, GO:0034755, SO:0001217, UniRef:UniRef50_P42964, UniRef:UniRef90_P42964
EC:4.2.1.-, GO:0003824, GO:0016829, RefSeq:WP_014112678.1, SO:0001217, UniParc:UPI00022BAA0A, UniRef:UniRef100_G4NTX2, UniRef:UniRef50_P42966, UniRef:UniRef90_P42966
BlastRules:WP_003234420, EC:3.5.2.9, GO:0004860, GO:0005524, GO:0005975, GO:0008152, GO:0017168, GO:0030435, SO:0001217, UniRef:UniRef50_P60495, UniRef:UniRef90_P60495
EC:3.5.2.9, GO:0005524, GO:0017168, GO:0030435, KEGG:K06350, RefSeq:WP_090557481.1, SO:0001217, UniParc:UPI000B868C46, UniRef:UniRef100_UPI000B868C46, UniRef:UniRef50_Q7WY77, UniRef:UniRef90_Q7\
GO:0003677, GO:0003700, GO:0045892, RefSeq:WP_061186210.1, SO:0001217, UniParc:UPI0006A8C56C, UniRef:UniRef100_A0A9Q4E8N6, UniRef:UniRef50_P42968, UniRef:UniRef90_P42968
EC:3.-.-.-, GO:0004622, GO:0016042, GO:0030435, SO:0001217, UniRef:UniRef50_P42969, UniRef:UniRef90_P42969
GO:0005886, RefSeq:WP_014112683.1, SO:0001217, UniParc:UPI00022BAA0F, UniRef:UniRef100_G4NTX7, UniRef:UniRef50_P42970, UniRef:UniRef90_P42970
RefSeq:WP_019257494.1, SO:0001217, UniParc:UPI0002DEF167, UniRef:UniRef100_G4NTX8, UniRef:UniRef50_A0A9P1JE27, UniRef:UniRef90_G4NTX8
BlastRules:WP_003246590, EC:3.4.16.4, GO:0005886, GO:0006508, GO:0008360, GO:0008658, GO:0009002, GO:0009252, GO:0045121, GO:0046677, GO:0071555, GO:0071972, KEGG:K21467, SO:0001217, UniRef:UniR
EC:1.-.-.-, GO:0005829, GO:0016491, KEGG:K26057, RefSeq:WP_014112686.1, SO:0001217, UniParc:UPI00022BAA12, UniRef:UniRef100_G4NTY0, UniRef:UniRef50_P42972, UniRef:UniRef90_P42972
BlastRules:WP_003246695, GO:0003677, GO:0006355, GO:0008982, GO:0009401, GO:0016301, KEGG:K03483, SO:0001217, UniRef:UniRef50_P96574, UniRef:UniRef90_P96574
EC:6.2.1.-, GO:0005524, GO:0016877, SO:0001217, UniRef:UniRef50_P96575, UniRef:UniRef90_P96575
RefSeq:WP_014112689.1, SO:0001217, UniParc:UPI00022BAA15, UniRef:UniRef100_G4NTY3, UniRef:UniRef50_P96576, UniRef:UniRef90_A0A9W5LGC7
EC:1.-.-.-, GO:0016614, RefSeq:WP_014112690.1, SO:0001217, UniParc:UPI00022BAA16, UniRef:UniRef100_G4NTY4, UniRef:UniRef50_P80873, UniRef:UniRef90_P80873
BlastRules:WP_003234400, EC:5.3.1.15, GO:0046872, GO:0047828, RefSeq:WP_079288478.1, SO:0001217, UniParc:UPI00086F8A90, UniRef:UniRef100_G4NTY5, UniRef:UniRef50_A3E7Z6, UniRef:UniRef90_P96578
RefSeq:WP_014112692.1, SO:0001217, UniParc:UPI00022BAA18, UniRef:UniRef100_G4NTY6, UniRef:UniRef50_P96579, UniRef:UniRef90_G4NTY6
RefSeq:WP_061186218.1, SO:0001217, UniParc:UPI0006A92178, UniRef:UniRef100_A0A9Q4E4I8, UniRef:UniRef50_P80238, UniRef:UniRef90_A0A9Q4E4I8
RFAM:RF01410, SO:0000655
BlastRules:WP_003234394, GO:0005886, GO:0008360, GO:0009252, GO:0015648, GO:0071555, SO:0001217, UniRef:UniRef50_P96581, UniRef:UniRef90_P96581
GO:0005886, RefSeq:WP_072565758.1, SO:0001217, UniParc:UPI00086958FC, UniRef:UniRef100_A0A9Q4E5U4, UniRef:UniRef50_O31485, UniRef:UniRef90_O31485
BlastRules:WP_003246585, GO:0005829, GO:0043200, GO:0043565, RefSeq:WP_072181712.1, SO:0001217, UniParc:UPI0006A90492, UniRef:UniRef100_A0A9Q4E8P6, UniRef:UniRef50_P96582, UniRef:UniRef90_P96582
EC:5.6.2.1, KEGG:K03169, SO:0001217, UniRef:UniRef50_Q65N90, UniRef:UniRef90_UPI00123BF36C
GO:0005886, GO:0005975, RefSeq:WP_072565759.1, SO:0001217, UniParc:UPI00086F824B, UniRef:UniRef100_UPI00086F824B, UniRef:UniRef50_O31486, UniRef:UniRef90_O31486
BlastRules:WP_003246541, GO:0005886, SO:0001217, UniRef:UniRef50_P96585, UniRef:UniRef90_P96585
GO:0005886, SO:0001217, UniRef:UniRef50_O31487, UniRef:UniRef90_O31487

SO:0001217, UniRef:UniRef50_A0A8J2TS31
GO:0005886, GO:0006011, SO:0001217, UniRef:UniRef50_O31488, UniRef:UniRef90_O31488
RFAM:RF00379, SO:0000140
BlastRules:WP_003246693, SO:0001217, UniRef:UniRef50_P96589
PFAM:PF00293.34
EC:1.2.3.3, GO:0000287, GO:0003824, GO:0030976, KEGG:K00158, SO:0001217, UniRef:UniRef50_P96591, UniRef:UniRef90_P96591
SO:0001217, UniRef:UniRef50_G4NU00, UniRef:UniRef90_G4NU00
GO:0005886, SO:0001217, UniRef:UniRef50_C0H3V4, UniRef:UniRef90_C0H3V4
GO:0005384, GO:0005886, GO:0006828, GO:0015086, GO:0015293, GO:0016020, GO:0022857, GO:0034755, GO:0046872, GO:0055085, SO:0001217, UniRef:UniRef50_P96593, UniRef:UniRef90_P96593
GO:0005886, RefSeq:WP_014112708.1, SO:0001217, UniParc:UPI00022BAA28, UniRef:UniRef100_G4NU03, UniRef:UniRef50_P96594, UniRef:UniRef90_P96594
SO:0001217, UniRef:UniRef50_P96595, UniRef:UniRef90_E0U352
RefSeq:WP_090557300.1, SO:0001217, UniParc:UPI00086980C5, UniRef:UniRef100_UPI00086980C5, UniRef:UniRef50_P96596, UniRef:UniRef90_P96596
KEGG:K06884, RefSeq:WP_014112711.1, SO:0001217, UniParc:UPI00022BAB93, UniRef:UniRef100_G4NU06, UniRef:UniRef50_A0A544T8S0, UniRef:UniRef90_A0AAP3CG56
RefSeq:WP_072181701.1, SO:0001217, UniParc:UPI0006A93CED, UniRef:UniRef100_UPI0006A93CED, UniRef:UniRef50_P96597, UniRef:UniRef90_G4NU07
EC:1.11.1.6, GO:0004096, GO:0046872, KEGG:K07217, RefSeq:WP_014112714.1, SO:0001217, UniParc:UPI00022BAB96, UniRef:UniRef100_G4NU09, UniRef:UniRef50_A0A7R7ZDZ6, UniRef:UniRef90_A0A7R7ZDZ6
BlastRules:WP_003234350, GO:0005576, GO:0030288, GO:0055085, KEGG:K11688, RefSeq:WP_072181699.1, SO:0001217, UniParc:UPI0006A86795, UniRef:UniRef100_UPI0006A86795, UniRef:UniRef50_P96600, UniRef
BlastRules:WP_003234348, EC:2.7.13.3, GO:0000155, GO:0005524, GO:0005886, RefSeq:WP_061186236.1, SO:0001217, UniParc:UPI0006A8FBB0, UniRef:UniRef100_UPI0006A8FBB0, UniRef:UniRef50_P96601, UniRef:U
BlastRules:WP_003234347, GO:0000156, GO:0003677, GO:0003700, GO:0005737, RefSeq:WP_061186237.1, SO:0001217, UniParc:UPI0006A8479C, UniRef:UniRef100_A0A9Q4E7M7, UniRef:UniRef50_P96602, UniRef:Uni
BlastRules:WP_003246626, GO:0005886, GO:0015138, GO:0015141, GO:0015366, GO:0070778, KEGG:K11103, RefSeq:WP_003225140.1, SO:0001217, UniParc:UPI0001D28677, UniRef:UniRef100_A0A9Q4DKH8, UniRef:
GO:0005886, GO:0055085, RefSeq:WP_061186238.1, SO:0001217, UniParc:UPI0006A93C72, UniRef:UniRef100_UPI0006A93C72, UniRef:UniRef50_P96604, UniRef:UniRef90_P96604
RFAM:RF00379, SO:0000140
RefSeq:WP_014112719.1, SO:0001217, UniParc:UPI00022BAB9B, UniRef:UniRef100_G4NU15, UniRef:UniRef50_S2XF52, UniRef:UniRef90_G4NU15
GO:0005524, GO:0016887, RefSeq:WP_072181698.1, SO:0001217, UniParc:UPI0006A84153, UniRef:UniRef100_UPI0006A84153, UniRef:UniRef50_P96605, UniRef:UniRef90_P96605
GO:0005886, RefSeq:WP_014112721.1, SO:0001217, UniParc:UPI00022BAB9D, UniRef:UniRef100_G4NU17, UniRef:UniRef50_P96606, UniRef:UniRef90_P96606
GO:0005886, RefSeq:WP_014112722.1, SO:0001217, UniParc:UPI00022BAB9E, UniRef:UniRef100_G4NU18, UniRef:UniRef50_P96607, UniRef:UniRef90_P96607
EC:1.3.99.-, GO:0003995, GO:0050660, RefSeq:WP_242734749.1, SO:0001217, UniParc:UPI001F60DD78, UniRef:UniRef100_UPI001F60DD78, UniRef:UniRef50_P96608, UniRef:UniRef90_P96608
RefSeq:WP_003246512.1, SO:0001217, UniParc:UPI0001976137, UniRef:UniRef100_P96609, UniRef:UniRef50_P96609, UniRef:UniRef90_P96609
RefSeq:WP_003225157.1, SO:0001217, UniParc:UPI0001976138, UniRef:UniRef100_L8ECQ9, UniRef:UniRef50_A0A7W4LU85, UniRef:UniRef90_L8ECQ9
GO:0005886, GO:0008324, GO:0016020, KEGG:K13283, SO:0001217, UniRef:UniRef50_P96610, UniRef:UniRef90_P96610
GO:0005737, GO:0005829, GO:0015035, GO:0045454, RefSeq:WP_003225161.1, SO:0001217, UniParc:UPI0001D2867F, UniRef:UniRef100_A0A9Q4DKC2, UniRef:UniRef50_P96611, UniRef:UniRef90_P96611
EC:6.3.2.4, GO:0005524, GO:0005829, GO:0008360, GO:0008716, GO:0009252, GO:0046872, GO:0071555, RefSeq:WP_061186244.1, SO:0001217, UniParc:UPI0006A87BE2, UniRef:UniRef100_UPI0006A87BE2, UniRef:U
PFAM:PF01225.30
SO:0001217, UniRef:UniRef50_A0A5S9M6Y3
BlastRules:WP_003732374, BlastRules:WP_010886411, COG:COG0513, COG:L, EC:3.6.4.13, GO:0003689, GO:0003723, GO:0003724, GO:0005524, GO:0005829, GO:0005886, GO:0006401, GO:0009409, GO:0010501, GO
GO:0005886, RefSeq:WP_372402453.1, SO:0001217, UniParc:UPI003558F152, UniRef:UniRef100_UPI003558F152, UniRef:UniRef50_P96615, UniRef:UniRef90_P96615
GO:0005886, KEGG:K08981, RefSeq:WP_041520861.1, SO:0001217, UniParc:UPI0005A183DB, UniRef:UniRef100_G4NU28, UniRef:UniRef50_P96616, UniRef:UniRef90_P96616
EC:3.4.21.-, GO:0004252, GO:0005886, GO:0006508, RefSeq:WP_003225175.1, SO:0001217, UniParc:UPI0001D28685, UniRef:UniRef100_A0A9Q4H8D6, UniRef:UniRef50_P96617, UniRef:UniRef90_P96617
EC:2.7.8.7, GO:0000287, GO:0005829, GO:0006633, GO:0008897, GO:0019878, RefSeq:WP_014112730.1, SO:0001217, UniParc:UPI00022BABA6, UniRef:UniRef100_G4NU30, UniRef:UniRef50_P96618, UniRef:UniRef90_
GO:0005886, GO:0030435, RefSeq:WP_072181690.1, SO:0001217, UniParc:UPI0006A84871, UniRef:UniRef100_A0A9Q4E3V5, UniRef:UniRef50_P96619, UniRef:UniRef90_P96619
EC:5.1.1.1, GO:0006522, GO:0008784, GO:0009252, KEGG:K01775, SO:0001217, UniRef:UniRef50_P10725, UniRef:UniRef90_A0A6H0WS64
BlastRules:WP_003225183, GO:0006355, GO:0042802, RefSeq:WP_003225183.1, SO:0001217, UniParc:UPI000005FF73, UniRef:UniRef100_P96621, UniRef:UniRef50_P96621, UniRef:UniRef90_P96621
BlastRules:WP_000635963, BlastRules:WP_003156187, COG:COG2337, COG:V, EC:3.1.27.-, GO:0003677, GO:0003723, GO:0004521, GO:0006402, GO:0016075, RefSeq:WP_003156187.1, SO:0001217, UniParc:UPI00000
BlastRules:WP_009966610, GO:0019825, GO:0020037, SO:0001217, UniParc:UPI00022BABA8, UniRef:UniRef100_A0A2M8SY61, UniRef:UniRef50_P42409, UniRef:UniRef90_P42409
BlastRules:WP_003225190, COG:COG1366, COG:T, RefSeq:WP_003225190.1, SO:0001217, UniParc:UPI000005FF76, UniRef:UniRef100_P42410, UniRef:UniRef50_P42410, UniRef:UniRef90_P42410
BlastRules:WP_003246640, EC:2.7.11.1, GO:0004676, GO:0004677, GO:0004679, GO:0004711, GO:0005524, GO:0035175, GO:0035402, GO:0035403, GO:0035979, GO:0044022, GO:0044023, GO:0044024, GO:0044025,
BlastRules:WP_003234295, EC:3.1.3.3, GO:0004721, GO:0016791, GO:0036424, RefSeq:WP_003225194.1, SO:0001217, UniParc:UPI0001D2868B, UniRef:UniRef100_A0A9Q4DKC3, UniRef:UniRef50_P40399, UniRef:UniR
GO:0043856, RefSeq:WP_003225196.1, SO:0001217, UniParc:UPI0001D2868C, UniRef:UniRef100_A0A9Q4HGF8, UniRef:UniRef50_P17903, UniRef:UniRef90_P17903
BlastRules:WP_000970578, BlastRules:WP_003234299, BlastRules:WP_003417160, BlastRules:WP_003893193, EC:2.7.11.1, GO:0004674, GO:0004676, GO:0004677, GO:0004679, GO:0004711, GO:0005524, GO:0006468
GO:0003677, GO:0003700, GO:0006352, GO:0006355, GO:0016987, KEGG:K03090, RefSeq:WP_003240361.1, SO:0001217, UniParc:UPI00022BABAC, UniRef:UniRef100_A0A2M8SY84, UniRef:UniRef50_P06574, UniRef:U
BlastRules:WP_003246608, EC:3.1.3.3, GO:0004721, GO:0009408, GO:0036424, KEGG:K05518, RefSeq:WP_072181688.1, SO:0001217, UniParc:UPI0006A8A6DF, UniRef:UniRef100_UPI0006A8A6DF, UniRef:UniRef50_P17
SO:0001217, UniRef:UniRef50_A0A941FIQ9
SO:0001217, UniRef:UniRef50_A0A5S9M2V8
GO:0030435, GO:0042763, RefSeq:WP_003225207.1, SO:0001217, UniParc:UPI0001976122, UniRef:UniRef100_A0A2K4Z9G8, UniRef:UniRef50_A0A2K4Z9G8, UniRef:UniRef90_A0A2K4Z9G8
GO:0005737, GO:0008270, GO:0033554, RefSeq:WP_014112738.1, SO:0001217, UniParc:UPI00022BABAF, UniRef:UniRef100_G4NW64, UniRef:UniRef50_P96628, UniRef:UniRef90_P96628
SO:0000257
SO:0000269
SO:0000259
SO:0000261
SO:0000265
SO:0000264
SO:0000264
RefSeq:WP_041520862.1, SO:0001217, UniParc:UPI00059F3690, UniRef:UniRef100_A0A9Q4ES81, UniRef:UniRef50_W8YYL1, UniRef:UniRef90_G4NW66
RefSeq:WP_014112741.1, SO:0001217, UniParc:UPI00022BABB2, UniRef:UniRef100_G4NW67, UniRef:UniRef50_Q49WS9, UniRef:UniRef90_G4NW67
RefSeq:WP_014112742.1, SO:0001217, UniParc:UPI00022BABB3, UniRef:UniRef100_G4NW68, UniRef:UniRef50_D4G5L7, UniRef:UniRef90_G4NW68
RefSeq:WP_014112743.1, SO:0001217, UniParc:UPI00022BABB4, UniRef:UniRef100_G4NW69, UniRef:UniRef50_A0A9P3BC58, UniRef:UniRef90_A0A9X8RA88
RefSeq:WP_014112744.1, SO:0001217, UniParc:UPI00022BAD30, UniRef:UniRef100_G4NW70, UniRef:UniRef50_A0A5J4JDJ4, UniRef:UniRef90_D4G5L9
KEGG:K21488, SO:0001217, UniRef:UniRef50_G4NW71, UniRef:UniRef50_M5P5N0, UniRef:UniRef90_A0AAP1H9E2, UniRef:UniRef90_G4NW71
SO:0001217, UniRef:UniRef50_G4NW72
KEGG:K16263, RefSeq:WP_014112747.1, SO:0001217, UniParc:UPI00022BAD33, UniRef:UniRef100_G4NW73, UniRef:UniRef50_R8D660, UniRef:UniRef90_G4NW73
RefSeq:WP_014112748.1, SO:0001217, UniParc:UPI00022BAD34, UniRef:UniRef100_G4NW74, UniRef:UniRef50_A0A841Q5S0, UniRef:UniRef90_G4NW74
RefSeq:WP_014112749.1, SO:0001217, UniParc:UPI00022BAD35, UniRef:UniRef100_G4NW75, UniRef:UniRef50_I2C1P2, UniRef:UniRef90_G4NW75
RefSeq:WP_014112750.1, SO:0001217, UniParc:UPI00022BAD36, UniRef:UniRef100_G4NW76, UniRef:UniRef50_D5E1T6, UniRef:UniRef90_G4NW76
RefSeq:WP_014112751.1, SO:0001217, UniParc:UPI00022BAD37, UniRef:UniRef100_G4NW77, UniRef:UniRef50_R2QTX4, UniRef:UniRef90_G4NW77
GO:0005829, GO:0016705, RefSeq:WP_014112752.1, SO:0001217, UniParc:UPI00022BAD38, UniRef:UniRef100_G4NW78, UniRef:UniRef50_P96651, UniRef:UniRef90_P96651
SO:0001217, UniRef:UniRef50_A0A1S8TW38, UniRef:UniRef90_G4NW79
EC:3.-.-.-, GO:0016787, RefSeq:WP_014112754.1, SO:0001217, UniParc:UPI00022BAD3A, UniRef:UniRef100_G4NW80, UniRef:UniRef50_P96655, UniRef:UniRef90_P96655
GO:0005886, GO:0022857, RefSeq:WP_014112755.1, SO:0001217, UniParc:UPI00022BAD3B, UniRef:UniRef100_G4NW81, UniRef:UniRef50_P96656, UniRef:UniRef90_P96656
RefSeq:WP_014112756.1, SO:0001217, UniParc:UPI00022BAD3C, UniRef:UniRef100_G4NW82, UniRef:UniRef50_A0A6A8LIN2, UniRef:UniRef90_G4NW82
SO:0001217, UniParc:UPI00022BAD3D, UniRef:UniRef100_G4NW83, UniRef:UniRef50_Q24QY4, UniRef:UniRef90_G4NW83
RefSeq:WP_014112758.1, SO:0001217, UniParc:UPI00022BAD3E, UniRef:UniRef100_G4NW84, UniRef:UniRef50_Q81DJ6, UniRef:UniRef90_G4NW84
RefSeq:WP_014112759.1, SO:0001217, UniParc:UPI00022BAD3F, UniRef:UniRef100_G4NW85, UniRef:UniRef50_W7L834, UniRef:UniRef90_G4NW85
KEGG:K05835, RefSeq:WP_014112760.1, SO:0001217, UniParc:UPI00022BAD40, UniRef:UniRef100_G4NW86, UniRef:UniRef50_G4NW86, UniRef:UniRef90_G4NW86
COG:COG3759, COG:S, KEGG:K08987, RefSeq:WP_014112761.1, SO:0001217, UniParc:UPI00022BAD41, UniRef:UniRef100_G4NW87, UniRef:UniRef50_B1YKF7, UniRef:UniRef90_A0A0G8F2L2
COG:COG3239, COG:I, EC:1.14.19.23, EC:1.14.19.45, KEGG:K10255, RefSeq:WP_072183283.1, SO:0001217, UniParc:UPI0006A89503, UniRef:UniRef100_UPI0006A89503, UniRef:UniRef50_O34653, UniRef:UniRef90_A0A
RFAM:RF01766, SO:0002168
BlastRules:WP_001990088, BlastRules:WP_003234247, COG:COG1278, COG:K, GO:0003676, GO:0003677, GO:0005737, GO:0010468, KEGG:K03704, RefSeq:WP_013351108.1, SO:0001217, UniParc:UPI0001E39E70, Ur
RFAM:RF01410, SO:0000655
GO:0009303, RefSeq:WP_041521131.1, SO:0001217, UniParc:UPI00059F4A36, UniRef:UniRef100_G4NW91, UniRef:UniRef50_P96659, UniRef:UniRef90_P96659
KEGG:K13653, SO:0001217, UniRef:UniRef50_A0A2W0CAU9, UniRef:UniRef90_UPI0030D0FDC5
RefSeq:WP_072183276.1, SO:0001217, UniParc:UPI0006A93997, UniRef:UniRef100_UPI0006A93997, UniRef:UniRef50_P96665, UniRef:UniRef90_UPI0006A93997
RefSeq:WP_072183275.1, SO:0001217, UniParc:UPI0006A89C1D, UniRef:UniRef100_UPI0006A89C1D, UniRef:UniRef50_P96666, UniRef:UniRef90_A0A0T6BSC9
SO:0001217, UniRef:UniRef50_G4NWA0, UniRef:UniRef90_G4NWA0
RefSeq:WP_165762157.1, SO:0001217, UniParc:UPI0006A83D9D, UniRef:UniRef100_A0A9Q4HHY1, UniRef:UniRef50_A0AAP3CIW4, UniRef:UniRef90_A0AAP3CIW4
SO:0001217, UniRef:UniRef50_A0A5D4N7R7
SO:0001217, UniRef:UniRef50_P96667, UniRef:UniRef90_UPI0035584DE6
SO:0001217, UniRef:UniRef50_A0A9W4KZ64, UniRef:UniRef90_G4NWA3
RefSeq:WP_014112777.1, SO:0001217, UniParc:UPI00022BAD51, UniRef:UniRef100_G4NWA4, UniRef:UniRef50_D4G5L7, UniRef:UniRef90_D4G5L7
SO:0001217, UniRef:UniRef50_UPI0011542A14
SO:0001217, UniRef:UniRef50_UPI002E206760
RefSeq:WP_061186267.1, SO:0001217, UniParc:UPI000772A488, UniRef:UniRef100_A0A9Q4E619, UniRef:UniRef50_P96655, UniRef:UniRef90_A0AAE2EMD9
RefSeq:WP_003225257.1, SO:0001217, UniParc:UPI0001D2877C, UniRef:UniRef100_A0A9Q4H7Q2, UniRef:UniRef50_U1LJK6, UniRef:UniRef90_G4NWA8
GO:0005886, RefSeq:WP_014112782.1, SO:0001217, UniParc:UPI00022BAD56, UniRef:UniRef100_G4NWA9, UniRef:UniRef50_P96668, UniRef:UniRef90_P96668
KEGG:K00375, SO:0001217, UniRef:UniRef50_P96669, UniRef:UniRef90_UPI0015771403
RefSeq:WP_072183269.1, SO:0001217, UniParc:UPI0006A801B9, UniRef:UniRef100_UPI0006A801B9, UniRef:UniRef50_P96670, UniRef:UniRef90_P96670
EC:3.-.-.-, GO:0016787, RefSeq:WP_072183268.1, SO:0001217, UniParc:UPI0006A92B2F, UniRef:UniRef100_UPI0006A92B2F, UniRef:UniRef50_P96671, UniRef:UniRef90_P96671
GO:0003677, RefSeq:WP_072183267.1, SO:0001217, UniParc:UPI0006A8DE42, UniRef:UniRef100_UPI0006A8DE42, UniRef:UniRef50_O31494, UniRef:UniRef90_O31494
COG:COG1284, COG:S, GO:0005886, RefSeq:WP_014112786.1, SO:0001217, UniParc:UPI00022BAD5A, UniRef:UniRef100_G4NWB3, UniRef:UniRef50_P96672, UniRef:UniRef90_P96672
RefSeq:WP_072183266.1, SO:0001217, UniParc:UPI00092ED063, UniRef:UniRef100_UPI00092ED063, UniRef:UniRef50_G4NWB4, UniRef:UniRef90_G4NWB4
EC:1.6.99.-, GO:0003955, GO:0009055, GO:0010181, SO:0001217, UniRef:UniRef50_P96674, UniRef:UniRef90_P96674
SO:0001217, UniRef:UniRef50_P96677, UniRef:UniRef90_E0U4C6
GO:0005886, GO:0015105, GO:0046685, KEGG:K03893, RefSeq:WP_087987652.1, SO:0001217, UniParc:UPI000B43DFAF, UniRef:UniRef100_UPI000B43DFAF, UniRef:UniRef50_Q5HF02, UniRef:UniRef90_P96678
EC:1.20.4.4, GO:0004725, GO:0030612, GO:0046685, KEGG:K03741, RefSeq:WP_014112792.1, SO:0001217, UniParc:UPI00022BAD60, UniRef:UniRef100_G4NWB9, UniRef:UniRef50_P45947, UniRef:UniRef90_G4NWB9
RefSeq:WP_072183096.1, SO:0001217, UniParc:UPI0006A86070, UniRef:UniRef100_UPI0006A86070, UniRef:UniRef50_P96679, UniRef:UniRef90_G4NWC0
EC:2.7.8.-, KEGG:K01005, RefSeq:WP_080376114.1, SO:0001217, UniParc:UPI0006A896A9, UniRef:UniRef100_UPI0006A896A9, UniRef:UniRef50_G4NWC1, UniRef:UniRef90_G4NWC1
SO:0001217, UniRef:UniRef50_A0A7Y8RUV8, UniRef:UniRef90_G4NWC2
SO:0001217, UniRef:UniRef50_Q62TD4, UniRef:UniRef90_G4NWC3
SO:0001217, UniRef:UniRef50_Q65HY4
SO:0001217, UniRef:UniRef50_A0A2T0B2F3, UniRef:UniRef90_G4NWC5
RefSeq:WP_061186276.1, SO:0001217, UniParc:UPI0006A8192B, UniRef:UniRef100_UPI0006A8192B, UniRef:UniRef50_A0A0M0L0N6, UniRef:UniRef90_E0U4D1
SO:0001217, UniRef:UniRef50_P96681, UniRef:UniRef90_A0A6H0WFV4
SO:0001217, UniRef:UniRef50_P96682, UniRef:UniRef90_A0A9W5PC46
SO:0001217, UniRef:UniRef50_A0A8J2W219
RefSeq:WP_014112803.1, SO:0001217, UniParc:UPI00022BAECC, UniRef:UniRef100_G4NR01, UniRef:UniRef50_P96684, UniRef:UniRef90_A0A2M8SYF3
SO:0001217, UniParc:UPI00022BAECD, UniRef:UniRef100_A0A9Q4E6E9, UniRef:UniRef50_C0H3W0, UniRef:UniRef90_A0A9Q4E6E9
COG:COG4585, COG:T, EC:2.7.13.3, KEGG:K11623, SO:0001217, UniParc:UPI00022BAECF, UniRef:UniRef100_G4NR04, UniRef:UniRef50_P96685, UniRef:UniRef90_Q65DI6
BlastRules:WP_003244318, COG:COG2197, COG:TK, KEGG:K11624, RefSeq:WP_014112807.1, SO:0001217, UniParc:UPI00022BAED0, UniRef:UniRef100_G4NR05, UniRef:UniRef50_G4NR05, UniRef:UniRef90_G4NR05
COG:COG2409, COG:R, GO:0005886, KEGG:K11625, SO:0001217, UniRef:UniRef50_P96687, UniRef:UniRef90_P96687
SO:0001217, UniRef:UniRef50_G4NR08, UniRef:UniRef90_G4NR08
SO:0001217, UniRef:UniRef50_E0U4E2, UniRef:UniRef90_E0U4E2
GO:0005304, GO:0005886, GO:0015188, GO:0015190, GO:0015658, GO:0015803, GO:0015818, GO:0015820, GO:0015829, GO:0016020, KEGG:K03311, RefSeq:WP_088680020.1, SO:0001217, UniParc:UPI000B52AF30,
KEGG:K26248, SO:0001217, UniRef:UniRef50_G4NR11, UniRef:UniRef90_G4NR11
GO:0005886, KEGG:K07150, RefSeq:WP_014112814.1, SO:0001217, UniParc:UPI00022BAED7, UniRef:UniRef100_G4NR12, UniRef:UniRef50_P96689, UniRef:UniRef90_P96689
KEGG:K19575, SO:0001217, UniRef:UniRef50_P96690, UniRef:UniRef90_A0AAP3CKG0
SO:0001217, UniRef:UniRef50_UPI00102E60D0, UniRef:UniRef90_UPI00102E60D0
BlastRules:WP_003244477, GO:0005886, GO:0008324, GO:0016020, KEGG:K13283, RefSeq:WP_072183084.1, SO:0001217, UniParc:UPI0006A849F8, UniRef:UniRef100_UPI0006A849F8, UniRef:UniRef50_C0SP78, UniRe
EC:1.-.-.-, GO:0005737, GO:0009056, GO:0009636, GO:0016491, RefSeq:WP_072183083.1, SO:0001217, UniParc:UPI0006A82757, UniRef:UniRef100_UPI0006A82757, UniRef:UniRef50_P96692, UniRef:UniRef90_P96692
EC:1.13.11.-, GO:0005737, GO:0009056, GO:0009636, GO:0046872, GO:0051213, SO:0001217, UniRef:UniRef50_P96693, UniRef:UniRef90_P96693

EC:1.-.-.-, GO:0005886, GO:0009056, GO:0009636, GO:0016491, RefSeq:WP_010333166.1, SO:0001217, UniParc:UPI00022BAEDC, UniRef:UniRef100_G4NR17, UniRef:UniRef50_P96694, UniRef:UniRef90_P96694
RefSeq:WP_014112819.1, SO:0001217, UniParc:UPI00022BAEDD, UniRef:UniRef100_G4NR18, UniRef:UniRef50_P96695, UniRef:UniRef90_P96695
RefSeq:WP_326198124.1, SO:0001217, UniParc:UPI002DC001C6, UniRef:UniRef100_UPI002DC001C6, UniRef:UniRef50_A0A0M0LKH4, UniRef:UniRef90_G4NR21
RefSeq:WP_326198125.1, SO:0001217, UniParc:UPI002DC008A3, UniRef:UniRef100_UPI002DC008A3, UniRef:UniRef50_F9DSI5, UniRef:UniRef90_A0A9Q4DKU3
RefSeq:WP_061186288.1, SO:0001217, UniParc:UPI00077239FA, UniRef:UniRef100_UPI00077239FA, UniRef:UniRef50_A0A9Q4E9G2, UniRef:UniRef90_A0A9Q4E9G2
GO:0006457, GO:0009408, GO:0009651, GO:0030435, GO:0042542, GO:0051082, GO:0051259, RefSeq:WP_072183078.1, SO:0001217, UniParc:UPI0006A8A109, UniRef:UniRef100_UPI0006A8A109, UniRef:UniRef50_P96
RefSeq:WP_014112826.1, SO:0001217, UniParc:UPI00022BAEE4, UniRef:UniRef100_G4NR25, UniRef:UniRef50_P96699, UniRef:UniRef90_G4NR25
RefSeq:WP_041520868.1, SO:0001217, UniParc:UPI0005A2CC3A, UniRef:UniRef100_UPI0005A2CC3A, UniRef:UniRef50_P96700, UniRef:UniRef90_UPI0005A2CC3A
RefSeq:WP_072183077.1, SO:0001217, UniParc:UPI0006A83C72, UniRef:UniRef100_UPI0006A83C72, UniRef:UniRef50_P96701, UniRef:UniRef90_G4NR27
RefSeq:WP_072183076.1, SO:0001217, UniParc:UPI0006A8B2B1, UniRef:UniRef100_UPI0006A8B2B1, UniRef:UniRef50_P96702, UniRef:UniRef90_E0U4G1
RefSeq:WP_072183075.1, SO:0001217, UniParc:UPI0006A81A4B, UniRef:UniRef100_UPI0006A81A4B, UniRef:UniRef50_P96703, UniRef:UniRef90_G4NR29
RefSeq:WP_087987715.1, SO:0001217, UniParc:UPI0006A8C36C, UniRef:UniRef100_UPI000772BB1D, UniRef:UniRef50_A4C3V1, UniRef:UniRef90_A0A5D4N0A8
RefSeq:WP_072183074.1, SO:0001217, UniParc:UPI0006A8CBD2, UniRef:UniRef100_UPI0006A8CBD2, UniRef:UniRef50_A0A3M3ESH5, UniRef:UniRef90_G4NR30
RefSeq:WP_014112832.1, SO:0001217, UniParc:UPI00022BAEEA, UniRef:UniRef100_G4NR31, UniRef:UniRef50_A0A0A1MT41, UniRef:UniRef90_G4NR31
SO:0001217, UniRef:UniRef50_A0A0H2VG78, UniRef:UniRef90_G4NR32
SO:0001217, UniRef:UniRef50_UPI001F4A00BB
RefSeq:WP_072183072.1, SO:0001217, UniParc:UPI0006A81304, UniRef:UniRef100_UPI0006A81304, UniRef:UniRef50_G4NR33, UniRef:UniRef90_G4NR33
SO:0001217, UniRef:UniRef50_A0A8B5Y824
RefSeq:WP_061186297.1, SO:0001217, UniParc:UPI0006A8DDD4, UniRef:UniRef100_A0A9Q4E967, UniRef:UniRef50_G4NR35, UniRef:UniRef90_G4NR35
BlastRules:WP_003234144, GO:0003677, GO:0005524, GO:0016887, NCBIProtein:WP_003234144.1, SO:0001217, UniRef:UniRef50_P39115, UniRef:UniRef90_A0AAP3CIW7
RFAM:RF02452, SO:0000655
GO:0005886, GO:0006865, GO:0055085, RefSeq:WP_003225371.1, SO:0001217, UniParc:UPI0001D28870, UniRef:UniRef100_A0A9Q4DKY6, UniRef:UniRef50_P96704, UniRef:UniRef90_P96704
BlastRules:WP_003234141, SO:0001217, UniRef:UniRef50_Q02886, UniRef:UniRef90_UPI0002E4B9F9
GO:0003677, GO:0003700, GO:0006355, GO:0006950, SO:0001217, UniRef:UniRef50_P96705, UniRef:UniRef90_P96705
GO:0005886, SO:0001217, UniRef:UniRef50_P96706, UniRef:UniRef90_P96706
SO:0001217, UniRef:UniRef50_A0A5S9M9H5
EC:1.-.-.-, GO:0016491, RefSeq:WP_014112842.1, SO:0001217, UniParc:UPI00022BB067, UniRef:UniRef100_G4NR41, UniRef:UniRef50_P96707, UniRef:UniRef90_P96707
GO:0003677, GO:0003700, SO:0001217, UniParc:UPI00022BB068, UniRef:UniRef100_G4NR42, UniRef:UniRef50_P96708, UniRef:UniRef90_P96708
RefSeq:WP_072565825.1, SO:0001217, UniParc:UPI00086D8B70, UniRef:UniRef100_UPI00086D8B70, UniRef:UniRef50_A0A2X4VYV6, UniRef:UniRef90_G4NR43
KEGG:K26605, SO:0001217, UniRef:UniRef50_A0A069DL18, UniRef:UniRef90_G4NR44
RefSeq:WP_014112846.1, SO:0001217, UniParc:UPI00022BB06B, UniRef:UniRef100_G4NR45, UniRef:UniRef50_R7ZHA5, UniRef:UniRef90_G4NR45
SO:0001217, UniRef:UniRef50_UPI002F919851
GO:0005886, RefSeq:WP_014112851.1, SO:0001217, UniParc:UPI00022BB070, UniRef:UniRef100_G4NR50, UniRef:UniRef50_O05493, UniRef:UniRef90_O05493
GO:0003700, GO:0043565, SO:0001217, UniRef:UniRef50_O05494, UniRef:UniRef90_O05494
EC:3.2.-.-, GO:0000272, GO:0008061, GO:0009313, GO:0016798, GO:0030435, GO:0031160, RefSeq:WP_090557237.1, SO:0001217, UniParc:UPI0008698003, UniRef:UniRef100_UPI0008698003, UniRef:UniRef50_O05495
EC:2.4.1.-, GO:0008194, GO:0009058, GO:0016758, RefSeq:WP_327816474.1, SO:0001217, UniParc:UPI002E232413, UniRef:UniRef100_UPI002E232413, UniRef:UniRef50_O05496, UniRef:UniRef90_O05496
RefSeq:WP_041520872.1, SO:0001217, UniParc:UPI0005A15461, UniRef:UniRef100_G4NR54, UniRef:UniRef50_O05497, UniRef:UniRef90_O05497
BlastRules:WP_003234119, EC:3.1.3.1, GO:0004035, GO:0046872, KEGG:K01077, RefSeq:WP_014112856.1, SO:0001217, UniParc:UPI00022BB075, UniRef:UniRef100_G4NR55, UniRef:UniRef50_P19405, UniRef:UniRef90
RefSeq:WP_072183058.1, SO:0001217, UniParc:UPI0006A8056F, UniRef:UniRef100_UPI0006A8056F, UniRef:UniRef50_W4R2A9, UniRef:UniRef90_G4NR56
SO:0001217, UniRef:UniRef50_O05500, UniRef:UniRef90_E0TTG5
SO:0001217, UniParc:UPI00022BB078, UniRef:UniRef100_G4NR58, UniRef:UniRef50_G4NR58, UniRef:UniRef90_G4NR58
SO:0001217, UniRef:UniRef50_O05502, UniRef:UniRef90_A0A2M8SYG1
SO:0001217, UniRef:UniRef50_O05503, UniRef:UniRef90_A0AAP3CLB4
BlastRules:WP_003234105, GO:0005886, GO:0022857, GO:0055085, KEGG:K18567, RefSeq:WP_014112862.1, SO:0001217, UniParc:UPI00022BB07B, UniRef:UniRef100_G4NR61, UniRef:UniRef50_Q797E3, UniRef:UniRe
RFAM:RF00167, SO:0000035
GO:0005737, GO:0008982, GO:0009401, GO:0015774, GO:0016301, RefSeq:WP_014112863.1, SO:0001217, UniParc:UPI00022BB07C, UniRef:UniRef100_G4NR62, UniRef:UniRef50_O05505, UniRef:UniRef90_O05505
GO:0005737, GO:0009401, GO:0015774, GO:0016301, RefSeq:WP_014112864.1, SO:0001217, UniParc:UPI00022BB07D, UniRef:UniRef100_G4NR63, UniRef:UniRef50_O05506, UniRef:UniRef90_O05506
EC:2.7.1.205, GO:0008982, GO:0009401, GO:0016020, GO:0019191, KEGG:K02761, SO:0001217, UniRef:UniRef50_T0TIR5, UniRef:UniRef90_A0A0D1L4M9
KEGG:K02761, SO:0001217, UniRef:UniRef50_D4G5T9, UniRef:UniRef90_D4G5T9
EC:3.2.1.86, GO:0000272, GO:0005829, GO:0008422, GO:0008706, GO:0016052, RefSeq:WP_072182137.1, SO:0001217, UniParc:UPI0006A81DDB, UniRef:UniRef100_UPI0006A81DDB, UniRef:UniRef50_O05508, UniRef:U
SO:0001217, UniRef:UniRef50_UPI0031F9FD7C
SO:0001217, UniRef:UniRef50_O05510, UniRef:UniRef90_A0A6H0WI01
EC:5.3.1.8, GO:0004476, GO:0005975, GO:0008270, SO:0001217, UniRef:UniRef50_O31646, UniRef:UniRef90_O05511
EC:3.2.1.78, GO:0000272, GO:0005576, GO:0006080, GO:0016985, SO:0001217, UniRef:UniRef50_O05512, UniRef:UniRef90_O05512
SO:0001217, UniRef:UniRef50_A0A1X9MAI7
PFAM:PF05067.18
SO:0001036
SO:0000260
RFAM:RF01690, SO:0000655
GO:0003735, GO:0005840, KEGG:K01977, RFAM:RF00177, SO:0001000
GO:0003735, GO:0005840, KEGG:K01980, RFAM:RF02541, SO:0001001
GO:0003735, GO:0005840, KEGG:K01985, RFAM:RF00001, SO:0000652
SO:0000266
SO:0000256
EC:2.7.4.16, GO:0000287, GO:0005524, GO:0009030, GO:0009228, GO:0009229, KEGG:K00946, RefSeq:WP_072181784.1, SO:0001217, UniParc:UPI0006A84AB0, UniRef:UniRef100_UPI0006A84AB0, UniRef:UniRef50_O0
GO:0002949, GO:0005524, GO:0005737, GO:0046872, RefSeq:WP_061186105.1, SO:0001217, UniParc:UPI0006A854E8, UniRef:UniRef100_A0A9Q4E3D3, UniRef:UniRef50_O05515, UniRef:UniRef90_O05515
EC:2.3.1.234, GO:0002949, GO:0005829, SO:0001217, UniRef:UniRef50_O05516, UniRef:UniRef90_O05516
EC:2.3.1.266, GO:0005737, GO:0008999, GO:0036211, GO:0042254, RefSeq:WP_072181782.1, SO:0001217, UniParc:UPI0006A93F6F, UniRef:UniRef100_UPI0006A93F6F, UniRef:UniRef50_O05517, UniRef:UniRef90_O055
EC:2.3.1.234, GO:0002949, GO:0005506, GO:0005737, GO:0061711, SO:0001217, UniRef:UniRef50_O05518, UniRef:UniRef90_O05518
GO:0003677, GO:0005524, GO:0016887, RefSeq:WP_014112878.1, SO:0001217, UniParc:UPI00022BB08B, UniRef:UniRef100_G4NU39, UniRef:UniRef50_O05519, UniRef:UniRef90_O05519
EC:4.6.1.17, GO:0006777, GO:0061799, KEGG:K03637, RefSeq:WP_014112879.1, SO:0001217, UniParc:UPI00022BB218, UniRef:UniRef100_G4NU40, UniRef:UniRef50_Q2FVX9, UniRef:UniRef90_O05520
GO:0003677, GO:0003700, GO:0005737, GO:0045892, GO:0051775, KEGG:K01926, RefSeq:WP_003225686.1, SO:0001217, UniParc:UPI0001D28B97, UniRef:UniRef100_A0A9Q4E3P6, UniRef:UniRef50_O05521, UniRef:U
BlastRules:WP_003234072, GO:0005829, GO:0008320, GO:0033281, GO:0043953, RefSeq:WP_003225684.1, SO:0001217, UniParc:UPI0001D28B96, UniRef:UniRef100_A0A9Q4HAK6, UniRef:UniRef50_O05522, UniRef:Un
GO:0005886, GO:0009977, GO:0015628, GO:0033281, GO:0043953, GO:0065002, KEGG:K03118, SO:0001217, UniParc:UPI00022BB219, UniRef:UniRef100_A0A9Q4HFD2, UniRef:UniRef50_O05523, UniRef:UniRef90_O05
GO:0005886, RefSeq:WP_003225680.1, SO:0001217, UniParc:UPI000005FFF2, UniRef:UniRef100_O05524, UniRef:UniRef50_O05524, UniRef:UniRef90_O05524
EC:3.4.-.-, GO:0004222, GO:0005886, GO:0071586, RefSeq:WP_014112881.1, SO:0001217, UniParc:UPI00022BB21A, UniRef:UniRef100_G4NU45, UniRef:UniRef50_O05525, UniRef:UniRef90_O05525
COG:COG0234, COG:O, GO:0005524, GO:0005737, GO:0006457, GO:0016887, GO:0044183, GO:0046872, GO:0051082, GO:0051085, GO:0051087, KEGG:K04078, RefSeq:WP_003155970.1, SO:0001217, UniParc:UPI00
COG:COG0459, COG:O, EC:5.6.1.7, GO:0005524, GO:0005737, GO:0016853, GO:0042026, GO:0051082, GO:0140662, RefSeq:WP_014112882.1, SO:0001217, UniParc:UPI00022BB21B, UniRef:UniRef100_A0A9Q4E272, U
RefSeq:WP_014112883.1, SO:0001217, UniParc:UPI00022BB21C, UniRef:UniRef100_G4NU48, UniRef:UniRef50_A0A1V3GCS4, UniRef:UniRef90_G4NU48
RefSeq:WP_014112884.1, SO:0001217, UniParc:UPI00022BB21D, UniRef:UniRef100_G4NU49, UniRef:UniRef50_G4NU49, UniRef:UniRef90_G4NU49
SO:0001217, UniRef:UniRef50_A0AAP1E6W1, UniRef:UniRef90_A0AAP1E6W1
SO:0001217, UniRef:UniRef50_A0AAP1E356, UniRef:UniRef90_A0AAP1E356
BlastRules:WP_003244156, EC:1.1.1.-, GO:0003939, GO:0008270, GO:0046526, SO:0001217, UniRef:UniRef50_Q06004, UniRef:UniRef90_Q06004
GO:0005886, GO:0006814, GO:0008643, GO:0015293, GO:0055085, KEGG:K16248, SO:0001217, UniRef:UniRef50_O34368, UniRef:UniRef90_O34368
EC:2.7.1.-, GO:0016301, SO:0001217, UniRef:UniRef50_O34768, UniRef:UniRef90_O34768
KEGG:K03969, SO:0001217, UniRef:UniRef50_P54617, UniRef:UniRef90_P54617
GO:0005886, RefSeq:WP_041336778.1, SO:0001217, UniParc:UPI00059E06D2, UniRef:UniRef100_A0AAP2M540, UniRef:UniRef50_O34434, UniRef:UniRef90_O34434
SO:0001217, UniRef:UniRef50_O35004, UniRef:UniRef90_UPI00100A1704
SO:0001217, UniRef:UniRef50_O34789, UniRef:UniRef90_O34789
GO:0005886, SO:0001217, UniRef:UniRef50_O34733, UniRef:UniRef90_O34733
BlastRules:WP_003234027, GO:0005886, GO:0015293, GO:0016020, GO:0055085, RefSeq:WP_061186120.1, SO:0001217, UniParc:UPI0007729BC0, UniRef:UniRef100_UPI0007729BC0, UniRef:UniRef50_O34718, UniRef:
BlastRules:WP_003234015, BlastRules:WP_033691573, EC:1.1.1.4, GO:0000721, GO:0005576, GO:0005737, GO:0008270, RefSeq:WP_061186121.1, SO:0001217, UniParc:UPI000772493C, UniRef:UniRef100_UPI000772
BlastRules:WP_003234013, RefSeq:WP_014112896.1, SO:0001217, UniParc:UPI00022BA4E2, UniRef:UniRef100_G4NU61, UniRef:UniRef50_P40775, UniRef:UniRef90_P40775
SO:0001217, UniRef:UniRef50_O34353
RefSeq:WP_014112898.1, SO:0001217, UniParc:UPI00022BB22A, UniRef:UniRef100_G4NU63, UniRef:UniRef50_O24817, UniRef:UniRef90_G4NU63
SO:0001217, UniRef:UniRef50_O34759, UniRef:UniRef90_O34759
EC:3.-.-.-, GO:0016020, GO:0016787, RefSeq:WP_014112899.1, SO:0001217, UniParc:UPI00022BB22B, UniRef:UniRef100_G4NU65, UniRef:UniRef50_O34592, UniRef:UniRef90_O34592
SO:0001217, UniRef:UniRef50_O34803, UniRef:UniRef90_O34803
BlastRules:WP_003243170, EC:1.10.3.2, GO:0005507, GO:0016491, GO:0030288, GO:0030435, RefSeq:WP_041521139.1, SO:0001217, UniParc:UPI00031BC42C, UniRef:UniRef100_UPI00031BC42C, UniRef:UniRef50_P0
GO:0005886, GO:0009450, GO:0015185, GO:0015812, GO:0016020, RefSeq:WP_014112901.1, SO:0001217, UniParc:UPI00022BB22D, UniRef:UniRef100_G4NU67, UniRef:UniRef50_P46349, UniRef:UniRef90_P46349
BlastRules:WP_003233995, GO:0005886, GO:0008324, GO:0016020, KEGG:K13283, SO:0001217, UniRef:UniRef50_P46348, UniRef:UniRef90_P46348
GO:0005524, GO:0016887, RefSeq:WP_072565865.1, SO:0001217, UniParc:UPI0009094DB7, UniRef:UniRef100_UPI0009094DB7, UniRef:UniRef50_P94474, UniRef:UniRef90_P94474
SO:0001217, UniRef:UniRef50_P94475, UniRef:UniRef90_E0TU48
EC:2.3.2.13, GO:0005886, KEGG:K22452, SO:0001217, UniRef:UniRef50_P94476, UniRef:UniRef90_P94476
PFAM:PF13559.11
COG:COG0519, COG:F, EC:6.3.5.2, GO:0003921, GO:0003922, GO:0005524, GO:0005829, GO:0006177, GO:0016462, SO:0001217, UniRef:UniRef50_Q73LZ4, UniRef:UniRef90_P29727
RFAM:RF00167, SO:0000035
BlastRules:WP_003243780, SO:0001217, UniRef:UniRef50_Q63GV3
GO:0005886, RefSeq:WP_014112909.1, SO:0001217, UniParc:UPI00022BB235, UniRef:UniRef100_G4NU75, UniRef:UniRef50_O34341, UniRef:UniRef90_O34341
RefSeq:WP_014112910.1, SO:0001217, UniParc:UPI00022BB236, UniRef:UniRef100_G4NU76, UniRef:UniRef50_O34904, UniRef:UniRef90_O34904
GO:0005886, RefSeq:WP_041520875.1, SO:0001217, UniParc:UPI0002F3AD2B, UniRef:UniRef100_A0A9Q4HGR2, UniRef:UniRef50_O34624, UniRef:UniRef90_O34624
RefSeq:WP_003219403.1, SO:0001217, UniParc:UPI000006001B, UniRef:UniRef100_O34700, UniRef:UniRef50_O34700, UniRef:UniRef90_O34700
RFAM:RF00167, SO:0000035
EC:5.4.99.18, GO:0006189, GO:0034023, RefSeq:WP_003219405.1, SO:0001217, UniParc:UPI0001D28E88, UniRef:UniRef100_G4NU79, UniRef:UniRef50_P12044, UniRef:UniRef90_P12044
EC:6.3.4.18, GO:0004638, GO:0005524, GO:0005829, GO:0006189, GO:0034028, GO:0046872, SO:0001217, UniRef:UniRef50_P12045, UniRef:UniRef90_P12045
EC:4.3.2.2, GO:0004018, GO:0005829, GO:0006167, GO:0006189, GO:0009152, GO:0044208, GO:0070626, KEGG:K01756, RefSeq:WP_024715850.1, SO:0001217, UniParc:UPI000465824C, UniRef:UniRef100_UPI000465
EC:6.3.2.6, GO:0004639, GO:0005524, GO:0006164, GO:0006189, GO:0009236, RefSeq:WP_014112915.1, SO:0001217, UniParc:UPI00022BB3B5, UniRef:UniRef100_G4NU82, UniRef:UniRef50_P12046, UniRef:UniRef90_
COG:COG1828, COG:F, EC:6.3.5.3, GO:0004642, GO:0005524, GO:0005737, GO:0006189, GO:0009152, RefSeq:WP_014112916.1, SO:0001217, UniParc:UPI00022BB3B6, UniRef:UniRef100_G4NU83, UniRef:UniRef50_P1
COG:COG0047, COG:F, EC:3.5.1.2, EC:6.3.5.3, GO:0004359, GO:0004642, GO:0005524, GO:0005737, GO:0006189, RefSeq:WP_014112917.1, SO:0001217, UniParc:UPI00022BB3B7, UniRef:UniRef100_G4NU84, UniRef:U
EC:6.3.5.3, GO:0000287, GO:0004642, GO:0005524, GO:0005737, GO:0006189, KEGG:K23269, RefSeq:WP_090558435.1, SO:0001217, UniParc:UPI00086AD2D8, UniRef:UniRef100_UPI00086AD2D8, UniRef:UniRef50_P1
COG:COG0034, COG:F, EC:2.4.2.14, GO:0000287, GO:0004044, GO:0006164, GO:0006189, GO:0009113, GO:0051539, KEGG:K00764, RefSeq:WP_014112919.1, SO:0001217, UniParc:UPI00022BB3B9, UniRef:UniRef100
EC:6.3.3.1, GO:0004637, GO:0004641, GO:0005524, GO:0005829, GO:0006189, GO:0046084, RefSeq:WP_072181553.1, SO:0001217, UniParc:UPI0006A83861, UniRef:UniRef100_UPI0006A83861, UniRef:UniRef50_P1204
EC:2.1.2.2, GO:0004644, GO:0005737, GO:0005829, GO:0006189, SO:0001217, UniRef:UniRef50_P12040, UniRef:UniRef90_P12040
COG:COG0138, COG:F, EC:2.1.2.3, EC:3.5.4.10, GO:0003937, GO:0004643, GO:0005829, GO:0006189, GO:0009152, KEGG:K00602, RefSeq:WP_072181550.1, SO:0001217, UniParc:UPI0006A8A266, UniRef:UniRef100_U
EC:6.3.4.13, GO:0004637, GO:0005524, GO:0006189, GO:0009113, GO:0046872, SO:0001217, UniRef:UniRef50_P12039, UniRef:UniRef90_P12039
GO:0005829, GO:0043200, GO:0043565, RefSeq:WP_014112924.1, SO:0001217, UniParc:UPI00022BB3BE, UniRef:UniRef100_G4NU91, UniRef:UniRef50_O31497, UniRef:UniRef90_O31497
KEGG:K16263, SO:0001217, UniRef:UniRef50_O34560, UniRef:UniRef90_UPI00086EC8F0
GO:0005886, RefSeq:WP_090558442.1, SO:0001217, UniParc:UPI00086E6A5E, UniRef:UniRef100_UPI00086E6A5E, UniRef:UniRef50_C0H3X3, UniRef:UniRef90_C0H3X3
EC:3.5.4.2, GO:0000034, GO:0006146, KEGG:K01486, SO:0001217, UniRef:UniRef50_O34909, UniRef:UniRef90_O34909
GO:0005886, SO:0001217, UniRef:UniRef50_O34968, UniRef:UniRef90_O34968
GO:0003700, GO:0043565, RefSeq:WP_003219429.1, SO:0001217, UniParc:UPI000006002C, UniRef:UniRef100_Q7BVT7, UniRef:UniRef50_Q7BVT7, UniRef:UniRef90_Q7BVT7
GO:0005886, GO:0006537, GO:0015930, SO:0001217, UniRef:UniRef50_O34849, UniRef:UniRef90_O34849
EC:2.5.1.n9, GO:0000287, GO:0002094, GO:0016765, GO:0046474, RefSeq:WP_014112931.1, SO:0001217, UniParc:UPI00022BB3C5, UniRef:UniRef100_G4NU99, UniRef:UniRef50_O34790, UniRef:UniRef90_O34790
EC:3.6.4.12, EC:5.6.2.4, GO:0000725, GO:0003677, GO:0003678, GO:0003689, GO:0005524, GO:0005737, GO:0005829, GO:0006268, GO:0006281, GO:0009295, GO:0009378, GO:0016853, GO:0016887, GO:0033202, GC

EC:6.5.1.2, GO:0003677, GO:0003911, GO:0005524, GO:0005829, GO:0006260, GO:0006281, GO:0006288, GO:0006355, GO:0046872, SO:0001217, UniRef:UniRef50_O31498, UniRef:UniRef90_O31498
GO:0005886, GO:0045121, SO:0001217, UniRef:UniRef50_O34629, UniRef:UniRef90_O34629
EC:2.7.1.230, GO:0004413, GO:0009088, GO:0046677, KEGG:K23983, SO:0001217, UniRef:UniRef50_O34640, UniRef:UniRef90_O34640
GO:0005886, SO:0001217, UniParc:UPI0001F5C131, UniRef:UniRef100_G4NUA4, UniRef:UniRef50_Q45514, UniRef:UniRef90_Q45514
GO:0005298, GO:0005886, GO:0006814, GO:0015193, GO:0015824, GO:0016020, GO:0031402, GO:0055085, RefSeq:WP_014112937.1, SO:0001217, UniParc:UPI00022BB3CA, UniRef:UniRef100_G4NUA5, UniRef:UniRef
EC:6.3.5.-, GO:0005524, GO:0006412, GO:0006424, GO:0006450, GO:0030956, GO:0050566, GO:0050567, GO:0070681, RefSeq:WP_003219442.1, SO:0001217, UniParc:UPI0000060035, UniRef:UniRef100_O06492, UniR
COG:COG0154, COG:J, EC:6.3.5.7, GO:0005524, GO:0006412, GO:0006424, GO:0030956, GO:0050567, KEGG:K02433, RefSeq:WP_003242878.1, SO:0001217, UniParc:UPI0000060036, UniRef:UniRef100_O06491, UniRe
COG:COG0064, COG:J, EC:6.3.5.-, GO:0005524, GO:0006412, GO:0016884, GO:0050566, GO:0050567, GO:0070681, KEGG:K02434, RefSeq:WP_003219444.1, SO:0001217, UniParc:UPI00001B0918, UniRef:UniRef100_A
GO:0003677, SO:0001217, UniRef:UniRef50_O31500, UniRef:UniRef90_O31500
SO:0001217, UniRef:UniRef50_A0A7W3XRM2, UniRef:UniRef90_UPI002281AB4D
SO:0001217, UniRef:UniRef50_A0A2M9NS30
EC:2.7.1.107, GO:0003951, GO:0004143, GO:0005524, GO:0006665, GO:0008654, GO:0016020, GO:0046872, SO:0001217, UniRef:UniRef50_O31502, UniRef:UniRef90_O31502
SO:0001217, UniRef:UniRef50_UPI001F05AD36
EC:2.1.1.189, EC:2.1.1.190, GO:0008649, GO:0031167, GO:0046872, GO:0051539, GO:0070041, GO:0070475, KEGG:K03215, SO:0001217, UniRef:UniRef50_O31503, UniRef:UniRef90_O31503
RefSeq:WP_014112942.1, SO:0001217, UniParc:UPI00022BB53A, UniRef:UniRef100_G4NUB4, UniRef:UniRef50_G4NUB4, UniRef:UniRef90_G4NUB4
RefSeq:WP_014112943.1, SO:0001217, UniParc:UPI00022BB53B, UniRef:UniRef100_G4NR72, UniRef:UniRef50_G4NR72, UniRef:UniRef90_G4NR72
SO:0001217, UniRef:UniRef50_A0A060PPR2, UniRef:UniRef90_A0A8E0S5U8
SO:0001217, UniRef:UniRef50_A0AAP3FVF2, UniRef:UniRef90_UPI003645097C
SO:0001217, UniRef:UniRef50_O31507
EC:3.1.3.-, GO:0004721, GO:0005737, GO:0030420, GO:0030435, KEGG:K06366, SO:0001217, UniRef:UniRef50_Q59HN8, UniRef:UniRef90_Q59HN8
KEGG:K20389, SO:0001217, UniRef:UniRef50_Q59HN7, UniRef:UniRef90_A0A9Q4E3I5
GO:0003677, GO:0005829, GO:0006355, SO:0001217, UniRef:UniRef50_P0A8A2, UniRef:UniRef90_O31509
GO:0000976, GO:0003700, GO:0006355, SO:0001217, UniRef:UniRef50_Q7WY76, UniRef:UniRef90_Q7WY76
RefSeq:WP_072182261.1, SO:0001217, UniParc:UPI0006A929C5, UniRef:UniRef100_UPI0006A929C5, UniRef:UniRef50_O31511, UniRef:UniRef90_O31511
SO:0001217, UniRef:UniRef50_A0A448IH78, UniRef:UniRef90_A0A9Q4HGG2
BlastRules:WP_003219489, RefSeq:WP_014112953.1, SO:0001217, UniParc:UPI00022BB545, UniRef:UniRef100_G4NR82, UniRef:UniRef50_Q45536, UniRef:UniRef90_Q45536
BlastRules:WP_003219491, RefSeq:WP_041520881.1, SO:0001217, UniParc:UPI00031AE0D1, UniRef:UniRef100_G4NR83, UniRef:UniRef50_Q45537, UniRef:UniRef90_Q45537
BlastRules:WP_000265278, BlastRules:WP_003233850, COG:COG3546, COG:P, RefSeq:WP_003219494.1, SO:0001217, UniParc:UPI0001D28FB3, UniRef:UniRef100_A0A9Q4DPU4, UniRef:UniRef50_Q45538, UniRef:UniRe
EC:2.3.1.-, GO:0008080, SO:0001217, UniRef:UniRef50_O31513, UniRef:UniRef90_O31513
GO:0005886, SO:0001217, UniRef:UniRef50_O31515, UniRef:UniRef90_O31515
BlastRules:WP_003243833, EC:2.7.13.3, GO:0000155, GO:0005524, GO:0005886, KEGG:K07718, RefSeq:WP_088679846.1, SO:0001217, UniParc:UPI000B534102, UniRef:UniRef100_UPI000B534102, UniRef:UniRef50_O3
BlastRules:WP_003244566, GO:0000160, GO:0003700, GO:0005737, GO:0043565, RefSeq:WP_088679845.1, SO:0001217, UniParc:UPI000B529A49, UniRef:UniRef100_UPI000B529A49, UniRef:UniRef50_O31517, UniRef:U
KEGG:K25676, SO:0001217, UniRef:UniRef50_O31518, UniRef:UniRef90_O31518
SO:0001217, UniRef:UniRef50_UPI00102E4001, UniRef:UniRef90_UPI00102E4001
SO:0001217, UniRef:UniRef50_A0A961L916
GO:0005886, GO:0055085, KEGG:K25678, RefSeq:WP_014112961.1, SO:0001217, UniParc:UPI00022BB54D, UniRef:UniRef100_G4NR91, UniRef:UniRef50_O31520, UniRef:UniRef90_O31520
SO:0001217, UniRef:UniRef50_O31521
GO:0003700, GO:0005886, GO:0043565, KEGG:K07720, SO:0001217, UniRef:UniRef50_O31522, UniRef:UniRef90_O31522
PFAM:PF00165.29, PFAM:PF12833.13
PFAM:PF09224.18
RefSeq:WP_072182251.1, SO:0001217, UniParc:UPI0006A9085D, UniRef:UniRef100_UPI0006A9085D, UniRef:UniRef50_O31525, UniRef:UniRef90_O31525
SO:0001217, UniRef:UniRef50_A0A2P8IF33
PFAM:PF21348.3
SO:0001217, UniRef:UniRef50_A0A653WGV8
EC:4.2.2.24, GO:0005576, GO:0016829, GO:0046872, GO:0071555, KEGG:K18197, SO:0001217, UniRef:UniRef50_O31527, UniRef:UniRef90_O31527
EC:3.1.1.-, GO:0016788, RefSeq:WP_072182248.1, SO:0001217, UniParc:UPI0006A88282, UniRef:UniRef100_UPI0006A88282, UniRef:UniRef50_O31528, UniRef:UniRef90_O31528
EC:3.2.1.23, GO:0004565, GO:0006012, GO:0009341, GO:0046872, SO:0001217, UniRef:UniRef50_O31529, UniRef:UniRef90_O31529
SO:0001217, UniRef:UniRef50_O31530, UniRef:UniRef90_O31530
GO:0005886, KEGG:K17318, SO:0001217, UniRef:UniRef50_P37966, UniRef:UniRef90_P37966
GO:0005886, GO:0055085, KEGG:K17319, SO:0001217, UniRef:UniRef50_C0SPB3, UniRef:UniRef90_P39128
GO:0005886, GO:0055085, KEGG:K17320, RefSeq:WP_014112974.1, SO:0001217, UniParc:UPI00022BB6D0, UniRef:UniRef100_G4NRA4, UniRef:UniRef50_P53561, UniRef:UniRef90_P39129
SO:0001217, UniRef:UniRef50_A0A354J1T8
PFAM:PF11975.13
RefSeq:WP_072565911.1, SO:0001217, UniParc:UPI00086CC9E4, UniRef:UniRef100_UPI00086CC9E4, UniRef:UniRef50_O31533, UniRef:UniRef90_G4NRA6
BlastRules:WP_003243958, EC:1.14.14.18, GO:0004392, GO:0005737, GO:0046872, KEGG:K07145, RefSeq:WP_003239990.1, SO:0001217, UniParc:UPI00022BB6D3, UniRef:UniRef100_A0A9W5LGY3, UniRef:UniRef50_O
SO:0001217, UniRef:UniRef50_O31535, UniRef:UniRef90_O31535
SO:0001217, UniRef:UniRef50_A0A0D1JDI8, UniRef:UniRef90_A0A0D1JDI8
GO:0005262, GO:0005886, GO:0006816, GO:0016020, GO:0030162, RefSeq:WP_014112981.1, SO:0001217, UniParc:UPI00022BB6D8, UniRef:UniRef100_G4NRB2, UniRef:UniRef50_O31539, UniRef:UniRef90_O31539
GO:0005886, SO:0001217, UniRef:UniRef50_O31540, UniRef:UniRef90_O31540
SO:0001217, UniRef:UniRef50_O31541
EC:1.14.-.-, GO:0004497, GO:0071949, SO:0001217, UniRef:UniRef50_O06489, UniRef:UniRef90_O06489
KEGG:K07448, RefSeq:WP_041520883.1, SO:0001217, UniParc:UPI0003125E60, UniRef:UniRef100_UPI0003125E60, UniRef:UniRef50_U6SSL3, UniRef:UniRef90_A0A9Q2QQ76
RefSeq:WP_372402539.1, SO:0001217, UniParc:UPI0035572EB7, UniRef:UniRef100_UPI0035572EB7, UniRef:UniRef50_O06490, UniRef:UniRef90_O06490
BlastRules:WP_000412767, BlastRules:WP_003242884, EC:1.14.14.1, EC:1.6.2.4, GO:0003958, GO:0005504, GO:0005506, GO:0005829, GO:0010181, GO:0016491, GO:0016712, GO:0019395, GO:0020037, GO:0050660, (
BlastRules:WP_003243930, EC:2.7.8.-, EC:2.7.8.20, GO:0005576, GO:0005886, GO:0016740, GO:0046872, GO:0070395, GO:0071555, KEGG:K19005, RefSeq:WP_072182904.1, SO:0001217, UniParc:UPI0006A7F539, Unil
EC:2.7.7.33, GO:0000166, GO:0009058, GO:0046872, GO:0047343, RefSeq:WP_014112990.1, SO:0001217, UniParc:UPI00022BB6E1, UniRef:UniRef100_G4NRC1, UniRef:UniRef50_O06486, UniRef:UniRef90_O06486
EC:4.2.1.45, KEGG:K01709, SO:0001217, UniRef:UniRef50_O06485, UniRef:UniRef90_O06485
SO:0001217, UniRef:UniRef50_O06484, UniRef:UniRef90_O06484
SO:0001217, UniRef:UniRef50_UPI00227FBB9B, UniRef:UniRef90_UPI00227FBB9B
EC:2.4.-.-, GO:0016757, SO:0001217, UniRef:UniRef50_O06483, UniRef:UniRef90_O06483
RefSeq:WP_072182907.1, SO:0001217, UniParc:UPI0006A8CEE0, UniRef:UniRef100_UPI0006A8CEE0, UniRef:UniRef50_O31542, UniRef:UniRef90_O31542
GO:0005886, GO:0022857, RefSeq:WP_072182908.1, SO:0001217, UniParc:UPI0006A8F35C, UniRef:UniRef100_UPI0006A8F35C, UniRef:UniRef50_O06481, UniRef:UniRef90_O06481
EC:3.-.-.-, EC:3.1.3.5, GO:0008253, RefSeq:WP_072182909.1, SO:0001217, UniParc:UPI0006A89805, UniRef:UniRef100_UPI0006A89805, UniRef:UniRef50_O06480, UniRef:UniRef90_O06480
GO:0005886, GO:0006865, GO:0015171, RefSeq:WP_014112995.1, SO:0001217, UniParc:UPI00022BB6E6, UniRef:UniRef100_G4NRC6, UniRef:UniRef50_Q797A7, UniRef:UniRef90_Q797A7
BlastRules:WP_003244104, EC:1.2.1.28, GO:0018479, GO:0050608, RefSeq:WP_014112996.1, SO:0001217, UniParc:UPI00022BB6E7, UniRef:UniRef100_G4NRC7, UniRef:UniRef50_O06478, UniRef:UniRef90_O06478
GO:0007165, GO:0016020, SO:0001217, UniRef:UniRef50_O06477, UniRef:UniRef90_O06477
EC:7.-.-.-, GO:0003677, GO:0005524, GO:0016887, SO:0001217, UniRef:UniRef50_O06476, UniRef:UniRef90_O06476
RefSeq:WP_072182912.1, SO:0001217, UniParc:UPI0006A8C761, UniRef:UniRef100_UPI0006A8C761, UniRef:UniRef50_O06475, UniRef:UniRef90_A0A9Q6A8A0
GO:0003677, GO:0003700, RefSeq:WP_003223002.1, SO:0001217, UniParc:UPI0001D28B62, UniRef:UniRef100_A0A9Q4DT80, UniRef:UniRef50_O06474, UniRef:UniRef90_O06474
GO:0005886, GO:0006825, GO:0022857, SO:0001217, UniRef:UniRef50_O06473, UniRef:UniRef90_O06473
RefSeq:WP_072182913.1, SO:0001217, UniParc:UPI0006A8C6A8, UniRef:UniRef100_UPI0006A8C6A8, UniRef:UniRef50_A0A378XPY2, UniRef:UniRef90_G4NRD3
COG:COG0488, COG:R, EC:7.-.-.-, GO:0003677, GO:0005524, GO:0016887, RefSeq:WP_087987746.1, SO:0001217, UniParc:UPI000B4508A0, UniRef:UniRef100_A0A9Q4E3F5, UniRef:UniRef50_O34512, UniRef:UniRef90_
EC:3.6.4.13, GO:0003689, GO:0003724, GO:0005524, GO:0005829, GO:0009409, GO:0016887, GO:0033592, GO:0042254, GO:0061775, GO:0140584, GO:0140665, GO:0140849, SO:0001217, UniRef:UniRef50_O34750, U
RefSeq:WP_014113006.1, SO:0001217, UniParc:UPI00022BB84B, UniRef:UniRef100_G4NRD8, UniRef:UniRef50_O34536, UniRef:UniRef90_G4NRD8
SO:0001217, UniRef:UniRef50_O34812, UniRef:UniRef90_G4NRD9
GO:0005886, GO:0022857, RefSeq:WP_088679824.1, SO:0001217, UniParc:UPI00086E04CA, UniRef:UniRef100_UPI00086E04CA, UniRef:UniRef50_O34440, UniRef:UniRef90_O34440
SO:0001217, UniRef:UniRef50_UPI0034D3E4AB
SO:0001217, UniRef:UniRef50_A0A5S9MFI3
SO:0001217, UniRef:UniRef50_A0A376MII6
BlastRules:WP_003243506, GO:0005886, GO:0022857, GO:0033214, RefSeq:WP_088679821.1, SO:0001217, UniParc:UPI000B531AAC, UniRef:UniRef100_UPI000B531AAC, UniRef:UniRef50_O34832, UniRef:UniRef90_O3
BlastRules:WP_003243835, GO:0005886, GO:0022857, GO:0033214, SO:0001217, UniRef:UniRef50_O34933, UniRef:UniRef90_O34933
BlastRules:WP_003243996, GO:0005886, GO:0030288, GO:1901678, KEGG:K23181, RefSeq:WP_088679820.1, SO:0001217, UniParc:UPI000869995A, UniRef:UniRef100_UPI000869995A, UniRef:UniRef50_O34348, UniRef
RefSeq:WP_014113014.1, SO:0001217, UniParc:UPI00022BB854, UniRef:UniRef100_G4NRE7, UniRef:UniRef50_O34626, UniRef:UniRef90_A0A9W5LH04
RefSeq:WP_003223026.1, SO:0001217, UniParc:UPI0001D28C30, UniRef:UniRef100_A0A9Q4E1W0, UniRef:UniRef50_P80241, UniRef:UniRef90_P80241
BlastRules:WP_003244025, EC:4.2.2.2, GO:0005576, GO:0030570, GO:0045490, GO:0046872, KEGG:K01728, RefSeq:WP_088679819.1, SO:0001217, UniParc:UPI000B52F25D, UniRef:UniRef100_UPI000B52F25D, UniRef
GO:0005886, GO:0022857, KEGG:K03319, RefSeq:WP_014113016.1, SO:0001217, UniParc:UPI00022BB856, UniRef:UniRef100_G4NRF0, UniRef:UniRef50_O34726, UniRef:UniRef90_O34726
EC:2.7.13.3, GO:0000155, GO:0005524, GO:0005886, SO:0001217, UniRef:UniRef50_O34427, UniRef:UniRef90_O34427
BlastRules:WP_003242754, GO:0000156, GO:0003677, GO:0003700, GO:0005737, KEGG:K11638, RefSeq:WP_072182775.1, SO:0001217, UniParc:UPI0006A868DC, UniRef:UniRef100_UPI0006A868DC, UniRef:UniRef50_(
RefSeq:WP_072565933.1, SO:0001217, UniParc:UPI00090BE725, UniRef:UniRef100_UPI00090BE725, UniRef:UniRef50_O34439, UniRef:UniRef90_O34439
BlastRules:WP_003242978, COG:C, COG:COG2851, GO:0005886, GO:0006101, GO:0015137, GO:0015293, GO:0022857, GO:0055085, RefSeq:WP_072182779.1, SO:0001217, UniParc:UPI0006A87A55, UniRef:UniRef100_
EC:3.-.-.-, GO:0016787, GO:0046872, RefSeq:WP_088679817.1, SO:0001217, UniParc:UPI000B52EC29, UniRef:UniRef100_UPI000B52EC29, UniRef:UniRef50_O34409, UniRef:UniRef90_O34409
EC:1.14.14.47, GO:0004517, GO:0006809, GO:0020037, GO:0046872, RefSeq:WP_088679816.1, SO:0001217, UniParc:UPI000B533C1C, UniRef:UniRef100_UPI000B533C1C, UniRef:UniRef50_O34453, UniRef:UniRef90_O:
PFAM:PF00708.24
GO:0003824, GO:0030151, GO:0030170, RefSeq:WP_072182784.1, SO:0001217, UniParc:UPI0006A8DD42, UniRef:UniRef100_A0A9Q4E1W6, UniRef:UniRef50_O34542, UniRef:UniRef90_O34542
RefSeq:WP_014113026.1, SO:0001217, UniParc:UPI00022BB860, UniRef:UniRef100_G4NUC6, UniRef:UniRef50_O34345, UniRef:UniRef90_O34345
RefSeq:WP_014113028.1, SO:0001217, UniParc:UPI00022BB862, UniRef:UniRef100_G4NUC8, UniRef:UniRef50_O34306, UniRef:UniRef90_G4NUC8
EC:3.4.11.18, GO:0004239, GO:0005737, GO:0006508, GO:0046872, GO:0070006, KEGG:K01265, RefSeq:WP_003223067.1, SO:0001217, UniParc:UPI0001D28C3E, UniRef:UniRef100_A0A9Q4DPV0, UniRef:UniRef50_O34
SO:0001217, UniRef:UniRef50_A0A0B0CWM6
BlastRules:WP_003233694, EC:2.7.8.20, GO:0005576, GO:0005886, GO:0016740, GO:0046872, GO:0070395, GO:0071555, KEGG:K19005, SO:0001217, UniRef:UniRef50_O34952, UniRef:UniRef90_O34952
SO:0001217, UniRef:UniRef50_O34887, UniRef:UniRef90_O34887
GO:0005283, GO:0005886, KEGG:K03310, RefSeq:WP_014113032.1, SO:0001217, UniParc:UPI00022BB866, UniRef:UniRef100_G4NUD3, UniRef:UniRef50_O34708, UniRef:UniRef90_O34708
SO:0001217, UniRef:UniRef50_A0AAP1E908
SO:0001217, UniRef:UniRef50_A0A7Z7QRZ4
SO:0001217, UniRef:UniRef50_UPI000301B6AB
GO:0003677, GO:0003700, GO:0006355, GO:0045892, KEGG:K03486, SO:0001217, UniRef:UniRef50_P39796, UniRef:UniRef90_P39796
EC:1.-.-.-, GO:0005829, GO:0016491, GO:0046256, RefSeq:WP_003223097.1, SO:0001217, UniParc:UPI0001D28C4A, UniRef:UniRef100_A0A9Q4DS43, UniRef:UniRef50_O34475, UniRef:UniRef90_O34475
EC:3.1.3.5, EC:3.1.3.6, EC:3.1.4.16, GO:0000166, GO:0005576, GO:0008254, GO:0008663, GO:0009166, GO:0030288, GO:0046872, GO:0106411, SO:0001217, UniRef:UniRef50_O34313, UniRef:UniRef90_O34313
BlastRules:WP_003243504, EC:3.2.-.-, GO:0006508, GO:0008233, RefSeq:WP_072182792.1, SO:0001217, UniParc:UPI0006A8CF94, UniRef:UniRef100_A0A9Q4HGD2, UniRef:UniRef50_P80876, UniRef:UniRef90_P80876
GO:0005886, GO:0016020, GO:0022857, KEGG:K26745, SO:0001217, UniRef:UniRef50_O34597, UniRef:UniRef90_O34597
RefSeq:WP_003223104.1, SO:0001217, UniParc:UPI0001D28C4E, UniRef:UniRef100_A0A9Q4DP28, UniRef:UniRef50_O35019, UniRef:UniRef90_O35019
EC:3.1.3.48, GO:0004725, GO:0004726, GO:0030946, GO:0140793, RefSeq:WP_003223106.1, SO:0001217, UniParc:UPI0001D28C4F, UniRef:UniRef100_E0TVK8, UniRef:UniRef50_O35016, UniRef:UniRef90_O35016
RefSeq:WP_061188176.1, SO:0001217, UniParc:UPI0006A8CB31, UniRef:UniRef100_A0A9Q4E2Z7, UniRef:UniRef50_O34418, UniRef:UniRef90_O34418
RefSeq:WP_014113047.1, SO:0001217, UniParc:UPI00022BBA0C, UniRef:UniRef100_G4NUE9, UniRef:UniRef50_A0A0A8XD39, UniRef:UniRef90_A0A9W5LGS9
SO:0001217, UniRef:UniRef50_E0TVL1, UniRef:UniRef90_E0TVL1
GO:0005886, GO:0022857, RefSeq:WP_090558483.1, SO:0001217, UniParc:UPI000869AFB9, UniRef:UniRef100_UPI000869AFB9, UniRef:UniRef50_O34929, UniRef:UniRef90_O34929
GO:0005886, GO:0006816, GO:0006874, GO:0015369, GO:0070588, RefSeq:WP_014113049.1, SO:0001217, UniParc:UPI00022BBA0E, UniRef:UniRef100_G4NUF1, UniRef:UniRef50_O34840, UniRef:UniRef90_O34840
SO:0001217, UniRef:UniRef50_O34579, UniRef:UniRef90_O34579

SO:0001217, UniRef:UniRef50_O35043
COG:COG0535, COG:DH, GO:0003824, GO:0008152, GO:0046872, GO:0051539, GO:1904047, SO:0001217, UniRef:UniRef50_O34400, UniRef:UniRef90_O34400
SO:0001217, UniRef:UniRef50_O35041, UniRef:UniRef90_O35041
EC:3.5.1.-, GO:0005975, GO:0016810, GO:0019213, GO:0030435, GO:0046872, GO:0071555, SO:0001217, UniRef:UniRef50_O34928, UniRef:UniRef90_O34928
EC:1.1.-.-, GO:0016054, GO:0016491, GO:0050661, GO:0051287, SO:0001217, UniRef:UniRef50_O34969, UniRef:UniRef90_O34969
SO:0001217, UniRef:UniRef50_O31543
EC:3.2.2.21, GO:0005737, GO:0006285, GO:0006307, GO:0008725, GO:0032131, GO:0032993, GO:0043916, GO:0052821, GO:0052822, GO:1990053, RefSeq:WP_072182803.1, SO:0001217, UniParc:UPI0006A87F57, Uni
EC:2.1.1.-, EC:2.1.1.190, GO:0008649, GO:0046872, GO:0051539, GO:0070041, GO:0070475, KEGG:K03215, SO:0001217, UniRef:UniRef50_O31545, UniRef:UniRef90_O31545
GO:0005886, RefSeq:WP_041520891.1, SO:0001217, UniParc:UPI0005A045DD, UniRef:UniRef100_UPI0005A045DD, UniRef:UniRef50_C0H3X6, UniRef:UniRef90_C0H3X6
COG:COG0042, COG:J, EC:1.3.1.-, GO:0000049, GO:0017150, GO:0050660, RefSeq:WP_072182520.1, SO:0001217, UniParc:UPI0006A88CD0, UniRef:UniRef100_UPI0006A88CD0, UniRef:UniRef50_O31546, UniRef:UniRe
RefSeq:WP_088679806.1, SO:0001217, UniParc:UPI000B5377AB, UniRef:UniRef100_UPI000B5377AB, UniRef:UniRef50_M7NF59, UniRef:UniRef90_G4NUG2
RefSeq:WP_061188182.1, SO:0001217, UniParc:UPI000772A286, UniRef:UniRef100_UPI000772A286, UniRef:UniRef50_O31547, UniRef:UniRef90_O31547
SO:0001217, UniRef:UniRef50_P40773, UniRef:UniRef90_UPI000772379F
BlastRules:WP_000921260, BlastRules:WP_003242898, EC:1.1.1.-, GO:0004739, GO:0006086, GO:0045150, RefSeq:WP_061188184.1, SO:0001217, UniParc:UPI00077234EC, UniRef:UniRef100_A0A9Q4HRK0, UniRef:UniF
BlastRules:WP_000198888, BlastRules:WP_003243124, EC:1.1.1.-, GO:0016491, GO:0045150, RefSeq:WP_087987735.1, SO:0001217, UniParc:UPI000869287B, UniRef:UniRef100_UPI000869287B, UniRef:UniRef50_P277
EC:2.3.1.12, GO:0004742, GO:0045150, RefSeq:WP_088679805.1, SO:0001217, UniParc:UPI00086F88FB, UniRef:UniRef100_UPI00086F88FB, UniRef:UniRef50_O31550, UniRef:UniRef90_O31550
BlastRules:WP_003243007, EC:1.8.1.4, GO:0004148, GO:0005737, GO:0006090, GO:0006103, GO:0045150, GO:0050660, KEGG:K00382, RefSeq:WP_061188187.1, SO:0001217, UniParc:UPI00077257B2, UniRef:UniRef10
PFAM:PF01590.32
SO:0001217, UniRef:UniRef50_N0B1U7
KEGG:K06425, RefSeq:WP_003239795.1, SO:0001217, UniParc:UPI00022BBA1F, UniRef:UniRef100_A0A9Q4DPY2, UniRef:UniRef50_A6TQ57, UniRef:UniRef90_A0A9Q4DPY2
SO:0001217, UniRef:UniRef50_Q9K9A5
RefSeq:WP_161490084.1, SO:0001217, UniParc:UPI00090C7EDA, UniRef:UniRef100_UPI00090C7EDA, UniRef:UniRef50_O31555, UniRef:UniRef90_D4G6H4
RefSeq:WP_072182480.1, SO:0001217, UniParc:UPI0006A8F098, UniRef:UniRef100_UPI0006A8F098, UniRef:UniRef50_O31556, UniRef:UniRef90_E0TVN6
RefSeq:WP_014113069.1, SO:0001217, UniParc:UPI00022BBA23, UniRef:UniRef100_G4NUH4, UniRef:UniRef50_O31557, UniRef:UniRef90_O31557
RefSeq:WP_061188192.1, SO:0001217, UniParc:UPI0007726553, UniRef:UniRef100_UPI0007726553, UniRef:UniRef50_O31558, UniRef:UniRef90_O31558
BlastRules:WP_003244008, EC:3.2.1.122, GO:0005975, GO:0016616, GO:0046872, GO:0050081, KEGG:K01232, SO:0001217, UniRef:UniRef50_P54716, UniRef:UniRef90_P54716
SO:0001217, UniRef:UniRef50_UPI002E27AD63
SO:0001217, UniRef:UniRef50_A0A930R1Q8
EC:2.7.1.-, EC:2.7.1.208, GO:0005886, GO:0008982, GO:0009401, GO:0016301, GO:0090563, KEGG:K02750, SO:0001217, UniRef:UniRef50_P54715, UniRef:UniRef90_P54715
GO:0005524, GO:0005886, GO:0016887, GO:0042626, GO:0055085, GO:0140359, RefSeq:WP_014113074.1, SO:0001217, UniParc:UPI00022BBBBC, UniRef:UniRef100_G4NUH9, UniRef:UniRef50_P54718, UniRef:UniRef9
GO:0005524, GO:0005886, GO:0016887, GO:0042626, GO:0055085, GO:0140359, SO:0001217, UniRef:UniRef50_P54719, UniRef:UniRef90_P54719
BlastRules:WP_003244204, EC:1.-.-.-, GO:0005886, GO:0009056, GO:0009636, GO:0016491, RefSeq:WP_003239773.1, SO:0001217, UniParc:UPI0002AD0356, UniRef:UniRef100_A0A2M8SX49, UniRef:UniRef50_P54720,
EC:1.13.11.2, GO:0008198, GO:0009056, GO:0009636, GO:0018577, SO:0001217, UniRef:UniRef50_P54721, UniRef:UniRef90_P54721
SO:0001217, UniRef:UniRef50_P94438, UniRef:UniRef90_UPI0026768F65
BlastRules:WP_003242530, GO:0000160, GO:0003677, GO:0005737, GO:0006355, RefSeq:WP_014113083.1, SO:0001217, UniParc:UPI00022BBBC5, UniRef:UniRef100_G4NUI8, UniRef:UniRef50_P94439, UniRef:UniRef9
BlastRules:WP_003244346, EC:7.6.2.-, GO:0005524, GO:0016887, RefSeq:WP_061188202.1, SO:0001217, UniParc:UPI0006A908BA, UniRef:UniRef100_UPI0006A908BA, UniRef:UniRef50_P94440, UniRef:UniRef90_P944
RefSeq:WP_072182489.1, SO:0001217, UniParc:UPI0006A92284, UniRef:UniRef100_UPI0006A92284, UniRef:UniRef50_P94441, UniRef:UniRef90_G4NUJ1
SO:0001217, UniRef:UniRef50_P94442, UniRef:UniRef90_E0TW93
RefSeq:WP_087987724.1, SO:0001217, UniParc:UPI000B436A59, UniRef:UniRef100_UPI000B436A59, UniRef:UniRef50_A0A0Q9PRR3, UniRef:UniRef90_G4NUJ3
BlastRules:WP_003233588, GO:0003677, GO:0005737, KEGG:K26739, RefSeq:WP_014113089.1, SO:0001217, UniParc:UPI00022BBBCB, UniRef:UniRef100_G4NUJ4, UniRef:UniRef50_P94443, UniRef:UniRef90_P94443
EC:3.1.1.3, GO:0004806, GO:0005576, GO:0016042, GO:0016298, KEGG:K01046, RefSeq:WP_080376109.1, SO:0001217, UniParc:UPI0006A898C1, UniRef:UniRef100_UPI0006A898C1, UniRef:UniRef50_Q79F14, UniRef:U
EC:2.3.-.-, GO:0005886, GO:0009246, GO:0016413, RefSeq:WP_072182496.1, SO:0001217, UniParc:UPI0006A845D7, UniRef:UniRef100_UPI0006A845D7, UniRef:UniRef50_O31559, UniRef:UniRef90_O31559
EC:3.-.-.-, GO:0005737, GO:0008270, GO:0016151, GO:0016765, GO:0016787, SO:0001217, UniRef:UniRef50_O31562, UniRef:UniRef90_O31562
SO:0001217, UniRef:UniRef50_D4G6K4, UniRef:UniRef90_D4G6K4
GO:0003677, GO:0003700, GO:0006355, GO:0006950, SO:0001217, UniRef:UniRef50_O31564, UniRef:UniRef90_O31564
SO:0001217, UniRef:UniRef50_UPI0022825F93, UniRef:UniRef90_UPI0022825F93
GO:0005737, GO:0005886, GO:0030288, GO:0045121, GO:1901678, SO:0001217, UniRef:UniRef50_O31567, UniRef:UniRef90_O31567
GO:0005886, GO:0022857, GO:0033214, SO:0001217, UniRef:UniRef50_O31568, UniRef:UniRef90_O31568
GO:0005886, GO:0022857, GO:0033214, SO:0001217, UniRef:UniRef50_O31569, UniRef:UniRef90_O31569
EC:5.3.3.17, KEGG:K06998, SO:0001217, UniRef:UniRef50_S0NXF1, UniRef:UniRef90_G4NRG1
EC:1.-.-.-, GO:0016491, SO:0001217, UniRef:UniRef50_O31571, UniRef:UniRef90_O31571
SO:0001217, UniRef:UniRef50_O31572, UniRef:UniRef90_O31572
RefSeq:WP_003223230.1, SO:0001217, UniParc:UPI00000600EA, UniRef:UniRef100_O31573, UniRef:UniRef50_O31573, UniRef:UniRef90_O31573
KEGG:K07071, SO:0001217, UniRef:UniRef50_O31574, UniRef:UniRef90_O31574
PFAM:PF21982.2, SO:0001217, UniRef:UniRef50_UPI000AB48F02
GO:0005737, GO:0006282, SO:0001217, UniRef:UniRef50_O31575, UniRef:UniRef90_O31575
RefSeq:WP_014113105.1, SO:0001217, UniParc:UPI00022BBBDB, UniRef:UniRef100_G4NRG6, UniRef:UniRef50_O31576, UniRef:UniRef90_O31576
GO:0005886, GO:0022857, GO:0055085, SO:0001217, UniRef:UniRef50_O31577, UniRef:UniRef90_O31577
GO:0030435, GO:0030436, GO:0042601, KEGG:K06428, RefSeq:WP_003223241.1, SO:0001217, UniParc:UPI00001B091D, UniRef:UniRef100_Q7WY75, UniRef:UniRef50_Q7WY75, UniRef:UniRef90_Q7WY75
RefSeq:WP_014113107.1, SO:0001217, UniParc:UPI00022BBBDD, UniRef:UniRef100_G4NRG9, UniRef:UniRef50_O31578, UniRef:UniRef90_O31578
RefSeq:WP_061188215.1, SO:0001217, UniParc:UPI000772179A, UniRef:UniRef100_UPI000772179A, UniRef:UniRef50_O31579, UniRef:UniRef90_D4G6M0
GO:0005886, SO:0001217, UniRef:UniRef50_O31580, UniRef:UniRef90_O31580
EC:3.-.-.-, GO:0016787, SO:0001217, UniRef:UniRef50_O31581, UniRef:UniRef90_O31581
EC:2.4.-.-, GO:0005886, GO:0016757, KEGG:K25617, RefSeq:WP_003223250.1, SO:0001217, UniParc:UPI0001D28DFB, UniRef:UniRef100_A0A9Q4HJS2, UniRef:UniRef50_Q45539, UniRef:UniRef90_Q45539
KEGG:K25619, SO:0001217, UniRef:UniRef50_O31582, UniRef:UniRef90_A0A9W5LGU7
GO:0005886, KEGG:K07038, RefSeq:WP_003223253.1, SO:0001217, UniParc:UPI0001D28DFD, UniRef:UniRef100_A0A9Q4DQ11, UniRef:UniRef50_O31583, UniRef:UniRef90_O31583
EC:3.2.2.31, GO:0000701, GO:0006284, GO:0006298, GO:0019104, GO:0032357, GO:0034039, GO:0035485, GO:0046872, GO:0051539, KEGG:K03575, SO:0001217, UniRef:UniRef50_P83847, UniRef:UniRef90_O31584
RefSeq:WP_003223257.1, SO:0001217, UniParc:UPI0001D28DFF, UniRef:UniRef100_A0A9Q4HJQ0, UniRef:UniRef50_O31585, UniRef:UniRef90_O31585
COG:COG1028, COG:I, EC:1.3.1.104, GO:0004318, GO:0016616, GO:0030497, GO:0050661, GO:0141148, RefSeq:WP_072182515.1, SO:0001217, UniParc:UPI0006A81C57, UniRef:UniRef100_UPI0006A81C57, UniRef:Uni
GO:0030435, RefSeq:WP_014113114.1, SO:0001217, UniParc:UPI00000BA209, UniRef:UniRef100_Q9AH72, UniRef:UniRef50_P84585, UniRef:UniRef90_P84585
RefSeq:WP_014113115.1, SO:0001217, UniParc:UPI00022BA401, UniRef:UniRef100_A0A9Q4DR98, UniRef:UniRef50_Q65M91, UniRef:UniRef90_E0TWC4
EC:3.6.1.15, EC:3.6.1.6, GO:0000287, GO:0017110, GO:0017111, KEGG:K07586, RefSeq:WP_003223272.1, SO:0001217, UniParc:UPI000195C540, UniRef:UniRef100_Q796Z1, UniRef:UniRef50_Q2G2M8, UniRef:UniRef90_
EC:7.6.2.-, GO:0005524, GO:0005886, GO:0016887, GO:0042626, GO:0055085, GO:0140359, SO:0001217, UniRef:UniRef50_P71082, UniRef:UniRef90_P71082
SO:0001217, UniRef:UniRef50_P71083, UniRef:UniRef90_A0A6H0WF12
SO:0001217, UniRef:UniRef50_W4VP23
SO:0001217, UniRef:UniRef50_A0A3D1BPD3, UniRef:UniRef90_UPI002E24BEB0
EC:1.11.1.24, GO:0005737, GO:0008379, GO:0016209, GO:0016491, GO:0034599, GO:0045454, KEGG:K03564, RefSeq:WP_003223280.1, SO:0001217, UniParc:UPI0001D28E04, UniRef:UniRef100_G4NRI5, UniRef:UniRef
BlastRules:WP_000110011, BlastRules:WP_002858684, BlastRules:WP_002987920, BlastRules:WP_003223285, GO:0000976, GO:0001216, GO:0001217, GO:0003700, GO:0005737, GO:0008270, GO:0032993, GO:004589
GO:0005886, RefSeq:WP_014113119.1, SO:0001217, UniParc:UPI0001F5B440, UniRef:UniRef100_A0A2M8SXA6, UniRef:UniRef50_Q7WY74, UniRef:UniRef90_Q7WY74
SO:0001217, UniRef:UniRef50_Q04385, UniRef:UniRef90_Q04385
RFAM:RF01690, SO:0000655
GO:0003735, GO:0005840, KEGG:K01977, RFAM:RF00177, SO:0001000
GO:0003735, GO:0005840, KEGG:K01980, RFAM:RF02541, SO:0001001
GO:0003735, GO:0005840, KEGG:K01985, RFAM:RF00001, SO:0000652
SO:0000257
SO:0000269
SO:0000259
SO:0000273
SO:0000266
SO:0000256
SO:0000267
SO:0000270
SO:0000272
SO:0000271
SO:0000262
SO:0000261
SO:0000260
SO:0000258
SO:0000264
SO:0000264
BlastRules:WP_003233481, GO:0030435, KEGG:K06377, RefSeq:WP_088679604.1, SO:0001217, UniParc:UPI000B5336BF, UniRef:UniRef100_UPI000B5336BF, UniRef:UniRef50_P71088, UniRef:UniRef90_P71088
RefSeq:WP_003224005.1, SO:0001217, UniParc:UPI0001D28843, UniRef:UniRef100_A0A9Q4HAM0, UniRef:UniRef50_O31586, UniRef:UniRef90_O31586
RefSeq:WP_041520898.1, SO:0001217, UniParc:UPI0005A0151E, UniRef:UniRef100_UPI0005A0151E, UniRef:UniRef50_E0TWD6, UniRef:UniRef90_A0A9Q4HI05
RefSeq:WP_088679603.1, SO:0001217, UniParc:UPI000B52B160, UniRef:UniRef100_UPI000B52B160, UniRef:UniRef50_P71089, UniRef:UniRef90_G4NRJ2
RFAM:RF00059, SO:0000035
EC:4.1.99.17, GO:0005829, GO:0008270, GO:0009228, GO:0009229, GO:0016830, GO:0051536, GO:0051539, SO:0001217, UniRef:UniRef50_Q82FI7, UniRef:UniRef90_A7Z2P5
SO:0001217, UniRef:UniRef50_A0A1V6E4U1
SO:0001217, UniParc:UPI0001D28847, UniRef:UniRef100_E0TWD9, UniRef:UniRef50_P21344, UniRef:UniRef90_E0TWD9
BlastRules:WP_003245322, EC:1.11.1.6, GO:0004096, GO:0005737, GO:0020037, GO:0042542, GO:0042744, GO:0046872, RefSeq:WP_003239645.1, SO:0001217, UniParc:UPI00022BA40D, UniRef:UniRef100_A0A9W5LI
EC:7.6.2.14, GO:0005524, GO:0005886, GO:0016887, SO:0001217, UniRef:UniRef50_P97027, UniRef:UniRef90_P97027
GO:0005886, GO:0042626, SO:0001217, UniRef:UniRef50_P40400, UniRef:UniRef90_P40400
SO:0001217, UniRef:UniRef50_P40401, UniRef:UniRef90_UPI0028FA90CC
EC:1.14.14.5, GO:0006790, GO:0008726, GO:0010181, GO:0046306, KEGG:K04091, RefSeq:WP_087987984.1, SO:0001217, UniParc:UPI000B44C2D0, UniRef:UniRef100_UPI000B44C2D0, UniRef:UniRef50_P40402, UniRe
SO:0001217, UniRef:UniRef50_P97028, UniRef:UniRef90_UPI000B52CBAC
GO:0003735, GO:0005737, GO:0005840, GO:0006412, GO:0015935, GO:0019843, KEGG:K02954, RefSeq:WP_061188229.1, SO:0001217, UniParc:UPI0006A8414D, UniRef:UniRef100_A0A9Q4E4V0, UniRef:UniRef50_O31
GO:0005886, RefSeq:WP_014113134.1, SO:0001217, UniParc:UPI00022BA414, UniRef:UniRef100_G4NRK3, UniRef:UniRef50_P97029, UniRef:UniRef90_P97029
GO:0003677, SO:0001217, UniParc:UPI00022BA415, UniRef:UniRef100_G4NRK4, UniRef:UniRef50_C0H3X9, UniRef:UniRef90_C0H3X9
SO:0000260
RefSeq:WP_088679599.1, SO:0001217, UniParc:UPI000B53126E, UniRef:UniRef100_UPI000B53126E, UniRef:UniRef50_O31588, UniRef:UniRef90_O31588
SO:0001217, UniRef:UniRef50_A0A5S9M5G0, UniRef:UniRef90_A0A5S9M5G0
SO:0001217, UniRef:UniRef50_A0A239YUP5
SO:0001217, UniRef:UniRef50_O31589, UniRef:UniRef90_O31589
EC:2.1.1.207, GO:0002130, GO:0003723, GO:0005737, GO:0006400, GO:0008173, GO:0008175, GO:0141098, GO:0141102, KEGG:K03216, SO:0001217, UniParc:UPI0001D28912, UniRef:UniRef100_A0A9W5LIH0, UniRef:
RefSeq:WP_014113139.1, SO:0001217, UniParc:UPI00022BA419, UniRef:UniRef100_G4NRK9, UniRef:UniRef50_P97032, UniRef:UniRef90_D4G6R2
RefSeq:WP_061188236.1, SO:0001217, UniParc:UPI0007725C02, UniRef:UniRef100_UPI0007725C02, UniRef:UniRef50_P39132, UniRef:UniRef90_P39132
RefSeq:WP_088679598.1, SO:0001217, UniParc:UPI000B533379, UniRef:UniRef100_UPI000B533379, UniRef:UniRef50_P39133, UniRef:UniRef90_A0A2M8T5Z0
BlastRules:WP_003233433, COG:COG2766, COG:T, GO:0004672, KEGG:K07180, RefSeq:WP_003224051.1, SO:0001217, UniParc:UPI0001D28916, UniRef:UniRef100_A0A9Q4DSG1, UniRef:UniRef50_P39134, UniRef:UniF
GO:0030436, KEGG:K09786, SO:0001217, UniRef:UniRef50_P45742, UniRef:UniRef90_P45742
GO:0003677, GO:0003700, GO:0006355, GO:0006950, RefSeq:WP_014113144.1, SO:0001217, UniParc:UPI00022BA41E, UniRef:UniRef100_G4NUJ8, UniRef:UniRef50_O31592, UniRef:UniRef90_O31592
GO:0005886, SO:0001217, UniRef:UniRef50_O31593, UniRef:UniRef90_O31593
KEGG:K03446, SO:0001217, UniRef:UniRef50_P54585, UniRef:UniRef90_UPI001012BEFC
GO:0003955, GO:0010181, GO:0016020, RefSeq:WP_014113147.1, SO:0001217, UniParc:UPI00022BA589, UniRef:UniRef100_G4NUK1, UniRef:UniRef50_P54586, UniRef:UniRef90_P54586
GO:0016020, RefSeq:WP_061188238.1, SO:0001217, UniParc:UPI0006A84739, UniRef:UniRef100_UPI0006A84739, UniRef:UniRef50_P54587, UniRef:UniRef90_P54587
RefSeq:WP_014113149.1, SO:0001217, UniParc:UPI00022BA58B, UniRef:UniRef100_G4NUK3, UniRef:UniRef50_P54589, UniRef:UniRef90_A0A2M8T681

GO:0003677, GO:0003700, KEGG:K07979, RefSeq:WP_014113150.1, SO:0001217, UniParc:UPI00022BA58C, UniRef:UniRef100_G4NUK4, UniRef:UniRef50_P54590, UniRef:UniRef90_P54590
GO:0005524, GO:0016887, RefSeq:WP_014113151.1, SO:0001217, UniParc:UPI00022BA58D, UniRef:UniRef100_G4NUK5, UniRef:UniRef50_P54591, UniRef:UniRef90_P54591
SO:0001217, UniRef:UniRef50_A0A0C2SHD1
GO:0005886, GO:0140359, SO:0001217, UniRef:UniRef50_P54593, UniRef:UniRef90_P54593
BlastRules:WP_000066484, BlastRules:WP_001161732, BlastRules:WP_001179150, BlastRules:WP_003224086, BlastRules:WP_123282393, COG:COG1278, COG:K, GO:0003676, GO:0003677, GO:0005737, GO:0009295,
GO:0005886, KEGG:K02073, RefSeq:WP_061188244.1, SO:0001217, UniParc:UPI0006A9295B, UniRef:UniRef100_UPI0006A9295B, UniRef:UniRef50_P54594, UniRef:UniRef90_P54594
GO:0000155, GO:0005886, GO:0043709, GO:0052621, GO:0071555, GO:1902201, RefSeq:WP_079288693.1, SO:0001217, UniParc:UPI0006A90871, UniRef:UniRef100_UPI0006A90871, UniRef:UniRef50_P54595, UniRef:U
GO:0005886, GO:0015184, GO:0015293, RefSeq:WP_072182539.1, SO:0001217, UniParc:UPI0006A886F8, UniRef:UniRef100_UPI0006A886F8, UniRef:UniRef50_P54596, UniRef:UniRef90_P54596
SO:0001217, UniRef:UniRef50_P54597, UniRef:UniRef90_E0TWZ3
KEGG:K06345, SO:0001217, UniRef:UniRef50_P54598, UniRef:UniRef90_G4NUL3
RefSeq:WP_061188249.1, SO:0001217, UniParc:UPI0007723C8C, UniRef:UniRef100_A0A9Q4E684, UniRef:UniRef50_P54599, UniRef:UniRef90_A0A5D4N0A3
GO:0030435, RefSeq:WP_041520902.1, SO:0001217, UniParc:UPI0005A0563A, UniRef:UniRef100_A0A9Q4E7Y9, UniRef:UniRef50_P54601, UniRef:UniRef90_P54601
SO:0001217, UniRef:UniRef50_P54602, UniRef:UniRef90_A0AA96ZPS6
PFAM:PF00746.26
EC:3.4.22.-, GO:0005886, GO:0006508, GO:0008234, SO:0001217, UniRef:UniRef50_P54603, UniRef:UniRef90_P54603
EC:5.4.99.23, KEGG:K06180, SO:0001217, UniRef:UniRef50_P54604, UniRef:UniRef90_A0A9W5LI91
RefSeq:WP_087987999.1, SO:0001217, UniParc:UPI00086E39DA, UniRef:UniRef100_UPI00086E39DA, UniRef:UniRef50_P54605, UniRef:UniRef90_P54605
RefSeq:WP_014113165.1, SO:0001217, UniParc:UPI00022BA59B, UniRef:UniRef100_G4NUM0, UniRef:UniRef50_P54606, UniRef:UniRef90_P54606
EC:3.1.3.-, GO:0016787, GO:0046872, RefSeq:WP_014113166.1, SO:0001217, UniParc:UPI00022BA59C, UniRef:UniRef100_G4NUM1, UniRef:UniRef50_P54607, UniRef:UniRef90_P54607
SO:0001217, UniRef:UniRef50_P54608
SO:0001217, UniRef:UniRef50_UPI002A4E11BB
SO:0001217, UniRef:UniRef50_A0A836MRZ0
EC:2.6.-.-, GO:0004015, GO:0006071, GO:0009102, GO:0030170, KEGG:K03851, SO:0001217, UniRef:UniRef50_P33189, UniRef:UniRef90_P33189
BlastRules:WP_000394287, BlastRules:WP_003233388, GO:0001072, GO:0003723, GO:0006071, GO:0031564, GO:0045893, GO:0046677, GO:0060567, GO:0071333, KEGG:K02443, RefSeq:WP_072182531.1, SO:000121
BlastRules:WP_001272201, BlastRules:WP_003233386, GO:0005886, GO:0015254, GO:0015793, RefSeq:WP_014113170.1, SO:0001217, UniParc:UPI00022BA5A0, UniRef:UniRef100_G4NUM6, UniRef:UniRef50_P18156, U
EC:2.7.1.30, GO:0004370, GO:0005524, GO:0005829, GO:0006071, GO:0006072, GO:0019563, RefSeq:WP_072182530.1, SO:0001217, UniParc:UPI0006A83A69, UniRef:UniRef100_UPI0006A83A69, UniRef:UniRef50_P181
BlastRules:WP_003233382, EC:1.1.5.3, GO:0004368, GO:0005737, GO:0019563, GO:0046168, KEGG:K00111, SO:0001217, UniRef:UniRef50_P18158, UniRef:UniRef90_P18158
SO:0001217, UniRef:UniRef50_A0A4J1Z7Z4
SO:0001217, UniRef:UniRef50_A0A1I0TU95
EC:2.7.13.3, GO:0000155, GO:0005524, GO:0016020, GO:0046983, RefSeq:WP_326198534.1, SO:0001217, UniParc:UPI002DBE1C0F, UniRef:UniRef100_UPI002DBE1C0F, UniRef:UniRef50_O07527, UniRef:UniRef90_O075
GO:0000160, GO:0003677, GO:0005737, GO:0006355, RefSeq:WP_088679566.1, SO:0001217, UniParc:UPI000B5343C0, UniRef:UniRef100_UPI000B5343C0, UniRef:UniRef50_O07528, UniRef:UniRef90_O07528
EC:1.7.-.-, GO:0005829, GO:0010181, GO:0016491, GO:0042802, KEGG:K03206, RefSeq:WP_088679565.1, SO:0001217, UniParc:UPI000B52C0CA, UniRef:UniRef100_UPI000B52C0CA, UniRef:UniRef50_O07529, UniRef:U
RefSeq:WP_003224139.1, SO:0001217, UniParc:UPI0001D289ED, UniRef:UniRef100_A0A9Q4E4X9, UniRef:UniRef50_O07530, UniRef:UniRef90_O07530
SO:0001217, UniRef:UniRef50_O07531, UniRef:UniRef90_O07531
SO:0001217, UniRef:UniRef50_O07532, UniRef:UniRef90_UPI000B445574
BlastRules:WP_003180105, BlastRules:WP_003245378, BlastRules:WP_010897227, BlastRules:WP_011031657, BlastRules:WP_011064694, BlastRules:WP_011231230, GO:0003677, GO:0003700, GO:0005829, GO:000635
GO:0008381, GO:0030435, SO:0001217, UniRef:UniRef50_P37874, UniRef:UniRef90_P37874
BlastRules:WP_001202722, BlastRules:WP_003233364, GO:0030435, KEGG:K06415, SO:0001217, UniRef:UniRef50_P37875, UniRef:UniRef90_P37875
BlastRules:WP_010886458, EC:3.1.3.1, GO:0004035, GO:0046872, KEGG:K01077, SO:0001217, UniRef:UniRef50_P19406, UniRef:UniRef90_P19406
RefSeq:WP_014113182.1, SO:0001217, UniParc:UPI00022BA714, UniRef:UniRef100_G4NUN9, UniRef:UniRef50_A0A0M2PCH6, UniRef:UniRef90_G4NUN9
BlastRules:WP_003233357, GO:0000976, GO:0003700, GO:0005737, GO:0006355, KEGG:K19242, RefSeq:WP_014113183.1, SO:0001217, UniParc:UPI00022BA715, UniRef:UniRef100_G4NUP0, UniRef:UniRef50_P39127,
BlastRules:WP_003244745, EC:2.3.3.16, GO:0004108, GO:0005975, GO:0006099, SO:0001217, UniRef:UniRef50_P39119, UniRef:UniRef90_P39119
EC:1.-.-.-, GO:0016614, RefSeq:WP_014113185.1, SO:0001217, UniParc:UPI00022BA717, UniRef:UniRef100_G4NUP2, UniRef:UniRef50_P80873, UniRef:UniRef90_O07575
BlastRules:WP_003245791, RefSeq:WP_014113186.1, SO:0001217, UniParc:UPI00022BA718, UniRef:UniRef100_G4NUP3, UniRef:UniRef50_A0A1V5MAL4, UniRef:UniRef90_I2C300
GO:0005886, GO:0015293, SO:0001217, UniRef:UniRef50_O34383, UniRef:UniRef90_O07577
KEGG:K00375, SO:0001217, UniRef:UniRef50_P96669, UniRef:UniRef90_G4NUP6
GO:0016747, SO:0001217, UniRef:UniRef50_O07579, UniRef:UniRef90_O07579
GO:0005886, RefSeq:WP_003224169.1, SO:0001217, UniParc:UPI00001AA1B7, UniRef:UniRef100_Q7X2K9, UniRef:UniRef50_O07580, UniRef:UniRef90_O07580
GO:0016020, SO:0001217, UniParc:UPI00022BA71D, UniRef:UniRef100_G4NUP9, UniRef:UniRef50_O07581, UniRef:UniRef90_O07581
BlastRules:WP_003224177, COG:COG1595, COG:K, GO:0003677, GO:0006352, GO:0006355, GO:0016987, RefSeq:WP_003224177.1, SO:0001217, UniParc:UPI000006014D, UniRef:UniRef100_O07582, UniRef:UniRef50_(
SO:0001217, UniRef:UniRef50_A0A5J4JL41
EC:2.3.1.n4, GO:0003841, GO:0005886, GO:0006654, RefSeq:WP_003224182.1, SO:0001217, UniParc:UPI0001D289FD, UniRef:UniRef100_A0A9Q4E5Z1, UniRef:UniRef50_O07584, UniRef:UniRef90_O07584
SO:0001217, UniRef:UniRef50_O32159, UniRef:UniRef90_G4NUQ3
SO:0001217, UniRef:UniRef50_A0AAC9WH94, UniRef:UniRef90_A0A9Q4E847
GO:0005886, GO:0050660, KEGG:K03699, SO:0001217, UniRef:UniRef50_P54428, UniRef:UniRef90_O07585
GO:0003677, GO:0003700, KEGG:K11923, SO:0001217, UniParc:UPI00022BA721, UniRef:UniRef100_G4NUQ6, UniRef:UniRef50_O07586, UniRef:UniRef90_O07586
SO:0001217, UniRef:UniRef50_D4G6Z4, UniRef:UniRef90_D4G6Z4
GO:0005886, GO:0050660, KEGG:K03699, SO:0001217, UniRef:UniRef50_P54428, UniRef:UniRef90_O07589
GO:0016020, GO:1903424, GO:1903425, RefSeq:WP_061188269.1, SO:0001217, UniParc:UPI0006A93715, UniRef:UniRef100_A0A9Q4HJ71, UniRef:UniRef50_O07590, UniRef:UniRef90_G4NUR0
RefSeq:WP_072182721.1, SO:0001217, UniParc:UPI0006A90C60, UniRef:UniRef100_UPI0006A90C60, UniRef:UniRef50_O07591, UniRef:UniRef90_E0TXS3
EC:3.1.4.46, GO:0006071, GO:0006629, GO:0008889, GO:0046872, KEGG:K01126, RefSeq:WP_332606712.1, SO:0001217, UniParc:UPI002FC784B0, UniRef:UniRef100_UPI002FC784B0, UniRef:UniRef50_O07592, UniRef:
RefSeq:WP_003224203.1, SO:0001217, UniParc:UPI0001D28A07, UniRef:UniRef100_A0A9Q4EB82, UniRef:UniRef50_Q62XA8, UniRef:UniRef90_A0AAE3WJG9
BlastRules:WP_003245529, GO:0005886, GO:0055085, KEGG:K16052, RefSeq:WP_072182723.1, SO:0001217, UniParc:UPI0006A85F22, UniRef:UniRef100_A0A9Q4E8B6, UniRef:UniRef50_O07594, UniRef:UniRef90_O075
EC:2.3.1.286, GO:0004407, GO:0005737, GO:0008270, GO:0032041, GO:0040029, GO:0046969, GO:0046970, GO:0070403, GO:0097372, GO:0140765, GO:0141222, SO:0001217, UniRef:UniRef50_O07595, UniRef:UniRef
EC:3.-.-.-, GO:0005886, GO:0005975, GO:0016810, SO:0001217, UniRef:UniRef50_O07596, UniRef:UniRef90_O07596
EC:2.6.1.21, GO:0005829, GO:0019478, GO:0019480, GO:0019752, GO:0030170, GO:0046416, GO:0046437, GO:0047810, KEGG:K00824, RefSeq:WP_072182726.1, SO:0001217, UniParc:UPI0006A81CBE, UniRef:UniRef1
GO:0005886, GO:0006814, GO:0015297, GO:0015385, GO:0016020, GO:1902600, KEGG:K03315, SO:0001217, UniRef:UniRef50_O07553, UniRef:UniRef90_O07553
RefSeq:WP_014113208.1, SO:0001217, UniParc:UPI00022BA72E, UniRef:UniRef100_G4NUR9, UniRef:UniRef50_O07552, UniRef:UniRef90_O07552
BlastRules:WP_003245157, EC:7.6.2.-, GO:0005524, GO:0005886, GO:0016887, GO:0042626, GO:0046677, GO:0055085, GO:0140359, RefSeq:WP_014113210.1, SO:0001217, UniParc:UPI00022BA730, UniRef:UniRef100_
SO:0001217, UniRef:UniRef50_A0A1W7ACC8
KEGG:K07118, SO:0001217, UniRef:UniRef50_O07548, UniRef:UniRef90_UPI000308FB91
GO:0003690, GO:0006265, GO:0030435, RefSeq:WP_003233287.1, SO:0001217, UniParc:UPI000004AB07, UniRef:UniRef100_P04832, UniRef:UniRef50_P04833, UniRef:UniRef90_P04832
SO:0001217, UniParc:UPI0001D28AA0, UniRef:UniRef100_A0A9Q4DL68, UniRef:UniRef50_O07546, UniRef:UniRef90_O07546
GO:0016020, GO:0030435, GO:0042601, SO:0001217, UniRef:UniRef50_O07545, UniRef:UniRef90_O07545
GO:0003824, GO:0005524, GO:0016020, GO:0030435, SO:0001217, UniRef:UniRef50_O07544, UniRef:UniRef90_O07544
SO:0001217, UniRef:UniRef50_Q65LU9, UniRef:UniRef90_UPI0020903BEF
RefSeq:WP_014113218.1, SO:0001217, UniParc:UPI00022BA8AD, UniRef:UniRef100_G4NRL8, UniRef:UniRef50_O07542, UniRef:UniRef90_O07542
SO:0001217, UniRef:UniRef50_O07541, UniRef:UniRef90_O07541
EC:3.1.3.104, GO:0000287, GO:0005829, GO:0016791, KEGG:K21064, RefSeq:WP_014113220.1, SO:0001217, UniParc:UPI00022BA8AF, UniRef:UniRef100_G4NRM0, UniRef:UniRef50_O07539, UniRef:UniRef90_O07539
EC:1.3.99.-, GO:0003824, GO:0005737, GO:0006779, GO:0006782, GO:0016491, GO:0046872, GO:0051536, GO:0051539, RefSeq:WP_014113221.1, SO:0001217, UniParc:UPI00022BA8B0, UniRef:UniRef100_G4NRM1, U
BlastRules:WP_003233272, KEGG:K26732, RefSeq:WP_063334972.1, SO:0001217, UniParc:UPI0006A8D2D4, UniRef:UniRef100_UPI0006A8D2D4, UniRef:UniRef50_O07536, UniRef:UniRef90_A0A081LAT2
BlastRules:WP_003233269, SO:0001217, UniRef:UniRef50_O07535, UniRef:UniRef90_G4NRM3
GO:0005886, KEGG:K26733, SO:0001217, UniRef:UniRef50_O07534, UniRef:UniRef90_O07534
EC:4.2.1.17, GO:0003824, GO:0005886, SO:0001217, UniRef:UniRef50_O07533, UniRef:UniRef90_O07533
RefSeq:WP_061188280.1, SO:0001217, UniParc:UPI0006A8FB70, UniRef:UniRef100_UPI0006A8FB70, UniRef:UniRef50_C0H3Y1, UniRef:UniRef90_E0TXV0
GO:0005524, GO:0005886, GO:0016887, RefSeq:WP_072182740.1, SO:0001217, UniParc:UPI0006A88F3B, UniRef:UniRef100_A0A9Q4HNB9, UniRef:UniRef50_C0SPB4, UniRef:UniRef90_C0SPB4
GO:0005886, GO:0140359, SO:0001217, UniRef:UniRef50_O07523, UniRef:UniRef90_O07523
SO:0001217, UniRef:UniRef50_O07522, UniRef:UniRef90_A0A5D4N2H6
GO:0005886, SO:0001217, UniRef:UniRef50_O08455, UniRef:UniRef90_O08455
COG:COG3481, COG:J, EC:3.1.-.-, GO:0000175, GO:0003677, GO:0031125, KEGG:K03698, RefSeq:WP_061188283.1, SO:0001217, UniParc:UPI0006A81F54, UniRef:UniRef100_A0A9Q4E8J9, UniRef:UniRef50_O07521, Ur
BlastRules:WP_003233252, GO:0005886, GO:0030435, RefSeq:WP_061188284.1, SO:0001217, UniParc:UPI0006A804B6, UniRef:UniRef100_UPI0006A804B6, UniRef:UniRef50_O07520, UniRef:UniRef90_O07520
EC:5.2.1.8, GO:0005886, GO:0006457, GO:0015031, GO:0045121, GO:0140839, GO:0140840, RefSeq:WP_061188285.1, SO:0001217, UniParc:UPI0006A86FA1, UniRef:UniRef100_A0A9Q4E894, UniRef:UniRef50_P24327,
GO:0016020, GO:0030435, RefSeq:WP_003327753.1, SO:0001217, UniParc:UPI000195C5B1, UniRef:UniRef100_C0H3Y2, UniRef:UniRef50_C0H3Y2, UniRef:UniRef90_C0H3Y2
GO:0016020, GO:0030435, RefSeq:WP_003155248.1, SO:0001217, UniParc:UPI0002458BAA, UniRef:UniRef100_L8EBJ6, UniRef:UniRef50_L8EBJ6, UniRef:UniRef90_L8EBJ6
GO:0005886, RefSeq:WP_019713826.1, SO:0001217, UniParc:UPI000318C411, UniRef:UniRef100_UPI000318C411, UniRef:UniRef50_O07518, UniRef:UniRef90_O07518
SO:0001217, UniRef:UniRef50_O07517, UniRef:UniRef90_O07517
GO:0003677, GO:0003700, GO:0006355, GO:0006950, GO:0030435, GO:0045892, KEGG:K09682, RefSeq:WP_014113235.1, SO:0001217, UniParc:UPI00022BA8BF, UniRef:UniRef100_G4NRN8, UniRef:UniRef50_P11065, U
GO:0005886, SO:0001217, UniRef:UniRef50_O07516, UniRef:UniRef90_O07516
GO:0005886, SO:0001217, UniRef:UniRef50_C0H3Y3, UniRef:UniRef90_C0H3Y3
BlastRules:WP_003233236, GO:0005886, GO:0006865, RefSeq:WP_003224283.1, SO:0001217, UniParc:UPI0001D28ABB, UniRef:UniRef100_G4NRP1, UniRef:UniRef50_O07515, UniRef:UniRef90_O07515
EC:2.6.1.52, GO:0003824, GO:0004648, GO:0005737, GO:0006564, GO:0030170, KEGG:K00831, SO:0001217, UniRef:UniRef50_Q59196, UniRef:UniRef90_P80862
GO:0003824, GO:0009117, RefSeq:WP_003224286.1, SO:0001217, UniParc:UPI0001D28ABD, UniRef:UniRef100_G4NRP3, UniRef:UniRef50_O07513, UniRef:UniRef90_O07513
BlastRules:WP_001292599, BlastRules:WP_003233230, GO:0005524, GO:0016887, GO:0030420, GO:0030435, SO:0001217, UniRef:UniRef50_P55339, UniRef:UniRef90_P55339
SO:0001217, UniRef:UniRef50_UPI001F60421C
GO:0005886, GO:0030420, GO:0030435, KEGG:K25157, SO:0001217, UniRef:UniRef50_P55340, UniRef:UniRef90_P55340
KEGG:K25159, RefSeq:WP_326198062.1, SO:0001217, UniParc:UPI002DB5D314, UniRef:UniRef100_UPI002DB5D314, UniRef:UniRef50_P55341, UniRef:UniRef90_P55341
BlastRules:WP_003245681, EC:3.5.1.-, GO:0016787, GO:0046872, KEGG:K01436, SO:0001217, UniRef:UniRef50_O07598, UniRef:UniRef90_O07598
GO:0005886, SO:0001217, UniRef:UniRef50_O07599, UniRef:UniRef90_O07599
BlastRules:WP_003224300, EC:1.14.14.18, GO:0004392, GO:0005737, GO:0046872, SO:0001217, UniRef:UniRef50_P38049, UniRef:UniRef90_P38049
EC:2.4.99.28, EC:3.4.16.4, GO:0005886, GO:0006508, GO:0008360, GO:0008658, GO:0008955, GO:0009002, GO:0009252, GO:0030288, GO:0071555, KEGG:K12555, RefSeq:WP_088679527.1, SO:0001217, UniParc:UPI0
EC:4.1.1.37, GO:0004853, GO:0005829, GO:0006779, GO:0006782, GO:0006783, KEGG:K01599, RefSeq:WP_003224305.1, SO:0001217, UniParc:UPI0001D28B72, UniRef:UniRef100_G4NRQ3, UniRef:UniRef50_P32395, U
EC:4.98.1.1, EC:4.99.1.9, GO:0004325, GO:0005737, GO:0006779, GO:0006783, GO:0046872, KEGG:K01772, SO:0001217, UniRef:UniRef50_P32396, UniRef:UniRef90_P32396
EC:1.3.3.15, EC:1.3.3.4, GO:0004729, GO:0005737, GO:0005886, GO:0006779, GO:0006783, GO:0016491, RefSeq:WP_014113247.1, SO:0001217, UniParc:UPI00022BAA2B, UniRef:UniRef100_G4NRQ6, UniRef:UniRef50_
GO:0003677, SO:0001217, UniRef:UniRef50_P32398, UniRef:UniRef90_P32398
GO:0005886, GO:0140359, KEGG:K01421, SO:0001217, UniRef:UniRef50_P32399, UniRef:UniRef90_P32399
BlastRules:WP_003233200, EC:2.3.1.180, EC:2.3.1.300, GO:0004315, GO:0005737, GO:0006633, GO:0033818, GO:0044550, SO:0001217, UniRef:UniRef50_O07600, UniRef:UniRef90_O07600
GO:0005886, RefSeq:WP_014113251.1, SO:0001217, UniParc:UPI00022BAA2F, UniRef:UniRef100_G4NRR0, UniRef:UniRef50_O07601, UniRef:UniRef90_O07601
EC:3.4.11.-, GO:0004177, GO:0006508, GO:0008237, GO:0046872, KEGG:K01269, SO:0001217, UniRef:UniRef50_O07603, UniRef:UniRef90_O07603
RefSeq:WP_014113256.1, SO:0001217, UniParc:UPI00022BAA34, UniRef:UniRef100_G4NRR5, UniRef:UniRef50_O07604, UniRef:UniRef90_O07604
BlastRules:WP_003245579, GO:0005886, GO:0006835, GO:0006865, GO:0015293, GO:0022857, GO:0045121, KEGG:K11102, RefSeq:WP_014113257.1, SO:0001217, UniParc:UPI00022BAA35, UniRef:UniRef100_G4NRR6
EC:3.-.-.-, GO:0042781, GO:0046872, KEGG:K00784, RefSeq:WP_014113259.1, SO:0001217, UniParc:UPI00022BAA37, UniRef:UniRef100_G4NRR8, UniRef:UniRef50_O07607, UniRef:UniRef90_O07607
BlastRules:WP_003244914, EC:6.3.1.20, GO:0005524, GO:0005737, GO:0009249, GO:0016979, GO:0017118, KEGG:K03800, RefSeq:WP_087988034.1, SO:0001217, UniParc:UPI000B441457, UniRef:UniRef100_UPI000B4
EC:4.-.-.-, GO:0016829, RefSeq:WP_072182330.1, SO:0001217, UniParc:UPI0006A92C85, UniRef:UniRef100_UPI0006A92C85, UniRef:UniRef50_O07609, UniRef:UniRef90_O07609
BlastRules:WP_003244686, EC:6.2.1.3, GO:0004467, GO:0005524, GO:0006635, SO:0001217, UniRef:UniRef50_O07610, UniRef:UniRef90_O07610
RefSeq:WP_061188298.1, SO:0001217, UniParc:UPI0006A83FC6, UniRef:UniRef100_UPI0006A83FC6, UniRef:UniRef50_O07611, UniRef:UniRef90_O07611
EC:3.4.24.-, GO:0004222, GO:0046872, GO:0071586, KEGG:K06013, RefSeq:WP_072182328.1, SO:0001217, UniParc:UPI0006A88046, UniRef:UniRef100_UPI0006A88046, UniRef:UniRef50_P40769, UniRef:UniRef90_P407
EC:3.4.21.62, GO:0004252, GO:0005576, GO:0006508, GO:0030435, GO:0046872, SO:0001217, UniRef:UniRef50_P07518, UniRef:UniRef90_P07518
SO:0001217, UniRef:UniRef50_O07614
EC:1.6.5.-, GO:0005737, GO:0043957, RefSeq:WP_072183333.1, SO:0001217, UniParc:UPI0006A8D6BE, UniRef:UniRef100_UPI0006A8D6BE, UniRef:UniRef50_O07615, UniRef:UniRef90_O07615
GO:0005886, GO:0030288, GO:0045121, GO:1901678, SO:0001217, UniRef:UniRef50_C0SP94, UniRef:UniRef90_C0SP94
PFAM:PF00300.27
PFAM:PF00300.27

EC:2.3.1.-, GO:0003988, GO:0006635, GO:0010124, SO:0001217, UniRef:UniRef50_O07618, UniRef:UniRef90_O07618
SO:0001217, UniRef:UniRef50_A0A0D1KSX8, UniRef:UniRef90_A0A0D1KSX8
GO:0005886, GO:0015225, KEGG:K03523, RefSeq:WP_014113274.1, SO:0001217, UniParc:UPI00022BAA46, UniRef:UniRef100_G4NRT4, UniRef:UniRef50_O07620, UniRef:UniRef90_O07620
BlastRules:WP_003245216, GO:0007165, GO:0009454, GO:0016020, GO:0019825, GO:0020037, GO:0046872, SO:0001217, UniRef:UniRef50_O07621, UniRef:UniRef90_O07621
EC:1.-.-.-, GO:0005737, GO:0016020, GO:0016491, GO:0046872, GO:0051537, RefSeq:WP_072183337.1, SO:0001217, UniParc:UPI0006A8B3C6, UniRef:UniRef100_UPI0006A8B3C6, UniRef:UniRef50_O07622, UniRef:Uni
EC:1.-.-.-, GO:0016614, RefSeq:WP_072183338.1, SO:0001217, UniParc:UPI0006A826F1, UniRef:UniRef100_UPI0006A826F1, UniRef:UniRef50_P80873, UniRef:UniRef90_P40397
RefSeq:WP_072183339.1, SO:0001217, UniParc:UPI0006A8EFF7, UniRef:UniRef100_UPI0006A8EFF7, UniRef:UniRef50_O31594, UniRef:UniRef90_O31594
BlastRules:WP_003245329, GO:0003677, GO:0030420, RefSeq:WP_024712928.1, SO:0001217, UniParc:UPI0004675DA4, UniRef:UniRef100_A0A9Q4E5F7, UniRef:UniRef50_P40396, UniRef:UniRef90_P40396
SO:0001217, UniRef:UniRef50_A0A2X3JCS7, UniRef:UniRef90_UPI000575FD56
RefSeq:WP_014113282.1, SO:0001217, UniParc:UPI00022BAA4E, UniRef:UniRef100_G4NRU2, UniRef:UniRef50_O07555, UniRef:UniRef90_O07555
SO:0001217, UniRef:UniRef50_A0A257NXD8
GO:0005886, GO:0015293, GO:0022857, GO:0055085, SO:0001217, UniRef:UniRef50_O07556, UniRef:UniRef90_O07556
GO:0005886, SO:0001217, UniRef:UniRef50_O07557, UniRef:UniRef90_O07557
RefSeq:WP_072183342.1, SO:0001217, UniParc:UPI0006A7F2C6, UniRef:UniRef100_UPI00077299E5, UniRef:UniRef50_O07558, UniRef:UniRef90_O07558
GO:0005886, RefSeq:WP_014113286.1, SO:0001217, UniParc:UPI00022BAA52, UniRef:UniRef100_G4NUS8, UniRef:UniRef50_O07559, UniRef:UniRef90_O07559
EC:3.4.21.89, GO:0004252, GO:0005886, GO:0006465, GO:0009306, GO:0016020, KEGG:K03100, RefSeq:WP_003239429.1, SO:0001217, UniParc:UPI0002ACEBC4, UniRef:UniRef100_A0A9W5PD04, UniRef:UniRef50_O0
PFAM:PF00070.33
SO:0001217, UniRef:UniRef50_UPI002F3F0E04
GO:0003677, GO:0003700, GO:0005737, RefSeq:WP_014113289.1, SO:0001217, UniParc:UPI00022BABB5, UniRef:UniRef100_G4NUT1, UniRef:UniRef50_Q796S4, UniRef:UniRef90_Q796S4
RefSeq:WP_072183343.1, SO:0001217, UniParc:UPI0006A7F991, UniRef:UniRef100_UPI0006A7F991, UniRef:UniRef50_D4FUS6, UniRef:UniRef90_D4FUS6
BlastRules:WP_003245772, GO:0005886, GO:0016020, GO:0022857, RefSeq:WP_014113291.1, SO:0001217, UniParc:UPI00022BABB7, UniRef:UniRef100_G4NUT3, UniRef:UniRef50_O07563, UniRef:UniRef90_O07563
BlastRules:WP_003244701, EC:1.1.1.361, GO:0000166, GO:0005829, GO:0016616, GO:0017000, GO:0102497, GO:0103074, KEGG:K18652, RefSeq:WP_014113292.1, SO:0001217, UniParc:UPI00022BABB8, UniRef:UniRe
BlastRules:WP_003244845, EC:3.1.3.92, GO:0000287, GO:0005829, GO:0016791, GO:0017000, KEGG:K18654, RefSeq:WP_090558615.1, SO:0001217, UniParc:UPI00086B9533, UniRef:UniRef100_UPI00086B9533, UniRe
PFAM:PF01041.23
SO:0001217, UniRef:UniRef50_UPI000A91452D, UniRef:UniRef90_UPI000A91452D
BlastRules:WP_009966948, GO:0000976, GO:0003700, GO:0006355, SO:0001217, UniRef:UniRef50_O07567, UniRef:UniRef90_O07567
GO:0005886, GO:0010468, KEGG:K07120, SO:0001217, UniRef:UniRef50_O07568, UniRef:UniRef90_O07568
SO:0001217, UniRef:UniRef50_O07569, UniRef:UniRef90_A0A6H0WJ46
SO:0001217, UniRef:UniRef50_O07570, UniRef:UniRef90_A0A2M8T5M8
RefSeq:WP_014113300.1, SO:0001217, UniParc:UPI00022BABC0, UniRef:UniRef100_G4NUU2, UniRef:UniRef50_O07572, UniRef:UniRef90_G4NUU2
SO:0001217, UniRef:UniRef50_UPI00227DA006
SO:0001217, UniRef:UniRef50_UPI001D13AA59
PFAM:PF12705.13
SO:0001217, UniRef:UniRef50_A0A1E5LEA8
EC:3.1.-.-, EC:3.6.4.12, EC:5.6.2.4, GO:0000724, GO:0003678, GO:0006302, GO:0016818, KEGG:K16898, SO:0001217, UniRef:UniRef50_UPI001FE7D257, UniRef:UniRef90_UPI002DB972E4
EC:3.1.11.-, GO:0003677, GO:0004519, GO:0004527, GO:0004529, GO:0006260, GO:0006281, GO:0006310, GO:0008408, SO:0001217, UniRef:UniRef50_P23479, UniRef:UniRef90_P23479
SO:0001217, UniRef:UniRef50_UPI00227F1DAA
SO:0001217, UniRef:UniRef50_A0A1L8I811
BlastRules:WP_003245113, RefSeq:WP_014113305.1, SO:0001217, UniParc:UPI00022BABC5, UniRef:UniRef100_G4NUU7, UniRef:UniRef50_O06715, UniRef:UniRef90_O06715
GO:0030435, KEGG:K06304, RefSeq:WP_010333667.1, SO:0001217, UniParc:UPI00022BABC6, UniRef:UniRef100_G4NUU8, UniRef:UniRef50_O06716, UniRef:UniRef90_O06716
KEGG:K06303, RefSeq:WP_041520914.1, SO:0001217, UniParc:UPI00059F03F2, UniRef:UniRef100_A0A9Q4HIT3, UniRef:UniRef50_O06717, UniRef:UniRef90_G4NUU9
BlastRules:WP_001052807, BlastRules:WP_003233093, GO:0030435, RefSeq:WP_014113307.1, SO:0001217, UniParc:UPI00022BABC8, UniRef:UniRef100_G4NUV0, UniRef:UniRef50_O06718, UniRef:UniRef90_O06718
GO:0030435, KEGG:K06301, RefSeq:WP_087988047.1, SO:0001217, UniParc:UPI000B452A06, UniRef:UniRef100_UPI000B452A06, UniRef:UniRef50_O06719, UniRef:UniRef90_O06719
RefSeq:WP_003224438.1, SO:0001217, UniParc:UPI0001D28C6E, UniRef:UniRef100_A0A9Q4HHN1, UniRef:UniRef50_O06720, UniRef:UniRef90_A0A9Q4HHN1
GO:0030435, KEGG:K06299, RefSeq:WP_003239409.1, SO:0001217, UniParc:UPI00022BABCA, UniRef:UniRef100_A0A2M8T5H1, UniRef:UniRef50_O06721, UniRef:UniRef90_O06721
KEGG:K06376, SO:0001217, UniRef:UniRef50_O06722, UniRef:UniRef90_G4NUV4
SO:0001217, UniRef:UniRef50_O06723, UniRef:UniRef90_G4NUV5
EC:5.3.2.2, GO:0005737, GO:0006107, GO:0008948, GO:0018773, GO:0046872, GO:0050163, SO:0001217, UniRef:UniRef50_O06724, UniRef:UniRef90_O06724
GO:0005886, RefSeq:WP_014113313.1, SO:0001217, UniParc:UPI00022BABCF, UniRef:UniRef100_G4NUV8, UniRef:UniRef50_Q65LI4, UniRef:UniRef90_O06725
SO:0001217, UniRef:UniRef50_UPI001F50351C
PFAM:PF00082.27
PFAM:PF17936.7
SO:0001217, UniRef:UniRef50_O06727, UniRef:UniRef90_O06727
EC:6.3.5.4, GO:0004066, GO:0005524, GO:0006529, GO:0030435, GO:0070981, KEGG:K01953, RefSeq:WP_088679509.1, SO:0001217, UniParc:UPI000B538A24, UniRef:UniRef100_UPI000B538A24, UniRef:UniRef50_O05
SO:0001217, UniRef:UniRef50_O06728, UniRef:UniRef90_E0TYU8
GO:0005886, GO:0015297, GO:0042910, RefSeq:WP_014113320.1, SO:0001217, UniParc:UPI00022BABD6, UniRef:UniRef100_G4NUW5, UniRef:UniRef50_O07940, UniRef:UniRef90_O07940
GO:0003700, GO:0043565, RefSeq:WP_061188331.1, SO:0001217, UniParc:UPI0006A90660, UniRef:UniRef100_A0A9Q4E557, UniRef:UniRef50_P40331, UniRef:UniRef90_P40331
BlastRules:WP_003245813, GO:0000976, GO:0003700, GO:0006355, SO:0001217, UniRef:UniRef50_P37947, UniRef:UniRef90_P37947
EC:1.1.1.370, GO:0016616, GO:0019310, GO:0070403, GO:0070404, GO:1902141, SO:0001217, UniRef:UniRef50_P40332, UniRef:UniRef90_P40332
RefSeq:WP_072566070.1, SO:0001217, UniParc:UPI00086EF106, UniRef:UniRef100_A0A9Q4HIS8, UniRef:UniRef50_O07939, UniRef:UniRef90_E0U0Y9
KEGG:K06895, RefSeq:WP_090558633.1, SO:0001217, UniParc:UPI000868A69E, UniRef:UniRef100_UPI000868A69E, UniRef:UniRef50_O06730, UniRef:UniRef90_E0U0Z0
GO:0003677, GO:0003700, GO:0008483, GO:0009058, GO:0030170, GO:1901605, SO:0001217, UniRef:UniRef50_Q796Q6, UniRef:UniRef90_Q796Q6
SO:0001217, UniRef:UniRef50_A0A0D1L8B1, UniRef:UniRef90_A0A0D1L8B1
EC:3.-.-.-, GO:0016020, GO:0016787, RefSeq:WP_014113328.1, SO:0001217, UniParc:UPI00022BAD68, UniRef:UniRef100_G4NUX3, UniRef:UniRef50_O06734, UniRef:UniRef90_O06734
EC:2.7.1.25, GO:0000103, GO:0004020, GO:0005524, GO:0070814, RefSeq:WP_072183104.1, SO:0001217, UniParc:UPI0006A892D9, UniRef:UniRef100_UPI0006A892D9, UniRef:UniRef50_P0A6J1, UniRef:UniRef90_O067
EC:2.7.7.4, GO:0000103, GO:0004781, GO:0005524, GO:0070814, RefSeq:WP_014113330.1, SO:0001217, UniParc:UPI00022BAD6A, UniRef:UniRef100_G4NUX5, UniRef:UniRef50_O34764, UniRef:UniRef90_O06736
EC:1.8.4.10, EC:1.8.4.8, GO:0003824, GO:0004604, GO:0005737, GO:0019344, GO:0019379, GO:0043866, GO:0046872, GO:0051539, GO:0070814, KEGG:K00390, RefSeq:WP_088679507.1, SO:0001217, UniParc:UPI000
EC:3.1.3.71, KEGG:K05979, RefSeq:WP_072183103.1, SO:0001217, UniParc:UPI0006A903B7, UniRef:UniRef100_UPI0006A903B7, UniRef:UniRef50_G4NUX7, UniRef:UniRef90_G4NUX7
SO:0001217, UniRef:UniRef50_O06739
GO:0005886, SO:0001217, UniParc:UPI0001E14EEB, UniRef:UniRef100_E0U0Z9, UniRef:UniRef50_O06740, UniRef:UniRef90_O06740
RefSeq:WP_072183101.1, SO:0001217, UniParc:UPI0006A8261A, UniRef:UniRef100_UPI0006A8261A, UniRef:UniRef50_O06741, UniRef:UniRef90_E0U100
GO:0005886, GO:0022857, GO:0055085, RefSeq:WP_014113335.1, SO:0001217, UniParc:UPI00022BAD6F, UniRef:UniRef100_G4NUY1, UniRef:UniRef50_Q796Q1, UniRef:UniRef90_Q796Q1
RefSeq:WP_014113336.1, SO:0001217, UniParc:UPI00022BAD70, UniRef:UniRef100_G4NUY2, UniRef:UniRef50_O06743, UniRef:UniRef90_G4NUY2
RefSeq:WP_014113337.1, SO:0001217, UniParc:UPI00022BAD71, UniRef:UniRef100_G4NUY3, UniRef:UniRef50_O06744, UniRef:UniRef90_A0A9W5LHW5
SO:0001217, UniRef:UniRef50_A0A368W0W9
SO:0001217, UniRef:UniRef50_W4RLJ2
RFAM:RF00162, SO:0000140
GO:0000166, GO:0005829, KEGG:K09767, RefSeq:WP_014113338.1, SO:0001217, UniParc:UPI00022BAD72, UniRef:UniRef100_G4NUY4, UniRef:UniRef50_Q81GN2, UniRef:UniRef90_O06746
GO:0003676, KEGG:K00243, SO:0001217, UniRef:UniRef50_O06747, UniRef:UniRef90_O06747
RefSeq:WP_014113340.1, SO:0001217, UniParc:UPI00022BAD74, UniRef:UniRef100_G4NUY6, UniRef:UniRef50_O06753, UniRef:UniRef90_G4NUY6
GO:0008289, RefSeq:WP_014113341.1, SO:0001217, UniParc:UPI00022BAD75, UniRef:UniRef100_G4NUY7, UniRef:UniRef50_P70945, UniRef:UniRef90_P70945
GO:0005886, RefSeq:WP_041520917.1, SO:0001217, UniParc:UPI0002F4AF7C, UniRef:UniRef100_A0A9Q4HHM1, UniRef:UniRef50_P39803, UniRef:UniRef90_P39803
GO:0004867, GO:0005737, RefSeq:WP_061188345.1, SO:0001217, UniParc:UPI0006A85F1E, UniRef:UniRef100_A0A9Q4HIQ9, UniRef:UniRef50_P39804, UniRef:UniRef90_P39804
RefSeq:WP_003239348.1, SO:0001217, UniParc:UPI00022BAD78, UniRef:UniRef100_A0A9W5LHV9, UniRef:UniRef50_C0H3Y5, UniRef:UniRef90_C0H3Y5
RefSeq:WP_003239346.1, SO:0001217, UniParc:UPI00022BAD79, UniRef:UniRef100_A0A9W5LHZ8, UniRef:UniRef50_A0A075JL74, UniRef:UniRef90_A0A9Q4EHB4
EC:3.1.3.104, GO:0000287, GO:0005829, GO:0009231, GO:0016791, GO:0043726, KEGG:K21064, RefSeq:WP_072183134.1, SO:0001217, UniParc:UPI0006A8F119, UniRef:UniRef100_UPI0006A8F119, UniRef:UniRef50_P7
EC:3.1.-.-, GO:0006508, GO:0008236, RefSeq:WP_088679506.1, SO:0001217, UniParc:UPI000B5333A6, UniRef:UniRef100_UPI000B5333A6, UniRef:UniRef50_P70948, UniRef:UniRef90_P70948
COG:COG2151, COG:O, RefSeq:WP_003224525.1, SO:0001217, UniParc:UPI0001D28D41, UniRef:UniRef100_A0A9Q4E5C0, UniRef:UniRef50_A0A0H2XI17, UniRef:UniRef90_P70949
RefSeq:WP_003232996.1, SO:0001217, UniParc:UPI00019761F0, UniRef:UniRef100_A0A6M4JH96, UniRef:UniRef50_A0A0J8JMJ3, UniRef:UniRef90_A0A6M4JH96
EC:1.-.-.-, GO:0016491, GO:0016899, GO:0071949, KEGG:K16653, RefSeq:WP_072183136.1, SO:0001217, UniParc:UPI0006A83ABC, UniRef:UniRef100_UPI0006A83ABC, UniRef:UniRef50_Q796P5, UniRef:UniRef90_Q79
GO:0005886, GO:0022857, RefSeq:WP_061188348.1, SO:0001217, UniParc:UPI0006A82A33, UniRef:UniRef100_A0A9Q4E541, UniRef:UniRef50_P70952, UniRef:UniRef90_P70952
EC:1.2.1.38, GO:0003942, GO:0005737, GO:0006526, GO:0006592, GO:0051287, GO:0070401, RefSeq:WP_014113347.1, SO:0001217, UniParc:UPI00022BAD7D, UniRef:UniRef100_G4NUZ7, UniRef:UniRef50_P23715, Un
EC:2.3.1.1, EC:2.3.1.35, GO:0004042, GO:0004358, GO:0005737, GO:0006526, GO:0006592, KEGG:K00620, SO:0001217, UniRef:UniRef50_Q07908, UniRef:UniRef90_P36843
EC:2.7.2.8, GO:0003991, GO:0005524, GO:0005737, GO:0006526, GO:0042450, SO:0001217, UniRef:UniRef50_P68729, UniRef:UniRef90_P68729
EC:2.6.1.11, GO:0003992, GO:0005737, GO:0006525, GO:0006526, GO:0008483, GO:0030170, GO:0042802, SO:0001217, UniRef:UniRef50_Q81M98, UniRef:UniRef90_P36839
EC:6.3.5.5, GO:0004088, GO:0004359, GO:0005524, GO:0005737, GO:0005951, GO:0006207, GO:0006526, GO:0006541, GO:0044205, SO:0001217, UniRef:UniRef50_P36838, UniRef:UniRef90_P36838
EC:6.3.4.16, EC:6.3.5.5, GO:0004087, GO:0004088, GO:0005524, GO:0005737, GO:0006526, GO:0006541, GO:0044205, GO:0046872, SO:0001217, UniRef:UniRef50_P18185, UniRef:UniRef90_P18185
EC:2.1.3.3, GO:0004585, GO:0005737, GO:0006526, GO:0016597, GO:0019240, GO:0042450, KEGG:K00611, RefSeq:WP_061188354.1, SO:0001217, UniParc:UPI0006A8E1EA, UniRef:UniRef100_UPI0006A8E1EA, UniRef:
RefSeq:WP_003224549.1, SO:0001217, UniParc:UPI0001D28E0C, UniRef:UniRef100_A0A9Q4DKM7, UniRef:UniRef50_O34585, UniRef:UniRef90_O34585
GO:0005886, RefSeq:WP_003224551.1, SO:0001217, UniParc:UPI0001D28E0D, UniRef:UniRef100_A0A9Q4E7Q7, UniRef:UniRef50_O34713, UniRef:UniRef90_O34713
RefSeq:WP_014113354.1, SO:0001217, UniParc:UPI00022BAD84, UniRef:UniRef100_G4NWC9, UniRef:UniRef50_O35001, UniRef:UniRef90_O35001
SO:0001217, UniRef:UniRef50_O34959, UniRef:UniRef90_UPI002FC783AE
BlastRules:WP_003245494, GO:0005886, KEGG:K05519, RefSeq:WP_061188356.1, SO:0001217, UniParc:UPI0006A8F708, UniRef:UniRef100_UPI0006A8F708, UniRef:UniRef50_O32436, UniRef:UniRef90_O32436
BlastRules:WP_003224559, GO:0030420, KEGG:K02254, RefSeq:WP_003224559.1, SO:0001217, UniParc:UPI00000601FD, UniRef:UniRef100_O32437, UniRef:UniRef50_O32437, UniRef:UniRef90_O32437
SO:0001217, UniParc:UPI0001D28E11, UniRef:UniRef100_G4NWD3, UniRef:UniRef50_O34891, UniRef:UniRef90_A0A9Q4H8N0
BlastRules:WP_000288132, BlastRules:WP_001100539, BlastRules:WP_003232971, EC:2.3.1.180, EC:2.3.1.300, GO:0004315, GO:0005737, GO:0006633, GO:0033818, GO:0061990, RefSeq:WP_072183381.1, SO:0001217
PFAM:PF00109.32
PFAM:PF02801.28
RefSeq:WP_072183382.1, SO:0001217, UniParc:UPI0006A911AA, UniRef:UniRef100_UPI0006A911AA, UniRef:UniRef50_O31596, UniRef:UniRef90_O31596
BlastRules:WP_003232965, GO:0005524, GO:0005886, GO:0015031, GO:0015833, GO:0016887, GO:0030420, GO:0030435, RefSeq:WP_061188358.1, SO:0001217, UniParc:UPI0006A7FD9C, UniRef:UniRef100_UPI0006A
BlastRules:WP_003232964, GO:0005524, GO:0005886, GO:0015031, GO:0015833, GO:0016887, GO:0030420, GO:0030435, GO:0055085, RefSeq:WP_061188359.1, SO:0001217, UniParc:UPI00077227F3, UniRef:UniRef10
SO:0001217, UniRef:UniRef50_P42061, UniRef:UniRef90_A0A8I2B6J3
RefSeq:WP_014113363.1, SO:0001217, UniParc:UPI00022BAEF6, UniRef:UniRef100_A0A2M8T594, UniRef:UniRef50_P42062, UniRef:UniRef90_A0A5C0WBR3
GO:0043190, GO:0140359, RefSeq:WP_072566092.1, SO:0001217, UniParc:UPI00090C99A5, UniRef:UniRef100_A0A9Q4HIP9, UniRef:UniRef50_P42063, UniRef:UniRef90_A0A410L113
RefSeq:WP_014113365.1, SO:0001217, UniParc:UPI00022BAEF8, UniRef:UniRef100_G4NWE2, UniRef:UniRef50_Q81TS7, UniRef:UniRef90_A7Z3C9
COG:COG0180, COG:J, EC:6.1.1.2, GO:0000166, GO:0004830, GO:0005524, GO:0005829, GO:0006436, SO:0001217, UniRef:UniRef50_P21656, UniRef:UniRef90_P21656
RFAM:RF00230, SO:0000140
SO:0001217, UniRef:UniRef50_A0A5S9MAC8
GO:0015031, GO:0015833, GO:0030420, GO:0030435, GO:0042597, GO:0043190, GO:0045121, GO:1904680, KEGG:K15580, SO:0001217, UniRef:UniRef50_P24141, UniRef:UniRef90_P24141
GO:0005886, GO:0015031, GO:0015833, GO:0030420, GO:0030435, GO:0045121, GO:0055085, SO:0001217, UniRef:UniRef50_P24138, UniRef:UniRef90_P24138
BlastRules:WP_003232954, GO:0005886, GO:0015031, GO:0015833, GO:0030420, GO:0030435, GO:0055085, RefSeq:WP_014113369.1, SO:0001217, UniParc:UPI00022BAEFC, UniRef:UniRef100_G4NWE6, UniRef:UniRe
BlastRules:WP_010886477, EC:7.4.2.6, GO:0005524, GO:0005886, GO:0015031, GO:0015833, GO:0016887, GO:0022857, GO:0030420, GO:0030435, GO:0055085, SO:0001217, UniParc:UPI00022BAEFD, UniRef:UniRef10
BlastRules:WP_003245567, EC:7.4.2.6, GO:0005524, GO:0005886, GO:0015031, GO:0015833, GO:0016887, GO:0030420, GO:0030435, GO:0055085, SO:0001217, UniRef:UniRef50_P24137, UniRef:UniRef90_P24137
SO:0001217, UniRef:UniRef50_O31600, UniRef:UniRef90_E0U148
EC:2.3.1.-, GO:0005737, GO:0016747, RefSeq:WP_003224597.1, SO:0001217, UniParc:UPI0001CE388D, UniRef:UniRef100_A0A9Q4E522, UniRef:UniRef50_O31601, UniRef:UniRef90_O31601
BlastRules:WP_000258267, BlastRules:WP_003245483, GO:0005737, GO:0045892, KEGG:K16509, RefSeq:WP_003239281.1, SO:0001217, UniParc:UPI00022BAF00, UniRef:UniRef100_A0A9W5PCR5, UniRef:UniRef50_O3
GO:0005886, RefSeq:WP_003224601.1, SO:0001217, UniParc:UPI0001D28E23, UniRef:UniRef100_A0A9Q4E781, UniRef:UniRef50_O31603, UniRef:UniRef90_O31603
GO:0030420, GO:0030435, GO:0030674, GO:0042174, GO:0045808, KEGG:K16511, RefSeq:WP_014113374.1, SO:0001217, UniParc:UPI00022BAF02, UniRef:UniRef100_G4NWF3, UniRef:UniRef50_P37958, UniRef:UniRef
KEGG:K06198, RefSeq:WP_072182808.1, SO:0001217, UniParc:UPI0006A80DB6, UniRef:UniRef100_UPI0006A80DB6, UniRef:UniRef50_O31604, UniRef:UniRef90_A0A9W5LHW7
SO:0001217, UniRef:UniRef50_UPI002741D33A
EC:3.4.24.-, GO:0004222, GO:0005737, GO:0006508, GO:0006518, GO:0046872, KEGG:K08602, SO:0001217, UniRef:UniRef50_O31605, UniRef:UniRef90_O31605
GO:0005886, RefSeq:WP_041520918.1, SO:0001217, UniParc:UPI00059FA8A2, UniRef:UniRef100_A0A9Q4HIP3, UniRef:UniRef50_C0H3Y6, UniRef:UniRef90_C0H3Y6
RFAM:RF01820, SO:0000655
BlastRules:WP_003245184, GO:0005737, GO:0046872, SO:0001217, UniRef:UniRef50_O31606, UniRef:UniRef90_O31606

GO:0005344, GO:0019825, GO:0020037, GO:0046872, RefSeq:WP_003224612.1, SO:0001217, UniParc:UPI0001D28F15, UniRef:UniRef100_A0A9Q4E7N8, UniRef:UniRef50_O31607, UniRef:UniRef90_O31607
EC:3.2.1.17, EC:4.2.2.n1, GO:0000270, GO:0005576, GO:0008933, GO:0016020, GO:0016787, GO:0071555, RefSeq:WP_250540712.1, SO:0001217, UniParc:UPI0006A8F4FD, UniRef:UniRef100_UPI0006A8F4FD, UniRef:U
EC:3.6.1.-, GO:0016787, RefSeq:WP_014113380.1, SO:0001217, UniParc:UPI00022BAF08, UniRef:UniRef100_G4NWG0, UniRef:UniRef50_O31609, UniRef:UniRef90_O31609
RefSeq:WP_072182811.1, SO:0001217, UniParc:UPI0006A81773, UniRef:UniRef100_UPI0006A81773, UniRef:UniRef50_O31610, UniRef:UniRef90_O31610
BlastRules:WP_003245294, EC:2.7.6.5, GO:0005524, GO:0005525, GO:0008728, GO:0015970, GO:0016301, GO:0046872, RefSeq:WP_019713982.1, SO:0001217, UniParc:UPI0003690453, UniRef:UniRef100_G4NWG2, Un
EC:2.7.1.23, GO:0003951, GO:0005524, GO:0005737, GO:0006741, GO:0019674, GO:0046872, GO:0051287, KEGG:K00858, RefSeq:WP_003224622.1, SO:0001217, UniParc:UPI0001D28F1A, UniRef:UniRef100_A0A9Q4E
EC:5.4.99.-, GO:0000455, GO:0003723, GO:0009982, GO:0140098, KEGG:K06180, RefSeq:WP_072182812.1, SO:0001217, UniParc:UPI0006A8D7E9, UniRef:UniRef100_UPI0006A8D7E9, UniRef:UniRef50_O31613, UniRef:
EC:3.6.1.17, GO:0004081, GO:0005525, GO:0005737, GO:0016151, GO:0016791, GO:0042763, RefSeq:WP_014113384.1, SO:0001217, UniParc:UPI00022BAF0C, UniRef:UniRef100_G4NWG5, UniRef:UniRef50_O31614, U
GO:0005886, GO:0006813, GO:0008324, GO:0015297, GO:1902600, RefSeq:WP_014113385.1, SO:0001217, UniParc:UPI00022BAF0D, UniRef:UniRef100_G4NWG6, UniRef:UniRef50_O31615, UniRef:UniRef90_O31615
RFAM:RF00059, SO:0000035
EC:3.5.99.2, GO:0005829, GO:0006772, GO:0009228, GO:0009229, GO:0050334, RefSeq:WP_072182813.1, SO:0001217, UniParc:UPI0006A8EC5F, UniRef:UniRef100_UPI0006A8EC5F, UniRef:UniRef50_P25052, UniRef:Ur
EC:5.3.99.10, GO:0004789, GO:0005737, GO:0009228, GO:0009229, GO:0016853, RefSeq:WP_072182825.1, SO:0001217, UniParc:UPI0006A8E3DF, UniRef:UniRef100_UPI0006A8E3DF, UniRef:UniRef50_P25053, UniRef:U
EC:1.4.3.19, GO:0005737, GO:0006520, GO:0009228, GO:0009229, GO:0009635, GO:0016647, GO:0043799, GO:0050660, GO:0071949, RefSeq:WP_242734485.1, SO:0001217, UniParc:UPI001F60FC8F, UniRef:UniRef100
GO:0003824, GO:0009228, SO:0001217, UniParc:UPI00022BAF11, UniRef:UniRef100_A0A9W5LHW0, UniRef:UniRef50_A0A1L3MXR6, UniRef:UniRef90_D4FV42
BlastRules:WP_000931993, BlastRules:WP_003916659, BlastRules:WP_010886483, EC:2.8.1.10, GO:0005737, GO:0009229, GO:1990107, KEGG:K03149, SO:0001217, UniParc:UPI0001D28F22, UniRef:UniRef100_A0A2M
EC:2.7.7.73, GO:0004792, GO:0005524, GO:0005737, GO:0005829, GO:0008146, GO:0008641, GO:0009228, GO:0009229, GO:0016779, GO:0046872, RefSeq:WP_087988077.1, SO:0001217, UniParc:UPI000B4329B6, Un
EC:2.7.1.49, EC:2.7.4.7, GO:0005524, GO:0005829, GO:0008902, GO:0008972, GO:0009228, GO:0009229, RefSeq:WP_014113391.1, SO:0001217, UniParc:UPI00022BB08D, UniRef:UniRef100_G4NWH3, UniRef:UniRef50_
EC:1.3.1.-, EC:1.3.1.9, GO:0004318, GO:0006633, GO:0030497, GO:0070417, RefSeq:WP_014113392.1, SO:0001217, UniParc:UPI00022BB08E, UniRef:UniRef100_G4NWH4, UniRef:UniRef50_P54616, UniRef:UniRef90_P5
SO:0001217, UniRef:UniRef50_O31622
GO:0030435, SO:0001217, UniRef:UniRef50_Q08312, UniRef:UniRef90_Q08312
KEGG:K06343, SO:0001217, UniRef:UniRef50_Q08311, UniRef:UniRef90_A0A9P2XTN2
BlastRules:WP_003244668, GO:0030435, GO:0031160, KEGG:K06342, RefSeq:WP_014113396.1, SO:0001217, UniParc:UPI00022BB092, UniRef:UniRef100_G4NWH8, UniRef:UniRef50_Q08313, UniRef:UniRef90_Q08313
BlastRules:WP_003244871, GO:0030435, GO:0031160, KEGG:K06340, RefSeq:WP_014113398.1, SO:0001217, UniParc:UPI00022BB094, UniRef:UniRef100_G4NWI0, UniRef:UniRef50_Q08309, UniRef:UniRef90_Q08309
BlastRules:WP_003244844, GO:0005886, GO:0030435, RefSeq:WP_090557727.1, SO:0001217, UniParc:UPI00086BBFE3, UniRef:UniRef100_UPI00086BBFE3, UniRef:UniRef50_O31623, UniRef:UniRef90_O31623
RefSeq:WP_014113402.1, SO:0001217, UniParc:UPI00022BB098, UniRef:UniRef100_G4NWI4, UniRef:UniRef50_C0H3Y8, UniRef:UniRef90_G4NWI4
GO:0005737, GO:0030435, SO:0001217, UniRef:UniRef50_O31625, UniRef:UniRef90_O31625
PFAM:PF13361.12
EC:5.6.2.4, GO:0000725, GO:0003677, GO:0003689, GO:0005524, GO:0005829, GO:0009378, GO:0016853, GO:0016887, GO:0033202, GO:0036121, GO:0043138, GO:0061749, GO:0061775, GO:0140584, GO:0140665, G
SO:0001217, UniRef:UniRef50_C0H3Y9, UniRef:UniRef90_C0H3Y9
SO:0001217, UniRef:UniRef50_O31628, UniRef:UniRef90_D4FV61
EC:3.1.-.-, GO:0016788, SO:0001217, UniRef:UniRef50_O31629, UniRef:UniRef90_O31629
SO:0001217, UniRef:UniRef50_O31630, UniRef:UniRef90_O31630
PFAM:PF00756.26
RFAM:RF00162, SO:0000140
SO:0001217, UniRef:UniRef50_A0A2X1RG69
EC:4.4.1.13, GO:0005737, GO:0009086, GO:0016846, GO:0019346, GO:0030170, GO:0047804, RefSeq:WP_061188379.1, SO:0001217, UniParc:UPI0006A804E4, UniRef:UniRef100_UPI0006A804E4, UniRef:UniRef50_O05
SO:0001217, UniRef:UniRef50_A0A024Q6S0, UniRef:UniRef90_UPI0002F70A15
GO:0005886, RefSeq:WP_072183575.1, SO:0001217, UniParc:UPI0006A93C7A, UniRef:UniRef100_UPI0006A93C7A, UniRef:UniRef50_O31634, UniRef:UniRef90_O31634
SO:0000273
SO:0001217, UniParc:UPI0001976719, UniRef:UniRef100_R9RUX2, UniRef:UniRef50_R9RUX2, UniRef:UniRef90_R9RUX2
EC:3.1.-.-, KEGG:K06367, SO:0001217, UniRef:UniRef50_R9RWQ9, UniRef:UniRef90_R9RWQ9
GO:0003824, RefSeq:WP_014113413.1, SO:0001217, UniParc:UPI00022BB0A3, UniRef:UniRef100_G4NWJ6, UniRef:UniRef50_O31641, UniRef:UniRef90_O31641
GO:0003677, GO:0006355, GO:0008982, GO:0009401, GO:0016301, KEGG:K02538, SO:0001217, UniRef:UniRef50_O31644, UniRef:UniRef90_O31644
SO:0001217, UniRef:UniRef50_A0AAP3CMI6
BlastRules:WP_003245781, GO:0005351, GO:0005886, GO:0009401, GO:0016301, GO:0022877, GO:0090563, RefSeq:WP_327816554.1, SO:0001217, UniParc:UPI002E1CD052, UniRef:UniRef100_UPI002E1CD052, UniRe
EC:5.3.1.8, GO:0004476, GO:0005975, GO:0008270, RefSeq:WP_072183571.1, SO:0001217, UniParc:UPI0006A87C9C, UniRef:UniRef100_UPI0006A87C9C, UniRef:UniRef50_O31646, UniRef:UniRef90_A0A9Q4DTD7
RefSeq:WP_072182414.1, SO:0001217, UniParc:UPI0006A87EBF, UniRef:UniRef100_UPI0006A87EBF, UniRef:UniRef50_E0U0C9, UniRef:UniRef90_A0A2M8T668
SO:0001217, UniRef:UniRef50_E0U0D0, UniRef:UniRef90_UPI00227F119D
RefSeq:WP_072182415.1, SO:0001217, UniParc:UPI0006A82E9C, UniRef:UniRef100_A0A9Q4E484, UniRef:UniRef50_O31649, UniRef:UniRef90_A0A9Q4DR46
EC:3.1.1.-, GO:0002161, GO:0043039, KEGG:K03976, SO:0001217, UniRef:UniRef50_E3EJE5, UniRef:UniRef90_A0A6H0WFX2
RefSeq:WP_014113421.1, SO:0001217, UniParc:UPI00022BB0AB, UniRef:UniRef100_G4NWK4, UniRef:UniRef50_C0H3Z2, UniRef:UniRef90_C0H3Z2
RefSeq:WP_014113423.1, SO:0001217, UniParc:UPI00022BB0AD, UniRef:UniRef100_G4NWK6, UniRef:UniRef50_G4NWK6, UniRef:UniRef90_G4NWK6
EC:2.5.1.141, GO:0005886, GO:0006783, GO:0008495, GO:0016020, GO:0048034, SO:0001217, UniParc:UPI00022BB0AE, UniRef:UniRef100_G4NWK7, UniRef:UniRef50_O31652, UniRef:UniRef90_O31652
KEGG:K06339, RefSeq:WP_072182419.1, SO:0001217, UniParc:UPI0006A873C9, UniRef:UniRef100_UPI0006A873C9, UniRef:UniRef50_A0A3E2JWE8, UniRef:UniRef90_A0A6H0WL97
EC:3.5.1.-, GO:0005886, GO:0005975, GO:0016810, GO:0046872, SO:0001217, UniRef:UniRef50_O34798, UniRef:UniRef90_O34798
RefSeq:WP_061187248.1, SO:0001217, UniParc:UPI0007728A6E, UniRef:UniRef100_UPI0007728A6E, UniRef:UniRef50_O34554, UniRef:UniRef90_O34554
RefSeq:WP_014113427.1, SO:0001217, UniParc:UPI00022BB0B1, UniRef:UniRef100_G4NRU5, UniRef:UniRef50_O34438, UniRef:UniRef90_O34438
SO:0001217, UniRef:UniRef50_O34458, UniRef:UniRef90_E0U0E1
SO:0001217, UniRef:UniRef50_O35027, UniRef:UniRef90_G4NRU7
SO:0001217, UniRef:UniRef50_O34960, UniRef:UniRef90_A0A9Q4E5L3
COG:C, COG:COG3383, EC:1.-.-.-, EC:1.17.1.9, GO:0008863, GO:0009326, GO:0015942, GO:0016020, GO:0022904, GO:0043546, GO:0046872, GO:0051537, GO:0051539, SO:0001217, UniRef:UniRef50_Q49ZN0, UniRef:
COG:C, COG:COG2427, RefSeq:WP_072182424.1, SO:0001217, UniParc:UPI0006A8C945, UniRef:UniRef100_UPI0006A8C945, UniRef:UniRef50_O34681, UniRef:UniRef90_O34681
RefSeq:WP_014113433.1, SO:0001217, UniParc:UPI00022BB0B7, UniRef:UniRef100_G4NRV1, UniRef:UniRef50_A0AAP1E6A9, UniRef:UniRef90_A0AAP1E6A9
RefSeq:WP_072182425.1, SO:0001217, UniParc:UPI0006A82D47, UniRef:UniRef100_UPI0006A82D47, UniRef:UniRef50_C0SPC3, UniRef:UniRef90_G4NRV2
RefSeq:WP_061187251.1, SO:0001217, UniParc:UPI0006A852EE, UniRef:UniRef100_UPI0006A852EE, UniRef:UniRef50_O34679, UniRef:UniRef90_O34679
EC:2.4.1.384, KEGG:K25230, SO:0001217, UniRef:UniRef50_O34539, UniRef:UniRef90_A0A6H0WGZ7
RefSeq:WP_014113438.1, SO:0001217, UniParc:UPI00022BB239, UniRef:UniRef100_A0A2M8T4S9, UniRef:UniRef50_C0H437, UniRef:UniRef90_C0H3Z3
EC:4.2.1.75, GO:0004852, GO:0006780, RefSeq:WP_072182427.1, SO:0001217, UniParc:UPI0006A89ED9, UniRef:UniRef100_UPI0006A89ED9, UniRef:UniRef50_O34394, UniRef:UniRef90_O34394
GO:0005886, KEGG:K02069, RefSeq:WP_072182428.1, SO:0001217, UniParc:UPI0006A93033, UniRef:UniRef100_UPI0006A93033, UniRef:UniRef50_O34684, UniRef:UniRef90_O34684
GO:0005315, GO:0005524, GO:0016020, GO:0016887, GO:0035435, RefSeq:WP_014113441.1, SO:0001217, UniParc:UPI00022BB23C, UniRef:UniRef100_G4NRV9, UniRef:UniRef50_O34756, UniRef:UniRef90_O34756
GO:0005886, RefSeq:WP_072182430.1, SO:0001217, UniParc:UPI0006A90ED2, UniRef:UniRef100_UPI0006A90ED2, UniRef:UniRef50_O34428, UniRef:UniRef90_O34428
RefSeq:WP_014113443.1, SO:0001217, UniParc:UPI00022BB23E, UniRef:UniRef100_G4NRW1, UniRef:UniRef50_O34612, UniRef:UniRef90_A0A2M8T4U5
COG:C, COG:COG2427, RefSeq:WP_003239166.1, SO:0001217, UniParc:UPI00022BB23F, UniRef:UniRef100_A0A2M8T4T9, UniRef:UniRef50_O34633, UniRef:UniRef90_Q65IU2
SO:0001217, UniRef:UniRef50_UPI000887C9C8
SO:0001217, UniRef:UniRef50_UPI002E20172F, UniRef:UniRef90_UPI0005859302
EC:5.3.1.12, KEGG:K01812, SO:0001217, UniRef:UniRef50_A0A356AYX6, UniRef:UniRef90_A0A7U5AW26
PFAM:PF02614.20
SO:0001217, UniRef:UniRef50_A0A1V2YF82
SO:0001217, UniRef:UniRef50_O34961
SO:0001217, UniRef:UniRef50_UPI00166624E6
EC:1.-.-.-, GO:0008270, GO:0016491, RefSeq:WP_072182434.1, SO:0001217, UniParc:UPI0006A8C0C7, UniRef:UniRef100_UPI0006A8C0C7, UniRef:UniRef50_O35045, UniRef:UniRef90_O35045
EC:4.2.1.8, GO:0006064, GO:0008927, KEGG:K01686, SO:0001217, UniRef:UniRef50_UPI001BE83AFB, UniRef:UniRef90_UPI001BE83AFB
SO:0001217, UniRef:UniRef50_V9GBZ4
SO:0001217, UniRef:UniRef50_UPI0005BCA13F
SO:0001217, UniRef:UniRef50_Q70K13, UniRef:UniRef90_Q70K13
GO:0005886, GO:0022857, SO:0001217, UniRef:UniRef50_O34456, UniRef:UniRef90_O34456
GO:0000976, GO:0003700, GO:0006355, RefSeq:WP_072566130.1, SO:0001217, UniParc:UPI00090A8E0E, UniRef:UniRef100_A0A9Q4E5A4, UniRef:UniRef50_Q9JMQ1, UniRef:UniRef90_Q9JMQ1
EC:1.1.1.58, GO:0005829, GO:0008926, GO:0009026, GO:0019592, GO:0019698, RefSeq:WP_088679486.1, SO:0001217, UniParc:UPI000B537348, UniRef:UniRef100_UPI000B537348, UniRef:UniRef50_O34354, UniRef:Ur
EC:4.2.1.7, GO:0008789, GO:0019698, KEGG:K01685, SO:0001217, UniRef:UniRef50_O34673, UniRef:UniRef90_O34673
GO:0005886, RefSeq:WP_014113455.1, SO:0001217, UniParc:UPI00022BB24C, UniRef:UniRef100_G4NRX5, UniRef:UniRef50_O34578, UniRef:UniRef90_O34578
GO:0046872, RefSeq:WP_072182440.1, SO:0001217, UniParc:UPI0006A89E5F, UniRef:UniRef100_UPI0006A89E5F, UniRef:UniRef50_O34334, UniRef:UniRef90_O34334
BlastRules:WP_003245490, EC:3.-.-.-, GO:0005524, GO:0005778, GO:0005829, GO:0016558, GO:0016887, GO:0043335, RefSeq:WP_072182441.1, SO:0001217, UniParc:UPI0006A855D5, UniRef:UniRef100_UPI0006A855
BlastRules:WP_010886491, EC:3.1.3.-, GO:0004721, GO:0005737, GO:0030435, KEGG:K06359, SO:0001217, UniParc:UPI0001D28EA0, UniRef:UniRef100_A0A9Q4HA15, UniRef:UniRef50_Q00828, UniRef:UniRef90_Q0082
BlastRules:WP_003245487, BlastRules:WP_106073840, GO:0005576, GO:0005737, GO:0030435, KEGG:K06352, RefSeq:WP_014113458.1, SO:0001217, UniParc:UPI00022BB24F, UniRef:UniRef100_G4NRX9, UniRef:UniR
RefSeq:WP_087987210.1, SO:0001217, UniParc:UPI000B4541D0, UniRef:UniRef100_UPI000B4541D0, UniRef:UniRef50_O34446, UniRef:UniRef90_O34446
EC:3.5.1.28, GO:0005576, GO:0008745, GO:0009253, GO:0009254, GO:0030420, GO:0030435, GO:0071555, RefSeq:WP_088679485.1, SO:0001217, UniParc:UPI000B534C89, UniRef:UniRef100_UPI000B534C89, UniRef:U
RefSeq:WP_079288551.1, SO:0001217, UniParc:UPI0009A39B8C, UniRef:UniRef100_UPI0009A39B8C, UniRef:UniRef50_O34593, UniRef:UniRef90_O34593
RefSeq:WP_072566136.1, SO:0001217, UniParc:UPI0009097204, UniRef:UniRef100_UPI0009097204, UniRef:UniRef50_O34785, UniRef:UniRef90_O34785
EC:1.11.1.6, KEGG:K07217, RefSeq:WP_072182443.1, SO:0001217, UniParc:UPI0006A8594A, UniRef:UniRef100_UPI0006A8594A, UniRef:UniRef50_O34423, UniRef:UniRef90_O34423
RefSeq:WP_014113465.1, SO:0001217, UniParc:UPI00022BB256, UniRef:UniRef100_G4NRY6, UniRef:UniRef50_P39780, UniRef:UniRef90_P39780
GO:0003677, SO:0001217, UniRef:UniRef50_P23789, UniRef:UniRef90_P23789
RefSeq:WP_061187276.1, SO:0001217, UniParc:UPI0006A8112E, UniRef:UniRef100_UPI0006A8112E, UniRef:UniRef50_C0H3Z4, UniRef:UniRef90_C0H3Z4
RefSeq:WP_072182445.1, SO:0001217, UniParc:UPI0006A91EE9, UniRef:UniRef100_UPI0006A91EE9, UniRef:UniRef50_P39781, UniRef:UniRef90_G4NRY9
SO:0001217, UniRef:UniRef50_A0A0C2RYT8, UniRef:UniRef90_A0AAP1E6B6
RefSeq:WP_014113468.1, SO:0001217, UniParc:UPI00022BB259, UniRef:UniRef100_G4NRZ1, UniRef:UniRef50_G4NRZ1, UniRef:UniRef90_G4NRZ1
SO:0001217, UniParc:UPI0001976214, UniRef:UniRef100_E0U1R4, UniRef:UniRef50_A0A060PF20, UniRef:UniRef90_A0A060PF20
BlastRules:WP_003245290, RefSeq:WP_072182447.1, SO:0001217, UniParc:UPI0006A8954E, UniRef:UniRef100_A0A9Q4HLS2, UniRef:UniRef50_P39783, UniRef:UniRef90_P39783
BlastRules:WP_003244900, RefSeq:WP_064813284.1, SO:0001217, UniParc:UPI00061E3F2D, UniRef:UniRef100_A0A9Q4HGG8, UniRef:UniRef50_P54344, UniRef:UniRef90_P54344
GO:0003677, GO:0006352, GO:0016987, KEGG:K24072, RefSeq:WP_072182448.1, SO:0001217, UniParc:UPI0006A87B46, UniRef:UniRef100_UPI0006A87B46, UniRef:UniRef50_P39784, UniRef:UniRef90_P39784
BlastRules:WP_003244697, GO:0051276, SO:0001217, UniRef:UniRef50_P39785, UniRef:UniRef90_P39785
KEGG:K06909, RefSeq:WP_072182450.1, SO:0001217, UniParc:UPI0006A935CE, UniRef:UniRef100_UPI0006A935CE, UniRef:UniRef50_P39786, UniRef:UniRef90_P39786
SO:0001217, UniRef:UniRef50_P45917, UniRef:UniRef90_P54325
RefSeq:WP_014113475.1, SO:0001217, UniParc:UPI00022BB3CD, UniRef:UniRef100_G4NRZ8, UniRef:UniRef50_P54326, UniRef:UniRef90_P54326
GO:0005576, RefSeq:WP_072182451.1, SO:0001217, UniParc:UPI0006A844D2, UniRef:UniRef100_UPI0006A844D2, UniRef:UniRef50_P54327, UniRef:UniRef90_P54327
RefSeq:WP_072182452.1, SO:0001217, UniParc:UPI0006A878C9, UniRef:UniRef100_UPI0006A878C9, UniRef:UniRef50_C0H3Z6, UniRef:UniRef90_C0H3Z6
SO:0001217, UniRef:UniRef50_P54328, UniRef:UniRef90_P54328
RefSeq:WP_072182453.1, SO:0001217, UniParc:UPI0006A8D6B2, UniRef:UniRef100_A0A9Q4E2Y4, UniRef:UniRef50_P54329, UniRef:UniRef90_P54329
RefSeq:WP_072182454.1, SO:0001217, UniParc:UPI0006A80DDF, UniRef:UniRef100_UPI0006A80DDF, UniRef:UniRef50_P54330, UniRef:UniRef90_P54330
RefSeq:WP_003220591.1, SO:0001217, UniParc:UPI0001D28EBA, UniRef:UniRef100_G4NS05, UniRef:UniRef50_P45927, UniRef:UniRef90_P54331
RefSeq:WP_003220592.1, SO:0001217, UniParc:UPI0001D28EBB, UniRef:UniRef100_A0A9Q4DSF4, UniRef:UniRef50_P54332, UniRef:UniRef90_P54332
SO:0001217, UniRef:UniRef50_P54333, UniRef:UniRef90_A0A6I7FAP8
SO:0001217, UniRef:UniRef50_D4FVI4
SO:0001217, UniRef:UniRef50_A0A5S9MFK5, UniRef:UniRef90_UPI00202A35B3
SO:0001217, UniRef:UniRef50_UPI002DB9FC95
SO:0001217, UniRef:UniRef50_P54336, UniRef:UniRef90_P54336
RefSeq:WP_041905131.1, SO:0001217, UniParc:UPI000508B253, UniRef:UniRef100_UPI000508B253, UniRef:UniRef50_P45934, UniRef:UniRef90_P54338

RefSeq:WP_072182459.1, SO:0001217, UniParc:UPI0006A94051, UniRef:UniRef100_UPI0006A94051, UniRef:UniRef50_P54339, UniRef:UniRef90_P54339
RefSeq:WP_044445109.1, SO:0001217, UniParc:UPI0005CA6946, UniRef:UniRef100_A0A9Q4DQJ5, UniRef:UniRef50_P45936, UniRef:UniRef90_P54340
RefSeq:WP_061187291.1, SO:0001217, UniParc:UPI0006A811F9, UniRef:UniRef100_UPI0006A811F9, UniRef:UniRef90_C0H3Z9
SO:0001217, UniRef:UniRef50_P54341, UniRef:UniRef90_E0U1U1
SO:0001217, UniRef:UniRef50_P54342, UniRef:UniRef90_D4FVJ3
RefSeq:WP_072182462.1, SO:0001217, UniParc:UPI0006A8B716, UniRef:UniRef100_UPI0006A8B716, UniRef:UniRef50_P54343, UniRef:UniRef90_P54343
RefSeq:WP_072182463.1, SO:0001217, UniParc:UPI0006A8527E, UniRef:UniRef100_UPI0006A8527E, UniRef:UniRef50_P39797, UniRef:UniRef90_P39797
RefSeq:WP_014113495.1, SO:0001217, UniParc:UPI00022BB3E1, UniRef:UniRef100_G4NS21, UniRef:UniRef50_P39798, UniRef:UniRef90_A0AAQ3IIZ5
GO:0016020, GO:0140911, RefSeq:WP_061187296.1, SO:0001217, UniParc:UPI0006A7F9A2, UniRef:UniRef100_UPI0006A7F9A2, UniRef:UniRef50_Q99163, UniRef:UniRef90_Q99163
EC:3.5.1.28, GO:0005576, GO:0008745, GO:0009253, GO:0009254, GO:0030420, GO:0030435, GO:0071555, SO:0001217, UniRef:UniRef50_P39800, UniRef:UniRef90_P39800
BlastRules:WP_003232646, GO:0030435, GO:0097351, KEGG:K06389, RefSeq:WP_003239095.1, SO:0001217, UniParc:UPI00022BB3E5, UniRef:UniRef100_A0A9Q4HGF9, UniRef:UniRef50_O34800, UniRef:UniRef90_O34
BlastRules:WP_003244695, GO:0005886, GO:0030435, KEGG:K06388, RefSeq:WP_061187299.1, SO:0001217, UniParc:UPI0006A820FB, UniRef:UniRef100_A0A9Q4HHF8, UniRef:UniRef50_O34853, UniRef:UniRef90_O34
GO:0005315, GO:0005886, GO:0015293, GO:0035435, KEGG:K03306, RefSeq:WP_088679479.1, SO:0001217, UniParc:UPI00086F616A, UniRef:UniRef100_A0A9Q4E3T1, UniRef:UniRef50_O34436, UniRef:UniRef90_O344
COG:COG1392, COG:P, RefSeq:WP_014113501.1, SO:0001217, UniParc:UPI00022BB3E8, UniRef:UniRef100_G4NV10, UniRef:UniRef50_O34454, UniRef:UniRef90_O34454
BlastRules:WP_003244819, GO:0003333, GO:0005886, GO:0015179, RefSeq:WP_072182467.1, SO:0001217, UniParc:UPI0006A8A95E, UniRef:UniRef100_UPI0006A8A95E, UniRef:UniRef50_O34739, UniRef:UniRef90_O34
EC:1.13.11.-, GO:0005737, GO:0009056, GO:0009636, GO:0046872, GO:0051213, KEGG:K15975, RefSeq:WP_072182468.1, SO:0001217, UniParc:UPI0006A8D1C8, UniRef:UniRef100_UPI0006A8D1C8, UniRef:UniRef50_(
RefSeq:WP_120698821.1, SO:0001217, UniParc:UPI000A9A04D5, UniRef:UniRef100_A0A9Q4E310, UniRef:UniRef50_A0A2K4Z9I1, UniRef:UniRef90_A0A9Q4E310
SO:0001217, UniRef:UniRef50_A0A356PB71
SO:0001217, UniRef:UniRef50_O34575, UniRef:UniRef90_G4NV14
EC:2.4.-.-, GO:0005886, GO:0016757, RefSeq:WP_014113506.1, SO:0001217, UniParc:UPI00022BB3ED, UniRef:UniRef100_G4NV15, UniRef:UniRef50_O34319, UniRef:UniRef90_O34319
BlastRules:WP_002853235, BlastRules:WP_003912936, BlastRules:WP_009872209, BlastRules:WP_009967069, EC:3.4.21.107, GO:0004252, GO:0005886, GO:0006508, SO:0001217, UniRef:UniRef50_Q9R9I1, UniRef:UniF
EC:1.5.1.2, SO:0001217, UniRef:UniRef50_Q00777, UniRef:UniRef90_E0U1V8
BlastRules:WP_003245814, EC:3.4.11.-, GO:0004177, GO:0006508, GO:0008237, GO:0030435, GO:0046872, KEGG:K16203, RefSeq:WP_014113510.1, SO:0001217, UniParc:UPI00022BB3F1, UniRef:UniRef100_G4NV19, (
BlastRules:WP_003245446, GO:0005886, GO:0015031, GO:0015833, GO:0030435, GO:0055085, KEGG:K16200, SO:0001217, UniRef:UniRef50_P24138, UniRef:UniRef90_P26903
BlastRules:WP_003245702, GO:0005886, GO:0015031, GO:0015833, GO:0030435, GO:0055085, SO:0001217, UniRef:UniRef50_P26904, UniRef:UniRef90_P26904
BlastRules:WP_003232615, EC:7.4.2.9, GO:0005524, GO:0005886, GO:0015031, GO:0015833, GO:0016887, GO:0022857, GO:0030435, GO:0055085, RefSeq:WP_061187309.1, SO:0001217, UniParc:UPI0006A8F440, UniF
PFAM:PF00496.27
KEGG:K15580, SO:0001217, UniRef:UniRef50_W4RKL7, UniRef:UniRef90_UPI0022803194
EC:3.4.16.-, GO:0004180, GO:0005737, GO:0006508, GO:0008236, GO:0016998, GO:0071555, KEGG:K01297, SO:0001217, UniRef:UniRef50_O34851, UniRef:UniRef90_O34851
BlastRules:WP_003244980, EC:5.1.1.20, GO:0016998, GO:0046872, GO:0071555, GO:0103031, KEGG:K19802, SO:0001217, UniRef:UniRef50_O34508, UniRef:UniRef90_O34508
SO:0001217, UniRef:UniRef50_A0A1B9B288, UniRef:UniRef90_UPI000A109F70
PFAM:PF00005.33
EC:7.4.2.-, GO:0005524, GO:0015833, GO:0016887, GO:0055085, SO:0001217, UniRef:UniRef50_C0SP98, UniRef:UniRef90_C0SP98
BlastRules:WP_003244689, EC:3.1.1.31, GO:0005829, GO:0006006, GO:0006098, GO:0017057, SO:0001217, UniRef:UniRef50_O34499, UniRef:UniRef90_O34499
EC:3.5.3.-, GO:0016990, GO:0019546, KEGG:K01482, RefSeq:WP_014113520.1, SO:0001217, UniParc:UPI00022BB55F, UniRef:UniRef100_G4NV29, UniRef:UniRef50_O34497, UniRef:UniRef90_O34497
EC:3.1.2.-, GO:0005737, GO:0005829, GO:0006637, GO:0009062, GO:0052816, SO:0001217, UniParc:UPI00022BB560, UniRef:UniRef100_G4NV30, UniRef:UniRef50_P49851, UniRef:UniRef90_P49851
EC:1.14.12.17, GO:0005344, GO:0005737, GO:0008941, GO:0009636, GO:0019825, GO:0020037, GO:0046210, GO:0046872, GO:0051409, GO:0071500, GO:0071949, KEGG:K05916, RefSeq:WP_014113522.1, SO:000121
RefSeq:WP_014113523.1, SO:0001217, UniParc:UPI00022BB562, UniRef:UniRef100_G4NV32, UniRef:UniRef50_O31653, UniRef:UniRef90_E0U2G5
GO:0005886, RefSeq:WP_014113524.1, SO:0001217, UniParc:UPI00022BB563, UniRef:UniRef100_G4NV33, UniRef:UniRef50_P49853, UniRef:UniRef90_P49853
RefSeq:WP_014113525.1, SO:0001217, UniParc:UPI00022BB564, UniRef:UniRef100_G4NV34, UniRef:UniRef50_P49854, UniRef:UniRef90_P49854
RefSeq:WP_014113526.1, SO:0001217, UniParc:UPI00022BB565, UniRef:UniRef100_G4NV35, UniRef:UniRef50_P49855, UniRef:UniRef90_A0A9W5LHK2
RFAM:RF00442, SO:0000035
BlastRules:WP_003232583, GO:0005886, GO:0006811, GO:0022857, GO:0055085, RefSeq:WP_014113527.1, SO:0001217, UniParc:UPI00022BB566, UniRef:UniRef100_G4NV36, UniRef:UniRef50_P49856, UniRef:UniRef9
BlastRules:WP_003245695, GO:0005886, GO:0006811, GO:0022857, GO:0055085, KEGG:K18925, RefSeq:WP_003218500.1, SO:0001217, UniParc:UPI0001D283F7, UniRef:UniRef100_G4NV37, UniRef:UniRef50_P49857, U
EC:3.5.1.10, GO:0006189, GO:0006730, GO:0008864, RefSeq:WP_014113528.1, SO:0001217, UniParc:UPI00022BB567, UniRef:UniRef100_G4NV38, UniRef:UniRef50_O34990, UniRef:UniRef90_O34990
EC:2.7.2.11, GO:0003723, GO:0004349, GO:0005524, GO:0005829, GO:0006561, GO:0055129, SO:0001217, UniRef:UniRef50_Q81P26, UniRef:UniRef90_P39820
SO:0001217, UniRef:UniRef50_UPI001C8A875C
SO:0001217, UniRef:UniRef50_A0A829H3E3
EC:1.11.1.28, GO:0006979, KEGG:K04063, RefSeq:WP_072182938.1, SO:0001217, UniParc:UPI0006A826C9, UniRef:UniRef100_A0A9Q4E7V5, UniRef:UniRef50_O34762, UniRef:UniRef90_O34762
BlastRules:WP_003245653, GO:0000122, GO:0000977, GO:0001227, GO:0005737, GO:0006355, GO:0006950, GO:0042542, GO:0042802, GO:0042803, GO:0045892, RefSeq:WP_003218505.1, SO:0001217, UniParc:UPI0
EC:1.11.1.28, GO:0006979, KEGG:K04063, SO:0001217, UniRef:UniRef50_P80242, UniRef:UniRef90_P80242
SO:0001217, UniRef:UniRef50_A0A1D9PIG7, UniRef:UniRef90_G4NV44
SO:0001217, UniRef:UniRef50_O34598, UniRef:UniRef90_UPI00031D2AE9
SO:0001217, UniRef:UniRef50_G4NV46, UniRef:UniRef90_G4NV46
EC:2.1.1.14, GO:0003871, GO:0008270, GO:0009086, GO:0032259, KEGG:K00549, SO:0001217, UniRef:UniRef50_P80877, UniRef:UniRef90_P80877
RFAM:RF00162, SO:0000140
BlastRules:WP_003232562, EC:3.4.21.-, GO:0004252, GO:0005737, GO:0006508, RefSeq:WP_014113539.1, SO:0001217, UniParc:UPI00022BB572, UniRef:UniRef100_G4NV49, UniRef:UniRef50_P11018, UniRef:UniRef90_
BlastRules:WP_003232560, RefSeq:WP_014113541.1, SO:0001217, UniParc:UPI00022BB574, UniRef:UniRef100_G4NV51, UniRef:UniRef50_O34860, UniRef:UniRef90_O34860
BlastRules:WP_003245037, KEGG:K16785, RefSeq:WP_061187327.1, SO:0001217, UniParc:UPI0006A84BED, UniRef:UniRef100_A0A9Q4HHD9, UniRef:UniRef50_O34572, UniRef:UniRef90_A0A9Q4HHD9
EC:7.6.2.-, GO:0005524, GO:0006810, GO:0009898, GO:0016887, GO:0042626, GO:0043190, SO:0001217, UniRef:UniRef50_O34362, UniRef:UniRef90_O34362
PFAM:PF00005.33
GO:0005886, KEGG:K16925, RefSeq:WP_014113544.1, SO:0001217, UniParc:UPI00022BB577, UniRef:UniRef100_G4NV54, UniRef:UniRef50_O34738, UniRef:UniRef90_O34738
BlastRules:WP_003244962, GO:0030975, KEGG:K24620, RefSeq:WP_072566171.1, SO:0001217, UniParc:UPI000868DE8C, UniRef:UniRef100_UPI000868DE8C, UniRef:UniRef50_O34911, UniRef:UniRef90_O34911
RFAM:RF00059, SO:0000035
BlastRules:WP_003232551, GO:0000156, GO:0000976, GO:0005829, GO:0006355, GO:0032993, KEGG:K18941, SO:0001217, UniRef:UniRef50_O34903, UniRef:UniRef90_O34903
EC:2.7.13.3, GO:0000155, GO:0000160, GO:0005524, GO:0005886, SO:0001217, UniRef:UniRef50_O34638, UniRef:UniRef90_O34638
SO:0001217, UniRef:UniRef50_O34638, UniRef:UniRef90_A0A7G7U9T6
SO:0001217, UniRef:UniRef50_O34551, UniRef:UniRef90_E0U2I9
SO:0001217, UniRef:UniRef50_O35012, UniRef:UniRef90_A0A5D4N3J4
RefSeq:WP_003218541.1, SO:0001217, UniParc:UPI00000602C0, UniRef:UniRef100_O34405, UniRef:UniRef50_O34405, UniRef:UniRef90_O34405
RFAM:RF00380, SO:0000140
SO:0001217, UniRef:UniRef50_A0A9W5PCJ4, UniRef:UniRef90_A0A9W5PCJ4
GO:0005886, GO:0015095, GO:0015693, GO:0016020, GO:0046872, SO:0001217, UniRef:UniRef50_O34442, UniRef:UniRef90_O34442
BlastRules:WP_003232541, COG:COG0789, COG:K, GO:0001046, GO:0003700, GO:0005886, GO:0043562, GO:0090294, RefSeq:WP_014113551.1, SO:0001217, UniParc:UPI00022BB57E, UniRef:UniRef100_G4NV62, Uni
RefSeq:WP_003218545.1, SO:0001217, UniParc:UPI0001D2840F, UniRef:UniRef100_A0A9Q4HHJ7, UniRef:UniRef50_Q65KM2, UniRef:UniRef90_A0A9P1JG91
BlastRules:WP_003245766, RefSeq:WP_014113552.1, SO:0001217, UniParc:UPI00022BB57F, UniRef:UniRef100_G4NV64, UniRef:UniRef50_O34763, UniRef:UniRef90_O34763
GO:0003677, GO:0003700, GO:0006355, GO:0006950, SO:0001217, UniRef:UniRef50_O34949, UniRef:UniRef90_O34949
RefSeq:WP_090557627.1, SO:0001217, UniParc:UPI00086C31CA, UniRef:UniRef100_UPI00086C31CA, UniRef:UniRef50_C0SPB9, UniRef:UniRef90_A0A9W5LHG4
SO:0001217, UniRef:UniRef50_O34495
EC:3.1.-.-, GO:0008758, GO:0009245, GO:0016020, GO:0046872, KEGG:K07098, SO:0001217, UniRef:UniRef50_O35040, UniRef:UniRef90_O35040
SO:0001217, UniRef:UniRef50_A0A160F530
GO:0005886, SO:0001217, UniRef:UniRef50_A0A150MDB8, UniRef:UniRef50_O34830, UniRef:UniRef90_O34830
EC:2.4.-.-, GO:0005886, GO:0016757, RefSeq:WP_072566174.1, SO:0001217, UniParc:UPI000909A19A, UniRef:UniRef100_UPI000909A19A, UniRef:UniRef50_O34755, UniRef:UniRef90_O34755
EC:6.5.1.1, GO:0003910, GO:0005524, GO:0006281, KEGG:K01971, SO:0001217, UniRef:UniRef50_O34398, UniRef:UniRef90_D4FVR3
SO:0001217, UniRef:UniRef50_A0A5S9M9T1
EC:2.7.7.65, GO:0005886, KEGG:K21023, SO:0001217, UniRef:UniRef50_O34311, UniRef:UniRef90_O34311
KEGG:K03975, SO:0001217, UniRef:UniRef50_O34908, UniRef:UniRef90_D4FVR7
GO:0005886, RefSeq:WP_014113563.1, SO:0001217, UniParc:UPI00022BB6F9, UniRef:UniRef100_G4NV75, UniRef:UniRef50_O34997, UniRef:UniRef90_O34997
GO:0003677, GO:0005737, GO:0006352, GO:0006355, GO:0016987, KEGG:K03093, RefSeq:WP_014113564.1, SO:0001217, UniParc:UPI00022BB6FA, UniRef:UniRef100_G4NV76, UniRef:UniRef50_O31654, UniRef:UniRef
SO:0001217, UniRef:UniRef50_O31655
GO:0003690, GO:0006265, GO:0030435, RefSeq:WP_003218568.1, SO:0001217, UniParc:UPI000004AB08, UniRef:UniRef100_P04833, UniRef:UniRef50_P04833, UniRef:UniRef90_P04833
BlastRules:WP_003245220, RefSeq:WP_014113566.1, SO:0001217, UniParc:UPI00022BB6FC, UniRef:UniRef100_G4NV79, UniRef:UniRef50_O31656, UniRef:UniRef90_O31656
EC:3.4.24.-, GO:0004222, GO:0005886, GO:0006508, GO:0008270, SO:0001217, UniRef:UniRef50_O31657, UniRef:UniRef90_O31657
GO:0005886, GO:0006813, GO:0008324, RefSeq:WP_014113568.1, SO:0001217, UniParc:UPI00022BB6FE, UniRef:UniRef100_G4NV81, UniRef:UniRef50_O31658, UniRef:UniRef90_O31658
RefSeq:WP_003232510.1, SO:0001217, UniParc:UPI000195C5D3, UniRef:UniRef100_C0H402, UniRef:UniRef50_C0H402, UniRef:UniRef90_C0H402
RefSeq:WP_014113569.1, SO:0001217, UniParc:UPI00022BB6FF, UniRef:UniRef100_G4NV83, UniRef:UniRef50_O31659, UniRef:UniRef90_A0A6H0WQD0
EC:2.3.-.-, GO:0005886, GO:0016747, RefSeq:WP_061187342.1, SO:0001217, UniParc:UPI0006A8047E, UniRef:UniRef100_UPI0006A8047E, UniRef:UniRef50_O31660, UniRef:UniRef90_O31660
EC:2.7.13.3, GO:0000155, GO:0005524, GO:0030435, SO:0001217, UniRef:UniRef50_O31661, UniRef:UniRef90_O31661
SO:0001217, UniRef:UniRef50_UPI00374809B8
EC:2.1.1.63, GO:0003908, GO:0005737, GO:0006307, GO:0032259, RefSeq:WP_072183220.1, SO:0001217, UniParc:UPI0006A839A1, UniRef:UniRef100_UPI0006A839A1, UniRef:UniRef50_P11742, UniRef:UniRef90_P1174
RefSeq:WP_003218585.1, SO:0001217, UniParc:UPI0001D284E6, UniRef:UniRef100_A0A9Q4E2W3, UniRef:UniRef50_A0A2K4Z9I7, UniRef:UniRef90_A0A2K4Z9I7
SO:0001217, UniRef:UniRef50_A0A9W5VD99
EC:2.7.1.100, GO:0005524, GO:0009086, GO:0019509, GO:0046522, KEGG:K00899, RefSeq:WP_133063226.1, SO:0001217, UniParc:UPI0006A7F951, UniRef:UniRef100_UPI0006A7F951, UniRef:UniRef50_O31663, UniRef
RFAM:RF00162, SO:0000140
EC:3.5.1.111, GO:0106008, RefSeq:WP_087987168.1, SO:0001217, UniParc:UPI0006A8CDE6, UniRef:UniRef100_UPI0006A8CDE6, UniRef:UniRef50_O31664, UniRef:UniRef90_O31664
BlastRules:WP_003244774, EC:2.6.1.117, GO:0008483, GO:0019509, GO:0030170, KEGG:K08969, SO:0001217, UniRef:UniRef50_P39643, UniRef:UniRef90_O31665
RFAM:RF00162, SO:0000140
EC:5.3.2.5, GO:0000287, GO:0016984, GO:0019509, GO:0043715, KEGG:K08965, SO:0001217, UniRef:UniRef50_Q5L1E2, UniRef:UniRef90_O31666
EC:3.1.3.87, GO:0005737, GO:0008253, GO:0016791, GO:0019509, GO:0043716, KEGG:K08966, RefSeq:WP_014113578.1, SO:0001217, UniParc:UPI00022BB708, UniRef:UniRef100_G4NVH3, UniRef:UniRef50_O31667, U
EC:4.2.1.109, GO:0005737, GO:0008270, GO:0019509, GO:0046570, RefSeq:WP_061187349.1, SO:0001217, UniParc:UPI0006A7FF18, UniRef:UniRef100_UPI0006A7FF18, UniRef:UniRef50_O31668, UniRef:UniRef90_O316
BlastRules:WP_003244952, EC:1.13.11.53, GO:0005506, GO:0006555, GO:0010308, GO:0010309, GO:0016151, GO:0019284, GO:0019509, RefSeq:WP_061187350.1, SO:0001217, UniParc:UPI00061FDAB9, UniRef:UniRef
GO:0005886, RefSeq:WP_061187351.1, SO:0001217, UniParc:UPI0006A8CE11, UniRef:UniRef100_UPI0006A8CE11, UniRef:UniRef50_O31670, UniRef:UniRef90_O31670
BlastRules:WP_003218598, EC:3.1.3.-, GO:0016787, GO:0030435, GO:0043937, GO:0046983, KEGG:K06376, RefSeq:WP_003218598.1, SO:0001217, UniParc:UPI00000602E2, UniRef:UniRef100_P05043, UniRef:UniRef50_
SO:0001217, UniRef:UniRef50_P06630, UniRef:UniRef90_UPI002280E4DB
BlastRules:WP_010886497, EC:2.7.13.3, GO:0000155, GO:0005524, GO:0005886, GO:0030435, SO:0001217, UniRef:UniRef50_O31671, UniRef:UniRef90_O31671
BlastRules:WP_003232475, GO:0003677, GO:0003700, GO:0006355, GO:0006950, RefSeq:WP_003232475.1, SO:0001217, UniParc:UPI00000602E5, UniRef:UniRef100_O31672, UniRef:UniRef50_O31672, UniRef:UniRef90_
GO:0005886, GO:0006935, GO:0097588, SO:0001217, UniRef:UniRef50_P28612, UniRef:UniRef90_P28612
KEGG:K02556, SO:0001217, UniRef:UniRef50_P28611, UniRef:UniRef90_UPI001EFBC74E
BlastRules:WP_009967104, SO:0001217, UniRef:UniRef50_A0A1D7QY86, UniRef:UniRef90_G4NVI4
GO:0005886, RefSeq:WP_061187355.1, SO:0001217, UniParc:UPI000772B7E3, UniRef:UniRef100_A0A9Q4E3X0, UniRef:UniRef50_O31674, UniRef:UniRef90_O31674
RFAM:RF00522, SO:0000035
EC:6.3.4.20, GO:0005524, GO:0008270, GO:0008616, GO:0016879, KEGG:K06920, RefSeq:WP_014113588.1, SO:0001217, UniParc:UPI00022BB870, UniRef:UniRef100_G4NVI6, UniRef:UniRef50_O31675, UniRef:UniRef90
EC:4.1.2.50, GO:0003824, GO:0008616, GO:0046872, GO:0070497, KEGG:K01737, SO:0001217, UniParc:UPI00022BB871, UniRef:UniRef100_G4NVI7, UniRef:UniRef50_O31676, UniRef:UniRef90_O31676
EC:4.3.99.3, GO:0000287, GO:0006400, GO:0008616, GO:0016840, GO:0042803, GO:0051539, GO:1904047, KEGG:K10026, SO:0001217, UniRef:UniRef50_O31677, UniRef:UniRef90_O31677
EC:1.7.1.13, GO:0005737, GO:0006400, GO:0008616, GO:0033739, GO:0046857, RefSeq:WP_072182860.1, SO:0001217, UniParc:UPI0006A89EB4, UniRef:UniRef100_UPI0006A89EB4, UniRef:UniRef50_O31678, UniRef:U
BlastRules:WP_003245384, KEGG:K06320, SO:0001217, UniRef:UniRef50_O31681, UniRef:UniRef90_UPI00201DEC3C
GO:0008932, RefSeq:WP_268393024.1, SO:0001217, UniParc:UPI002280B8D0, UniRef:UniRef100_A0A9Q4HLM1, UniRef:UniRef50_C0H403, UniRef:UniRef90_C0H403
SO:0001217, UniRef:UniRef50_G4NVJ2, UniRef:UniRef90_G4NVJ2
RefSeq:WP_087987157.1, SO:0001217, UniParc:UPI000B45097F, UniRef:UniRef100_UPI000B45097F, UniRef:UniRef50_O31683, UniRef:UniRef90_A0A2M8T4B7
COG:COG4873, COG:S, RefSeq:WP_003232446.1, SO:0001217, UniParc:UPI000195C5E0, UniRef:UniRef100_O31684, UniRef:UniRef50_O31684, UniRef:UniRef90_O31684
RefSeq:WP_003218622.1, SO:0001217, UniParc:UPI000195C5E1, UniRef:UniRef100_C0H405, UniRef:UniRef50_C0H405, UniRef:UniRef90_C0H405
SO:0001217, UniRef:UniRef50_O31685, UniRef:UniRef90_UPI002280480F
BlastRules:WP_003245552, GO:0016020, GO:0030435, RefSeq:WP_268442752.1, SO:0001217, UniParc:UPI00227EF525, UniRef:UniRef100_A0A9Q4E4R6, UniRef:UniRef50_O31686, UniRef:UniRef90_O31686
GO:0016209, GO:0016491, GO:0030435, GO:0043594, RefSeq:WP_072566193.1, SO:0001217, UniParc:UPI00090C4EAD, UniRef:UniRef100_UPI00090C4EAD, UniRef:UniRef50_O31687, UniRef:UniRef90_O31687

SO:0001217, UniRef:UniRef50_UPI0028B03468
RefSeq:WP_014113599.1, SO:0001217, UniParc:UPI00022BB87B, UniRef:UniRef100_G4NVK0, UniRef:UniRef50_A0A9Q6A7Z3, UniRef:UniRef90_G4NVK0
BlastRules:WP_003244662, EC:3.4.13.-, GO:0004177, GO:0006508, GO:0008235, GO:0016805, GO:0046872, RefSeq:WP_088679429.1, SO:0001217, UniParc:UPI000B5331C4, UniRef:UniRef100_UPI000B5331C4, UniRef:U
RefSeq:WP_003218633.1, SO:0001217, UniParc:UPI0001976230, UniRef:UniRef100_A0A080UE22, UniRef:UniRef50_A0A080UE22, UniRef:UniRef90_A0A080UE22
GO:0000976, GO:0003700, GO:0006355, RefSeq:WP_014113601.1, SO:0001217, UniParc:UPI00022BB87D, UniRef:UniRef100_G4NVK3, UniRef:UniRef50_O31690, UniRef:UniRef90_O31690
SO:0001217, UniRef:UniRef50_UPI002E22B5A1, UniRef:UniRef90_UPI00025D3110
BlastRules:WP_000473197, BlastRules:WP_003244661, EC:2.7.1.199, EC:2.7.1.69, SO:0001217, UniRef:UniRef50_P20166, UniRef:UniRef90_A0A9Q4HZS1
COG:COG1925, COG:TG, GO:0005737, GO:0009401, GO:0043610, RefSeq:WP_010334034.1, SO:0001217, UniParc:UPI00028884BD, UniRef:UniRef100_A0A9Q6A806, UniRef:UniRef50_P08877, UniRef:UniRef90_P08877
SO:0001217, UniRef:UniRef50_UPI0034D5A87D
SO:0001217, UniRef:UniRef50_A0A0D0SEZ9
SO:0001217, UniRef:UniRef50_H1LIZ2, UniRef:UniRef90_UPI0022A12919
BlastRules:WP_003218644, KEGG:K06315, RefSeq:WP_072182867.1, SO:0001217, UniParc:UPI0006A8F057, UniRef:UniRef100_UPI0006A8F057, UniRef:UniRef50_P37955, UniRef:UniRef90_P37955
EC:4.1.99.14, GO:0003677, GO:0003913, GO:0006281, GO:0006290, GO:0016830, GO:0030435, GO:0042601, GO:0046500, GO:0046872, GO:0051536, GO:0051539, GO:1904047, KEGG:K03716, RefSeq:WP_014113605.1
RefSeq:WP_072182868.1, SO:0001217, UniParc:UPI0006A8C773, UniRef:UniRef100_A0A9Q4E3K1, UniRef:UniRef50_Q796K9, UniRef:UniRef90_G4NVL0
GO:0005886, GO:0007165, GO:0045121, GO:0050918, SO:0001217, UniRef:UniRef50_P54576, UniRef:UniRef90_P54576
EC:1.1.-.-, GO:0005886, GO:0016054, GO:0016616, GO:0045121, GO:0050661, GO:0051287, RefSeq:WP_041905438.1, SO:0001217, UniParc:UPI0004FFF431, UniRef:UniRef100_A0A9Q4E7P6, UniRef:UniRef50_O34948, U
RefSeq:WP_072182870.1, SO:0001217, UniParc:UPI0006A869B6, UniRef:UniRef100_UPI0006A869B6, UniRef:UniRef50_O31398, UniRef:UniRef90_A0A9Q4E4Q7
EC:3.4.16.4, GO:0005886, GO:0006508, GO:0008360, GO:0008658, GO:0009002, GO:0009252, GO:0046677, GO:0071555, GO:0071972, KEGG:K21466, RefSeq:WP_133063225.1, SO:0001217, UniParc:UPI0006A8BD7A, U
BlastRules:WP_000087674, BlastRules:WP_003245779, EC:2.7.13.3, GO:0000155, GO:0005524, GO:0006355, GO:0030435, GO:0042802, SO:0001217, UniRef:UniRef50_P16497, UniRef:UniRef90_P16497
EC:2.6.1.-, GO:0003824, GO:0005737, GO:0006520, GO:0008483, GO:0009058, GO:0009089, GO:0019877, GO:0030170, KEGG:K00841, RefSeq:WP_003218653.1, SO:0001217, UniParc:UPI0001D285D2, UniRef:UniRef10
SO:0001217, UniRef:UniRef50_C0H406, UniRef:UniRef90_C0H406
BlastRules:WP_009967126, GO:0000160, GO:0006935, SO:0001217, UniRef:UniRef50_P37599, UniRef:UniRef90_P37599
RefSeq:WP_003232406.1, SO:0001217, UniParc:UPI0000060308, UniRef:UniRef100_P42430, UniRef:UniRef50_P42430, UniRef:UniRef90_P42430
SO:0001217, UniRef:UniRef50_UPI002282A3FA, UniRef:UniRef90_UPI002282A3FA
EC:2.-.-.-, GO:0008360, GO:0016757, GO:0018104, GO:0030435, GO:0031160, GO:0071555, GO:0071972, SO:0001217, UniRef:UniRef50_O34816, UniRef:UniRef90_O34816
GO:0008758, GO:0009245, GO:0016020, GO:0046872, KEGG:K07098, RefSeq:WP_014113616.1, SO:0001217, UniParc:UPI00022BB88C, UniRef:UniRef100_G4NVM3, UniRef:UniRef50_O34870, UniRef:UniRef90_O34870
SO:0001217, UniRef:UniRef50_A0A2W4MUI8
GO:0071111, SO:0001217, UniRef:UniRef50_O35014, UniRef:UniRef90_O35014
SO:0001217, UniRef:UniRef50_UPI0027EC1DE3
RFAM:RF02273, SO:0000655
RefSeq:WP_003218671.1, SO:0001217, UniParc:UPI0000060310, UniRef:UniRef100_O34588, UniRef:UniRef50_O34588, UniRef:UniRef90_O34588
KEGG:K09776, RefSeq:WP_003218672.1, SO:0001217, UniParc:UPI0001D286A6, UniRef:UniRef100_A0A9W5LHD3, UniRef:UniRef50_O34776, UniRef:UniRef90_O34776
BlastRules:WP_010886502, RefSeq:WP_003218673.1, SO:0001217, UniParc:UPI000197623B, UniRef:UniRef100_O31697, UniRef:UniRef50_O31697, UniRef:UniRef90_O31697
GO:0180001, RefSeq:WP_003218674.1, SO:0001217, UniParc:UPI0000060313, UniRef:UniRef100_O31698, UniRef:UniRef50_O31698, UniRef:UniRef90_O31698
BlastRules:WP_003232387, GO:0000976, GO:0003700, GO:0006355, RefSeq:WP_014113620.1, SO:0001217, UniParc:UPI00022BBA2B, UniRef:UniRef100_G4NVN1, UniRef:UniRef50_O34827, UniRef:UniRef90_O34827
GO:0010181, GO:0016651, RefSeq:WP_072183211.1, SO:0001217, UniParc:UPI0006A7F731, UniRef:UniRef100_UPI0006A7F731, UniRef:UniRef50_O34737, UniRef:UniRef90_O34737
RefSeq:WP_061187373.1, SO:0001217, UniParc:UPI0006A8BF48, UniRef:UniRef100_UPI0006A8BF48, UniRef:UniRef50_O34879, UniRef:UniRef90_O34879
GO:0009055, GO:0010181, GO:0016651, RefSeq:WP_072183212.1, SO:0001217, UniParc:UPI0006A80B62, UniRef:UniRef100_UPI0006A80B62, UniRef:UniRef50_O34589, UniRef:UniRef90_O34589
EC:2.3.1.89, GO:0009089, GO:0019877, GO:0047200, KEGG:K05822, RefSeq:WP_072183213.1, SO:0001217, UniParc:UPI0006A80EC7, UniRef:UniRef100_UPI0006A80EC7, UniRef:UniRef50_O34981, UniRef:UniRef90_O34
SO:0001217, UniRef:UniRef50_W4VF30
RefSeq:WP_003218684.1, SO:0001217, UniParc:UPI0001D286AC, UniRef:UniRef100_G4NVN7, UniRef:UniRef50_O34783, UniRef:UniRef90_O34783
BlastRules:WP_003244994, GO:0005886, GO:0008381, RefSeq:WP_014113625.1, SO:0001217, UniParc:UPI00022BBA30, UniRef:UniRef100_G4NVN8, UniRef:UniRef50_O34897, UniRef:UniRef90_O34897
BlastRules:WP_003245371, COG:COG0450, COG:V, EC:1.11.1.-, GO:0005829, GO:0006979, GO:0008379, GO:0042744, GO:0045454, RefSeq:WP_003245371.1, SO:0001217, UniParc:UPI000006031C, UniRef:UniRef100_C
EC:1.8.-.-, GO:0005737, GO:0016209, GO:0016491, SO:0001217, UniRef:UniRef50_O31699, UniRef:UniRef90_O31699
BlastRules:WP_003232378, GO:0003677, GO:0005737, GO:0009295, KEGG:K26248, RefSeq:WP_014113628.1, SO:0001217, UniParc:UPI0001F5B1E2, UniRef:UniRef100_A0A0D1L215, UniRef:UniRef50_O34857, UniRef:Ur
GO:0030435, KEGG:K06437, RefSeq:WP_072182656.1, SO:0001217, UniParc:UPI0006A9369D, UniRef:UniRef100_UPI0006A9369D, UniRef:UniRef50_O31700, UniRef:UniRef90_O31700
SO:0001217, UniRef:UniRef50_O31701, UniRef:UniRef90_G4NVP4
EC:2.7.7.80, GO:0004792, GO:0005524, GO:0005737, GO:0005829, GO:0006777, GO:0008146, GO:0008641, GO:0016779, GO:0046872, GO:0061605, SO:0001217, UniRef:UniRef50_O31702, UniRef:UniRef90_O31702
EC:2.10.1.1, GO:0005737, GO:0005829, GO:0006777, GO:0046872, GO:0061599, GO:1990586, RefSeq:WP_014113632.1, SO:0001217, UniParc:UPI00022BBA36, UniRef:UniRef100_G4NWK9, UniRef:UniRef50_O31703, Ur
GO:0003824, GO:0005525, GO:0006777, SO:0001217, UniRef:UniRef50_O31704, UniRef:UniRef90_UPI001575EF63
EC:2.8.1.12, GO:0005829, GO:0006777, GO:0030366, KEGG:K03635, RefSeq:WP_014113634.1, SO:0001217, UniParc:UPI00022BBA38, UniRef:UniRef100_G4NWL1, UniRef:UniRef50_O31705, UniRef:UniRef90_O31705
GO:0000166, GO:0003824, GO:0006777, GO:1990133, RefSeq:WP_072182652.1, SO:0001217, UniParc:UPI0006A8FEFA, UniRef:UniRef100_A0A9Q4E3S9, UniRef:UniRef50_O31706, UniRef:UniRef90_O31706
GO:0005524, GO:0005886, GO:0016887, GO:0034040, GO:0055085, GO:0140359, RefSeq:WP_072182651.1, SO:0001217, UniParc:UPI0006A82E71, UniRef:UniRef100_UPI0006A82E71, UniRef:UniRef50_O31707, UniRef:U
GO:0005524, GO:0005886, GO:0016887, GO:0034040, GO:0055085, GO:0140359, SO:0001217, UniRef:UniRef50_O31708, UniRef:UniRef90_O31708
BlastRules:WP_003232360, GO:0005886, KEGG:K24951, RefSeq:WP_072182649.1, SO:0001217, UniParc:UPI0006A88B3E, UniRef:UniRef100_UPI0006A88B3E, UniRef:UniRef50_O31709, UniRef:UniRef90_O31709
SO:0001217, UniRef:UniRef50_UPI001927D6F3, UniRef:UniRef90_UPI001927D6F3
GO:0005886, GO:0022857, GO:0042802, KEGG:K24950, SO:0001217, UniRef:UniRef50_O31710, UniRef:UniRef90_O31710
EC:7.6.2.-, GO:0005524, GO:0005886, GO:0016887, GO:0022857, GO:0055085, SO:0001217, UniRef:UniRef50_O31711, UniRef:UniRef90_O31711
SO:0001217, UniRef:UniRef50_I2NNV6
BlastRules:WP_003232354, GO:0005886, GO:0022857, GO:0042802, GO:0045121, GO:2001070, KEGG:K24948, SO:0001217, UniRef:UniRef50_O31712, UniRef:UniRef90_O31712
GO:0003677, GO:0003700, SO:0001217, UniRef:UniRef50_O31713, UniRef:UniRef90_O31713
EC:2.7.1.56, GO:0005524, GO:0005829, GO:0008443, GO:0008662, RefSeq:WP_079288731.1, SO:0001217, UniParc:UPI0009A358BB, UniRef:UniRef100_UPI0009A358BB, UniRef:UniRef50_O31714, UniRef:UniRef90_O317
SO:0001217, UniRef:UniRef50_A0A7U9J8R3
SO:0001217, UniRef:UniRef50_A0A7U9J7E4
EC:3.4.21.89, GO:0004252, GO:0005886, GO:0006465, GO:0009306, GO:0016020, KEGG:K03100, SO:0001217, UniRef:UniRef50_P28628, UniRef:UniRef90_P71013
SO:0001217, UniRef:UniRef50_UPI00102EC34E
GO:0005886, RefSeq:WP_003222329.1, SO:0001217, UniParc:UPI0001D28468, UniRef:UniRef100_A0A9Q4DMS5, UniRef:UniRef50_O31715, UniRef:UniRef90_O31715
SO:0001217, UniRef:UniRef50_R5Y8E8, UniRef:UniRef90_UPI00022BACA5
SO:0001217, UniRef:UniRef50_R5Y8E8, UniRef:UniRef90_UPI00022BACA5
SO:0001217, UniRef:UniRef50_O31717, UniRef:UniRef90_UPI00374DC73C
SO:0001217, UniRef:UniRef50_A0A150NET9
SO:0001217, UniRef:UniRef50_A0A1V5KX59
SO:0001217, UniRef:UniRef50_A0A7V7B457
RefSeq:WP_003238865.1, SO:0001217, UniParc:UPI0000060334, UniRef:UniRef100_Q45492, UniRef:UniRef50_Q45492, UniRef:UniRef90_Q45492
BlastRules:WP_003245743, COG:COG1077, COG:DZ, GO:0000902, GO:0005524, GO:0005737, GO:0008360, GO:0045121, SO:0001217, UniRef:UniRef50_P39763, UniRef:UniRef90_P39763
BlastRules:WP_003244728, GO:0003677, RefSeq:WP_041520942.1, SO:0001217, UniParc:UPI0005A1E437, UniRef:UniRef100_A0A9Q4E6G3, UniRef:UniRef50_P08874, UniRef:UniRef90_P39758
BlastRules:WP_003232333, EC:2.7.13.3, GO:0000155, GO:0005524, GO:0005886, GO:0006355, GO:0030435, GO:0045121, SO:0001217, UniRef:UniRef50_P39764, UniRef:UniRef90_P39764
BlastRules:WP_003222311, GO:0005886, GO:0006813, GO:0008324, RefSeq:WP_003222311.1, SO:0001217, UniParc:UPI0000060339, UniRef:UniRef100_P39760, UniRef:UniRef50_P39760, UniRef:UniRef90_P39760
EC:3.5.4.2, GO:0000034, GO:0006146, GO:0006168, SO:0001217, UniRef:UniRef50_P39761, UniRef:UniRef90_P39761
COG:COG0595, COG:J, EC:3.1.-.-, GO:0003723, GO:0004521, GO:0004534, GO:0005737, GO:0006364, GO:0006397, GO:0008270, GO:0042802, RefSeq:WP_061187390.1, SO:0001217, UniParc:UPI0006A895E9, UniRef:U
BlastRules:WP_003222303, EC:2.7.7.6, GO:0000428, GO:0001054, GO:0001055, GO:0001056, GO:0001057, GO:0001058, GO:0001066, GO:0003677, RefSeq:WP_014113654.1, SO:0001217, UniParc:UPI00022BBBE5, Uni
EC:3.1.3.-, GO:0000287, GO:0005829, GO:0016791, KEGG:K21064, SO:0001217, UniRef:UniRef50_Q45494, UniRef:UniRef90_Q45494
EC:3.5.1.88, GO:0006412, GO:0036211, GO:0042586, GO:0043686, GO:0046872, KEGG:K01462, RefSeq:WP_072182639.1, SO:0001217, UniParc:UPI0006A8A5B6, UniRef:UniRef100_UPI0006A8A5B6, UniRef:UniRef50_Q4
GO:0016020, RefSeq:WP_003222298.1, SO:0001217, UniParc:UPI0001D2845B, UniRef:UniRef100_A0A9Q4HJR3, UniRef:UniRef50_C0H409, UniRef:UniRef90_C0H409
SO:0001217, UniRef:UniRef50_P21884, UniRef:UniRef90_UPI00035D9B0E
GO:0004739, GO:0006086, GO:0045254, RefSeq:WP_072183596.1, SO:0001217, UniParc:UPI0006A85305, UniRef:UniRef100_UPI0006A85305, UniRef:UniRef50_P21881, UniRef:UniRef90_Q1RS58
BlastRules:WP_000068166, BlastRules:WP_003232313, BlastRules:WP_010874748, BlastRules:WP_033016213, COG:C, COG:COG0022, EC:1.2.4.1, GO:0004739, GO:0005576, GO:0005737, RefSeq:WP_072183597.1, SO:
BlastRules:WP_000863426, BlastRules:WP_003232311, EC:2.3.1.12, GO:0004149, GO:0004742, GO:0005829, GO:0006099, RefSeq:WP_072183598.1, SO:0001217, UniParc:UPI0006A80910, UniRef:UniRef100_UPI0006A8
COG:C, COG:COG1249, EC:1.8.1.4, GO:0004148, GO:0005737, GO:0050660, KEGG:K00382, RefSeq:WP_014113661.1, SO:0001217, UniParc:UPI00022BBBEB, UniRef:UniRef100_G4NWP5, UniRef:UniRef50_P21880, UniR
BlastRules:WP_003245672, GO:0005886, RefSeq:WP_061187393.1, SO:0001217, UniParc:UPI0006A889EE, UniRef:UniRef100_A0A9Q4E7X8, UniRef:UniRef50_P39910, UniRef:UniRef90_P39910
RFAM:RF02376, SO:0000655
BlastRules:WP_003244780, BlastRules:WP_011053233, EC:4.1.1.19, GO:0005737, GO:0008295, GO:0008792, GO:0009446, RefSeq:WP_014113664.1, SO:0001217, UniParc:UPI00022BBBEE, UniRef:UniRef100_G4NWP9,
RefSeq:WP_003221303.1, SO:0001217, UniParc:UPI0001D287E8, UniRef:UniRef100_A0A9Q4DKZ2, UniRef:UniRef50_Q81MS3, UniRef:UniRef90_Q45497
RefSeq:WP_003221304.1, SO:0001217, UniParc:UPI0001D287E9, UniRef:UniRef100_A0A9Q4E8Y7, UniRef:UniRef50_Q45498, UniRef:UniRef90_Q45498
SO:0001217, UniParc:UPI0001CE3367, UniRef:UniRef100_O31719, UniRef:UniRef50_O31719, UniRef:UniRef90_O31719
EC:3.1.3.25, EC:3.1.3.5, GO:0006020, GO:0007165, GO:0008934, GO:0046854, GO:0046872, GO:0106411, KEGG:K01092, RefSeq:WP_014113666.1, SO:0001217, UniParc:UPI00022BBBF0, UniRef:UniRef100_G4NWQ3, U
SO:0001217, UniRef:UniRef50_O31720
EC:2.1.1.-, GO:0008168, GO:0032259, RefSeq:WP_088679062.1, SO:0001217, UniParc:UPI0006A894F9, UniRef:UniRef100_UPI0006A894F9, UniRef:UniRef50_Q45500, UniRef:UniRef90_Q45500
BlastRules:WP_003245026, EC:3.4.24.28, GO:0004222, GO:0005576, GO:0006508, GO:0046872, RefSeq:WP_072183576.1, SO:0001217, UniParc:UPI0006A8B725, UniRef:UniRef100_UPI0006A8B725, UniRef:UniRef50_P0
SO:0001217, UniRef:UniRef50_O07625, UniRef:UniRef50_UPI001E5EA5E0, UniRef:UniRef90_G4NWQ7
RefSeq:WP_072183303.1, SO:0001217, UniParc:UPI0006A8C32B, UniRef:UniRef100_UPI0006A8C32B, UniRef:UniRef50_O07626, UniRef:UniRef90_E0U3Z2
SO:0001217, UniRef:UniRef50_A0A0D1J8V3, UniRef:UniRef90_A0A0D1J8V3
SO:0001217, UniRef:UniRef50_O07628, UniRef:UniRef50_UPI002570B016, UniRef:UniRef90_G4NWR0
RefSeq:WP_088679064.1, SO:0001217, UniParc:UPI000869B229, UniRef:UniRef100_UPI000869B229, UniRef:UniRef50_O07629, UniRef:UniRef90_UPI002DB91AF5
GO:0005886, RefSeq:WP_003221324.1, SO:0001217, UniParc:UPI0001D287F4, UniRef:UniRef100_A0A9Q4E7W8, UniRef:UniRef50_O07630, UniRef:UniRef90_O07630
GO:0003924, GO:0005525, GO:0006412, GO:0006950, SO:0001217, UniRef:UniRef50_A0A0R2DHU7, UniRef:UniRef90_UPI0002D476B4
GO:0005886, RefSeq:WP_014113676.1, SO:0001217, UniParc:UPI00022BBBFA, UniRef:UniRef100_G4NWR4, UniRef:UniRef50_O07632, UniRef:UniRef90_O07632
RefSeq:WP_003221329.1, SO:0001217, UniParc:UPI0001D287F7, UniRef:UniRef100_A0A9Q4DL02, UniRef:UniRef50_O07633, UniRef:UniRef90_O07633
GO:0016020, GO:0030435, RefSeq:WP_014113677.1, SO:0001217, UniParc:UPI00022BBBFB, UniRef:UniRef100_G4NWR6, UniRef:UniRef50_O07634, UniRef:UniRef90_O07634
SO:0001217, UniRef:UniRef50_UPI002DBFC089
SO:0001217, UniRef:UniRef50_A0A2D5QI97
RefSeq:WP_014113679.1, SO:0001217, UniParc:UPI00022BBBFD, UniRef:UniRef100_G4NWR8, UniRef:UniRef50_O07636, UniRef:UniRef90_O07636
SO:0001217, UniRef:UniRef50_W4QR38
SO:0001217, UniRef:UniRef50_W4QNX2, UniRef:UniRef90_UPI0015566334
RefSeq:WP_003221339.1, SO:0001217, UniParc:UPI0001D287FC, UniRef:UniRef100_A0A9Q4DLW3, UniRef:UniRef50_O07638, UniRef:UniRef90_O07638
BlastRules:WP_009967159, EC:2.4.99.28, GO:0005886, GO:0008360, GO:0008955, GO:0009252, GO:0015648, GO:0032153, GO:0051301, GO:0071555, KEGG:K03588, RefSeq:WP_072183310.1, SO:0001217, UniParc:UPI
EC:6.4.1.1, GO:0004736, GO:0005524, GO:0005737, GO:0006090, GO:0006094, GO:0046872, KEGG:K01958, SO:0001217, UniRef:UniRef50_Q9KWU4, UniRef:UniRef90_Q9KWU4
BlastRules:WP_003245869, EC:1.17.99.9, GO:0005886, GO:0006784, GO:0016653, GO:0046872, KEGG:K02259, RefSeq:WP_072183312.1, SO:0001217, UniParc:UPI0006A85FAD, UniRef:UniRef100_UPI0006A85FAD, UniR
EC:2.5.1.141, GO:0005886, GO:0006783, GO:0008495, GO:0016020, GO:0048034, SO:0001217, UniRef:UniRef50_P24009, UniRef:UniRef90_P24009
EC:7.1.1.9, GO:0004129, GO:0005507, GO:0005886, GO:0016020, GO:0020037, GO:0042773, KEGG:K02275, RefSeq:WP_014113685.1, SO:0001217, UniParc:UPI00022BA424, UniRef:UniRef100_G4NWS7, UniRef:UniRef5
EC:7.1.1.9, GO:0004129, GO:0005886, GO:0006119, GO:0009060, GO:0015990, GO:0020037, GO:0022904, GO:0046872, SO:0001217, UniRef:UniRef50_P16262, UniRef:UniRef90_P24010
SO:0001217, UniRef:UniRef50_A0A5S9M6A6
EC:7.1.1.9, GO:0004129, GO:0005886, GO:0009319, GO:0009486, GO:0015990, GO:0019646, SO:0001217, UniRef:UniRef50_P24012, UniRef:UniRef90_P24012
EC:7.1.1.9, GO:0004129, GO:0005886, GO:0016020, RefSeq:WP_061187406.1, SO:0001217, UniParc:UPI0006A86D85, UniRef:UniRef100_UPI0006A86D85, UniRef:UniRef50_P24013, UniRef:UniRef90_P24013
GO:0005886, KEGG:K02862, RefSeq:WP_014113687.1, SO:0001217, UniParc:UPI00022BA427, UniRef:UniRef100_G4NWT1, UniRef:UniRef50_O34329, UniRef:UniRef90_O34329
RefSeq:WP_014113688.1, SO:0001217, UniParc:UPI00022BA428, UniRef:UniRef100_A0A9Q4EAZ7, UniRef:UniRef50_O34743, UniRef:UniRef90_O34743
RefSeq:WP_014113689.1, SO:0001217, UniParc:UPI00022BA429, UniRef:UniRef100_G4NWT3, UniRef:UniRef50_O34682, UniRef:UniRef90_O34682
GO:0005886, SO:0001217, UniRef:UniRef50_O34586, UniRef:UniRef90_O34586
RefSeq:WP_014113692.1, SO:0001217, UniParc:UPI00022BA42C, UniRef:UniRef100_G4NWT6, UniRef:UniRef50_O34880, UniRef:UniRef90_O34880
RefSeq:WP_014113693.1, SO:0001217, UniParc:UPI00022BA42D, UniRef:UniRef100_A0A2M8T426, UniRef:UniRef50_O34958, UniRef:UniRef90_O34958
BlastRules:WP_003221370, GO:0005737, GO:0030420, GO:0030435, RefSeq:WP_003221370.1, SO:0001217, UniParc:UPI0000060368, UniRef:UniRef100_O34412, UniRef:UniRef50_O34412, UniRef:UniRef90_O34412

SO:0001217, UniRef:UniRef50_Q81W41, UniRef:UniRef90_UPI002FD9A96E
RFAM:RF00516, SO:0000140
EC:2.1.1.-, EC:2.1.1.171, GO:0008168, GO:0031167, GO:0052913, RefSeq:WP_014113694.1, SO:0001217, UniParc:UPI00022BA42E, UniRef:UniRef100_G4NS24, UniRef:UniRef50_O34331, UniRef:UniRef90_O34331
EC:2.7.7.3, GO:0004595, GO:0005524, GO:0005737, GO:0015937, KEGG:K00954, RefSeq:WP_003221378.1, SO:0001217, UniParc:UPI0001D288D1, UniRef:UniRef100_A0A9Q4E9J0, UniRef:UniRef50_O34797, UniRef:UniF
BlastRules:WP_003245492, GO:0005886, GO:0016020, GO:0030435, GO:0030436, SO:0001217, UniRef:UniRef50_O34765, UniRef:UniRef90_O34765
GO:0004622, GO:0016042, GO:0046470, RefSeq:WP_061187409.1, SO:0001217, UniParc:UPI0006A81141, UniRef:UniRef100_A0A9Q4E986, UniRef:UniRef50_O34731, UniRef:UniRef90_O34731
BlastRules:WP_003245451, EC:3.4.21.-, GO:0004176, GO:0004252, GO:0005524, GO:0005886, GO:0006508, GO:0030163, RefSeq:WP_072183051.1, SO:0001217, UniParc:UPI0006A8B875, UniRef:UniRef100_UPI0006A8E
EC:6.3.4.-, GO:0000049, GO:0005524, GO:0005737, GO:0006400, GO:0016879, KEGG:K27343, RefSeq:WP_072183050.1, SO:0001217, UniParc:UPI0006A84D0E, UniRef:UniRef100_UPI0006A84D0E, UniRef:UniRef50_O34
RefSeq:WP_072183048.1, SO:0001217, UniParc:UPI0006A8196A, UniRef:UniRef100_UPI0006A8196A, UniRef:UniRef50_O34445, UniRef:UniRef90_O34445
COG:COG0333, COG:J, GO:0003735, GO:0006412, GO:0015934, RefSeq:WP_003154457.1, SO:0001217, UniParc:UPI0000060371, UniRef:UniRef100_O34687, UniRef:UniRef50_O34687, UniRef:UniRef90_O34687
BlastRules:WP_003232210, GO:0003677, KEGG:K06314, RefSeq:WP_061187413.1, SO:0001217, UniParc:UPI0006A91855, UniRef:UniRef100_UPI0006A91855, UniRef:UniRef50_O34549, UniRef:UniRef90_O34549
EC:2.3.1.-, GO:0008080, GO:0016747, RefSeq:WP_014113703.1, SO:0001217, UniParc:UPI00022BA437, UniRef:UniRef100_G4NS34, UniRef:UniRef50_O34468, UniRef:UniRef90_O34468
EC:1.1.1.169, GO:0005737, GO:0008677, GO:0015940, GO:0050661, RefSeq:WP_088679067.1, SO:0001217, UniParc:UPI000B534A2C, UniRef:UniRef100_UPI000B534A2C, UniRef:UniRef50_O34661, UniRef:UniRef90_O3
EC:6.-.-.-, GO:0003824, GO:0016874, GO:0071793, KEGG:K22136, SO:0001217, UniRef:UniRef50_P55342, UniRef:UniRef90_P55342
GO:0000976, GO:0003700, GO:0005737, GO:0006355, GO:0009295, GO:2000143, SO:0001217, UniParc:UPI0001CE344C, UniRef:UniRef100_P55343, UniRef:UniRef50_P55343, UniRef:UniRef90_P55343
EC:2.1.1.199, GO:0005737, GO:0016434, GO:0070475, GO:0071424, KEGG:K03438, RefSeq:WP_014113706.1, SO:0001217, UniParc:UPI00022BA43A, UniRef:UniRef100_G4NS38, UniRef:UniRef50_Q07876, UniRef:UniRef
GO:0000910, GO:0005886, GO:0016020, GO:0032153, GO:0043093, KEGG:K03586, RefSeq:WP_003221405.1, SO:0001217, UniParc:UPI0001D288DC, UniRef:UniRef100_A0A9Q4E3F4, UniRef:UniRef50_Q07867, UniRef:U
PFAM:PF03717.20
SO:0001217, UniRef:UniRef50_A0A4U2MID5
EC:3.4.16.4, GO:0005886, GO:0006508, GO:0008360, GO:0008658, GO:0009002, GO:0009252, GO:0030435, GO:0030436, GO:0071555, KEGG:K08384, SO:0001217, UniRef:UniRef50_Q03524, UniRef:UniRef90_Q03524
EC:6.3.2.13, GO:0000287, GO:0005524, GO:0005737, GO:0008360, GO:0008765, GO:0009058, GO:0009252, GO:0016874, GO:0051301, GO:0071555, KEGG:K01928, SO:0001217, UniRef:UniRef50_Q03523, UniRef:UniRe
SO:0001217, UniRef:UniRef50_A0A7C7D3P0
EC:2.7.8.13, GO:0005886, GO:0008360, GO:0008963, GO:0009252, GO:0016020, GO:0046872, GO:0051301, GO:0051992, GO:0071555, KEGG:K01000, RefSeq:WP_003238695.1, SO:0001217, UniParc:UPI00022BA43E, U
EC:6.3.2.9, GO:0005524, GO:0005737, GO:0008360, GO:0008764, GO:0009252, GO:0051301, GO:0071555, KEGG:K01925, RefSeq:WP_088679068.1, SO:0001217, UniParc:UPI000B535A4A, UniRef:UniRef100_UPI000B53
COG:COG0772, COG:D, GO:0005886, GO:0008360, GO:0009252, GO:0015648, GO:0016020, GO:0030435, GO:0030436, GO:0032153, GO:0051301, KEGG:K03588, SO:0001217, UniRef:UniRef50_P07373, UniRef:UniRef9
EC:2.4.1.227, GO:0005886, GO:0005975, GO:0008360, GO:0009252, GO:0030259, GO:0050511, GO:0051301, GO:0051991, GO:0071555, KEGG:K02563, RefSeq:WP_061187422.1, SO:0001217, UniParc:UPI0006A8C281,
EC:1.3.1.98, GO:0005829, GO:0008360, GO:0008762, GO:0009252, GO:0050660, GO:0051301, GO:0071555, GO:0071949, SO:0001217, UniRef:UniRef50_P18579, UniRef:UniRef90_P18579
BlastRules:WP_003232178, GO:0005886, GO:0030435, GO:0032153, GO:0043093, RefSeq:WP_003221428.1, SO:0001217, UniParc:UPI00000B0291, UniRef:UniRef100_Q45546, UniRef:UniRef50_P16655, UniRef:UniRef90_
GO:0030435, GO:0051301, RefSeq:WP_014113714.1, SO:0001217, UniParc:UPI00022BA5AC, UniRef:UniRef100_G4NS49, UniRef:UniRef50_C0SPA3, UniRef:UniRef90_C0SPA3
GO:0005886, SO:0001217, UniRef:UniRef50_Q45544, UniRef:UniRef90_Q45544
GO:0005886, RefSeq:WP_010334164.1, SO:0001217, UniParc:UPI00022BA5AE, UniRef:UniRef100_A0A9Q4ELJ2, UniRef:UniRef50_P28265, UniRef:UniRef90_P28265
GO:0005515, GO:0005524, GO:0009898, GO:0016887, GO:0030435, GO:0032153, GO:0043093, GO:0051301, RefSeq:WP_014113716.1, SO:0001217, UniParc:UPI00022BA5AF, UniRef:UniRef100_G4NS52, UniRef:UniRef5
KEGG:K03531, SO:0001217, UniRef:UniRef50_A0A5S9M961, UniRef:UniRef90_A0A5S9M961
SO:0001217, UniRef:UniRef50_A0A5S9M7M0
EC:3.4.21.-, GO:0004252, GO:0005576, GO:0006508, SO:0001217, UniRef:UniRef50_P16397, UniRef:UniRef90_P16397
EC:3.4.23.-, GO:0004190, GO:0005886, GO:0006508, GO:0008233, GO:0030435, GO:0030436, KEGG:K06383, SO:0001217, UniParc:UPI00022BA5B2, UniRef:UniRef100_G4NS56, UniRef:UniRef50_P13801, UniRef:UniRef9
COG:COG5811, COG:DK, GO:0003677, GO:0003700, GO:0006352, GO:0016987, GO:0030435, KEGG:K03091, RefSeq:WP_003221446.1, SO:0001217, UniParc:UPI0000134844, UniRef:UniRef100_P06222, UniRef:UniRef50
GO:0003677, GO:0003700, GO:0006352, GO:0006355, GO:0016987, GO:0030435, KEGG:K03091, RefSeq:WP_003221449.1, SO:0001217, UniParc:UPI0001D289A7, UniRef:UniRef100_A0A9Q4E4K5, UniRef:UniRef50_P19
EC:7.-.-.-, GO:0005524, GO:0016887, GO:0042626, GO:0043190, SO:0001217, UniRef:UniRef50_O31723, UniRef:UniRef90_O31723
RFAM:RF00059, SO:0000035
EC:3.5.1.-, EC:3.5.1.16, GO:0009228, GO:0009229, GO:0016787, GO:0046872, KEGG:K20895, RefSeq:WP_072183039.1, SO:0001217, UniParc:UPI0006A80343, UniRef:UniRef100_UPI0006A80343, UniRef:UniRef50_O317
GO:0030435, RefSeq:WP_003221455.1, SO:0001217, UniParc:UPI0001D289AA, UniRef:UniRef100_A0A9Q4HLC7, UniRef:UniRef50_O31725, UniRef:UniRef90_O31725
EC:2.4.2.1, GO:0004000, GO:0005507, GO:0016491, GO:0017061, GO:0046936, KEGG:K05810, RefSeq:WP_072183038.1, SO:0001217, UniParc:UPI0006A93EFC, UniRef:UniRef100_UPI0006A93EFC, UniRef:UniRef50_O3
GO:0030170, KEGG:K06997, SO:0001217, UniRef:UniRef50_O31727, UniRef:UniRef90_O31727
GO:0000917, GO:0005737, GO:0043093, RefSeq:WP_014113725.1, SO:0001217, UniParc:UPI00022BA5B8, UniRef:UniRef100_A0A9Q4DL32, UniRef:UniRef50_O31728, UniRef:UniRef90_A7Z4F9
GO:0005886, KEGG:K02221, RefSeq:WP_003221463.1, SO:0001217, UniParc:UPI000006038F, UniRef:UniRef100_O31729, UniRef:UniRef50_O31729, UniRef:UniRef90_O31729
GO:0000049, GO:0003735, GO:0005737, GO:0015935, GO:0019843, GO:0042274, GO:0043023, GO:0072344, GO:1990112, RefSeq:WP_072566243.1, SO:0001217, UniParc:UPI000909BE6A, UniRef:UniRef100_UPI000909
BlastRules:WP_001131611, BlastRules:WP_003221468, COG:COG3599, COG:D, GO:0000917, GO:0005737, GO:0030435, GO:0042802, RefSeq:WP_003221468.1, SO:0001217, UniParc:UPI0000060391, UniRef:UniRef100_
RFAM:RF00230, SO:0000140
EC:6.1.1.5, GO:0000049, GO:0000166, GO:0002161, GO:0004822, GO:0005524, GO:0005829, GO:0006428, RefSeq:WP_014113727.1, SO:0001217, UniParc:UPI00022BA5BB, UniRef:UniRef100_G4NS70, UniRef:UniRef50_
BlastRules:WP_003245479, GO:0008270, RefSeq:WP_072183037.1, SO:0001217, UniParc:UPI0006A80853, UniRef:UniRef100_UPI0006A80853, UniRef:UniRef50_Q45478, UniRef:UniRef90_Q45478
EC:3.4.23.36, GO:0004190, GO:0005886, GO:0006508, GO:0009306, RefSeq:WP_014113729.1, SO:0001217, UniParc:UPI00022BA5BD, UniRef:UniRef100_G4NS72, UniRef:UniRef50_Q45479, UniRef:UniRef90_Q45479
EC:5.4.99.-, GO:0000455, GO:0003723, GO:0009982, GO:0120159, KEGG:K06180, SO:0001217, UniRef:UniRef50_Q45480, UniRef:UniRef90_Q45480
RFAM:RF00515, SO:0005836
SO:0001217, UniRef:UniRef50_A0A929EG79
RFAM:RF00515, SO:0005836
BlastRules:WP_003221479, GO:0005886, GO:0015505, GO:0098721, KEGG:K02824, SO:0001217, UniRef:UniRef50_P39766, UniRef:UniRef90_P39766
RFAM:RF00515, SO:0005836
EC:2.1.3.2, GO:0004070, GO:0006207, GO:0006520, GO:0016597, GO:0044205, RefSeq:WP_072183035.1, SO:0001217, UniParc:UPI0006A92602, UniRef:UniRef100_UPI0006A92602, UniRef:UniRef50_P05654, UniRef:UniF
EC:3.5.2.3, GO:0004038, GO:0004151, GO:0005737, GO:0006145, GO:0006221, GO:0008270, GO:0044205, GO:0046872, SO:0001217, UniRef:UniRef50_Q81WF0, UniRef:UniRef90_P25995
PFAM:PF00988.28
SO:0001217, UniRef:UniRef50_U1FFB4
SO:0001217, UniRef:UniRef50_A0A4Q1ZSM6
SO:0001217, UniRef:UniRef50_A0A376E3B5
GO:0009055, GO:0016491, GO:0044205, GO:0046872, GO:0050660, GO:0051537, SO:0001217, UniRef:UniRef50_P25983, UniRef:UniRef90_P25983
EC:1.3.1.14, GO:0004152, GO:0004589, GO:0005737, GO:0006207, GO:0006221, GO:0044205, SO:0001217, UniRef:UniRef50_P25996, UniRef:UniRef90_P25996
SO:0001217, UniRef:UniRef50_UPI0033659D85
EC:4.1.1.23, GO:0004590, GO:0005829, GO:0006207, GO:0044205, RefSeq:WP_061187436.1, SO:0001217, UniParc:UPI00077230CA, UniRef:UniRef100_UPI00077230CA, UniRef:UniRef50_P25971, UniRef:UniRef90_P259
EC:2.4.2.10, GO:0000287, GO:0004588, GO:0006222, GO:0019856, GO:0044205, RefSeq:WP_061187437.1, SO:0001217, UniParc:UPI0006A8B858, UniRef:UniRef100_UPI0006A8B858, UniRef:UniRef50_P25972, UniRef:Ur
RFAM:RF00162, SO:0000140
EC:1.8.4.10, EC:1.8.4.8, GO:0003824, GO:0004604, GO:0005737, GO:0019344, GO:0019379, GO:0043866, GO:0046872, GO:0051539, GO:0070814, RefSeq:WP_003221498.1, SO:0001217, UniParc:UPI0001CE3524, UniRe
SO:0001217, UniRef:UniRef50_A0A150MSF5, UniRef:UniRef90_UPI001F61E2B8
GO:0005315, GO:0005886, GO:0035435, KEGG:K16331, SO:0001217, UniRef:UniRef50_O34734, UniRef:UniRef90_O34734
EC:2.7.7.4, GO:0000103, GO:0004781, GO:0005524, GO:0070814, RefSeq:WP_014113740.1, SO:0001217, UniParc:UPI00022BA737, UniRef:UniRef100_G4NS86, UniRef:UniRef50_O34764, UniRef:UniRef90_O34764
EC:2.7.1.25, GO:0000103, GO:0004020, GO:0005524, GO:0070814, RefSeq:WP_061187438.1, SO:0001217, UniParc:UPI0006A92266, UniRef:UniRef100_A0A9Q4E3C3, UniRef:UniRef50_P0A6J1, UniRef:UniRef90_O34577
EC:2.1.1.107, GO:0004851, GO:0009236, GO:0019354, GO:0032259, RefSeq:WP_072183030.1, SO:0001217, UniParc:UPI0006A89A47, UniRef:UniRef100_UPI0006A89A47, UniRef:UniRef50_P29928, UniRef:UniRef90_O347
EC:4.99.1.4, GO:0019354, GO:0046872, GO:0051266, SO:0001217, UniRef:UniRef50_O34632, UniRef:UniRef90_O34632
SO:0001217, UniRef:UniRef50_UPI0022A2E28A
RefSeq:WP_072183028.1, SO:0001217, UniParc:UPI0006A92C0B, UniRef:UniRef100_UPI0006A92C0B, UniRef:UniRef50_O34813, UniRef:UniRef90_A0A9Q4E4I5
BlastRules:WP_003232089, GO:0000049, GO:0019843, GO:0043023, GO:0072344, GO:1990112, RefSeq:WP_061187442.1, SO:0001217, UniParc:UPI00077271A0, UniRef:UniRef100_UPI00077271A0, UniRef:UniRef50_O34
SO:0001217, UniRef:UniRef50_A0A2T0LF58, UniRef:UniRef90_UPI0031F3130C
EC:7.2.2.10, GO:0005388, GO:0005524, GO:0005886, GO:0006816, GO:0015662, GO:0016020, GO:0016887, GO:0034220, GO:0046872, SO:0001217, UniRef:UniRef50_O34431, UniRef:UniRef90_O34431
EC:3.1.26.-, GO:0016891, SO:0001217, UniRef:UniRef50_O34441, UniRef:UniRef90_O34441
BlastRules:WP_003154355, GO:0003677, KEGG:K09777, RefSeq:WP_003154355.1, SO:0001217, UniParc:UPI000016847F, UniRef:UniRef100_Q7WY72, UniRef:UniRef50_Q7WY72, UniRef:UniRef90_Q7WY72
EC:2.7.4.8, GO:0004385, GO:0005524, GO:0005829, GO:0006163, RefSeq:WP_014113748.1, SO:0001217, UniParc:UPI00022BA73F, UniRef:UniRef100_G4NS95, UniRef:UniRef50_O34328, UniRef:UniRef90_O34328
EC:2.7.7.6, GO:0000428, GO:0001054, GO:0001055, GO:0001056, GO:0001057, GO:0001058, GO:0001066, GO:0003677, GO:0003899, GO:0006351, RefSeq:WP_003221520.1, SO:0001217, UniParc:UPI00000603AB, UniF
EC:4.1.1.36, EC:6.3.2.5, GO:0004632, GO:0004633, GO:0010181, GO:0015937, GO:0015941, GO:0046872, GO:0071513, SO:0001217, UniRef:UniRef50_O35033, UniRef:UniRef90_O35033
EC:3.6.1.-, EC:3.6.4.-, GO:0003677, GO:0003678, GO:0005524, GO:0005737, GO:0006260, GO:0006268, GO:0006269, GO:0006270, GO:0006302, GO:0006310, GO:0008270, GO:0009295, GO:0016787, GO:0032508, GO:(
EC:3.5.1.88, GO:0006412, GO:0036211, GO:0042586, GO:0043686, GO:0046872, KEGG:K01462, RefSeq:WP_014113751.1, SO:0001217, UniParc:UPI00022BA742, UniRef:UniRef100_G4NS99, UniRef:UniRef50_P94462, U
EC:2.1.2.9, GO:0004479, GO:0005737, GO:0005829, GO:0071951, RefSeq:WP_072183021.1, SO:0001217, UniParc:UPI0006A91562, UniRef:UniRef100_A0A9Q4E299, UniRef:UniRef50_P94463, UniRef:UniRef90_P94463
PFAM:PF01029.23
SO:0001217, UniRef:UniRef50_W4QB04
COG:COG0820, COG:J, EC:2.1.1.192, GO:0000049, GO:0002935, GO:0005737, GO:0008757, GO:0016433, GO:0019843, GO:0030488, GO:0046872, GO:0051539, GO:0070040, GO:0070475, KEGG:K06941, RefSeq:WP_0
EC:3.1.3.16, KEGG:K20074, SO:0001217, UniRef:UniRef50_UPI0011A4DEA1, UniRef:UniRef90_UPI0011A4DEA1
SO:0001217, UniRef:UniRef50_A0A5S9MBC3
BlastRules:WP_000904759, BlastRules:WP_003232062, EC:2.7.11.1, GO:0004672, GO:0004674, GO:0004676, GO:0004677, GO:0004679, GO:0004711, GO:0005524, GO:0005886, GO:0006468, GO:0007165, GO:0009847,
EC:3.6.1.-, GO:0003924, GO:0005525, GO:0005737, GO:0006412, GO:0019843, GO:0042274, GO:0043022, GO:0046872, KEGG:K06949, SO:0001217, UniRef:UniRef50_O34530, UniRef:UniRef90_O34530
EC:3.1.3.100, EC:3.6.1.-, GO:0003924, GO:0005525, GO:0006412, GO:0043022, KEGG:K06949, SO:0001217, UniRef:UniRef50_UPI00227DCB92, UniRef:UniRef90_UPI00227DCB92
EC:5.1.3.1, GO:0004750, GO:0005829, GO:0005975, GO:0006098, GO:0009052, GO:0019323, GO:0046872, KEGG:K01783, RefSeq:WP_072183016.1, SO:0001217, UniParc:UPI0006A86016, UniRef:UniRef100_UPI0006A8
EC:2.7.6.2, GO:0004788, GO:0005524, GO:0006772, GO:0009229, GO:0016301, GO:0030975, KEGG:K00949, RefSeq:WP_014113758.1, SO:0001217, UniParc:UPI00022BA749, UniRef:UniRef100_G4NSI8, UniRef:UniRef50
COG:COG5844, COG:D, GO:0016020, GO:0030435, KEGG:K06414, RefSeq:WP_003221545.1, SO:0001217, UniParc:UPI00000603B5, UniRef:UniRef100_P37817, UniRef:UniRef50_P37817, UniRef:UniRef90_P37817
COG:COG0227, COG:J, GO:0003735, GO:0005840, GO:0006412, GO:1990904, RefSeq:WP_003221548.1, SO:0001217, UniParc:UPI00000603B6, UniRef:UniRef100_P37807, UniRef:UniRef50_P37807, UniRef:UniRef90_P3
COG:COG1302, COG:S, RefSeq:WP_003221550.1, SO:0001217, UniParc:UPI0001D28A68, UniRef:UniRef100_A0A9Q4DL08, UniRef:UniRef50_O34318, UniRef:UniRef90_O34318
GO:0004371, GO:0006071, SO:0001217, UniRef:UniRef50_O34751, UniRef:UniRef90_O34751
EC:4.3.1.17, GO:0003941, GO:0006094, GO:0006565, GO:0046872, GO:0051539, KEGG:K01752, RefSeq:WP_019714380.1, SO:0001217, UniParc:UPI000307260F, UniRef:UniRef100_A0A5D4NE21, UniRef:UniRef50_O346
EC:4.3.1.17, GO:0003941, GO:0006094, GO:0006565, GO:0046872, GO:0051539, KEGG:K01752, RefSeq:WP_014113761.1, SO:0001217, UniParc:UPI00022BA74C, UniRef:UniRef100_G4NSJ3, UniRef:UniRef50_O34607, U
PFAM:PF17191.10
EC:3.6.4.12, EC:5.6.2.4, GO:0000400, GO:0003676, GO:0003678, GO:0003689, GO:0005524, GO:0005737, GO:0006281, GO:0006310, GO:0009295, GO:0009378, GO:0016853, GO:0016887, GO:0036121, GO:0061749, GO
SO:0001217, UniRef:UniRef50_A0A1Y0GXM3, UniRef:UniRef90_A0A5S9M8P1
GO:0003677, GO:0003700, GO:0006633, GO:0045717, GO:0045892, SO:0001217, UniRef:UniRef50_O34835, UniRef:UniRef50_UPI0020D22FB4, UniRef:UniRef90_O34835
GO:0003700, GO:0045717, GO:0045892, SO:0001217, UniRef:UniRef50_O34835, UniRef:UniRef90_UPI0000E66C91
EC:2.3.1.274, GO:0005737, GO:0006633, GO:0008610, GO:0008654, GO:0043811, KEGG:K03621, SO:0001217, UniRef:UniRef50_P71018, UniRef:UniRef90_P71018
EC:2.3.1.39, GO:0004314, GO:0006633, SO:0001217, UniRef:UniRef50_A0A740PMJ5, UniRef:UniRef90_E0TTQ8
EC:1.1.1.100, GO:0004316, GO:0006633, GO:0016616, GO:0030497, GO:0051287, SO:0001217, UniRef:UniRef50_P51831, UniRef:UniRef90_P51831
COG:COG0236, COG:I, GO:0000035, GO:0000036, GO:0005829, GO:0006633, GO:0009245, GO:0016020, RefSeq:WP_003154310.1, SO:0001217, UniParc:UPI00000603BF, UniRef:UniRef100_P80643, UniRef:UniRef50_P8
BlastRules:WP_001146873, BlastRules:WP_003232030, EC:3.1.26.3, GO:0003723, GO:0003725, GO:0004525, GO:0005737, GO:0006364, GO:0006396, GO:0006397, GO:0008033, GO:0010468, GO:0019843, GO:0046872,
SO:0001217, UniRef:UniRef50_A0A378M996
GO:0005524, GO:0007059, GO:0030261, KEGG:K03529, SO:0001217, UniRef:UniRef50_UPI0022811A3C, UniRef:UniRef90_UPI0022811A3C
EC:3.6.5.4, GO:0003924, GO:0005047, GO:0005525, GO:0005737, GO:0005886, GO:0006605, GO:0006614, GO:0009306, GO:0016887, KEGG:K03110, RefSeq:WP_014113769.1, SO:0001217, UniParc:UPI00022BA8CE, Ur
KEGG:K09787, RefSeq:WP_003238590.1, SO:0001217, UniParc:UPI00000603C4, UniRef:UniRef100_P37104, UniRef:UniRef50_P37104, UniRef:UniRef90_P37104
COG:COG0541, COG:U, EC:3.6.5.4, GO:0003924, GO:0005048, GO:0005525, GO:0006614, GO:0008312, GO:0009306, GO:0016887, GO:0048500, GO:0048501, KEGG:K03106, RefSeq:WP_014113771.1, SO:0001217, Uni
COG:COG0228, COG:J, GO:0000314, GO:0003735, GO:0005737, GO:0006412, GO:0015935, GO:0022627, RefSeq:WP_003220815.1, SO:0001217, UniParc:UPI00000603C6, UniRef:UniRef100_P21474, UniRef:UniRef50_P
RefSeq:WP_072183007.1, SO:0001217, UniParc:UPI0006A80053, UniRef:UniRef100_UPI0006A80053, UniRef:UniRef50_O31739, UniRef:UniRef90_O31739
GO:0005737, GO:0005840, GO:0006364, GO:0042274, GO:0043022, RefSeq:WP_061187452.1, SO:0001217, UniParc:UPI0006A8FF8D, UniRef:UniRef100_A0A9Q4E444, UniRef:UniRef50_O31740, UniRef:UniRef90_O31740
EC:2.1.1.228, GO:0002939, GO:0005829, GO:0030488, GO:0052906, KEGG:K00554, SO:0001217, UniRef:UniRef50_O31741, UniRef:UniRef90_O31741
SO:0001217, UniRef:UniRef50_A0A3B9K4I4
RFAM:RF00556, SO:0000140
COG:COG0335, COG:J, GO:0000311, GO:0003735, GO:0006412, GO:0022625, KEGG:K02884, RefSeq:WP_003220825.1, SO:0001217, UniParc:UPI0000133C99, UniRef:UniRef100_O31742, UniRef:UniRef50_O31742, UniF
GO:0003723, GO:0003924, GO:0005525, GO:0005737, GO:0006412, GO:0042254, RefSeq:WP_072566265.1, SO:0001217, UniParc:UPI00086ECD1B, UniRef:UniRef100_UPI00086ECD1B, UniRef:UniRef50_O31743, UniRef
SO:0001217, UniRef:UniRef50_W7AUC7
SO:0001217, UniRef:UniRef50_O31745
GO:0009306, GO:0016020, RefSeq:WP_014113777.1, SO:0001217, UniParc:UPI00022BA8D6, UniRef:UniRef100_G4NSL5, UniRef:UniRef50_O34867, UniRef:UniRef90_O34867
COG:C, COG:COG0045, EC:6.2.1.5, GO:0000287, GO:0003824, GO:0004775, GO:0004776, GO:0005524, GO:0005829, GO:0006099, GO:0006104, GO:0042709, GO:0046872, RefSeq:WP_019714397.1, SO:0001217, UniPa
EC:6.2.1.5, GO:0000166, GO:0003824, GO:0004775, GO:0004776, GO:0006099, GO:0009361, RefSeq:WP_003238566.1, SO:0001217, UniParc:UPI00000603D1, UniRef:UniRef100_P80865, UniRef:UniRef50_P80865, UniRe
GO:0005737, GO:0009294, GO:0030420, GO:1990814, RefSeq:WP_061187457.1, SO:0001217, UniParc:UPI0006A8C466, UniRef:UniRef100_UPI0006A8C466, UniRef:UniRef50_P39813, UniRef:UniRef90_P39813

COG:COG0551, COG:L, EC:5.6.2.1, GO:0003677, GO:0003917, GO:0005694, GO:0005737, GO:0006260, GO:0006265, GO:0046872, KEGG:K03168, SO:0001217, UniRef:UniRef50_P39814, UniRef:UniRef90_P39814
EC:2.1.1.74, GO:0002098, GO:0005737, GO:0005829, GO:0008033, GO:0030488, GO:0047151, GO:0050660, RefSeq:WP_014113783.1, SO:0001217, UniParc:UPI00022BA8DC, UniRef:UniRef100_G4NSM2, UniRef:UniRef5
GO:0003677, GO:0006310, GO:0007059, GO:0015074, GO:0051301, RefSeq:WP_014113784.1, SO:0001217, UniParc:UPI00022BA8DD, UniRef:UniRef100_G4NSM3, UniRef:UniRef50_P39776, UniRef:UniRef90_A0A9P1JG
BlastRules:WP_003238555, EC:3.4.21.-, EC:3.4.25.2, GO:0004298, GO:0005839, GO:0008236, GO:0009376, GO:0046872, GO:0051603, KEGG:K01419, RefSeq:WP_003238555.1, SO:0001217, UniParc:UPI00000603D6, Ur
EC:3.4.25.2, GO:0004176, GO:0005524, GO:0006508, GO:0009376, SO:0001217, UniRef:UniRef50_A0A2W4JS96, UniRef:UniRef90_UPI00228293DD
EC:3.4.25.2, GO:0004176, GO:0005524, GO:0006508, GO:0008233, GO:0009376, GO:0016887, GO:0036402, GO:0043335, GO:0051603, SO:0001217, UniRef:UniRef50_P39778, UniRef:UniRef90_P39778
BlastRules:WP_000421288, BlastRules:WP_003220850, COG:COG4465, COG:K, GO:0003677, GO:0003700, GO:0005525, GO:0005737, GO:0045892, KEGG:K03706, RefSeq:WP_003220850.1, SO:0001217, UniParc:UPI00
GO:0009288, GO:0030694, GO:0044780, GO:0071973, GO:0071978, RefSeq:WP_014113786.1, SO:0001217, UniParc:UPI00022BA8DF, UniRef:UniRef100_G4NSM7, UniRef:UniRef50_P24500, UniRef:UniRef90_P24500
GO:0009288, GO:0030694, GO:0044780, GO:0071973, GO:0071978, RefSeq:WP_072182136.1, SO:0001217, UniParc:UPI0006A8C88A, UniRef:UniRef100_UPI0006A8C88A, UniRef:UniRef50_P24501, UniRef:UniRef90_P24
GO:0003774, GO:0005198, GO:0009425, GO:0009431, GO:0044780, GO:0071973, GO:0071978, SO:0001217, UniRef:UniRef50_P24502, UniRef:UniRef90_P24502
GO:0001539, GO:0003774, GO:0005198, GO:0005886, GO:0009431, GO:0044780, GO:0071978, KEGG:K02409, RefSeq:WP_072182134.1, SO:0001217, UniParc:UPI0006A84127, UniRef:UniRef100_UPI0006A84127, UniRe
GO:0003774, GO:0005886, GO:0006935, GO:0009288, GO:0009425, GO:0044780, GO:0071973, GO:0071978, KEGG:K02410, RefSeq:WP_003220860.1, SO:0001217, UniParc:UPI0001D2834B, UniRef:UniRef100_A0A9Q4E
GO:0005829, GO:0015031, GO:0044780, GO:0071978, KEGG:K02411, RefSeq:WP_072182133.1, SO:0001217, UniParc:UPI0006A8F039, UniRef:UniRef100_UPI0006A8F039, UniRef:UniRef50_P23449, UniRef:UniRef90_P23
PFAM:PF02874.29
PFAM:PF00006.31
EC:7.1.2.2, GO:0005524, GO:0005737, GO:0016887, GO:0030254, GO:0030257, GO:0044780, GO:0045261, GO:0046933, GO:0046961, GO:0071973, GO:0071978, KEGG:K02412, SO:0001217, UniRef:UniRef50_P23445, U
GO:0003774, GO:0005886, GO:0006935, GO:0009288, GO:0015031, GO:0044780, GO:0071973, GO:0071978, SO:0001217, UniRef:UniRef50_P20487, UniRef:UniRef90_P20487
GO:0044781, RefSeq:WP_072182132.1, SO:0001217, UniParc:UPI0006A8E7B0, UniRef:UniRef100_UPI0006A8E7B0, UniRef:UniRef50_P23454, UniRef:UniRef90_P23454
KEGG:K02414, SO:0001217, UniRef:UniRef50_P23451, UniRef:UniRef90_UPI00142472E6
SO:0001217, UniRef:UniRef50_P23455, UniRef:UniRef90_A0A9Q4H8W3
GO:0005829, GO:0009288, GO:0009424, GO:0030694, GO:0044780, GO:0071978, RefSeq:WP_014113794.1, SO:0001217, UniParc:UPI00022BA8E7, UniRef:UniRef100_G4NSN8, UniRef:UniRef50_P23446, UniRef:UniRef90
GO:0005886, GO:0006935, GO:0009425, GO:0071973, GO:0071978, KEGG:K02415, RefSeq:WP_072566272.1, SO:0001217, UniParc:UPI0006A876B8, UniRef:UniRef100_A0A9Q4E714, UniRef:UniRef50_P23452, UniRef:Ur
GO:0001539, GO:0003774, GO:0005886, GO:0009288, GO:0009425, GO:0044780, GO:0050918, GO:0071978, SO:0001217, UniRef:UniRef50_P23453, UniRef:UniRef90_P23453
EC:3.-.-.-, GO:0003774, GO:0004721, GO:0005886, GO:0006935, GO:0009425, GO:0016020, GO:0016311, GO:0044780, GO:0071973, GO:0071978, GO:1902021, RefSeq:WP_061187464.1, SO:0001217, UniParc:UPI0006
BlastRules:WP_003244957, GO:0000160, GO:0005737, GO:0006935, GO:0046872, GO:0071978, RefSeq:WP_003220882.1, SO:0001217, UniParc:UPI0001D28356, UniRef:UniRef100_A0A9Q4DMT1, UniRef:UniRef50_P240
GO:0005886, GO:0044781, RefSeq:WP_072183478.1, SO:0001217, UniParc:UPI0006A86778, UniRef:UniRef100_UPI0006A86778, UniRef:UniRef50_P35536, UniRef:UniRef90_P35536
GO:0005886, GO:0009306, GO:0009425, GO:0016020, GO:0044780, GO:0071978, KEGG:K02419, RefSeq:WP_003220886.1, SO:0001217, UniParc:UPI0001D28358, UniRef:UniRef100_G4NSP5, UniRef:UniRef50_P35528, U
GO:0005886, GO:0009306, GO:0009425, GO:0016020, GO:0044780, GO:0071978, KEGG:K02420, RefSeq:WP_014113798.1, SO:0001217, UniParc:UPI00022BAA57, UniRef:UniRef100_A0A2M8T3L7, UniRef:UniRef50_P35
GO:0005886, GO:0006605, GO:0009425, GO:0016020, GO:0044780, GO:0071978, KEGG:K02421, RefSeq:WP_072183479.1, SO:0001217, UniParc:UPI0006A8B452, UniRef:UniRef100_UPI0006A8B452, UniRef:UniRef50_P
GO:0005886, GO:0009306, GO:0015031, GO:0016020, GO:0044780, GO:0071978, KEGG:K02401, RefSeq:WP_061187468.1, SO:0001217, UniParc:UPI0006A92D31, UniRef:UniRef100_UPI0006A92D31, UniRef:UniRef50_P
GO:0005886, GO:0009306, GO:0016020, GO:0044780, GO:0071978, KEGG:K02400, RefSeq:WP_072183480.1, SO:0001217, UniParc:UPI0006A940B2, UniRef:UniRef100_UPI0006A940B2, UniRef:UniRef50_P35620, UniRef
GO:0003924, GO:0005047, GO:0005525, GO:0005886, GO:0006605, GO:0006614, GO:0015031, GO:0042802, GO:0044781, KEGG:K02404, RefSeq:WP_072183481.1, SO:0001217, UniParc:UPI0006A8C199, UniRef:UniRef
BlastRules:WP_010886507, GO:0005524, GO:0005829, GO:0009898, GO:0016887, GO:0044781, SO:0001217, UniRef:UniRef50_P40742, UniRef:UniRef90_P40742
SO:0001217, UniRef:UniRef50_I7KUY7
BlastRules:WP_003245734, SO:0001217, UniRef:UniRef50_P29072, UniRef:UniRef90_E0TUD2
BlastRules:WP_003231938, KEGG:K03408, SO:0001217, UniRef:UniRef50_P39802, UniRef:UniRef90_A0A9Q4DN32
BlastRules:WP_003231935, EC:3.-.-.-, GO:0016787, GO:0050918, SO:0001217, UniRef:UniRef50_P40403, UniRef:UniRef90_P40403
BlastRules:WP_003244852, EC:3.5.1.44, GO:0006935, GO:0050568, KEGG:K03411, RefSeq:WP_003220909.1, SO:0001217, UniParc:UPI0001D2842A, UniRef:UniRef100_A0A9Q4E412, UniRef:UniRef50_P40404, UniRef:Un
BlastRules:WP_003220911, GO:0003677, GO:0003899, GO:0006352, GO:0006355, GO:0016987, GO:0071978, KEGG:K02405, RefSeq:WP_003220911.1, SO:0001217, UniParc:UPI00000603F6, UniRef:UniRef100_P10726, U
SO:0001217, UniRef:UniRef50_P40405, UniRef:UniRef90_G4NSQ8
COG:COG0052, COG:J, GO:0003735, GO:0006412, GO:0015935, GO:0022627, KEGG:K02967, RefSeq:WP_072566278.1, SO:0001217, UniParc:UPI00086F325E, UniRef:UniRef100_UPI00086F325E, UniRef:UniRef50_P214
COG:COG0264, COG:J, GO:0003746, GO:0005737, GO:0006414, RefSeq:WP_014113808.1, SO:0001217, UniParc:UPI00022BAA61, UniRef:UniRef100_G4NSR0, UniRef:UniRef50_P80700, UniRef:UniRef90_P80700
SO:0001217, UniRef:UniRef50_A0A3B8JVX0
GO:0005737, GO:0006412, GO:0006415, GO:0008135, GO:0043023, RefSeq:WP_003220926.1, SO:0001217, UniParc:UPI0001D2842D, UniRef:UniRef100_A0A9Q4E4D1, UniRef:UniRef50_P81101, UniRef:UniRef90_P81101
EC:2.5.1.-, GO:0000287, GO:0005829, GO:0008834, GO:0016094, GO:0016765, GO:0030145, SO:0001217, UniRef:UniRef50_O31751, UniRef:UniRef90_O31751
BlastRules:WP_003231924, EC:2.7.7.41, GO:0004605, GO:0005886, GO:0016024, KEGG:K00981, SO:0001217, UniRef:UniRef50_O31752, UniRef:UniRef90_O31752
EC:1.1.1.267, GO:0005737, GO:0008299, GO:0019288, GO:0030145, GO:0030604, GO:0046872, GO:0051484, GO:0070402, KEGG:K00099, RefSeq:WP_061187474.1, SO:0001217, UniParc:UPI0006A90CE0, UniRef:UniRef
EC:3.4.24.-, GO:0004222, GO:0005886, GO:0006508, GO:0016020, GO:0046872, SO:0001217, UniParc:UPI00022BAA65, UniRef:UniRef100_G4NV85, UniRef:UniRef50_O31754, UniRef:UniRef90_O31754
EC:6.1.1.15, GO:0002161, GO:0004827, GO:0005524, GO:0005737, GO:0005829, GO:0006433, GO:0016740, KEGG:K01881, RefSeq:WP_072182689.1, SO:0001217, UniParc:UPI0006A839AE, UniRef:UniRef100_UPI0006A
PFAM:PF01336.31, PFAM:PF11490.13
EC:2.7.7.7, GO:0003677, GO:0003887, GO:0005737, GO:0006260, GO:0006261, GO:0008408, GO:0009295, GO:0046677, KEGG:K03763, SO:0001217, UniRef:UniRef50_P13267, UniRef:UniRef90_P13267
GO:0000028, GO:0005829, GO:0006412, GO:0042274, RefSeq:WP_003220940.1, SO:0001217, UniParc:UPI0001D28434, UniRef:UniRef100_A0A9Q4DN26, UniRef:UniRef50_P32726, UniRef:UniRef90_P32726
COG:COG0195, COG:K, GO:0003700, GO:0003723, GO:0005829, GO:0006353, GO:0031564, SO:0001217, UniRef:UniRef50_P32727, UniRef:UniRef90_P32727
BlastRules:WP_009967250, KEGG:K07742, RefSeq:WP_003220944.1, SO:0001217, UniParc:UPI0001D28436, UniRef:UniRef100_G4NV90, UniRef:UniRef50_P32728, UniRef:UniRef90_P32728
GO:0003723, GO:0003735, GO:0042254, GO:1990904, RefSeq:WP_072182686.1, SO:0001217, UniParc:UPI0006A92123, UniRef:UniRef100_UPI0006A92123, UniRef:UniRef50_P32729, UniRef:UniRef90_P32729
GO:0003743, GO:0003924, GO:0005525, GO:0005737, GO:0005829, GO:0006413, RefSeq:WP_041520956.1, SO:0001217, UniParc:UPI0002D4968F, UniRef:UniRef100_A0A9Q4E2T6, UniRef:UniRef50_P17889, UniRef:UniR
COG:COG1550, COG:S, KEGG:K09764, RefSeq:WP_003220950.1, SO:0001217, UniParc:UPI0000060406, UniRef:UniRef100_P32730, UniRef:UniRef50_P32730, UniRef:UniRef90_P32730
GO:0005829, GO:0006364, GO:0030490, GO:0043024, KEGG:K02834, RefSeq:WP_019258420.1, SO:0001217, UniParc:UPI0003802006, UniRef:UniRef100_A0A2M8T3I9, UniRef:UniRef50_P32731, UniRef:UniRef90_P3273
SO:0001217, UniRef:UniRef50_UPI002E1E50AA
EC:5.4.99.25, GO:0001522, GO:0003723, GO:0006400, GO:0009451, GO:0009982, GO:0031119, GO:0160148, GO:1990481, KEGG:K03177, SO:0001217, UniRef:UniRef50_P32732, UniRef:UniRef90_P32732
EC:2.7.1.26, EC:2.7.7.2, GO:0003919, GO:0005524, GO:0006747, GO:0006771, GO:0008531, GO:0009231, GO:0009398, KEGG:K11753, RefSeq:WP_072182683.1, SO:0001217, UniParc:UPI0006A94184, UniRef:UniRef100
COG:COG0184, COG:J, GO:0003735, GO:0005840, GO:0006412, GO:0019843, GO:0022627, RefSeq:WP_003154182.1, SO:0001217, UniParc:UPI000159767A, UniRef:UniRef100_A7Z4T9, UniRef:UniRef50_P21473, UniRef
COG:COG1185, COG:J, EC:2.7.7.8, GO:0000175, GO:0000287, GO:0003723, GO:0004654, GO:0005829, GO:0006396, GO:0006401, GO:0006402, GO:0030420, KEGG:K00962, RefSeq:WP_014113820.1, SO:0001217, UniP
GO:0005975, GO:0016810, RefSeq:WP_014113821.1, SO:0001217, UniParc:UPI00022BABD8, UniRef:UniRef100_A0A9Q4HL82, UniRef:UniRef50_P50850, UniRef:UniRef90_P50850
SO:0001217, UniRef:UniRef50_UPI003001ABCE
SO:0001217, UniRef:UniRef50_UPI003002FF60, UniRef:UniRef90_UPI002280AA2D
SO:0001217, UniRef:UniRef50_UPI00227FB75A, UniRef:UniRef90_UPI00227FB75A
GO:0030435, RefSeq:WP_003238478.1, SO:0001217, UniParc:UPI00022BABDA, UniRef:UniRef100_A0A2M8T3H7, UniRef:UniRef50_Q04811, UniRef:UniRef90_Q04811
KEGG:K06410, SO:0001217, UniRef:UniRef50_A0A5S9M9E0, UniRef:UniRef90_UPI00254608D2
EC:1.3.1.-, GO:0003824, GO:0016491, GO:0030435, GO:0030436, SO:0001217, UniRef:UniRef50_Q04809, UniRef:UniRef90_Q04809
EC:1.3.1.-, GO:0003824, GO:0016491, GO:0030435, GO:0030436, KEGG:K06411, RefSeq:WP_061187482.1, SO:0001217, UniParc:UPI0006A7FF41, UniRef:UniRef100_UPI0006A7FF41, UniRef:UniRef50_Q04810, UniRef:Ur
EC:1.2.1.11, GO:0004073, GO:0009086, GO:0009088, GO:0009089, GO:0009097, GO:0019877, GO:0046983, GO:0050661, GO:0051287, GO:0071266, KEGG:K00133, RefSeq:WP_072182681.1, SO:0001217, UniParc:UPI0
EC:2.7.2.4, GO:0004072, GO:0005524, GO:0005829, GO:0009088, GO:0009089, GO:0009090, GO:0019877, RefSeq:WP_003220974.1, SO:0001217, UniParc:UPI0001D28442, UniRef:UniRef100_G4NVA5, UniRef:UniRef50_
EC:4.3.3.7, GO:0005829, GO:0008840, GO:0009089, GO:0019877, RefSeq:WP_014113826.1, SO:0001217, UniParc:UPI00022BABDE, UniRef:UniRef100_G4NVA6, UniRef:UniRef50_Q04796, UniRef:UniRef90_Q04796
EC:3.1.-.-, GO:0003723, GO:0004521, GO:0004534, GO:0005737, GO:0006364, GO:0006397, GO:0008270, SO:0001217, UniRef:UniRef50_O31760, UniRef:UniRef90_O31760
PFAM:PF17770.6
BlastRules:WP_000139812, BlastRules:WP_003245712, GO:0005737, GO:0006508, GO:0008233, GO:0015031, SO:0001217, UniRef:UniRef50_Q99171, UniRef:UniRef90_Q99171
BlastRules:WP_003231871, RefSeq:WP_041520957.1, SO:0001217, UniParc:UPI0003104B77, UniRef:UniRef100_A0A9Q4E1W2, UniRef:UniRef50_C0H413, UniRef:UniRef90_C0H413
BlastRules:WP_003245350, GO:0003677, GO:0005524, GO:0005886, GO:0007059, GO:0016887, GO:0030435, GO:0051301, RefSeq:WP_133063220.1, SO:0001217, UniParc:UPI0006A909AA, UniRef:UniRef100_UPI0006A9
SO:0001217, UniRef:UniRef50_O31761
BlastRules:WP_010886509, GO:0005886, GO:0022857, GO:0055085, SO:0001217, UniRef:UniRef50_O31762, UniRef:UniRef90_O31762
GO:0046872, RefSeq:WP_072182678.1, SO:0001217, UniParc:UPI0006A88422, UniRef:UniRef100_UPI0006A88422, UniRef:UniRef50_O31764, UniRef:UniRef90_O31764
SO:0001217, UniRef:UniRef50_A0A9X9F8B2
BlastRules:WP_003244676, EC:1.-.-.-, GO:0016616, GO:0030497, SO:0001217, UniRef:UniRef50_O31767, UniRef:UniRef90_O31767
RefSeq:WP_003220996.1, SO:0001217, UniParc:UPI0001D2851F, UniRef:UniRef100_A0A9Q4HGW4, UniRef:UniRef50_O31768, UniRef:UniRef90_I2C5B6
RefSeq:WP_003220998.1, SO:0001217, UniParc:UPI0001CE37F1, UniRef:UniRef100_A0A9Q4E2T0, UniRef:UniRef50_O31769, UniRef:UniRef90_O31769
GO:0003677, GO:0005886, KEGG:K15539, SO:0001217, UniRef:UniRef50_O31771, UniRef:UniRef90_O31771
EC:2.7.8.5, GO:0005886, GO:0006655, GO:0008444, GO:0008654, GO:0016020, GO:0046474, KEGG:K00995, SO:0001217, UniRef:UniRef50_P46322, UniRef:UniRef90_P46322
EC:3.5.1.42, KEGG:K03743, SO:0001217, UniRef:UniRef50_P46323, UniRef:UniRef90_P46323
COG:COG0468, COG:L, GO:0003677, GO:0003684, GO:0003697, GO:0005524, GO:0005829, GO:0006281, GO:0006310, GO:0008094, GO:0009432, GO:0016887, GO:0140664, KEGG:K03553, RefSeq:WP_014113838.1, S
GO:0005886, GO:0030435, RefSeq:WP_072182674.1, SO:0001217, UniParc:UPI0006A90D9C, UniRef:UniRef100_UPI0006A90D9C, UniRef:UniRef50_O31773, UniRef:UniRef90_O31773
COG:COG1418, COG:JR, EC:3.1.-.-, GO:0003723, GO:0004521, GO:0005886, GO:0006402, GO:0016020, RefSeq:WP_003221010.1, SO:0001217, UniParc:UPI0000060425, UniRef:UniRef100_O31774, UniRef:UniRef50_O3
BlastRules:WP_003245138, EC:3.1.4.16, GO:0004113, GO:0005737, GO:0008663, GO:0046872, KEGG:K09769, RefSeq:WP_003221012.1, SO:0001217, UniParc:UPI0001D28525, UniRef:UniRef100_A0A9Q4E1V2, UniRef:U
BlastRules:WP_000404341, BlastRules:WP_000427804, BlastRules:WP_003154135, COG:COG2359, COG:S, GO:0000917, GO:0003676, GO:0030435, KEGG:K06416, RefSeq:WP_003154135.1, SO:0001217, UniParc:UPI0
EC:1.1.1.103, GO:0005737, GO:0006567, GO:0008270, GO:0008743, GO:0019518, RefSeq:WP_072182673.1, SO:0001217, UniParc:UPI0006A84ED5, UniRef:UniRef100_UPI0006A84ED5, UniRef:UniRef50_O31776, UniRef:
EC:2.3.1.29, EC:2.3.1.47, GO:0008710, GO:0008890, GO:0009058, GO:0009102, GO:0030170, SO:0001217, UniRef:UniRef50_O31777, UniRef:UniRef90_O31777
COG:COG0621, COG:J, EC:2.8.4.3, GO:0005829, GO:0006400, GO:0016782, GO:0035597, GO:0046872, GO:0051539, GO:1904047, KEGG:K06168, RefSeq:WP_079287760.1, SO:0001217, UniParc:UPI0009A2681E, UniRe
BlastRules:WP_003231834, RefSeq:WP_003231834.1, SO:0001217, UniParc:UPI000006042B, UniRef:UniRef100_O31779, UniRef:UniRef50_O31779, UniRef:UniRef90_O31779
BlastRules:WP_003231833, COG:COG5880, COG:D, GO:0030435, GO:0042802, KEGG:K06328, RefSeq:WP_003221022.1, SO:0001217, UniParc:UPI0001D2852A, UniRef:UniRef100_A0A9Q4HDF7, UniRef:UniRef50_P1401
GO:0003684, GO:0005524, GO:0005829, GO:0006298, GO:0030983, GO:0140664, RefSeq:WP_072182671.1, SO:0001217, UniParc:UPI0006A8B477, UniRef:UniRef100_A0A9Q4HGV9, UniRef:UniRef50_P49849, UniRef:Uni
GO:0005524, GO:0006298, GO:0016887, GO:0030983, GO:0032300, GO:0140664, SO:0001217, UniRef:UniRef50_P49850, UniRef:UniRef90_P49850
RFAM:RF02453, SO:0000655
SO:0001217, UniRef:UniRef50_Q7WY71, UniRef:UniRef90_UPI002DBFC7DD
GO:0005886, RefSeq:WP_087987076.1, SO:0001217, UniParc:UPI00086AE6B1, UniRef:UniRef100_UPI00086AE6B1, UniRef:UniRef50_O31780, UniRef:UniRef90_O31780
KEGG:K02167, RefSeq:WP_014113847.1, SO:0001217, UniParc:UPI00022BAD8B, UniRef:UniRef100_G4NVD6, UniRef:UniRef50_O34381, UniRef:UniRef90_E0TV32
EC:3.-.-.-, GO:0005737, GO:0016787, GO:0017000, GO:0046872, RefSeq:WP_090557822.1, SO:0001217, UniParc:UPI00086F167B, UniRef:UniRef100_UPI00086F167B, UniRef:UniRef50_O34769, UniRef:UniRef90_O34769
EC:2.3.1.39, GO:0004314, GO:0005829, GO:0006633, GO:0017000, RefSeq:WP_090557824.1, SO:0001217, UniParc:UPI00086D2F3D, UniRef:UniRef100_UPI00086D2F3D, UniRef:UniRef50_O34825, UniRef:UniRef90_O348
RefSeq:WP_090557826.1, SO:0001217, UniParc:UPI000869FF2D, UniRef:UniRef100_UPI000869FF2D, UniRef:UniRef50_O34877, UniRef:UniRef90_G4NVD9
EC:2.3.1.39, GO:0004314, GO:0006633, SO:0001217, UniRef:UniRef50_O34787, UniRef:UniRef90_G4NVE0
PFAM:PF21607.2
GO:0000035, GO:0000036, GO:0005829, GO:0009245, GO:0016020, GO:0017000, RefSeq:WP_014113852.1, SO:0001217, UniParc:UPI00022BAD90, UniRef:UniRef100_G4NVE1, UniRef:UniRef50_Q7PC63, UniRef:UniRef9
EC:4.1.1.87, GO:0004315, GO:0005829, GO:0006633, GO:0016829, GO:0017000, RefSeq:WP_090557830.1, SO:0001217, UniParc:UPI000B81D75E, UniRef:UniRef100_UPI000B81D75E, UniRef:UniRef50_P40804, UniRef:U
EC:2.3.3.-, GO:0004421, GO:0005737, GO:0006084, GO:0017000, KEGG:K15311, RefSeq:WP_014113854.1, SO:0001217, UniParc:UPI00022BAD92, UniRef:UniRef100_G4NVE3, UniRef:UniRef50_P40830, UniRef:UniRef90_
EC:4.2.1.-, EC:4.2.1.17, GO:0005737, GO:0016829, GO:0017000, SO:0001217, UniRef:UniRef50_P40805, UniRef:UniRef90_P40805
EC:4.-.-.-, GO:0005737, GO:0006635, GO:0016829, GO:0017000, RefSeq:WP_014113856.1, SO:0001217, UniParc:UPI00022BAD94, UniRef:UniRef100_G4NVE5, UniRef:UniRef50_P40802, UniRef:UniRef90_P40802
SO:0001217, UniRef:UniRef50_P40806, UniRef:UniRef90_E0TV42
PFAM:PF00106.31, PFAM:PF00550.31, PFAM:PF13561.12
SO:0001217, UniRef:UniRef50_UPI001D3C73BB
SO:0001217, UniRef:UniRef50_A0A5J4KU04
SO:0001217, UniRef:UniRef50_UPI000E2F4D72, UniRef:UniRef90_UPI002E1D93F6
PFAM:PF00106.31, PFAM:PF00550.31, PFAM:PF13561.12, PFAM:PF14765.12
SO:0001217, UniRef:UniRef50_W7YI61
SO:0001217, UniRef:UniRef50_UPI00073339DA, UniRef:UniRef90_UPI00073339DA
SO:0001217, UniRef:UniRef50_UPI00366C23A9
PFAM:PF00109.32, PFAM:PF00550.31, PFAM:PF14765.12, PFAM:PF21089.3
PFAM:PF00109.32, PFAM:PF02801.28
PFAM:PF22336.2
SO:0001217, UniRef:UniRef50_UPI00227DF61E, UniRef:UniRef90_UPI0022831DF8
SO:0001217, UniRef:UniRef50_UPI0021A8493A
SO:0001217, UniRef:UniRef50_UPI00209E2CAF, UniRef:UniRef90_UPI00209E2CAF
SO:0001217, UniRef:UniRef50_W7YY16, UniRef:UniRef90_UPI0005769FF9
PFAM:PF00106.31, PFAM:PF13561.12
PFAM:PF00550.31, PFAM:PF23297.1
PFAM:PF00109.32

PFAM:PF00106.31, PFAM:PF00109.32, PFAM:PF02801.28, PFAM:PF13561.12, PFAM:PF14765.12, PFAM:PF21089.3, PFAM:PF22336.2, PFAM:PF22621.2
SO:0001217, UniRef:UniRef50_UPI002029C616
SO:0001217, UniRef:UniRef50_A0A5J4KJP9
SO:0001217, UniRef:UniRef50_UPI002FFF7925, UniRef:UniRef90_UPI002FFF7925
PFAM:PF00561.26, PFAM:PF00975.26
SO:0001217, UniRef:UniRef50_A0A8D5UEK6
SO:0001217, UniRef:UniRef50_O31786, UniRef:UniRef90_O31786
SO:0001217, UniRef:UniRef50_O31787, UniRef:UniRef90_O31787
PFAM:PF00082.27
EC:3.4.21.-, GO:0004252, GO:0005737, GO:0006508, SO:0001217, UniRef:UniRef50_O31788, UniRef:UniRef90_O31788
RefSeq:WP_041520966.1, SO:0001217, UniParc:UPI00030118C4, UniRef:UniRef100_A0A9Q4E1T4, UniRef:UniRef50_A0A4Y6A8A6, UniRef:UniRef90_A0A0C3L1T4
PFAM:PF05908.16
SO:0001217, UniRef:UniRef50_O31789, UniRef:UniRef90_A0A6H0WH96
EC:1.11.1.28, KEGG:K04063, SO:0001217, UniRef:UniRef50_O31790, UniRef:UniRef90_A0A6H0WKV2
BlastRules:WP_114272531, GO:0005886, GO:0022857, GO:0055085, KEGG:K11815, SO:0001217, UniRef:UniRef50_P0CW83, UniRef:UniRef90_P0CW83
BlastRules:WP_003221087, GO:0005886, GO:0022857, GO:0055085, KEGG:K11814, RefSeq:WP_072183726.1, SO:0001217, UniParc:UPI0006A8F831, UniRef:UniRef100_UPI0006A8F831, UniRef:UniRef50_P0CW81, UniRe
RefSeq:WP_041520968.1, SO:0001217, UniParc:UPI0002D96D94, UniRef:UniRef100_A0A9Q4HL65, UniRef:UniRef50_A0A0M3R9B2, UniRef:UniRef90_A0A9Q4HDE8
SO:0001217, UniRef:UniRef50_O31794, UniRef:UniRef90_O31794
EC:2.5.1.75, GO:0005524, GO:0005737, GO:0006400, GO:0052381, RefSeq:WP_072566310.1, SO:0001217, UniParc:UPI0008693400, UniRef:UniRef100_A0A9Q4HGU6, UniRef:UniRef50_Q81JG8, UniRef:UniRef90_O31795
GO:0003723, GO:0005829, GO:0006355, GO:0043487, GO:0045974, RefSeq:WP_003221097.1, SO:0001217, UniParc:UPI0000060447, UniRef:UniRef100_O31796, UniRef:UniRef50_O31796, UniRef:UniRef90_O31796
RefSeq:WP_061187515.1, SO:0001217, UniParc:UPI0006A89BEA, UniRef:UniRef100_UPI0006A89BEA, UniRef:UniRef50_O31797, UniRef:UniRef90_O31797
RefSeq:WP_003221102.1, SO:0001217, UniParc:UPI0001D2860E, UniRef:UniRef100_A0A9Q4DRS7, UniRef:UniRef50_O31798, UniRef:UniRef90_O31798
SO:0001217, UniRef:UniRef50_A0AAX4BEX3, UniRef:UniRef90_UPI002351BC95
SO:0001217, UniRef:UniRef50_P50620
COG:COG0208, COG:F, EC:1.17.4.1, GO:0004748, GO:0005971, GO:0009263, GO:0046872, RefSeq:WP_014113879.1, SO:0001217, UniParc:UPI00022BADA7, UniRef:UniRef100_G4NVQ0, UniRef:UniRef50_P50621, UniRe
SO:0001217, UniRef:UniRef50_P50619, UniRef:UniRef90_A0A9Q2QRZ4
EC:3.5.1.28, GO:0005576, GO:0008745, GO:0009253, GO:0030288, GO:0030420, GO:0030435, GO:0042834, GO:0043093, GO:0046872, GO:0071555, KEGG:K01448, SO:0001217, UniRef:UniRef50_Q06320, UniRef:UniRe
COG:COG0464, COG:MDT, GO:0005524, GO:0016887, GO:0030435, GO:0030436, KEGG:K06413, RefSeq:WP_061187517.1, SO:0001217, UniParc:UPI0006A92AAE, UniRef:UniRef100_A0A9Q4E7M1, UniRef:UniRef50_P27
GO:0003924, GO:0005525, GO:0005737, GO:0043022, GO:0046872, SO:0001217, UniRef:UniRef50_P94478, UniRef:UniRef90_P94478
SO:0001217, UniRef:UniRef50_W7Z892, UniRef:UniRef90_UPI00158592AC
SO:0001217, UniRef:UniRef50_A0A1V5PQ32, UniRef:UniRef90_UPI00192799FE
BlastRules:WP_000656783, BlastRules:WP_003238341, GO:0003677, GO:0003700, GO:0042802, RefSeq:WP_072183300.1, SO:0001217, UniParc:UPI0006A8F114, UniRef:UniRef100_UPI0006A8F114, UniRef:UniRef50_P37
COG:COG0174, COG:E, EC:6.3.1.2, GO:0000287, GO:0004356, GO:0005524, GO:0005737, GO:0006542, GO:0016595, GO:0043562, GO:0045892, GO:0070406, GO:0090295, GO:0140297, GO:1904797, KEGG:K01915, SC
SO:0001217, UniRef:UniRef50_G4NVQ9, UniRef:UniRef90_G4NVQ9
RefSeq:WP_041520971.1, SO:0001217, UniParc:UPI0005A187A8, UniRef:UniRef100_UPI0005A187A8, UniRef:UniRef50_G4NVR1, UniRef:UniRef90_G4NVR1
RefSeq:WP_014113891.1, SO:0001217, UniParc:UPI00022BAF1D, UniRef:UniRef100_G4NVR2, UniRef:UniRef50_A0A7G5CIR9, UniRef:UniRef90_G4NVR2
GO:0005886, GO:0006814, GO:0008643, GO:0015293, GO:0055085, RefSeq:WP_072566315.1, SO:0001217, UniParc:UPI000868E5FC, UniRef:UniRef100_UPI000868E5FC, UniRef:UniRef50_P94488, UniRef:UniRef90_P944
EC:3.2.1.37, GO:0005886, GO:0009044, GO:0045493, RefSeq:WP_090557853.1, SO:0001217, UniParc:UPI00086EA74B, UniRef:UniRef100_UPI00086EA74B, UniRef:UniRef50_P07129, UniRef:UniRef90_P94489
GO:0003677, GO:0042732, RefSeq:WP_090557854.1, SO:0001217, UniParc:UPI000B84D71B, UniRef:UniRef100_UPI000B84D71B, UniRef:UniRef50_P16557, UniRef:UniRef90_P16557
EC:5.3.1.5, GO:0000287, GO:0005737, GO:0009045, GO:0042732, KEGG:K01805, RefSeq:WP_014113895.1, SO:0001217, UniParc:UPI00022BAF21, UniRef:UniRef100_G4NVR6, UniRef:UniRef50_P21938, UniRef:UniRef90
EC:2.7.1.17, GO:0004856, GO:0005524, GO:0005997, GO:0005998, GO:0042732, KEGG:K00854, RefSeq:WP_090557856.1, SO:0001217, UniParc:UPI000868CEB5, UniRef:UniRef100_UPI000868CEB5, UniRef:UniRef50_P3
RefSeq:WP_072566319.1, SO:0001217, UniParc:UPI00086F11EA, UniRef:UniRef100_UPI00086F11EA, UniRef:UniRef50_P00694, UniRef:UniRef90_G4NVR8
RefSeq:WP_090557858.1, SO:0001217, UniParc:UPI00086F5462, UniRef:UniRef100_UPI00086F5462, UniRef:UniRef50_G4NVR9, UniRef:UniRef90_G4NVR9
GO:0005886, GO:0015293, GO:0016020, GO:0055085, SO:0001217, UniRef:UniRef50_P54723, UniRef:UniRef90_P94493
EC:5.1.1.1, GO:0005829, GO:0006522, GO:0008784, GO:0009252, GO:0030170, GO:0030632, KEGG:K01775, RefSeq:WP_090557862.1, SO:0001217, UniParc:UPI00086BCF17, UniRef:UniRef100_A0A9Q4HJI0, UniRef:Unil
SO:0001217, UniRef:UniRef50_P94495, UniRef:UniRef90_G4NVS5
BlastRules:WP_003245820, EC:3.6.1.23, GO:0000287, GO:0004170, GO:0006226, GO:0046081, RefSeq:WP_072183639.1, SO:0001217, UniParc:UPI0006A90F07, UniRef:UniRef100_A0A9Q4E8P4, UniRef:UniRef50_O3491
RefSeq:WP_157883196.1, SO:0001217, UniParc:UPI000AE73D58, UniRef:UniRef100_UPI001A7E0B0B, UniRef:UniRef50_A0A6N7R0X4, UniRef:UniRef90_G4NVS7
EC:2.1.1.45, GO:0004799, GO:0005829, GO:0006231, GO:0006235, GO:0032259, KEGG:K00560, RefSeq:WP_087987051.1, SO:0001217, UniParc:UPI000868DBA9, UniRef:UniRef100_UPI000868DBA9, UniRef:UniRef50_P(
SO:0001217, UniRef:UniRef50_G4NVT1, UniRef:UniRef90_G4NVT1
GO:0005576, SO:0001217, UniRef:UniRef50_O31803, UniRef:UniRef90_O31803
RefSeq:WP_079287793.1, SO:0001217, UniParc:UPI00086D170C, UniRef:UniRef100_A0A9Q4E904, UniRef:UniRef50_C0H417, UniRef:UniRef90_G4NVT3
RefSeq:WP_014113913.1, SO:0001217, UniParc:UPI00022BAF33, UniRef:UniRef100_G4NVT4, UniRef:UniRef50_G4NVT4, UniRef:UniRef90_G4NVT4
SO:0001217, UniRef:UniRef50_O31805
RefSeq:WP_061186406.1, SO:0001217, UniParc:UPI000772844F, UniRef:UniRef100_UPI000772844F, UniRef:UniRef50_O31806, UniRef:UniRef90_E0TVU1
RefSeq:WP_090557865.1, SO:0001217, UniParc:UPI00086CEED8, UniRef:UniRef100_UPI00086CEED8, UniRef:UniRef50_A0A2M8T389, UniRef:UniRef90_G4NVT8
RefSeq:WP_014113918.1, SO:0001217, UniParc:UPI00022BAF38, UniRef:UniRef100_G4NVT9, UniRef:UniRef50_O31807, UniRef:UniRef90_O31807
KEGG:K07240, RefSeq:WP_079287796.1, SO:0001217, UniParc:UPI00086DAF77, UniRef:UniRef100_UPI00086DAF77, UniRef:UniRef50_A0A3C0EBB9, UniRef:UniRef90_A0A9Q4DV69
RefSeq:WP_090557867.1, SO:0001217, UniParc:UPI00086CFD6C, UniRef:UniRef100_UPI00086CFD6C, UniRef:UniRef50_O31811, UniRef:UniRef90_A0A9Q4DSZ1
SO:0001217, UniRef:UniRef50_O31813
RefSeq:WP_061186399.1, SO:0001217, UniParc:UPI0006A85EBA, UniRef:UniRef100_UPI0006A85EBA, UniRef:UniRef50_O31814, UniRef:UniRef90_G4NVU7
RFAM:RF02377, SO:0000655
GO:0005886, GO:0016787, RefSeq:WP_090557869.1, SO:0001217, UniParc:UPI00086D446E, UniRef:UniRef100_UPI00086D446E, UniRef:UniRef50_O31815, UniRef:UniRef90_O31815
GO:0005886, RefSeq:WP_014113928.1, SO:0001217, UniParc:UPI00022BB0BC, UniRef:UniRef100_G4NVU9, UniRef:UniRef50_O31816, UniRef:UniRef90_O31816
EC:2.5.1.-, GO:0000287, GO:0005737, GO:0016765, GO:0046677, KEGG:K11210, NCBIFam:NF038306.1, RefSeq:WP_072566331.1, SO:0001217, UniParc:UPI00086B8D05, UniRef:UniRef100_UPI00086B8D05, UniRef:UniF
RefSeq:WP_090557871.1, SO:0001217, UniParc:UPI0008692BEB, UniRef:UniRef100_UPI0008692BEB, UniRef:UniRef50_A0A5D4NBP5, UniRef:UniRef90_A0A5D4NBP5
RFAM:RF02454, SO:0000655
COG:COG1974, COG:KT, EC:3.4.21.88, GO:0003677, GO:0004252, GO:0006260, GO:0006281, GO:0006508, GO:0009432, GO:0045892, KEGG:K01356, RefSeq:WP_003238209.1, SO:0001217, UniParc:UPI0000060479, Ur
GO:0000917, GO:0005737, GO:0006281, GO:0009432, GO:0051782, SO:0001217, UniRef:UniRef50_Q45056, UniRef:UniRef90_Q45056
GO:0000150, GO:0003677, RefSeq:WP_014113933.1, SO:0001217, UniParc:UPI00022BB0C1, UniRef:UniRef100_G4NVV5, UniRef:UniRef50_Q45057, UniRef:UniRef90_Q45057
SO:0001217, UniRef:UniRef50_UPI00017546ED, UniRef:UniRef90_UPI00017546ED
EC:2.2.1.1, GO:0004802, GO:0005829, GO:0006098, GO:0046872, KEGG:K00615, SO:0001217, UniRef:UniRef50_P45694, UniRef:UniRef90_P45694
GO:0008156, GO:0030435, RefSeq:WP_014113937.1, SO:0001217, UniParc:UPI00022BB0C5, UniRef:UniRef100_G4NVV9, UniRef:UniRef50_P45707, UniRef:UniRef90_P45707
GO:0005886, KEGG:K09976, RefSeq:WP_003221221.1, SO:0001217, UniParc:UPI000006047F, UniRef:UniRef100_P45708, UniRef:UniRef50_P45708, UniRef:UniRef90_P45708
BlastRules:WP_010886518, EC:3.1.3.-, GO:0016787, GO:0030435, GO:0043937, GO:0046983, KEGG:K06376, RefSeq:WP_024121579.1, SO:0001217, UniParc:UPI000309C52C, UniRef:UniRef100_A0A9Q4DT00, UniRef:Un
BlastRules:WP_001152684, BlastRules:WP_003231585, GO:0005886, GO:0017004, GO:0030435, RefSeq:WP_087987047.1, SO:0001217, UniParc:UPI0008696861, UniRef:UniRef100_A0A9Q4HIE5, UniRef:UniRef50_P4570
GO:0000160, RefSeq:WP_014113939.1, SO:0001217, UniParc:UPI00022BB0C7, UniRef:UniRef100_G4NVW3, UniRef:UniRef50_P45709, UniRef:UniRef90_P45709
GO:0005886, SO:0001217, UniParc:UPI00022BB0C8, UniRef:UniRef100_A0A9Q4HHS9, UniRef:UniRef50_P45710, UniRef:UniRef90_P45710
GO:0005886, SO:0001217, UniRef:UniRef50_P45711, UniRef:UniRef90_P45711
BlastRules:WP_010886520, KEGG:K06335, SO:0001217, UniParc:UPI0001D28713, UniRef:UniRef100_A0A9Q4E8Q9, UniRef:UniRef50_Q45058, UniRef:UniRef90_A0A9Q4E8Q9
GO:0030435, GO:0030436, KEGG:K06433, RefSeq:WP_003221237.1, SO:0001217, UniParc:UPI0000060486, UniRef:UniRef100_P71032, UniRef:UniRef50_P71032, UniRef:UniRef90_P71032
GO:0030435, GO:0030436, GO:0042601, KEGG:K06432, SO:0001217, UniParc:UPI00022BB0C9, UniRef:UniRef100_A0A9W5LIL8, UniRef:UniRef50_P71031, UniRef:UniRef90_P71031
SO:0001217, UniRef:UniRef50_A0A926B9G5
EC:4.2.1.3, GO:0003729, GO:0003730, GO:0003994, GO:0005829, GO:0006099, GO:0006101, GO:0019679, GO:0030350, GO:0043937, GO:0046872, GO:0047456, GO:0051539, KEGG:K27802, SO:0001217, UniRef:UniRef
SO:0001217, UniRef:UniRef50_UPI0022830553
SO:0001217, UniParc:UPI0001D28717, UniRef:UniRef100_A0A9W5LIJ2, UniRef:UniRef50_D4FXA5, UniRef:UniRef90_D4FXA5
BlastRules:WP_000527987, BlastRules:WP_003231569, GO:0030435, GO:0030436, GO:0042601, KEGG:K06431, RefSeq:WP_003221249.1, SO:0001217, UniParc:UPI0001D28718, UniRef:UniRef100_A0A9Q4DR24, UniRef:
GO:0030435, GO:0030436, KEGG:K06434, RefSeq:WP_003221250.1, SO:0001217, UniParc:UPI0001D28719, UniRef:UniRef100_A0A9Q4EBY3, UniRef:UniRef50_Q45060, UniRef:UniRef90_Q45060
EC:3.1.2.-, GO:0047617, KEGG:K07107, RefSeq:WP_014113943.1, SO:0001217, UniParc:UPI00022BB0CC, UniRef:UniRef100_G4NVX4, UniRef:UniRef50_Q45061, UniRef:UniRef90_Q45061
RefSeq:WP_003221255.1, SO:0001217, UniParc:UPI0001D2871B, UniRef:UniRef100_G4NVX5, UniRef:UniRef50_Q45062, UniRef:UniRef90_Q45062
RefSeq:WP_014113944.1, SO:0001217, UniParc:UPI00022BB0CD, UniRef:UniRef100_G4NWT9, UniRef:UniRef50_Q45063, UniRef:UniRef90_Q45063
EC:2.3.1.15, EC:2.3.1.275, GO:0005886, GO:0006654, GO:0008374, GO:0008654, GO:0043772, KEGG:K08591, RefSeq:WP_003221258.1, SO:0001217, UniParc:UPI0001D2871D, UniRef:UniRef100_G4NWU0, UniRef:UniRe
RefSeq:WP_014113945.1, SO:0001217, UniParc:UPI00022BB0CE, UniRef:UniRef100_G4NWU1, UniRef:UniRef50_Q45065, UniRef:UniRef90_Q45065
COG:COG0187, COG:L, EC:5.6.2.2, GO:0003677, GO:0003918, GO:0005524, GO:0005694, GO:0006265, GO:0007059, GO:0034335, GO:0046872, KEGG:K02470, SO:0001217, UniRef:UniRef50_Q59961, UniRef:UniRef90_
EC:5.6.2.2, GO:0003677, GO:0003918, GO:0005524, GO:0005694, GO:0005737, GO:0006265, GO:0007059, GO:0009330, GO:0019897, GO:0034335, SO:0001217, UniRef:UniRef50_Q45066, UniRef:UniRef90_Q45066
RefSeq:WP_014113948.1, SO:0001217, UniParc:UPI00022BB0D1, UniRef:UniRef100_G4NWU4, UniRef:UniRef50_Q65J07, UniRef:UniRef90_A0A9W5PD19
GO:0005886, RefSeq:WP_014113949.1, SO:0001217, UniParc:UPI00022BB0D2, UniRef:UniRef100_G4NWU5, UniRef:UniRef50_Q45067, UniRef:UniRef90_Q45067
GO:0005283, GO:0005886, KEGG:K03310, SO:0001217, UniRef:UniRef50_O31464, UniRef:UniRef90_Q45068
SO:0001217, UniRef:UniRef50_Q47096, UniRef:UniRef90_G4NWU7
RefSeq:WP_061186388.1, SO:0001217, UniParc:UPI0007727924, UniRef:UniRef100_A0A9Q4E800, UniRef:UniRef50_Q45069, UniRef:UniRef90_E0TWG5
RefSeq:WP_061186387.1, SO:0001217, UniParc:UPI00077231F6, UniRef:UniRef100_UPI00077231F6, UniRef:UniRef50_O31821, UniRef:UniRef90_G4NWU9
EC:2.7.7.9, GO:0003983, GO:0006011, KEGG:K00963, RefSeq:WP_061186386.1, SO:0001217, UniParc:UPI00077290E9, UniRef:UniRef100_UPI00077290E9, UniRef:UniRef50_A0A2R6XZ01, UniRef:UniRef90_G4NWV0
GO:0005886, GO:0071555, KEGG:K03975, SO:0001217, UniRef:UniRef50_O31823, UniRef:UniRef90_O31823
BlastRules:WP_003245472, EC:3.1.-.-, GO:0004527, GO:0005737, KEGG:K07097, RefSeq:WP_072181942.1, SO:0001217, UniParc:UPI0006A81158, UniRef:UniRef100_UPI0006A81158, UniRef:UniRef50_O31824, UniRef:Ur
EC:6.4.1.-, GO:0005524, GO:0006552, GO:0016874, GO:1905202, SO:0001217, UniRef:UniRef50_O31825, UniRef:UniRef90_O31825
EC:4.2.1.17, GO:0003824, GO:0006635, GO:0016836, SO:0001217, UniRef:UniRef50_O34893, UniRef:UniRef90_O34893
BlastRules:WP_003231525, EC:4.1.3.4, GO:0004419, GO:0006552, GO:0046872, GO:0046951, KEGG:K01640, RefSeq:WP_061186382.1, SO:0001217, UniParc:UPI0006A89878, UniRef:UniRef100_A0A9Q4HJL5, UniRef:Uni
RefSeq:WP_061186381.1, SO:0001217, UniParc:UPI00077263A2, UniRef:UniRef100_UPI00077263A2, UniRef:UniRef50_C0H419, UniRef:UniRef90_C0H419
EC:6.3.4.14, GO:0003989, GO:0004075, GO:0005524, GO:0006633, GO:0046872, GO:2001295, SO:0001217, UniRef:UniRef50_O34544, UniRef:UniRef90_O34544
PFAM:PF00289.28
PFAM:PF13193.12
SO:0001217, UniRef:UniRef50_UPI002B1ED243, UniRef:UniRef90_UPI002B1ED243
EC:1.3.99.-, GO:0003995, GO:0005737, GO:0033539, GO:0050660, SO:0001217, UniRef:UniRef50_O34421, UniRef:UniRef90_O34421
SO:0001217, UniRef:UniRef50_UPI003016F41B
EC:3.2.1.-, GO:0016798, RefSeq:WP_090557887.1, SO:0001217, UniParc:UPI00086AA98B, UniRef:UniRef100_UPI00086AA98B, UniRef:UniRef50_O35015, UniRef:UniRef90_O35015
GO:0005886, RefSeq:WP_003220186.1, SO:0001217, UniParc:UPI00000B30BB, UniRef:UniRef100_A0A9Q4HFB5, UniRef:UniRef50_O34506, UniRef:UniRef90_O34506
SO:0001217, UniRef:UniRef50_UPI002E1641F0
SO:0001217, UniRef:UniRef50_UPI00227F96F0
SO:0001217, UniRef:UniRef50_UPI002AB3C0F9
PFAM:PF13193.12
PFAM:PF00550.31
SO:0001217, UniRef:UniRef50_A0A161UC18
SO:0001217, UniRef:UniRef50_A0A8I1LTI0
SO:0001217, UniRef:UniRef50_UPI000C032DAE
SO:0001217, UniRef:UniRef50_UPI00227E1D57
PFAM:PF00202.27
SO:0001217, UniRef:UniRef50_UPI0004709CBA
EC:2.3.1.39, GO:0004314, GO:0006633, SO:0001217, UniRef:UniRef50_Q9R9J2, UniRef:UniRef90_Q9R9J2

EC:3.4.16.4, EC:3.4.21.-, GO:0004175, GO:0009002, GO:0009252, KEGG:K07259, SO:0001217, UniRef:UniRef50_A0A6H0WJ26, UniRef:UniRef90_A0A6H0WJ26
SO:0001217, UniRef:UniRef50_A0AAP3CXN9, UniRef:UniRef90_A0AAP3CQ87
GO:0005886, GO:0006811, GO:0015297, GO:0042910, RefSeq:WP_061186372.1, SO:0001217, UniParc:UPI00077240BB, UniRef:UniRef100_A0A9Q4EBG4, UniRef:UniRef50_O34474, UniRef:UniRef90_O34474
BlastRules:WP_004399558, KEGG:K23598, RefSeq:WP_061186371.1, SO:0001217, UniParc:UPI0006A8AD09, UniRef:UniRef100_UPI0006A8AD09, UniRef:UniRef50_O34841, UniRef:UniRef90_O34841
SO:0001036
GO:0003677, GO:0006310, GO:0015074, GO:0044826, GO:0046718, GO:0075713, RefSeq:WP_003220317.1, SO:0001217, UniParc:UPI0001D28BFB, UniRef:UniRef100_G4NWX2, UniRef:UniRef50_O35009, UniRef:UniRef9
GO:0003677, RefSeq:WP_061186370.1, SO:0001217, UniParc:UPI0006A89848, UniRef:UniRef100_A0A9Q4E770, UniRef:UniRef50_O34555, UniRef:UniRef90_O34555
EC:2.3.2.2, EC:3.4.19.13, GO:0005576, GO:0006508, GO:0006750, GO:0006751, GO:0036374, GO:0103068, RefSeq:WP_014113977.1, SO:0001217, UniParc:UPI00022BB26E, UniRef:UniRef100_G4NWX5, UniRef:UniRef50
SO:0001217, UniRef:UniRef50_O34685, UniRef:UniRef90_UPI00200AE899
SO:0001217, UniRef:UniRef50_O35017, UniRef:UniRef90_G4NWX7
PFAM:PF07992.20
SO:0001217, UniRef:UniRef50_A0A3B9JCQ3
EC:1.4.1.13, GO:0003824, GO:0004355, GO:0006537, GO:0008152, GO:0015930, GO:0016491, GO:0019676, GO:0046872, GO:0051538, GO:0097054, SO:0001217, UniRef:UniRef50_P39812, UniRef:UniRef90_P39812
SO:0001217, UniRef:UniRef50_A0A9E6WA82
BlastRules:WP_004399246, GO:0003677, GO:0003700, GO:0006355, GO:0006537, GO:0032993, RefSeq:WP_014113982.1, SO:0001217, UniParc:UPI00022BB3F4, UniRef:UniRef100_G4NWY0, UniRef:UniRef50_P20668, U
EC:2.7.2.11, GO:0003723, GO:0004349, GO:0005524, GO:0005829, GO:0006561, GO:0055129, RefSeq:WP_014113983.1, SO:0001217, UniParc:UPI00022BB3F5, UniRef:UniRef100_G4NWY1, UniRef:UniRef50_O07509, Ur
EC:1.5.1.2, GO:0004735, GO:0005737, GO:0006561, GO:0055129, KEGG:K00286, SO:0001217, UniParc:UPI00022BB3F6, UniRef:UniRef100_A0A9Q4HI57, UniRef:UniRef50_P0CI77, UniRef:UniRef90_P0CI77
BlastRules:WP_003220337, GO:0003677, GO:0006274, SO:0001217, UniParc:UPI0001977078, UniRef:UniRef100_P0CI76, UniRef:UniRef50_P0CI76, UniRef:UniRef90_P0CI76
EC:1.14.-.-, GO:0004497, GO:0005506, GO:0016705, GO:0020037, KEGG:K23138, SO:0001217, UniRef:UniRef50_P27632, UniRef:UniRef90_P27632
SO:0001217, UniRef:UniRef50_A0A072NJ12, UniRef:UniRef90_UPI0021A5D077
SO:0001217, UniRef:UniRef50_A0A925ZZ95
SO:0001217, UniRef:UniRef50_P28670, UniRef:UniRef90_E0TY15
SO:0001217, UniRef:UniRef50_P28671, UniRef:UniRef90_E0TY16
SO:0001217, UniRef:UniRef50_S2XN71, UniRef:UniRef90_A0A9Q4DNS4
SO:0001217, UniRef:UniRef50_D4FXG6, UniRef:UniRef90_E0TY18
SO:0001217, UniRef:UniRef50_Q97DR8, UniRef:UniRef90_UPI0025A27D4B
SO:0001217, UniRef:UniRef50_R2QQ86, UniRef:UniRef90_G4NWZ1
SO:0001217, UniRef:UniRef50_A0A1I6TWK5, UniRef:UniRef90_G4NWZ2
GO:0005886, GO:0022857, SO:0001217, UniRef:UniRef50_O05390, UniRef:UniRef90_O34864
EC:2.7.1.-, GO:0005975, GO:0016301, SO:0001217, UniRef:UniRef50_O34861, UniRef:UniRef90_O34861
EC:1.1.1.-, GO:0016616, GO:0051287, SO:0001217, UniRef:UniRef50_O34815, UniRef:UniRef90_O34815
RFAM:RF00162, SO:0000140
EC:1.-.-.-, GO:0016491, GO:0043546, GO:0046872, GO:0051536, RefSeq:WP_072183203.1, SO:0001217, UniParc:UPI0006A86423, UniRef:UniRef100_UPI0006A86423, UniRef:UniRef50_C0SP82, UniRef:UniRef90_C0SP82
RefSeq:WP_014113998.1, SO:0001217, UniParc:UPI00022BB404, UniRef:UniRef100_G4NWZ7, UniRef:UniRef50_O31829, UniRef:UniRef90_E0TY26
SO:0001217, UniRef:UniRef50_A0A0U9H813, UniRef:UniRef90_UPI002DBF596E
RefSeq:WP_230939490.1, SO:0001217, UniParc:UPI00050855C3, UniRef:UniRef100_A0A9Q4HG86, UniRef:UniRef50_O31830, UniRef:UniRef90_G4NWZ9
RefSeq:WP_003220370.1, SO:0001217, UniParc:UPI0001D28CB8, UniRef:UniRef100_A0A9Q4E8H5, UniRef:UniRef50_Q62TZ3, UniRef:UniRef90_L8E9J0
SO:0001217, UniRef:UniRef50_Q7WY68, UniRef:UniRef90_E0TY30
SO:0001217, UniRef:UniRef50_C0SP89
SO:0001217, UniRef:UniRef50_W4VMA9
BlastRules:WP_003231419, GO:0005576, RefSeq:WP_072183200.1, SO:0001217, UniParc:UPI0006A89C66, UniRef:UniRef100_UPI0006A89C66, UniRef:UniRef50_O34918, UniRef:UniRef90_O34918
GO:0005886, RefSeq:WP_088679193.1, SO:0001217, UniParc:UPI000B52BB1C, UniRef:UniRef100_UPI000B52BB1C, UniRef:UniRef50_O34343, UniRef:UniRef90_O34343
EC:4.2.2.10, GO:0005576, GO:0030570, GO:0047490, KEGG:K01732, RefSeq:WP_014114006.1, SO:0001217, UniParc:UPI00022BB40C, UniRef:UniRef100_G4NX06, UniRef:UniRef50_P94449, UniRef:UniRef90_P94449
RefSeq:WP_088679194.1, SO:0001217, UniParc:UPI000B52DB3D, UniRef:UniRef100_UPI000B52DB3D, UniRef:UniRef50_U4TX69, UniRef:UniRef90_A0A150F9L3
SO:0001217, UniRef:UniRef50_A0A9W5LF71, UniRef:UniRef90_G4NX08
RefSeq:WP_072183551.1, SO:0001217, UniParc:UPI0006A93825, UniRef:UniRef100_UPI0006A93825, UniRef:UniRef50_C7YPS5, UniRef:UniRef90_G4NX09
RefSeq:WP_072183552.1, SO:0001217, UniParc:UPI0006A92644, UniRef:UniRef100_UPI0006A92644, UniRef:UniRef50_O31916, UniRef:UniRef90_G4NX10
SO:0001217, UniRef:UniRef50_G4NX11, UniRef:UniRef90_G4NX11
RefSeq:WP_041520976.1, SO:0001217, UniParc:UPI00059EF30C, UniRef:UniRef100_UPI00059EF30C, UniRef:UniRef50_C0H420, UniRef:UniRef90_A0A9Q4HJA1
EC:4.1.1.2, GO:0005737, GO:0033609, GO:0046564, GO:0046872, KEGG:K01569, SO:0001217, UniRef:UniRef50_O34767, UniRef:UniRef90_O34767
RefSeq:WP_088679196.1, SO:0001217, UniParc:UPI000B52ABAB, UniRef:UniRef100_UPI000B52ABAB, UniRef:UniRef50_O34985, UniRef:UniRef90_E0TY43
RefSeq:WP_061186341.1, SO:0001217, UniParc:UPI0006A895E1, UniRef:UniRef100_A0A9Q4E8G4, UniRef:UniRef50_O34339, UniRef:UniRef90_A0A9Q4E8G4
SO:0001217, UniRef:UniRef50_O31832
RefSeq:WP_088679198.1, SO:0001217, UniParc:UPI000B52C9B0, UniRef:UniRef100_UPI000B52C9B0, UniRef:UniRef50_O34611, UniRef:UniRef90_O34611
RefSeq:WP_079287838.1, SO:0001217, UniParc:UPI00086D3CFE, UniRef:UniRef100_UPI00086D3CFE, UniRef:UniRef50_O31833, UniRef:UniRef90_A0A9W5LF55
RefSeq:WP_003220412.1, SO:0001217, UniParc:UPI0001D28CCB, UniRef:UniRef100_A0A5B8Z1Z8, UniRef:UniRef50_A0A166IG75, UniRef:UniRef90_G9QJK1
RefSeq:WP_088679199.1, SO:0001217, UniParc:UPI000B531803, UniRef:UniRef100_UPI000B531803, UniRef:UniRef50_O34535, UniRef:UniRef90_E0TY50
SO:0001217, UniRef:UniRef50_A0AAX1Q898
GO:0005886, KEGG:K15268, RefSeq:WP_088679201.1, SO:0001217, UniParc:UPI000B52D4A8, UniRef:UniRef100_UPI000B52D4A8, UniRef:UniRef50_O34416, UniRef:UniRef90_O34416
RefSeq:WP_088679202.1, SO:0001217, UniParc:UPI000B52B53F, UniRef:UniRef100_UPI000B52B53F, UniRef:UniRef50_C0H415, UniRef:UniRef90_A0A9Q4DUZ7
SO:0001217, UniRef:UniRef50_G4NPW2, UniRef:UniRef90_G4NPW2
RefSeq:WP_014114023.1, SO:0001217, UniParc:UPI00022BB585, UniRef:UniRef100_G4NPW4, UniRef:UniRef50_D4FX37, UniRef:UniRef90_E0TY56
RefSeq:WP_088679203.1, SO:0001217, UniParc:UPI000B5358FC, UniRef:UniRef100_UPI000B5358FC, UniRef:UniRef50_O34947, UniRef:UniRef90_E0TYV0
EC:3.5.2.6, GO:0008800, GO:0030655, NCBIFam:NF012167.0, SO:0001217, UniRef:UniRef50_P39824, UniRef:UniRef90_A0AAP3CMD7
SO:0001217, UniRef:UniRef50_G4NPW7, UniRef:UniRef90_G4NPW7
RefSeq:WP_014114028.1, SO:0001217, UniParc:UPI00022BB58A, UniRef:UniRef100_G4NPW9, UniRef:UniRef50_A0A1I4ETA5, UniRef:UniRef90_A0A9W5PBD1
GO:0046872, RefSeq:WP_072183553.1, SO:0001217, UniParc:UPI0006A92E05, UniRef:UniRef100_UPI0006A92E05, UniRef:UniRef50_O07079, UniRef:UniRef90_O07079
SO:0001217, UniRef:UniRef50_P94482, UniRef:UniRef90_A0A9Q4HBQ2
BlastRules:WP_003231377, EC:3.2.1.8, GO:0031176, GO:0045493, RefSeq:WP_014114032.1, SO:0001217, UniParc:UPI00022BB58E, UniRef:UniRef100_G9B9X7, UniRef:UniRef50_V9TXH2, UniRef:UniRef90_P18429
SO:0001217, UniRef:UniRef50_G4NPX4, UniRef:UniRef90_G4NPX4
BlastRules:WP_003244531, EC:3.2.1.73, GO:0005576, GO:0005975, GO:0042972, KEGG:K01216, RefSeq:WP_072183712.1, SO:0001217, UniParc:UPI0006A851B8, UniRef:UniRef100_UPI0006A851B8, UniRef:UniRef50_P2
SO:0001217, UniRef:UniRef50_UPI00289F2BDB
SO:0001217, UniRef:UniRef50_UPI0020C2F8C5, UniRef:UniRef90_UPI002030B39F
SO:0001217, UniRef:UniRef50_UPI000A7B7C10
EC:3.5.1.2, EC:4.3.3.6, GO:0004359, GO:0016884, GO:0042823, KEGG:K08681, RefSeq:WP_088679205.1, SO:0001217, UniParc:UPI0006A820C7, UniRef:UniRef100_UPI0006A820C7, UniRef:UniRef50_P37528, UniRef:Uni
EC:4.3.3.6, GO:0003824, GO:0042823, KEGG:K06215, RefSeq:WP_088679206.1, SO:0001217, UniParc:UPI0006A8CE58, UniRef:UniRef100_UPI0006A8CE58, UniRef:UniRef50_P37527, UniRef:UniRef90_A0A0M0LC30
KEGG:K00375, SO:0001217, UniRef:UniRef50_A0A0D0FP81, UniRef:UniRef90_G4NPY6
RefSeq:WP_088679207.1, SO:0001217, UniParc:UPI000B52FE30, UniRef:UniRef100_UPI000B52FE30, UniRef:UniRef50_UPI000B52FE30, UniRef:UniRef90_UPI000B52FE30
SO:0001217, UniRef:UniRef50_UPI003390F932
SO:0001217, UniRef:UniRef50_V9H200, UniRef:UniRef90_G4NPZ1
EC:2.3.1.79, KEGG:K00661, RefSeq:WP_014114051.1, SO:0001217, UniParc:UPI00022BB5A1, UniRef:UniRef100_G4NPZ2, UniRef:UniRef50_A0A939BRA4, UniRef:UniRef90_A0A2M8SZT9
RefSeq:WP_088679210.1, SO:0001217, UniParc:UPI0006A8125E, UniRef:UniRef100_UPI0006A8125E, UniRef:UniRef50_C0H438, UniRef:UniRef90_A0A9Y1YVV0
GO:0005886, SO:0001217, UniRef:UniRef50_O31934, UniRef:UniRef90_O31934
RefSeq:WP_133063221.1, SO:0001217, UniParc:UPI0006A84F88, UniRef:UniRef100_UPI0006A84F88, UniRef:UniRef50_O34363, UniRef:UniRef90_E0TYX2
RFAM:RF01411, SO:0000655
GO:0000049, GO:0005737, GO:0015031, RefSeq:WP_014114058.1, SO:0001217, UniParc:UPI00022BB5A8, UniRef:UniRef100_G4NPZ9, UniRef:UniRef50_P37584, UniRef:UniRef90_P37584
GO:0003700, GO:0043565, SO:0001217, UniParc:UPI00022BB5A9, UniRef:UniRef100_G4NQ00, UniRef:UniRef50_O34901, UniRef:UniRef90_O34901
EC:2.3.1.1, KEGG:K22476, RefSeq:WP_072183764.1, SO:0001217, UniParc:UPI0006A8B317, UniRef:UniRef100_UPI0006A8B317, UniRef:UniRef50_O34376, UniRef:UniRef90_UPI0009A35ADC
RefSeq:WP_014114060.1, SO:0001217, UniParc:UPI00022BB5AA, UniRef:UniRef100_G4NQ01, UniRef:UniRef50_O34892, UniRef:UniRef90_A0A9W5LF34
EC:3.-.-.-, GO:0016787, GO:0046872, RefSeq:WP_061188163.1, SO:0001217, UniParc:UPI0006A8AFAB, UniRef:UniRef100_UPI0006A8AFAB, UniRef:UniRef50_O34910, UniRef:UniRef90_O34910
RefSeq:WP_014114062.1, SO:0001217, UniParc:UPI00022BB5AC, UniRef:UniRef100_G4NQ03, UniRef:UniRef50_O34637, UniRef:UniRef90_G4NQ03
GO:0003677, GO:0003700, GO:0005737, RefSeq:WP_072183763.1, SO:0001217, UniParc:UPI0006A815C1, UniRef:UniRef100_UPI0006A815C1, UniRef:UniRef50_O34920, UniRef:UniRef90_O34920
SO:0001217, UniRef:UniRef50_P94497
GO:0003677, GO:0003700, GO:0005737, GO:0046872, SO:0001217, UniRef:UniRef50_O31844, UniRef:UniRef90_O31844
PFAM:PF13702.11
SO:0001217, UniRef:UniRef50_UPI002DBD9ABE, UniRef:UniRef90_UPI002DBD9ABE
GO:0005886, KEGG:K08976, RefSeq:WP_014114067.1, SO:0001217, UniParc:UPI00022BB5B1, UniRef:UniRef100_G4NQ08, UniRef:UniRef50_O31845, UniRef:UniRef90_O31845
RefSeq:WP_014114068.1, SO:0001217, UniParc:UPI00022BB5B2, UniRef:UniRef100_G4NQ09, UniRef:UniRef50_O34516, UniRef:UniRef90_O34516
RefSeq:WP_072183484.1, SO:0001217, UniParc:UPI0006A93036, UniRef:UniRef100_UPI0006A93036, UniRef:UniRef50_O35042, UniRef:UniRef90_O35042
SO:0001217, UniRef:UniRef50_O35046
BlastRules:WP_004399588, COG:COG3239, COG:I, EC:1.14.19.-, GO:0005886, GO:0006629, GO:0006633, GO:0016717, KEGG:K10255, SO:0001217, UniRef:UniRef50_O34653, UniRef:UniRef90_O34653
BlastRules:WP_003231271, COG:COG4585, COG:T, EC:2.7.13.3, GO:0000155, GO:0004672, GO:0004721, GO:0005524, GO:0005886, GO:0042802, GO:0046983, KEGG:K07778, SO:0001217, UniRef:UniRef50_O34757, Ur
BlastRules:WP_004399356, COG:COG2197, COG:TK, GO:0000160, GO:0003677, GO:0005737, GO:0006355, KEGG:K07693, RefSeq:WP_014114073.1, SO:0001217, UniParc:UPI00022BB5B7, UniRef:UniRef100_G4NQ14,
GO:0016020, GO:0022857, GO:0055085, SO:0001217, UniRef:UniRef50_A0A2L1UID0, UniRef:UniRef90_G4NQ15
GO:0004553, GO:0005576, GO:0009254, GO:0019867, RefSeq:WP_014114077.1, SO:0001217, UniParc:UPI00022BB713, UniRef:UniRef100_G4NQ18, UniRef:UniRef50_O34669, UniRef:UniRef90_O34669
EC:5.6.2.4, GO:0003677, GO:0003678, GO:0003689, GO:0005524, GO:0005694, GO:0005737, GO:0006260, GO:0006281, GO:0006310, GO:0009378, GO:0009432, GO:0016853, GO:0016887, GO:0030894, GO:0032508, G
RFAM:RF00013, SO:0000655
SO:0001217, UniRef:UniRef50_D5DN82
SO:0001217, UniRef:UniRef50_D5DN83
GO:0008270, RefSeq:WP_072183491.1, SO:0001217, UniParc:UPI0006A80E19, UniRef:UniRef100_UPI0006A80E19, UniRef:UniRef50_P80872, UniRef:UniRef90_P80872
RefSeq:WP_003220141.1, SO:0001217, UniParc:UPI0000060506, UniRef:UniRef100_O31847, UniRef:UniRef50_O31847, UniRef:UniRef90_O31847
RefSeq:WP_003241656.1, SO:0001217, UniParc:UPI00022BB719, UniRef:UniRef100_A0A2M8T2Y1, UniRef:UniRef50_O31848, UniRef:UniRef90_O31848
BlastRules:WP_000080288, BlastRules:WP_004399332, EC:1.2.1.3, GO:0004029, GO:0006081, RefSeq:WP_014114083.1, SO:0001217, UniParc:UPI00022BB71A, UniRef:UniRef100_G4NQ26, UniRef:UniRef50_O34660, Uni
EC:4.2.1.137, GO:0005811, GO:0005886, GO:0016104, GO:0016829, GO:0016866, KEGG:K18115, SO:0001217, UniRef:UniRef50_Q796C3, UniRef:UniRef90_Q796C3
EC:1.15.1.1, GO:0004784, GO:0046872, KEGG:K04564, RefSeq:WP_072183650.1, SO:0001217, UniParc:UPI0006A87232, UniRef:UniRef100_UPI0006A87232, UniRef:UniRef50_O35023, UniRef:UniRef90_O35023
GO:0005886, GO:0015293, RefSeq:WP_014114086.1, SO:0001217, UniParc:UPI00022BB71D, UniRef:UniRef100_G4NQ29, UniRef:UniRef50_O34383, UniRef:UniRef90_O34383
SO:0001217, UniRef:UniRef50_A0A4R1QLI1
EC:2.3.1.61, GO:0004149, GO:0006099, GO:0045252, SO:0001217, UniRef:UniRef50_P16263, UniRef:UniRef90_UPI0030005B41
EC:1.2.4.2, GO:0004591, GO:0005829, GO:0006096, GO:0006099, GO:0030976, GO:0045252, RefSeq:WP_061188149.1, SO:0001217, UniParc:UPI000772413C, UniRef:UniRef100_A0A9Q4E2Z5, UniRef:UniRef50_P23129,
RefSeq:WP_072183463.1, SO:0001217, UniParc:UPI0006A817A0, UniRef:UniRef100_UPI0006A817A0, UniRef:UniRef50_O31849, UniRef:UniRef90_O31849
GO:0005524, GO:0016887, RefSeq:WP_072183464.1, SO:0001217, UniParc:UPI0006A881D5, UniRef:UniRef100_UPI0006A881D5, UniRef:UniRef50_Q49XL1, UniRef:UniRef90_O31850
GO:0004784, GO:0005507, GO:0005886, GO:0008270, GO:0019430, GO:0042802, RefSeq:WP_072183465.1, SO:0001217, UniParc:UPI0006A88E60, UniRef:UniRef100_UPI0006A88E60, UniRef:UniRef50_O31851, UniRef:U
EC:3.4.19.11, GO:0006508, GO:0008234, GO:0008932, GO:0009986, GO:0071555, RefSeq:WP_087986980.1, SO:0001217, UniParc:UPI00086AD777, UniRef:UniRef100_UPI00086AD777, UniRef:UniRef50_O07532, UniRef:U
EC:2.4.1.-, GO:0008194, GO:0009058, GO:0016758, RefSeq:WP_088679216.1, SO:0001217, UniParc:UPI000B537B25, UniRef:UniRef100_UPI000B537B25, UniRef:UniRef50_O31853, UniRef:UniRef90_O31853
BlastRules:WP_010886531, EC:2.7.7.85, GO:0004016, GO:0005524, GO:0006171, GO:0106408, KEGG:K18672, RefSeq:WP_003220108.1, SO:0001217, UniParc:UPI0001D28984, UniRef:UniRef100_A0A9Q4HL55, UniRef:U
SO:0001217, UniParc:UPI0001D28983, UniRef:UniRef100_E0TZN8, UniRef:UniRef50_I2C6G7, UniRef:UniRef90_A0A060PP39
GO:0005886, GO:0006811, GO:0015297, GO:0016020, GO:0042910, GO:0046677, RefSeq:WP_072183468.1, SO:0001217, UniParc:UPI0006A8956E, UniRef:UniRef100_UPI0006A8956E, UniRef:UniRef50_O31855, UniRef:U
BlastRules:WP_003231216, RefSeq:WP_061188145.1, SO:0001217, UniParc:UPI0006A87DF4, UniRef:UniRef100_UPI0006A87DF4, UniRef:UniRef50_O34860, UniRef:UniRef90_O31856
EC:3.5.1.-, GO:0016811, GO:0019213, GO:0046872, RefSeq:WP_003220099.1, SO:0001217, UniParc:UPI0001D288C6, UniRef:UniRef100_A0A9Q4HF67, UniRef:UniRef50_O31857, UniRef:UniRef90_O31857
RefSeq:WP_014114096.1, SO:0001217, UniParc:UPI00022BB727, UniRef:UniRef100_G4NSA8, UniRef:UniRef50_O31858, UniRef:UniRef90_O31858
RefSeq:WP_003220095.1, SO:0001217, UniParc:UPI0001D288C4, UniRef:UniRef100_G4NSA9, UniRef:UniRef50_O68260, UniRef:UniRef90_O68260
GO:0005215, GO:0005886, GO:0006810, KEGG:K05786, RefSeq:WP_072183656.1, SO:0001217, UniParc:UPI0006A8565F, UniRef:UniRef100_UPI0006A8565F, UniRef:UniRef50_O31859, UniRef:UniRef90_O31859
SO:0001217, UniRef:UniRef50_Q7WY67, UniRef:UniRef90_A0A2M8T2W5

RefSeq:WP_014114099.1, SO:0001217, UniParc:UPI00022BB72A, UniRef:UniRef100_G4NSB2, UniRef:UniRef50_O31861, UniRef:UniRef90_E0TZP6
SO:0001217, UniRef:UniRef50_D4FXN9, UniRef:UniRef90_D4FXN9
SO:0001217, UniRef:UniRef50_D4FXP0
RefSeq:WP_061188142.1, SO:0001217, UniParc:UPI0006A8804D, UniRef:UniRef100_A0A9Q4E2Y7, UniRef:UniRef50_P94502, UniRef:UniRef90_D4FXP1
BlastRules:WP_004399427, GO:0000976, GO:0001217, GO:0032993, GO:0045892, SO:0001217, UniRef:UniRef50_O34844, UniRef:UniRef90_O34844
EC:1.-.-.-, GO:0005737, GO:0009056, GO:0009636, GO:0016491, RefSeq:WP_014114104.1, SO:0001217, UniParc:UPI00022BB72F, UniRef:UniRef100_G4NSB8, UniRef:UniRef50_P81102, UniRef:UniRef90_P81102
EC:3.1.-.-, GO:0005737, GO:0009056, GO:0009636, GO:0016787, SO:0001217, UniRef:UniRef50_O34842, UniRef:UniRef90_O34842
EC:1.13.11.-, GO:0005737, GO:0009056, GO:0009636, GO:0046872, GO:0051213, KEGG:K15975, SO:0001217, UniRef:UniRef50_O34543, UniRef:UniRef90_O34543
EC:1.13.11.-, GO:0005737, GO:0009056, GO:0009636, GO:0046872, GO:0051213, KEGG:K15975, SO:0001217, UniRef:UniRef50_O34543, UniRef:UniRef90_O34543
GO:0005886, SO:0001217, UniRef:UniRef50_C0H431, UniRef:UniRef90_C0H431
GO:0005886, GO:0022857, GO:0055085, SO:0001217, UniRef:UniRef50_O34745, UniRef:UniRef90_O34745
BlastRules:WP_003231186, EC:3.4.21.102, GO:0004175, GO:0004252, GO:0006508, GO:0007165, GO:0030288, RefSeq:WP_072183150.1, SO:0001217, UniParc:UPI0006A84AD8, UniRef:UniRef100_UPI0006A84AD8, UniR
SO:0001217, UniRef:UniRef50_O34954
GO:0016020, RefSeq:WP_014114110.1, SO:0001217, UniParc:UPI00022BB88F, UniRef:UniRef100_G4NSC5, UniRef:UniRef50_O34654, UniRef:UniRef90_O34654
BlastRules:WP_004399387, EC:3.4.-.-, GO:0004180, GO:0005886, GO:0006508, SO:0001217, UniRef:UniRef50_O34866, UniRef:UniRef90_O34866
EC:2.4.2.1, GO:0004731, GO:0005829, GO:0006139, GO:0006152, KEGG:K03784, RefSeq:WP_014114112.1, SO:0001217, UniParc:UPI00022BB891, UniRef:UniRef100_G4NSC7, UniRef:UniRef50_O34925, UniRef:UniRef90
RefSeq:WP_003218142.1, SO:0001217, UniParc:UPI0001D28E59, UniRef:UniRef100_A0A9Q4DTE9, UniRef:UniRef50_C0H432, UniRef:UniRef90_C0H432
BlastRules:WP_004399431, RefSeq:WP_003218144.1, SO:0001217, UniParc:UPI0001D28E5A, UniRef:UniRef100_A0A9Q4E1W7, UniRef:UniRef50_O30472, UniRef:UniRef90_O30472
SO:0001217, UniRef:UniRef50_O34349, UniRef:UniRef90_A0AAQ3ENG2
RefSeq:WP_003241581.1, SO:0001217, UniParc:UPI00022BB893, UniRef:UniRef100_A0A2M8T2P0, UniRef:UniRef50_O31863, UniRef:UniRef90_O31863
GO:0005886, RefSeq:WP_003218150.1, SO:0001217, UniParc:UPI0001D28E5D, UniRef:UniRef100_G4NSD2, UniRef:UniRef50_C0H433, UniRef:UniRef90_C0H433
SO:0001217, UniRef:UniRef50_O34414, UniRef:UniRef90_UPI001C638C8B
SO:0001217, UniRef:UniRef50_O31864, UniRef:UniRef90_O31864
SO:0001217, UniRef:UniRef50_C0H434
EC:5.4.3.2, GO:0046440, GO:0050066, GO:0051539, GO:1904047, SO:0001217, UniRef:UniRef50_Q9XBQ8, UniRef:UniRef90_A0A6H0WK62
EC:2.3.1.264, GO:0008080, KEGG:K21935, RefSeq:WP_090557989.1, SO:0001217, UniParc:UPI00086F7302, UniRef:UniRef100_UPI00086F7302, UniRef:UniRef50_O34895, UniRef:UniRef90_A0A2M8T2N6
EC:3.-.-.-, GO:0016787, GO:0046872, SO:0001217, UniRef:UniRef50_O34984, UniRef:UniRef90_O34984
EC:2.8.3.-, GO:0008410, SO:0001217, UniRef:UniRef50_O34466, UniRef:UniRef90_O34466
SO:0001217, UniRef:UniRef50_A0A1X9MAU4
EC:2.6.-.-, GO:0008483, GO:0030170, SO:0001217, UniRef:UniRef50_O34662, UniRef:UniRef90_O34662
BlastRules:WP_003230830, EC:2.3.1.-, GO:0016747, KEGG:K06323, RefSeq:WP_087986968.1, SO:0001217, UniParc:UPI000B4470E1, UniRef:UniRef100_A0A9Q4E3S0, UniRef:UniRef50_P42093, UniRef:UniRef90_P42093
KEGG:K06322, RefSeq:WP_327816607.1, SO:0001217, UniParc:UPI002E1F897C, UniRef:UniRef100_UPI002E1F897C, UniRef:UniRef50_P42092, UniRef:UniRef90_G4NSE4
KEGG:K06321, RefSeq:WP_061188126.1, SO:0001217, UniParc:UPI0006A918A8, UniRef:UniRef100_A0A9Q4E2K0, UniRef:UniRef50_P42091, UniRef:UniRef90_G4NSE5
RefSeq:WP_014114126.1, SO:0001217, UniParc:UPI00022BB8A0, UniRef:UniRef100_G4NSE6, UniRef:UniRef50_P42089, UniRef:UniRef90_G4NSE6
KEGG:K06320, RefSeq:WP_072566444.1, SO:0001217, UniParc:UPI00090AE622, UniRef:UniRef100_UPI00090AE622, UniRef:UniRef50_P42090, UniRef:UniRef90_G4NSE7
EC:3.1.3.8, GO:0005576, GO:0016158, RefSeq:WP_327816608.1, SO:0001217, UniParc:UPI002E1AAE5D, UniRef:UniRef100_UPI002E1AAE5D, UniRef:UniRef50_O31097, UniRef:UniRef90_P42094
EC:4.2.1.115, EC:5.1.3.-, KEGG:K15894, RefSeq:WP_014114129.1, SO:0001217, UniParc:UPI00022BB8A3, UniRef:UniRef100_G4NSE9, UniRef:UniRef50_I2C6L6, UniRef:UniRef90_E0TZT1
EC:1.8.4.12, GO:0005737, GO:0006979, GO:0030091, GO:0033743, RefSeq:WP_014114130.1, SO:0001217, UniParc:UPI00022BB8A4, UniRef:UniRef100_G4NSF0, UniRef:UniRef50_P54155, UniRef:UniRef90_P54155
EC:1.8.4.11, GO:0005737, GO:0008113, GO:0034599, GO:0036211, GO:0036456, RefSeq:WP_327816365.1, SO:0001217, UniParc:UPI002E1DC8AB, UniRef:UniRef100_UPI002E1DC8AB, UniRef:UniRef50_P54154, UniRef:U
GO:0003677, GO:0003700, GO:0006355, GO:0006950, SO:0001217, UniRef:UniRef50_P54182, UniRef:UniRef90_P54182
SO:0001217, UniRef:UniRef50_A0A8J4H354
SO:0001217, UniRef:UniRef50_UPI002DC557D0
RefSeq:WP_003218204.1, SO:0001217, UniParc:UPI0001D28E72, UniRef:UniRef100_A0A9Q4HGR9, UniRef:UniRef50_P54180, UniRef:UniRef90_D4FXT5
GO:0016020, SO:0001217, UniRef:UniRef50_P54179, UniRef:UniRef90_P54179
GO:0004622, RefSeq:WP_014114135.1, SO:0001217, UniParc:UPI00022BB8AA, UniRef:UniRef100_G4NSF6, UniRef:UniRef50_P40766, UniRef:UniRef90_P40766
BlastRules:WP_003245947, GO:0005886, GO:0046872, RefSeq:WP_072183585.1, SO:0001217, UniParc:UPI0006A8CE28, UniRef:UniRef100_UPI0006A8CE28, UniRef:UniRef50_P54178, UniRef:UniRef90_P54178
RefSeq:WP_003218212.1, SO:0001217, UniParc:UPI0001D28E76, UniRef:UniRef100_A0A9Q4E3R1, UniRef:UniRef50_P54177, UniRef:UniRef90_P54177
EC:4.3.1.19, GO:0003941, GO:0004794, GO:0006520, GO:0006565, GO:0006566, GO:0006567, GO:0009097, GO:0030170, RefSeq:WP_014114137.1, SO:0001217, UniParc:UPI00022BB8AC, UniRef:UniRef100_G4NSF9, U
SO:0001217, UniRef:UniRef50_P54156, UniRef:UniRef90_A0A9Q4ERE4
GO:0005886, GO:0140911, RefSeq:WP_014114139.1, SO:0001217, UniParc:UPI00022BB8AE, UniRef:UniRef100_G4NSG1, UniRef:UniRef50_P54175, UniRef:UniRef90_P54175
RefSeq:WP_014114140.1, SO:0001217, UniParc:UPI00022BB8AF, UniRef:UniRef100_G4NSG2, UniRef:UniRef50_P54174, UniRef:UniRef90_P54174
BlastRules:WP_003230799, EC:1.5.1.3, GO:0004146, GO:0005829, GO:0006730, GO:0046452, GO:0046654, GO:0046655, GO:0050661, KEGG:K00287, SO:0001217, UniRef:UniRef50_Q54277, UniRef:UniRef90_P11045
EC:2.1.1.45, GO:0004799, GO:0005829, GO:0006231, GO:0006235, GO:0032259, RefSeq:WP_061187676.1, SO:0001217, UniParc:UPI0007729AFB, UniRef:UniRef100_UPI0007729AFB, UniRef:UniRef50_P0A884, UniRef:Ui
GO:0006629, GO:0008962, RefSeq:WP_014114143.1, SO:0001217, UniParc:UPI00022BB8B2, UniRef:UniRef100_G4NSG5, UniRef:UniRef50_P54173, UniRef:UniRef90_P54173
GO:0016020, RefSeq:WP_041520984.1, SO:0001217, UniParc:UPI0005A06F00, UniRef:UniRef100_A0A9Q4HFI4, UniRef:UniRef50_P54172, UniRef:UniRef90_P54172
EC:2.1.1.242, GO:0008990, KEGG:K15984, SO:0001217, UniRef:UniRef50_P54171, UniRef:UniRef90_P54171
BlastRules:WP_003230794, GO:0003756, GO:0006979, GO:0016491, GO:0033194, GO:0045454, KEGG:K26958, RefSeq:WP_003218231.1, SO:0001217, UniParc:UPI0001D28E80, UniRef:UniRef100_A0A9Q4DQA6, UniRef:
SO:0001217, UniRef:UniRef50_A0A2H5X8B3
GO:0016491, RefSeq:WP_072183531.1, SO:0001217, UniParc:UPI0006A7FE03, UniRef:UniRef100_UPI0006A7FE03, UniRef:UniRef50_P54169, UniRef:UniRef90_P54169
KEGG:K06950, RefSeq:WP_014114148.1, SO:0001217, UniParc:UPI00022BBA4A, UniRef:UniRef100_G4NSH1, UniRef:UniRef50_P54168, UniRef:UniRef90_G4NSH1
EC:1.-.-.-, GO:0004601, GO:0034599, KEGG:K00432, RefSeq:WP_014114149.1, SO:0001217, UniParc:UPI00022BBA4B, UniRef:UniRef100_G4NSH2, UniRef:UniRef50_P52035, UniRef:UniRef90_P52035
EC:2.3.1.31, EC:2.3.1.46, GO:0004414, GO:0005737, GO:0008899, GO:0019281, KEGG:K00651, RefSeq:WP_041520985.1, SO:0001217, UniParc:UPI0002DA9858, UniRef:UniRef100_G4NSH3, UniRef:UniRef50_Q72X44, Ui
EC:2.4.1.315, GO:0005886, GO:0009246, GO:0009247, GO:0047228, GO:0070395, KEGG:K03429, RefSeq:WP_088679235.1, SO:0001217, UniParc:UPI0006A84544, UniRef:UniRef100_UPI0006A84544, UniRef:UniRef50_P5
BlastRules:WP_003230776, COG:COG1278, COG:K, GO:0003677, GO:0005737, KEGG:K03704, RefSeq:WP_003153604.1, SO:0001217, UniParc:UPI000043D72E, UniRef:UniRef100_Q65IA2, UniRef:UniRef50_P42016, UniR
BlastRules:WP_003230774, RefSeq:WP_003218247.1, SO:0001217, UniParc:UPI0001D28F7A, UniRef:UniRef100_A0A9Q4HEE2, UniRef:UniRef50_P68731, UniRef:UniRef90_P68731
SO:0001217, UniRef:UniRef50_O32007, UniRef:UniRef90_O32007
SO:0001217, UniRef:UniRef50_P54165, UniRef:UniRef90_P54165
GO:0003676, GO:0004523, SO:0001217, UniRef:UniRef50_P54164, UniRef:UniRef90_P54164
GO:0005886, GO:0022857, GO:1990397, KEGG:K09125, SO:0001217, UniRef:UniRef50_P54163, UniRef:UniRef90_P54163
GO:0003676, GO:0004523, GO:0046872, RefSeq:WP_003218254.1, SO:0001217, UniParc:UPI0001D28F7F, UniRef:UniRef100_A0A9Q4HL32, UniRef:UniRef50_P54162, UniRef:UniRef90_P54162
GO:0030435, RefSeq:WP_003218255.1, SO:0001217, UniParc:UPI00001B093D, UniRef:UniRef100_Q7WY66, UniRef:UniRef50_Q7WY66, UniRef:UniRef90_Q7WY66
SO:0001217, UniRef:UniRef50_A0A6I7F4L2, UniRef:UniRef90_UPI00227E9017
SO:0001217, UniRef:UniRef50_A0A7W9YQJ3
RefSeq:WP_003230761.1, SO:0001217, UniParc:UPI000195C680, UniRef:UniRef100_C0H443, UniRef:UniRef50_C0H443, UniRef:UniRef90_C0H443
SO:0001217, UniRef:UniRef50_P54160, UniRef:UniRef90_P54160
SO:0001217, UniRef:UniRef50_P54159, UniRef:UniRef90_UPI000316650A
RefSeq:WP_109491777.1, SO:0001217, UniParc:UPI0006A8E79A, UniRef:UniRef100_A0A9Q4E3P1, UniRef:UniRef50_A0A1R1RG03, UniRef:UniRef90_A0A5D4NBV9
GO:0004671, GO:0005886, KEGG:K16168, RefSeq:WP_061187668.1, SO:0001217, UniParc:UPI0006A89D11, UniRef:UniRef100_UPI0006A89D11, UniRef:UniRef50_P54158, UniRef:UniRef90_P54158
SO:0001217, UniRef:UniRef50_G4NSS0, UniRef:UniRef90_G4NSS0
SO:0001217, UniRef:UniRef50_A0A150NFF7, UniRef:UniRef90_UPI0030EFA54E
SO:0001217, UniRef:UniRef50_A0A7U4KS70
EC:2.4.2.22, GO:0000310, GO:0005737, GO:0006166, GO:0032265, GO:0043101, GO:0046110, KEGG:K03816, RefSeq:WP_327816358.1, SO:0001217, UniParc:UPI002E1D154F, UniRef:UniRef100_UPI002E1D154F, UniRef:
RFAM:RF00167, SO:0000035
BlastRules:WP_000215091, BlastRules:WP_004398513, EC:3.4.17.19, GO:0004181, GO:0006508, GO:0008270, KEGG:K01299, SO:0001217, UniRef:UniRef50_P50848, UniRef:UniRef90_P50848
GO:0005886, GO:0015649, GO:0016020, GO:0046411, KEGG:K02526, SO:0001217, UniRef:UniRef50_P50847, UniRef:UniRef90_P50847
EC:4.1.2.14, GO:0005737, GO:0008675, GO:0008700, KEGG:K01625, RefSeq:WP_072182315.1, SO:0001217, UniParc:UPI0006A8276C, UniRef:UniRef100_UPI0006A8276C, UniRef:UniRef50_P50846, UniRef:UniRef90_P50
EC:2.7.1.45, GO:0005524, GO:0008673, SO:0001217, UniRef:UniRef50_P50845, UniRef:UniRef90_P50845
GO:0000976, GO:0003700, GO:0003910, GO:0005524, GO:0006281, GO:0006310, GO:0006355, SO:0001217, UniRef:UniRef50_P50844, UniRef:UniRef90_P50844
PFAM:PF00356.27, PFAM:PF01381.28, PFAM:PF13443.12
EC:5.3.1.17, GO:0008270, GO:0008697, GO:0019698, GO:0042840, GO:0045490, GO:0046872, KEGG:K01815, SO:0001217, UniRef:UniRef50_P50843, UniRef:UniRef90_P50843
EC:1.1.1.127, GO:0008678, GO:0016616, GO:0045490, GO:0047001, GO:0051287, SO:0001217, UniRef:UniRef50_P50842, UniRef:UniRef90_P50842
EC:5.6.2.3, GO:0003677, GO:0003678, GO:0003689, GO:0005524, GO:0006139, GO:0009378, GO:0016853, GO:0016887, GO:0036121, GO:0046872, GO:0051536, GO:0061749, GO:0061775, GO:0140584, GO:0140665, G
RefSeq:WP_014114171.1, SO:0001217, UniParc:UPI00022BBA62, UniRef:UniRef100_G4NST2, UniRef:UniRef50_G4NST2, UniRef:UniRef90_G4NST2
RefSeq:WP_010327933.1, SO:0001217, UniParc:UPI000195C68E, UniRef:UniRef100_C0H444, UniRef:UniRef50_C0H444, UniRef:UniRef90_C0H444
RefSeq:WP_268455959.1, SO:0001217, UniParc:UPI00227E9B84, UniRef:UniRef100_UPI00227E9B84, UniRef:UniRef50_P50840, UniRef:UniRef90_I2C6W8
RFAM:RF00011, SO:0000386
COG:COG3599, COG:D, GO:0005737, GO:0008360, GO:0051301, SO:0001217, UniRef:UniRef50_P0CI74, UniRef:UniRef90_P0CI74
RefSeq:WP_003225627.1, SO:0001217, UniParc:UPI0001D28AD4, UniRef:UniRef100_A0A9Q4E2G3, UniRef:UniRef50_P50838, UniRef:UniRef90_P50838
KEGG:K07502, RefSeq:WP_072566465.1, SO:0001217, UniParc:UPI00086F9056, UniRef:UniRef100_UPI00086F9056, UniRef:UniRef50_P50837, UniRef:UniRef90_P50837
BlastRules:WP_003246149, EC:3.6.4.-, GO:0003676, GO:0005524, GO:0006289, GO:0016787, GO:0036297, GO:0043138, KEGG:K06877, SO:0001217, UniRef:UniRef50_P50830, UniRef:UniRef90_P50830
GO:0005737, GO:0009401, GO:0016301, SO:0001217, UniRef:UniRef50_P50829, UniRef:UniRef90_P50829
RefSeq:WP_072182236.1, SO:0001217, UniParc:UPI0006A8D1DD, UniRef:UniRef100_UPI0006A8D1DD, UniRef:UniRef50_P50836, UniRef:UniRef90_UPI0002DB1045
RefSeq:WP_014114177.1, SO:0001217, UniParc:UPI00022BBA68, UniRef:UniRef100_G4NSU1, UniRef:UniRef50_P50835, UniRef:UniRef90_P50835
RefSeq:WP_003225611.1, SO:0001217, UniParc:UPI0001D28ACC, UniRef:UniRef100_A0A9Q4DQ71, UniRef:UniRef50_P50834, UniRef:UniRef90_P50834
RefSeq:WP_044454164.1, SO:0001217, UniParc:UPI0005CB0DE8, UniRef:UniRef100_UPI0005CB0DE8, UniRef:UniRef50_P50833, UniRef:UniRef90_P50833
RefSeq:WP_014114178.1, SO:0001217, UniParc:UPI00022BBC00, UniRef:UniRef100_G4NSU4, UniRef:UniRef50_P50832, UniRef:UniRef90_P50832
BlastRules:WP_003225606, GO:0030435, GO:0034301, KEGG:K06430, RefSeq:WP_003225606.1, SO:0001217, UniParc:UPI00001B093E, UniRef:UniRef100_Q7WY65, UniRef:UniRef50_Q7WY65, UniRef:UniRef90_Q7WY65
SO:0001217, UniRef:UniRef50_A0A9Q4DPJ1, UniRef:UniRef90_A0A9Q4DPJ1
SO:0001217, UniRef:UniRef50_UPI000B224C37
EC:3.1.21.10, GO:0000287, GO:0003676, GO:0003677, GO:0004518, GO:0004519, GO:0005737, GO:0006281, GO:0006310, GO:0007059, KEGG:K03700, RefSeq:WP_014114180.1, SO:0001217, UniParc:UPI00022BBC02,
GO:0005886, GO:0006508, GO:0008360, GO:0008658, GO:0008955, GO:0009002, GO:0009252, GO:0030288, GO:0046677, GO:0071555, SO:0001217, UniRef:UniRef50_P39793, UniRef:UniRef90_P39793
RefSeq:WP_014114182.1, SO:0001217, UniParc:UPI00022BBC04, UniRef:UniRef100_G4NSU9, UniRef:UniRef50_P39789, UniRef:UniRef90_P39789
EC:4.2.99.18, GO:0003677, GO:0006281, GO:0006285, GO:0019104, GO:0046872, GO:0051539, GO:0140078, RefSeq:WP_003225594.1, SO:0001217, UniParc:UPI0001D28AC5, UniRef:UniRef100_A0A2M8T2K9, UniRef:Ui
BlastRules:WP_000728550, BlastRules:WP_004398499, GO:0006269, GO:1990077, RefSeq:WP_072566472.1, SO:0001217, UniParc:UPI00090AC327, UniRef:UniRef100_UPI00090AC327, UniRef:UniRef50_P39787, UniRef:
EC:6.1.1.22, GO:0003676, GO:0004816, GO:0005524, GO:0005737, GO:0006421, GO:0016740, SO:0001217, UniRef:UniRef50_P39772, UniRef:UniRef90_P39772
SO:0001217, UniRef:UniRef50_UPI0022816E30, UniRef:UniRef90_UPI0022816E30
EC:2.6.1.1, GO:0004069, GO:0005737, GO:0006520, GO:0009058, GO:0030170, SO:0001217, UniRef:UniRef50_P23034, UniRef:UniRef90_P53001
GO:0016020, SO:0001217, UniRef:UniRef50_P54396, UniRef:UniRef90_P54396
RefSeq:WP_003225588.1, SO:0001217, UniParc:UPI0000060633, UniRef:UniRef100_P54395, UniRef:UniRef50_P54395, UniRef:UniRef90_P54395
EC:3.1.-.-, EC:3.6.4.12, GO:0003677, GO:0003678, GO:0003887, GO:0005524, GO:0005737, GO:0006260, GO:0006974, GO:0008408, GO:0016818, KEGG:K03722, SO:0001217, UniRef:UniRef50_P54394, UniRef:UniRef90_
EC:4.1.1.11, GO:0004068, GO:0005829, GO:0006523, GO:0015940, KEGG:K01579, RefSeq:WP_003225586.1, SO:0001217, UniParc:UPI0000038DEA, UniRef:UniRef100_P52999, UniRef:UniRef50_P52999, UniRef:UniRef90
EC:6.3.2.1, GO:0004592, GO:0015940, KEGG:K01918, SO:0001217, UniRef:UniRef50_B9DKF5, UniRef:UniRef90_A0A6H0WKE4
EC:2.1.2.11, GO:0000287, GO:0003864, GO:0005737, GO:0015940, KEGG:K00606, SO:0001217, UniRef:UniRef50_Q8Y601, UniRef:UniRef90_P52996
RFAM:RF01749, SO:0005836
EC:6.3.4.15, KEGG:K03524, SO:0001217, UniRef:UniRef50_W4RH87, UniRef:UniRef90_UPI00227DCF29
PFAM:PF01743.26, PFAM:PF08279.18, PFAM:PF12627.12, PFAM:PF13735.12
EC:2.4.1.-, GO:0000166, GO:0016757, GO:0071793, SO:0001217, UniRef:UniRef50_P42982, UniRef:UniRef90_P42982
EC:3.5.1.-, GO:0016811, GO:0019213, GO:0046872, GO:0071793, SO:0001217, UniRef:UniRef50_P42981, UniRef:UniRef90_P42981
SO:0001217, UniRef:UniRef50_Q9XCV1
EC:1.17.1.8, GO:0005829, GO:0008839, GO:0009089, GO:0016726, GO:0019877, GO:0050661, GO:0051287, KEGG:K00215, SO:0001217, UniRef:UniRef50_Q8EQD3, UniRef:UniRef90_P42976
RefSeq:WP_003225569.1, SO:0001217, UniParc:UPI0001D28A16, UniRef:UniRef100_A0A9Q4E2R4, UniRef:UniRef50_A4IQ68, UniRef:UniRef90_I2C700
GO:0005886, RefSeq:WP_003230635.1, SO:0001217, UniParc:UPI000195C6A8, UniRef:UniRef100_P42978, UniRef:UniRef50_P42978, UniRef:UniRef90_P42978
GO:0016020, GO:0030436, RefSeq:WP_003225565.1, SO:0001217, UniParc:UPI0001D28A14, UniRef:UniRef100_A0A9Q4E5E4, UniRef:UniRef50_P54393, UniRef:UniRef90_P54393
GO:0005886, SO:0001217, UniRef:UniRef50_P54392, UniRef:UniRef90_P54392
SO:0001217, UniRef:UniRef50_A0A941FNX6
GO:0005506, GO:0005886, GO:0009055, GO:0016491, GO:0020037, SO:0001217, UniRef:UniRef50_P46913, UniRef:UniRef90_P46913

SO:0001217, UniRef:UniRef50_S7SVE1, UniRef:UniRef90_S7SVE1
SO:0001217, UniRef:UniRef50_S7SVP5
GO:0005886, GO:0016491, GO:0046872, GO:0051537, RefSeq:WP_003225556.1, SO:0001217, UniParc:UPI0000F1CC3E, UniRef:UniRef100_A3F3A1, UniRef:UniRef50_P46911, UniRef:UniRef90_P46911
RefSeq:WP_072182226.1, SO:0001217, UniParc:UPI0006A91610, UniRef:UniRef100_A0A9Q4HFS5, UniRef:UniRef50_P54391, UniRef:UniRef90_P54391
GO:0005515, RefSeq:WP_003225551.1, SO:0001217, UniParc:UPI0001D28A11, UniRef:UniRef100_A0A9Q4HGN5, UniRef:UniRef50_P54390, UniRef:UniRef90_P54390
SO:0001217, UniRef:UniRef50_UPI0022827496, UniRef:UniRef90_UPI0022827496
SO:0001217, UniRef:UniRef50_UPI00228197F2, UniRef:UniRef90_UPI00227E5794
SO:0001217, UniRef:UniRef50_R0NUC6
EC:1.3.1.12, GO:0004665, GO:0006571, GO:0008977, GO:0070403, RefSeq:WP_072182223.1, SO:0001217, UniParc:UPI0006A808A2, UniRef:UniRef100_UPI0006A808A2, UniRef:UniRef50_P20692, UniRef:UniRef90_P2069
SO:0001217, UniRef:UniRef50_A0A5S9MFF3, UniRef:UniRef90_UPI0022821367
SO:0001217, UniRef:UniRef50_UPI00228C2123
EC:4.2.1.20, GO:0000162, GO:0004834, GO:0005829, GO:0006568, KEGG:K01695, RefSeq:WP_072182221.1, SO:0001217, UniParc:UPI0006A860C9, UniRef:UniRef100_UPI0006A860C9, UniRef:UniRef50_P07601, UniRef:
EC:4.2.1.20, GO:0000162, GO:0004834, GO:0005737, GO:0006568, RefSeq:WP_072182220.1, SO:0001217, UniParc:UPI0006A824D5, UniRef:UniRef100_A0A9Q4E2D3, UniRef:UniRef50_P07600, UniRef:UniRef90_P07600
PFAM:PF00218.27, PFAM:PF00697.28
EC:2.4.2.18, GO:0000162, GO:0000287, GO:0004048, GO:0005829, KEGG:K00766, RefSeq:WP_072182217.1, SO:0001217, UniParc:UPI0006A8292B, UniRef:UniRef100_UPI0006A8292B, UniRef:UniRef50_P03947, UniRef:
EC:4.1.3.27, GO:0000162, GO:0004049, GO:0046872, KEGG:K01657, SO:0001217, UniRef:UniRef50_P03963, UniRef:UniRef90_P03963
RFAM:RF02370, SO:0000140
EC:5.4.99.5, GO:0004106, GO:0005737, GO:0008652, GO:0009073, GO:0046417, KEGG:K06208, RefSeq:WP_014114208.1, SO:0001217, UniParc:UPI00022BA443, UniRef:UniRef100_G4NSY5, UniRef:UniRef50_P19080, U
EC:4.2.3.4, GO:0000166, GO:0003856, GO:0005737, GO:0008652, GO:0009073, GO:0009423, GO:0046872, RefSeq:WP_088679253.1, SO:0001217, UniParc:UPI000B52FDE6, UniRef:UniRef100_UPI000B52FDE6, UniRef:U
COG:COG0082, COG:E, EC:4.2.3.5, GO:0004107, GO:0005829, GO:0008652, GO:0009073, GO:0009423, GO:0010181, SO:0001217, UniRef:UniRef50_P31104, UniRef:UniRef90_P31104
BlastRules:WP_000582898, BlastRules:WP_003230589, EC:2.1.1.80, GO:0006935, GO:0008983, GO:0032259, RefSeq:WP_072566487.1, SO:0001217, UniParc:UPI00086C4ED1, UniRef:UniRef100_UPI00086C4ED1, UniRef
EC:2.7.4.6, GO:0004550, GO:0005524, GO:0005737, GO:0006183, GO:0006228, GO:0006241, GO:0046872, SO:0001217, UniParc:UPI00022BA447, UniRef:UniRef100_G4NSZ0, UniRef:UniRef50_A6N0M9, UniRef:UniRef90
EC:2.5.1.30, GO:0000010, GO:0004659, GO:0008299, GO:0009234, GO:0016765, GO:0030435, GO:0046872, KEGG:K24873, SO:0001217, UniRef:UniRef50_P31114, UniRef:UniRef90_P31114
EC:2.1.1.163, GO:0008168, GO:0009234, GO:0032259, GO:0043770, RefSeq:WP_088679254.1, SO:0001217, UniParc:UPI00086AAA45, UniRef:UniRef100_UPI00086AAA45, UniRef:UniRef50_P31113, UniRef:UniRef90_P31
BlastRules:WP_004398550, EC:2.5.1.30, GO:0000010, GO:0008299, GO:0009234, GO:0016765, GO:0030435, KEGG:K00805, SO:0001217, UniRef:UniRef50_P31112, UniRef:UniRef90_P31112
GO:0003723, GO:0006353, GO:0006355, KEGG:K06285, RefSeq:WP_003225517.1, SO:0001217, UniParc:UPI0000F1CAAE, UniRef:UniRef100_A3F3H8, UniRef:UniRef50_P19466, UniRef:UniRef90_P19466
EC:3.5.4.16, GO:0003934, GO:0005525, GO:0005737, GO:0006729, GO:0006730, GO:0008270, GO:0046654, KEGG:K01495, RefSeq:WP_088679255.1, SO:0001217, UniParc:UPI00086E5348, UniRef:UniRef100_UPI00086E
BlastRules:WP_003153447, COG:B, COG:COG0776, GO:0003677, GO:0005829, GO:0009295, GO:0030261, GO:0030527, RefSeq:WP_003153447.1, SO:0001217, UniParc:UPI0000060656, UniRef:UniRef100_P08821, UniR
COG:COG5831, COG:D, EC:3.6.1.-, GO:0000270, GO:0003689, GO:0005524, GO:0005737, GO:0009847, GO:0016887, GO:0030435, GO:0030436, GO:0031160, GO:0042244, GO:0042601, GO:0043595, GO:0043934, GO:
RefSeq:WP_061187630.1, SO:0001217, UniParc:UPI0006A900EE, UniRef:UniRef100_A0A9Q4E1G9, UniRef:UniRef50_P39911, UniRef:UniRef90_P39911
GO:0005886, RefSeq:WP_003225508.1, SO:0001217, UniParc:UPI0001D28958, UniRef:UniRef100_A0A9Q4DQP0, UniRef:UniRef50_P50744, UniRef:UniRef90_P50744
EC:1.1.1.94, GO:0005829, GO:0005975, GO:0006072, GO:0006650, GO:0008654, GO:0009331, GO:0046167, GO:0046168, GO:0047952, GO:0051287, SO:0001217, UniRef:UniRef50_P46919, UniRef:UniRef90_P46919
BlastRules:WP_001125893, BlastRules:WP_003230565, COG:COG1160, COG:J, EC:3.6.5.-, GO:0003924, GO:0005525, GO:0005737, GO:0042254, GO:0043022, KEGG:K03977, RefSeq:WP_014114217.1, SO:0001217, Uni
RefSeq:WP_014114218.1, SO:0001217, UniParc:UPI00022BA44D, UniRef:UniRef100_G4NVY1, UniRef:UniRef50_C0H445, UniRef:UniRef90_C0H445
GO:0005886, KEGG:K05739, RefSeq:WP_014114219.1, SO:0001217, UniParc:UPI00022BA44E, UniRef:UniRef100_G4NVY2, UniRef:UniRef50_P50742, UniRef:UniRef90_P50742
GO:0005886, RefSeq:WP_072182211.1, SO:0001217, UniParc:UPI0006A84212, UniRef:UniRef100_UPI0006A84212, UniRef:UniRef50_P50741, UniRef:UniRef90_P50741
RefSeq:WP_003225499.1, SO:0001217, UniParc:UPI000197617F, UniRef:UniRef100_C0H446, UniRef:UniRef50_C0H446, UniRef:UniRef90_C0H446
EC:5.3.3.2, GO:0000287, GO:0004452, GO:0005737, GO:0008299, GO:0010181, GO:0016491, GO:0070402, RefSeq:WP_072182239.1, SO:0001217, UniParc:UPI0006A886D3, UniRef:UniRef100_UPI0006A886D3, UniRef:Ur
GO:0003676, GO:0003729, GO:0003735, GO:0006412, GO:0022627, RefSeq:WP_003225495.1, SO:0001217, UniParc:UPI0001D28952, UniRef:UniRef100_A0A9Q4E401, UniRef:UniRef50_P38494, UniRef:UniRef90_P38494
EC:2.7.4.25, GO:0004127, GO:0005524, GO:0005829, GO:0006220, GO:0015949, GO:0036430, GO:0036431, RefSeq:WP_072182210.1, SO:0001217, UniParc:UPI0006A8D5C7, UniRef:UniRef100_UPI0006A8D5C7, UniRef:
SO:0001217, UniRef:UniRef50_P38492
RefSeq:WP_072182208.1, SO:0001217, UniParc:UPI0006A9260D, UniRef:UniRef100_UPI0006A9260D, UniRef:UniRef50_P38491, UniRef:UniRef90_A0A2M8T611
GO:0009847, GO:0030435, GO:0030436, KEGG:K06313, RefSeq:WP_014114226.1, SO:0001217, UniParc:UPI00022BA455, UniRef:UniRef100_G4NVZ0, UniRef:UniRef50_P38490, UniRef:UniRef90_P38490
EC:4.2.2.29, GO:0003824, GO:0009847, GO:0016787, GO:0030435, GO:0042763, GO:0071555, KEGG:K27245, RefSeq:WP_072182207.1, SO:0001217, UniParc:UPI0006A81D95, UniRef:UniRef100_UPI0006A81D95, UniRef
EC:3.4.-.-, GO:0005886, GO:0006508, GO:0007165, GO:0008233, GO:0070007, RefSeq:WP_014114228.1, SO:0001217, UniParc:UPI00022BA457, UniRef:UniRef100_G4NVZ2, UniRef:UniRef50_P50738, UniRef:UniRef90_P5
EC:1.8.1.-, GO:0004497, GO:0050660, KEGG:K26960, RefSeq:WP_072182206.1, SO:0001217, UniParc:UPI0006A803CF, UniRef:UniRef100_UPI0006A803CF, UniRef:UniRef50_P50736, UniRef:UniRef90_P50736
SO:0001217, UniRef:UniRef50_P39633
SO:0001217, UniRef:UniRef50_A0A0B0D0I0
KEGG:K16511, RefSeq:WP_072182204.1, SO:0001217, UniParc:UPI0006A81C29, UniRef:UniRef100_UPI0006A81C29, UniRef:UniRef50_P50734, UniRef:UniRef90_G4NVZ5
GO:0005524, GO:0008758, GO:0009245, GO:0016020, KEGG:K07098, RefSeq:WP_014114232.1, SO:0001217, UniParc:UPI00022BA45B, UniRef:UniRef100_G4NVZ6, UniRef:UniRef50_P50733, UniRef:UniRef90_P50733
SO:0001217, UniRef:UniRef50_P50732, UniRef:UniRef90_G4NVZ7
RefSeq:WP_088679258.1, SO:0001217, UniParc:UPI0006A8B27E, UniRef:UniRef100_UPI0006A8B27E, UniRef:UniRef50_P50731, UniRef:UniRef90_G4NVZ8
GO:0004222, GO:0005886, GO:0071586, RefSeq:WP_014114235.1, SO:0001217, UniParc:UPI00022BA45E, UniRef:UniRef100_G4NVZ9, UniRef:UniRef50_P50730, UniRef:UniRef90_P50730
EC:5.6.2.4, GO:0003677, GO:0003689, GO:0005524, GO:0005694, GO:0005737, GO:0006281, GO:0006310, GO:0009378, GO:0016853, GO:0016887, GO:0030894, GO:0032508, GO:0036121, GO:0043138, GO:0043590, G
GO:0005737, GO:0009295, SO:0001217, UniRef:UniRef50_P50728, UniRef:UniRef90_P50728
GO:0005506, GO:0009055, GO:0051539, RefSeq:WP_003225461.1, SO:0001217, UniParc:UPI000006066F, UniRef:UniRef100_P50727, UniRef:UniRef50_P50727, UniRef:UniRef90_P50727
BlastRules:WP_004399159, KEGG:K23675, RefSeq:WP_014114238.1, SO:0001217, UniParc:UPI00022BA461, UniRef:UniRef100_G4NW03, UniRef:UniRef50_P50726, UniRef:UniRef90_A0A9Q2LK89
SO:0001217, UniRef:UniRef50_E0U1X9, UniRef:UniRef90_E0U1X9
RFAM:RF00050, SO:0000035
SO:0001217, UniRef:UniRef50_A0A1I0TYC1
SO:0001217, UniRef:UniRef50_P35146, UniRef:UniRef90_E0U1Y1
SO:0001217, UniRef:UniRef50_P35166, UniRef:UniRef90_UPI002DBE3A96
GO:0003677, GO:0005886, GO:0006352, GO:0006355, GO:0016987, RefSeq:WP_003223881.1, SO:0001217, UniParc:UPI0000F1CBF0, UniRef:UniRef100_A0A9Q4DSF0, UniRef:UniRef50_P35165, UniRef:UniRef90_P35165
EC:2.7.13.3, KEGG:K07651, SO:0001217, UniRef:UniRef50_A0A1R0YNL3, UniRef:UniRef90_UPI00209F0451
PFAM:PF18698.7
BlastRules:WP_000426862, BlastRules:WP_003246107, COG:COG0745, COG:TK, GO:0000156, GO:0000976, GO:0005829, GO:0006355, GO:0032993, RefSeq:WP_080376113.1, SO:0001217, UniParc:UPI0006A8E9D9, Ur
SO:0001217, UniRef:UniRef50_UPI002B4B2232
BlastRules:WP_000910533, BlastRules:WP_003230515, GO:0005886, GO:0017004, KEGG:K07399, RefSeq:WP_014114246.1, SO:0001217, UniParc:UPI00022BA5CA, UniRef:UniRef100_G4NW12, UniRef:UniRef50_P35161
BlastRules:WP_000742206, BlastRules:WP_003230513, GO:0005886, GO:0015036, GO:0016209, GO:0017004, RefSeq:WP_014114247.1, SO:0001217, UniParc:UPI00022BA5CB, UniRef:UniRef100_G4NW13, UniRef:UniRe
SO:0001217, UniRef:UniRef50_A0A380FGT7
BlastRules:WP_000029511, BlastRules:WP_003230509, COG:COG0700, COG:S, GO:0005886, GO:0030435, KEGG:K06374, RefSeq:WP_003223896.1, SO:0001217, UniParc:UPI0001D2875A, UniRef:UniRef100_A0A9Q4HC
BlastRules:WP_000247805, BlastRules:WP_004398980, GO:0005886, GO:0030435, KEGG:K06373, RefSeq:WP_014114249.1, SO:0001217, UniParc:UPI00022BA5CC, UniRef:UniRef100_G4NW16, UniRef:UniRef50_P35157
BlastRules:WP_003230505, EC:3.4.16.4, GO:0006508, GO:0008360, GO:0009002, GO:0009252, GO:0016020, GO:0030435, GO:0071555, KEGG:K07258, RefSeq:WP_072182917.1, SO:0001217, UniParc:UPI0006A8EBEE, U
RefSeq:WP_014114251.1, SO:0001217, UniParc:UPI00022BA5CE, UniRef:UniRef100_G4NW18, UniRef:UniRef50_P35156, UniRef:UniRef90_P35156
GO:0005515, GO:0005737, GO:0006260, GO:0007059, GO:0051301, GO:0051304, KEGG:K06024, SO:0001217, UniRef:UniRef50_P35155, UniRef:UniRef90_P35155
BlastRules:WP_001199756, BlastRules:WP_003246108, GO:0005737, GO:0006260, GO:0007059, GO:0042802, GO:0051301, SO:0001217, UniRef:UniRef50_P35154, UniRef:UniRef90_P35154
KEGG:K09763, RefSeq:WP_072183495.1, SO:0001217, UniParc:UPI0006A927BF, UniRef:UniRef100_UPI0006A927BF, UniRef:UniRef50_P17617, UniRef:UniRef90_E0U1Z6
BlastRules:WP_000908398, BlastRules:WP_003223910, EC:2.3.1.-, GO:0009231, GO:0016747, KEGG:K02859, RefSeq:WP_003223910.1, SO:0001217, UniParc:UPI0000060683, UniRef:UniRef100_P17622, UniRef:UniRef50
BlastRules:WP_000230891, BlastRules:WP_003223915, EC:2.5.1.78, GO:0000906, GO:0005829, GO:0009231, GO:0009349, KEGG:K00794, RefSeq:WP_003223915.1, SO:0001217, UniParc:UPI00000013A5, UniRef:UniRef1
EC:3.5.4.25, EC:4.1.99.12, GO:0000287, GO:0003935, GO:0005525, GO:0005829, GO:0008270, GO:0008686, GO:0009231, GO:0030145, KEGG:K14652, RefSeq:WP_014114254.1, SO:0001217, UniParc:UPI00022BA5D1, U
BlastRules:WP_000493929, BlastRules:WP_004398505, EC:2.5.1.9, GO:0004746, GO:0009231, KEGG:K00793, RefSeq:WP_072183496.1, SO:0001217, UniParc:UPI0006A9287C, UniRef:UniRef100_UPI0006A9287C, UniRef
EC:1.1.1.193, EC:3.5.4.26, GO:0008270, GO:0008703, GO:0008835, GO:0009231, GO:0050661, RefSeq:WP_072183497.1, SO:0001217, UniParc:UPI0006A8FFD7, UniRef:UniRef100_UPI0006A8FFD7, UniRef:UniRef50_P17
RFAM:RF00050, SO:0000035
RefSeq:WP_003223922.1, SO:0001217, UniParc:UPI0001D28763, UniRef:UniRef100_G4NW27, UniRef:UniRef50_P17616, UniRef:UniRef90_P17616
EC:3.4.21.89, GO:0004252, GO:0005886, GO:0006465, GO:0009306, GO:0016020, KEGG:K03100, RefSeq:WP_072183498.1, SO:0001217, UniParc:UPI0006A8AB51, UniRef:UniRef100_UPI0006A8AB51, UniRef:UniRef50_F
SO:0001217, UniRef:UniRef50_UPI000A08E565
RefSeq:WP_003223929.1, SO:0001217, UniParc:UPI0000168481, UniRef:UniRef100_C0H447, UniRef:UniRef50_C0H447, UniRef:UniRef90_C0H447
RefSeq:WP_072183501.1, SO:0001217, UniParc:UPI0006A82935, UniRef:UniRef100_UPI0006A82935, UniRef:UniRef50_O32013, UniRef:UniRef90_G4NW33
EC:5.2.1.8, GO:0003755, GO:0005737, GO:0006457, GO:0140839, GO:0140840, RefSeq:WP_003223931.1, SO:0001217, UniParc:UPI000006068E, UniRef:UniRef100_P35137, UniRef:UniRef50_P35137, UniRef:UniRef90_P3
GO:0005886, GO:0045121, RefSeq:WP_014114262.1, SO:0001217, UniParc:UPI00022BA5D9, UniRef:UniRef100_G4NW35, UniRef:UniRef50_P31847, UniRef:UniRef90_P31847
EC:4.1.1.20, GO:0008836, GO:0009089, GO:0030170, KEGG:K01586, SO:0001217, UniRef:UniRef50_P23630, UniRef:UniRef90_P23630
GO:0005886, GO:0009847, GO:0030435, KEGG:K06408, RefSeq:WP_041520989.1, SO:0001217, UniParc:UPI00059F85D3, UniRef:UniRef100_UPI00059F85D3, UniRef:UniRef50_P31845, UniRef:UniRef90_P31845
BlastRules:WP_003230469, GO:0030435, RefSeq:WP_072183502.1, SO:0001217, UniParc:UPI0006A8E00E, UniRef:UniRef100_UPI0006A8E00E, UniRef:UniRef50_P40870, UniRef:UniRef90_P40870
GO:0030435, GO:0030436, KEGG:K06407, RefSeq:WP_003236718.1, SO:0001217, UniParc:UPI0002ACE2B3, UniRef:UniRef100_A0A2M8T258, UniRef:UniRef50_C0H450, UniRef:UniRef90_C0H450
GO:0005886, GO:0016746, GO:0030435, GO:0030436, GO:0031160, KEGG:K06406, RefSeq:WP_014114264.1, SO:0001217, UniParc:UPI00022BA5DB, UniRef:UniRef100_G4NW37, UniRef:UniRef50_P40869, UniRef:UniRe
GO:0005886, GO:0030435, GO:0030436, KEGG:K06405, RefSeq:WP_014114265.1, SO:0001217, UniParc:UPI00022BA5DC, UniRef:UniRef100_G4NW38, UniRef:UniRef50_P40868, UniRef:UniRef90_P40868
BlastRules:WP_000572931, BlastRules:WP_003230463, GO:0005886, GO:0030435, KEGG:K06404, RefSeq:WP_088679260.1, SO:0001217, UniParc:UPI000B53724F, UniRef:UniRef100_UPI000B53724F, UniRef:UniRef50_P
SO:0001217, UniRef:UniRef50_P40866
GO:0003677, GO:0003700, GO:0006352, GO:0006355, GO:0016987, GO:0030435, KEGG:K03091, RefSeq:WP_014114268.1, SO:0001217, UniParc:UPI00022BA5DF, UniRef:UniRef100_G4NW41, UniRef:UniRef50_P07860,
BlastRules:WP_001243400, BlastRules:WP_003230452, EC:2.7.11.1, GO:0004672, GO:0004676, GO:0004677, GO:0004679, GO:0004711, GO:0005524, GO:0006468, GO:0016989, GO:0030435, GO:0030436, GO:0035175,
GO:0006355, GO:0030435, GO:0043856, GO:0045152, RefSeq:WP_003223957.1, SO:0001217, UniParc:UPI0001D28833, UniRef:UniRef100_A0A9Q4DRE1, UniRef:UniRef50_P10727, UniRef:UniRef90_P10727
BlastRules:WP_004398637, EC:3.4.16.4, GO:0005576, GO:0006508, GO:0008360, GO:0009002, GO:0009252, GO:0071555, KEGG:K07258, RefSeq:WP_041520991.1, SO:0001217, UniParc:UPI0003181D2D, UniRef:UniRef1
EC:2.4.2.1, GO:0004731, GO:0005737, GO:0006139, GO:0009116, KEGG:K03783, RefSeq:WP_014114270.1, SO:0001217, UniParc:UPI00022BA5E1, UniRef:UniRef100_G4NW45, UniRef:UniRef50_P46354, UniRef:UniRef90
EC:5.4.2.7, GO:0000287, GO:0005737, GO:0005829, GO:0006015, GO:0006018, GO:0008973, GO:0009117, GO:0030145, GO:0043094, KEGG:K01839, RefSeq:WP_061187609.1, SO:0001217, UniParc:UPI00077264B9, Un
GO:0003677, GO:0005737, GO:0006310, GO:0006313, GO:0007059, GO:0009009, GO:0009037, GO:0051301, RefSeq:WP_003236743.1, SO:0001217, UniParc:UPI00022BA5E3, UniRef:UniRef100_A0A2M8T247, UniRef:Un
GO:0005886, SO:0001217, UniRef:UniRef50_C0H451, UniRef:UniRef90_C0H451
BlastRules:WP_000824996, BlastRules:WP_003223972, GO:0000976, GO:0001217, GO:0003700, GO:0005737, GO:0008270, GO:0032993, GO:0045892, GO:1900376, RefSeq:WP_003223972.1, SO:0001217, UniParc:UPI0
GO:0005886, GO:0030428, GO:0030435, GO:0030436, GO:0042173, GO:1901893, RefSeq:WP_014114272.1, SO:0001217, UniParc:UPI00022BA5E4, UniRef:UniRef100_G4NW50, UniRef:UniRef50_P37873, UniRef:UniRef90
SO:0001217, UniRef:UniRef50_P54573, UniRef:UniRef90_E0U227
EC:1.1.1.38, GO:0004471, GO:0008948, GO:0046872, GO:0051287, RefSeq:WP_072183677.1, SO:0001217, UniParc:UPI0006A911AC, UniRef:UniRef100_UPI0006A911AC, UniRef:UniRef50_P54572, UniRef:UniRef90_P545
GO:0005886, GO:0006814, GO:0015297, GO:0015385, GO:0016020, GO:1902600, KEGG:K03315, RefSeq:WP_061187604.1, SO:0001217, UniParc:UPI0006A8344B, UniRef:UniRef100_A0A9Q4E4B3, UniRef:UniRef50_P545
SO:0001217, UniRef:UniRef50_A0A351TMQ7
PFAM:PF00206.25
BlastRules:WP_000722287, BlastRules:WP_004399032, EC:3.5.1.1, GO:0004067, GO:0005737, GO:0006520, RefSeq:WP_003223983.1, SO:0001217, UniParc:UPI0001D2883F, UniRef:UniRef100_A0A9Q4E8F8, UniRef:UniR
BlastRules:WP_000893556, BlastRules:WP_004399024, GO:0003677, RefSeq:WP_014114278.1, SO:0001217, UniParc:UPI00022BA755, UniRef:UniRef100_G4NX20, UniRef:UniRef50_Q07683, UniRef:UniRef90_Q07683
RefSeq:WP_061187603.1, SO:0001217, UniParc:UPI0006A885D2, UniRef:UniRef100_UPI0006A885D2, UniRef:UniRef50_P38423, UniRef:UniRef90_P38423
BlastRules:WP_004398600, EC:3.6.1.13, GO:0005829, GO:0006753, GO:0019693, GO:0046872, GO:0047631, RefSeq:WP_088679261.1, SO:0001217, UniParc:UPI000B52E4CA, UniRef:UniRef100_UPI000B52E4CA, UniRef
BlastRules:WP_003230420, RefSeq:WP_014114281.1, SO:0001217, UniParc:UPI00022BA758, UniRef:UniRef100_G4NX23, UniRef:UniRef50_K6E8M3, UniRef:UniRef90_G4NX23
EC:1.-.-.-, GO:0016491, RefSeq:WP_072182158.1, SO:0001217, UniParc:UPI0006A90F81, UniRef:UniRef100_UPI0006A90F81, UniRef:UniRef50_P54569, UniRef:UniRef90_P54569
SO:0001217, UniRef:UniRef50_G4NX25, UniRef:UniRef90_G4NX25
SO:0001217, UniRef:UniRef50_P54567, UniRef:UniRef90_E0U2N2
GO:0009252, GO:0016020, GO:0051301, KEGG:K03588, RefSeq:WP_072182160.1, SO:0001217, UniParc:UPI0006A8F927, UniRef:UniRef100_UPI0006A8F927, UniRef:UniRef50_A0A024P806, UniRef:UniRef90_A0A9Q4HH
COG:COG0772, COG:D, GO:0008360, GO:0016020, KEGG:K05837, RefSeq:WP_072182161.1, SO:0001217, UniParc:UPI0006A7F41A, UniRef:UniRef100_UPI0006A7F41A, UniRef:UniRef50_F5L504, UniRef:UniRef90_A0AA
RefSeq:WP_061187596.1, SO:0001217, UniParc:UPI0006A87D71, UniRef:UniRef100_A0A9Q4HLZ8, UniRef:UniRef50_J8Z144, UniRef:UniRef90_G4NX29
RefSeq:WP_003226554.1, SO:0001217, UniParc:UPI0001D285AC, UniRef:UniRef100_G4NX30, UniRef:UniRef50_P54566, UniRef:UniRef90_P54566
RefSeq:WP_072182162.1, SO:0001217, UniParc:UPI0006A8600E, UniRef:UniRef100_UPI0006A8600E, UniRef:UniRef50_P54565, UniRef:UniRef90_P54565
SO:0001217, UniRef:UniRef50_P54564, UniRef:UniRef90_E0U2N8
SO:0001217, UniRef:UniRef50_A0A5E7U243, UniRef:UniRef90_G4NX33
GO:0016747, RefSeq:WP_014114290.1, SO:0001217, UniParc:UPI00022BA761, UniRef:UniRef100_G4NX34, UniRef:UniRef50_P54562, UniRef:UniRef90_P54562
RefSeq:WP_014114291.1, SO:0001217, UniParc:UPI00022BA762, UniRef:UniRef100_G4NX35, UniRef:UniRef50_P54561, UniRef:UniRef90_P54561
EC:2.7.7.7, GO:0000287, GO:0003684, GO:0003887, GO:0005737, GO:0006261, GO:0009432, GO:0042276, SO:0001217, UniRef:UniRef50_P54560, UniRef:UniRef90_P54560
RefSeq:WP_061187593.1, SO:0001217, UniParc:UPI0006A91917, UniRef:UniRef100_A0A9Q4E493, UniRef:UniRef50_O32014, UniRef:UniRef90_O32014
SO:0001217, UniRef:UniRef50_G4NX39, UniRef:UniRef90_G4NX39
SO:0001217, UniRef:UniRef50_G4NX40, UniRef:UniRef90_G4NX40
GO:0005886, GO:0022857, SO:0001217, UniRef:UniRef50_P54559, UniRef:UniRef90_P54559

RefSeq:WP_165766225.1, SO:0001217, UniParc:UPI0006A8729D, UniRef:UniRef100_A0A9Q4E3B2, UniRef:UniRef50_G4NX42, UniRef:UniRef90_A0A2M8T603
RefSeq:WP_072182168.1, SO:0001217, UniParc:UPI0006A82F33, UniRef:UniRef100_UPI0006A82F33, UniRef:UniRef50_P54557, UniRef:UniRef90_P54557
EC:2.7.1.33, GO:0004594, GO:0005524, GO:0005737, GO:0015937, KEGG:K00867, RefSeq:WP_014114300.1, SO:0001217, UniParc:UPI00022BA76B, UniRef:UniRef100_G4NX44, UniRef:UniRef50_P54556, UniRef:UniRef9
SO:0001217, UniRef:UniRef50_A0A379RXM4
SO:0001217, UniRef:UniRef50_A0A7H4NU56
SO:0001217, UniRef:UniRef50_A0A431WJ11
PFAM:PF00106.31
EC:3.-.-.-, GO:0016787, GO:0046872, RefSeq:WP_072182171.1, SO:0001217, UniParc:UPI0006A8DC51, UniRef:UniRef100_UPI0006A8DC51, UniRef:UniRef50_P54553, UniRef:UniRef90_P54553
RFAM:RF00230, SO:0000140
EC:1.5.1.2, GO:0004735, GO:0005737, GO:0006561, GO:0055129, KEGG:K00286, RefSeq:WP_072182172.1, SO:0001217, UniParc:UPI0006A85455, UniRef:UniRef100_UPI0006A85455, UniRef:UniRef50_P54552, UniRef:Ur
GO:0016787, SO:0001217, UniRef:UniRef50_P54551, UniRef:UniRef90_P54551
EC:1.6.99.1, GO:0003959, GO:0009636, GO:0010181, GO:0050661, RefSeq:WP_072182174.1, SO:0001217, UniParc:UPI0006A8D2F2, UniRef:UniRef100_UPI0006A8D2F2, UniRef:UniRef50_P54550, UniRef:UniRef90_P545
RefSeq:WP_072182175.1, SO:0001217, UniParc:UPI0006A802C0, UniRef:UniRef100_UPI0006A802C0, UniRef:UniRef50_P54549, UniRef:UniRef90_P54549
COG:COG0267, COG:J, GO:0003735, GO:0005737, GO:0005840, GO:0006412, GO:1990904, RefSeq:WP_003325527.1, SO:0001217, UniParc:UPI0001E85586, UniRef:UniRef100_G4NX52, UniRef:UniRef50_Q041X4, UniRe
EC:3.1.26.11, GO:0008270, GO:0016891, GO:0042780, GO:0042781, GO:0042802, KEGG:K00784, RefSeq:WP_014114308.1, SO:0001217, UniParc:UPI00022BA773, UniRef:UniRef100_G4NX53, UniRef:UniRef50_P54548, U
EC:1.1.1.49, GO:0004345, GO:0005829, GO:0006006, GO:0006098, GO:0009051, GO:0016614, GO:0050661, RefSeq:WP_014114310.1, SO:0001217, UniParc:UPI00022BA775, UniRef:UniRef100_G4NX55, UniRef:UniRef50
COG:COG0362, COG:G, EC:1.1.1.44, GO:0004616, GO:0005829, GO:0006098, GO:0009051, GO:0046177, GO:0050661, RefSeq:WP_014114311.1, SO:0001217, UniParc:UPI00022BA776, UniRef:UniRef100_G4NX56, UniR
SO:0001217, UniRef:UniRef50_W4VCZ8
BlastRules:WP_010886563, GO:0005886, SO:0001217, UniParc:UPI00022BA778, UniRef:UniRef100_G4NX58, UniRef:UniRef50_Q7WY64, UniRef:UniRef90_Q7WY64
GO:0005886, GO:0015031, GO:0032977, GO:0051205, KEGG:K03217, RefSeq:WP_072182025.1, SO:0001217, UniParc:UPI0006A83408, UniRef:UniRef100_UPI0006A83408, UniRef:UniRef50_P54544, UniRef:UniRef90_P54
SO:0001217, UniRef:UniRef50_Q5WBJ8
EC:3.4.11.4, GO:0006508, GO:0008237, GO:0046872, KEGG:K01258, RefSeq:WP_014114316.1, SO:0001217, UniParc:UPI00022BA8E9, UniRef:UniRef100_G4NX61, UniRef:UniRef50_P54542, UniRef:UniRef90_P54542
EC:6.4.1.3, GO:0003989, GO:0004658, GO:0005524, GO:0006633, GO:0009317, SO:0001217, UniRef:UniRef50_P54541, UniRef:UniRef90_P54541
EC:5.1.99.1, GO:0004462, GO:0004493, GO:0046491, GO:0046872, RefSeq:WP_014114318.1, SO:0001217, UniParc:UPI00022BA8EB, UniRef:UniRef100_G4NX63, UniRef:UniRef50_P54540, UniRef:UniRef90_P54540
RefSeq:WP_041520994.1, SO:0001217, UniParc:UPI00059EF6D2, UniRef:UniRef100_A0A9Q4HGN3, UniRef:UniRef50_A0A6M3ZDJ6, UniRef:UniRef90_A0A6M3ZDJ6
EC:2.-.-.-, GO:0008360, GO:0016757, GO:0018104, GO:0071555, GO:0071972, RefSeq:WP_014114319.1, SO:0001217, UniParc:UPI00022BA8EC, UniRef:UniRef100_G4NX64, UniRef:UniRef50_P54539, UniRef:UniRef90_P
GO:0005886, RefSeq:WP_003226485.1, SO:0001217, UniParc:UPI0001D284CF, UniRef:UniRef100_G4NX65, UniRef:UniRef50_P54538, UniRef:UniRef90_P54538
BlastRules:WP_004398740, GO:0005524, GO:0005886, GO:0015424, GO:0016887, RefSeq:WP_014114320.1, SO:0001217, UniParc:UPI00022BA8ED, UniRef:UniRef100_G4NX66, UniRef:UniRef50_P54537, UniRef:UniRef9
GO:0005886, GO:0006865, GO:0022857, GO:0043190, SO:0001217, UniRef:UniRef50_P54536, UniRef:UniRef90_P54536
BlastRules:WP_004398706, GO:0005886, GO:0006865, GO:0015276, RefSeq:WP_061186909.1, SO:0001217, UniParc:UPI000772C6E8, UniRef:UniRef100_UPI000772C6E8, UniRef:UniRef50_P54535, UniRef:UniRef90_P54
BlastRules:WP_003226477, GO:0045454, KEGG:K26958, RefSeq:WP_003226477.1, SO:0001217, UniParc:UPI00000606C7, UniRef:UniRef100_P54534, UniRef:UniRef50_P54534, UniRef:UniRef90_P54534
EC:2.7.1.-, GO:0004143, GO:0005524, GO:0006665, GO:0008654, GO:0016020, GO:0046872, RefSeq:WP_014114322.1, SO:0001217, UniParc:UPI00022BA8EF, UniRef:UniRef100_G4NX70, UniRef:UniRef50_P39074, UniRe
BlastRules:WP_118998032, GO:0005886, GO:0022857, GO:0046677, RefSeq:WP_014114323.1, SO:0001217, UniParc:UPI00022BA8F0, UniRef:UniRef100_G4NX71, UniRef:UniRef50_P33449, UniRef:UniRef90_P33449
BlastRules:WP_003230325, GO:0003677, GO:0003700, RefSeq:WP_061186907.1, SO:0001217, UniParc:UPI0006A86DDF, UniRef:UniRef100_UPI0006A86DDF, UniRef:UniRef50_P39075, UniRef:UniRef90_P39075
EC:2.3.1.168, GO:0004149, GO:0005829, GO:0006096, GO:0006099, GO:0043754, RefSeq:WP_072182031.1, SO:0001217, UniParc:UPI0006A87018, UniRef:UniRef100_UPI0006A87018, UniRef:UniRef50_P37942, UniRef:U
BlastRules:WP_000290076, BlastRules:WP_004398638, COG:C, COG:COG0022, EC:1.2.4.4, GO:0003863, GO:0007584, GO:0009083, KEGG:K00167, RefSeq:WP_061186905.1, SO:0001217, UniParc:UPI0006A80A04, UniF
BlastRules:WP_000852555, BlastRules:WP_004398565, EC:1.2.4.4, GO:0003863, GO:0009083, GO:0046872, SO:0001217, UniRef:UniRef50_P37940, UniRef:UniRef90_P37940
SO:0001217, UniRef:UniRef50_A0A838GAL8
SO:0001217, UniRef:UniRef50_V9G6N0
EC:2.7.2.7, GO:0005524, GO:0005737, GO:0006083, GO:0006113, GO:0008776, GO:0016310, GO:0047761, KEGG:K00929, SO:0001217, UniRef:UniRef50_Q05619, UniRef:UniRef90_P54532
BlastRules:WP_003230309, COG:COG0334, COG:E, EC:1.4.1.9, GO:0006552, GO:0050049, RefSeq:WP_072182032.1, SO:0001217, UniParc:UPI0006A88561, UniRef:UniRef100_A0A9Q4HGM7, UniRef:UniRef50_Q60030, U
EC:2.3.1.19, GO:0019605, GO:0050182, RefSeq:WP_014114330.1, SO:0001217, UniParc:UPI00022BA8F8, UniRef:UniRef100_G4NX79, UniRef:UniRef50_P54530, UniRef:UniRef90_P54530
GO:0000160, GO:0005524, GO:0005886, GO:0006355, GO:0016887, GO:0043565, KEGG:K06714, RefSeq:WP_072182034.1, SO:0001217, UniParc:UPI0006A887E6, UniRef:UniRef100_UPI0006A887E6, UniRef:UniRef50_P5
GO:0005886, SO:0001217, UniRef:UniRef50_O32015, UniRef:UniRef90_O32015
EC:4.1.3.-, EC:4.1.3.30, GO:0019629, GO:0046421, GO:0046872, KEGG:K03417, SO:0001217, UniRef:UniRef50_P54528, UniRef:UniRef90_P54528
EC:4.2.1.-, EC:4.2.1.3, EC:4.2.1.79, GO:0006099, GO:0019679, GO:0030435, GO:0047547, GO:0051537, KEGG:K01720, SO:0001217, UniRef:UniRef50_P74840, UniRef:UniRef90_P45859
EC:2.3.3.-, EC:2.3.3.16, GO:0004108, GO:0005975, GO:0006099, GO:0019679, GO:0030435, GO:0036440, GO:0050440, SO:0001217, UniRef:UniRef50_P45858, UniRef:UniRef90_P45858
EC:1.3.99.-, GO:0003995, GO:0005737, GO:0030435, GO:0033539, GO:0050660, RefSeq:WP_014114336.1, SO:0001217, UniParc:UPI00022BA8FE, UniRef:UniRef100_G4NX85, UniRef:UniRef50_P45857, UniRef:UniRef90_
SO:0001217, UniRef:UniRef50_P45856, UniRef:UniRef90_UPI00037AA63A
PFAM:PF02803.24
EC:2.3.1.9, GO:0003985, GO:0005737, GO:0016747, GO:0030435, SO:0001217, UniRef:UniRef50_P45855, UniRef:UniRef90_P45855
PFAM:PF00108.29
EC:3.5.1.28, GO:0008745, GO:0009253, KEGG:K01448, SO:0001217, UniRef:UniRef50_P54525, UniRef:UniRef90_P54525
SO:0001217, UniRef:UniRef50_P40770, UniRef:UniRef90_A0A9W5PDP7
EC:1.-.-.-, GO:0010181, GO:0016491, SO:0001217, UniRef:UniRef50_P54524, UniRef:UniRef90_P54524
SO:0001217, UniRef:UniRef50_G4NX94, UniRef:UniRef90_G4NX94
RefSeq:WP_014114348.1, SO:0001217, UniParc:UPI00022BA90A, UniRef:UniRef100_G4NX97, UniRef:UniRef50_G4NX97, UniRef:UniRef90_G4NX97
EC:3.1.21.3, KEGG:K01153, SO:0001217, UniParc:UPI00022BA90B, UniRef:UniRef100_G4NX98, UniRef:UniRef50_A0A1B7V9P3, UniRef:UniRef90_G4NX98
EC:3.1.21.3, KEGG:K01154, RefSeq:WP_014114350.1, SO:0001217, UniParc:UPI00022BAA6E, UniRef:UniRef100_G4NQ38, UniRef:UniRef50_G4NQ38, UniRef:UniRef90_G4NQ38
EC:2.1.1.72, GO:0003677, GO:0009007, GO:0009307, KEGG:K03427, RefSeq:WP_014114351.1, SO:0001217, UniParc:UPI00022BAA6F, UniRef:UniRef100_G4NQ39, UniRef:UniRef50_G4NQ39, UniRef:UniRef90_G4NQ39
RefSeq:WP_014114352.1, SO:0001217, UniParc:UPI00022BAA70, UniRef:UniRef100_G4NQ40, UniRef:UniRef50_G4NQ40, UniRef:UniRef90_G4NQ40
SO:0001217, UniParc:UPI00022BAA71, UniRef:UniRef100_G4NQ41, UniRef:UniRef50_G4NQ41, UniRef:UniRef90_G4NQ41
RefSeq:WP_014114354.1, SO:0001217, UniParc:UPI00022BAA72, UniRef:UniRef100_G4NQ42, UniRef:UniRef50_D9S3S7, UniRef:UniRef90_G4NQ42
COG:COG5801, COG:DT, GO:0000160, GO:0003677, GO:0003700, GO:0005509, GO:0005737, GO:0006355, GO:0008356, GO:0030435, GO:0042173, GO:0043937, GO:0045881, GO:0045893, GO:0051606, GO:0090529, G
SO:0001217, UniRef:UniRef50_UPI001D06047A
GO:0005524, GO:0005737, GO:0006281, GO:0006302, GO:0006310, GO:0009432, GO:0016887, GO:0043590, RefSeq:WP_072182046.1, SO:0001217, UniParc:UPI0006A88C11, UniRef:UniRef100_UPI0006A88C11, UniRef:
BlastRules:WP_001032581, BlastRules:WP_003230265, GO:0003677, GO:0003700, GO:0005737, GO:0006355, GO:0006526, GO:0034618, GO:0051259, GO:1900079, KEGG:K03402, RefSeq:WP_014114358.1, SO:000121
EC:2.1.1.-, GO:0003723, GO:0006364, GO:0008168, GO:0032259, RefSeq:WP_072566534.1, SO:0001217, UniParc:UPI00086E0997, UniRef:UniRef100_A0A9Q4E5D9, UniRef:UniRef50_P19672, UniRef:UniRef90_P19672
EC:2.2.1.7, GO:0000287, GO:0005737, GO:0005829, GO:0008615, GO:0008661, GO:0009228, GO:0009240, GO:0016114, GO:0019288, GO:0030976, GO:0052865, KEGG:K01662, SO:0001217, UniRef:UniRef50_P54523, U
EC:2.5.1.10, GO:0004337, GO:0004659, GO:0005737, GO:0008299, GO:0046872, RefSeq:WP_072182049.1, SO:0001217, UniParc:UPI0006A93B3A, UniRef:UniRef100_A0A9Q4E448, UniRef:UniRef50_Q08291, UniRef:UniR
EC:3.1.11.6, GO:0005829, GO:0006308, GO:0008855, GO:0009318, RefSeq:WP_003226409.1, SO:0001217, UniParc:UPI000195C644, UniRef:UniRef100_P54522, UniRef:UniRef50_P54522, UniRef:UniRef90_P54522
EC:3.1.11.6, GO:0003676, GO:0005737, GO:0006308, GO:0008855, GO:0009295, GO:0009318, KEGG:K03601, SO:0001217, UniRef:UniRef50_P54521, UniRef:UniRef90_P54521
EC:1.5.1.5, EC:3.5.4.9, GO:0000105, GO:0004477, GO:0004488, GO:0005829, GO:0006164, GO:0009086, GO:0035999, RefSeq:WP_014114365.1, SO:0001217, UniParc:UPI00022BAA7D, UniRef:UniRef100_G4NQ54, UniR
GO:0003723, GO:0005829, GO:0006353, GO:0031564, KEGG:K03625, RefSeq:WP_014114366.1, SO:0001217, UniParc:UPI00022BAA7E, UniRef:UniRef100_G4NQ55, UniRef:UniRef50_P54520, UniRef:UniRef90_P54520
BlastRules:WP_003230254, RefSeq:WP_003236896.1, SO:0001217, UniParc:UPI00022BAA7F, UniRef:UniRef100_A0A2M8T1W9, UniRef:UniRef50_P54519, UniRef:UniRef90_P54519
EC:6.3.4.14, GO:0003989, GO:0004075, GO:0005524, GO:0006633, GO:0009317, GO:0016874, GO:0046872, GO:2001295, RefSeq:WP_014114367.1, SO:0001217, UniParc:UPI00022BAA80, UniRef:UniRef100_G4NQ57, U
EC:6.4.1.2, GO:0003989, GO:0006633, GO:0009317, RefSeq:WP_072182052.1, SO:0001217, UniParc:UPI0006A8AFA1, UniRef:UniRef100_UPI0006A8AFA1, UniRef:UniRef50_P49786, UniRef:UniRef90_P49786
BlastRules:WP_003230250, GO:0005886, GO:0030435, KEGG:K06397, RefSeq:WP_043857804.1, SO:0001217, UniParc:UPI0005C259E0, UniRef:UniRef100_A0A0D1L783, UniRef:UniRef50_P49785, UniRef:UniRef90_P4978
GO:0005886, GO:0030435, GO:0030436, KEGG:K06396, RefSeq:WP_072182053.1, SO:0001217, UniParc:UPI0006A910C5, UniRef:UniRef100_UPI0006A910C5, UniRef:UniRef50_P49784, UniRef:UniRef90_P49784
GO:0005886, GO:0016020, GO:0030435, GO:0030436, KEGG:K06395, RefSeq:WP_014114371.1, SO:0001217, UniParc:UPI00022BAA84, UniRef:UniRef100_G4NQ61, UniRef:UniRef50_P49783, UniRef:UniRef90_P49783
GO:0005886, GO:0030435, GO:0030436, KEGG:K06394, RefSeq:WP_072182054.1, SO:0001217, UniParc:UPI0006A811E0, UniRef:UniRef100_UPI0006A811E0, UniRef:UniRef50_P49782, UniRef:UniRef90_P49782
GO:0005886, GO:0030435, GO:0030436, KEGG:K06393, RefSeq:WP_003230228.1, SO:0001217, UniParc:UPI00000606F0, UniRef:UniRef100_P49781, UniRef:UniRef50_P49781, UniRef:UniRef90_P49781
COG:COG5824, COG:D, GO:0005886, GO:0030435, GO:0030436, RefSeq:WP_003153142.1, SO:0001217, UniParc:UPI00000606F1, UniRef:UniRef100_P49780, UniRef:UniRef50_P49780, UniRef:UniRef90_P49780
GO:0030435, GO:0030436, KEGG:K06391, RefSeq:WP_003226383.1, SO:0001217, UniParc:UPI0001D283DB, UniRef:UniRef100_A0A9Q4E3D2, UniRef:UniRef50_Q01368, UniRef:UniRef90_Q01368
GO:0005524, GO:0016887, GO:0030435, GO:0030436, KEGG:K06390, RefSeq:WP_072182055.1, SO:0001217, UniParc:UPI0006A93B10, UniRef:UniRef100_UPI0006A93B10, UniRef:UniRef50_Q01367, UniRef:UniRef90_Q0
GO:0005886, RefSeq:WP_003226379.1, SO:0001217, UniParc:UPI0001D283D9, UniRef:UniRef100_A0A9Q4E4D2, UniRef:UniRef50_P49779, UniRef:UniRef90_P49779
GO:0003746, GO:0005737, GO:0006414, GO:0043043, KEGG:K02356, RefSeq:WP_003226377.1, SO:0001217, UniParc:UPI000195C651, UniRef:UniRef100_P49778, UniRef:UniRef50_P49778, UniRef:UniRef90_P49778
BlastRules:WP_003230224, EC:3.4.-.-, GO:0004177, GO:0008235, GO:0046872, KEGG:K01262, SO:0001217, UniRef:UniRef50_P54518, UniRef:UniRef90_P54518
EC:4.2.1.10, GO:0003855, GO:0008652, GO:0009073, GO:0009423, GO:0019631, KEGG:K03786, RefSeq:WP_014114377.1, SO:0001217, UniParc:UPI00022BAA8A, UniRef:UniRef100_G4NQ70, UniRef:UniRef50_P54517, U
SO:0001217, UniRef:UniRef50_K6DBD5
SO:0001217, UniRef:UniRef50_UPI00227EB842
GO:0005886, RefSeq:WP_014114380.1, SO:0001217, UniParc:UPI00022BAA8D, UniRef:UniRef100_G4NQ73, UniRef:UniRef50_P54514, UniRef:UniRef90_P54514
EC:3.1.1.-, GO:0005886, GO:0016042, GO:0016787, RefSeq:WP_014114381.1, SO:0001217, UniParc:UPI00022BAA8E, UniRef:UniRef100_G4NQ74, UniRef:UniRef50_P54513, UniRef:UniRef90_P54513
COG:COG1321, COG:K, GO:0003677, GO:0003700, GO:0005737, GO:0006355, GO:0030026, GO:0030145, GO:0046983, KEGG:K11924, RefSeq:WP_014114382.1, SO:0001217, UniParc:UPI00022BAA8F, UniRef:UniRef10
BlastRules:WP_004398586, EC:2.3.1.181, GO:0009107, GO:0033819, RefSeq:WP_088679274.1, SO:0001217, UniParc:UPI0006A8EE7C, UniRef:UniRef100_UPI0006A8EE7C, UniRef:UniRef50_P54511, UniRef:UniRef90_P54
GO:0005886, RefSeq:WP_014664580.1, SO:0001217, UniParc:UPI00025978DE, UniRef:UniRef100_A0A9Q4E5R2, UniRef:UniRef50_P54510, UniRef:UniRef90_P54510
COG:COG1003, COG:E, EC:1.4.4.2, GO:0004375, GO:0005829, GO:0005960, GO:0006546, GO:0016594, GO:0019464, GO:0030170, RefSeq:WP_061186883.1, SO:0001217, UniParc:UPI0006A859E5, UniRef:UniRef100_UF
EC:1.4.4.2, GO:0004375, GO:0006546, GO:0009116, GO:0019464, SO:0001217, UniRef:UniRef50_P54376, UniRef:UniRef90_P54376
PFAM:PF02347.22
EC:2.1.2.10, GO:0004047, GO:0005737, GO:0005829, GO:0005960, GO:0008483, GO:0019464, RefSeq:WP_072183732.1, SO:0001217, UniParc:UPI0006A82BC2, UniRef:UniRef100_UPI0006A82BC2, UniRef:UniRef50_P54
RFAM:RF00504, SO:0000035
EC:3.6.4.-, GO:0003677, GO:0003682, GO:0004386, GO:0005524, GO:0016787, GO:0031507, GO:0045944, GO:0140750, KEGG:K03580, RefSeq:WP_072566541.1, SO:0001217, UniParc:UPI0006A91EE5, UniRef:UniRef10
SO:0001217, UniRef:UniRef50_P54508, UniRef:UniRef90_P54508
GO:0006355, GO:0046983, RefSeq:WP_003226347.1, SO:0001217, UniParc:UPI0001D283CB, UniRef:UniRef100_A0A9Q4DMV5, UniRef:UniRef50_P23308, UniRef:UniRef90_P23308
BlastRules:WP_003226345, COG:COG1396, COG:K, GO:0003677, GO:0003700, GO:0006355, GO:0010629, GO:0030435, GO:0045892, GO:0046983, SO:0001217, UniParc:UPI0001CE373F, UniRef:UniRef100_A0A086W10
BlastRules:WP_004398632, GO:0005576, GO:0030435, GO:0042802, RefSeq:WP_014114390.1, SO:0001217, UniParc:UPI00022BABF3, UniRef:UniRef100_G4NQ85, UniRef:UniRef50_P54507, UniRef:UniRef90_P54507
EC:3.4.21.89, GO:0004252, GO:0005886, GO:0006465, RefSeq:WP_014114391.1, SO:0001217, UniParc:UPI00022BABF4, UniRef:UniRef100_G4NQ86, UniRef:UniRef50_P54506, UniRef:UniRef90_P54506
GO:0097311, KEGG:K19433, RefSeq:WP_090558099.1, SO:0001217, UniParc:UPI000869E3C6, UniRef:UniRef100_UPI000869E3C6, UniRef:UniRef50_P40949, UniRef:UniRef90_E0U410
RefSeq:WP_014114393.1, SO:0001217, UniParc:UPI00022BABF6, UniRef:UniRef100_G4NQ88, UniRef:UniRef50_O32019, UniRef:UniRef90_O32019
RefSeq:WP_003226330.1, SO:0001217, UniParc:UPI0001D283C6, UniRef:UniRef100_A0A9Q4HGK4, UniRef:UniRef50_O32020, UniRef:UniRef90_O32020
KEGG:K02249, RefSeq:WP_072183695.1, SO:0001217, UniParc:UPI0006A87247, UniRef:UniRef100_UPI0006A87247, UniRef:UniRef50_P25959, UniRef:UniRef90_E0U413
SO:0001217, UniRef:UniRef50_P25958, UniRef:UniRef90_E0U414
KEGG:K02247, RefSeq:WP_072183693.1, SO:0001217, UniParc:UPI0006A8D900, UniRef:UniRef100_UPI0006A8D900, UniRef:UniRef50_P25957, UniRef:UniRef90_E0U415
SO:0001217, UniRef:UniRef50_P25956, UniRef:UniRef90_A0A2M8T1V0
BlastRules:WP_001178688, BlastRules:WP_003230162, GO:0005886, GO:0009289, GO:0009986, GO:0030420, RefSeq:WP_014114400.1, SO:0001217, UniParc:UPI00022BABFD, UniRef:UniRef100_G4NQ96, UniRef:UniRe
GO:0005886, GO:0009306, GO:0030420, RefSeq:WP_014114401.1, SO:0001217, UniParc:UPI00022BABFE, UniRef:UniRef100_G4NQ97, UniRef:UniRef50_P25954, UniRef:UniRef90_P25954
BlastRules:WP_001013201, BlastRules:WP_004399124, GO:0005524, GO:0005886, GO:0016887, GO:0030420, RefSeq:WP_072183690.1, SO:0001217, UniParc:UPI0006A8FED3, UniRef:UniRef100_UPI0006A8FED3, UniRe
RefSeq:WP_014114403.1, SO:0001217, UniParc:UPI00022BAC00, UniRef:UniRef100_G4NQ99, UniRef:UniRef50_E0U420, UniRef:UniRef90_E0U420
GO:0000287, GO:0005886, GO:0015087, GO:0015095, GO:0050897, RefSeq:WP_072183689.1, SO:0001217, UniParc:UPI0006A850C0, UniRef:UniRef100_A0A9Q4E352, UniRef:UniRef50_P40948, UniRef:UniRef90_P40948
GO:0005886, GO:0050660, KEGG:K03699, SO:0001217, UniRef:UniRef50_O05241, UniRef:UniRef90_P54505
RefSeq:WP_014114406.1, SO:0001217, UniParc:UPI00022BAC03, UniRef:UniRef100_G4NQA2, UniRef:UniRef50_P54504, UniRef:UniRef90_E0U423
SO:0000261
BlastRules:WP_003230147, GO:0005737, KEGG:K16509, RefSeq:WP_003226302.1, SO:0001217, UniParc:UPI0001D2830D, UniRef:UniRef100_A0A9Q4HHQ6, UniRef:UniRef50_P54503, UniRef:UniRef90_P54503
GO:0005886, RefSeq:WP_003226300.1, SO:0001217, UniParc:UPI0000060716, UniRef:UniRef100_P54502, UniRef:UniRef50_P54502, UniRef:UniRef90_P54502
EC:3.-.-.-, GO:0016787, GO:0046872, SO:0001217, UniRef:UniRef50_P54501, UniRef:UniRef90_P54501
GO:0005886, RefSeq:WP_014114408.1, SO:0001217, UniParc:UPI00022BAC05, UniRef:UniRef100_G4NQA6, UniRef:UniRef50_P54500, UniRef:UniRef90_P54500
RefSeq:WP_010334936.1, SO:0001217, UniParc:UPI00022BAC06, UniRef:UniRef100_A0A9Q2QW89, UniRef:UniRef50_Q56200, UniRef:UniRef90_P54499
RefSeq:WP_072181772.1, SO:0001217, UniParc:UPI0006A8D546, UniRef:UniRef100_UPI0006A8D546, UniRef:UniRef50_P54498, UniRef:UniRef90_A0A6H0WL13

GO:0004181, GO:0006508, GO:0008270, SO:0001217, UniRef:UniRef50_P54497, UniRef:UniRef90_P54497
GO:0005576, GO:0005886, GO:0046872, RefSeq:WP_014114411.1, SO:0001217, UniParc:UPI00022BAC09, UniRef:UniRef100_G4NQB0, UniRef:UniRef50_P54496, UniRef:UniRef90_P54496
SO:0001217, UniRef:UniRef50_W4RXH1
SO:0001217, UniRef:UniRef50_P54494, UniRef:UniRef90_E0U434
EC:3.4.21.105, KEGG:K19225, SO:0001217, UniRef:UniRef50_UPI00227EFDCC, UniRef:UniRef90_UPI00227EFDCC
SO:0001217, UniRef:UniRef50_P54492, UniRef:UniRef90_P54492
EC:6.3.3.2, GO:0006730, GO:0030272, RefSeq:WP_072181768.1, SO:0001217, UniParc:UPI0006A86071, UniRef:UniRef100_UPI0006A86071, UniRef:UniRef50_P54491, UniRef:UniRef90_E0U438
COG:COG0267, COG:J, GO:0003735, GO:0005737, GO:0005840, GO:0006412, GO:1990904, KEGG:K02913, RefSeq:WP_003153056.1, SO:0001217, UniParc:UPI00015978F9, UniRef:UniRef100_P56849, UniRef:UniRef50_
RefSeq:WP_072181767.1, SO:0001217, UniParc:UPI0006A8D709, UniRef:UniRef100_UPI0006A8D709, UniRef:UniRef50_P54490, UniRef:UniRef90_G4NPV1
RefSeq:WP_072181766.1, SO:0001217, UniParc:UPI0006A89586, UniRef:UniRef100_UPI0006A89586, UniRef:UniRef50_P54489, UniRef:UniRef90_G4NPV2
GO:0005886, RefSeq:WP_003226272.1, SO:0001217, UniParc:UPI0001D282FE, UniRef:UniRef100_A0A9Q4DMZ8, UniRef:UniRef50_O32022, UniRef:UniRef90_O32022
RefSeq:WP_014114420.1, SO:0001217, UniParc:UPI00022BAC12, UniRef:UniRef100_G4NPV4, UniRef:UniRef50_O32023, UniRef:UniRef90_O32023
EC:7.3.2.1, GO:0005524, GO:0005886, GO:0006817, GO:0009898, GO:0015415, GO:0016887, GO:0035435, GO:0055052, RefSeq:WP_061186865.1, SO:0001217, UniParc:UPI00077234B4, UniRef:UniRef100_UPI00077234
EC:7.3.2.1, GO:0005524, GO:0005886, GO:0006817, GO:0009898, GO:0015415, GO:0016887, GO:0035435, GO:0055052, SO:0001217, UniRef:UniRef50_P46341, UniRef:UniRef90_P46341
GO:0005315, GO:0005886, GO:0006817, GO:0015415, GO:0035435, GO:0055052, RefSeq:WP_088679282.1, SO:0001217, UniParc:UPI000B531A6A, UniRef:UniRef100_UPI000B531A6A, UniRef:UniRef50_P46340, UniRef:U
GO:0005315, GO:0005886, GO:0006817, GO:0015415, GO:0055052, RefSeq:WP_013308020.1, SO:0001217, UniParc:UPI0001E14EF1, UniRef:UniRef100_A0A9Q4E403, UniRef:UniRef50_P46339, UniRef:UniRef90_P46339
GO:0005886, GO:0006817, GO:0042301, SO:0001217, UniRef:UniRef50_P46338, UniRef:UniRef90_P46338
EC:3.4.16.4, GO:0005886, GO:0006508, GO:0008360, GO:0008658, GO:0009002, GO:0009252, GO:0046677, GO:0071555, GO:0071972, RefSeq:WP_061186864.1, SO:0001217, UniParc:UPI000772B537, UniRef:UniRef100
GO:0005886, GO:0022857, RefSeq:WP_061186863.1, SO:0001217, UniParc:UPI000772B194, UniRef:UniRef100_A0A9Q4E577, UniRef:UniRef50_P54487, UniRef:UniRef90_P54487
EC:1.15.1.1, GO:0004784, GO:0005576, GO:0005737, GO:0019430, GO:0046872, KEGG:K04564, RefSeq:WP_014114427.1, SO:0001217, UniParc:UPI00022BADAB, UniRef:UniRef100_G4NPR1, UniRef:UniRef50_P54375, U
GO:0005886, KEGG:K09793, SO:0001217, UniRef:UniRef50_P54486, UniRef:UniRef90_P54486
COG:COG0821, COG:I, EC:1.17.7.1, EC:1.17.7.3, GO:0005506, GO:0008152, GO:0016114, GO:0019288, GO:0046429, GO:0051539, GO:0141197, KEGG:K03526, SO:0001217, UniRef:UniRef50_Q81LV7, UniRef:UniRef90_F
GO:0005886, SO:0001217, UniParc:UPI00022BADAF, UniRef:UniRef100_G4NPR6, UniRef:UniRef50_P54481, UniRef:UniRef90_P54481
EC:3.1.3.-, GO:0016787, KEGG:K05967, RefSeq:WP_072181757.1, SO:0001217, UniParc:UPI0006A94075, UniRef:UniRef100_UPI0006A94075, UniRef:UniRef50_P54480, UniRef:UniRef90_P54480
BlastRules:WP_003899290, BlastRules:WP_004398578, GO:0000976, GO:0001217, GO:0003700, GO:0005737, GO:0008270, GO:0032993, GO:0045892, GO:1900376, KEGG:K02076, RefSeq:WP_014114433.1, SO:000121
GO:0005886, RefSeq:WP_014114434.1, SO:0001217, UniParc:UPI00022BADB2, UniRef:UniRef100_G4NPR9, UniRef:UniRef50_P54478, UniRef:UniRef90_P54478
RefSeq:WP_072181756.1, SO:0001217, UniParc:UPI0006A9025B, UniRef:UniRef100_UPI0006A9025B, UniRef:UniRef50_P54477, UniRef:UniRef90_P54477
EC:3.1.21.2, GO:0003677, GO:0003906, GO:0006281, GO:0006284, GO:0008081, GO:0008270, GO:0008833, RefSeq:WP_032727307.1, SO:0001217, UniParc:UPI000505133B, UniRef:UniRef100_A0A9Q4H9E8, UniRef:Unil
BlastRules:WP_003721956, BlastRules:WP_004399101, EC:3.6.4.13, GO:0003689, GO:0003723, GO:0003724, GO:0005524, GO:0005829, GO:0006401, GO:0009409, GO:0016887, GO:0033592, GO:0042254, GO:0043590,
RefSeq:WP_072183566.1, SO:0001217, UniParc:UPI0006A8C3A2, UniRef:UniRef100_UPI0006A8C3A2, UniRef:UniRef50_P54474, UniRef:UniRef90_A0A5D4NA38
COG:COG0761, COG:I, EC:1.17.7.4, GO:0016114, GO:0019288, GO:0046872, GO:0050992, GO:0051539, GO:0051745, KEGG:K03527, RefSeq:WP_014114439.1, SO:0001217, UniParc:UPI00022BADB7, UniRef:UniRef100
EC:3.5.4.16, GO:0005737, GO:0046872, GO:0051540, KEGG:K22391, RefSeq:WP_072183189.1, SO:0001217, UniParc:UPI0006A871BA, UniRef:UniRef100_UPI0006A871BA, UniRef:UniRef50_P54472, UniRef:UniRef90_P5
EC:2.1.1.217, GO:0005737, GO:0008033, GO:0032259, GO:0160105, KEGG:K06967, SO:0001217, UniRef:UniRef50_P54471, UniRef:UniRef90_P54471
BlastRules:WP_000828148, BlastRules:WP_003230068, GO:0005506, GO:0005886, GO:0009055, GO:0020037, RefSeq:WP_014114442.1, SO:0001217, UniParc:UPI00022BADBA, UniRef:UniRef100_G4NPS7, UniRef:UniRe
COG:COG0568, COG:K, GO:0003677, GO:0005737, GO:0006352, GO:0006355, GO:0016987, KEGG:K03086, RefSeq:WP_003226225.1, SO:0001217, UniParc:UPI000195C683, UniRef:UniRef100_P06224, UniRef:UniRef50_
EC:2.7.7.101, GO:0000428, GO:0003677, GO:0003678, GO:0003896, GO:0005524, GO:0005737, GO:0006269, GO:0008270, GO:1990077, KEGG:K02316, RefSeq:WP_088679287.1, SO:0001217, UniParc:UPI000B52BC14,
SO:0001217, UniRef:UniRef50_P17868
EC:2.7.11.32, EC:2.7.4.27, GO:0004674, GO:0005524, GO:0016776, GO:0043531, RefSeq:WP_003226219.1, SO:0001217, UniParc:UPI0001D2905C, UniRef:UniRef100_G4NPT2, UniRef:UniRef50_P54470, UniRef:UniRef90_
BlastRules:WP_003237058, SO:0001217, UniRef:UniRef50_A0A2T5G6Z1, UniRef:UniRef90_A0A7W4LQZ7
SO:0001217, UniRef:UniRef50_UPI002280092D
SO:0001217, UniRef:UniRef50_A0A3C2CY57
COG:COG0752, COG:J, EC:6.1.1.14, GO:0000166, GO:0004820, GO:0005524, GO:0005829, GO:0006426, GO:0016740, KEGG:K01878, SO:0001217, UniRef:UniRef50_P54380, UniRef:UniRef90_P54380
GO:0003677, GO:0005737, GO:0006281, GO:0006302, GO:0006310, GO:0043590, KEGG:K03584, RefSeq:WP_014114447.1, SO:0001217, UniParc:UPI00022BADBF, UniRef:UniRef100_G4NPT6, UniRef:UniRef50_P42095, U
RefSeq:WP_003230053.1, SO:0001217, UniParc:UPI000195C687, UniRef:UniRef100_C0H452, UniRef:UniRef50_C0H452, UniRef:UniRef90_C0H452
COG:COG1159, COG:J, GO:0000028, GO:0003723, GO:0003924, GO:0005525, GO:0005829, GO:0005886, GO:0019843, GO:0032297, GO:0043024, GO:0051781, GO:0070181, RefSeq:WP_014114448.1, SO:0001217, Uni
EC:3.5.4.5, GO:0004126, GO:0005829, GO:0008270, GO:0009972, GO:0042802, KEGG:K01489, RefSeq:WP_003226204.1, SO:0001217, UniParc:UPI0001D29057, UniRef:UniRef100_A0A9Q4HD69, UniRef:UniRef50_P1907
EC:2.7.1.66, GO:0005524, GO:0005886, GO:0008654, GO:0030435, GO:0036433, RefSeq:WP_014114450.1, SO:0001217, UniParc:UPI00022BADC2, UniRef:UniRef100_G4NPU3, UniRef:UniRef50_P19638, UniRef:UniRef90
EC:3.1.-.-, GO:0004222, GO:0004521, GO:0004540, GO:0005737, GO:0006364, GO:0008270, GO:0042254, GO:0046872, RefSeq:WP_014114451.1, SO:0001217, UniParc:UPI00022BADC3, UniRef:UniRef100_G4NPU4, Uni
BlastRules:WP_003721972, BlastRules:WP_010886570, EC:3.1.4.59, GO:0005886, GO:0016787, GO:0046872, GO:0106409, KEGG:K07037, RefSeq:WP_072183154.1, SO:0001217, UniParc:UPI0006A82B21, UniRef:UniRef
RefSeq:WP_014114453.1, SO:0001217, UniParc:UPI00022BADC5, UniRef:UniRef100_G4NPU6, UniRef:UniRef50_G4NPU6, UniRef:UniRef90_G4NPU6
GO:0005524, GO:0005829, KEGG:K06217, RefSeq:WP_003226195.1, SO:0001217, UniParc:UPI0001D29053, UniRef:UniRef100_A0A9Q4DQ56, UniRef:UniRef50_P46343, UniRef:UniRef90_P46343
GO:0005886, GO:0030435, GO:0030436, KEGG:K06438, RefSeq:WP_044428713.1, SO:0001217, UniParc:UPI0005C97576, UniRef:UniRef100_A0A9Q4DMM6, UniRef:UniRef50_P54469, UniRef:UniRef90_P54469
GO:0030436, RefSeq:WP_003226191.1, SO:0001217, UniParc:UPI000006074E, UniRef:UniRef100_P54468, UniRef:UniRef50_P54468, UniRef:UniRef90_P54468
RefSeq:WP_072183155.1, SO:0001217, UniParc:UPI0006A9311F, UniRef:UniRef100_UPI0006A9311F, UniRef:UniRef50_P54467, UniRef:UniRef90_A0A5D4N9X6
BlastRules:WP_003230026, COG:COG4864, COG:M, GO:0005886, GO:0045121, RefSeq:WP_003226187.1, SO:0001217, UniParc:UPI0001D29050, UniRef:UniRef100_A0A9Q4DMA1, UniRef:UniRef50_P54466, UniRef:UniR
GO:0005886, KEGG:K07403, RefSeq:WP_072183156.1, SO:0001217, UniParc:UPI0006A89B97, UniRef:UniRef100_A0A9Q4HJ25, UniRef:UniRef50_P54465, UniRef:UniRef90_P54465
COG:COG1610, COG:R, KEGG:K09117, RefSeq:WP_003226182.1, SO:0001217, UniParc:UPI0001D2904E, UniRef:UniRef100_A0A9Q4E6S0, UniRef:UniRef50_P54464, UniRef:UniRef90_Q65H62
BlastRules:WP_000048061, COG:COG0828, COG:J, GO:0003735, GO:0005840, GO:0006412, GO:1990904, RefSeq:WP_003152957.1, SO:0001217, UniParc:UPI0000060753, UniRef:UniRef100_P21478, UniRef:UniRef50_P
GO:0005436, GO:0005886, GO:0044341, SO:0001217, UniRef:UniRef50_P54463, UniRef:UniRef90_P54463
COG:COG0621, COG:J, EC:2.8.4.5, GO:0005737, GO:0006400, GO:0035598, GO:0035600, GO:0046872, GO:0051539, GO:0061712, GO:1904047, KEGG:K18707, SO:0001217, UniRef:UniRef50_P54462, UniRef:UniRef90_I
EC:2.1.1.193, GO:0005737, GO:0006364, GO:0008168, GO:0070042, GO:0070475, KEGG:K09761, SO:0001217, UniRef:UniRef50_P54461, UniRef:UniRef90_P54461
EC:2.1.1.-, GO:0005737, GO:0008757, GO:0032259, GO:0036211, GO:0042054, SO:0001217, UniRef:UniRef50_P54460, UniRef:UniRef90_P54460
COG:COG0484, COG:O, GO:0005524, GO:0005737, GO:0006260, GO:0006457, GO:0008270, GO:0009408, GO:0031072, GO:0042026, GO:0051082, GO:0051085, RefSeq:WP_014114461.1, SO:0001217, UniParc:UPI0002
RFAM:RF02378, SO:0000655
COG:COG0443, COG:O, GO:0005524, GO:0005576, GO:0005829, GO:0006457, GO:0016887, GO:0031072, GO:0042026, GO:0044183, GO:0051082, GO:0051085, GO:0140662, RefSeq:WP_014114462.1, SO:0001217, Un
GO:0000774, GO:0005737, GO:0006457, GO:0042803, GO:0051082, GO:0051087, KEGG:K03687, RefSeq:WP_003237097.1, SO:0001217, UniParc:UPI0002AC18B6, UniRef:UniRef100_A0A9W5LJU8, UniRef:UniRef50_P15
GO:0003677, GO:0006355, GO:0045892, RefSeq:WP_090558124.1, SO:0001217, UniParc:UPI00086F0410, UniRef:UniRef100_UPI00086F0410, UniRef:UniRef50_P25499, UniRef:UniRef90_P25499
GO:0004109, GO:0005737, GO:0006779, GO:0046872, GO:0051539, GO:0051989, RefSeq:WP_072183546.1, SO:0001217, UniParc:UPI0006A8B604, UniRef:UniRef100_UPI0006A8B604, UniRef:UniRef50_P54304, UniRef:U
COG:COG0481, COG:J, EC:3.6.5.n1, GO:0003746, GO:0003924, GO:0005525, GO:0005886, GO:0043022, GO:0045727, GO:1990606, RefSeq:WP_014114466.1, SO:0001217, UniParc:UPI00022BAF4A, UniRef:UniRef100_(
RefSeq:WP_061186856.1, SO:0001217, UniParc:UPI0006A9087B, UniRef:UniRef100_UPI0006A9087B, UniRef:UniRef50_P38425, UniRef:UniRef90_P38425
GO:0030435, KEGG:K06385, RefSeq:WP_072183545.1, SO:0001217, UniParc:UPI0006A8FB51, UniRef:UniRef100_UPI0006A8FB51, UniRef:UniRef50_P37968, UniRef:UniRef90_P37968
EC:3.4.24.78, GO:0004175, GO:0004222, GO:0006508, GO:0009847, GO:0030436, KEGG:K06012, RefSeq:WP_041905682.1, SO:0001217, UniParc:UPI0004FF6978, UniRef:UniRef100_A0A9Q4HJ21, UniRef:UniRef50_P223
GO:0003723, GO:0003735, GO:0005829, GO:0005840, GO:0006412, GO:0015935, GO:0070181, KEGG:K02968, RefSeq:WP_003229989.1, SO:0001217, UniParc:UPI000195C6A0, UniRef:UniRef100_P21477, UniRef:UniRef
EC:2.7.7.7, GO:0003677, GO:0003887, GO:0005737, GO:0006260, GO:0006261, GO:0009295, GO:0009360, RefSeq:WP_061186855.1, SO:0001217, UniParc:UPI0006A864A1, UniRef:UniRef100_A0A9Q4E8C3, UniRef:UniR
SO:0001217, UniRef:UniRef50_P39695
EC:3.5.-.-, GO:0004132, GO:0005737, GO:0008270, GO:0009972, RefSeq:WP_061186853.1, SO:0001217, UniParc:UPI0006A852A3, UniRef:UniRef100_A0A9Q4E6P0, UniRef:UniRef50_P32393, UniRef:UniRef90_P32393
RefSeq:WP_072183542.1, SO:0001217, UniParc:UPI0006A7F2CD, UniRef:UniRef100_UPI0006A7F2CD, UniRef:UniRef50_P39694, UniRef:UniRef90_E0U4S5
GO:0004735, GO:0006561, GO:0055129, KEGG:K02239, RefSeq:WP_072183541.1, SO:0001217, UniParc:UPI0006A8553F, UniRef:UniRef100_UPI0006A8553F, UniRef:UniRef50_P39696, UniRef:UniRef90_P39696
EC:2.1.1.-, GO:0008168, GO:0032259, SO:0001217, UniRef:UniRef50_P54458, UniRef:UniRef90_P54458
GO:0005737, GO:0017148, GO:0042256, GO:0043022, GO:0043023, GO:0090071, RefSeq:WP_003237112.1, SO:0001217, UniParc:UPI00022BAF54, UniRef:UniRef100_A0A2M8T1L0, UniRef:UniRef50_P54457, UniRef:UniF
EC:3.6.1.41, GO:0000166, GO:0016787, GO:0046872, RefSeq:WP_072183539.1, SO:0001217, UniParc:UPI0006A87E88, UniRef:UniRef100_UPI0006A87E88, UniRef:UniRef50_P54456, UniRef:UniRef90_P54456
SO:0001217, UniRef:UniRef50_W7DWT1
SO:0001217, UniRef:UniRef50_A0A5S9MGD8, UniRef:UniRef90_A0A5S9MGD8
GO:0003723, RefSeq:WP_003226133.1, SO:0001217, UniParc:UPI000006076A, UniRef:UniRef100_P54454, UniRef:UniRef50_P54454, UniRef:UniRef90_P54454
EC:1.1.1.25, GO:0004764, GO:0005829, GO:0008652, GO:0009073, GO:0009423, GO:0019632, GO:0050661, SO:0001217, UniRef:UniRef50_P54374, UniRef:UniRef90_P54374
EC:1.1.1.25, GO:0004764, GO:0005829, GO:0008652, GO:0009073, GO:0009423, GO:0019632, GO:0050661, SO:0001217, UniRef:UniRef50_P54374, UniRef:UniRef90_P54374
GO:0003924, GO:0005525, GO:0005737, GO:0032297, GO:0042254, RefSeq:WP_014114480.1, SO:0001217, UniParc:UPI00022BAF59, UniRef:UniRef100_G4NXC7, UniRef:UniRef50_P54453, UniRef:UniRef90_P54453
COG:COG2179, COG:R, EC:3.1.3.-, GO:0005737, GO:0008962, GO:0016791, SO:0001217, UniParc:UPI00045F63D2, UniRef:UniRef100_D4FZ12, UniRef:UniRef50_P54452, UniRef:UniRef90_P54452
SO:0001217, UniRef:UniRef50_E0U4T6, UniRef:UniRef90_E0U4T6
GO:0005886, GO:0016788, RefSeq:WP_003226118.1, SO:0001217, UniParc:UPI0001D28F65, UniRef:UniRef100_A0A9Q4DML3, UniRef:UniRef50_P54451, UniRef:UniRef90_P54451
EC:3.5.1.28, GO:0005576, GO:0008745, GO:0009253, GO:0009254, GO:0030420, GO:0030435, GO:0071555, RefSeq:WP_072183536.1, SO:0001217, UniParc:UPI0006A8DD90, UniRef:UniRef100_UPI0006A8DD90, UniRef:
RefSeq:WP_014114483.1, SO:0001217, UniParc:UPI00022BAF5C, UniRef:UniRef100_G4NXD2, UniRef:UniRef50_P54449, UniRef:UniRef90_E0U4U0
EC:1.1.1.-, EC:1.1.1.343, GO:0004616, GO:0006098, GO:0016054, GO:0016616, GO:0019521, GO:0050661, KEGG:K00033, RefSeq:WP_079287983.1, SO:0001217, UniParc:UPI00086C3C86, UniRef:UniRef100_UPI00086C
SO:0001217, UniRef:UniRef50_P54447, UniRef:UniRef90_A0A9Q4E6Y9
BlastRules:WP_009967785, EC:3.-.-.-, GO:0004518, GO:0005576, GO:0030435, RefSeq:WP_014114486.1, SO:0001217, UniParc:UPI00022BAF5F, UniRef:UniRef100_G4NXD5, UniRef:UniRef50_P42983, UniRef:UniRef90_P
COG:COG5814, COG:DK, GO:0003677, GO:0003700, GO:0006352, GO:0006355, GO:0016987, GO:0030435, KEGG:K03091, RefSeq:WP_013308023.1, SO:0001217, UniParc:UPI00015FF696, UniRef:UniRef100_D4FZ22, U
SO:0001217, UniRef:UniRef50_E0U4U9
RefSeq:WP_014114487.1, SO:0001217, UniParc:UPI00022BAF60, UniRef:UniRef100_G4NXD7, UniRef:UniRef50_A0A147K511, UniRef:UniRef90_G4NXD7
COG:COG3616, COG:E, RefSeq:WP_014114488.1, SO:0001217, UniParc:UPI00022BAF61, UniRef:UniRef100_G4NXD8, UniRef:UniRef50_W8TJG7, UniRef:UniRef90_Q65H18
COG:COG0251, COG:V, RefSeq:WP_041521011.1, SO:0001217, UniParc:UPI0005A1F3A8, UniRef:UniRef100_G4NXD9, UniRef:UniRef50_Q65H17, UniRef:UniRef90_Q65H17
RefSeq:WP_014114490.1, SO:0001217, UniParc:UPI00022BAF63, UniRef:UniRef100_G4NXE0, UniRef:UniRef50_G4NXE0, UniRef:UniRef90_G4NXE0
COG:COG0498, COG:E, RefSeq:WP_014114491.1, SO:0001217, UniParc:UPI00022BB0DF, UniRef:UniRef100_G4NXE1, UniRef:UniRef50_I0JIK5, UniRef:UniRef90_A0A6I7FFA2
COG:COG0251, COG:V, RefSeq:WP_014114492.1, SO:0001217, UniParc:UPI00022BB0E0, UniRef:UniRef100_G4NXE2, UniRef:UniRef50_Q62SH1, UniRef:UniRef90_Q62SH1
COG:COG2964, COG:K, RefSeq:WP_014114493.1, SO:0001217, UniParc:UPI00022BB0E1, UniRef:UniRef100_G4NXE3, UniRef:UniRef50_A0A1G8Q7U8, UniRef:UniRef90_A0A1R1QSE2
SO:0001217, UniRef:UniRef50_UPI001BA4FF8E, UniRef:UniRef90_UPI000BA89502
PFAM:PF00515.34, PFAM:PF07719.23, PFAM:PF13181.12
BlastRules:WP_004398770, GO:0005576, GO:0005737, GO:0030435, KEGG:K06354, SO:0001217, UniRef:UniRef50_O32025, UniRef:UniRef90_O32025
SO:0001217, UniRef:UniRef50_P45942
RefSeq:WP_014114498.1, SO:0001217, UniParc:UPI00022BB0E5, UniRef:UniRef100_G4NXE8, UniRef:UniRef50_Q81H91, UniRef:UniRef90_G4NXE8
RefSeq:WP_014114499.1, SO:0001217, UniParc:UPI00022BB0E6, UniRef:UniRef100_G4NXE9, UniRef:UniRef50_G4NXE9, UniRef:UniRef90_G4NXE9
PFAM:PF18624.6
KEGG:K21497, RefSeq:WP_087986849.1, SO:0001217, UniParc:UPI00086A4373, UniRef:UniRef100_UPI00086A4373, UniRef:UniRef50_A0A164F662, UniRef:UniRef90_G4NXF1
SO:0001217, UniParc:UPI00022BB0E9, UniRef:UniRef100_G4NXF2, UniRef:UniRef50_G4NXF2, UniRef:UniRef90_G4NXF2
RefSeq:WP_072183224.1, SO:0001217, UniParc:UPI00092E20D2, UniRef:UniRef100_UPI00092E20D2, UniRef:UniRef50_A0A0E3WJN7, UniRef:UniRef90_G4NXF3
SO:0001217, UniRef:UniRef50_Q97LM7, UniRef:UniRef90_G4NXF4
RefSeq:WP_087986848.1, SO:0001217, UniParc:UPI0006A83E79, UniRef:UniRef100_UPI0006A83E79, UniRef:UniRef50_P54437, UniRef:UniRef90_G4NXF5
SO:0001217, UniRef:UniRef50_A0A0J5V686, UniRef:UniRef90_UPI000E734546
GO:0006749, GO:0050313, GO:0070813, SO:0001217, UniRef:UniRef50_P54435, UniRef:UniRef90_P54435
RefSeq:WP_072183435.1, SO:0001217, UniParc:UPI0006A8BFFD, UniRef:UniRef100_UPI0006A8BFFD, UniRef:UniRef50_P54433, UniRef:UniRef90_P54433
SO:0001217, UniRef:UniRef50_A0AA96S5H2, UniRef:UniRef90_A0AAX3RT91
KEGG:K07092, RefSeq:WP_072183437.1, SO:0001217, UniParc:UPI0006A8FCE8, UniRef:UniRef100_UPI0006A8FCE8, UniRef:UniRef50_P54432, UniRef:UniRef90_P54432
GO:0003677, GO:0045892, GO:0046872, SO:0001217, UniRef:UniRef50_P54431, UniRef:UniRef90_P54431
SO:0001217, UniRef:UniRef50_UPI002E1B154A, UniRef:UniRef90_UPI001E53B92E
RefSeq:WP_014114512.1, SO:0001217, UniParc:UPI00022BB0F3, UniRef:UniRef100_G4NXG2, UniRef:UniRef50_G4NXG2, UniRef:UniRef90_G4NXG2
RefSeq:WP_014114514.1, SO:0001217, UniParc:UPI00022BB0F5, UniRef:UniRef100_G4NXG4, UniRef:UniRef50_C0ZDF0, UniRef:UniRef90_G4NXG4
COG:COG0446, COG:I, RefSeq:WP_014114515.1, SO:0001217, UniParc:UPI00022BB0F6, UniRef:UniRef100_G4NXG5, UniRef:UniRef50_C0ZDE9, UniRef:UniRef90_G4NXG5
COG:COG2197, COG:TK, RefSeq:WP_014114516.1, SO:0001217, UniParc:UPI00022BB0F7, UniRef:UniRef100_G4NXG6, UniRef:UniRef50_A0A2S0K1I6, UniRef:UniRef90_Q65H06
COG:COG4585, COG:T, SO:0001217, UniRef:UniRef50_A0A0K9F9N0, UniRef:UniRef90_Q62SG3
RefSeq:WP_014114518.1, SO:0001217, UniParc:UPI00022BB0F9, UniRef:UniRef100_G4NXG8, UniRef:UniRef50_P54437, UniRef:UniRef90_G4NXG8
RefSeq:WP_014114519.1, SO:0001217, UniParc:UPI00022BB0FA, UniRef:UniRef100_G4NXG9, UniRef:UniRef50_N8QEB6, UniRef:UniRef90_G4NXG9
RefSeq:WP_014114520.1, SO:0001217, UniParc:UPI00022BB0FB, UniRef:UniRef100_G4NXH0, UniRef:UniRef50_P96655, UniRef:UniRef90_A0AAE2EMD9
SO:0001217, UniParc:UPI00022BB0FC, UniRef:UniRef100_G4NXH1, UniRef:UniRef50_G4NXH1, UniRef:UniRef90_G4NXH1
RefSeq:WP_014114523.1, SO:0001217, UniParc:UPI00022BB0FE, UniRef:UniRef100_G4NXH3, UniRef:UniRef50_S1NI88, UniRef:UniRef90_G4NXH3
KEGG:K00389, RefSeq:WP_041521014.1, SO:0001217, UniParc:UPI0005A0CB48, UniRef:UniRef100_UPI0005A0CB48, UniRef:UniRef50_A0A0K9GSS6, UniRef:UniRef90_UPI0005A0CB48

RefSeq:WP_014114524.1, SO:0001217, UniParc:UPI00022BB0FF, UniRef:UniRef100_G4NXH4, UniRef:UniRef50_Q46130, UniRef:UniRef90_G4NXH4
EC:2.7.1.-, KEGG:K02757, RefSeq:WP_014114525.1, SO:0001217, UniParc:UPI00022BB100, UniRef:UniRef100_G4NXH5, UniRef:UniRef50_M1MWZ9, UniRef:UniRef90_G4NXH5
EC:7.2.2.8, GO:0005507, GO:0046872, KEGG:K17686, RefSeq:WP_014114526.1, SO:0001217, UniParc:UPI00022BB101, UniRef:UniRef100_G4NXH6, UniRef:UniRef50_Q4A0G1, UniRef:UniRef90_G4NXH6
GO:0005507, RefSeq:WP_014114527.1, SO:0001217, UniParc:UPI00022BB102, UniRef:UniRef100_G4NXH7, UniRef:UniRef50_Q4A0G2, UniRef:UniRef90_G4NXH7
KEGG:K21600, RefSeq:WP_014114528.1, SO:0001217, UniParc:UPI00022BB103, UniRef:UniRef100_G4NXH8, UniRef:UniRef50_A0A2W0C5J5, UniRef:UniRef90_G4NXH8
RefSeq:WP_014114529.1, SO:0001217, UniParc:UPI00022BB104, UniRef:UniRef100_G4NXQ8, UniRef:UniRef50_G4NXQ8, UniRef:UniRef90_G4NXQ8
RefSeq:WP_014114530.1, SO:0001217, UniParc:UPI00022BB105, UniRef:UniRef100_G4NXQ9, UniRef:UniRef50_G4NXQ9, UniRef:UniRef90_G4NXQ9
SO:0001217, UniParc:UPI00022BB106, UniRef:UniRef100_G4NXR0, UniRef:UniRef50_G4NXR0, UniRef:UniRef90_G4NXR0
SO:0001217, UniRef:UniRef50_A0A810DXC6, UniRef:UniRef90_A0A8B5Y835
RefSeq:WP_014114533.1, SO:0001217, UniParc:UPI00022BB108, UniRef:UniRef100_G4NXR2, UniRef:UniRef50_A0A089M8A7, UniRef:UniRef90_G4NXR2
RefSeq:WP_014114534.1, SO:0001217, UniParc:UPI00022BB109, UniRef:UniRef100_G4NXR3, UniRef:UniRef50_A0A0D0QXG1, UniRef:UniRef90_G4NXR3
GO:0016491, RefSeq:WP_014114536.1, SO:0001217, UniParc:UPI00022BB10B, UniRef:UniRef100_G4NXR5, UniRef:UniRef50_A0A1S2MKT3, UniRef:UniRef90_A0A7G7FYL9
KEGG:K03623, RefSeq:WP_087986851.1, SO:0001217, UniParc:UPI00086D805D, UniRef:UniRef100_UPI00086D805D, UniRef:UniRef50_O07938, UniRef:UniRef90_A0A0D1KS98
SO:0001217, UniRef:UniRef50_A0A268ABP6, UniRef:UniRef90_G4NXR8
BlastRules:WP_003229881, GO:0003677, GO:0003700, KEGG:K19575, RefSeq:WP_087986844.1, SO:0001217, UniParc:UPI00086B44E4, UniRef:UniRef100_UPI00086B44E4, UniRef:UniRef50_P39842, UniRef:UniRef90_P3
BlastRules:WP_003229880, GO:0005886, GO:0042910, GO:0046677, RefSeq:WP_088679364.1, SO:0001217, UniParc:UPI00086B9F42, UniRef:UniRef100_UPI00086B9F42, UniRef:UniRef50_P39843, UniRef:UniRef90_P398
BlastRules:WP_003229879, EC:2.3.1.57, GO:0004145, GO:0046203, GO:0046208, SO:0001217, UniRef:UniRef50_P39909, UniRef:UniRef90_P39909
RefSeq:WP_072175780.1, SO:0001217, UniParc:UPI0002AB7C16, UniRef:UniRef100_A0A6D1AE24, UniRef:UniRef50_A0A2M8SZZ0, UniRef:UniRef90_A0A2M8SZZ0
COG:C, COG:COG1853, RefSeq:WP_014114549.1, SO:0001217, UniParc:UPI00022BB279, UniRef:UniRef100_G4NXS8, UniRef:UniRef50_A0A3T1D692, UniRef:UniRef90_Q65H10
RefSeq:WP_014114550.1, SO:0001217, UniParc:UPI00022BB27A, UniRef:UniRef100_G4NXS9, UniRef:UniRef50_G4NXS9, UniRef:UniRef90_G4NXS9
SO:0001217, UniRef:UniRef50_UPI00227E596D
SO:0001217, UniRef:UniRef50_G4NXT1, UniRef:UniRef90_G4NXT1
RefSeq:WP_061186629.1, SO:0001217, UniParc:UPI0006A8A65F, UniRef:UniRef100_UPI0006A8A65F, UniRef:UniRef50_A0A7I8DM31, UniRef:UniRef90_A0A9Q4HHT7
SO:0001217, UniRef:UniRef50_A0A268BQ35, UniRef:UniRef90_E0TTV8
GO:0008270, RefSeq:WP_072183443.1, SO:0001217, UniParc:UPI0006A80A48, UniRef:UniRef100_UPI0006A80A48, UniRef:UniRef50_O05410, UniRef:UniRef90_O05410
RefSeq:WP_004398671.1, SO:0001217, UniParc:UPI000195C606, UniRef:UniRef100_O07934, UniRef:UniRef50_O07934, UniRef:UniRef90_O07934
RefSeq:WP_072183444.1, SO:0001217, UniParc:UPI0006A91D94, UniRef:UniRef100_UPI0006A91D94, UniRef:UniRef50_O07934, UniRef:UniRef90_G4NXU2
GO:0030435, RefSeq:WP_061186636.1, SO:0001217, UniParc:UPI0006A884C0, UniRef:UniRef100_A0A9Q4E9L0, UniRef:UniRef50_O07919, UniRef:UniRef90_O07919
GO:0030435, RefSeq:WP_072183445.1, SO:0001217, UniParc:UPI0006A82268, UniRef:UniRef100_A0A9Q4E7M4, UniRef:UniRef50_O07949, UniRef:UniRef90_O07949
COG:COG1063, COG:ER, EC:1.-.-.-, GO:0008270, GO:0016491, SO:0001217, UniRef:UniRef50_O06012, UniRef:UniRef90_O06012
RefSeq:WP_003226012.1, SO:0001217, UniParc:UPI0001D28E43, UniRef:UniRef100_A0A9Q4HH94, UniRef:UniRef50_O06011, UniRef:UniRef90_O06011
RefSeq:WP_003226009.1, SO:0001217, UniParc:UPI0001D28E42, UniRef:UniRef100_G4NXU6, UniRef:UniRef50_G4NXU6, UniRef:UniRef90_G4NXU6
SO:0001217, UniRef:UniRef50_A0A9Q4HHT3, UniRef:UniRef90_A0A9Q4HHT3
BlastRules:WP_004398725, GO:0003677, GO:0003700, RefSeq:WP_014114566.1, SO:0001217, UniParc:UPI00022BB28A, UniRef:UniRef100_G4NXU9, UniRef:UniRef50_O06008, UniRef:UniRef90_O06008
SO:0001217, UniRef:UniRef50_G5F3I6, UniRef:UniRef90_G4NXV0
BlastRules:WP_003229837, EC:1.1.1.-, GO:0008270, GO:0016616, RefSeq:WP_072183449.1, SO:0001217, UniParc:UPI0006A9068C, UniRef:UniRef100_UPI0006A9068C, UniRef:UniRef50_C0SPA5, UniRef:UniRef90_C0SP
SO:0001217, UniRef:UniRef50_Q2NW12
SO:0001217, UniRef:UniRef50_A0A0B6AAT0
EC:3.2.1.80, KEGG:K03332, SO:0001217, UniRef:UniRef50_P05656, UniRef:UniRef90_UPI002DB653BC
BlastRules:WP_003229834, COG:COG3716, COG:G, GO:0005886, GO:0009401, KEGG:K02771, RefSeq:WP_014114571.1, SO:0001217, UniParc:UPI00022BB28F, UniRef:UniRef100_G4NXV4, UniRef:UniRef50_P26382, Un
BlastRules:WP_003229833, COG:COG3715, COG:G, GO:0005886, GO:0009401, KEGG:K11196, RefSeq:WP_014114572.1, SO:0001217, UniParc:UPI00022BB290, UniRef:UniRef100_G4NXV5, UniRef:UniRef50_P69801, Un
BlastRules:WP_003229832, EC:2.7.1.202, GO:0005737, GO:0008982, GO:0009401, GO:0016301, KEGG:K11195, RefSeq:WP_014114573.1, SO:0001217, UniParc:UPI00022BB291, UniRef:UniRef100_G4NXV6, UniRef:UniRe
BlastRules:WP_003246144, EC:2.7.1.202, GO:0005737, GO:0009401, GO:0016020, GO:0016301, GO:0016773, KEGG:K11194, RefSeq:WP_014114574.1, SO:0001217, UniParc:UPI00022BB292, UniRef:UniRef100_G4NXV7
BlastRules:WP_003229830, GO:0003677, GO:0003723, GO:0005524, GO:0009401, GO:0016020, GO:0016301, GO:0016887, GO:0045893, SO:0001217, UniRef:UniRef50_P23914, UniRef:UniRef90_P23914
RefSeq:WP_240509977.1, SO:0001217, UniParc:UPI001F0A4970, UniRef:UniRef100_UPI001F0A4970, UniRef:UniRef50_UPI001F0A4970, UniRef:UniRef90_UPI001F0A4970
GO:0005886, GO:0006865, GO:0055085, RefSeq:WP_072183452.1, SO:0001217, UniParc:UPI0006A7F3F2, UniRef:UniRef100_UPI0006A7F3F2, UniRef:UniRef50_P96704, UniRef:UniRef90_O06005
GO:0005886, GO:0006865, GO:0015171, KEGG:K05834, SO:0001217, UniRef:UniRef50_O05406, UniRef:UniRef90_O05406
RefSeq:WP_072183453.1, SO:0001217, UniParc:UPI0006A7F54B, UniRef:UniRef100_UPI0006A7F54B, UniRef:UniRef50_O05405, UniRef:UniRef90_O05405
BlastRules:WP_004398891, GO:0003677, GO:0006352, GO:0006355, GO:0016987, RefSeq:WP_072183454.1, SO:0001217, UniParc:UPI0006A89D55, UniRef:UniRef100_UPI0006A89D55, UniRef:UniRef50_O05404, UniRef:U
GO:0005886, RefSeq:WP_088679369.1, SO:0001217, UniParc:UPI0006A937AC, UniRef:UniRef100_A0A9Q4E6G5, UniRef:UniRef50_O05403, UniRef:UniRef90_O05403
KEGG:K16568, SO:0001217, UniRef:UniRef50_O05402, UniRef:UniRef90_G4NXW6
RefSeq:WP_088884223.1, SO:0001217, UniParc:UPI00086B0BD6, UniRef:UniRef100_A0A9Q4EAP1, UniRef:UniRef50_O05401, UniRef:UniRef90_E0TTY9
RFAM:RF02450, SO:0000655
SO:0001217, UniRef:UniRef50_A0A1I6SIR9
SO:0001217, UniRef:UniRef50_UPI003877D770
BlastRules:WP_009967878, GO:0000976, GO:0003700, GO:0006355, RefSeq:WP_041521019.1, SO:0001217, UniParc:UPI0005A26DC7, UniRef:UniRef100_UPI0005A26DC7, UniRef:UniRef50_O08335, UniRef:UniRef90_O0
SO:0001217, UniRef:UniRef50_O05400, UniRef:UniRef90_UPI002351DEBA
RefSeq:WP_003237348.1, SO:0001217, UniParc:UPI00022BB41D, UniRef:UniRef100_A0A2M8SZM3, UniRef:UniRef50_O32027, UniRef:UniRef90_O32027
COG:COG2116, COG:P, GO:0005886, GO:0015499, GO:0015724, SO:0001217, UniRef:UniRef50_O05399, UniRef:UniRef90_O05399
SO:0001217, UniRef:UniRef50_O05398, UniRef:UniRef90_O05398
EC:1.17.1.9, GO:0008863, GO:0009326, GO:0015942, GO:0016020, GO:0022904, GO:0043546, GO:0046872, GO:0051537, GO:0051539, SO:0001217, UniRef:UniRef50_Q795Y4, UniRef:UniRef90_Q795Y4
RefSeq:WP_003225938.1, SO:0001217, UniParc:UPI0001D28D63, UniRef:UniRef100_A0A9Q4HH85, UniRef:UniRef50_O05396, UniRef:UniRef90_O05396
EC:4.4.1.1, GO:0003962, GO:0004123, GO:0005737, GO:0008284, GO:0019343, GO:0019346, GO:0030170, GO:0043418, GO:0044540, GO:0047982, GO:0080146, KEGG:K17217, SO:0001217, UniRef:UniRef50_O05394, U
BlastRules:WP_001103859, BlastRules:WP_003229809, EC:2.5.1.134, GO:0004122, GO:0004124, GO:0005737, GO:0006535, GO:0008284, GO:0019344, SO:0001217, UniRef:UniRef50_O05393, UniRef:UniRef90_O05393
EC:3.2.2.16, EC:3.2.2.9, GO:0005829, GO:0008782, GO:0008930, GO:0009164, GO:0019284, GO:0019509, KEGG:K01243, RefSeq:WP_061186654.1, SO:0001217, UniParc:UPI0007721346, UniRef:UniRef100_A0A9Q4E6F
EC:2.1.1.-, GO:0000179, GO:0005829, GO:0031167, SO:0001217, UniRef:UniRef50_O32029, UniRef:UniRef90_O32029
SO:0001217, UniRef:UniRef50_O32030, UniRef:UniRef90_O32030
SO:0001217, UniRef:UniRef50_A0A5S9MD81
EC:3.4.16.4, GO:0005886, GO:0006508, GO:0008658, GO:0009002, GO:0009252, GO:0030435, GO:0051301, GO:0071555, GO:0071972, KEGG:K21468, SO:0001217, UniRef:UniRef50_O32032, UniRef:UniRef90_O32032
GO:0003677, GO:0006354, GO:0032784, GO:0070063, RefSeq:WP_003225918.1, SO:0001217, UniParc:UPI0001D28D5A, UniRef:UniRef100_A0A9Q4E6D4, UniRef:UniRef50_P80240, UniRef:UniRef90_P80240
COG:COG0572, COG:F, EC:2.7.1.48, GO:0004849, GO:0005524, GO:0005737, GO:0043771, GO:0044206, GO:0044211, KEGG:K00876, RefSeq:WP_003225916.1, SO:0001217, UniParc:UPI0000060810, UniRef:UniRef100_
EC:3.4.-.-, GO:0006400, GO:0006508, GO:0008233, KEGG:K08303, RefSeq:WP_072182145.1, SO:0001217, UniParc:UPI0006A845CF, UniRef:UniRef100_UPI0006A845CF, UniRef:UniRef50_O32034, UniRef:UniRef90_O3203
EC:3.4.-.-, GO:0006400, GO:0006508, GO:0008233, SO:0001217, UniRef:UniRef50_O32035, UniRef:UniRef90_O32035
EC:2.1.1.-, GO:0000287, GO:0008171, GO:0016300, GO:0030488, RefSeq:WP_014114600.1, SO:0001217, UniParc:UPI00022BB429, UniRef:UniRef100_G4NY71, UniRef:UniRef50_O32036, UniRef:UniRef90_O32036
EC:4.2.2.29, GO:0005886, GO:0008932, GO:0009252, GO:0071555, KEGG:K07082, SO:0001217, UniRef:UniRef50_O34758, UniRef:UniRef90_O34758
RefSeq:WP_003237470.1, SO:0001217, UniParc:UPI00022BB42C, UniRef:UniRef100_A0A2M8SZK8, UniRef:UniRef50_O34828, UniRef:UniRef90_O34828
EC:3.1.-.-, EC:3.1.21.10, GO:0000967, GO:0004518, GO:0005829, GO:0006364, SO:0001217, UniRef:UniRef50_O34634, UniRef:UniRef90_O34634
COG:COG4472, COG:S, RefSeq:WP_003225903.1, SO:0001217, UniParc:UPI00001B094C, UniRef:UniRef100_Q7WY61, UniRef:UniRef50_Q7WY61, UniRef:UniRef90_Q7WY61
SO:0001217, UniRef:UniRef50_S7U4X5, UniRef:UniRef90_UPI00366B62FD
SO:0001217, UniRef:UniRef50_A0A4Y1ZB53
GO:0005886, GO:0055085, RefSeq:WP_242734101.1, SO:0001217, UniParc:UPI001F612708, UniRef:UniRef100_UPI001F612708, UniRef:UniRef50_O34472, UniRef:UniRef90_O34472
EC:5.6.2.3, GO:0003677, GO:0003689, GO:0005524, GO:0005737, GO:0006310, GO:0009295, GO:0009338, GO:0009378, GO:0016853, GO:0016887, GO:0017116, GO:0036121, GO:0043139, GO:0061749, GO:0061775, G
PFAM:PF00515.34, PFAM:PF07719.23, PFAM:PF09976.15, PFAM:PF12895.13, PFAM:PF13174.12, PFAM:PF13176.12, PFAM:PF13181.12, PFAM:PF13371.12, PFAM:PF13374.12, PFAM:PF13414.12, PFAM:PF13424.12, PFA
EC:2.8.1.13, GO:0000049, GO:0002143, GO:0005524, GO:0005737, GO:0008033, GO:0016740, GO:0016783, GO:0103016, KEGG:K00566, SO:0001217, UniRef:UniRef50_Q812R6, UniRef:UniRef90_O35020
EC:2.8.1.7, GO:0016226, GO:0031071, GO:0046872, GO:0051536, KEGG:K04487, SO:0001217, UniRef:UniRef50_O34599, UniRef:UniRef90_O34599
BlastRules:WP_003225879, COG:COG1959, COG:K, GO:0001046, GO:0003700, GO:0005829, GO:0006355, GO:0032991, GO:0032993, GO:0042802, GO:0042803, GO:0051291, SO:0001217, UniRef:UniRef50_O34527, Un
GO:0000731, GO:0003677, GO:0005524, GO:0006261, GO:0006310, GO:0008047, GO:0009378, GO:0016887, GO:0017116, SO:0001217, UniRef:UniRef50_O34528, UniRef:UniRef90_O34528
EC:6.1.-.-, GO:0005524, GO:0008641, GO:0061503, GO:0061504, RefSeq:WP_061186668.1, SO:0001217, UniParc:UPI000772A971, UniRef:UniRef100_UPI000772A971, UniRef:UniRef50_O32037, UniRef:UniRef90_O32037
RFAM:RF00013, SO:0000655
EC:6.1.1.-, EC:6.1.1.23, GO:0003676, GO:0004815, GO:0005524, GO:0005737, GO:0006422, GO:0016740, GO:0050560, SO:0001217, UniRef:UniRef50_Q81LI7, UniRef:UniRef90_O32038
EC:6.1.1.12, GO:0000166, GO:0004812, GO:0005524, GO:0006418, GO:0016874, SO:0001217, UniRef:UniRef50_A0A938G708, UniRef:UniRef90_UPI00227ED0FF
EC:6.1.1.21, GO:0004821, GO:0005524, GO:0005737, GO:0006427, GO:0016740, SO:0001217, UniRef:UniRef50_O32039, UniRef:UniRef90_O32039
RFAM:RF00230, SO:0000140
RefSeq:WP_003222701.1, SO:0001217, UniParc:UPI0001D288FA, UniRef:UniRef100_A0A9Q4HC75, UniRef:UniRef50_O32040, UniRef:UniRef90_O32040
SO:0001217, UniRef:UniRef50_UPI0022815D45
PFAM:PF01520.23
EC:3.1.1.96, GO:0000049, GO:0004045, GO:0005737, GO:0006399, GO:0008152, GO:0019478, GO:0043908, GO:0051500, GO:0106026, KEGG:K07560, RefSeq:WP_072183431.1, SO:0001217, UniParc:UPI0006A84C35, U
SO:0001217, UniRef:UniRef50_A0A971RJV7, UniRef:UniRef90_UPI00030D195C
EC:2.7.6.5, EC:3.1.7.2, KEGG:K01139, SO:0001217, UniRef:UniRef50_A0A098B0Y6, UniRef:UniRef90_UPI00167C4502
SO:0001217, UniRef:UniRef50_A0A7C6Y4K1
COG:COG0503, COG:F, EC:2.4.2.7, GO:0002055, GO:0003999, GO:0005737, GO:0006166, GO:0006168, GO:0009116, GO:0016208, GO:0044209, RefSeq:WP_014114618.1, SO:0001217, UniParc:UPI00022BB5BE, UniRef:
EC:3.1.-.-, GO:0003677, GO:0005737, GO:0006281, GO:0006310, GO:0008409, GO:0009295, KEGG:K07462, SO:0001217, UniRef:UniRef50_O32044, UniRef:UniRef90_O32044
SO:0001217, UniRef:UniRef50_O32045, UniRef:UniRef90_A0A9Q4DTU4
SO:0001217, UniRef:UniRef50_O07535, UniRef:UniRef90_G4NY99
GO:0005886, GO:0006605, GO:0006886, GO:0015031, GO:0015450, GO:0043952, GO:0065002, KEGG:K12257, SO:0001217, UniRef:UniRef50_O32047, UniRef:UniRef90_O32047
BlastRules:WP_004398498, GO:0005737, GO:0030420, RefSeq:WP_072183429.1, SO:0001217, UniParc:UPI0006A8F0B6, UniRef:UniRef100_UPI0006A8F0B6, UniRef:UniRef50_O32049, UniRef:UniRef90_O32049
GO:0005886, GO:0030435, GO:0030436, KEGG:K06409, RefSeq:WP_072183428.1, SO:0001217, UniParc:UPI0006A8D986, UniRef:UniRef100_UPI0006A8D986, UniRef:UniRef50_Q00758, UniRef:UniRef90_Q00758
GO:0005886, RefSeq:WP_014114625.1, SO:0001217, UniParc:UPI00022BB5C5, UniRef:UniRef100_G4NYA3, UniRef:UniRef50_O32050, UniRef:UniRef90_O32050
GO:0005886, SO:0001217, UniParc:UPI00022BB5C6, UniRef:UniRef100_G4NYA4, UniRef:UniRef50_O32051, UniRef:UniRef90_O32051
GO:0005886, GO:0009306, GO:0015031, GO:0015450, KEGG:K03210, RefSeq:WP_003222677.1, SO:0001217, UniParc:UPI0001D288ED, UniRef:UniRef100_A0A9Q4DRR4, UniRef:UniRef50_O32052, UniRef:UniRef90_O320
COG:COG0343, COG:J, EC:2.4.2.29, GO:0002099, GO:0005737, GO:0005829, GO:0006400, GO:0008479, GO:0008616, GO:0046872, GO:0101030, KEGG:K00773, RefSeq:WP_003237529.1, SO:0001217, UniParc:UPI0002
SO:0001217, UniRef:UniRef50_A0A380FJR9
GO:0005886, SO:0001217, UniRef:UniRef50_C0H463, UniRef:UniRef90_C0H463
EC:3.6.4.-, EC:3.6.4.12, GO:0000400, GO:0003677, GO:0003689, GO:0005524, GO:0005737, GO:0006281, GO:0006310, GO:0009378, GO:0016887, GO:0036121, GO:0048476, GO:0061749, GO:0061775, GO:0140584, GO
EC:3.6.4.12, GO:0000400, GO:0003678, GO:0005524, GO:0005737, GO:0006281, GO:0006310, GO:0009378, GO:0009379, GO:0036121, GO:0048476, GO:0061749, GO:1990518, SO:0001217, UniRef:UniRef50_O05392, U
BlastRules:WP_003229715, GO:0030435, KEGG:K06318, RefSeq:WP_014114631.1, SO:0001217, UniParc:UPI00022BB5CC, UniRef:UniRef100_G4NYB1, UniRef:UniRef50_O05391, UniRef:UniRef90_O05391
GO:0005886, GO:0022857, SO:0001217, UniRef:UniRef50_O05390, UniRef:UniRef90_O05390
SO:0001217, UniRef:UniRef50_UPI002B21A7DA, UniRef:UniRef90_UPI002B21A7DA
PFAM:PF02894.22, PFAM:PF22725.1
SO:0001217, UniRef:UniRef50_A0A8D3X3A4
EC:2.7.11.1, GO:0004676, GO:0004677, GO:0004679, GO:0004711, GO:0005524, GO:0035175, GO:0035402, GO:0035403, GO:0035979, GO:0044022, GO:0044023, GO:0044024, GO:0044025, GO:0072354, GO:0072371,
RefSeq:WP_014114635.1, SO:0001217, UniParc:UPI00022BB5D0, UniRef:UniRef100_G4NYB5, UniRef:UniRef50_L8EAY3, UniRef:UniRef90_G4NYB5
GO:0005283, GO:0005886, KEGG:K03310, RefSeq:WP_014114636.1, SO:0001217, UniParc:UPI00022BB5D1, UniRef:UniRef100_G4NYB6, UniRef:UniRef50_O32060, UniRef:UniRef90_O32060
GO:0003677, GO:0005829, GO:0006355, RefSeq:WP_072183433.1, SO:0001217, UniParc:UPI0006A931C0, UniRef:UniRef100_UPI0006A931C0, UniRef:UniRef50_Q8Y6Z5, UniRef:UniRef90_P94447
GO:0030435, GO:0043595, RefSeq:WP_041521024.1, SO:0001217, UniParc:UPI0002DE0DDC, UniRef:UniRef100_G4NYB8, UniRef:UniRef50_P94446, UniRef:UniRef90_P94446
BlastRules:WP_003229695, GO:0008932, GO:0030435, GO:0043595, GO:0051117, RefSeq:WP_087986778.1, SO:0001217, UniParc:UPI000B431814, UniRef:UniRef100_UPI000B431814, UniRef:UniRef50_O32062, UniRef:U
EC:2.5.1.72, GO:0005829, GO:0008987, GO:0009435, GO:0034628, GO:0046872, GO:0051539, KEGG:K03517, SO:0001217, UniRef:UniRef50_Q9KWZ1, UniRef:UniRef90_Q9KWZ1
EC:2.4.2.19, GO:0004514, GO:0005737, GO:0009435, GO:0034213, KEGG:K00767, RefSeq:WP_041521025.1, SO:0001217, UniParc:UPI0005A1EFFE, UniRef:UniRef100_G4NYC1, UniRef:UniRef50_P39666, UniRef:UniRef9
EC:1.4.3.16, GO:0008734, GO:0009435, KEGG:K00278, SO:0001217, UniRef:UniRef50_P38032, UniRef:UniRef90_G4NYC2
EC:2.8.1.7, GO:0031071, GO:0046872, GO:0051536, KEGG:K04487, SO:0001217, UniRef:UniRef50_P38033, UniRef:UniRef90_P38033
GO:0003677, GO:0036094, SO:0001217, UniRef:UniRef50_P39667, UniRef:UniRef90_P39667
SO:0001217, UniRef:UniRef50_A0A5S9MHW4
SO:0001217, UniRef:UniRef50_UPI00232F5F4D
BlastRules:WP_003222630, EC:5.4.99.5, KEGG:K06209, RefSeq:WP_003222630.1, SO:0001217, UniParc:UPI0000060845, UniRef:UniRef100_P21204, UniRef:UniRef50_P21204, UniRef:UniRef90_P21204

EC:3.6.5.-, GO:0000287, GO:0003924, GO:0005525, GO:0005737, GO:0042254, SO:0001217, UniRef:UniRef50_P20964, UniRef:UniRef90_P20964
BlastRules:WP_004399131, EC:2.7.-.-, GO:0000155, GO:0005737, GO:0030435, KEGG:K06375, RefSeq:WP_072183417.1, SO:0001217, UniParc:UPI0006A8387C, UniRef:UniRef100_A0A9Q4E5I9, UniRef:UniRef50_P06535
GO:0000315, GO:0003735, GO:0006412, GO:0022625, KEGG:K02899, RefSeq:WP_003222623.1, SO:0001217, UniParc:UPI0000050EFE, UniRef:UniRef100_P05657, UniRef:UniRef50_P05657, UniRef:UniRef90_P05657
EC:3.4.22.-, GO:0006508, GO:0008234, GO:0042254, KEGG:K07584, RefSeq:WP_061186689.1, SO:0001217, UniParc:UPI0006A94089, UniRef:UniRef100_A0A9Q4E6K1, UniRef:UniRef50_P26942, UniRef:UniRef90_P26942
COG:COG0261, COG:J, GO:0003723, GO:0003735, GO:0005737, GO:0005840, GO:0006412, GO:0019843, GO:1990904, KEGG:K02888, RefSeq:WP_003229668.1, SO:0001217, UniParc:UPI0000060849, UniRef:UniRef100
RFAM:RF00559, SO:0000140
BlastRules:WP_000599071, BlastRules:WP_004398649, EC:3.4.24.-, GO:0006508, GO:0008237, GO:0016020, GO:0030435, GO:0046872, KEGG:K06402, RefSeq:WP_072183416.1, SO:0001217, UniParc:UPI0006A89A9E, U
BlastRules:WP_000797461, BlastRules:WP_004398684, GO:0004222, GO:0016020, GO:0030435, KEGG:K06401, RefSeq:WP_014114652.1, SO:0001217, UniParc:UPI00022BB739, UniRef:UniRef100_G4NYD4, UniRef:UniR
GO:0000917, GO:0000918, GO:0005524, GO:0005829, GO:0009898, GO:0016887, RefSeq:WP_014114654.1, SO:0001217, UniParc:UPI00022BB73B, UniRef:UniRef100_G4NYD6, UniRef:UniRef50_Q01464, UniRef:UniRef9
GO:0000902, GO:0000917, GO:0000918, GO:0004857, GO:0032272, GO:1901891, RefSeq:WP_003222613.1, SO:0001217, UniParc:UPI0001D2880E, UniRef:UniRef100_A0A9Q4HH62, UniRef:UniRef50_Q01463, UniRef:Uni
GO:0005886, GO:0008360, GO:0016020, RefSeq:WP_014114655.1, SO:0001217, UniParc:UPI00022BB73C, UniRef:UniRef100_G4NYD8, UniRef:UniRef50_Q01467, UniRef:UniRef90_Q01467
GO:0005886, GO:0008360, GO:0016504, KEGG:K03570, RefSeq:WP_003222609.1, SO:0001217, UniParc:UPI0001CE3CEB, UniRef:UniRef100_G4NYD9, UniRef:UniRef50_Q01466, UniRef:UniRef90_Q01466
BlastRules:WP_000466737, BlastRules:WP_000913396, BlastRules:WP_003094393, BlastRules:WP_003229650, RefSeq:WP_014114656.1, SO:0001217, UniParc:UPI00022BB73D, UniRef:UniRef100_G4NYE0, UniRef:UniRe
GO:0003677, GO:0006281, GO:0006508, GO:0008237, GO:0046872, KEGG:K03630, RefSeq:WP_041521028.1, SO:0001217, UniParc:UPI0002E74D71, UniRef:UniRef100_UPI0002E74D71, UniRef:UniRef50_Q02170, UniRef
EC:3.6.1.9, GO:0005737, GO:0009117, GO:0036218, GO:0036221, GO:0047429, GO:0106379, RefSeq:WP_014114658.1, SO:0001217, UniParc:UPI00022BB73F, UniRef:UniRef100_G4NYE2, UniRef:UniRef50_Q02169, UniR
KEGG:K06380, SO:0001217, UniRef:UniRef50_P37575, UniRef:UniRef90_UPI002091423A
GO:0004190, GO:0005886, GO:0006465, GO:0008168, GO:0030420, GO:0030435, GO:0032259, GO:0046872, RefSeq:WP_061186692.1, SO:0001217, UniParc:UPI000772412C, UniRef:UniRef100_UPI000772412C, UniRef:
PFAM:PF02875.26
PFAM:PF08245.18
SO:0001217, UniRef:UniRef50_W4VHF7
SO:0001217, UniRef:UniRef50_A0A7X6PKX6
SO:0001217, UniRef:UniRef50_A0A5S9MHH6
PFAM:PF00133.28
RFAM:RF00230, SO:0000140
GO:0030436, GO:0042601, SO:0001217, UniRef:UniRef50_P37964, UniRef:UniRef90_P37964
GO:0030436, GO:0042601, SO:0001217, UniRef:UniRef50_P37964, UniRef:UniRef90_P37964
GO:0030436, RefSeq:WP_230939517.1, SO:0001217, UniParc:UPI001E313A05, UniRef:UniRef100_UPI002DDD82EF, UniRef:UniRef50_A0A2M8SZC6, UniRef:UniRef90_A0A2M8SZC6
PFAM:PF20918.2
SO:0001217, UniRef:UniRef50_Q3B1A1
EC:4.2.1.24, GO:0003824, GO:0004655, GO:0005829, GO:0006782, GO:0006783, GO:0008270, GO:0033014, GO:0046872, KEGG:K01698, RefSeq:WP_014114668.1, SO:0001217, UniParc:UPI00022BB749, UniRef:UniRef1
BlastRules:WP_003229629, EC:4.2.1.75, GO:0004852, GO:0006780, GO:0006782, RefSeq:WP_072183582.1, SO:0001217, UniParc:UPI0006A927D1, UniRef:UniRef100_UPI0006A927D1, UniRef:UniRef50_P21248, UniRef:U
EC:2.5.1.61, GO:0004418, GO:0005737, GO:0006782, GO:0006783, GO:0033014, KEGG:K01749, RefSeq:WP_072183583.1, SO:0001217, UniParc:UPI0006A8F5E5, UniRef:UniRef100_UPI0006A8F5E5, UniRef:UniRef50_P1
GO:0005886, GO:0006779, GO:0017004, GO:0020037, KEGG:K02497, RefSeq:WP_003222575.1, SO:0001217, UniParc:UPI000195C663, UniRef:UniRef100_P16645, UniRef:UniRef50_P16645, UniRef:UniRef90_P16645
EC:1.2.1.70, GO:0006782, GO:0008883, GO:0050661, KEGG:K02492, SO:0001217, UniRef:UniRef50_Q65GJ9, UniRef:UniRef90_P16618
GO:0005886, RefSeq:WP_014114672.1, SO:0001217, UniParc:UPI00022BB74D, UniRef:UniRef100_G4NYN8, UniRef:UniRef50_P40736, UniRef:UniRef90_P40736
GO:0000917, GO:0003924, GO:0005525, GO:0005737, GO:0005829, GO:0042254, GO:0043022, GO:0046872, RefSeq:WP_014114673.1, SO:0001217, UniParc:UPI00022BB74E, UniRef:UniRef100_G4NYN9, UniRef:UniRef5
EC:3.4.21.53, GO:0004176, GO:0004252, GO:0005524, GO:0005737, GO:0006508, GO:0006515, GO:0016887, GO:0034605, GO:0043565, KEGG:K01338, SO:0001217, UniRef:UniRef50_P37945, UniRef:UniRef90_P37945
EC:3.4.21.53, GO:0004176, GO:0004252, GO:0005524, GO:0005737, GO:0006508, GO:0016887, GO:0030163, GO:0030436, KEGG:K04076, RefSeq:WP_072566639.1, SO:0001217, UniParc:UPI00090B3476, UniRef:UniRef
EC:3.4.21.92, GO:0005524, GO:0009368, GO:0051082, GO:0140662, RefSeq:WP_003222561.1, SO:0001217, UniParc:UPI0001D28735, UniRef:UniRef100_A0A9Q4E2W2, UniRef:UniRef50_P50866, UniRef:UniRef90_A0A9Q
EC:5.2.1.8, GO:0003755, GO:0005854, GO:0015031, GO:0051082, GO:0051083, SO:0001217, UniRef:UniRef50_P80698, UniRef:UniRef90_UPI000B53887E
BlastRules:WP_003229609, RefSeq:WP_088679384.1, SO:0001217, UniParc:UPI000B52D74C, UniRef:UniRef100_UPI000B52D74C, UniRef:UniRef50_P94569, UniRef:UniRef90_P94569
SO:0001217, UniRef:UniRef50_Q67MJ3, UniRef:UniRef90_A0A8I2B5L1
SO:0001217, UniRef:UniRef50_UPI002282B285
EC:1.1.1.85, GO:0000287, GO:0003862, GO:0005829, GO:0009098, GO:0051287, KEGG:K00052, SO:0001217, UniRef:UniRef50_P05645, UniRef:UniRef90_P05645
SO:0001217, UniRef:UniRef50_A0A5S9MGL5
PFAM:PF22617.1
SO:0001217, UniRef:UniRef50_A0A4Y5ZP02
COG:COG0059, COG:EH, EC:1.1.1.86, GO:0000287, GO:0004455, GO:0005829, GO:0009097, GO:0009099, GO:0050661, KEGG:K00053, SO:0001217, UniRef:UniRef50_Q0AV19, UniRef:UniRef90_P37253
EC:2.2.1.6, GO:0003984, GO:0005829, GO:0005948, GO:0009082, GO:0009097, GO:0009099, GO:1990610, KEGG:K01653, RefSeq:WP_003222543.1, SO:0001217, UniParc:UPI0001D2872E, UniRef:UniRef100_G4NYQ1, U
EC:2.2.1.6, GO:0000287, GO:0003984, GO:0005948, GO:0009097, GO:0009099, GO:0030976, GO:0050660, KEGG:K01652, SO:0001217, UniRef:UniRef50_P37251, UniRef:UniRef90_P37251
RFAM:RF00230, SO:0000140
RefSeq:WP_014114685.1, SO:0001217, UniParc:UPI00022BB8BB, UniRef:UniRef100_G4NYQ3, UniRef:UniRef50_P94563, UniRef:UniRef90_A0A2M8SZA2
EC:2.3.1.-, GO:0016747, RefSeq:WP_072182699.1, SO:0001217, UniParc:UPI0006A8CFFE, UniRef:UniRef100_UPI0006A8CFFE, UniRef:UniRef50_P94562, UniRef:UniRef90_P94562
SO:0001217, UniRef:UniRef50_P94560, UniRef:UniRef90_A0A9W5LKB8
SO:0001036
EC:3.1.4.-, GO:0016787, GO:0046872, KEGG:K07095, RefSeq:WP_072566647.1, SO:0001217, UniParc:UPI00090ABD9D, UniRef:UniRef100_A0A9Q4E192, UniRef:UniRef50_P94559, UniRef:UniRef90_P94559
EC:3.6.1.66, GO:0000166, GO:0005737, GO:0005829, GO:0009117, GO:0009143, GO:0009146, GO:0017111, GO:0035870, GO:0036220, GO:0036222, GO:0046872, GO:0047429, KEGG:K01519, SO:0001217, UniRef:UniRe
BlastRules:WP_001261764, BlastRules:WP_003246039, EC:2.7.7.56, GO:0000049, GO:0000175, GO:0006364, GO:0008033, GO:0009022, GO:0016075, KEGG:K00989, RefSeq:WP_088679387.1, SO:0001217, UniParc:UPI0
BlastRules:WP_001126757, BlastRules:WP_003229578, GO:0030435, KEGG:K06298, RefSeq:WP_061186715.1, SO:0001217, UniParc:UPI0006A8A3B9, UniRef:UniRef100_A0A9Q4E2B7, UniRef:UniRef50_P39072, UniRef:U
EC:5.1.1.3, GO:0008152, GO:0008360, GO:0008881, GO:0009252, GO:0071555, SO:0001217, UniParc:UPI00022BB8C1, UniRef:UniRef100_G4NYR0, UniRef:UniRef50_Q836J0, UniRef:UniRef90_P94556
GO:0003677, GO:0003700, GO:0006355, GO:0006950, RefSeq:WP_014114692.1, SO:0001217, UniParc:UPI00022BB8C2, UniRef:UniRef100_G4NYR1, UniRef:UniRef50_P97247, UniRef:UniRef90_P97247
BlastRules:WP_000659484, BlastRules:WP_003184172, COG:COG5905, COG:DK, GO:0003677, GO:0006355, RefSeq:WP_003184172.1, SO:0001217, UniParc:UPI0000111783, UniRef:UniRef100_P11470, UniRef:UniRef50_
EC:3.1.2.-, KEGG:K07107, SO:0001217, UniRef:UniRef50_P11469, UniRef:UniRef90_A0A9W5PDX8
EC:1.3.5.1, GO:0006099, GO:0008177, GO:0009055, GO:0009060, GO:0016491, GO:0022904, GO:0046872, GO:0051536, GO:0051537, GO:0051538, GO:0051539, RefSeq:WP_003222517.1, SO:0001217, UniParc:UPI0001
COG:C, COG:COG1053, EC:1.3.5.1, GO:0000104, GO:0005886, GO:0006099, GO:0008177, GO:0009055, GO:0009061, GO:0045121, GO:0050660, RefSeq:WP_041338046.1, SO:0001217, UniParc:UPI00059DB1E9, UniRef
BlastRules:WP_000678351, BlastRules:WP_003222512, GO:0005886, GO:0006099, GO:0046872, RefSeq:WP_003222512.1, SO:0001217, UniParc:UPI0000060876, UniRef:UniRef100_P08064, UniRef:UniRef50_P08064, Uni
RefSeq:WP_014114695.1, SO:0001217, UniParc:UPI00022BB8C5, UniRef:UniRef100_G4NYR7, UniRef:UniRef50_P42955, UniRef:UniRef90_P42955
EC:2.7.2.4, GO:0004072, GO:0005524, GO:0005829, GO:0009088, GO:0009089, GO:0009090, GO:0019877, SO:0001217, UniRef:UniRef50_P08495, UniRef:UniRef90_P08495
RFAM:RF00168, SO:0000035
GO:0003677, GO:0005737, GO:0006289, GO:0009380, GO:0009381, GO:0009432, KEGG:K03703, RefSeq:WP_114151369.1, SO:0001217, UniParc:UPI000DEEF2A1, UniRef:UniRef100_UPI000DEEF2A1, UniRef:UniRef50_C
COG:COG3118, COG:O, GO:0005737, GO:0005829, GO:0015035, GO:0045454, RefSeq:WP_003222500.1, SO:0001217, UniParc:UPI000006087B, UniRef:UniRef100_P14949, UniRef:UniRef50_P14949, UniRef:UniRef90_P1
BlastRules:WP_004398654, EC:3.2.1.55, GO:0000272, GO:0005737, GO:0031222, GO:0046373, GO:0046556, KEGG:K01209, SO:0001217, UniRef:UniRef50_P94552, UniRef:UniRef90_P94552
SO:0001217, UniRef:UniRef50_A0A9W5LKC5
PFAM:PF00766.25, PFAM:PF01012.27
SO:0001217, UniRef:UniRef50_UPI003519F187, UniRef:UniRef90_UPI003519F187
BlastRules:WP_001029032, BlastRules:WP_004398765, GO:0009055, KEGG:K03521, SO:0001217, UniRef:UniRef50_P94550, UniRef:UniRef90_P94550
EC:4.2.1.17, GO:0004300, GO:0006635, SO:0001217, UniRef:UniRef50_P94549, UniRef:UniRef90_P94549
BlastRules:WP_003229547, GO:0000976, GO:0003700, GO:0005737, GO:0006355, GO:0006631, GO:0016042, RefSeq:WP_003222489.1, SO:0001217, UniParc:UPI0001D2863A, UniRef:UniRef100_A0A9Q4DR28, UniRef:Ur
BlastRules:WP_004399166, EC:6.2.1.3, GO:0004467, GO:0005524, RefSeq:WP_072182848.1, SO:0001217, UniParc:UPI0006A88BAE, UniRef:UniRef100_UPI0006A88BAE, UniRef:UniRef50_P94547, UniRef:UniRef90_P9454
SO:0001217, UniRef:UniRef50_UPI0031F9E1C7
PFAM:PF03994.20
GO:0003690, GO:0004519, GO:0005524, GO:0005737, GO:0006259, GO:0006298, GO:0016887, GO:0019843, GO:0030983, GO:0043023, GO:0045910, GO:0072344, GO:0140664, KEGG:K07456, RefSeq:WP_072182847.1
GO:0003677, GO:0003887, GO:0006260, GO:0006281, GO:0008310, GO:0046872, SO:0001217, UniRef:UniRef50_P94544, UniRef:UniRef90_P94544
GO:0005886, GO:0009403, RefSeq:WP_003222477.1, SO:0001217, UniParc:UPI0001D28635, UniRef:UniRef100_A0A9Q4H9V5, UniRef:UniRef50_P94543, UniRef:UniRef90_P94543
GO:0000917, GO:0000921, GO:0005829, GO:0005886, GO:0030428, GO:0032153, GO:0043093, GO:0051301, RefSeq:WP_003237682.1, SO:0001217, UniParc:UPI00022BB8D1, UniRef:UniRef100_A0A2M8SZ73, UniRef:Ur
EC:3.1.26.4, GO:0000287, GO:0003723, GO:0004523, GO:0005737, GO:0006298, GO:0006401, GO:0032299, GO:0043137, KEGG:K03471, RefSeq:WP_061186725.1, SO:0001217, UniParc:UPI0006A8565B, UniRef:UniRef1
PFAM:PF03147.19
PFAM:PF17759.6
EC:6.1.1.20, GO:0000049, GO:0000287, GO:0004826, GO:0005524, GO:0006432, GO:0009328, GO:0016740, KEGG:K01890, SO:0001217, UniRef:UniRef50_P17922, UniRef:UniRef90_P17922
EC:6.1.1.20, GO:0000049, GO:0000166, GO:0000287, GO:0004826, GO:0005524, GO:0005737, GO:0006432, GO:0016740, KEGG:K01889, RefSeq:WP_014114709.1, SO:0001217, UniParc:UPI00022BBA6A, UniRef:UniRef1
EC:2.1.1.-, GO:0003723, GO:0005737, GO:0006364, GO:0008173, GO:0032259, RefSeq:WP_014114710.1, SO:0001217, UniParc:UPI00022BBA6B, UniRef:UniRef100_G4NYT7, UniRef:UniRef50_P94538, UniRef:UniRef90_P
GO:0030435, GO:0030436, KEGG:K06426, RefSeq:WP_003222464.1, SO:0001217, UniParc:UPI0001D2862F, UniRef:UniRef100_A0A9Q4E169, UniRef:UniRef50_P94537, UniRef:UniRef90_P94537
SO:0001217, UniRef:UniRef50_UPI002415F226, UniRef:UniRef90_UPI002415F226
PFAM:PF13556.12
PFAM:PF01565.28
SO:0001217, UniRef:UniRef50_UPI002DDD53ED
EC:1.1.99.14, GO:0005886, GO:0019154, GO:0046872, GO:0047809, GO:0051539, KEGG:K11473, SO:0001217, UniRef:UniRef50_P94534, UniRef:UniRef90_P94534
SO:0001217, UniParc:UPI00022BBA6F, UniRef:UniRef100_G4NYU2, UniRef:UniRef50_E0TVZ5, UniRef:UniRef90_E0TVZ5
BlastRules:WP_003229512, GO:0005886, GO:0009267, GO:0015031, GO:0015833, GO:0031669, KEGG:K06200, SO:0001217, UniRef:UniRef50_P94532, UniRef:UniRef90_P94532
SO:0001217, UniRef:UniRef50_A0A2M8SZA1, UniRef:UniRef90_E0TVZ7
RefSeq:WP_061186733.1, SO:0001217, UniParc:UPI0006A81CC7, UniRef:UniRef100_A0A9Q4E430, UniRef:UniRef50_G4NYU5, UniRef:UniRef90_G4NYU5
SO:0001217, UniParc:UPI00022BBA73, UniRef:UniRef100_G4NYU6, UniRef:UniRef50_R2QUL7, UniRef:UniRef90_G4NYU6
BlastRules:WP_004398747, EC:3.2.1.55, GO:0000272, GO:0005737, GO:0031222, GO:0046373, GO:0046556, SO:0001217, UniRef:UniRef50_P94531, UniRef:UniRef90_P94531
BlastRules:WP_003229508, GO:0005886, GO:0055085, KEGG:K17236, RefSeq:WP_003222443.1, SO:0001217, UniParc:UPI0001D28624, UniRef:UniRef100_A0A9Q4HEV6, UniRef:UniRef50_P94530, UniRef:UniRef90_P945
GO:0005886, GO:0055085, KEGG:K17235, RefSeq:WP_072182835.1, SO:0001217, UniParc:UPI0006A862C3, UniRef:UniRef100_UPI0006A862C3, UniRef:UniRef50_P94529, UniRef:UniRef90_P94529
GO:0005886, KEGG:K17234, SO:0001217, UniRef:UniRef50_P94528, UniRef:UniRef90_P94528
BlastRules:WP_003229504, EC:1.1.1.261, GO:0003856, GO:0005737, GO:0006650, GO:0008654, GO:0046872, GO:0106357, GO:0106358, KEGG:K00096, RefSeq:WP_072182833.1, SO:0001217, UniParc:UPI0006A91A05,
BlastRules:WP_010886590, EC:3.1.3.23, GO:0005737, GO:0016791, GO:0036424, GO:0046872, GO:0050308, KEGG:K02101, SO:0001217, UniParc:UPI00022BBA76, UniRef:UniRef100_G4NYV2, UniRef:UniRef50_P94526,
EC:5.1.3.4, GO:0005829, GO:0008270, GO:0008742, GO:0016832, GO:0019323, GO:0019568, GO:0019569, RefSeq:WP_003222433.1, SO:0001217, UniParc:UPI0001D2861F, UniRef:UniRef100_A0A9Q4E2S3, UniRef:UniRe
EC:2.7.1.16, GO:0005524, GO:0005737, GO:0008741, GO:0019150, GO:0019321, GO:0019568, GO:0019569, RefSeq:WP_061186737.1, SO:0001217, UniParc:UPI0006A940CF, UniRef:UniRef100_UPI0006A940CF, UniRef:U
EC:5.3.1.4, GO:0005829, GO:0005996, GO:0008733, GO:0019569, GO:0030145, KEGG:K01804, SO:0001217, UniRef:UniRef50_P94523, UniRef:UniRef90_P94523
EC:3.2.1.99, KEGG:K06113, SO:0001217, UniRef:UniRef50_P94522, UniRef:UniRef90_D6RV88
EC:3.4.11.-, GO:0004177, GO:0006508, GO:0008237, GO:0046872, RefSeq:WP_072182831.1, SO:0001217, UniParc:UPI0006A8F2CF, UniRef:UniRef100_UPI0006A8F2CF, UniRef:UniRef50_P94521, UniRef:UniRef90_P9452
BlastRules:WP_003229493, GO:0016020, RefSeq:WP_003222423.1, SO:0001217, UniParc:UPI0001D28553, UniRef:UniRef100_G4NYV9, UniRef:UniRef50_P94520, UniRef:UniRef90_P94520
GO:0005886, RefSeq:WP_003222422.1, SO:0001217, UniParc:UPI0001D28552, UniRef:UniRef100_G4NYW0, UniRef:UniRef50_P94519, UniRef:UniRef90_P94519
COG:COG0292, COG:J, GO:0000027, GO:0000315, GO:0003735, GO:0005840, GO:0006412, GO:0019843, GO:0022625, GO:1990904, RefSeq:WP_014114727.1, SO:0001217, UniParc:UPI00022BBA7C, UniRef:UniRef100_
COG:COG0290, COG:J, GO:0003743, GO:0005829, GO:0006413, GO:0032790, GO:0043022, SO:0001217, UniParc:UPI00022BBA7D, UniRef:UniRef100_G4NZ43, UniRef:UniRef50_Q81L15, UniRef:UniRef90_P55872
RFAM:RF00558, SO:0000140
RefSeq:WP_090558205.1, SO:0001217, UniParc:UPI000868F61C, UniRef:UniRef100_UPI000868F61C, UniRef:UniRef50_P94517, UniRef:UniRef90_A0A9Q4DQH2
SO:0001217, UniRef:UniRef50_A0A5S9MD62
GO:0005886, GO:0012501, GO:0019835, GO:0031640, KEGG:K05339, SO:0001217, UniRef:UniRef50_P94516, UniRef:UniRef90_P94516
GO:0005886, GO:0012501, GO:0019835, GO:0031640, KEGG:K05338, RefSeq:WP_014114732.1, SO:0001217, UniParc:UPI00022BBA81, UniRef:UniRef100_G4NZ47, UniRef:UniRef50_P94515, UniRef:UniRef90_P94515
BlastRules:WP_003229479, GO:0000156, GO:0000976, GO:0005829, GO:0006355, GO:0032993, RefSeq:WP_072182748.1, SO:0001217, UniParc:UPI0006A8D6F0, UniRef:UniRef100_UPI0006A8D6F0, UniRef:UniRef50_P9
BlastRules:WP_000933570, BlastRules:WP_003229477, EC:2.7.13.3, GO:0000155, GO:0005524, GO:0005886, GO:0071555, KEGG:K07704, SO:0001217, UniRef:UniRef50_P94513, UniRef:UniRef90_P94513
EC:3.1.3.3, GO:0006564, GO:0016311, GO:0016791, GO:0036424, KEGG:K25528, RefSeq:WP_061186744.1, SO:0001217, UniParc:UPI0007725568, UniRef:UniRef100_A0A9Q4E572, UniRef:UniRef50_P94512, UniRe
SO:0001217, UniRef:UniRef50_A0A380EL26
SO:0001217, UniRef:UniRef50_A0A9D1HXV7
GO:0030436, SO:0001217, UniRef:UniRef50_P06569, UniRef:UniRef90_P06569
GO:0005886, SO:0001217, UniRef:UniRef50_P06568, UniRef:UniRef90_P06568
EC:3.6.4.-, GO:0003677, GO:0005524, GO:0006260, GO:0006269, GO:0016887, GO:0046872, GO:1990077, RefSeq:WP_014114738.1, SO:0001217, UniParc:UPI00022BBC1C, UniRef:UniRef100_G4NZ53, UniRef:UniRef50_

BlastRules:WP_003229464, GO:0003677, GO:0005524, GO:0006260, GO:0006269, GO:1990077, KEGG:K03346, RefSeq:WP_014114739.1, SO:0001217, UniParc:UPI00022BBC1D, UniRef:UniRef100_G4NZ54, UniRef:UniR
GO:0003677, GO:0005524, GO:0006355, GO:0008270, GO:0045892, GO:0140110, RefSeq:WP_003223586.1, SO:0001217, UniParc:UPI00000608AD, UniRef:UniRef100_Q45549, UniRef:UniRef50_Q5M5X8, UniRef:UniRef90
COG:COG1586, COG:E, EC:4.1.1.50, GO:0004014, GO:0005829, GO:0008295, RefSeq:WP_003223584.1, SO:0001217, UniParc:UPI00018E18CB, UniRef:UniRef100_O34426, UniRef:UniRef50_O34426, UniRef:UniRef90_O3
EC:1.2.1.59, GO:0004365, GO:0005737, GO:0006006, GO:0006094, GO:0016620, GO:0043891, GO:0047100, GO:0050661, GO:0051287, RefSeq:WP_003223582.1, SO:0001217, UniParc:UPI0001D28470, UniRef:UniRef100
GO:0003677, RefSeq:WP_014114741.1, SO:0001217, UniParc:UPI00022BBC1F, UniRef:UniRef100_G4NZ58, UniRef:UniRef50_O34533, UniRef:UniRef90_O34533
GO:0005886, GO:0022857, GO:0055085, SO:0001217, UniRef:UniRef50_O34367, UniRef:UniRef90_O34367
SO:0001217, UniRef:UniRef50_O34678
EC:2.7.1.24, GO:0004140, GO:0005524, GO:0005737, GO:0015937, KEGG:K00859, SO:0001217, UniRef:UniRef50_O34932, UniRef:UniRef90_O34932
GO:0005886, GO:0016020, GO:0030435, GO:0030436, RefSeq:WP_014114745.1, SO:0001217, UniParc:UPI00022BBC23, UniRef:UniRef100_A0A9Q4E139, UniRef:UniRef50_C0SP79, UniRef:UniRef90_C0SP79
EC:3.2.2.23, GO:0003684, GO:0003906, GO:0005737, GO:0006281, GO:0006284, GO:0008270, GO:0008534, GO:0034039, GO:0046872, GO:0140078, SO:0001217, UniRef:UniRef50_O34403, UniRef:UniRef90_O34403
EC:2.7.7.7, GO:0003676, GO:0003677, GO:0003887, GO:0006261, GO:0006302, GO:0008408, GO:0008409, SO:0001217, UniRef:UniRef50_O34996, UniRef:UniRef90_O34996
BlastRules:WP_001032128, BlastRules:WP_003916717, BlastRules:WP_004398493, EC:2.7.13.3, GO:0000155, GO:0000160, GO:0005524, GO:0005886, GO:0006355, GO:0006817, GO:0009927, GO:0016310, GO:0045121,
BlastRules:WP_001065226, BlastRules:WP_003229442, BlastRules:WP_003403867, GO:0000156, GO:0000976, GO:0005829, GO:0006355, GO:0006817, GO:0032993, RefSeq:WP_003223561.1, SO:0001217, UniParc:UPI0
COG:C, COG:COG0039, EC:1.1.1.37, GO:0004459, GO:0005975, GO:0006089, GO:0006090, GO:0006099, GO:0016616, GO:0030060, RefSeq:WP_003223559.1, SO:0001217, UniParc:UPI0001D283A4, UniRef:UniRef100_A
COG:C, COG:COG0538, EC:1.1.1.42, GO:0000287, GO:0004450, GO:0006097, GO:0006099, GO:0051287, KEGG:K00031, RefSeq:WP_003223557.1, SO:0001217, UniParc:UPI0001D283A3, UniRef:UniRef100_A0A9Q4DSR
EC:2.3.3.1, EC:2.3.3.16, GO:0004108, GO:0005737, GO:0005975, GO:0006099, GO:0046912, KEGG:K01659, RefSeq:WP_014114751.1, SO:0001217, UniParc:UPI00022BBC29, UniRef:UniRef100_G4NZ71, UniRef:UniRef50
GO:0005886, RefSeq:WP_014114752.1, SO:0001217, UniParc:UPI00022BBC2A, UniRef:UniRef100_G4NZ72, UniRef:UniRef50_B7GGT5, UniRef:UniRef90_O34811
GO:0005886, GO:0016020, GO:0030436, GO:0055085, RefSeq:WP_072182597.1, SO:0001217, UniParc:UPI0006A8A30F, UniRef:UniRef100_UPI0006A8A30F, UniRef:UniRef50_O34991, UniRef:UniRef90_O34991
GO:0005886, KEGG:K07113, RefSeq:WP_003237784.1, SO:0001217, UniParc:UPI00022BBC2D, UniRef:UniRef100_A0A9W5LKI2, UniRef:UniRef50_O32064, UniRef:UniRef90_O32064
EC:2.7.1.40, GO:0000287, GO:0004743, GO:0005524, GO:0005737, GO:0005829, GO:0006096, GO:0016301, GO:0030955, KEGG:K00873, RefSeq:WP_041521039.1, SO:0001217, UniParc:UPI0005A2196B, UniRef:UniRef1
COG:COG0205, COG:G, EC:2.7.1.11, GO:0003872, GO:0005524, GO:0005945, GO:0006002, GO:0006096, GO:0016208, GO:0030388, GO:0042802, GO:0046872, GO:0048029, GO:0061621, GO:0070095, KEGG:K00850, F
EC:2.1.3.15, EC:6.4.1.2, GO:0003989, GO:0005524, GO:0006633, GO:0009317, GO:0016743, GO:0046677, GO:2001295, SO:0001217, UniRef:UniRef50_O34847, UniRef:UniRef90_O34847
EC:2.1.3.15, EC:6.4.1.2, GO:0003989, GO:0005524, GO:0006633, GO:0008270, GO:0009317, GO:0016743, GO:2001295, RefSeq:WP_014114757.1, SO:0001217, UniParc:UPI00022BBC30, UniRef:UniRef100_G4NZ79, UniF
BlastRules:WP_003229415, SO:0001217, UniRef:UniRef50_A0A2U3KL65
PFAM:PF01336.31
EC:2.7.7.7, GO:0003676, GO:0003887, GO:0005737, GO:0006260, GO:0008408, GO:0009295, KEGG:K02337, SO:0001217, UniRef:UniRef50_O34623, UniRef:UniRef90_O34623
BlastRules:WP_003229410, GO:0016020, GO:0030435, RefSeq:WP_003223537.1, SO:0001217, UniParc:UPI0001D28399, UniRef:UniRef100_A0A2M8SZ12, UniRef:UniRef50_C0H3P8, UniRef:UniRef90_C0H3P8
BlastRules:WP_003229408, GO:0005886, RefSeq:WP_003223535.1, SO:0001217, UniParc:UPI0001D28398, UniRef:UniRef100_G4NZ83, UniRef:UniRef50_O34460, UniRef:UniRef90_O34460
SO:0001217, UniRef:UniRef50_C0H3P9, UniRef:UniRef90_C0H3P9
SO:0001217, UniRef:UniRef50_W4VIK7
GO:0005886, RefSeq:WP_003223529.1, SO:0001217, UniParc:UPI0001D28395, UniRef:UniRef100_A0A9Q4DSR0, UniRef:UniRef50_O34922, UniRef:UniRef90_O34922
RefSeq:WP_014114761.1, SO:0001217, UniParc:UPI00022BBC34, UniRef:UniRef100_G4NZ88, UniRef:UniRef50_O34921, UniRef:UniRef90_O34921
GO:0016787, RefSeq:WP_014114762.1, SO:0001217, UniParc:UPI00022BBC35, UniRef:UniRef100_G4NZ89, UniRef:UniRef50_Q795U4, UniRef:UniRef90_Q795U4
EC:1.-.-.-, GO:0016616, GO:0030497, RefSeq:WP_061186759.1, SO:0001217, UniParc:UPI0006A89FC3, UniRef:UniRef100_A0A9Q4ESN7, UniRef:UniRef50_O34308, UniRef:UniRef90_O34308
RefSeq:WP_003223517.1, SO:0001217, UniParc:UPI0001D28391, UniRef:UniRef100_G4NZ92, UniRef:UniRef50_G4NZ92, UniRef:UniRef90_G4NZ92
EC:4.3.2.1, GO:0004056, GO:0005829, GO:0006526, GO:0042450, KEGG:K01755, SO:0001217, UniRef:UniRef50_O34858, UniRef:UniRef90_O34858
SO:0001217, UniRef:UniRef50_A0A5S9MH99, UniRef:UniRef90_A0A5S9MH99
PFAM:PF20979.2
SO:0001217, UniRef:UniRef50_A0RJM4
GO:0005829, GO:0006777, SO:0001217, UniRef:UniRef50_O34457, UniRef:UniRef90_O34457
SO:0001217, UniRef:UniRef50_W7YDX4
SO:0001217, UniRef:UniRef50_A0A2X3EN22
GO:0003677, GO:0008170, RefSeq:WP_072182021.1, SO:0001217, UniParc:UPI0006A925FD, UniRef:UniRef100_UPI0006A925FD, UniRef:UniRef50_P37876, UniRef:UniRef90_P37876
PFAM:PF08534.16
EC:1.11.1.-, EC:1.11.1.24, GO:0008379, GO:0016684, KEGG:K11065, SO:0001217, UniRef:UniRef50_P80864, UniRef:UniRef90_P80864
RefSeq:WP_014114771.1, SO:0001217, UniParc:UPI00022BA46A, UniRef:UniRef100_G4NZA1, UniRef:UniRef50_O34917, UniRef:UniRef90_O34917
GO:0005886, RefSeq:WP_072182020.1, SO:0001217, UniParc:UPI0006A8EA43, UniRef:UniRef100_UPI0006A8EA43, UniRef:UniRef50_O34424, UniRef:UniRef90_O34424
EC:3.4.21.-, GO:0005886, GO:0006508, GO:0008236, GO:0045121, KEGG:K04773, RefSeq:WP_014114773.1, SO:0001217, UniParc:UPI00022BA46C, UniRef:UniRef100_G4NZA3, UniRef:UniRef50_O34525, UniRef:UniRef9
SO:0001217, UniRef:UniRef50_W7B4R2
EC:3.5.-.-, GO:0016810, SO:0001217, UniRef:UniRef50_O34355, UniRef:UniRef90_O34355
EC:6.2.1.-, GO:0004321, GO:0005524, GO:0006633, GO:0006637, GO:0015645, GO:0016405, SO:0001217, UniRef:UniRef50_C0SPB0, UniRef:UniRef90_C0SPB0
GO:0003690, GO:0006265, GO:0030435, RefSeq:WP_014114778.1, SO:0001217, UniParc:UPI00022BA471, UniRef:UniRef100_A0A9Q4EM71, UniRef:UniRef50_P84584, UniRef:UniRef90_P04831
EC:2.8.1.4, GO:0000049, GO:0002937, GO:0004810, GO:0005524, GO:0005829, GO:0009228, GO:0009229, GO:0052837, GO:0140741, KEGG:K03151, SO:0001217, UniRef:UniRef50_O34595, UniRef:UniRef90_O34595
EC:2.8.1.7, GO:0031071, GO:0046872, GO:0051536, KEGG:K04487, RefSeq:WP_088679937.1, SO:0001217, UniParc:UPI000B52E377, UniRef:UniRef100_UPI000B52E377, UniRef:UniRef50_O34874, UniRef:UniRef90_O348
GO:0015658, GO:0015803, GO:0016020, KEGG:K03311, RefSeq:WP_061186769.1, SO:0001217, UniParc:UPI0006A894B4, UniRef:UniRef100_UPI0006A894B4, UniRef:UniRef50_O34545, UniRef:UniRef90_A0A9Q4HAR9
GO:0000917, GO:0000921, GO:0005886, GO:0005940, GO:0045121, GO:0051781, KEGG:K06286, RefSeq:WP_087987942.1, SO:0001217, UniParc:UPI000B436870, UniRef:UniRef100_UPI000B436870, UniRef:UniRef50_O
EC:3.1.3.15, GO:0000105, GO:0004401, KEGG:K04486, RefSeq:WP_014114783.1, SO:0001217, UniParc:UPI00022BA476, UniRef:UniRef100_G4NZB3, UniRef:UniRef50_O34411, UniRef:UniRef90_O34411
BlastRules:WP_003229313, GO:0000976, GO:0003700, GO:0006355, RefSeq:WP_003223478.1, SO:0001217, UniParc:UPI0001D282BD, UniRef:UniRef100_G4NZB4, UniRef:UniRef50_O34970, UniRef:UniRef90_O34970
GO:0005829, GO:0033745, RefSeq:WP_014114784.1, SO:0001217, UniParc:UPI00022BA477, UniRef:UniRef100_G4NZB5, UniRef:UniRef50_O34553, UniRef:UniRef90_O34553
GO:0005886, GO:0043709, GO:0052621, GO:1902201, SO:0001217, UniRef:UniRef50_O34325, UniRef:UniRef90_O34325
SO:0001217, UniRef:UniRef50_UPI0023B06F61
KEGG:K02986, SO:0001217, UniRef:UniRef50_A0AAW9J461, UniRef:UniRef90_UPI00203B58A3
RefSeq:WP_014114787.1, SO:0001217, UniParc:UPI00022BA47A, UniRef:UniRef100_G4NZB9, UniRef:UniRef50_A0AAT9J3C2, UniRef:UniRef90_A0AAT9J3C2
PFAM:PF01479.30, PFAM:PF22421.1
SO:0001217, UniRef:UniRef50_A0A3D1A0P8
RFAM:RF00230, SO:0000140
EC:6.2.1.1, GO:0003987, GO:0005524, GO:0005829, GO:0005886, GO:0006085, GO:0045121, GO:0046872, SO:0001217, UniRef:UniRef50_P39062, UniRef:UniRef90_P39062
BlastRules:WP_000581406, BlastRules:WP_003229296, EC:2.3.1.-, GO:0009847, GO:0019152, GO:0030435, GO:0034078, GO:0043894, GO:0045014, GO:0045150, GO:0071311, KEGG:K04766, RefSeq:WP_014114790.1,
GO:0030435, GO:0045150, SO:0001217, UniRef:UniRef50_P39066, UniRef:UniRef90_P39066
GO:0004407, GO:0030435, GO:0040029, GO:0045150, RefSeq:WP_041521040.1, SO:0001217, UniParc:UPI0005A01CF1, UniRef:UniRef100_G4NZJ2, UniRef:UniRef50_P39067, UniRef:UniRef90_P39067
GO:0005886, SO:0001217, UniRef:UniRef50_P39064, UniRef:UniRef90_P39064
GO:0005886, GO:0006935, GO:0071978, RefSeq:WP_061186798.1, SO:0001217, UniParc:UPI0007726D46, UniRef:UniRef100_A0A9Q4E2L7, UniRef:UniRef50_P46826, UniRef:UniRef90_P39063
GO:0000976, GO:0001216, GO:0001217, GO:0001228, GO:0003677, GO:0003700, GO:0005975, GO:0006355, GO:0032993, GO:0045892, GO:0045893, SO:0001217, UniRef:UniRef50_P25144, UniRef:UniRef90_P25144
SO:0001217, UniRef:UniRef50_A0A9W5E899
SO:0001217, UniRef:UniRef50_A0A5S9MHI0
GO:0006979, GO:0016491, GO:0019899, GO:0045454, GO:0061770, KEGG:K26959, RefSeq:WP_014114796.1, SO:0001217, UniParc:UPI00022BA483, UniRef:UniRef100_G4NZJ7, UniRef:UniRef50_P39914, UniRef:UniRef9
SO:0001217, UniRef:UniRef50_P40780, UniRef:UniRef90_UPI0022827C97
GO:0016020, RefSeq:WP_014114798.1, SO:0001217, UniParc:UPI00022BA485, UniRef:UniRef100_G4NZJ9, UniRef:UniRef50_P40779, UniRef:UniRef90_P40779
EC:6.3.2.8, GO:0005524, GO:0005737, GO:0008360, GO:0008763, GO:0009252, GO:0051301, GO:0071555, RefSeq:WP_014114799.1, SO:0001217, UniParc:UPI00022BA5E7, UniRef:UniRef100_G4NZK0, UniRef:UniRef50_
SO:0001217, UniRef:UniRef50_A0A917XR65
GO:0000049, SO:0001217, UniRef:UniRef50_O34943, UniRef:UniRef90_O34943
RefSeq:WP_003223439.1, SO:0001217, UniParc:UPI0001D282AA, UniRef:UniRef100_A0A9Q4E218, UniRef:UniRef50_Q812N6, UniRef:UniRef90_O34496
RefSeq:WP_003237915.1, SO:0001217, UniParc:UPI0002AC1515, UniRef:UniRef100_A0A9Q4E083, UniRef:UniRef50_O34357, UniRef:UniRef90_O34357
SO:0001217, UniRef:UniRef50_O34305, UniRef:UniRef90_O34305
EC:3.4.11.-, GO:0004177, GO:0006508, GO:0008237, GO:0046872, RefSeq:WP_061186783.1, SO:0001217, UniParc:UPI0006A8C7C0, UniRef:UniRef100_UPI0006A8C7C0, UniRef:UniRef50_O34924, UniRef:UniRef90_O349
SO:0001217, UniRef:UniRef50_O32065, UniRef:UniRef90_O32065
EC:1.1.1.38, GO:0004470, GO:0004471, GO:0006090, GO:0006108, GO:0008948, GO:0046872, GO:0051287, RefSeq:WP_014114806.1, SO:0001217, UniParc:UPI00022BA5EF, UniRef:UniRef100_G4NZK9, UniRef:UniRef50
SO:0001217, UniRef:UniRef50_W7BUT2
SO:0001217, UniRef:UniRef50_W7CA11
EC:2.1.1.33, GO:0006400, GO:0008176, RefSeq:WP_014114808.1, SO:0001217, UniParc:UPI00022BA5F1, UniRef:UniRef100_G4NZL1, UniRef:UniRef50_O34522, UniRef:UniRef90_G4NZL1
RefSeq:WP_003237926.1, SO:0001217, UniParc:UPI0000060905, UniRef:UniRef100_O32066, UniRef:UniRef50_O32066, UniRef:UniRef90_O32066
EC:2.7.1.15, EC:2.7.1.229, GO:0004747, GO:0005524, GO:0005737, GO:0006270, GO:0006974, GO:0016773, GO:0019200, GO:0051301, RefSeq:WP_003223423.1, SO:0001217, UniParc:UPI0001D29015, UniRef:UniRef10
EC:3.2.1.41, GO:0051060, KEGG:K01200, RefSeq:WP_072182004.1, SO:0001217, UniParc:UPI0006A86031, UniRef:UniRef100_UPI0006A86031, UniRef:UniRef50_C0SPA0, UniRef:UniRef90_G4NZL4
EC:2.7.1.-, GO:0004143, GO:0005524, GO:0006665, GO:0008654, GO:0016020, GO:0046872, SO:0001217, UniRef:UniRef50_O34799, UniRef:UniRef90_O34799
SO:0001217, UniRef:UniRef50_UPI0035E5746F
SO:0001217, UniRef:UniRef50_O34471, UniRef:UniRef90_O34471
EC:3.1.4.58, GO:0004113, GO:0005829, GO:0008033, GO:0008664, GO:0010738, GO:0034237, RefSeq:WP_061186786.1, SO:0001217, UniParc:UPI0006A91FA6, UniRef:UniRef100_UPI0006A91FA6, UniRef:UniRef50_O345
SO:0001217, UniRef:UniRef50_A0AA87C9R0
SO:0001217, UniRef:UniRef50_W7Y607
SO:0001217, UniRef:UniRef50_O34944
BlastRules:WP_003229234, GO:0005345, GO:0005886, KEGG:K06901, RefSeq:WP_041521042.1, SO:0001217, UniParc:UPI0002F74732, UniRef:UniRef100_G4NZM0, UniRef:UniRef50_O34978, UniRef:UniRef90_O34978
SO:0001217, UniRef:UniRef50_O35029, UniRef:UniRef90_A0A9Q4E194
EC:7.-.-.-, GO:0005524, GO:0016887, SO:0001217, UniRef:UniRef50_O34977, UniRef:UniRef90_O34977
COG:COG1349, COG:KG, GO:0003677, GO:0003700, RefSeq:WP_003152337.1, SO:0001217, UniParc:UPI0000060910, UniRef:UniRef100_O32067, UniRef:UniRef50_O32067, UniRef:UniRef90_O32067
EC:5.4.99.-, GO:0000455, GO:0003723, GO:0120159, RefSeq:WP_014114819.1, SO:0001217, UniParc:UPI00022BA5FC, UniRef:UniRef100_G4NZM4, UniRef:UniRef50_O32068, UniRef:UniRef90_O32068
BlastRules:WP_003229224, GO:0005886, GO:0008360, GO:0009252, GO:0051301, GO:0071555, SO:0001217, UniRef:UniRef50_O34674, UniRef:UniRef90_O34674
SO:0001217, UniRef:UniRef50_A0A0J5GN56
GO:0005886, GO:0022857, GO:0031460, KEGG:K02168, SO:0001217, UniRef:UniRef50_P54417, UniRef:UniRef90_P54417
GO:0030435, RefSeq:WP_072181999.1, SO:0001217, UniParc:UPI0006A871BB, UniRef:UniRef100_UPI0006A871BB, UniRef:UniRef50_P94496, UniRef:UniRef90_P94496
SO:0001217, UniRef:UniRef50_O34378, UniRef:UniRef90_A0A2M8SYS6
SO:0001217, UniRef:UniRef50_D4G087
PFAM:PF02894.22
PFAM:PF02894.22
SO:0001217, UniRef:UniRef50_D4G088, UniRef:UniRef90_D4G088
BlastRules:WP_014906487, EC:3.2.1.172, GO:0005737, GO:0005975, GO:0102211, KEGG:K15532, SO:0001217, UniRef:UniRef50_O34559, UniRef:UniRef90_O34559
GO:0005886, GO:0055085, RefSeq:WP_014114828.1, SO:0001217, UniParc:UPI00022BA605, UniRef:UniRef100_G4NZN4, UniRef:UniRef50_C0SPB3, UniRef:UniRef90_C0SPB3
SO:0001217, UniRef:UniRef50_D4G092, UniRef:UniRef90_D4G092
SO:0001217, UniRef:UniRef50_UPI0022824969
GO:0005886, KEGG:K25673, RefSeq:WP_014114830.1, SO:0001217, UniParc:UPI00022BA77B, UniRef:UniRef100_G4NZN6, UniRef:UniRef50_Q795R2, UniRef:UniRef90_Q795R2
SO:0001217, UniRef:UniRef50_P53561
GO:0008460, RefSeq:WP_014114833.1, SO:0001217, UniParc:UPI00022BA77E, UniRef:UniRef100_G4NZN9, UniRef:UniRef50_P53560, UniRef:UniRef90_P53560
EC:1.14.14.46, KEGG:K16593, SO:0001217, UniRef:UniRef50_P53554, UniRef:UniRef90_A0A9Q4HB98
SO:0001217, UniRef:UniRef50_I3E3F3
EC:2.8.1.6, GO:0004076, GO:0005506, GO:0009102, GO:0051536, GO:0051537, GO:0051539, GO:1904047, KEGG:K01012, SO:0001217, UniRef:UniRef50_Q8KZM7, UniRef:UniRef90_Q8KZM7
EC:6.3.3.3, GO:0000287, GO:0004141, GO:0005524, GO:0009102, KEGG:K01935, RefSeq:WP_072181991.1, SO:0001217, UniParc:UPI0006A81D0A, UniRef:UniRef100_UPI0006A81D0A, UniRef:UniRef50_P53558, UniRef:U
EC:2.3.1.47, GO:0008710, GO:0009102, GO:0030170, RefSeq:WP_072181990.1, SO:0001217, UniParc:UPI0006A862FA, UniRef:UniRef100_UPI0006A862FA, UniRef:UniRef50_P53556, UniRef:UniRef90_P53556
EC:2.6.1.105, EC:2.6.1.62, GO:0004015, GO:0005737, GO:0009102, GO:0030170, KEGG:K19563, SO:0001217, UniRef:UniRef50_P53555, UniRef:UniRef90_P53555
EC:6.2.1.14, GO:0000287, GO:0005524, GO:0009102, GO:0042410, KEGG:K01906, SO:0001217, UniRef:UniRef50_P53559, UniRef:UniRef90_P53559
SO:0001217, UniRef:UniRef50_A0A0U1NY48, UniRef:UniRef90_G4NZP6
SO:0001217, UniRef:UniRef50_O34973, UniRef:UniRef90_A0A9Q4DTU8
SO:0001217, UniRef:UniRef50_O34829, UniRef:UniRef90_A0A6H0WNJ2
GO:0005886, GO:0015774, RefSeq:WP_072181984.1, SO:0001217, UniParc:UPI0006A83F46, UniRef:UniRef100_UPI0006A83F46, UniRef:UniRef50_O34335, UniRef:UniRef90_O34335
GO:0005886, GO:0015774, GO:0055085, RefSeq:WP_090558263.1, SO:0001217, UniParc:UPI00086A14B3, UniRef:UniRef100_UPI00086A14B3, UniRef:UniRef50_O34706, UniRef:UniRef90_O34706

GO:0005886, GO:0015774, GO:0055085, RefSeq:WP_090558264.1, SO:0001217, UniParc:UPI00086E32FA, UniRef:UniRef100_UPI00086E32FA, UniRef:UniRef50_O34518, UniRef:UniRef90_O34518
BlastRules:WP_003246000, EC:3.2.1.22, GO:0004557, GO:0005737, GO:0005975, GO:0016616, GO:0046872, KEGG:K07406, RefSeq:WP_090558266.1, SO:0001217, UniParc:UPI00086A3915, UniRef:UniRef100_UPI00086
IS:ISBsu1, SO:0001217, UniParc:UPI00022BA78C, UniRef:UniRef100_G4NZQ3, UniRef:UniRef50_A0A2T6KCC6, UniRef:UniRef90_A0A7W4LYJ7
GO:0004803, IS:ISBsu1, SO:0001217, UniRef:UniRef50_R2RXM0, UniRef:UniRef90_UPI0022831251
RFAM:RF01458, SO:0000655
EC:2.8.1.-, GO:0016740, RefSeq:WP_014114849.1, SO:0001217, UniParc:UPI00022BA78E, UniRef:UniRef100_G4NZQ5, UniRef:UniRef50_O32072, UniRef:UniRef90_O32072
EC:6.1.1.4, GO:0000166, GO:0002161, GO:0004823, GO:0005524, GO:0005829, GO:0006429, KEGG:K01869, SO:0001217, UniRef:UniRef50_A4IRY3, UniRef:UniRef90_P36430
RFAM:RF00230, SO:0000140
GO:0005886, SO:0001217, UniRef:UniRef50_O34881, UniRef:UniRef90_O34881
BlastRules:WP_003723977, BlastRules:WP_004399022, GO:0009881, RefSeq:WP_014114852.1, SO:0001217, UniParc:UPI00022BA791, UniRef:UniRef100_G4NZQ8, UniRef:UniRef50_O34627, UniRef:UniRef90_O34627
GO:0005886, GO:0022857, RefSeq:WP_072181979.1, SO:0001217, UniParc:UPI0006A804B7, UniRef:UniRef100_A0A9Q4HG47, UniRef:UniRef50_O34546, UniRef:UniRef90_O34546
SO:0001217, UniRef:UniRef50_Q795Q5
SO:0001217, UniRef:UniRef50_A0A5S9MF26
SO:0001217, UniRef:UniRef50_V9GAG3
BlastRules:WP_003229137, GO:0005524, GO:0005886, GO:0016887, GO:0022857, GO:0046677, GO:0055085, RefSeq:WP_003223342.1, SO:0001217, UniParc:UPI0001D28F0A, UniRef:UniRef100_E0TY60, UniRef:UniRef5
BlastRules:WP_004398652, EC:2.7.13.3, GO:0000155, GO:0005524, GO:0005886, KEGG:K11629, SO:0001217, UniRef:UniRef50_O35044, UniRef:UniRef90_O35044
BlastRules:WP_004399109, GO:0000156, GO:0000976, GO:0005829, GO:0006355, GO:0032993, KEGG:K11630, SO:0001217, UniRef:UniRef50_Q2G0E0, UniRef:UniRef90_O34951
BlastRules:WP_004398682, GO:0005886, GO:0022857, KEGG:K16918, RefSeq:WP_014114859.1, SO:0001217, UniParc:UPI00022BA798, UniRef:UniRef100_G4NZR5, UniRef:UniRef50_O35005, UniRef:UniRef90_O35005
GO:0005524, GO:0005886, GO:0016887, GO:0022857, GO:0055085, RefSeq:WP_014114860.1, SO:0001217, UniParc:UPI00022BA799, UniRef:UniRef100_G4NZR6, UniRef:UniRef50_O34392, UniRef:UniRef90_O34392
SO:0001217, UniRef:UniRef50_O34898, UniRef:UniRef90_A0A9W5LKV6
BlastRules:WP_003246175, SO:0001217, UniRef:UniRef50_O34898, UniRef:UniRef90_A0A2M8SYP6
GO:0005524, GO:0005886, GO:0016887, RefSeq:WP_014114863.1, SO:0001217, UniParc:UPI00022BA79C, UniRef:UniRef100_G4NZR9, UniRef:UniRef50_O34641, UniRef:UniRef90_O34641
GO:0003677, GO:0003700, KEGG:K07979, RefSeq:WP_003223328.1, SO:0001217, UniParc:UPI0001D28F02, UniRef:UniRef100_A0A9Q4E162, UniRef:UniRef50_O34712, UniRef:UniRef90_O34712
RefSeq:WP_014114864.1, SO:0001217, UniParc:UPI00022BA79D, UniRef:UniRef100_G4NZS1, UniRef:UniRef50_L8ECR1, UniRef:UniRef90_L8ECR1
RefSeq:WP_003223324.1, SO:0001217, UniParc:UPI0001D28F00, UniRef:UniRef100_A0A9Q4E3R5, UniRef:UniRef50_O32073, UniRef:UniRef90_O32073
GO:0003824, GO:0046872, GO:0051536, GO:0051539, GO:1904047, KEGG:K07139, RefSeq:WP_072181974.1, SO:0001217, UniParc:UPI0006A92A4B, UniRef:UniRef100_UPI0006A92A4B, UniRef:UniRef50_O35008, UniRe
EC:2.1.1.61, GO:0004808, GO:0008033, GO:0032259, RefSeq:WP_014114866.1, SO:0001217, UniParc:UPI00022BA90C, UniRef:UniRef100_G4NZS4, UniRef:UniRef50_O34614, UniRef:UniRef90_O34614
BlastRules:WP_004399076, EC:4.2.3.130, GO:0016829, KEGG:K16188, SO:0001217, UniRef:UniRef50_O34707, UniRef:UniRef90_O34707
BlastRules:WP_004398545, EC:3.1.1.-, GO:0016020, GO:0016298, GO:0017000, RefSeq:WP_041521047.1, SO:0001217, UniParc:UPI0005A2AB9A, UniRef:UniRef100_A0A9Q4HK82, UniRef:UniRef50_O34705, UniRef:UniRe
EC:2.-.-.-, GO:0016740, KEGG:K01726, RefSeq:WP_041905866.1, SO:0001217, UniParc:UPI00050766BA, UniRef:UniRef100_A0A9Q4DPU6, UniRef:UniRef50_Q5KW03, UniRef:UniRef90_O34696
GO:0003333, GO:0005886, GO:0015171, GO:0016020, RefSeq:WP_079288138.1, SO:0001217, UniParc:UPI0009A2858F, UniRef:UniRef100_UPI0009A2858F, UniRef:UniRef50_O34618, UniRef:UniRef90_O34618
SO:0001217, UniRef:UniRef50_W4VKY0
EC:6.3.5.4, GO:0004066, GO:0005524, GO:0006529, GO:0070981, SO:0001217, UniRef:UniRef50_P54420, UniRef:UniRef90_P54420
SO:0001217, UniRef:UniRef50_A0A5S9MHT8
COG:COG0192, COG:H, EC:2.5.1.6, GO:0000287, GO:0004478, GO:0005524, GO:0005737, GO:0006556, GO:0006730, KEGG:K00789, SO:0001217, UniRef:UniRef50_P54419, UniRef:UniRef90_P54419
RFAM:RF00162, SO:0000140
EC:4.1.1.49, GO:0004612, GO:0005524, GO:0005829, GO:0006094, GO:0046872, KEGG:K01610, SO:0001217, UniRef:UniRef50_P54418, UniRef:UniRef90_P54418
RefSeq:WP_014114873.1, SO:0001217, UniParc:UPI00022BA913, UniRef:UniRef100_G4NZT2, UniRef:UniRef50_O34365, UniRef:UniRef90_O34365
EC:3.4.21.-, GO:0004252, GO:0006508, RefSeq:WP_072181972.1, SO:0001217, UniParc:UPI0006A93044, UniRef:UniRef100_UPI0006A93044, UniRef:UniRef50_O34493, UniRef:UniRef90_O34493
GO:0005886, GO:0009970, SO:0001217, UniRef:UniRef50_C0SP84, UniRef:UniRef90_C0SP84
EC:7.-.-.-, GO:0005524, GO:0016887, RefSeq:WP_327816326.1, SO:0001217, UniParc:UPI002E20912A, UniRef:UniRef100_UPI002E20912A, UniRef:UniRef50_O34314, UniRef:UniRef90_O34314
SO:0001217, UniRef:UniRef50_A0A5S9MG50
EC:3.6.1.55, GO:0005737, GO:0006139, GO:0016818, GO:0017111, GO:0035539, GO:0046872, RefSeq:WP_014114878.1, SO:0001217, UniParc:UPI00022BA918, UniRef:UniRef100_A0A9Q4E3Q3, UniRef:UniRef50_O3501
GO:0005886, RefSeq:WP_090558270.1, SO:0001217, UniParc:UPI00086D5739, UniRef:UniRef100_UPI00086D5739, UniRef:UniRef50_O34883, UniRef:UniRef90_O34883
GO:0008199, GO:0016722, KEGG:K04047, RefSeq:WP_003219364.1, SO:0001217, UniParc:UPI0001D28D87, UniRef:UniRef100_A0A9Q4HEH8, UniRef:UniRef50_Q8RPQ2, UniRef:UniRef90_P80879
RefSeq:WP_003219363.1, SO:0001217, UniParc:UPI0001D28D86, UniRef:UniRef100_A0A9Q4H9M2, UniRef:UniRef50_A0A0U2ITM2, UniRef:UniRef90_A0A0D1LC19
SO:0001217, UniRef:UniRef50_P40768, UniRef:UniRef90_A0AAP3FVT8
EC:4.4.1.21, GO:0005506, GO:0009372, GO:0043768, RefSeq:WP_014114882.1, SO:0001217, UniParc:UPI00022BA91C, UniRef:UniRef100_G4NZU3, UniRef:UniRef50_O34667, UniRef:UniRef90_A7Z800
EC:4.2.1.1, GO:0004089, GO:0008270, KEGG:K01673, RefSeq:WP_061187207.1, SO:0001217, UniParc:UPI0006A871B4, UniRef:UniRef100_UPI0006A871B4, UniRef:UniRef50_O34872, UniRef:UniRef90_O34872
GO:0003735, GO:0005840, GO:0006412, GO:1990904, RefSeq:WP_003219346.1, SO:0001217, UniParc:UPI0000060951, UniRef:UniRef100_O34967, UniRef:UniRef50_O34967, UniRef:UniRef90_O34967
EC:7.1.1.7, KEGG:K00425, RefSeq:WP_014114886.1, SO:0001217, UniParc:UPI00022BA920, UniRef:UniRef100_G4NZU8, UniRef:UniRef50_C0SP90, UniRef:UniRef90_A0A0D1L5A6
EC:7.1.1.7, GO:0005886, GO:0030435, KEGG:K00426, RefSeq:WP_003219339.1, SO:0001217, UniParc:UPI0001D28D81, UniRef:UniRef100_E0TY97, UniRef:UniRef50_O34505, UniRef:UniRef90_O34505
RefSeq:WP_003219337.1, SO:0001217, UniParc:UPI0001D28D80, UniRef:UniRef100_A0A9Q4E022, UniRef:UniRef50_C0H3Q2, UniRef:UniRef90_C0H3Q2
GO:0005886, GO:0006811, GO:0010043, GO:0043190, GO:0055085, KEGG:K11709, SO:0001217, UniRef:UniRef50_O34500, UniRef:UniRef90_O34500
KEGG:K11708, SO:0001217, UniRef:UniRef50_O35024, UniRef:UniRef90_G4NZV2
BlastRules:WP_004398644, GO:0005524, GO:0006811, GO:0016887, GO:0042626, GO:0043190, RefSeq:WP_014114890.1, SO:0001217, UniParc:UPI00022BA924, UniRef:UniRef100_G4NZV3, UniRef:UniRef50_O34338, U
BlastRules:WP_000669594, BlastRules:WP_003229060, GO:0005886, GO:0007155, GO:0030001, GO:0045121, GO:0046872, KEGG:K11707, RefSeq:WP_014114891.1, SO:0001217, UniParc:UPI00022BA925, UniRef:UniRe
EC:4.2.1.113, GO:0000287, GO:0009234, GO:0016829, GO:0043748, KEGG:K02549, RefSeq:WP_087987971.1, SO:0001217, UniParc:UPI000B42D0FA, UniRef:UniRef100_UPI000B42D0FA, UniRef:UniRef50_A0A0P0ZBS7,
EC:6.2.1.26, GO:0005524, GO:0008756, GO:0009234, GO:0016405, RefSeq:WP_327816324.1, SO:0001217, UniParc:UPI002E1EBA88, UniRef:UniRef100_UPI002E1EBA88, UniRef:UniRef50_P23971, UniRef:UniRef90_P239
COG:COG0447, COG:H, EC:4.1.3.36, GO:0005829, GO:0008935, GO:0009234, GO:0071890, KEGG:K01661, RefSeq:WP_010329489.1, SO:0001217, UniParc:UPI00022BA928, UniRef:UniRef100_G4NZV7, UniRef:UniRef50_
EC:4.2.99.20, GO:0009234, GO:0016020, GO:0070205, RefSeq:WP_088679904.1, SO:0001217, UniParc:UPI000B535AAA, UniRef:UniRef100_UPI000B535AAA, UniRef:UniRef50_P23974, UniRef:UniRef90_P23974
EC:2.2.1.9, GO:0000287, GO:0009234, GO:0030145, GO:0030976, GO:0070204, KEGG:K02551, RefSeq:WP_327816323.1, SO:0001217, UniParc:UPI002E23E854, UniRef:UniRef100_UPI002E23E854, UniRef:UniRef50_P23
EC:5.4.4.2, GO:0000287, GO:0008909, GO:0009234, GO:0009697, SO:0001217, UniRef:UniRef50_P23973, UniRef:UniRef90_P23973
GO:0008270, RefSeq:WP_014114898.1, SO:0001217, UniParc:UPI00022BA92D, UniRef:UniRef100_G4NZW2, UniRef:UniRef50_P42408, UniRef:UniRef90_P42408
EC:1.1.1.22, GO:0000271, GO:0003979, GO:0006065, GO:0051287, GO:0071555, KEGG:K00012, RefSeq:WP_072181960.1, SO:0001217, UniParc:UPI0006A87C81, UniRef:UniRef100_UPI0006A87C81, UniRef:UniRef50_O3
EC:4.-.-.-, GO:0016829, RefSeq:WP_072181959.1, SO:0001217, UniParc:UPI0006A907B7, UniRef:UniRef100_A0A9Q4E1U3, UniRef:UniRef50_O34886, UniRef:UniRef90_O34886
EC:2.4.-.-, GO:0016757, GO:0016758, KEGG:K06338, SO:0001217, UniRef:UniRef50_O34413, UniRef:UniRef90_O34413
SO:0001217, UniRef:UniRef50_A0A9Q4DPQ6, UniRef:UniRef90_A0A9Q4DPQ6
BlastRules:WP_000581265, BlastRules:WP_003229031, GO:0030435, GO:0042601, KEGG:K06337, RefSeq:WP_061187219.1, SO:0001217, UniParc:UPI0006A941FA, UniRef:UniRef100_A0A9Q4E128, UniRef:UniRef50_P46
BlastRules:WP_003229028, EC:2.4.-.-, GO:0016757, GO:0030435, KEGG:K06338, RefSeq:WP_327816318.1, SO:0001217, UniParc:UPI002E210BD8, UniRef:UniRef100_UPI002E210BD8, UniRef:UniRef50_P46915, UniRef:U
BlastRules:WP_010886600, GO:0030435, GO:0042601, KEGG:K06331, SO:0001217, UniParc:UPI00022BAA98, UniRef:UniRef100_A0A9Q4E3N4, UniRef:UniRef50_O34656, UniRef:UniRef90_O34656
GO:0005886, GO:0016020, GO:0033013, RefSeq:WP_088679896.1, SO:0001217, UniParc:UPI0008695CDC, UniRef:UniRef100_UPI0008695CDC, UniRef:UniRef50_Q81BL7, UniRef:UniRef90_O34694
BlastRules:WP_004399110, EC:2.4.1.1, GO:0005737, GO:0005980, GO:0008184, GO:0030170, GO:0031220, SO:0001217, UniRef:UniRef50_P39123, UniRef:UniRef90_P39123
EC:2.4.1.21, GO:0004373, GO:0005978, GO:0009011, SO:0001217, UniRef:UniRef50_P39125, UniRef:UniRef90_P39125
SO:0001217, UniRef:UniRef50_A0A139D1K0
EC:2.7.7.27, GO:0005978, GO:0008878, KEGG:K00975, RefSeq:WP_088679893.1, SO:0001217, UniParc:UPI000B52DA0F, UniRef:UniRef100_UPI000B52DA0F, UniRef:UniRef50_P39124, UniRef:UniRef90_P39124
PFAM:PF24894.1
EC:2.7.7.27, GO:0005524, GO:0005978, GO:0008878, KEGG:K00975, SO:0001217, UniRef:UniRef50_P39122, UniRef:UniRef90_P39122
SO:0001217, UniRef:UniRef50_A0A1G6BW13
EC:2.4.1.18, GO:0003844, GO:0004553, GO:0005737, GO:0005829, GO:0005978, GO:0043169, KEGG:K00700, RefSeq:WP_088679891.1, SO:0001217, UniParc:UPI000B53651B, UniRef:UniRef100_UPI000B53651B, UniRe
SO:0000259
SO:0000269
SO:0000257
SO:0000263
SO:0000260
SO:0000262
SO:0000267
SO:0000256
SO:0000266
SO:0000269
SO:0000263
SO:0000266
SO:0000254
SO:0000268
SO:0001036
SO:0000264
SO:0000260
SO:0000264
SO:0000265
SO:0000270
SO:0000273
GO:0003735, GO:0005840, KEGG:K01985, RFAM:RF00001, SO:0000652
GO:0003735, GO:0005840, KEGG:K01980, RFAM:RF02541, SO:0001001
GO:0003735, GO:0005840, KEGG:K01977, RFAM:RF00177, SO:0001000
RFAM:RF01690, SO:0000655
RFAM:RF00059, SO:0000035
GO:0005886, GO:0015234, GO:0015888, KEGG:K16789, RefSeq:WP_072183354.1, SO:0001217, UniParc:UPI0006A8EAA3, UniRef:UniRef100_UPI0006A8EAA3, UniRef:UniRef50_O32074, UniRef:UniRef90_O32074
EC:2.3.1.-, GO:0016747, RefSeq:WP_072183353.1, SO:0001217, UniParc:UPI0006A8E03D, UniRef:UniRef100_UPI0006A8E03D, UniRef:UniRef50_O32075, UniRef:UniRef90_O32075
BlastRules:WP_003228960, GO:0002020, GO:0005886, GO:0008360, GO:0045121, GO:0072659, KEGG:K07192, RefSeq:WP_079288286.1, SO:0001217, UniParc:UPI00086A3897, UniRef:UniRef100_UPI00086A3897, UniRe
GO:0005886, GO:0045121, RefSeq:WP_041521049.1, SO:0001217, UniParc:UPI0002FEBFAA, UniRef:UniRef100_UPI0002FEBFAA, UniRef:UniRef50_O32077, UniRef:UniRef90_O32077
RefSeq:WP_041521050.1, SO:0001217, UniParc:UPI0005A1FA15, UniRef:UniRef100_G4NZY1, UniRef:UniRef50_O32078, UniRef:UniRef90_O32078
SO:0001217, UniRef:UniRef50_O32079, UniRef:UniRef90_A0A9Q6ABA7
EC:1.1.1.1, KEGG:K11440, SO:0001217, UniRef:UniRef50_A0A535B9S4, UniRef:UniRef90_UPI002FFD9359
PFAM:PF00465.25
EC:1.2.1.8, GO:0008802, GO:0019285, GO:0046872, RefSeq:WP_072183350.1, SO:0001217, UniParc:UPI0006A91ED2, UniRef:UniRef100_UPI0006A91ED2, UniRef:UniRef50_P71016, UniRef:UniRef90_P71016
BlastRules:WP_003244320, GO:0003677, RefSeq:WP_014114921.1, SO:0001217, UniParc:UPI00022BAAA7, UniRef:UniRef100_G4NZY5, UniRef:UniRef50_P71015, UniRef:UniRef90_P71015
SO:0001217, UniRef:UniRef50_UPI002DDD9293, UniRef:UniRef90_UPI0010D68C38
RFAM:RF00379, SO:0000140
BlastRules:WP_003243377, GO:0005886, GO:0006813, GO:0008324, GO:0042802, RefSeq:WP_014114922.1, SO:0001217, UniParc:UPI00022BAAA8, UniRef:UniRef100_G4NZY7, UniRef:UniRef50_O32080, UniRef:UniRef9
BlastRules:WP_003243582, GO:0005886, GO:0015379, RefSeq:WP_072183347.1, SO:0001217, UniParc:UPI0006A86F63, UniRef:UniRef100_UPI0006A86F63, UniRef:UniRef50_O32081, UniRef:UniRef90_O32081
GO:0005886, GO:0006974, RefSeq:WP_003222348.1, SO:0001217, UniParc:UPI0000060979, UniRef:UniRef100_O32082, UniRef:UniRef50_O32082, UniRef:UniRef90_O32082
BlastRules:WP_003228938, EC:3.2.1.-, GO:0004040, GO:0005576, GO:0016798, GO:0071555, RefSeq:WP_014114924.1, SO:0001217, UniParc:UPI00022BAAAA, UniRef:UniRef100_G4NZZ0, UniRef:UniRef50_O32083, UniR
GO:0005886, GO:0022857, GO:0055085, RefSeq:WP_061188064.1, SO:0001217, UniParc:UPI000772A455, UniRef:UniRef100_UPI000772A455, UniRef:UniRef50_O32084, UniRef:UniRef90_O32084
BlastRules:WP_003243534, EC:1.13.11.20, GO:0008198, GO:0016702, GO:0017172, RefSeq:WP_061188063.1, SO:0001217, UniParc:UPI000772720B, UniRef:UniRef100_A0A9Q4HID9, UniRef:UniRef50_O32085, UniRef:Ur
SO:0000254
EC:3.6.1.27, GO:0005886, GO:0008360, GO:0009252, GO:0016020, GO:0016311, GO:0046677, GO:0050380, GO:0071555, KEGG:K06153, RefSeq:WP_087987453.1, SO:0001217, UniParc:UPI000B43022D, UniRef:UniRef1
GO:0005886, GO:0055085, RefSeq:WP_014114932.1, SO:0001217, UniParc:UPI00022BAAB2, UniRef:UniRef100_G4NZZ8, UniRef:UniRef50_O32086, UniRef:UniRef90_O32086
BlastRules:WP_003228926, EC:1.1.1.371, GO:0000166, GO:0102497, RefSeq:WP_072565185.1, SO:0001217, UniParc:UPI00090AAD62, UniRef:UniRef100_A0A9Q4HH88, UniRef:UniRef50_O05265, UniRef:UniRef90_O052
EC:5.3.1.14, GO:0005737, GO:0008740, GO:0019299, GO:0019301, GO:0019324, GO:0030145, KEGG:K01813, RefSeq:WP_326229448.1, SO:0001217, UniParc:UPI001C787BFE, UniRef:UniRef100_UPI001C787BFE, UniRe
EC:5.1.3.32, GO:0005737, GO:0016857, GO:0019299, GO:0062192, KEGG:K03534, RefSeq:WP_061188059.1, SO:0001217, UniParc:UPI0006A853A5, UniRef:UniRef100_UPI0006A853A5, UniRef:UniRef50_Q65Q29, UniRef:
EC:2.7.1.5, GO:0008993, GO:0019301, KEGG:K00848, SO:0001217, UniRef:UniRef50_O05262, UniRef:UniRef90_UPI000B436E3B
BlastRules:WP_009968057, KEGG:K02430, RefSeq:WP_072182195.1, SO:0001217, UniParc:UPI0006A85D03, UniRef:UniRef100_UPI0006A85D03, UniRef:UniRef50_O05261, UniRef:UniRef90_E0TZ37
EC:1.-.-.-, GO:0016491, SO:0001217, UniRef:UniRef50_P40747, UniRef:UniRef90_P40747
GO:0005886, GO:0006935, GO:0007165, RefSeq:WP_326198157.1, SO:0001217, UniParc:UPI002DBBE8AD, UniRef:UniRef100_UPI002DBBE8AD, UniRef:UniRef50_P39215, UniRef:UniRef90_P39217
GO:0005886, GO:0006935, GO:0007165, GO:0045121, RefSeq:WP_061188055.1, SO:0001217, UniParc:UPI0006A8271A, UniRef:UniRef100_UPI0006A8271A, UniRef:UniRef50_P39215, UniRef:UniRef90_P39214
GO:0005886, GO:0006935, GO:0007165, SO:0001217, UniRef:UniRef50_P39215, UniRef:UniRef90_P39216

BlastRules:WP_003243461, GO:0005886, GO:0006935, GO:0007165, GO:0045121, SO:0001217, UniParc:UPI0022831E75, UniRef:UniRef100_A0A9Q4E4D9, UniRef:UniRef50_P39215, UniRef:UniRef90_P39215
EC:2.3.2.13, GO:0003810, GO:0030435, KEGG:K00686, SO:0001217, UniRef:UniRef50_P40746, UniRef:UniRef90_P40746
RefSeq:WP_014114945.1, SO:0001217, UniParc:UPI00022BAC1F, UniRef:UniRef100_G4P011, UniRef:UniRef50_C0H3Q3, UniRef:UniRef90_C0H3Q3
RefSeq:WP_090557208.1, SO:0001217, UniParc:UPI00086C7061, UniRef:UniRef100_UPI00086C7061, UniRef:UniRef50_O05413, UniRef:UniRef90_E0TZ45
RefSeq:WP_072182189.1, SO:0001217, UniParc:UPI0006A9301D, UniRef:UniRef100_A0A9Q4HGR1, UniRef:UniRef50_O05243, UniRef:UniRef90_O05243
EC:3.2.1.10, GO:0004556, GO:0004574, GO:0005737, GO:0009313, SO:0001217, UniRef:UniRef50_O05242, UniRef:UniRef90_O05242
SO:0001217, UniRef:UniRef50_W4RMS4
GO:0005886, GO:0050660, KEGG:K03699, SO:0001217, UniRef:UniRef50_O05241, UniRef:UniRef90_O05241
EC:3.4.24.-, GO:0005886, GO:0006508, GO:0008237, GO:0046872, KEGG:K06973, RefSeq:WP_003220791.1, SO:0001217, UniParc:UPI0001D28277, UniRef:UniRef100_A0A9Q4DNH0, UniRef:UniRef50_O05248, UniRef:Uni
GO:0005886, RefSeq:WP_014114950.1, SO:0001217, UniParc:UPI00022BAC24, UniRef:UniRef100_G4P017, UniRef:UniRef50_C0H3Q4, UniRef:UniRef90_C0H3Q4
GO:0005886, SO:0001217, UniRef:UniRef50_Q5BU39, UniRef:UniRef90_Q5BU39
GO:0005267, GO:0005886, SO:0001217, UniRef:UniRef50_Q795M8, UniRef:UniRef90_Q795M8
RefSeq:WP_014114953.1, SO:0001217, UniParc:UPI00022BAC27, UniRef:UniRef100_G4P020, UniRef:UniRef50_O05246, UniRef:UniRef90_A0A2M8SWA0
SO:0001217, UniRef:UniRef50_O05245, UniRef:UniRef90_D4G0M6
COG:COG0166, COG:G, EC:5.3.1.9, GO:0004347, GO:0005829, GO:0006094, GO:0006096, GO:0048029, GO:0051156, GO:0097367, RefSeq:WP_003220778.1, SO:0001217, UniParc:UPI0001D28271, UniRef:UniRef100_A0
SO:0001217, UniRef:UniRef50_A0AAP8T2U6
SO:0001217, UniRef:UniRef50_A0A5S9MEL3
SO:0001217, UniRef:UniRef50_A0A640LH31
SO:0001217, UniRef:UniRef50_UPI000EADC167
GO:0005886, KEGG:K09779, RefSeq:WP_003220771.1, SO:0001217, UniParc:UPI0000060994, UniRef:UniRef100_O32087, UniRef:UniRef50_O32087, UniRef:UniRef90_O32087
GO:0003676, GO:0005737, RefSeq:WP_003228864.1, SO:0001217, UniParc:UPI0000060995, UniRef:UniRef100_P80870, UniRef:UniRef50_P80870, UniRef:UniRef90_P80870
RFAM:RF02451, SO:0000655
EC:2.6.1.-, GO:0005737, GO:0006520, GO:0008483, GO:0008652, GO:0030170, RefSeq:WP_014114958.1, SO:0001217, UniParc:UPI00022BAC2C, UniRef:UniRef100_G4P027, UniRef:UniRef50_Q795M6, UniRef:UniRef90_
GO:0043565, RefSeq:WP_003220765.1, SO:0001217, UniParc:UPI0001D2826C, UniRef:UniRef100_A0A9Q4HGQ7, UniRef:UniRef50_O05236, UniRef:UniRef90_O05236
SO:0001217, UniRef:UniRef50_W4RJ85
RefSeq:WP_014114961.1, SO:0001217, UniParc:UPI00022BAC2F, UniRef:UniRef100_G4P030, UniRef:UniRef50_O05234, UniRef:UniRef90_O05234
PFAM:PF00155.27
SO:0001217, UniRef:UniRef50_A0A150M8Q9
BlastRules:WP_000420390, BlastRules:WP_003159138, BlastRules:WP_003228853, EC:2.7.13.3, GO:0000155, GO:0005524, GO:0005886, GO:0030435, GO:0045881, SO:0001217, UniRef:UniRef50_Q08430, UniRef:UniRef
GO:0005886, KEGG:K06347, SO:0001217, UniRef:UniRef50_Q08429, UniRef:UniRef90_Q08429
KEGG:K06348, SO:0001217, UniRef:UniRef50_O05231, UniRef:UniRef90_UPI002E21FDCC
GO:0005886, GO:0022857, KEGG:K08161, SO:0001217, UniRef:UniRef50_P40760, UniRef:UniRef90_P40760
GO:0005886, GO:0006508, GO:0008360, GO:0008658, GO:0008955, GO:0009002, GO:0009252, GO:0030288, GO:0071555, SO:0001217, UniRef:UniRef50_P40750, UniRef:UniRef90_P40750
GO:0015035, RefSeq:WP_003220746.1, SO:0001217, UniParc:UPI0001D28262, UniRef:UniRef100_A0A9Q4E5J4, UniRef:UniRef50_P40761, UniRef:UniRef90_P40761
GO:0005886, RefSeq:WP_003220744.1, SO:0001217, UniParc:UPI0001D28261, UniRef:UniRef100_A0A2M8SWS4, UniRef:UniRef50_O05249, UniRef:UniRef90_O05249
BlastRules:WP_003228835, EC:2.7.13.3, GO:0000155, GO:0005524, GO:0005886, GO:0006355, KEGG:K11614, RefSeq:WP_072182180.1, SO:0001217, UniParc:UPI0006A80A1B, UniRef:UniRef100_UPI0006A80A1B, UniRe
BlastRules:WP_009968074, GO:0000156, GO:0003677, GO:0003700, GO:0005737, RefSeq:WP_003220739.1, SO:0001217, UniParc:UPI0001D2825F, UniRef:UniRef100_G4P041, UniRef:UniRef50_O05251, UniRef:UniRef90
BlastRules:WP_003228831, GO:0005886, GO:0045121, KEGG:K07335, SO:0001217, UniRef:UniRef50_O05252, UniRef:UniRef90_O05252
EC:7.6.2.-, GO:0005524, GO:0005886, GO:0016887, KEGG:K23537, SO:0001217, UniRef:UniRef50_A2RKA7, UniRef:UniRef90_O05253
SO:0001217, UniRef:UniRef50_UPI002851DAB7
BlastRules:WP_003228827, GO:0005886, GO:0022857, KEGG:K23536, RefSeq:WP_014114973.1, SO:0001217, UniParc:UPI00022BADCD, UniRef:UniRef100_G4P045, UniRef:UniRef50_O05255, UniRef:UniRef90_O05255
BlastRules:WP_003244241, GO:0005886, GO:0006814, GO:0008514, GO:0015293, KEGG:K11616, RefSeq:WP_014114974.1, SO:0001217, UniParc:UPI00022BADCE, UniRef:UniRef100_G4P046, UniRef:UniRef50_O05256,
RefSeq:WP_072182178.1, SO:0001217, UniParc:UPI0006A824F6, UniRef:UniRef100_UPI0006A824F6, UniRef:UniRef50_O05257, UniRef:UniRef90_G4P047
GO:0005886, GO:0015385, GO:0030435, GO:0051139, GO:0098662, SO:0001217, UniRef:UniRef50_Q9K2S2, UniRef:UniRef90_Q9K2S2
GO:0005886, GO:0006812, GO:0006814, GO:0008324, GO:0015297, GO:1902600, SO:0001217, UniRef:UniRef50_O05260, UniRef:UniRef90_O05260
GO:0005886, GO:0008137, GO:0015297, GO:0015385, GO:0015386, GO:0042773, SO:0001217, UniRef:UniRef50_O05229, UniRef:UniRef90_O05229
GO:0005886, GO:0006814, GO:0008324, GO:0015297, GO:1902600, RefSeq:WP_014114979.1, SO:0001217, UniParc:UPI00022BADD3, UniRef:UniRef100_G4P052, UniRef:UniRef50_Q7WY60, UniRef:UniRef90_Q7WY60
GO:0005886, GO:0015385, GO:0036376, RefSeq:WP_014114980.1, SO:0001217, UniParc:UPI00022BADD4, UniRef:UniRef100_G4P053, UniRef:UniRef50_O05228, UniRef:UniRef90_O05228
GO:0005886, GO:0015297, GO:0015385, GO:0051139, GO:0098662, RefSeq:WP_072182177.1, SO:0001217, UniParc:UPI0006A89616, UniRef:UniRef100_UPI0006A89616, UniRef:UniRef50_O05227, UniRef:UniRef90_O052
EC:3.1.2.-, GO:0005829, GO:0061522, RefSeq:WP_003220719.1, SO:0001217, UniParc:UPI0001D28FD3, UniRef:UniRef100_G4P055, UniRef:UniRef50_P14205, UniRef:UniRef90_P14205
BlastRules:WP_003220716, GO:0000160, GO:0003677, GO:0005737, GO:0006355, GO:0030420, KEGG:K07691, RefSeq:WP_003220716.1, SO:0001217, UniParc:UPI00000008F9, UniRef:UniRef100_P14204, UniRef:UniRef
SO:0001217, UniRef:UniRef50_Q99027
GO:0005186, GO:0005576, GO:0030420, KEGG:K02253, RefSeq:WP_014114983.1, SO:0001217, UniParc:UPI0001F5B2D4, UniRef:UniRef100_I2DA18, UniRef:UniRef50_D4G0R3, UniRef:UniRef90_D4G0R3
KEGG:K02251, SO:0001217, UniRef:UniRef50_G4P059, UniRef:UniRef90_G4P059
BlastRules:WP_003220708, GO:1900192, RefSeq:WP_003220708.1, SO:0001217, UniParc:UPI00000008F8, UniRef:UniRef100_Q99039, UniRef:UniRef50_Q99039, UniRef:UniRef90_Q99039
RefSeq:WP_014114986.1, SO:0001217, UniParc:UPI00022BADD9, UniRef:UniRef100_G4P062, UniRef:UniRef50_O32089, UniRef:UniRef90_A0A9Q4E588
SO:0001217, UniRef:UniRef50_P14203, UniRef:UniRef90_P14203
SO:0001217, UniRef:UniRef50_A0A063YNU2
EC:6.3.4.21, GO:0004516, GO:0005829, GO:0009435, GO:0016740, GO:0034355, KEGG:K00763, SO:0001217, UniRef:UniRef50_O32090, UniRef:UniRef90_O32090
EC:3.-.-.-, GO:0016787, KEGG:K08281, SO:0001217, UniRef:UniRef50_O32091, UniRef:UniRef90_O32091
RefSeq:WP_014114990.1, SO:0001217, UniParc:UPI00022BADDD, UniRef:UniRef100_G4P066, UniRef:UniRef50_O32092, UniRef:UniRef90_D4G0S1
RefSeq:WP_014114991.1, SO:0001217, UniParc:UPI00022BADDE, UniRef:UniRef100_G4P067, UniRef:UniRef50_O32093, UniRef:UniRef90_O32093
RefSeq:WP_014114992.1, SO:0001217, UniParc:UPI00022BADDF, UniRef:UniRef100_G4P068, UniRef:UniRef50_O32094, UniRef:UniRef90_G4P068
GO:0005886, GO:0055085, RefSeq:WP_014114993.1, SO:0001217, UniParc:UPI00022BADE0, UniRef:UniRef100_G4P069, UniRef:UniRef50_O32095, UniRef:UniRef90_O32095
SO:0001217, UniRef:UniRef50_O32096
RefSeq:WP_014114995.1, SO:0001217, UniParc:UPI00022BADE2, UniRef:UniRef100_G4P071, UniRef:UniRef50_O32097, UniRef:UniRef90_O32097
EC:3.-.-.-, GO:0016787, RefSeq:WP_014114996.1, SO:0001217, UniParc:UPI00022BADE3, UniRef:UniRef100_G4P072, UniRef:UniRef50_O32098, UniRef:UniRef90_O32098
EC:1.1.1.320, GO:0004757, GO:0005737, GO:0006729, GO:0016491, GO:0102306, SO:0001217, UniRef:UniRef50_O32099, UniRef:UniRef90_O32099
GO:0005886, GO:0009306, SO:0001217, UniRef:UniRef50_O32100, UniRef:UniRef90_O32100
SO:0001217, UniRef:UniRef50_A0A5S9MJK0
PFAM:PF01580.24
SO:0001217, UniRef:UniRef50_UPI001E358A8B
GO:0009306, SO:0001217, UniRef:UniRef50_P71070, UniRef:UniRef90_E0TZY1
BlastRules:WP_003228763, RefSeq:WP_010329928.1, SO:0001217, UniParc:UPI00022BAF65, UniRef:UniRef100_A0A9W5LG20, UniRef:UniRef50_I2C9M8, UniRef:UniRef90_A0A9W5LG20
GO:0005576, RefSeq:WP_003220661.1, SO:0001217, UniParc:UPI000195C766, UniRef:UniRef100_C0SP85, UniRef:UniRef50_C0SP85, UniRef:UniRef90_C0SP85
SO:0001217, UniRef:UniRef50_A0A7U9HWJ8
PFAM:PF17853.6
PFAM:PF05222.21
EC:1.4.1.1, GO:0000286, GO:0042853, KEGG:K00259, SO:0001217, UniRef:UniRef50_A0A645EUI9, UniRef:UniRef90_UPI00366F62F7
SO:0001217, UniRef:UniRef50_C0SPB1, UniRef:UniRef90_C0SPB1
GO:0005829, GO:0019290, RefSeq:WP_014115006.1, SO:0001217, UniParc:UPI00022BAF6A, UniRef:UniRef100_A0A9Q4E566, UniRef:UniRef50_C0H3Q6, UniRef:UniRef90_C0H3Q6
SO:0001217, UniRef:UniRef50_A0A4U3ABN2
SO:0001217, UniRef:UniRef50_UPI0006BEC1FB
SO:0001217, UniRef:UniRef50_UPI00117C782E
SO:0001217, UniRef:UniRef50_UPI00241DDF63, UniRef:UniRef90_UPI0022807B99
EC:3.3.2.1, GO:0008908, KEGG:K01252, SO:0001217, UniRef:UniRef50_P45743, UniRef:UniRef90_P45743
SO:0001217, UniRef:UniRef50_A0A7Z8ZEM3
SO:0001217, UniRef:UniRef50_A0A6I5CHQ6, UniRef:UniRef90_UPI001961B526
PFAM:PF00425.24
SO:0001217, UniRef:UniRef50_A0A2U1FRB1
EC:1.3.1.28, GO:0005737, GO:0008667, GO:0016616, GO:0019290, GO:0071281, RefSeq:WP_088679765.1, SO:0001217, UniParc:UPI000B5343AA, UniRef:UniRef100_UPI000B5343AA, UniRef:UniRef50_P39071, UniRef:U
BlastRules:WP_003228740, EC:3.1.-.-, GO:0005737, GO:0052689, SO:0001217, UniRef:UniRef50_O32102, UniRef:UniRef90_O32102
EC:1.-.-.-, GO:0016491, GO:0046872, SO:0001217, UniRef:UniRef50_O32103, UniRef:UniRef90_O32103
GO:0005886, GO:0015225, KEGG:K03523, RefSeq:WP_014115015.1, SO:0001217, UniParc:UPI00022BAF73, UniRef:UniRef100_G4P093, UniRef:UniRef50_O32104, UniRef:UniRef90_O32104
GO:0005886, KEGG:K07084, RefSeq:WP_014115016.1, SO:0001217, UniParc:UPI00022BAF74, UniRef:UniRef100_G4P094, UniRef:UniRef50_O32105, UniRef:UniRef90_O32105
EC:3.4.11.1, GO:0005737, GO:0006508, GO:0008233, GO:0030145, GO:0070006, RefSeq:WP_326247717.1, SO:0001217, UniParc:UPI002DB97D5C, UniRef:UniRef100_UPI002DB97D5C, UniRef:UniRef50_O32106, UniRef:
GO:0005886, KEGG:K09775, RefSeq:WP_014115018.1, SO:0001217, UniParc:UPI00022BAF76, UniRef:UniRef100_G4P096, UniRef:UniRef50_O32107, UniRef:UniRef90_O32107
BlastRules:WP_009969090, GO:0004553, GO:0009254, GO:0019867, SO:0001217, UniRef:UniRef50_O32108, UniRef:UniRef90_O32108
GO:0005886, RefSeq:WP_014115020.1, SO:0001217, UniParc:UPI00022BAF78, UniRef:UniRef100_G4P098, UniRef:UniRef50_O32109, UniRef:UniRef90_O32109
EC:1.6.-.-, GO:0003955, GO:0019646, RefSeq:WP_072182287.1, SO:0001217, UniParc:UPI0006A82525, UniRef:UniRef100_UPI0006A82525, UniRef:UniRef50_Q2FZV7, UniRef:UniRef90_O05267
COG:COG0492, COG:O, EC:1.18.1.2, GO:0004324, GO:0050660, GO:0050661, KEGG:K21567, RefSeq:WP_003220618.1, SO:0001217, UniParc:UPI000195C771, UniRef:UniRef100_O05268, UniRef:UniRef50_O05268, UniR
RefSeq:WP_003220614.1, SO:0001217, UniParc:UPI0001D28EC0, UniRef:UniRef100_E0U004, UniRef:UniRef50_O32111, UniRef:UniRef90_E0U004
EC:1.7.1.7, GO:0003920, GO:0006163, GO:1902560, RefSeq:WP_014115023.1, SO:0001217, UniParc:UPI00022BAF7B, UniRef:UniRef100_G4P0A3, UniRef:UniRef50_Q81JJ9, UniRef:UniRef90_O05269
BlastRules:WP_000275806, BlastRules:WP_000275834, BlastRules:WP_003244329, GO:0030435, KEGG:K07734, SO:0001217, UniRef:UniRef50_P21341, UniRef:UniRef90_P21341
RefSeq:WP_072182288.1, SO:0001217, UniParc:UPI0006A870D7, UniRef:UniRef100_UPI0006A870D7, UniRef:UniRef50_A0A0E4HAE8, UniRef:UniRef90_G4P0A5
GO:0005737, GO:0016226, GO:0051537, GO:0097428, RefSeq:WP_003222918.1, SO:0001217, UniParc:UPI0001D28A93, UniRef:UniRef100_A0A9Q4DNW2, UniRef:UniRef50_O32113, UniRef:UniRef90_O32113
EC:5.1.1.7, GO:0005829, GO:0008837, GO:0009089, KEGG:K01778, RefSeq:WP_072182289.1, SO:0001217, UniParc:UPI0006A8630A, UniRef:UniRef100_UPI0006A8630A, UniRef:UniRef50_Q81XR2, UniRef:UniRef90_O32
SO:0001217, UniRef:UniRef50_O32115, UniRef:UniRef90_UPI0034CDC2E9
RefSeq:WP_003222913.1, SO:0001217, UniParc:UPI00000609E3, UniRef:UniRef100_O32116, UniRef:UniRef50_O32116, UniRef:UniRef90_O32116
EC:1.6.-.-, GO:0016491, SO:0001217, UniRef:UniRef50_O32117, UniRef:UniRef90_O32117
RefSeq:WP_072182293.1, SO:0001217, UniParc:UPI0006A8442E, UniRef:UniRef100_UPI0006A8442E, UniRef:UniRef50_O32118, UniRef:UniRef90_O32118
GO:0005506, GO:0009399, GO:0016226, GO:0051536, RefSeq:WP_003151955.1, SO:0001217, UniParc:UPI00019765F2, UniRef:UniRef100_O32119, UniRef:UniRef50_O32119, UniRef:UniRef90_O32119
SO:0001217, UniRef:UniRef50_P39839, UniRef:UniRef90_A0A6H0WRC5
EC:2.7.1.39, GO:0004413, GO:0005524, GO:0005737, GO:0009088, KEGG:K00872, RefSeq:WP_072182295.1, SO:0001217, UniParc:UPI0006A8DDC5, UniRef:UniRef100_UPI0006A8DDC5, UniRef:UniRef50_P04948, UniRe
EC:4.2.3.1, GO:0004795, GO:0005737, GO:0009088, GO:0016841, GO:0019344, GO:0030170, KEGG:K01733, SO:0001217, UniRef:UniRef50_Q9K7E3, UniRef:UniRef90_P04990
EC:1.1.1.3, GO:0004412, GO:0005576, GO:0005737, GO:0009086, GO:0009088, GO:0046872, GO:0050661, GO:0070403, RefSeq:WP_061188012.1, SO:0001217, UniParc:UPI0006A8C436, UniRef:UniRef100_A0A9Q4E5E5
BlastRules:WP_003242649, GO:0016020, GO:0030435, GO:0030436, GO:0042601, SO:0001217, UniRef:UniRef50_O32123, UniRef:UniRef90_O32123
GO:0006629, GO:0008962, RefSeq:WP_014115036.1, SO:0001217, UniParc:UPI00022BB115, UniRef:UniRef100_G4P0B9, UniRef:UniRef50_O32124, UniRef:UniRef90_O32124
BlastRules:WP_003243196, EC:3.1.3.5, GO:0005737, GO:0016791, GO:0046872, GO:0106411, RefSeq:WP_072182296.1, SO:0001217, UniParc:UPI0006A81905, UniRef:UniRef100_UPI0006A81905, UniRef:UniRef50_O3212
EC:3.1.-.-, GO:0004540, GO:0110001, RefSeq:WP_072182297.1, SO:0001217, UniParc:UPI0006A881AD, UniRef:UniRef100_UPI0006A881AD, UniRef:UniRef50_O32126, UniRef:UniRef90_O32126
RefSeq:WP_003228682.1, SO:0001217, UniParc:UPI000195C6E5, UniRef:UniRef100_O32127, UniRef:UniRef50_O32127, UniRef:UniRef90_O32127
GO:0005886, RefSeq:WP_061188010.1, SO:0001217, UniParc:UPI0006A87790, UniRef:UniRef100_UPI0006A87790, UniRef:UniRef50_O32128, UniRef:UniRef90_O32128
COG:COG0320, COG:H, EC:2.8.1.8, GO:0005737, GO:0009107, GO:0016992, GO:0046872, GO:0051539, GO:1904047, KEGG:K03644, RefSeq:WP_003222888.1, SO:0001217, UniParc:UPI000012E6C0, UniRef:UniRef100_
BlastRules:WP_003243462, EC:3.4.-.-, GO:0004222, GO:0006508, GO:0030154, GO:0030435, GO:0046872, GO:0071555, KEGG:K21472, RefSeq:WP_041521059.1, SO:0001217, UniParc:UPI0005A2FE76, UniRef:UniRef10
GO:0005886, GO:0030435, GO:0030436, RefSeq:WP_072182298.1, SO:0001217, UniParc:UPI0006A92C87, UniRef:UniRef100_UPI0006A92C87, UniRef:UniRef50_O32131, UniRef:UniRef90_O32131
SO:0001217, UniRef:UniRef50_O32132, UniRef:UniRef90_O32132

GO:0008253, GO:0008768, GO:0009166, GO:0030288, GO:0046872, RefSeq:WP_072182300.1, SO:0001217, UniParc:UPI0006A91C15, UniRef:UniRef100_UPI0006A91C15, UniRef:UniRef50_O32133, UniRef:UniRef90_O32
GO:0005886, SO:0001217, UniRef:UniRef50_O32134, UniRef:UniRef90_O32134
SO:0001217, UniRef:UniRef50_W4VE29
SO:0001217, UniRef:UniRef50_UPI00103962A7
RefSeq:WP_014115046.1, SO:0001217, UniParc:UPI00022BB11F, UniRef:UniRef100_G4P0D1, UniRef:UniRef50_O32136, UniRef:UniRef90_O32136
SO:0001217, UniRef:UniRef50_UPI002B1EFAE8, UniRef:UniRef90_UPI002B1EFAE8
SO:0001217, UniRef:UniRef50_UPI00046E904F
BlastRules:WP_003244351, GO:0003677, GO:0006144, SO:0001217, UniRef:UniRef50_O32138, UniRef:UniRef90_O32138
BlastRules:WP_003243942, GO:0005886, GO:0042906, GO:0042907, SO:0001217, UniRef:UniRef50_A0A2A5JY22, UniRef:UniRef90_O32139
BlastRules:WP_003244570, GO:0005886, GO:0042906, GO:0042907, KEGG:K16169, SO:0001217, UniRef:UniRef50_O32140, UniRef:UniRef90_O32140
EC:1.7.3.3, EC:4.1.1.97, GO:0004846, GO:0019628, KEGG:K16838, SO:0001217, UniRef:UniRef50_O32141, UniRef:UniRef90_A0A9Q4DQM2
RefSeq:WP_003240909.1, SO:0001217, UniParc:UPI0001E85AE1, UniRef:UniRef100_A0A9W5LFY2, UniRef:UniRef50_A0A9W5LFY2, UniRef:UniRef90_A0A9W5LFY2
SO:0001217, UniRef:UniRef50_G4P0D9, UniRef:UniRef90_G4P0D9
RefSeq:WP_014115056.1, SO:0001217, UniParc:UPI00022BB129, UniRef:UniRef100_G4P0E1, UniRef:UniRef50_A0A7Y8RZX4, UniRef:UniRef90_G4P0E1
RefSeq:WP_014115057.1, SO:0001217, UniParc:UPI00022BB12A, UniRef:UniRef100_G4P0E2, UniRef:UniRef50_Q62Z42, UniRef:UniRef90_G4P0E2
RefSeq:WP_041521061.1, SO:0001217, UniParc:UPI0005A2BADB, UniRef:UniRef100_UPI0005A2BADB, UniRef:UniRef50_UPI0027DBA269, UniRef:UniRef90_UPI0005A2BADB
EC:1.17.1.4, GO:0004854, GO:0009114, GO:0046872, GO:0051537, RefSeq:WP_072182705.1, SO:0001217, UniParc:UPI0006A8A257, UniRef:UniRef100_UPI0006A8A257, UniRef:UniRef50_O32143, UniRef:UniRef90_O321
EC:1.17.1.4, GO:0016491, SO:0001217, UniRef:UniRef50_O32144, UniRef:UniRef90_A0A5D4N1X2
EC:1.17.1.4, SO:0001217, UniRef:UniRef50_O32145, UniRef:UniRef90_G4P0E5
EC:2.7.7.76, KEGG:K07141, SO:0001217, UniRef:UniRef50_O32146, UniRef:UniRef90_E0U0R3
KEGG:K07402, RefSeq:WP_327816455.1, SO:0001217, UniParc:UPI002E1BCA46, UniRef:UniRef100_UPI002E1BCA46, UniRef:UniRef50_O32147, UniRef:UniRef90_A0AAE2NXF6
BlastRules:WP_003244520, EC:2.6.1.112, GO:0000256, GO:0004760, GO:0005777, GO:0006144, GO:0008453, GO:0019265, RefSeq:WP_090557126.1, SO:0001217, UniParc:UPI000868F01C, UniRef:UniRef100_UPI00086
EC:3.5.3.9, GO:0000256, GO:0005737, GO:0006144, GO:0008270, GO:0009442, GO:0047652, SO:0001217, UniRef:UniRef50_O32149, UniRef:UniRef90_O32149
PFAM:PF04389.22
SO:0001217, UniRef:UniRef50_G4P0F0, UniRef:UniRef90_G4P0F0
EC:7.-.-.-, GO:0005524, GO:0008643, GO:0016887, GO:0055052, GO:0140359, SO:0001217, UniRef:UniRef50_O32151, UniRef:UniRef90_O32151
BlastRules:WP_003228628, GO:0003677, GO:0003700, GO:0045892, KEGG:K10711, SO:0001217, UniRef:UniRef50_O32152, UniRef:UniRef90_O32152
EC:2.7.1.-, GO:0005524, GO:0016301, RefSeq:WP_061187988.1, SO:0001217, UniParc:UPI000772C9C7, UniRef:UniRef100_UPI000772C9C7, UniRef:UniRef50_O32153, UniRef:UniRef90_O32153
GO:0005886, GO:0055085, SO:0001217, UniRef:UniRef50_O32154, UniRef:UniRef90_O32154
GO:0005886, GO:0055085, SO:0001217, UniRef:UniRef50_O32155, UniRef:UniRef90_O32155
SO:0001217, UniRef:UniRef50_UPI002E1CEEC8
SO:0001217, UniRef:UniRef50_UPI00036BB078, UniRef:UniRef90_UPI00036BB078
BlastRules:WP_003243688, EC:3.5.-.-, GO:0004360, GO:0006002, GO:0006047, GO:0006487, GO:0016787, GO:0097367, KEGG:K10708, RefSeq:WP_061187984.1, SO:0001217, UniParc:UPI0006A7F2DF, UniRef:UniRef10
GO:0006289, RefSeq:WP_088679747.1, SO:0001217, UniParc:UPI0006A8DCD4, UniRef:UniRef100_UPI0006A8DCD4, UniRef:UniRef50_O32158, UniRef:UniRef90_O32158
EC:1.-.-.-, GO:0005737, GO:0016491, RefSeq:WP_072183529.1, SO:0001217, UniParc:UPI0006A84421, UniRef:UniRef100_UPI0006A84421, UniRef:UniRef50_O32159, UniRef:UniRef90_O32159
KEGG:K06424, RefSeq:WP_003240873.1, SO:0001217, UniParc:UPI00022BB2A3, UniRef:UniRef100_A0A9Q2QRY0, UniRef:UniRef50_Q7WY59, UniRef:UniRef90_A0A9Q4DRC9
RefSeq:WP_003222817.1, SO:0001217, UniParc:UPI0001D289DA, UniRef:UniRef100_A0A9Q4E990, UniRef:UniRef50_O32160, UniRef:UniRef90_A0A9Q4E990
RefSeq:WP_014115076.1, SO:0001217, UniParc:UPI00022BB2A5, UniRef:UniRef100_G4P0G3, UniRef:UniRef50_A0A919WE90, UniRef:UniRef90_G4P0G3
COG:COG0719, COG:O, GO:0016226, KEGG:K09014, RefSeq:WP_003228608.1, SO:0001217, UniParc:UPI0000060A10, UniRef:UniRef100_O32162, UniRef:UniRef50_O32162, UniRef:UniRef90_O32162
BlastRules:WP_000009523, BlastRules:WP_003222809, EC:2.-.-.-, GO:0005198, GO:0005737, GO:0006879, GO:0008198, GO:0016226, GO:0016740, GO:0051537, RefSeq:WP_003222809.1, SO:0001217, UniParc:UPI0000
BlastRules:WP_001020754, BlastRules:WP_003228604, EC:2.8.1.7, GO:0006534, GO:0030170, GO:0031071, KEGG:K11717, RefSeq:WP_061187980.1, SO:0001217, UniParc:UPI0007727BEE, UniRef:UniRef100_UPI00077
GO:0016226, KEGG:K09015, RefSeq:WP_072183526.1, SO:0001217, UniParc:UPI0006A942CF, UniRef:UniRef100_UPI0006A942CF, UniRef:UniRef50_O32165, UniRef:UniRef90_O32165
SO:0001217, UniRef:UniRef50_A0A1G1R5F3, UniRef:UniRef90_W4RTY9
GO:0005524, GO:0016226, SO:0001217, UniRef:UniRef50_A0A235F418, UniRef:UniRef90_UPI00366C2E4B
SO:0001217, UniRef:UniRef50_C0H3Q9, UniRef:UniRef90_C0H3Q9
GO:0016491, GO:0051920, SO:0001217, UniRef:UniRef50_O32166, UniRef:UniRef90_O32166
BlastRules:WP_000757014, BlastRules:WP_003228595, GO:0005886, GO:0006865, KEGG:K02073, RefSeq:WP_014115080.1, SO:0001217, UniParc:UPI00022BB2A9, UniRef:UniRef100_G4P0H1, UniRef:UniRef50_O32167,
SO:0001217, UniRef:UniRef50_A0A380H8S2
BlastRules:WP_003242531, EC:7.4.2.11, GO:0005524, GO:0005886, GO:0016887, GO:0033232, RefSeq:WP_072183524.1, SO:0001217, UniParc:UPI0006A81774, UniRef:UniRef100_UPI0006A81774, UniRef:UniRef50_O32
RFAM:RF00162, SO:0000140
RefSeq:WP_014115084.1, SO:0001217, UniParc:UPI00022BB2AD, UniRef:UniRef100_G4P0H5, UniRef:UniRef50_O32170, UniRef:UniRef90_O32170
GO:0005737, GO:0005829, GO:0015035, GO:0045454, RefSeq:WP_014115085.1, SO:0001217, UniParc:UPI00022BB2AE, UniRef:UniRef100_G4P0H6, UniRef:UniRef50_O32171, UniRef:UniRef90_O32171
GO:0006364, GO:0043822, SO:0001217, UniRef:UniRef50_O32172, UniRef:UniRef90_O32172
RefSeq:WP_003222783.1, SO:0001217, UniParc:UPI0001D289C9, UniRef:UniRef100_A0A9Q4E6B3, UniRef:UniRef50_O32173, UniRef:UniRef90_O32173
GO:0005829, GO:0005960, GO:0009107, GO:0009249, GO:0019464, RefSeq:WP_072183522.1, SO:0001217, UniParc:UPI0006A8A9F0, UniRef:UniRef100_UPI0006A8A9F0, UniRef:UniRef50_O32174, UniRef:UniRef90_O32
EC:1.20.4.1, GO:0016491, KEGG:K00537, RefSeq:WP_003222779.1, SO:0001217, UniParc:UPI0000060A1E, UniRef:UniRef100_O32175, UniRef:UniRef50_O32175, UniRef:UniRef90_O32175
BlastRules:WP_003244094, EC:1.3.99.-, GO:0003995, GO:0006635, GO:0050660, RefSeq:WP_072183521.1, SO:0001217, UniParc:UPI0006A7F92F, UniRef:UniRef100_UPI0006A7F92F, UniRef:UniRef50_O32176, UniRef:Un
EC:2.3.1.16, EC:2.3.1.9, GO:0003988, GO:0006635, GO:0010124, RefSeq:WP_072183520.1, SO:0001217, UniParc:UPI0006A828EA, UniRef:UniRef100_UPI0006A828EA, UniRef:UniRef50_O32177, UniRef:UniRef90_O32177
EC:1.1.1.35, GO:0003857, GO:0006635, GO:0016491, GO:0070403, SO:0001217, UniRef:UniRef50_O32178, UniRef:UniRef90_O32178
RefSeq:WP_003240817.1, SO:0001217, UniParc:UPI0001CE3B60, UniRef:UniRef100_A0A0C3KA95, UniRef:UniRef50_C0H3R0, UniRef:UniRef90_C0H3R0
EC:1.5.5.2, GO:0004657, GO:0006562, GO:0010133, GO:0071949, SO:0001217, UniRef:UniRef50_Q8RMG1, UniRef:UniRef90_Q8RMG1
RefSeq:WP_014115091.1, SO:0001217, UniParc:UPI00022BB2B4, UniRef:UniRef100_G4P0I6, UniRef:UniRef50_C0H3R1, UniRef:UniRef90_C0H3R1
RefSeq:WP_003222766.1, SO:0001217, UniParc:UPI0001CE3B62, UniRef:UniRef100_A0A162TP72, UniRef:UniRef50_O32180, UniRef:UniRef90_O32180
BlastRules:WP_003242592, GO:0003677, GO:0003700, GO:0006355, GO:0006950, RefSeq:WP_072183518.1, SO:0001217, UniParc:UPI0006A8941B, UniRef:UniRef100_A0A9Q4HH29, UniRef:UniRef50_O32181, UniRef:Un
SO:0001217, UniRef:UniRef50_A0A4Q5VHQ6
GO:0005886, GO:0022857, GO:0055085, SO:0001217, UniRef:UniRef50_O32182, UniRef:UniRef90_O32182
EC:5.3.2.-, GO:0016853, RefSeq:WP_072183517.1, SO:0001217, UniParc:UPI0006A83D7A, UniRef:UniRef100_UPI0006A83D7A, UniRef:UniRef50_O32183, UniRef:UniRef90_O32183
RefSeq:WP_014115095.1, SO:0001217, UniParc:UPI00022BB2B8, UniRef:UniRef100_G4P0J1, UniRef:UniRef50_O32185, UniRef:UniRef90_O32185
GO:0000976, GO:0003700, GO:0006355, RefSeq:WP_061187969.1, SO:0001217, UniParc:UPI0006A8DE0B, UniRef:UniRef100_UPI0006A8DE0B, UniRef:UniRef50_O32186, UniRef:UniRef90_O32186
RefSeq:WP_014115097.1, SO:0001217, UniParc:UPI00022BB2BA, UniRef:UniRef100_A0A9Q4E6A6, UniRef:UniRef50_O32187, UniRef:UniRef90_O32187
GO:0005524, GO:0005886, GO:0006826, GO:0016887, SO:0001217, UniRef:UniRef50_O32188, UniRef:UniRef90_O32188
SO:0001217, UniRef:UniRef50_O32189, UniRef:UniRef90_UPI0020900205
SO:0001217, UniRef:UniRef50_A0A919X7H3
EC:3.4.24.-, KEGG:K08602, SO:0001217, UniRef:UniRef50_C6J175, UniRef:UniRef90_D4G164
EC:1.-.-.-, GO:0016491, RefSeq:WP_088679743.1, SO:0001217, UniParc:UPI000B537CD2, UniRef:UniRef100_UPI000B537CD2, UniRef:UniRef50_P37959, UniRef:UniRef90_P37959
BlastRules:WP_003228537, GO:0003677, GO:0008199, GO:0016722, KEGG:K04047, RefSeq:WP_003221689.1, SO:0001217, UniParc:UPI0001D28C12, UniRef:UniRef100_A0A9Q4HAM8, UniRef:UniRef50_P37960, UniRef:U
BlastRules:WP_003228534, EC:3.4.21.107, GO:0004252, GO:0005886, GO:0006508, RefSeq:WP_014115104.1, SO:0001217, UniParc:UPI00022BB437, UniRef:UniRef100_G4P0K0, UniRef:UniRef50_Q9R9I1, UniRef:UniRef5
BlastRules:WP_003228529, GO:0000156, GO:0000976, GO:0005829, GO:0006355, GO:0032993, KEGG:K07770, SO:0001217, UniRef:UniRef50_O32192, UniRef:UniRef90_O32192
BlastRules:WP_003228527, EC:2.7.13.3, GO:0000155, GO:0005524, GO:0005886, RefSeq:WP_014115106.1, SO:0001217, UniParc:UPI00022BB438, UniRef:UniRef100_G4P0K3, UniRef:UniRef50_O32193, UniRef:UniRef90_
BlastRules:WP_003221681, RefSeq:WP_003221681.1, SO:0001217, UniParc:UPI00000BB818, UniRef:UniRef100_O32302, UniRef:UniRef50_O32302, UniRef:UniRef90_O32302
GO:0003677, RefSeq:WP_014115107.1, SO:0001217, UniParc:UPI00022BB439, UniRef:UniRef100_G4P0K5, UniRef:UniRef50_P40950, UniRef:UniRef90_P40950
EC:4.2.1.2, GO:0004333, GO:0005737, GO:0006099, GO:0006106, GO:0006108, GO:0045239, KEGG:K01679, RefSeq:WP_014115108.1, SO:0001217, UniParc:UPI00022BB43A, UniRef:UniRef100_G4P0K6, UniRef:UniRef5
RefSeq:WP_014115109.1, SO:0001217, UniParc:UPI00022BB43B, UniRef:UniRef100_G4P0K7, UniRef:UniRef50_C0H3R3, UniRef:UniRef90_C0H3R3
GO:0005886, GO:0009847, KEGG:K06288, SO:0001217, UniRef:UniRef50_P07868, UniRef:UniRef90_P07868
SO:0001217, UniRef:UniRef50_A0A7W1XD49
GO:0005886, GO:0006865, GO:0009847, KEGG:K06289, RefSeq:WP_072183667.1, SO:0001217, UniParc:UPI0006A8F438, UniRef:UniRef100_UPI0006A8F438, UniRef:UniRef50_P07869, UniRef:UniRef90_P07869
KEGG:K06290, SO:0001217, UniRef:UniRef50_P07870, UniRef:UniRef90_G4P0L0
KEGG:K06290, SO:0001217, UniRef:UniRef50_P07870, UniRef:UniRef90_G4P0L0
BlastRules:WP_003243201, GO:0000160, GO:0003677, GO:0005737, GO:0006355, RefSeq:WP_079288356.1, SO:0001217, UniParc:UPI00086B1FA8, UniRef:UniRef100_UPI00086B1FA8, UniRef:UniRef50_Q7A2Q1, UniRef:
BlastRules:WP_003244464, EC:2.7.13.3, GO:0000155, GO:0005524, GO:0005886, GO:0046983, KEGG:K11617, RefSeq:WP_326198200.1, SO:0001217, UniParc:UPI002DB712F4, UniRef:UniRef100_UPI002DB712F4, UniRe
BlastRules:WP_003242605, GO:0005886, KEGG:K11622, RefSeq:WP_072565245.1, SO:0001217, UniParc:UPI00086F1383, UniRef:UniRef100_UPI00086F1383, UniRef:UniRef50_O32199, UniRef:UniRef90_O32199
SO:0001217, UniRef:UniRef50_O32200, UniRef:UniRef90_E0U1G8
SO:0001217, UniRef:UniRef50_O32201
BlastRules:WP_003243447, GO:0005886, RefSeq:WP_003221659.1, SO:0001217, UniParc:UPI0001D28B45, UniRef:UniRef100_A0A9Q4DNA4, UniRef:UniRef50_O32202, UniRef:UniRef90_O32202
GO:0005886, GO:0022857, KEGG:K08217, RefSeq:WP_061187956.1, SO:0001217, UniParc:UPI00077270E9, UniRef:UniRef100_A0A9Q4E6A3, UniRef:UniRef50_O32203, UniRef:UniRef90_O32203
EC:2.5.1.17, GO:0005524, GO:0005737, GO:0006779, GO:0008817, GO:0009236, SO:0001217, UniRef:UniRef50_O34899, UniRef:UniRef90_O34899
SO:0001217, UniParc:UPI00022BB447, UniRef:UniRef100_G4P0M0, UniRef:UniRef50_O34631, UniRef:UniRef90_E0U1H3
GO:0005886, GO:0022857, GO:0033214, SO:0001217, UniRef:UniRef50_O34451, UniRef:UniRef90_O34451
SO:0001217, UniRef:UniRef50_UPI002DBE3AF8
RFAM:RF00174, SO:0000035
EC:1.-.-.-, GO:0016491, RefSeq:WP_072181915.1, SO:0001217, UniParc:UPI0006A93034, UniRef:UniRef100_A0A9Q4HI26, UniRef:UniRef50_O34782, UniRef:UniRef90_O34782
EC:3.1.1.-, GO:0004341, GO:0005509, GO:0005737, GO:0019853, KEGG:K01053, RefSeq:WP_014115125.1, SO:0001217, UniParc:UPI00022BB44B, UniRef:UniRef100_G4P0M4, UniRef:UniRef50_O34940, UniRef:UniRef90
SO:0001217, UniRef:UniRef50_UPI002DB90C4D
BlastRules:WP_003243545, GO:0000156, GO:0000976, GO:0005829, GO:0006355, GO:0032993, SO:0001217, UniRef:UniRef50_P94504, UniRef:UniRef90_P94504
KEGG:K26990, RefSeq:WP_003221636.1, SO:0001217, UniParc:UPI0001D28B3B, UniRef:UniRef100_A0A9Q4HAM7, UniRef:UniRef50_C0H3R4, UniRef:UniRef90_G4P0M7
BlastRules:WP_003243707, KEGG:K26992, SO:0001217, UniRef:UniRef50_O34843, UniRef:UniRef90_A0A9W5LFN8
RefSeq:WP_003221632.1, SO:0001217, UniParc:UPI0001D28B39, UniRef:UniRef100_E0U1I2, UniRef:UniRef50_C0H3R5, UniRef:UniRef90_E0U1I2
BlastRules:WP_003243476, EC:4.1.1.2, GO:0005737, GO:0033609, GO:0046564, GO:0046872, KEGG:K01569, RefSeq:WP_014115129.1, SO:0001217, UniParc:UPI00022BB44F, UniRef:UniRef100_G4P0N0, UniRef:UniRef5
KEGG:K26991, RefSeq:WP_014115130.1, SO:0001217, UniParc:UPI00022BB450, UniRef:UniRef100_G4P0N1, UniRef:UniRef50_O34686, UniRef:UniRef90_G4P0N1
GO:0005886, GO:0022857, KEGG:K24948, SO:0001217, UniRef:UniRef50_P46324, UniRef:UniRef90_P46324
EC:7.-.-.-, GO:0005524, GO:0005886, GO:0016887, GO:0022857, GO:0055085, RefSeq:WP_014115132.1, SO:0001217, UniParc:UPI00022BB452, UniRef:UniRef100_G4P0N3, UniRef:UniRef50_O31711, UniRef:UniRef90_O
GO:0005886, KEGG:K24950, SO:0001217, UniRef:UniRef50_O35007, UniRef:UniRef90_O35007
EC:7.2.2.16, GO:0005524, GO:0005886, GO:0015625, GO:0016887, RefSeq:WP_061187946.1, SO:0001217, UniParc:UPI0006A93951, UniRef:UniRef100_A0A9Q4HFP7, UniRef:UniRef50_P49938, UniRef:UniRef90_P49938
GO:0005886, GO:0022857, GO:0033214, RefSeq:WP_014115135.1, SO:0001217, UniParc:UPI00022BB455, UniRef:UniRef100_G4P0N6, UniRef:UniRef50_P49937, UniRef:UniRef90_P49937
GO:0005886, GO:0022857, GO:0033214, GO:0045121, SO:0001217, UniRef:UniRef50_P49936, UniRef:UniRef90_P49936
GO:0005886, GO:0030288, GO:0045121, GO:1901678, RefSeq:WP_061187944.1, SO:0001217, UniParc:UPI0006A89A36, UniRef:UniRef100_UPI0006A89A36, UniRef:UniRef50_P37580, UniRef:UniRef90_P37580
GO:0005886, GO:0006865, GO:0015297, KEGG:K03758, RefSeq:WP_072181911.1, SO:0001217, UniParc:UPI0006A8CACB, UniRef:UniRef100_UPI0006A8CACB, UniRef:UniRef50_O32204, UniRef:UniRef90_O32204
RFAM:RF00168, SO:0000035
GO:0030435, GO:0030436, KEGG:K06427, RefSeq:WP_003240697.1, SO:0001217, UniParc:UPI00022BB5DC, UniRef:UniRef100_A0A2M8SVL0, UniRef:UniRef50_Q7WY58, UniRef:UniRef90_Q7WY58
GO:0005886, RefSeq:WP_061187941.1, SO:0001217, UniParc:UPI0006A8DCF4, UniRef:UniRef100_A0A9Q4E1I5, UniRef:UniRef50_O32205, UniRef:UniRef90_O32205
EC:2.7.8.20, GO:0005576, GO:0005886, GO:0046872, KEGG:K19005, RefSeq:WP_072181910.1, SO:0001217, UniParc:UPI0006A80457, UniRef:UniRef100_UPI0006A80457, UniRef:UniRef50_O32206, UniRef:UniRef90_O32
GO:0003677, SO:0001217, UniRef:UniRef50_O32207, UniRef:UniRef90_O32207
GO:0005886, GO:0015412, GO:0015689, GO:0030288, GO:0030973, GO:0046872, GO:0055052, RefSeq:WP_072181909.1, SO:0001217, UniParc:UPI0006A90531, UniRef:UniRef100_UPI0006A90531, UniRef:UniRef50_O32
GO:0005886, GO:0015412, GO:0015689, GO:0055052, SO:0001217, UniRef:UniRef50_A0A0U1P4K9, UniRef:UniRef90_A0A6H0WLJ5
BlastRules:WP_003243374, EC:1.1.1.-, EC:1.1.1.283, GO:0004033, GO:0043892, GO:0044281, RefSeq:WP_003221597.1, SO:0001217, UniParc:UPI0001D28B28, UniRef:UniRef100_A0A9Q4H9S3, UniRef:UniRef50_O32210
BlastRules:WP_003243913, SO:0001217, UniRef:UniRef50_O32211, UniRef:UniRef90_O32211
GO:0005886, GO:0006814, GO:0006885, GO:0015385, GO:0015386, GO:0016020, GO:0051453, GO:0071805, GO:0098719, GO:1902600, KEGG:K24163, RefSeq:WP_090557088.1, SO:0001217, UniParc:UPI000869173F, U
SO:0001217, UniRef:UniRef50_A0A6S6UIT2
EC:1.8.1.2, GO:0004783, GO:0005829, GO:0006790, GO:0009337, GO:0010181, GO:0016491, GO:0016651, GO:0019344, GO:0050660, GO:0070814, SO:0001217, UniRef:UniRef50_O32214, UniRef:UniRef90_O32214
SO:0001217, UniRef:UniRef50_UPI000B85D486
EC:3.6.4.12, GO:0000725, GO:0003677, GO:0003689, GO:0005524, GO:0005829, GO:0009378, GO:0016887, GO:0036121, GO:0043138, GO:0061749, GO:0061775, GO:0140584, GO:0140665, GO:0140849, GO:1990518,
SO:0001217, UniRef:UniRef50_A0A1X9MAC9
GO:0005886, GO:0006457, GO:0015035, GO:0030420, GO:0045121, RefSeq:WP_003235152.1, SO:0001217, UniParc:UPI00022BB5E9, UniRef:UniRef100_A0A2M8SVN6, UniRef:UniRef50_O32217, UniRef:UniRef90_O3221

GO:0005886, GO:0016491, GO:0030420, GO:0045121, RefSeq:WP_072565263.1, SO:0001217, UniParc:UPI00090A7323, UniRef:UniRef100_UPI00090A7323, UniRef:UniRef50_O32218, UniRef:UniRef90_O32218
EC:7.2.2.12, EC:7.2.2.21, GO:0005524, GO:0005886, GO:0006824, GO:0006825, GO:0008551, GO:0015086, GO:0016020, GO:0016463, GO:0016887, GO:0030001, GO:0046686, GO:0046872, GO:0055085, SO:0001217, U
SO:0001217, UniRef:UniRef50_A0A150LC89
EC:7.2.2.8, GO:0005507, GO:0005524, GO:0005886, GO:0016020, GO:0016887, GO:0042802, GO:0043682, GO:0055070, GO:0140581, KEGG:K17686, SO:0001217, UniRef:UniRef50_O32220, UniRef:UniRef90_O32220
PFAM:PF00403.32
GO:0005507, GO:0005737, GO:0006825, RefSeq:WP_014115156.1, SO:0001217, UniParc:UPI00022BB5ED, UniRef:UniRef100_G4P0R0, UniRef:UniRef50_O32221, UniRef:UniRef90_O32221
BlastRules:WP_003228404, BlastRules:WP_003404935, GO:0000976, GO:0001217, GO:0005737, GO:0032993, GO:0046872, KEGG:K21600, RefSeq:WP_014115157.1, SO:0001217, UniParc:UPI00022BB5EE, UniRef:UniRe
EC:1.1.1.371, GO:0005829, GO:0017000, GO:0070401, GO:0070402, GO:0102497, KEGG:K16044, SO:0001217, UniRef:UniRef50_O32223, UniRef:UniRef90_O32223
BlastRules:WP_003243587, EC:1.6.5.-, GO:0009055, GO:0010181, GO:0016652, GO:0016655, KEGG:K01118, SO:0001217, UniRef:UniRef50_O32224, UniRef:UniRef90_O32224
SO:0001217, UniRef:UniRef50_O32225, UniRef:UniRef90_E0U261
SO:0001217, UniRef:UniRef50_G4P0R5, UniRef:UniRef90_G4P0R5
SO:0001217, UniRef:UniRef50_A0A2X1A342, UniRef:UniRef90_G4P0R6
SO:0001217, UniRef:UniRef50_A0A160F4H0
GO:0005886, RefSeq:WP_014115164.1, SO:0001217, UniParc:UPI00022BB5F5, UniRef:UniRef100_G4P0R8, UniRef:UniRef50_O32226, UniRef:UniRef90_O32226
GO:0005886, GO:0022857, KEGG:K03297, RefSeq:WP_014115165.1, SO:0001217, UniParc:UPI00022BB5F6, UniRef:UniRef100_G4P0R9, UniRef:UniRef50_O32227, UniRef:UniRef90_O32227
GO:0000976, GO:0003700, GO:0006355, RefSeq:WP_061187926.1, SO:0001217, UniParc:UPI0007726580, UniRef:UniRef100_A0A9Q4E4U9, UniRef:UniRef50_O32228, UniRef:UniRef90_O32228
EC:1.-.-.-, GO:0016491, RefSeq:WP_326198567.1, SO:0001217, UniParc:UPI002DC032EE, UniRef:UniRef100_UPI002DC032EE, UniRef:UniRef50_O32229, UniRef:UniRef90_O32229
SO:0000584
SO:0001217, UniRef:UniRef50_Q81XA8
EC:3.1.13.1, GO:0003723, GO:0004518, GO:0005829, GO:0006402, GO:0008859, SO:0001217, UniRef:UniRef50_O32231, UniRef:UniRef90_O32231
EC:3.1.1.1, GO:0016020, GO:0016298, GO:0106435, SO:0001217, UniRef:UniRef50_O32232, UniRef:UniRef90_O32232
GO:0005886, GO:0009306, GO:0015136, GO:0015450, GO:0015739, GO:0016020, GO:0043952, GO:0065002, RefSeq:WP_003220028.1, SO:0001217, UniParc:UPI0000031A3F, UniRef:UniRef100_O32233, UniRef:UniRef50
RefSeq:WP_061187923.1, SO:0001217, UniParc:UPI00077297B9, UniRef:UniRef100_UPI0006A93F53, UniRef:UniRef50_O32234, UniRef:UniRef90_G4P0S7
SO:0001217, UniRef:UniRef50_O32235, UniRef:UniRef90_A0AAI8HSG1
BlastRules:WP_003220034, GO:0003677, GO:0003700, GO:0006355, SO:0001217, UniRef:UniRef50_O32236, UniRef:UniRef90_O32236
RefSeq:WP_061187922.1, SO:0001217, UniParc:UPI0006A8AD4F, UniRef:UniRef100_UPI0006A8AD4F, UniRef:UniRef50_O32236, UniRef:UniRef90_G4P0T0
BlastRules:WP_003242888, GO:0000976, GO:0001217, GO:0032993, GO:0045892, RefSeq:WP_003220037.1, SO:0001217, UniParc:UPI00000BCAB6, UniRef:UniRef100_O52854, UniRef:UniRef50_O32238, UniRef:UniRef9
GO:0004888, GO:0005886, GO:0006935, GO:0007165, SO:0001217, UniRef:UniRef50_O32239, UniRef:UniRef90_O32239
SO:0001217, UniRef:UniRef50_UPI00202910E4, UniRef:UniRef90_UPI0001F5BA73
RefSeq:WP_041521070.1, SO:0001217, UniParc:UPI0005A0D058, UniRef:UniRef100_G0Z2I0, UniRef:UniRef50_P33113, UniRef:UniRef90_G0Z2I0
COG:COG0745, COG:TK, GO:0000156, GO:0000976, GO:0005829, GO:0006355, GO:0032993, RefSeq:WP_014115176.1, SO:0001217, UniParc:UPI000219FA41, UniRef:UniRef100_G0Z2H9, UniRef:UniRef50_P33112, UniF
KEGG:K20492, RefSeq:WP_079288388.1, SO:0001217, UniParc:UPI0009A2E969, UniRef:UniRef100_UPI0009A2E969, UniRef:UniRef50_Q65NU1, UniRef:UniRef90_G0Z2H8
KEGG:K20491, RefSeq:WP_061187918.1, SO:0001217, UniParc:UPI000772912A, UniRef:UniRef100_UPI000772912A, UniRef:UniRef50_Q65NU2, UniRef:UniRef90_G0Z2H7
COG:COG1131, COG:JV, RefSeq:WP_014115179.1, SO:0001217, UniParc:UPI00022BB758, UniRef:UniRef100_G0Z2H6, UniRef:UniRef50_P0C0E3, UniRef:UniRef90_A0A0D1KHK3
RefSeq:WP_072181750.1, SO:0001217, UniParc:UPI0006A85681, UniRef:UniRef100_UPI0006A85681, UniRef:UniRef50_E0U281, UniRef:UniRef90_E0U281
BlastRules:WP_003228374, GO:0005886, GO:0006865, GO:0055085, RefSeq:WP_014115185.1, SO:0001217, UniParc:UPI00022BB75B, UniRef:UniRef100_G4P0U3, UniRef:UniRef50_P39775, UniRef:UniRef90_P39775
BlastRules:WP_003228372, GO:0006865, GO:0022857, GO:0043190, RefSeq:WP_072181751.1, SO:0001217, UniParc:UPI0006A8DBCE, UniRef:UniRef100_UPI0006A8DBCE, UniRef:UniRef50_Q45462, UniRef:UniRef90_Q
BlastRules:WP_003228370, GO:0005886, GO:0006865, GO:0031460, GO:0055085, RefSeq:WP_072181752.1, SO:0001217, UniParc:UPI0006A8BF4E, UniRef:UniRef100_UPI0006A8BF4E, UniRef:UniRef50_Q45461, UniRef:
BlastRules:WP_003242811, GO:0005524, GO:0006865, GO:0016020, GO:0016887, GO:0031460, SO:0001217, UniRef:UniRef50_Q45460, UniRef:UniRef90_Q45460
GO:0003677, GO:0003700, SO:0001217, UniRef:UniRef50_O34709, UniRef:UniRef90_C0SPB8
BlastRules:WP_003244403, GO:0005886, GO:0006865, GO:0055085, RefSeq:WP_072183253.1, SO:0001217, UniParc:UPI0006A84CB5, UniRef:UniRef100_A0A9Q4E8F0, UniRef:UniRef50_P39775, UniRef:UniRef90_O3474
BlastRules:WP_003228350, GO:0006865, GO:0022857, GO:0043190, SO:0001217, UniRef:UniRef50_Q45462, UniRef:UniRef90_O32243
BlastRules:WP_003228349, RefSeq:WP_061187913.1, SO:0001217, UniParc:UPI0006A7FF28, UniRef:UniRef100_UPI0006A7FF28, UniRef:UniRef50_R2T098, UniRef:UniRef90_A0A6H0WMI3
BlastRules:WP_003243370, GO:0005524, GO:0006865, GO:0016020, GO:0016887, GO:0031460, RefSeq:WP_014115193.1, SO:0001217, UniParc:UPI00022BB763, UniRef:UniRef100_G4P0V2, UniRef:UniRef50_Q45460, Ur
BlastRules:WP_003244093, GO:0003677, GO:0003700, RefSeq:WP_014115194.1, SO:0001217, UniParc:UPI00022BB764, UniRef:UniRef100_G4P0V3, UniRef:UniRef50_O34709, UniRef:UniRef90_O34709
SO:0001217, UniRef:UniRef50_E0U2U2, UniRef:UniRef90_G4P0V4
GO:0005886, SO:0001217, UniRef:UniRef50_O32246, UniRef:UniRef90_O32246
PFAM:PF22819.2
SO:0001217, UniRef:UniRef50_O32247, UniRef:UniRef90_A0A7G7UFG2
SO:0001217, UniRef:UniRef50_A8MKP1, UniRef:UniRef90_D4G1L1
EC:4.2.1.11, GO:0000015, GO:0000287, GO:0004634, GO:0005576, GO:0006096, GO:0009986, KEGG:K01689, RefSeq:WP_007409956.1, SO:0001217, UniParc:UPI0001597854, UniRef:UniRef100_A7Z8Y1, UniRef:UniRef5
BlastRules:WP_001231153, BlastRules:WP_003228330, EC:5.4.2.12, GO:0005829, GO:0006007, GO:0006096, GO:0030145, GO:0030435, GO:0046537, KEGG:K15633, SO:0001217, UniRef:UniRef50_Q9X519, UniRef:UniRe
COG:COG0149, COG:G, EC:5.3.1.1, GO:0004807, GO:0005829, GO:0006094, GO:0006096, GO:0019563, GO:0046166, KEGG:K01803, SO:0001217, UniRef:UniRef50_P27876, UniRef:UniRef90_P27876
SO:0001217, UniRef:UniRef50_A0A920WBG1
EC:2.7.2.3, GO:0004618, GO:0005524, GO:0005829, GO:0006094, GO:0006096, GO:0043531, KEGG:K00927, SO:0001217, UniRef:UniRef50_P18912, UniRef:UniRef90_P40924
COG:COG0057, COG:G, EC:1.2.1.12, GO:0004365, GO:0005737, GO:0006006, GO:0006096, GO:0016620, GO:0050661, GO:0051287, SO:0001217, UniRef:UniRef50_P09124, UniRef:UniRef90_P09124
BlastRules:WP_001258187, BlastRules:WP_009968188, GO:0003677, GO:0030246, KEGG:K05311, RefSeq:WP_014115202.1, SO:0001217, UniParc:UPI00022BB76D, UniRef:UniRef100_G4P0W5, UniRef:UniRef50_O32253
BlastRules:WP_000256438, BlastRules:WP_003243899, GO:0005886, GO:0015293, GO:0016020, GO:0055085, RefSeq:WP_072183257.1, SO:0001217, UniParc:UPI0006A8600A, UniRef:UniRef100_UPI0006A8600A, UniRef
BlastRules:WP_003243070, GO:0000976, GO:0003700, GO:0005737, GO:0006355, SO:0001217, UniRef:UniRef50_P96711, UniRef:UniRef90_P96711
SO:0001217, UniRef:UniRef50_W4QAE8
PFAM:PF00126.33
KEGG:K15269, SO:0001217, UniRef:UniRef50_O32256, UniRef:UniRef90_UPI0035CA2A65
GO:0003333, GO:0005886, GO:0015171, GO:0016020, RefSeq:WP_014115210.1, SO:0001217, UniParc:UPI00022BB8D8, UniRef:UniRef100_G4P0X3, UniRef:UniRef50_O32257, UniRef:UniRef90_O32257
RFAM:RF00230, SO:0000140
SO:0001217, UniRef:UniRef50_D4G1M5, UniRef:UniRef90_G4P0X4
SO:0001217, UniRef:UniRef50_UPI00283B35F9, UniRef:UniRef90_UPI00283B35F9
RFAM:RF01458, SO:0000655
SO:0001217, UniRef:UniRef50_A0A075R5J2
SO:0001217, UniRef:UniRef50_G4P0X7, UniRef:UniRef90_G4P0X7
BlastRules:WP_003228309, GO:0006089, SO:0001217, UniRef:UniRef50_O32259, UniRef:UniRef90_O32259
BlastRules:WP_003228307, GO:0006089, GO:0046872, GO:0051536, GO:0051539, KEGG:K18929, SO:0001217, UniRef:UniRef50_O07021, UniRef:UniRef90_O07021
BlastRules:WP_003244353, GO:0005829, GO:0006089, GO:0016491, KEGG:K18928, RefSeq:WP_072183260.1, SO:0001217, UniParc:UPI0006A8B8A9, UniRef:UniRef100_UPI0006A8B8A9, UniRef:UniRef50_O07020, UniRe
RefSeq:WP_003219931.1, SO:0001217, UniParc:UPI0001D287BD, UniRef:UniRef100_A0A9Q4HIC1, UniRef:UniRef50_G4P0Y2, UniRef:UniRef90_G4P0Y2
RefSeq:WP_014115218.1, SO:0001217, UniParc:UPI00022BB8E0, UniRef:UniRef100_G4P0Y3, UniRef:UniRef50_Q8DX26, UniRef:UniRef90_G4P0Y3
SO:0001217, UniRef:UniRef50_A0AB33AS41
SO:0001217, UniRef:UniRef50_A0A941FR08
EC:2.7.13.3, KEGG:K07778, RefSeq:WP_061187901.1, SO:0001217, UniParc:UPI0007722D19, UniRef:UniRef100_UPI0007722D19, UniRef:UniRef50_G4P0Y5, UniRef:UniRef90_G4P0Y5
GO:0043190, GO:0140359, RefSeq:WP_003219923.1, SO:0001217, UniParc:UPI0001D286EA, UniRef:UniRef100_G4P0Y6, UniRef:UniRef50_O07017, UniRef:UniRef90_O07017
SO:0001217, UniRef:UniRef50_O07016, UniRef:UniRef90_UPI001C934165
SO:0001217, UniRef:UniRef50_A0A6M8BFL3, UniRef:UniRef90_A0A5D4N910
RefSeq:WP_326197875.1, SO:0001217, UniParc:UPI002DB7F149, UniRef:UniRef100_UPI002DB7F149, UniRef:UniRef50_O07014, UniRef:UniRef90_G4P0Y9
EC:3.2.1.89, KEGG:K01224, SO:0001217, UniRef:UniRef50_Q65CX5, UniRef:UniRef90_UPI000379309B
BlastRules:WP_003243532, EC:3.2.1.23, GO:0004565, GO:0006012, GO:0009341, GO:0046872, RefSeq:WP_014115225.1, SO:0001217, UniParc:UPI00022BB8E7, UniRef:UniRef100_G4P0Z1, UniRef:UniRef50_O07012, Uni
BlastRules:WP_003228283, GO:0005886, GO:0015423, GO:0015768, GO:0042956, RefSeq:WP_014115226.1, SO:0001217, UniParc:UPI00022BB8E8, UniRef:UniRef100_G4P0Z2, UniRef:UniRef50_O07011, UniRef:UniRef9
BlastRules:WP_003243623, GO:0015423, GO:0042956, GO:1990060, RefSeq:WP_061187895.1, SO:0001217, UniParc:UPI00077268CC, UniRef:UniRef100_UPI00077268CC, UniRef:UniRef50_O32261, UniRef:UniRef90_O32
BlastRules:WP_003228280, SO:0001217, UniRef:UniRef50_O07009, UniRef:UniRef90_UPI0006A91FDA
BlastRules:WP_003244199, GO:0000976, GO:0003700, GO:0006355, SO:0001217, UniRef:UniRef50_O07008, UniRef:UniRef90_O07008
SO:0001217, UniRef:UniRef50_UPI002DB9B498, UniRef:UniRef90_UPI002DB9B498
PFAM:PF00392.27, PFAM:PF01022.26, PFAM:PF08279.18
BlastRules:WP_003228275, GO:0005886, GO:0015129, GO:0015295, KEGG:K03303, RefSeq:WP_072182607.1, SO:0001217, UniParc:UPI0006A8FA8A, UniRef:UniRef100_UPI0006A8FA8A, UniRef:UniRef50_P33231, UniRe
GO:0000428, GO:0001216, GO:0003677, GO:0006352, GO:0016779, GO:0016987, RefSeq:WP_072182608.1, SO:0001217, UniParc:UPI0006A81272, UniRef:UniRef100_UPI0006A81272, UniRef:UniRef50_P24219, UniRef:U
RefSeq:WP_003219905.1, SO:0001217, UniParc:UPI0000060AA6, UniRef:UniRef100_P71066, UniRef:UniRef50_P71066, UniRef:UniRef90_P71066
EC:2.-.-.-, GO:0000271, GO:0016740, RefSeq:WP_072182609.1, SO:0001217, UniParc:UPI0006A8B41D, UniRef:UniRef100_UPI0006A8B41D, UniRef:UniRef50_P71065, UniRef:UniRef90_P71065
SO:0001217, UniRef:UniRef50_A0A8J2ZVI6
EC:2.-.-.-, GO:0000271, GO:0005886, GO:0016780, KEGG:K19428, SO:0001217, UniRef:UniRef50_P71062, UniRef:UniRef90_P71062
GO:0000271, GO:0005886, KEGG:K19418, SO:0001217, UniRef:UniRef50_P71060, UniRef:UniRef90_P71060
SO:0001217, UniRef:UniRef50_P71059, UniRef:UniRef90_A0A9Q4EQT4
SO:0001217, UniRef:UniRef50_UPI0022EA7488
EC:2.-.-.-, KEGG:K19426, SO:0001217, UniRef:UniRef50_P71058, UniRef:UniRef90_A0A6H0WNE8
EC:2.4.-.-, GO:0000271, GO:0016757, SO:0001217, UniRef:UniRef50_P71057, UniRef:UniRef90_P71057
GO:0000271, GO:0005886, KEGG:K19419, SO:0001217, UniRef:UniRef50_P71056, UniRef:UniRef90_P71056
PFAM:PF13692.12
EC:2.4.-.-, GO:0000271, GO:0016757, SO:0001217, UniRef:UniRef50_P71055, UniRef:UniRef90_P71055
PFAM:PF00535.33, PFAM:PF13641.12
EC:2.4.-.-, GO:0000271, GO:0016757, SO:0001217, UniRef:UniRef50_P71053, UniRef:UniRef90_P71053
SO:0001217, UniRef:UniRef50_P71052
RFAM:RF01735, SO:0005836
BlastRules:WP_003228249, EC:2.7.10.2, GO:0004715, GO:0005524, GO:0035401, GO:0045226, GO:0140801, RefSeq:WP_072182617.1, SO:0001217, UniParc:UPI0006A90443, UniRef:UniRef100_UPI0006A90443, UniRef:U
GO:0004713, GO:0005886, RefSeq:WP_072182618.1, SO:0001217, UniParc:UPI0006A8683A, UniRef:UniRef100_UPI0006A8683A, UniRef:UniRef50_P71050, UniRef:UniRef90_P71050
BlastRules:WP_003242589, GO:0003677, GO:0003700, GO:0006355, GO:0046983, KEGG:K19417, RefSeq:WP_072182619.1, SO:0001217, UniParc:UPI0006A92DC9, UniRef:UniRef100_UPI0006A92DC9, UniRef:UniRef50_
EC:3.1.1.-, GO:0004104, RefSeq:WP_088679717.1, SO:0001217, UniParc:UPI000B53771D, UniRef:UniRef100_UPI000B53771D, UniRef:UniRef50_P37967, UniRef:UniRef90_P37967
RefSeq:WP_072182621.1, SO:0001217, UniParc:UPI0006A911FC, UniRef:UniRef100_UPI0006A911FC, UniRef:UniRef50_A0A2K4Z9N5, UniRef:UniRef90_E0U2Z7
BlastRules:WP_003243190, EC:4.1.1.102, GO:0009056, GO:0009636, GO:0016831, KEGG:K13727, RefSeq:WP_003235304.1, SO:0001217, UniParc:UPI00022BBA92, UniRef:UniRef100_A0A2M8SV63, UniRef:UniRef50_O0
RefSeq:WP_014115249.1, SO:0001217, UniParc:UPI00022BBA93, UniRef:UniRef100_G4P120, UniRef:UniRef50_O07005, UniRef:UniRef90_O07005
BlastRules:WP_003244427, EC:5.1.1.10, GO:0008784, GO:0008837, GO:0018111, GO:0018113, GO:0030378, GO:0030435, GO:0047679, GO:0050157, RefSeq:WP_072182622.1, SO:0001217, UniParc:UPI0006A81946, Un
RefSeq:WP_072182623.1, SO:0001217, UniParc:UPI0006A8CFAF, UniRef:UniRef100_UPI0006A8CFAF, UniRef:UniRef50_P32959, UniRef:UniRef90_G4P122
RefSeq:WP_072182624.1, SO:0001217, UniParc:UPI0006A861AD, UniRef:UniRef100_UPI0006A861AD, UniRef:UniRef50_A0A2M8SV41, UniRef:UniRef90_A0A2M8SV41
BlastRules:WP_001022105, EC:2.4.1.10, GO:0005576, GO:0009758, GO:0046872, GO:0050053, KEGG:K00692, RefSeq:WP_072182625.1, SO:0001217, UniParc:UPI0006A8E16D, UniRef:UniRef100_UPI0006A8E16D, UniRe
EC:3.2.1.64, GO:0004575, GO:0005737, GO:0005886, GO:0005987, GO:0033912, RefSeq:WP_072182626.1, SO:0001217, UniParc:UPI0006A8E0E8, UniRef:UniRef100_UPI0006A8E0E8, UniRef:UniRef50_O07003, UniRef:Ur
BlastRules:WP_003228228, GO:0005886, GO:0006865, GO:0015293, RefSeq:WP_014115255.1, SO:0001217, UniParc:UPI00022BBA99, UniRef:UniRef100_G4P126, UniRef:UniRef50_O07002, UniRef:UniRef90_O07002
GO:0003677, SO:0001217, UniRef:UniRef50_O07001, UniRef:UniRef90_O07001
GO:0005886, GO:0022857, GO:0055085, RefSeq:WP_014115257.1, SO:0001217, UniParc:UPI00022BBA9B, UniRef:UniRef100_G4P128, UniRef:UniRef50_O32262, UniRef:UniRef90_O32262
GO:0005886, GO:0022857, GO:0055085, RefSeq:WP_014115258.1, SO:0001217, UniParc:UPI00022BBA9C, UniRef:UniRef100_G4P129, UniRef:UniRef50_O06999, UniRef:UniRef90_O06999
RefSeq:WP_014115259.1, SO:0001217, UniParc:UPI00022BBA9D, UniRef:UniRef100_G4P130, UniRef:UniRef50_O06998, UniRef:UniRef90_O06998
SO:0001036
COG:COG0740, COG:O, EC:3.4.21.92, GO:0004176, GO:0004252, GO:0005737, GO:0006508, GO:0006515, GO:0009368, GO:0051117, KEGG:K01358, RefSeq:WP_003219849.1, SO:0001217, UniParc:UPI0001D285F6, Un
EC:5.4.2.6, GO:0000287, GO:0005975, GO:0008801, SO:0001217, UniRef:UniRef50_O06995, UniRef:UniRef90_G4P132
EC:3.2.1.10, GO:0004556, GO:0004574, GO:0005737, GO:0009313, GO:0046872, RefSeq:WP_072182629.1, SO:0001217, UniParc:UPI0006A90200, UniRef:UniRef100_UPI0006A90200, UniRef:UniRef50_O06994, UniRef:Ur
EC:2.4.1.8, KEGG:K00691, SO:0001217, UniRef:UniRef50_E6ENP7, UniRef:UniRef90_G4P134
SO:0001217, UniRef:UniRef50_O06992, UniRef:UniRef90_G4P135
BlastRules:WP_003242930, COG:COG3833, COG:G, RefSeq:WP_072182632.1, SO:0001217, UniParc:UPI0006A8518B, UniRef:UniRef100_UPI0006A8518B, UniRef:UniRef50_O06991, UniRef:UniRef90_D4G1U9
BlastRules:WP_003243114, GO:0015423, GO:0042956, GO:1990060, RefSeq:WP_014115266.1, SO:0001217, UniParc:UPI00022BBAA4, UniRef:UniRef100_G4P137, UniRef:UniRef50_O06990, UniRef:UniRef90_O06990
BlastRules:WP_003243721, GO:0015144, GO:0015768, GO:0042956, GO:0055052, GO:1901982, SO:0001217, UniRef:UniRef50_P59213, UniRef:UniRef90_O06989

SO:0001217, UniRef:UniRef50_A0A4P5PGZ2, UniRef:UniRef90_UPI0020917058
GO:0000976, GO:0003700, GO:0006355, RefSeq:WP_014115269.1, SO:0001217, UniParc:UPI00022BBAA7, UniRef:UniRef100_G4P140, UniRef:UniRef50_O06987, UniRef:UniRef90_O06987
EC:3.2.2.-, GO:0005829, GO:0009691, GO:0102682, KEGG:K22522, RefSeq:WP_014115270.1, SO:0001217, UniParc:UPI00022BBAA8, UniRef:UniRef100_G4P141, UniRef:UniRef50_O06986, UniRef:UniRef90_O06986
SO:0001217, UniParc:UPI00022BBAA9, UniRef:UniRef100_G4P142, UniRef:UniRef50_O32263, UniRef:UniRef90_O32263
GO:0005886, GO:0055085, KEGG:K03321, RefSeq:WP_014115272.1, SO:0001217, UniParc:UPI00022BBC38, UniRef:UniRef100_G4P143, UniRef:UniRef50_O06984, UniRef:UniRef90_O06984
EC:4.2.1.1, GO:0004089, GO:0008270, KEGG:K01673, SO:0001217, UniParc:UPI00022BBC39, UniRef:UniRef100_A0A9Q4E5J5, UniRef:UniRef50_O34872, UniRef:UniRef90_O06983
PFAM:PF00389.36
SO:0001217, UniRef:UniRef50_A0A2X3LPC9
GO:0005886, GO:0055085, RefSeq:WP_088679713.1, SO:0001217, UniParc:UPI000B52E5D6, UniRef:UniRef100_UPI000B52E5D6, UniRef:UniRef50_O06981, UniRef:UniRef90_O06981
BlastRules:WP_003228167, EC:7.-.-.-, GO:0005524, GO:0005886, GO:0016887, GO:0022857, GO:0055085, RefSeq:WP_072183372.1, SO:0001217, UniParc:UPI0006A8745E, UniRef:UniRef100_UPI0006A8745E, UniRef:Uni
EC:2.7.13.3, GO:0000155, GO:0000160, GO:0005524, GO:0005886, GO:0009927, KEGG:K11633, RefSeq:WP_061187864.1, SO:0001217, UniParc:UPI0006A8A0C2, UniRef:UniRef100_A0A9Q4HGU4, UniRef:UniRef50_O06
BlastRules:WP_003244535, GO:0000156, GO:0000976, GO:0005829, GO:0006355, GO:0032993, KEGG:K11634, RefSeq:WP_072183373.1, SO:0001217, UniParc:UPI0006A8E2DC, UniRef:UniRef100_UPI0006A8E2DC, UniI
GO:0005886, RefSeq:WP_088679712.1, SO:0001217, UniParc:UPI0006A8F111, UniRef:UniRef100_UPI0006A8F111, UniRef:UniRef50_C0H3R7, UniRef:UniRef90_C0H3R7
RefSeq:WP_088679711.1, SO:0001217, UniParc:UPI000B52BA75, UniRef:UniRef100_UPI000B52BA75, UniRef:UniRef50_O06977, UniRef:UniRef90_G4P151
RefSeq:WP_003219835.1, SO:0001217, UniParc:UPI0000060ADB, UniRef:UniRef100_O06976, UniRef:UniRef50_O06976, UniRef:UniRef90_O06976
COG:COG1481, COG:K, GO:0003677, GO:0043937, GO:0051301, RefSeq:WP_003219831.1, SO:0001217, UniParc:UPI0001D285EA, UniRef:UniRef100_A0A9Q4E5T8, UniRef:UniRef50_O06975, UniRef:UniRef90_O06975
BlastRules:WP_003244450, GO:0005737, GO:0008360, GO:0043743, SO:0001217, UniRef:UniRef50_O06974, UniRef:UniRef90_O06974
GO:0005524, GO:0005525, KEGG:K06958, SO:0001217, UniRef:UniRef50_O06973, UniRef:UniRef90_O06973
EC:3.6.-.-, GO:0016787, GO:0046872, SO:0001217, UniRef:UniRef50_O06972, UniRef:UniRef90_O06972
EC:1.8.1.9, GO:0004791, GO:0005737, GO:0019430, GO:0045454, GO:0071949, RefSeq:WP_003228139.1, SO:0001217, UniParc:UPI00022BBC45, UniRef:UniRef100_A0A0D1KBN7, UniRef:UniRef50_P80880, UniRef:UniRe
SO:0001217, UniRef:UniRef50_P40767
RefSeq:WP_072182992.1, SO:0001217, UniParc:UPI0006A90462, UniRef:UniRef100_UPI0006A90462, UniRef:UniRef50_O06968, UniRef:UniRef90_O06968
BlastRules:WP_003243345, EC:7.6.2.-, GO:0005524, GO:0005886, GO:0016887, GO:0034040, GO:0046677, GO:0055085, GO:0140359, RefSeq:WP_072182990.1, SO:0001217, UniParc:UPI0006A84F54, UniRef:UniRef100_
RefSeq:WP_014115289.1, SO:0001217, UniParc:UPI00022BBC4A, UniRef:UniRef100_G4P163, UniRef:UniRef50_O32265, UniRef:UniRef90_O32265
SO:0001217, UniRef:UniRef50_A0AAP1E9N3, UniRef:UniRef90_UPI00192CA34F
EC:3.5.4.19, EC:3.6.1.31, GO:0000105, GO:0004635, GO:0004636, GO:0005524, GO:0005737, RefSeq:WP_072182571.1, SO:0001217, UniParc:UPI0006A8B746, UniRef:UniRef100_UPI0006A8B746, UniRef:UniRef50_O349
EC:4.3.2.10, GO:0000105, GO:0000107, GO:0005737, GO:0016829, RefSeq:WP_044454616.1, SO:0001217, UniParc:UPI0005CB1615, UniRef:UniRef100_UPI0005CB1615, UniRef:UniRef50_O34727, UniRef:UniRef90_O347
EC:5.3.1.16, GO:0000105, GO:0000162, GO:0003949, GO:0005737, RefSeq:WP_014115294.1, SO:0001217, UniParc:UPI00022BBC4F, UniRef:UniRef100_G4P168, UniRef:UniRef50_O35006, UniRef:UniRef90_O35006
EC:4.3.2.10, GO:0000105, GO:0000107, GO:0004359, GO:0005737, GO:0016829, RefSeq:WP_072182569.1, SO:0001217, UniParc:UPI0006A8736C, UniRef:UniRef100_UPI0006A8736C, UniRef:UniRef50_O34565, UniRef:U
EC:4.2.1.19, GO:0000105, GO:0004424, GO:0005737, SO:0001217, UniRef:UniRef50_O34683, UniRef:UniRef90_O34683
SO:0001217, UniRef:UniRef50_A0A7X8MLJ5
SO:0001217, UniRef:UniRef50_A0A920K4M3
EC:2.4.2.17, GO:0000105, GO:0003879, GO:0005524, GO:0005737, KEGG:K00765, RefSeq:WP_041521078.1, SO:0001217, UniParc:UPI0005A19CA4, UniRef:UniRef100_G4P172, UniRef:UniRef50_O34520, UniRef:UniRef9
PFAM:PF13393.11, PFAM:PF21996.1
GO:0000105, GO:0004821, GO:0005737, GO:0006427, GO:0016740, SO:0001217, UniRef:UniRef50_Q9K6Z0, UniRef:UniRef90_O34459
RefSeq:WP_072182567.1, SO:0001217, UniParc:UPI0006A92840, UniRef:UniRef100_UPI0006A92840, UniRef:UniRef50_O34735, UniRef:UniRef90_O34735
EC:4.2.2.2, GO:0005576, GO:0030570, GO:0045490, GO:0046872, GO:0047490, RefSeq:WP_088679708.1, SO:0001217, UniParc:UPI000B5313C7, UniRef:UniRef100_UPI000B5313C7, UniRef:UniRef50_O34310, UniRef:Un
EC:2.3.1.-, GO:0016746, RefSeq:WP_088679707.1, SO:0001217, UniParc:UPI000B535A91, UniRef:UniRef100_UPI000B535A91, UniRef:UniRef50_O34993, UniRef:UniRef90_O34993
EC:3.6.1.1, GO:0000287, GO:0004427, GO:0005829, GO:0006281, GO:0008967, RefSeq:WP_088679706.1, SO:0001217, UniParc:UPI000B5337D5, UniRef:UniRef100_UPI000B5337D5, UniRef:UniRef50_Q9JMQ2, UniRef:U
GO:0005886, RefSeq:WP_072182564.1, SO:0001217, UniParc:UPI0006A842B1, UniRef:UniRef100_UPI0006A842B1, UniRef:UniRef50_O34382, UniRef:UniRef90_O34382
EC:2.5.1.145, GO:0005886, GO:0008961, GO:0009249, GO:0016020, GO:0042158, RefSeq:WP_072182563.1, SO:0001217, UniParc:UPI0006A80B47, UniRef:UniRef100_UPI0006A80B47, UniRef:UniRef50_O34752, UniRef:U
EC:2.7.11.-, EC:2.7.4.-, GO:0000155, GO:0000160, GO:0000287, GO:0004674, GO:0004712, GO:0005524, GO:0006109, KEGG:K06023, RefSeq:WP_014115306.1, SO:0001217, UniParc:UPI00022BBC5B, UniRef:UniRef100
EC:3.5.1.25, GO:0005506, GO:0006044, GO:0006046, GO:0008448, GO:0019262, GO:0042803, KEGG:K01443, SO:0001217, UniRef:UniRef50_O34450, UniRef:UniRef90_O34450
EC:3.5.99.6, GO:0004342, GO:0005737, GO:0005975, GO:0006043, GO:0006044, GO:0006046, GO:0019262, GO:0042802, SO:0001217, UniRef:UniRef50_O35000, UniRef:UniRef90_O35000
BlastRules:WP_003228089, GO:0003677, GO:0003700, GO:0045892, SO:0001217, UniRef:UniRef50_O34817, UniRef:UniRef90_O34817
GO:0016787, SO:0001217, UniRef:UniRef50_O34986, UniRef:UniRef90_O34986
SO:0001217, UniRef:UniRef50_O34692, UniRef:UniRef90_E0U3N0
BlastRules:WP_003244436, EC:1.14.15.13, GO:0005506, GO:0016713, GO:0020037, GO:0046148, KEGG:K17474, RefSeq:WP_072182559.1, SO:0001217, UniParc:UPI0006A8B0AC, UniRef:UniRef100_UPI0006A8B0AC, Ur
EC:2.3.2.22, GO:0016755, GO:0046148, KEGG:K17485, RefSeq:WP_072182558.1, SO:0001217, UniParc:UPI00086BC104, UniRef:UniRef100_UPI00086BC104, UniRef:UniRef50_Q65EX3, UniRef:UniRef90_O34351
BlastRules:WP_003228073, GO:0003677, GO:0003700, GO:0045892, GO:0045893, RefSeq:WP_014115315.1, SO:0001217, UniParc:UPI00022BA46D, UniRef:UniRef100_G4P189, UniRef:UniRef50_P40762, UniRef:UniRef9
SO:0001217, UniRef:UniRef50_O34307, UniRef:UniRef90_UPI001C049F24
GO:0005886, KEGG:K08972, RefSeq:WP_003219771.1, SO:0001217, UniParc:UPI0001D28506, UniRef:UniRef100_G4P191, UniRef:UniRef50_O34648, UniRef:UniRef90_O34648
SO:0001217, UniRef:UniRef50_O34719
GO:0005886, RefSeq:WP_061187838.1, SO:0001217, UniParc:UPI0006A86205, UniRef:UniRef100_A0A9Q4EBZ8, UniRef:UniRef50_O34628, UniRef:UniRef90_O34628
GO:0005886, RefSeq:WP_003219765.1, SO:0001217, UniParc:UPI0001D28503, UniRef:UniRef100_G4P194, UniRef:UniRef50_O34322, UniRef:UniRef90_O34322
RefSeq:WP_003219764.1, SO:0001217, UniParc:UPI0001D28502, UniRef:UniRef100_A0A9Q4E7S8, UniRef:UniRef50_O34604, UniRef:UniRef90_O34604
EC:3.1.25.-, GO:0003677, GO:0005524, GO:0005737, GO:0006289, GO:0008270, GO:0009380, GO:0009381, GO:0009432, GO:0016887, KEGG:K03701, RefSeq:WP_014115320.1, SO:0001217, UniParc:UPI00022BA492, U
COG:COG0556, COG:L, GO:0003677, GO:0004386, GO:0005524, GO:0005737, GO:0006289, GO:0009380, GO:0009381, GO:0009432, GO:0016887, KEGG:K03702, RefSeq:WP_014115321.1, SO:0001217, UniParc:UPI00
GO:0005886, RefSeq:WP_003228053.1, SO:0001217, UniParc:UPI0000060B07, UniRef:UniRef100_P37953, UniRef:UniRef50_P37953, UniRef:UniRef90_P37953
SO:0001217, UniRef:UniRef50_UPI000AEB14A7, UniRef:UniRef90_UPI000AEB14A7
GO:0005524, GO:0016301, SO:0001217, UniRef:UniRef50_O34796, UniRef:UniRef90_O34796
SO:0001217, UniRef:UniRef50_UPI001124E470
RefSeq:WP_014115324.1, SO:0001217, UniParc:UPI00022BA497, UniRef:UniRef100_G4P1A2, UniRef:UniRef50_O34643, UniRef:UniRef90_A0AAP3CU86
GO:0005886, GO:0022857, GO:0055085, SO:0001217, UniRef:UniRef50_O34502, UniRef:UniRef90_O34502
BlastRules:WP_003242631, GO:0000917, GO:0005886, RefSeq:WP_014115326.1, SO:0001217, UniParc:UPI00022BA499, UniRef:UniRef100_G4P1A4, UniRef:UniRef50_O34375, UniRef:UniRef90_O34375
BlastRules:WP_015251121, SO:0001217, UniParc:UPI0001529547, UniRef:UniRef100_A0A063XC20, UniRef:UniRef50_A0A063XC20, UniRef:UniRef90_A0A063XC20
BlastRules:WP_003228041, EC:3.4.21.102, GO:0004175, GO:0004252, GO:0006508, GO:0006518, GO:0007165, GO:0008233, GO:0030288, GO:0030435, GO:0042277, GO:0042802, GO:0042803, SO:0001217, UniRef:UniF
BlastRules:WP_000625533, BlastRules:WP_000645021, BlastRules:WP_003416117, BlastRules:WP_009968247, GO:0005886, GO:0008356, GO:0016020, GO:0045121, GO:0045881, GO:0070297, GO:0090529, SO:000121
GO:0000910, GO:0005524, GO:0005886, GO:0008356, GO:0016887, GO:0022857, GO:0045881, GO:0055085, GO:0070297, GO:0090529, RefSeq:WP_003219741.1, SO:0001217, UniParc:UPI0001D284F8, UniRef:UniRef10
BlastRules:WP_000727961, BlastRules:WP_000727964, BlastRules:WP_003242725, GO:0005506, GO:0005886, GO:0009055, GO:0020037, RefSeq:WP_014115329.1, SO:0001217, UniParc:UPI00022BA49C, UniRef:UniRef
GO:0005886, RefSeq:WP_072182551.1, SO:0001217, UniParc:UPI0006A870C2, UniRef:UniRef100_UPI0006A870C2, UniRef:UniRef50_O34792, UniRef:UniRef90_O34792
GO:0004803, IS:ISBsu1, SO:0001217, UniRef:UniRef50_R2RXM0, UniRef:UniRef90_UPI0022831251
RFAM:RF01458, SO:0000655
SO:0001217, UniRef:UniRef50_A0A075R5J2, UniRef:UniRef90_E0TTR4
SO:0001217, UniRef:UniRef50_A0A345PEN8, UniRef:UniRef90_UPI002DB6E09E
PFAM:PF00472.26
PFAM:PF03462.23
COG:COG0653, COG:U, EC:7.4.2.8, GO:0005524, GO:0005829, GO:0005886, GO:0006605, GO:0006886, GO:0008564, GO:0017038, GO:0031522, GO:0043952, GO:0046872, GO:0065002, KEGG:K03070, SO:0001217, Ur
BlastRules:WP_003228031, GO:0005840, GO:0022627, GO:0043024, GO:0045182, GO:0045900, KEGG:K05808, RefSeq:WP_014115335.1, SO:0001217, UniParc:UPI00022BA4A2, UniRef:UniRef100_G4P1B5, UniRef:UniRe
RefSeq:WP_014115337.1, SO:0001217, UniParc:UPI00022BA4A4, UniRef:UniRef100_G4P1B7, UniRef:UniRef50_C0H3R9, UniRef:UniRef90_C0H3R9
GO:0005829, GO:0044781, RefSeq:WP_041521080.1, SO:0001217, UniParc:UPI0005A13744, UniRef:UniRef100_A0A9Q4E7F5, UniRef:UniRef50_P39740, UniRef:UniRef90_P39740
GO:0005829, GO:0044780, GO:0071973, KEGG:K02422, RefSeq:WP_072182549.1, SO:0001217, UniParc:UPI0006A88CF6, UniRef:UniRef100_UPI0006A88CF6, UniRef:UniRef50_P39739, UniRef:UniRef90_P39739
GO:0005576, GO:0007155, GO:0009421, GO:0009424, GO:0071973, KEGG:K02407, RefSeq:WP_072182548.1, SO:0001217, UniParc:UPI0006A8B58E, UniRef:UniRef100_UPI0006A8B58E, UniRef:UniRef50_P39738, UniRef
RefSeq:WP_072182547.1, SO:0001217, UniParc:UPI0006A820EB, UniRef:UniRef100_A0A9Q4E398, UniRef:UniRef50_P39737, UniRef:UniRef90_P39737
SO:0001217, UniRef:UniRef50_A0A1I3C6X8
GO:0003723, GO:0005829, GO:0006109, GO:0006402, GO:0045947, GO:0048027, GO:1902208, RefSeq:WP_003219727.1, SO:0001217, UniParc:UPI0000060B1A, UniRef:UniRef100_P33911, UniRef:UniRef50_P33911, Uni
GO:0005737, GO:0006417, GO:0044780, GO:0045727, GO:0071978, GO:1902021, GO:1902210, KEGG:K13626, RefSeq:WP_072182545.1, SO:0001217, UniParc:UPI0006A88CD1, UniRef:UniRef100_UPI0006A88CD1, UniF
RefSeq:WP_072182544.1, SO:0001217, UniParc:UPI0006A8DB5E, UniRef:UniRef100_UPI0006A8DB5E, UniRef:UniRef50_P96502, UniRef:UniRef90_P96502
GO:0001539, GO:0005198, GO:0005576, GO:0009424, GO:0071973, KEGG:K02397, RefSeq:WP_072182543.1, SO:0001217, UniParc:UPI0006A8914C, UniRef:UniRef100_UPI0006A8914C, UniRef:UniRef50_P96501, UniRef
GO:0005198, GO:0005576, GO:0009424, GO:0044780, RefSeq:WP_072182542.1, SO:0001217, UniParc:UPI0006A8BE91, UniRef:UniRef100_UPI0006A8BE91, UniRef:UniRef50_P39810, UniRef:UniRef90_P39810
BlastRules:WP_003227999, GO:0044780, SO:0001217, UniRef:UniRef50_P39808, UniRef:UniRef90_P39808
GO:0016989, GO:0044781, GO:0045892, RefSeq:WP_014115349.1, SO:0001217, UniParc:UPI00022BA610, UniRef:UniRef100_G4P1C9, UniRef:UniRef50_P39809, UniRef:UniRef90_P39809
GO:0030420, KEGG:K02242, RefSeq:WP_061186927.1, SO:0001217, UniParc:UPI000772CD56, UniRef:UniRef100_UPI000772CD56, UniRef:UniRef50_P39147, UniRef:UniRef90_P39147
GO:0030420, KEGG:K02241, RefSeq:WP_014115352.1, SO:0001217, UniParc:UPI00022BA613, UniRef:UniRef100_G4P1D2, UniRef:UniRef50_P39146, UniRef:UniRef90_P39146
KEGG:K02240, SO:0001217, UniRef:UniRef50_D5DWD0, UniRef:UniRef90_G4P1D3
GO:0008289, RefSeq:WP_014115354.1, SO:0001217, UniParc:UPI00022BA615, UniRef:UniRef100_G4P1D4, UniRef:UniRef50_P32436, UniRef:UniRef90_P32436
BlastRules:WP_003219701, COG:COG2197, COG:TK, GO:0000160, GO:0003677, GO:0005737, GO:0045893, RefSeq:WP_003219701.1, SO:0001217, UniParc:UPI000002C3A4, UniRef:UniRef100_P13800, UniRef:UniRef50_
BlastRules:WP_003227983, EC:2.7.13.3, EC:3.1.3.-, GO:0000155, GO:0004721, GO:0005524, GO:0005737, GO:0016020, GO:0046983, KEGG:K07777, RefSeq:WP_014115355.1, SO:0001217, UniParc:UPI00022BA616, Uni
GO:0005737, GO:0006446, SO:0001217, UniRef:UniRef50_P32437, UniRef:UniRef90_P32437
EC:2.7.8.33, GO:0000287, GO:0005886, GO:0009103, GO:0016757, GO:0016780, GO:0019350, GO:0030145, GO:0036380, GO:0044038, GO:0071555, KEGG:K02851, RefSeq:WP_061186932.1, SO:0001217, UniParc:UPI0
EC:2.4.-.-, GO:0016757, GO:0050845, GO:0071555, SO:0001217, UniRef:UniRef50_O32267, UniRef:UniRef90_O32267
EC:2.4.-.-, GO:0016757, GO:0050845, GO:0071555, KEGG:K16698, RefSeq:WP_072183701.1, SO:0001217, UniParc:UPI0006A7FC42, UniRef:UniRef100_UPI0006A7FC42, UniRef:UniRef50_O32268, UniRef:UniRef90_O322
GO:0005886, GO:0050845, GO:0071555, KEGG:K16706, RefSeq:WP_326198478.1, SO:0001217, UniParc:UPI002DB9C8CE, UniRef:UniRef100_UPI002DB9C8CE, UniRef:UniRef50_O32269, UniRef:UniRef90_O32269
BlastRules:WP_010886625, GO:0005886, GO:0050845, GO:0071555, KEGG:K16705, RefSeq:WP_014115370.1, SO:0001217, UniParc:UPI00022BA625, UniRef:UniRef100_G4P1F1, UniRef:UniRef50_O32270, UniRef:UniRef
BlastRules:WP_003242596, EC:1.1.1.22, GO:0000271, GO:0003979, GO:0005737, GO:0006065, GO:0051287, GO:0071555, KEGG:K00012, SO:0001217, UniRef:UniRef50_O32271, UniRef:UniRef90_O32271
EC:2.4.-.-, GO:0016757, GO:0050845, GO:0071555, RefSeq:WP_061186937.1, SO:0001217, UniParc:UPI000772510A, UniRef:UniRef100_UPI000772510A, UniRef:UniRef50_O32272, UniRef:UniRef90_O32272
GO:0005886, GO:0050845, GO:0071555, KEGG:K16694, RefSeq:WP_041521199.1, SO:0001217, UniParc:UPI0005A22A02, UniRef:UniRef100_A0A9Q4E391, UniRef:UniRef50_O32273, UniRef:UniRef90_O32273
RefSeq:WP_014115374.1, SO:0001217, UniParc:UPI00022BA629, UniRef:UniRef100_G4P1F5, UniRef:UniRef50_E7FXE4, UniRef:UniRef90_G4P1F5
SO:0001217, UniRef:UniRef50_E0U4V7, UniRef:UniRef90_UPI0009A25F60
SO:0001217, UniRef:UniRef50_UPI000281DF30
SO:0001217, UniRef:UniRef50_P54525
EC:3.5.1.28, KEGG:K01448, SO:0001217, UniRef:UniRef50_B7ZDM6, UniRef:UniRef90_UPI002DC022EC
EC:4.2.2.29, KEGG:K06381, RefSeq:WP_072183459.1, SO:0001217, UniParc:UPI0006A9040A, UniRef:UniRef100_UPI0006A9040A, UniRef:UniRef50_Q02113, UniRef:UniRef90_E0U4V9
SO:0001217, UniRef:UniRef50_A0AAP3CJ11, UniRef:UniRef90_UPI002DBD1D36
EC:2.7.8.-, KEGG:K01005, SO:0001217, UniRef:UniRef50_G4P1G0, UniRef:UniRef90_G4P1G0
EC:5.1.3.14, GO:0005737, GO:0008761, GO:0019350, GO:0071555, KEGG:K01791, SO:0001217, UniRef:UniRef50_P39131, UniRef:UniRef90_P39131
SO:0001217, UniRef:UniRef50_G4P1G2, UniRef:UniRef90_G4P1G2
PFAM:PF13692.12
EC:2.7.7.9, GO:0003983, GO:0005886, GO:0006011, GO:0009246, GO:0045121, KEGG:K00963, SO:0001217, UniRef:UniRef50_Q05852, UniRef:UniRef90_Q05852
EC:7.5.2.4, GO:0005524, GO:0005886, GO:0015438, GO:0016020, GO:0016887, GO:0140359, RefSeq:WP_072183589.1, SO:0001217, UniParc:UPI0006A93A97, UniRef:UniRef100_UPI0006A93A97, UniRef:UniRef50_P4295
BlastRules:WP_001273058, BlastRules:WP_003227928, KEGG:K09692, RefSeq:WP_079288833.1, SO:0001217, UniParc:UPI0008693C5B, UniRef:UniRef100_UPI0008693C5B, UniRef:UniRef50_P42953, UniRef:UniRef90_G
SO:0001217, UniRef:UniRef50_UPI00042A7AF6
EC:2.7.7.39, GO:0005737, GO:0019350, GO:0046872, GO:0047348, GO:0071555, RefSeq:WP_072181824.1, SO:0001217, UniParc:UPI0006A8424B, UniRef:UniRef100_UPI0006A8424B, UniRef:UniRef50_Q8RKI6, UniRef:Ur
EC:2.4.1.187, GO:0019350, GO:0047244, GO:0071555, KEGG:K05946, RefSeq:WP_014115391.1, SO:0001217, UniParc:UPI00022BA7AC, UniRef:UniRef100_G4P1H2, UniRef:UniRef50_P27620, UniRef:UniRef90_Q8RKI7
EC:2.7.8.44, GO:0005886, GO:0019350, GO:0047355, GO:0071555, SO:0001217, UniRef:UniRef50_P27621, UniRef:UniRef90_Q8RKI8

EC:2.7.7.40, GO:0008299, GO:0047349, GO:0050518, GO:0071555, GO:1902012, RefSeq:WP_072181820.1, SO:0001217, UniParc:UPI0006A847C2, UniRef:UniRef100_UPI0006A847C2, UniRef:UniRef50_Q2G2C4, UniRef:U
EC:1.1.1.405, GO:0008270, GO:0050256, GO:0071555, GO:1902012, KEGG:K05352, RefSeq:WP_003219623.1, SO:0001217, UniParc:UPI00000BC0AB, UniRef:UniRef100_Q8RKJ0, UniRef:UniRef50_Q2G1B9, UniRef:UniRe
EC:2.7.8.46, GO:0005886, GO:0019350, GO:0047355, GO:0071555, RefSeq:WP_072181819.1, SO:0001217, UniParc:UPI0006A8D991, UniRef:UniRef100_UPI0006A8D991, UniRef:UniRef50_Q8RKJ1, UniRef:UniRef90_Q8RI
SO:0001217, UniRef:UniRef50_A0A0M4G2U6
SO:0001217, UniRef:UniRef50_A0A5S9MGT9
PFAM:PF05036.18
EC:5.3.1.8, GO:0004476, GO:0005975, GO:0008270, SO:0001217, UniRef:UniRef50_O31646, UniRef:UniRef90_P39841
KEGG:K06291, SO:0001217, UniRef:UniRef50_P39569, UniRef:UniRef90_UPI0006A9141B
KEGG:K06292, SO:0001217, UniRef:UniRef50_P39570, UniRef:UniRef90_UPI001C1F3DFE
KEGG:K06293, RefSeq:WP_072181813.1, SO:0001217, UniParc:UPI0006A86117, UniRef:UniRef100_UPI0006A86117, UniRef:UniRef50_P39571, UniRef:UniRef90_E0U4Y5
GO:0005886, GO:0015293, GO:0016020, GO:0055085, RefSeq:WP_087987260.1, SO:0001217, UniParc:UPI000B454CA5, UniRef:UniRef100_UPI000B454CA5, UniRef:UniRef50_A0A0H2VG78, UniRef:UniRef90_C0SPB2
EC:2.7.8.-, GO:0005886, GO:0016740, GO:0071555, KEGG:K01005, SO:0001217, UniRef:UniRef50_Q7WY78, UniRef:UniRef90_Q7WY78
EC:3.1.3.104, GO:0000287, GO:0005829, GO:0009231, GO:0016791, GO:0043726, KEGG:K21064, SO:0001217, UniRef:UniRef50_P96741, UniRef:UniRef90_P96741
BlastRules:WP_003242751, EC:3.4.17.13, EC:3.4.19.-, GO:0005576, GO:0006508, GO:0008234, KEGG:K19303, RefSeq:WP_087987442.1, SO:0001217, UniParc:UPI000B443AAC, UniRef:UniRef100_UPI000B443AAC, UniR
BlastRules:WP_003227896, RefSeq:WP_014115405.1, SO:0001217, UniParc:UPI00022BA7BA, UniRef:UniRef100_G4P1I7, UniRef:UniRef50_P96739, UniRef:UniRef90_P96739
PFAM:PF09587.15
KEGG:K07282, SO:0001217, UniRef:UniRef50_P96738, UniRef:UniRef90_P96738
BlastRules:WP_000468007, BlastRules:WP_003227889, GO:0016020, GO:0045227, GO:0070501, KEGG:K22116, RefSeq:WP_003227889.1, SO:0001217, UniParc:UPI0000060B4B, UniRef:UniRef100_P96737, UniRef:UniRe
SO:0001217, UniRef:UniRef50_P96736
SO:0001217, UniRef:UniRef50_P36944
SO:0001217, UniRef:UniRef50_A0AB37HG73, UniRef:UniRef90_UPI00102E3E2E
EC:5.4.99.62, GO:0005829, GO:0016866, GO:0016872, GO:0019303, GO:0048029, GO:0062193, KEGG:K06726, RefSeq:WP_014115409.1, SO:0001217, UniParc:UPI00022BA932, UniRef:UniRef100_G4P1J3, UniRef:UniRe
BlastRules:WP_001217684, BlastRules:WP_003244379, EC:7.5.2.7, EC:7.5.2.8, EC:7.6.2.5, GO:0005524, GO:0005886, GO:0015439, GO:0015611, GO:0015886, GO:0016887, GO:0017004, GO:0022857, GO:0043190, RefS
BlastRules:WP_009968282, GO:0005886, GO:0022857, RefSeq:WP_014115411.1, SO:0001217, UniParc:UPI00022BA934, UniRef:UniRef100_G4P1J5, UniRef:UniRef50_P36948, UniRef:UniRef90_P36948
GO:0005886, GO:0030246, GO:0045121, SO:0001217, UniRef:UniRef50_P36949, UniRef:UniRef90_P36949
GO:0005576, SO:0001217, UniRef:UniRef50_P96729, UniRef:UniRef90_P96729
RefSeq:WP_003219574.1, SO:0001217, UniParc:UPI0001D2824E, UniRef:UniRef100_A0A9Q4DMU9, UniRef:UniRef50_P96728, UniRef:UniRef90_P96728
EC:1.6.99.-, GO:0003955, GO:0009055, GO:0010181, RefSeq:WP_014115415.1, SO:0001217, UniParc:UPI00022BA938, UniRef:UniRef100_G4P1J9, UniRef:UniRef50_P80871, UniRef:UniRef90_P80871
EC:4.1.1.5, GO:0045151, GO:0047605, KEGG:K01575, RefSeq:WP_003235587.1, SO:0001217, UniParc:UPI00022BA939, UniRef:UniRef100_A0A9W5PEM9, UniRef:UniRef50_Q04777, UniRef:UniRef90_Q04777
EC:2.2.1.6, GO:0000287, GO:0003984, GO:0005948, GO:0009097, GO:0009099, GO:0030976, GO:0034077, GO:0045151, GO:0050660, SO:0001217, UniRef:UniRef50_Q04789, UniRef:UniRef90_Q04789
KEGG:K21757, RefSeq:WP_014115417.1, SO:0001217, UniParc:UPI00022BA93B, UniRef:UniRef100_G4P1K2, UniRef:UniRef50_Q04778, UniRef:UniRef90_G4P1K2
GO:0030435, KEGG:K06330, RefSeq:WP_014115418.1, SO:0001217, UniParc:UPI00022BA93C, UniRef:UniRef100_G4P1K3, UniRef:UniRef50_Q45535, UniRef:UniRef90_Q45535
GO:0030435, GO:0043593, RefSeq:WP_072565383.1, SO:0001217, UniParc:UPI00090C7FB4, UniRef:UniRef100_UPI00090C7FB4, UniRef:UniRef50_P39801, UniRef:UniRef90_P39801
GO:0010181, GO:0016646, RefSeq:WP_014115420.1, SO:0001217, UniParc:UPI00022BA93E, UniRef:UniRef100_G4P1K5, UniRef:UniRef50_O05220, UniRef:UniRef90_O05220
RefSeq:WP_072181889.1, SO:0001217, UniParc:UPI0006A8FBBC, UniRef:UniRef100_UPI0006A8FBBC, UniRef:UniRef50_G4P1K6, UniRef:UniRef90_G4P1K6
EC:2.3.2.2, GO:0006508, GO:0006750, GO:0006751, GO:0036374, GO:0103068, SO:0001217, UniRef:UniRef50_O05218, UniRef:UniRef90_O05218
BlastRules:WP_003242900, GO:0005829, GO:0043200, GO:0043565, RefSeq:WP_014115423.1, SO:0001217, UniParc:UPI00022BA941, UniRef:UniRef100_G4P1K8, UniRef:UniRef50_O05217, UniRef:UniRef90_O05217
BlastRules:WP_003227843, GO:0005886, GO:0015109, KEGG:K07240, SO:0001217, UniRef:UniRef50_O05216, UniRef:UniRef90_O05216
BlastRules:WP_003117270, BlastRules:WP_003227840, GO:0005886, GO:0015109, KEGG:K07240, RefSeq:WP_014115425.1, SO:0001217, UniParc:UPI00022BA943, UniRef:UniRef100_G4P1L0, UniRef:UniRef50_O05215,
RefSeq:WP_061186449.1, SO:0001217, UniParc:UPI0006A8AFD2, UniRef:UniRef100_UPI0006A8AFD2, UniRef:UniRef50_P96727, UniRef:UniRef90_G4P1L1
EC:1.-.-.-, GO:0016491, RefSeq:WP_072181886.1, SO:0001217, UniParc:UPI0006A8FF92, UniRef:UniRef100_UPI0006A8FF92, UniRef:UniRef50_P96726, UniRef:UniRef90_P96726
GO:0000976, GO:0003700, GO:0005737, GO:0006355, RefSeq:WP_072181885.1, SO:0001217, UniParc:UPI0006A8300F, UniRef:UniRef100_UPI0006A8300F, UniRef:UniRef50_P96725, UniRef:UniRef90_P96725
KEGG:K09955, SO:0001217, UniRef:UniRef50_A0A1S8TI80, UniRef:UniRef90_E0U519
SO:0001217, UniRef:UniRef50_A0A0M2PNI9, UniRef:UniRef90_G4P1L5
EC:3.1.21.7, KEGG:K05982, SO:0001217, UniRef:UniRef50_P96724, UniRef:UniRef90_UPI000BAE2BD7
SO:0001217, UniRef:UniRef50_A0AAI8HKM1, UniRef:UniRef50_G4P1L7, UniRef:UniRef90_UPI000B450D04
SO:0001217, UniRef:UniRef50_A0A917D8G5, UniRef:UniRef90_A0A0T6BMR4
PFAM:PF09000.15
SO:0001217, UniRef:UniRef50_P96722, UniRef:UniRef90_A0A7G7UES6
RefSeq:WP_003221757.1, SO:0001217, UniParc:UPI0000060B68, UniRef:UniRef100_P96721, UniRef:UniRef50_P96721, UniRef:UniRef90_P96721
RefSeq:WP_014115436.1, SO:0001217, UniParc:UPI00022BA94E, UniRef:UniRef100_G4P1M3, UniRef:UniRef50_P96720, UniRef:UniRef90_E0TTZ3
BlastRules:WP_003243223, EC:1.1.1.22, GO:0000271, GO:0003979, GO:0005737, GO:0006065, GO:0051287, GO:0071555, RefSeq:WP_061186457.1, SO:0001217, UniParc:UPI0006A9093C, UniRef:UniRef100_UPI0006A9
RefSeq:WP_072181878.1, SO:0001217, UniParc:UPI0006A88529, UniRef:UniRef100_UPI0006A88529, UniRef:UniRef50_P96717, UniRef:UniRef90_G4P1M6
EC:2.7.10.2, GO:0004715, GO:0005524, GO:0035401, GO:0045226, GO:0140801, SO:0001217, UniRef:UniRef50_P96716, UniRef:UniRef90_P96716
BlastRules:WP_003227811, GO:0004713, GO:0005886, GO:0045227, RefSeq:WP_014115441.1, SO:0001217, UniParc:UPI00022BA953, UniRef:UniRef100_G4P1M8, UniRef:UniRef50_P96715, UniRef:UniRef90_P96715
RefSeq:WP_003221769.1, SO:0001217, UniParc:UPI0001D28CDE, UniRef:UniRef100_A0A9Q4DKW2, UniRef:UniRef50_C0H3S3, UniRef:UniRef90_C0H3S3
GO:0008270, SO:0001217, UniRef:UniRef50_P96714, UniRef:UniRef90_P96714
SO:0001217, UniRef:UniRef50_P94593
PFAM:PF00271.37
SO:0001217, UniRef:UniRef50_A0A5S9MI68, UniRef:UniRef90_A0A5S9MI68
EC:3.1.3.-, GO:0000287, GO:0005829, GO:0016791, SO:0001217, UniRef:UniRef50_P94592, UniRef:UniRef90_P94592
BlastRules:WP_003244075, GO:0003677, GO:0003700, SO:0001217, UniRef:UniRef50_P94591, UniRef:UniRef90_P94591
GO:0003697, GO:0005737, GO:0006260, GO:0006310, GO:0009295, GO:0030420, RefSeq:WP_014115447.1, SO:0001217, UniParc:UPI00022BAABB, UniRef:UniRef100_G4P1N5, UniRef:UniRef50_C0SPB6, UniRef:UniRef9
GO:0005886, SO:0001217, UniRef:UniRef50_P94589, UniRef:UniRef90_P94589
SO:0001217, UniRef:UniRef50_P94588, UniRef:UniRef90_P94588
SO:0001217, UniRef:UniRef50_D4G2F2, UniRef:UniRef90_D4G2F2
SO:0001217, UniRef:UniRef50_A0A9W5LLL1, UniRef:UniRef90_A0A9W5LLL1
RefSeq:WP_061186464.1, SO:0001217, UniParc:UPI0007725475, UniRef:UniRef100_A0A9Q4E1A1, UniRef:UniRef50_G4P1P0, UniRef:UniRef90_G4P1P0
SO:0001217, UniRef:UniRef50_Q62YG5, UniRef:UniRef90_G4P1P1
GO:0005886, GO:0006811, GO:0008381, GO:0016020, KEGG:K03282, RefSeq:WP_014115454.1, SO:0001217, UniParc:UPI00022BAAC2, UniRef:UniRef100_G4P1P2, UniRef:UniRef50_P94585, UniRef:UniRef90_P94585
EC:4.2.1.59, GO:0005737, GO:0006633, GO:0009245, GO:0016020, GO:0019171, KEGG:K02372, SO:0001217, UniRef:UniRef50_P94584, UniRef:UniRef90_P94584
BlastRules:WP_003227782, EC:3.1.-.-, GO:0005737, KEGG:K06362, RefSeq:WP_072181870.1, SO:0001217, UniParc:UPI0006A8D057, UniRef:UniRef100_UPI0006A8D057, UniRef:UniRef50_P94583, UniRef:UniRef90_P945
BlastRules:WP_003244381, GO:0009288, GO:0044781, GO:0071978, RefSeq:WP_061186466.1, SO:0001217, UniParc:UPI0006A89180, UniRef:UniRef100_UPI0006A89180, UniRef:UniRef50_P39753, UniRef:UniRef90_P397
BlastRules:WP_003244097, GO:0009288, GO:0044781, GO:0071978, SO:0001217, UniRef:UniRef50_P39752, UniRef:UniRef90_P39752
BlastRules:WP_003227776, COG:COG1077, COG:DZ, GO:0000902, GO:0005524, GO:0005737, GO:0008360, RefSeq:WP_014115458.1, SO:0001217, UniParc:UPI00022BAAC6, UniRef:UniRef100_G4P1P7, UniRef:UniRef5
GO:0003677, GO:0003700, GO:0030435, GO:0030436, GO:0140110, KEGG:K06283, RefSeq:WP_003221804.1, SO:0001217, UniParc:UPI0000060B7E, UniRef:UniRef100_P15281, UniRef:UniRef50_P15281, UniRef:UniRef9
GO:0003677, GO:0003700, RefSeq:WP_003235672.1, SO:0001217, UniParc:UPI00022BAAC7, UniRef:UniRef100_A0A9W5LLD3, UniRef:UniRef50_P94578, UniRef:UniRef90_P94578
GO:0005886, GO:0022857, RefSeq:WP_072565398.1, SO:0001217, UniParc:UPI00086CE511, UniRef:UniRef100_A0A9Q4E2X7, UniRef:UniRef50_P94577, UniRef:UniRef90_P94577
GO:0005576, GO:0016837, SO:0001217, UniRef:UniRef50_P94576, UniRef:UniRef90_P94576
BlastRules:WP_003243156, GO:0005886, GO:0015205, GO:0015293, GO:0015851, KEGG:K03457, RefSeq:WP_014115461.1, SO:0001217, UniParc:UPI00022BAACA, UniRef:UniRef100_G4P1Q2, UniRef:UniRef50_P94575,
GO:0005886, GO:0022857, GO:0055085, SO:0001217, UniRef:UniRef50_P94574, UniRef:UniRef90_P94574
EC:3.-.-.-, GO:0008908, SO:0001217, UniRef:UniRef50_P94573, UniRef:UniRef90_P94573
GO:0005886, RefSeq:WP_072181865.1, SO:0001217, UniParc:UPI0006A8285C, UniRef:UniRef100_UPI0006A8285C, UniRef:UniRef50_P94572, UniRef:UniRef90_P94572
BlastRules:WP_003227757, GO:0005886, GO:0008519, GO:0072488, KEGG:K03320, RefSeq:WP_014115465.1, SO:0001217, UniParc:UPI00022BAACE, UniRef:UniRef100_G4P1Q6, UniRef:UniRef50_Q07429, UniRef:UniRe
GO:0005524, GO:0005829, GO:0005886, GO:0006808, GO:0030234, RefSeq:WP_003221829.1, SO:0001217, UniParc:UPI0000060B88, UniRef:UniRef100_Q07428, UniRef:UniRef50_Q07428, UniRef:UniRef90_Q07428
BlastRules:WP_003243362, EC:3.6.1.27, GO:0005886, GO:0008360, GO:0009252, GO:0046677, GO:0050380, GO:0071555, RefSeq:WP_014115466.1, SO:0001217, UniParc:UPI00022BAACF, UniRef:UniRef100_G4P1Q8, U
SO:0001217, UniRef:UniRef50_P71045, UniRef:UniRef90_G4P1Q9
BlastRules:WP_003227751, GO:0004222, GO:0016020, GO:0030435, GO:0042601, KEGG:K06386, RefSeq:WP_061186472.1, SO:0001217, UniParc:UPI000772A904, UniRef:UniRef100_A0A9Q4HG46, UniRef:UniRef50_P71
EC:2.3.1.183, GO:0009635, GO:0016747, GO:0046677, GO:0102971, SO:0001217, UniParc:UPI00022BAAD2, UniRef:UniRef100_G4P1R1, UniRef:UniRef50_P71043, UniRef:UniRef90_P71043
SO:0001217, UniRef:UniRef50_P71042, UniRef:UniRef90_A0A9Q4HKH7
KEGG:K07257, SO:0001217, UniRef:UniRef50_D4G2H6, UniRef:UniRef90_D4G2H6
SO:0001217, UniRef:UniRef50_J9BNF2
GO:0005886, RefSeq:WP_014115472.1, SO:0001217, UniParc:UPI00022BAAD5, UniRef:UniRef100_G4P1R4, UniRef:UniRef50_P71041, UniRef:UniRef90_P71041
EC:2.7.8.-, GO:0005886, GO:0008808, GO:0016780, GO:0032049, GO:0043934, GO:0055085, KEGG:K06131, SO:0001217, UniRef:UniRef50_P71040, UniRef:UniRef90_P71040
KEGG:K21743, RefSeq:WP_088679669.1, SO:0001217, UniParc:UPI000B530455, UniRef:UniRef100_UPI000B530455, UniRef:UniRef50_P71039, UniRef:UniRef90_G4P1R6
GO:0016020, RefSeq:WP_088679668.1, SO:0001217, UniParc:UPI000B52DF7F, UniRef:UniRef100_UPI000B52DF7F, UniRef:UniRef50_P71038, UniRef:UniRef90_P71038
GO:0016646, KEGG:K07118, SO:0001217, UniRef:UniRef50_P71037, UniRef:UniRef90_P71037
GO:0003677, GO:0003700, GO:0005829, GO:0006355, RefSeq:WP_014115477.1, SO:0001217, UniParc:UPI00022BAC3D, UniRef:UniRef100_G4P1R9, UniRef:UniRef50_P71036, UniRef:UniRef90_P71036
EC:3.5.1.5, GO:0005737, GO:0009039, GO:0016151, GO:0019627, GO:0043419, RefSeq:WP_014115478.1, SO:0001217, UniParc:UPI00022BAC3E, UniRef:UniRef100_G4P1S0, UniRef:UniRef50_P77837, UniRef:UniRef90_F
RFAM:RF02514, SO:0000655
EC:3.5.1.5, GO:0009039, GO:0035550, GO:0043419, RefSeq:WP_014115479.1, SO:0001217, UniParc:UPI00022BAC3F, UniRef:UniRef100_G4P1S1, UniRef:UniRef50_P71035, UniRef:UniRef90_P71035
EC:3.5.1.5, GO:0005737, GO:0009039, GO:0016151, GO:0043419, RefSeq:WP_003227724.1, SO:0001217, UniParc:UPI0000060B96, UniRef:UniRef100_P75030, UniRef:UniRef50_P18316, UniRef:UniRef90_P75030
GO:0005524, GO:0009898, GO:0015840, GO:0033221, GO:0055052, RefSeq:WP_014115480.1, SO:0001217, UniParc:UPI00022BAC40, UniRef:UniRef100_G4P1S3, UniRef:UniRef50_G4P1S3, UniRef:UniRef90_G4P1S3
GO:0005524, GO:0009898, GO:0015840, GO:0033221, GO:0055052, RefSeq:WP_014115481.1, SO:0001217, UniParc:UPI00022BAC41, UniRef:UniRef100_G4P1S4, UniRef:UniRef50_C9KIW3, UniRef:UniRef90_G4P1S4
GO:0005886, GO:0015840, GO:0033221, GO:0055052, RefSeq:WP_014115482.1, SO:0001217, UniParc:UPI00022BAC42, UniRef:UniRef100_G4P1S5, UniRef:UniRef50_A0A0M0KDH9, UniRef:UniRef90_G4P1S5
GO:0005886, GO:0015840, GO:0033221, GO:0055052, RefSeq:WP_014115483.1, SO:0001217, UniParc:UPI00022BAC43, UniRef:UniRef100_G4P1S6, UniRef:UniRef50_A0A381YUM8, UniRef:UniRef90_G4P1S6
GO:0015840, GO:0030288, GO:0033219, GO:0033221, GO:0055052, KEGG:K11959, SO:0001217, UniRef:UniRef50_A0A0Q9Z1G3, UniRef:UniRef90_A0A8I2B9B3
RefSeq:WP_014115485.1, SO:0001217, UniParc:UPI00022BAC45, UniRef:UniRef100_G4P1S8, UniRef:UniRef50_P70964, UniRef:UniRef90_A0A9Q2LHK6
RefSeq:WP_014115486.1, SO:0001217, UniParc:UPI00022BAC46, UniRef:UniRef100_G4P1S9, UniRef:UniRef50_D5DIX4, UniRef:UniRef90_A0A6H0WNK5
BlastRules:WP_003227715, EC:3.1.3.-, GO:0004721, GO:0005737, GO:0030435, RefSeq:WP_014115487.1, SO:0001217, UniParc:UPI00022BAC47, UniRef:UniRef100_G4P1T0, UniRef:UniRef50_P70962, UniRef:UniRef90_F
EC:4.1.99.22, GO:0005525, GO:0006777, GO:0046872, GO:0051539, GO:0061798, GO:0061799, GO:1904047, RefSeq:WP_061186479.1, SO:0001217, UniParc:UPI0006A7F416, UniRef:UniRef100_A0A9Q4E4C0, UniRef:Un
GO:0005737, GO:0006777, GO:0009061, GO:0009326, GO:0015942, GO:0016783, GO:0097163, KEGG:K02379, RefSeq:WP_014115490.1, SO:0001217, UniParc:UPI00022BAC4A, UniRef:UniRef100_G4P1T3, UniRef:UniRe
GO:0005886, RefSeq:WP_014115491.1, SO:0001217, UniParc:UPI00022BAC4B, UniRef:UniRef100_G4P1T4, UniRef:UniRef50_P70966, UniRef:UniRef90_P70966
RefSeq:WP_072181855.1, SO:0001217, UniParc:UPI0006A93A02, UniRef:UniRef100_UPI0006A93A02, UniRef:UniRef50_P70961, UniRef:UniRef90_P70961
RefSeq:WP_014115494.1, SO:0001217, UniParc:UPI00022BAC4E, UniRef:UniRef100_G4P1T7, UniRef:UniRef50_P70960, UniRef:UniRef90_P70960
EC:4.2.2.29, GO:0030435, GO:0030436, KEGG:K06381, RefSeq:WP_014115495.1, SO:0001217, UniParc:UPI00022BAC4F, UniRef:UniRef100_G4P1T8, UniRef:UniRef50_P07372, UniRef:UniRef90_P07372
COG:COG0766, COG:M, EC:2.5.1.7, GO:0005737, GO:0008360, GO:0008760, GO:0009252, GO:0019277, GO:0051301, GO:0071555, KEGG:K00790, SO:0001217, UniRef:UniRef50_P70965, UniRef:UniRef90_P70965
SO:0001217, UniRef:UniRef50_O32277, UniRef:UniRef90_O32277
GO:0005886, RefSeq:WP_003221879.1, SO:0001217, UniParc:UPI0001D28DD4, UniRef:UniRef100_A0A9Q4E1F7, UniRef:UniRef50_O32278, UniRef:UniRef90_O32278
SO:0001217, UniRef:UniRef50_P70958, UniRef:UniRef90_A0A9Q4HPL6
EC:7.1.2.2, GO:0005524, GO:0005886, GO:0015986, GO:0045261, GO:0046933, RefSeq:WP_003221882.1, SO:0001217, UniParc:UPI0001D28DD6, UniRef:UniRef100_A0A9Q4DLR6, UniRef:UniRef50_P37812, UniRef:UniRe
COG:C, COG:COG0055, EC:7.1.2.2, GO:0005524, GO:0005886, GO:0015986, GO:0016887, GO:0045261, GO:0046933, GO:0046961, RefSeq:WP_003221884.1, SO:0001217, UniParc:UPI0001D28DD7, UniRef:UniRef100_A
EC:7.1.2.2, GO:0005524, GO:0005886, GO:0015986, GO:0042777, GO:0045121, GO:0045261, GO:0046933, KEGG:K02115, RefSeq:WP_003235766.1, SO:0001217, UniParc:UPI00022BAC55, UniRef:UniRef100_A0A2M8T1
COG:C, COG:COG0056, EC:7.1.2.2, GO:0005524, GO:0005886, GO:0015986, GO:0043531, GO:0045261, GO:0046933, GO:0046961, SO:0001217, UniRef:UniRef50_P37808, UniRef:UniRef90_P37808
SO:0001217, UniRef:UniRef50_A0A920DQK4
SO:0001217, UniRef:UniRef50_A0A2S6TLQ1, UniRef:UniRef90_UPI0021F0846F
EC:7.1.2.2, GO:0005886, GO:0015986, GO:0016020, GO:0045121, GO:0045261, GO:0046933, SO:0001217, UniRef:UniRef50_P37811, UniRef:UniRef90_P37811
EC:7.1.2.2, GO:0005886, GO:0015078, GO:0015986, GO:0045263, GO:0046933, RefSeq:WP_003235774.1, SO:0001217, UniParc:UPI00022BAC58, UniRef:UniRef100_A0A2M8T164, UniRef:UniRef50_P37814, UniRef:UniRe
COG:C, COG:COG0636, EC:7.1.2.2, GO:0005886, GO:0008289, GO:0015078, GO:0015986, GO:0045263, GO:0046933, RefSeq:WP_003151167.1, SO:0001217, UniParc:UPI0000060BAA, UniRef:UniRef100_P37815, UniRef
EC:7.1.2.2, GO:0005886, GO:0015078, GO:0015986, GO:0042777, GO:0045263, GO:0046933, KEGG:K02108, RefSeq:WP_003221896.1, SO:0001217, UniParc:UPI0001D28DDC, UniRef:UniRef100_A0A9Q4H828, UniRef:Ur
BlastRules:WP_003243159, GO:0005886, GO:0045263, GO:1902600, RefSeq:WP_003221899.1, SO:0001217, UniParc:UPI0001D28DDD, UniRef:UniRef100_A0A9Q4H9R3, UniRef:UniRef50_P37816, UniRef:UniRef90_P3781

EC:2.4.2.9, GO:0000287, GO:0004845, GO:0005525, GO:0006223, GO:0009116, GO:0044206, RefSeq:WP_003227670.1, SO:0001217, UniParc:UPI0000060BAD, UniRef:UniRef100_P39149, UniRef:UniRef50_P39149, UniRe
BlastRules:WP_000349814, BlastRules:WP_000919159, BlastRules:WP_003227669, COG:COG0112, COG:E, EC:2.1.2.1, GO:0004372, GO:0005829, GO:0019264, GO:0030170, GO:0035999, KEGG:K00600, RefSeq:WP_07
RefSeq:WP_061186484.1, SO:0001217, UniParc:UPI00077255F9, UniRef:UniRef100_UPI00077255F9, UniRef:UniRef50_P39157, UniRef:UniRef90_P39157
EC:5.3.1.-, EC:5.3.1.6, GO:0004751, GO:0009052, GO:0019316, KEGG:K01808, RefSeq:WP_003221910.1, SO:0001217, UniParc:UPI0000060BB0, UniRef:UniRef100_P39156, UniRef:UniRef50_P39156, UniRef:UniRef90_P3
EC:3.9.1.2, GO:0004725, GO:0098627, RefSeq:WP_014115508.1, SO:0001217, UniParc:UPI00022BAC5F, UniRef:UniRef100_G4P1W0, UniRef:UniRef50_P39155, UniRef:UniRef90_P39155
GO:0005384, GO:0005886, KEGG:K23242, SO:0001217, UniRef:UniRef50_P39154, UniRef:UniRef90_P39154
EC:2.7.7.87, GO:0000049, GO:0002949, GO:0003725, GO:0005524, GO:0005737, GO:0006400, GO:0006450, GO:0016779, GO:0061710, RefSeq:WP_061186486.1, SO:0001217, UniParc:UPI0006A8D7F4, UniRef:UniRef10
SO:0001217, UniParc:UPI00022BADE8, UniRef:UniRef100_G4P1W3, UniRef:UniRef50_P39152, UniRef:UniRef90_P39152
GO:0030435, GO:0030436, KEGG:K06387, RefSeq:WP_041521093.1, SO:0001217, UniParc:UPI0003112C64, UniRef:UniRef100_UPI0003112C64, UniRef:UniRef50_P39151, UniRef:UniRef90_P39151
GO:0005886, RefSeq:WP_014115512.1, SO:0001217, UniParc:UPI00022BADE9, UniRef:UniRef100_G4P1W4, UniRef:UniRef50_P39150, UniRef:UniRef90_P39150
GO:0005886, RefSeq:WP_014115513.1, SO:0001217, UniParc:UPI00022BADEA, UniRef:UniRef100_G4P1W5, UniRef:UniRef50_P45874, UniRef:UniRef90_P45874
EC:2.1.1.297, GO:0003676, GO:0006412, GO:0006415, GO:0008757, GO:0018364, GO:0032259, GO:0036009, GO:0102559, KEGG:K02493, RefSeq:WP_061186488.1, SO:0001217, UniParc:UPI000772403B, UniRef:UniRef
COG:COG0216, COG:J, GO:0003747, GO:0005737, GO:0006415, GO:0016149, KEGG:K02835, RefSeq:WP_003227645.1, SO:0001217, UniParc:UPI0000060BB9, UniRef:UniRef100_P45872, UniRef:UniRef50_B9DS45, Uni
GO:0046872, RefSeq:WP_014115515.1, SO:0001217, UniParc:UPI00022BADEC, UniRef:UniRef100_G4P1W8, UniRef:UniRef50_P52096, UniRef:UniRef90_P45871
GO:0003690, GO:0005737, GO:0006355, GO:0007059, GO:0008356, GO:0030261, GO:0030435, KEGG:K11686, SO:0001217, UniRef:UniRef50_P45870, UniRef:UniRef90_P45870
GO:0005886, GO:0055085, RefSeq:WP_072183002.1, SO:0001217, UniParc:UPI0006A88F9E, UniRef:UniRef100_A0A9Q4E0Q9, UniRef:UniRef50_P45869, UniRef:UniRef90_P45869
SO:0001217, UniRef:UniRef50_P45868
EC:2.7.1.21, GO:0004797, GO:0005524, GO:0005829, GO:0008270, GO:0046104, GO:0071897, KEGG:K00857, SO:0001217, UniRef:UniRef50_Q97F65, UniRef:UniRef90_Q03221
COG:COG0254, COG:J, GO:0003735, GO:0005840, GO:0006412, GO:0019843, GO:0046872, GO:1990904, RefSeq:WP_003151132.1, SO:0001217, UniParc:UPI0000060BBF, UniRef:UniRef100_Q03223, UniRef:UniRef50_C
RFAM:RF03156, SO:0000140
COG:COG1158, COG:K, EC:3.6.4.-, GO:0003723, GO:0004386, GO:0005524, GO:0006353, GO:0008186, GO:0016887, KEGG:K03628, RefSeq:WP_003221939.1, SO:0001217, UniParc:UPI000016E932, UniRef:UniRef100_C
COG:COG1494, COG:G, EC:3.1.3.11, GO:0005737, GO:0006071, GO:0006094, GO:0030388, GO:0042132, GO:0046872, KEGG:K02446, SO:0001217, UniRef:UniRef50_Q03224, UniRef:UniRef90_Q03224
EC:2.5.1.7, GO:0005737, GO:0008360, GO:0008760, GO:0009252, GO:0019277, GO:0051301, GO:0071555, KEGG:K00790, SO:0001217, UniRef:UniRef50_P19670, UniRef:UniRef90_P19670
EC:2.5.1.7, GO:0005737, GO:0008360, GO:0008760, GO:0009252, GO:0019277, GO:0051301, GO:0071555, KEGG:K00790, SO:0001217, UniRef:UniRef50_P19670, UniRef:UniRef90_P19670
EC:2.2.1.2, GO:0005975, GO:0016832, KEGG:K00616, SO:0001217, UniRef:UniRef50_P19669, UniRef:UniRef90_A0A081LE83
PFAM:PF01116.26
SO:0001217, UniRef:UniRef50_A0A2X3FV12
BlastRules:WP_000398594, BlastRules:WP_003227621, COG:COG5803, COG:DT, EC:2.7.-.-, GO:0000160, GO:0005737, GO:0016301, GO:0030435, GO:0046872, RefSeq:WP_003227621.1, SO:0001217, UniParc:UPI00000
GO:0097367, GO:1901135, SO:0001217, UniRef:UniRef50_P06629, UniRef:UniRef90_P06629
EC:6.3.4.2, GO:0003883, GO:0004359, GO:0005524, GO:0005829, GO:0006221, GO:0019856, GO:0042802, GO:0044210, GO:0046872, KEGG:K01937, RefSeq:WP_014115524.1, SO:0001217, UniParc:UPI00022BADF5, Ur
RFAM:RF02371, SO:0000140
EC:2.7.7.6, GO:0003899, GO:0006351, GO:0006355, KEGG:K03048, RefSeq:WP_053407039.1, SO:0001217, UniParc:UPI0006A95697, UniRef:UniRef100_UPI0006A95697, UniRef:UniRef50_A7Z9T5, UniRef:UniRef90_A0A9
BlastRules:WP_000545520, BlastRules:WP_003242719, EC:1.3.99.-, SO:0001217, UniRef:UniRef50_P45867, UniRef:UniRef90_A0A5D4N895
EC:1.-.-.-, GO:0005886, GO:0016491, GO:0046872, GO:0051539, KEGG:K08264, SO:0001217, UniRef:UniRef50_P45866, UniRef:UniRef90_P45866
SO:0001217, UniRef:UniRef50_UPI000ACD8341
EC:3.-.-.-, GO:0004518, GO:0004519, GO:0006281, GO:0006289, GO:0006290, GO:0009411, GO:0009650, KEGG:K13281, SO:0001217, UniRef:UniRef50_P45864, UniRef:UniRef90_P45864
RefSeq:WP_003221969.1, SO:0001217, UniParc:UPI0001D28EE8, UniRef:UniRef100_A0A9Q4E348, UniRef:UniRef50_P45863, UniRef:UniRef90_P45863
SO:0001217, UniRef:UniRef50_P45862, UniRef:UniRef90_G4P1Z1
SO:0001217, UniRef:UniRef50_UPI00325A662B
EC:2.7.8.-, GO:0005886, GO:0008808, GO:0016780, GO:0032049, KEGG:K06131, SO:0001217, UniRef:UniRef50_P45860, UniRef:UniRef90_P45860
SO:0001217, UniRef:UniRef50_I2CBB9, UniRef:UniRef90_UPI0010093EC8
EC:1.7.5.1, EC:1.7.99.4, GO:0005886, GO:0008940, GO:0009055, GO:0009325, GO:0019645, GO:0020037, GO:0042128, GO:0046872, GO:0160182, RefSeq:WP_072182963.1, SO:0001217, UniParc:UPI0006A8D277, UniRe
GO:0051082, GO:0051131, KEGG:K00373, SO:0001217, UniRef:UniRef50_P42178, UniRef:UniRef90_A0A2M8T130
EC:1.7.5.1, EC:1.7.99.4, GO:0005886, GO:0008940, GO:0009055, GO:0009061, GO:0009325, GO:0016020, GO:0042126, GO:0042128, GO:0046872, GO:0051538, GO:0051539, GO:0160182, KEGG:K00371, SO:0001217, U
SO:0001217, UniRef:UniRef50_A0A3E0IMI0, UniRef:UniRef90_A0A0D1KIW8
EC:1.7.5.1, EC:1.7.99.-, EC:1.7.99.4, GO:0008940, GO:0009325, GO:0042126, KEGG:K00370, SO:0001217, UniRef:UniRef50_P9WJQ3, UniRef:UniRef90_A0A6A8LC65
RFAM:RF01690, SO:0000655
BlastRules:WP_003243032, GO:0003677, RefSeq:WP_014115540.1, SO:0001217, UniParc:UPI00022BAE05, UniRef:UniRef100_G4P200, UniRef:UniRef50_P46910, UniRef:UniRef90_P46910
GO:0005886, RefSeq:WP_072182969.1, SO:0001217, UniParc:UPI0006A7FAD8, UniRef:UniRef100_UPI0006A7FAD8, UniRef:UniRef50_P46909, UniRef:UniRef90_P46909
BlastRules:WP_000611911, BlastRules:WP_003243241, BlastRules:WP_005662331, GO:0003677, GO:0003700, GO:0005829, GO:0046872, GO:0051539, RefSeq:WP_072182971.1, SO:0001217, UniParc:UPI0006A85AE3, U
BlastRules:WP_000019827, BlastRules:WP_000841851, BlastRules:WP_003898992, BlastRules:WP_010886631, GO:0005886, GO:0015112, GO:0042128, KEGG:K02575, SO:0001217, UniRef:UniRef50_P46907, UniRef:Uni
SO:0001217, UniRef:UniRef50_W8YRU1
SO:0001217, UniRef:UniRef50_A0A2T4S724
RefSeq:WP_014115545.1, SO:0001217, UniParc:UPI00022BAF87, UniRef:UniRef100_G4NXI0, UniRef:UniRef50_O07624, UniRef:UniRef90_O07624
GO:0005576, GO:0031640, GO:0042742, RefSeq:WP_003222002.1, SO:0001217, UniParc:UPI0000060BDA, UniRef:UniRef100_O07623, UniRef:UniRef50_O07623, UniRef:UniRef90_O07623
EC:1.21.98.-, GO:0005737, GO:0016491, GO:0030152, GO:0046872, GO:0051539, KEGG:K27612, SO:0001217, UniRef:UniRef50_P71011, UniRef:UniRef90_P71011
BlastRules:WP_003222006, GO:0005886, GO:0030152, GO:0030153, KEGG:K27613, RefSeq:WP_061186507.1, SO:0001217, UniParc:UPI00077230ED, UniRef:UniRef100_A0A9Q4HG77, UniRef:UniRef50_P71010, UniRef:U
GO:0005524, GO:0015031, GO:0016887, GO:0030153, GO:0043213, RefSeq:WP_061186508.1, SO:0001217, UniParc:UPI000772BB11, UniRef:UniRef100_UPI000772BB11, UniRef:UniRef50_P71009, UniRef:UniRef90_P71
BlastRules:WP_003222009, BlastRules:WP_003243158, GO:0005886, GO:0030152, GO:0030153, KEGG:K27615, SO:0001217, UniRef:UniRef50_P71008, UniRef:UniRef90_P71008
GO:0030152, GO:0046872, KEGG:K27616, SO:0001217, UniRef:UniRef50_P71007, UniRef:UniRef90_Q8RKH3
EC:3.4.24.-, GO:0006508, GO:0008237, GO:0030152, GO:0046872, KEGG:K27617, RefSeq:WP_072182980.1, SO:0001217, UniParc:UPI0006A85EC5, UniRef:UniRef100_UPI0006A85EC5, UniRef:UniRef50_P71006, UniRef:
GO:0005886, GO:0030152, KEGG:K27618, RefSeq:WP_242733684.1, SO:0001217, UniParc:UPI001F61B7CC, UniRef:UniRef100_UPI001F61B7CC, UniRef:UniRef50_P71005, UniRef:UniRef90_Q8RKH1
SO:0001217, UniRef:UniRef50_P71003, UniRef:UniRef90_G4NXI9
SO:0001217, UniRef:UniRef50_UPI001CD058B7
RefSeq:WP_041521094.1, SO:0001217, UniParc:UPI0005A2BE25, UniRef:UniRef100_UPI0005A2BE25, UniRef:UniRef50_A0A1J9T953, UniRef:UniRef90_A0A2M8T0Y6
RefSeq:WP_014115557.1, SO:0001217, UniParc:UPI00022BAF93, UniRef:UniRef100_G4NXJ3, UniRef:UniRef50_A0A6H0WP92, UniRef:UniRef90_A0A6H0WP92
RefSeq:WP_014115558.1, SO:0001217, UniParc:UPI00022BAF94, UniRef:UniRef100_G4NXJ4, UniRef:UniRef50_A0A2M8T115, UniRef:UniRef90_A0A6H0WML6
SO:0001217, UniParc:UPI00022BAF95, UniRef:UniRef100_G4NXJ5, UniRef:UniRef50_A0A6H0WMM8, UniRef:UniRef90_A0A6H0WMM8
RefSeq:WP_014115560.1, SO:0001217, UniParc:UPI00022BAF96, UniRef:UniRef100_G4NXJ6, UniRef:UniRef50_A0A174YTF6, UniRef:UniRef90_G4NXJ6
RefSeq:WP_014115561.1, SO:0001217, UniParc:UPI00022BAF97, UniRef:UniRef100_G4NXJ7, UniRef:UniRef50_A0A6H0WR42, UniRef:UniRef90_A0A6H0WR42
COG:COG0010, COG:E, EC:3.5.3.11, GO:0008295, GO:0008783, GO:0033389, GO:0046872, RefSeq:WP_014115562.1, SO:0001217, UniParc:UPI00022BAF98, UniRef:UniRef100_G4NXJ8, UniRef:UniRef50_P70999, UniRef
BlastRules:WP_000424696, BlastRules:WP_003227543, COG:COG0421, COG:E, EC:2.5.1.16, GO:0003824, GO:0004766, GO:0005829, GO:0008295, RefSeq:WP_003222027.1, SO:0001217, UniParc:UPI0001D28FE3, UniR
EC:2.4.99.28, EC:3.4.16.4, GO:0005886, GO:0006508, GO:0008360, GO:0008658, GO:0008955, GO:0009002, GO:0009252, GO:0030288, GO:0071555, KEGG:K05366, RefSeq:WP_072182347.1, SO:0001217, UniParc:UPI0
SO:0001217, UniRef:UniRef50_P70996, UniRef:UniRef90_P70996
EC:3.4.24.-, GO:0005886, GO:0006508, GO:0008237, GO:0046872, SO:0001217, UniRef:UniRef50_P70995, UniRef:UniRef90_P70995
SO:0001217, UniRef:UniRef50_A0A5S9MLV6, UniRef:UniRef90_UPI0020BDEA37
EC:5.3.2.6, GO:0008152, GO:0016853, GO:0016862, RefSeq:WP_003222038.1, SO:0001217, UniParc:UPI0000060BEC, UniRef:UniRef100_P70994, UniRef:UniRef50_P70994, UniRef:UniRef90_P70994
GO:0003677, GO:0003700, GO:0006355, GO:0006950, RefSeq:WP_014115567.1, SO:0001217, UniParc:UPI00022BAF9D, UniRef:UniRef100_A0A9Q4E1L0, UniRef:UniRef50_P70993, UniRef:UniRef90_P70993
EC:6.1.1.3, GO:0000049, GO:0004829, GO:0005524, GO:0005737, GO:0006435, GO:0016740, GO:0046872, KEGG:K01868, RefSeq:WP_090556970.1, SO:0001217, UniParc:UPI00086E2F1B, UniRef:UniRef100_UPI00086E
RFAM:RF00230, SO:0000140
RFAM:RF00230, SO:0000140
RFAM:RF00230, SO:0000140
RefSeq:WP_133063232.1, SO:0001217, UniParc:UPI0006A8DC0A, UniRef:UniRef100_UPI0006A8DC0A, UniRef:UniRef50_A0A2K4Z9N1, UniRef:UniRef90_G4NXK8
KEGG:K09388, RefSeq:WP_003222046.1, SO:0001217, UniParc:UPI0001D28FEB, UniRef:UniRef100_A0A2M8T0Y9, UniRef:UniRef50_P71046, UniRef:UniRef90_P71046
GO:0006203, GO:0008832, RefSeq:WP_061186520.1, SO:0001217, UniParc:UPI0007725251, UniRef:UniRef100_UPI0007725251, UniRef:UniRef50_P39651, UniRef:UniRef90_P39651
RefSeq:WP_003222050.1, SO:0001217, UniParc:UPI0000060BF3, UniRef:UniRef100_O32280, UniRef:UniRef50_O32280, UniRef:UniRef90_O32280
BlastRules:WP_003243464, GO:0003677, GO:0030435, GO:0042763, KEGG:K06314, SO:0001217, UniRef:UniRef50_P39650, UniRef:UniRef90_P39650
GO:0016020, KEGG:K03298, SO:0001217, UniRef:UniRef50_A0A662Z2F8, UniRef:UniRef90_UPI002280EB4F
BlastRules:WP_003227511, EC:2.3.1.204, GO:0009107, GO:0009249, GO:0033819, KEGG:K16869, SO:0001217, UniRef:UniRef50_P39648, UniRef:UniRef90_P39648
BlastRules:WP_003243173, GO:0000976, GO:0003700, GO:0006355, RefSeq:WP_072182879.1, SO:0001217, UniParc:UPI0006A82216, UniRef:UniRef100_UPI0006A82216, UniRef:UniRef50_P39647, UniRef:UniRef90_P396
COG:C, COG:COG0280, EC:2.3.1.8, GO:0005737, GO:0006085, GO:0006086, GO:0008959, RefSeq:WP_014115576.1, SO:0001217, UniParc:UPI00022BAFA6, UniRef:UniRef100_G4NXL7, UniRef:UniRef50_P39646, UniRef:
EC:1.3.98.5, GO:0004601, GO:0006783, GO:0006785, GO:0020037, GO:0046872, RefSeq:WP_014115577.1, SO:0001217, UniParc:UPI00022BAFA7, UniRef:UniRef100_G4NXL8, UniRef:UniRef50_Q8Y5F1, UniRef:UniRef90_
BlastRules:WP_003244095, EC:1.3.1.-, GO:0005737, GO:0016628, GO:0017000, GO:0050661, KEGG:K19550, RefSeq:WP_090556967.1, SO:0001217, UniParc:UPI00086861F9, UniRef:UniRef100_UPI00086861F9, UniRef:U
BlastRules:WP_003242568, EC:2.6.1.-, GO:0005737, GO:0008483, GO:0017000, GO:0030170, RefSeq:WP_072182881.1, SO:0001217, UniParc:UPI0006A9102D, UniRef:UniRef100_UPI0006A9102D, UniRef:UniRef50_P396
BlastRules:WP_003244502, GO:0005886, GO:0022857, KEGG:K19552, RefSeq:WP_061186525.1, SO:0001217, UniParc:UPI0006A85B99, UniRef:UniRef100_UPI0006A85B99, UniRef:UniRef50_P39642, UniRef:UniRef90_P3
BlastRules:WP_003242921, EC:6.3.2.49, GO:0005524, GO:0017000, GO:0034026, GO:0046872, KEGG:K13037, RefSeq:WP_072182882.1, SO:0001217, UniParc:UPI0006A8640C, UniRef:UniRef100_UPI0006A8640C, UniRe
EC:1.1.1.385, GO:0016491, GO:0017000, RefSeq:WP_072182883.1, SO:0001217, UniParc:UPI0006A86D37, UniRef:UniRef100_UPI0006A86D37, UniRef:UniRef50_P39640, UniRef:UniRef90_P39640
BlastRules:WP_003244300, EC:5.3.3.19, GO:0005506, GO:0005737, GO:0016863, GO:0017000, GO:0050897, KEGG:K19547, RefSeq:WP_072182884.1, SO:0001217, UniParc:UPI0006A92B89, UniRef:UniRef100_UPI0006A
BlastRules:WP_009968341, EC:4.1.1.100, GO:0005737, GO:0016831, GO:0017000, KEGG:K19546, RefSeq:WP_072182885.1, SO:0001217, UniParc:UPI0006A8FC01, UniRef:UniRef100_UPI0006A8FC01, UniRef:UniRef50_
GO:0005886, GO:0022857, GO:0055085, RefSeq:WP_061186529.1, SO:0001217, UniParc:UPI0006A91FCD, UniRef:UniRef100_UPI0006A91FCD, UniRef:UniRef50_P39637, UniRef:UniRef90_P39637
GO:0003333, GO:0005886, GO:0006525, GO:0015171, GO:0016020, KEGG:K02205, SO:0001217, UniRef:UniRef50_P39137, UniRef:UniRef90_P39636
SO:0001217, UniRef:UniRef50_P39635
EC:1.2.1.88, GO:0003842, GO:0006537, GO:0006562, GO:0009898, GO:0010133, KEGG:K00294, RefSeq:WP_088679627.1, SO:0001217, UniParc:UPI000B533673, UniRef:UniRef100_UPI000B533673, UniRef:UniRef50_P3
BlastRules:WP_003227482, EC:1.4.1.2, GO:0004352, GO:0006520, GO:0006538, RefSeq:WP_072183229.1, SO:0001217, UniParc:UPI0006A81C2D, UniRef:UniRef100_UPI0006A81C2D, UniRef:UniRef50_P39633, UniRef:Ur
BlastRules:WP_003242493, GO:0005576, RefSeq:WP_061186532.1, SO:0001217, UniParc:UPI0006A86E52, UniRef:UniRef100_UPI0006A86E52, UniRef:UniRef50_P39632, UniRef:UniRef90_P39632
SO:0001217, UniRef:UniRef50_Q7WY56, UniRef:UniRef90_A0A5D4N722
EC:1.1.1.133, GO:0008831, GO:0009243, GO:0019305, KEGG:K00067, RefSeq:WP_041521097.1, SO:0001217, UniParc:UPI00031541C3, UniRef:UniRef100_A0A9Q4E0W3, UniRef:UniRef50_P39631, UniRef:UniRef90_A0A6
EC:4.2.1.46, GO:0008460, GO:0009225, GO:0019305, SO:0001217, UniRef:UniRef50_P39630, UniRef:UniRef90_A0A6H0WNW8
SO:0001217, UniRef:UniRef50_A0A5S9MLA6
PFAM:PF12804.13
KEGG:K07257, SO:0001217, UniRef:UniRef50_P39626, UniRef:UniRef90_E0TW32
GO:0016051, GO:0047444, GO:0070085, SO:0001217, UniRef:UniRef50_P39625, UniRef:UniRef90_P39625
EC:2.3.1.-, GO:0016747, RefSeq:WP_061186535.1, SO:0001217, UniParc:UPI0007728069, UniRef:UniRef100_UPI0007728069, UniRef:UniRef50_P39624, UniRef:UniRef90_P39624
GO:0000271, GO:0008483, GO:0030170, SO:0001217, UniRef:UniRef50_P39623, UniRef:UniRef90_P39623
GO:0016020, GO:0047355, SO:0001217, UniRef:UniRef50_P39622, UniRef:UniRef90_P39622
BlastRules:WP_003244383, GO:0016757, RefSeq:WP_014115596.1, SO:0001217, UniParc:UPI00022BB148, UniRef:UniRef100_G4NXN9, UniRef:UniRef50_P39621, UniRef:UniRef90_P39621
GO:0030435, GO:0030436, GO:0031160, KEGG:K06305, RefSeq:WP_014115597.1, SO:0001217, UniParc:UPI00022BB149, UniRef:UniRef100_G4NXP0, UniRef:UniRef50_P39620, UniRef:UniRef90_P39620
GO:0005886, RefSeq:WP_003222123.1, SO:0001217, UniParc:UPI0001D28297, UniRef:UniRef100_G4NXP1, UniRef:UniRef50_P39619, UniRef:UniRef90_P39619
GO:0005886, GO:0042906, GO:0042907, SO:0001217, UniRef:UniRef50_P39618, UniRef:UniRef90_P39618
RefSeq:WP_072183239.1, SO:0001217, UniParc:UPI0006A8FA63, UniRef:UniRef100_UPI0006A8FA63, UniRef:UniRef50_P39617, UniRef:UniRef90_P39617
EC:1.2.1.3, GO:0004029, GO:0005737, GO:0006081, SO:0001217, UniRef:UniRef50_P39616, UniRef:UniRef90_P39616
EC:3.2.2.27, GO:0004844, GO:0005737, GO:0006284, GO:0097510, KEGG:K03648, SO:0001217, UniRef:UniRef50_P39615, UniRef:UniRef90_P39615
EC:2.4.-.-, GO:0016757, RefSeq:WP_014115603.1, SO:0001217, UniParc:UPI00022BB14F, UniRef:UniRef100_G4NXP7, UniRef:UniRef50_P39614, UniRef:UniRef90_P39614
GO:0005886, RefSeq:WP_072183242.1, SO:0001217, UniParc:UPI0006A8B035, UniRef:UniRef100_UPI0006A8B035, UniRef:UniRef50_P39613, UniRef:UniRef90_P39613
SO:0001217, UniRef:UniRef50_UPI002B1EDABF, UniRef:UniRef90_UPI002281F258
GO:0003677, SO:0001217, UniRef:UniRef50_C0H3S6, UniRef:UniRef90_C0H3S6
EC:2.7.1.35, EC:2.7.1.49, GO:0005524, GO:0005829, GO:0008478, GO:0008902, GO:0008972, GO:0009228, GO:0046872, RefSeq:WP_072183245.1, SO:0001217, UniParc:UPI0006A892BC, UniRef:UniRef100_UPI0006A89
RefSeq:WP_014115608.1, SO:0001217, UniParc:UPI00022BB154, UniRef:UniRef100_G4NXQ2, UniRef:UniRef50_P39609, UniRef:UniRef90_P39609
EC:3.2.1.26, GO:0004564, GO:0005737, GO:0005985, SO:0001217, UniRef:UniRef50_P07819, UniRef:UniRef90_P07819
BlastRules:WP_003227426, GO:0005886, GO:0009401, GO:0015771, GO:0016301, GO:0022878, GO:0090589, RefSeq:WP_014115610.1, SO:0001217, UniParc:UPI00022BB156, UniRef:UniRef100_G4NXQ4, UniRef:UniRef

GO:0005886, GO:0015499, GO:0015724, SO:0001217, UniRef:UniRef50_P39608, UniRef:UniRef90_P39608
BlastRules:WP_003243088, GO:0003723, GO:0045893, KEGG:K26932, RefSeq:WP_061186544.1, SO:0001217, UniParc:UPI0006A8169F, UniRef:UniRef100_A0A9Q4E377, UniRef:UniRef50_P26212, UniRef:UniRef90_P2621
RefSeq:WP_003222155.1, SO:0001217, UniParc:UPI00015D990F, UniRef:UniRef100_P39607, UniRef:UniRef50_P39607, UniRef:UniRef90_P39607
BlastRules:WP_003227419, EC:3.4.21.-, GO:0004252, GO:0005576, GO:0006508, SO:0001217, UniRef:UniRef50_P29141, UniRef:UniRef90_P29141
SO:0001217, UniRef:UniRef50_A0A5S9MI10
SO:0001217, UniRef:UniRef50_A0A7Y3M8W5
EC:1.5.1.38, GO:0008752, GO:0016491, GO:0052873, RefSeq:WP_014115615.1, SO:0001217, UniParc:UPI00022BB2C2, UniRef:UniRef100_G4NXZ0, UniRef:UniRef50_P39605, UniRef:UniRef90_P39605
BlastRules:WP_003227413, BlastRules:WP_003400364, EC:2.4.99.28, GO:0005886, GO:0008360, GO:0008955, GO:0009252, GO:0015648, GO:0032153, GO:0051301, GO:0071555, RefSeq:WP_061186547.1, SO:0001217,
SO:0001217, UniRef:UniRef50_D4G2Z7, UniRef:UniRef90_D4G2Z7
BlastRules:WP_003186174, BlastRules:WP_003227411, BlastRules:WP_010898226, BlastRules:WP_011246976, GO:0005886, GO:0009847, GO:0043594, SO:0001217, UniParc:UPI00019765F6, UniRef:UniRef100_P39603,
EC:1.10.3.-, GO:0005886, GO:0009319, GO:0009486, GO:0015078, GO:0015990, GO:0016020, GO:0016682, GO:0019646, GO:0042773, KEGG:K02829, SO:0001217, UniRef:UniRef50_E0TW64, UniRef:UniRef90_E0TW64
EC:1.10.3.-, GO:0004129, GO:0005886, GO:0009055, GO:0009319, GO:0009486, GO:0015078, GO:0015990, GO:0016020, GO:0016682, GO:0019646, GO:0042773, RefSeq:WP_003236031.1, SO:0001217, UniParc:UPI000
EC:1.10.3.-, GO:0004129, GO:0005507, GO:0005886, GO:0009060, GO:0015990, GO:0016020, GO:0016682, GO:0020037, GO:0022904, GO:0042773, KEGG:K02827, RefSeq:WP_061186549.1, SO:0001217, UniParc:UPI0
EC:1.10.3.-, GO:0004129, GO:0005507, GO:0005886, GO:0009055, GO:0009486, GO:0015078, GO:0015990, GO:0016020, GO:0016682, GO:0042773, GO:0045121, KEGG:K02826, SO:0001217, UniRef:UniRef50_P34957, U
GO:0005886, SO:0001217, UniParc:UPI00022BB2C9, UniRef:UniRef100_A0A9W5PEN4, UniRef:UniRef50_P96594, UniRef:UniRef90_O32282
EC:2.7.7.12, GO:0005737, GO:0006012, GO:0008108, GO:0017103, GO:0019388, SO:0001217, UniRef:UniRef50_P39575, UniRef:UniRef90_P39575
EC:2.7.1.6, GO:0000287, GO:0004335, GO:0005524, GO:0005829, GO:0006012, GO:0046835, KEGG:K00849, SO:0001217, UniRef:UniRef50_P96993, UniRef:UniRef90_P39574
GO:0000271, GO:0005886, SO:0001217, UniRef:UniRef50_P39602, UniRef:UniRef90_P39602
GO:0003677, RefSeq:WP_072565468.1, SO:0001217, UniParc:UPI00086BCFDC, UniRef:UniRef100_UPI00086BCFDC, UniRef:UniRef50_P39601, UniRef:UniRef90_P39601
GO:0006355, GO:0046983, RefSeq:WP_014115625.1, SO:0001217, UniParc:UPI00022BB2CE, UniRef:UniRef100_G4NY04, UniRef:UniRef50_P0C8M5, UniRef:UniRef90_P0C8M5
GO:0005886, RefSeq:WP_038429826.1, SO:0001217, UniParc:UPI0005302D82, UniRef:UniRef100_UPI0005302D82, UniRef:UniRef50_P39600, UniRef:UniRef90_P39600
SO:0001217, UniRef:UniRef50_A0A9W4KTY5
SO:0001217, UniRef:UniRef50_B9E981
GO:0016491, SO:0001217, UniRef:UniRef50_P39598, UniRef:UniRef90_P39598
SO:0001217, UniRef:UniRef50_P39597
BlastRules:WP_003243208, GO:0005886, GO:0006826, GO:0045121, GO:0046872, KEGG:K07224, RefSeq:WP_088680046.1, SO:0001217, UniParc:UPI000B5349CD, UniRef:UniRef100_UPI000B5349CD, UniRef:UniRef50_
BlastRules:WP_003243918, GO:0005886, GO:0015093, GO:0033573, GO:0034755, KEGG:K07243, SO:0001217, UniRef:UniRef50_P39595, UniRef:UniRef90_P39595
EC:2.5.1.3, GO:0000287, GO:0004789, GO:0005737, GO:0009228, GO:0009229, SO:0001217, UniRef:UniRef50_P39594, UniRef:UniRef90_P39594
BlastRules:WP_001092689, BlastRules:WP_003244274, EC:2.7.1.50, GO:0000287, GO:0004417, GO:0005524, GO:0009228, GO:0009229, KEGG:K00878, RefSeq:WP_072182118.1, SO:0001217, UniParc:UPI0006A92233, U
GO:0003677, GO:0003700, GO:0005829, GO:0006355, RefSeq:WP_014115634.1, SO:0001217, UniParc:UPI00022BB2D7, UniRef:UniRef100_G4NY14, UniRef:UniRef50_P39592, UniRef:UniRef90_P39592
GO:0005886, GO:0012501, GO:0019835, GO:0031640, RefSeq:WP_003222205.1, SO:0001217, UniParc:UPI0001D28378, UniRef:UniRef100_A0A9Q4DLY0, UniRef:UniRef50_P39591, UniRef:UniRef90_P39591
GO:0005886, RefSeq:WP_014478359.1, SO:0001217, UniParc:UPI00022BBB93, UniRef:UniRef100_UPI00022BBB93, UniRef:UniRef50_P39590, UniRef:UniRef90_P39590
SO:0001217, UniRef:UniRef50_UPI002E15E09B
SO:0001217, UniRef:UniRef50_A0A370GQJ6
RefSeq:WP_003222222.1, SO:0001217, UniParc:UPI0001D28381, UniRef:UniRef100_A0A9Q4HHY2, UniRef:UniRef50_P39588, UniRef:UniRef90_P39588
EC:2.1.1.-, GO:0003723, GO:0005737, GO:0006364, GO:0008168, GO:0032259, SO:0001217, UniRef:UniRef50_P39587, UniRef:UniRef90_P39587
BlastRules:WP_003243380, RefSeq:WP_072182115.1, SO:0001217, UniParc:UPI0006A80BBC, UniRef:UniRef100_UPI0006A80BBC, UniRef:UniRef50_P39586, UniRef:UniRef90_A0AAP3CJP6
RefSeq:WP_326200192.1, SO:0001217, UniParc:UPI002DB6EC5D, UniRef:UniRef100_UPI002DB6EC5D, UniRef:UniRef50_P39585, UniRef:UniRef90_E0TWQ0
EC:2.7.1.205, GO:0008982, GO:0009401, GO:0016020, GO:0019191, KEGG:K02761, RefSeq:WP_072182113.1, SO:0001217, UniParc:UPI0006A8C3AF, UniRef:UniRef100_UPI0006A8C3AF, UniRef:UniRef50_P39584, UniRef
SO:0001217, UniRef:UniRef50_P16396
BlastRules:WP_003243800, GO:0005886, GO:0008982, GO:0009401, GO:0015771, GO:0016301, GO:0090589, RefSeq:WP_072182111.1, SO:0001217, UniParc:UPI0006A8991D, UniRef:UniRef100_UPI0006A8991D, UniRef
GO:0003723, GO:0045893, KEGG:K26931, RefSeq:WP_061186566.1, SO:0001217, UniParc:UPI0006A85D97, UniRef:UniRef100_A0A9Q4E4C2, UniRef:UniRef50_P15401, UniRef:UniRef90_P15401
GO:0000166, GO:0016757, GO:0046872, RefSeq:WP_014115643.1, SO:0001217, UniParc:UPI00022BB2E1, UniRef:UniRef100_G4NY26, UniRef:UniRef50_P25148, UniRef:UniRef90_P25148
RefSeq:WP_072182110.1, SO:0001217, UniParc:UPI0006A93949, UniRef:UniRef100_A0A9Q4E5M4, UniRef:UniRef50_P25149, UniRef:UniRef90_A0A9W5PEW9
GO:0003677, GO:0003700, GO:0006355, GO:0006950, SO:0001217, UniRef:UniRef50_P25150, UniRef:UniRef90_P25150
RFAM:RF00230, SO:0000140
EC:6.1.1.1, GO:0000166, GO:0003723, GO:0004831, GO:0005524, GO:0005829, GO:0006437, GO:0043039, KEGG:K01866, SO:0001217, UniRef:UniRef50_P25151, UniRef:UniRef90_P25151
GO:0004177, GO:0005576, GO:0006508, GO:0008235, GO:0046872, RefSeq:WP_087987352.1, SO:0001217, UniParc:UPI000B42DE40, UniRef:UniRef100_UPI000B42DE40, UniRef:UniRef50_P25152, UniRef:UniRef90_P25
BlastRules:WP_003244564, EC:2.7.6.5, GO:0005524, GO:0005525, GO:0008728, GO:0015970, GO:0016301, RefSeq:WP_003222250.1, SO:0001217, UniParc:UPI0001D2844C, UniRef:UniRef100_A0A9Q4DLP2, UniRef:UniF
EC:2.5.1.74, GO:0004659, GO:0005886, GO:0009234, GO:0016020, GO:0032194, GO:0042371, GO:0046428, SO:0001217, UniRef:UniRef50_P39582, UniRef:UniRef90_P39582
GO:0005886, SO:0001217, UniParc:UPI0001D2844F, UniRef:UniRef100_C0H3T3, UniRef:UniRef50_C0H3T3, UniRef:UniRef90_C0H3T3
EC:6.1.1.13, EC:6.2.1.54, GO:0005524, GO:0005737, GO:0019350, GO:0047473, GO:0070395, SO:0001217, UniRef:UniRef50_Q81G39, UniRef:UniRef90_P39581
EC:2.3.1.-, GO:0005886, GO:0016746, GO:0070395, KEGG:K03739, RefSeq:WP_061186573.1, SO:0001217, UniParc:UPI000772AEA8, UniRef:UniRef100_A0A9Q4HHW3, UniRef:UniRef50_P39580, UniRef:UniRef90_P39580
BlastRules:WP_000807310, BlastRules:WP_003227325, EC:6.1.1.13, GO:0005737, GO:0019350, GO:0036370, GO:0070395, GO:0071555, RefSeq:WP_003227325.1, SO:0001217, UniParc:UPI0000060C4B, UniRef:UniRef10
GO:0005886, GO:0070394, GO:0070395, KEGG:K03740, RefSeq:WP_326246863.1, SO:0001217, UniParc:UPI002DBCE376, UniRef:UniRef100_UPI002DBCE376, UniRef:UniRef50_P39578, UniRef:UniRef90_P39578
EC:1.-.-.-, GO:0005737, GO:0016020, GO:0016491, RefSeq:WP_072182085.1, SO:0001217, UniParc:UPI0006A9409D, UniRef:UniRef100_UPI0006A9409D, UniRef:UniRef50_P39577, UniRef:UniRef90_P39577
EC:2.6.1.42, GO:0004084, GO:0009081, GO:0009097, GO:0009098, GO:0009099, GO:0052655, GO:0052656, KEGG:K00826, SO:0001217, UniRef:UniRef50_P39576, UniRef:UniRef90_P39576
SO:0001217, UniRef:UniRef50_A0A9W5TUV7
SO:0001217, UniRef:UniRef50_A0A2X3D9V8
SO:0001217, UniRef:UniRef50_A0A7M2RFP2
SO:0001217, UniRef:UniRef50_UPI001E547A53
SO:0001217, UniRef:UniRef50_A0A5S9MI93
BlastRules:WP_003218755, EC:2.7.1.-, GO:0005737, GO:0008982, GO:0009401, GO:0016301, RefSeq:WP_003218755.1, SO:0001217, UniParc:UPI0000060C51, UniRef:UniRef100_P46318, UniRef:UniRef50_P46318, UniRe
BlastRules:WP_003243034, GO:0003723, GO:0006355, GO:0008982, GO:0009401, GO:0016301, SO:0001217, UniRef:UniRef50_P46321, UniRef:UniRef90_P46321
RefSeq:WP_061186616.1, SO:0001217, UniParc:UPI0006A86836, UniRef:UniRef100_A0A9Q4E674, UniRef:UniRef50_O32283, UniRef:UniRef90_O32283
EC:3.2.2.-, GO:0003677, GO:0003905, GO:0006284, KEGG:K03652, RefSeq:WP_072182082.1, SO:0001217, UniParc:UPI0006A87068, UniRef:UniRef100_UPI0006A87068, UniRef:UniRef50_P94378, UniRef:UniRef90_P9437
BlastRules:WP_003243042, EC:1.11.1.6, GO:0004096, GO:0005737, GO:0020037, GO:0042542, GO:0042744, GO:0046872, KEGG:K03781, RefSeq:WP_072182081.1, SO:0001217, UniParc:UPI0006A8D83B, UniRef:UniRef
GO:0005886, GO:0022857, RefSeq:WP_072565488.1, SO:0001217, UniParc:UPI00090967DB, UniRef:UniRef100_UPI00090967DB, UniRef:UniRef50_P94376, UniRef:UniRef90_P94376
GO:0005886, GO:0140359, RefSeq:WP_014115664.1, SO:0001217, UniParc:UPI00022BB46E, UniRef:UniRef100_G4NY51, UniRef:UniRef50_P94375, UniRef:UniRef90_P94375
SO:0001217, UniRef:UniRef50_P94374
GO:0005886, RefSeq:WP_003218770.1, SO:0001217, UniParc:UPI0001D28796, UniRef:UniRef100_A0A9Q4HA44, UniRef:UniRef50_P94373, UniRef:UniRef90_P94373
RefSeq:WP_014115666.1, SO:0001217, UniParc:UPI00022BB470, UniRef:UniRef100_G4NY54, UniRef:UniRef50_G4NY54, UniRef:UniRef90_G4NY54
RefSeq:WP_072183107.1, SO:0001217, UniParc:UPI0006A865B5, UniRef:UniRef100_UPI0006A865B5, UniRef:UniRef50_P94371, UniRef:UniRef90_A0A9W5PEV2
PFAM:PF04542.20
GO:0005886, GO:0022857, KEGG:K03457, RefSeq:WP_072183108.1, SO:0001217, UniParc:UPI0006A8656C, UniRef:UniRef100_A0A9Q4HJB7, UniRef:UniRef50_P94369, UniRef:UniRef90_P94369
EC:4.2.1.136, GO:0005524, GO:0046496, GO:0052855, GO:0052856, GO:0110051, RefSeq:WP_072183109.1, SO:0001217, UniParc:UPI0006A83D67, UniRef:UniRef100_UPI0006A83D67, UniRef:UniRef50_P94368, UniRef:U
EC:7.4.2.-, GO:0005524, GO:0005886, GO:0006865, GO:0016020, GO:0016887, GO:0033228, GO:0033230, GO:0034775, GO:0045454, GO:0050456, GO:0055051, GO:0140359, SO:0001217, UniRef:UniRef50_P94367, Uni
SO:0001217, UniRef:UniRef50_W7DCN8
SO:0001217, UniRef:UniRef50_W7CTX0
EC:7.1.1.-, EC:7.1.1.7, GO:0005886, GO:0009055, GO:0016682, GO:0019646, GO:0046872, GO:0070069, KEGG:K00426, SO:0001217, UniRef:UniRef50_P94365, UniRef:UniRef90_P94365
BlastRules:WP_000381950, BlastRules:WP_000448880, BlastRules:WP_001286162, BlastRules:WP_003243795, EC:1.10.3.-, EC:7.1.1.7, GO:0005886, GO:0009055, GO:0016682, GO:0019646, GO:0020037, GO:0046872, G
GO:0005886, GO:0006101, GO:0015531, KEGG:K11616, SO:0001217, UniRef:UniRef50_P94363, UniRef:UniRef90_P94363
SO:0001217, UniRef:UniRef50_A0A376FF37
SO:0001217, UniRef:UniRef50_P94362, UniRef:UniRef90_UPI00227E4DB6
SO:0001217, UniRef:UniRef50_O32284
SO:0001217, UniRef:UniRef50_P94361, UniRef:UniRef90_A0AAP3CKL0
RefSeq:WP_014115680.1, SO:0001217, UniParc:UPI00022BB601, UniRef:UniRef100_G4NY68, UniRef:UniRef50_G4NY68, UniRef:UniRef90_G4NY68
SO:0001217, UniRef:UniRef50_A0A1S2MF60, UniRef:UniRef90_A0A5D4N5D6
BlastRules:WP_003242648, EC:7.5.2.-, GO:0005524, GO:0015774, GO:0016887, GO:0055052, GO:0140359, RefSeq:WP_072183115.1, SO:0001217, UniParc:UPI0006A92E11, UniRef:UniRef100_UPI0006A92E11, UniRef:Ur
RefSeq:WP_061186586.1, SO:0001217, UniParc:UPI0007725B8B, UniRef:UniRef100_G4NYF2, UniRef:UniRef50_P94359, UniRef:UniRef90_G4NYF2
EC:1.2.1.3, GO:0004029, RefSeq:WP_114151073.1, SO:0001217, UniParc:UPI000DEFD1B9, UniRef:UniRef100_UPI000DEFD1B9, UniRef:UniRef50_P94358, UniRef:UniRef90_P94358
GO:0005886, RefSeq:WP_014115685.1, SO:0001217, UniParc:UPI00022BB606, UniRef:UniRef100_G4NYF4, UniRef:UniRef50_P94357, UniRef:UniRef90_P94357
RFAM:RF00442, SO:0000035
GO:0005576, SO:0001217, UniRef:UniRef50_P94356, UniRef:UniRef90_P94356
EC:5.1.3.2, GO:0003978, GO:0005829, GO:0005996, GO:0006012, RefSeq:WP_014115687.1, SO:0001217, UniParc:UPI00022BB608, UniRef:UniRef100_G4NYF6, UniRef:UniRef50_P55180, UniRef:UniRef90_P55180
GO:0005886, SO:0001217, UniRef:UniRef50_P55182, UniRef:UniRef90_P55182
EC:2.7.13.3, GO:0000155, GO:0005524, GO:0005886, GO:0046983, SO:0001217, UniRef:UniRef50_P55183, UniRef:UniRef90_P55183
BlastRules:WP_003243101, GO:0000160, GO:0003677, GO:0005737, GO:0006355, SO:0001217, UniRef:UniRef50_P55184, UniRef:UniRef90_P55184
EC:3.4.11.4, GO:0005829, GO:0006508, GO:0008237, GO:0008270, GO:0043171, GO:0045148, KEGG:K01258, SO:0001217, UniRef:UniRef50_P55179, UniRef:UniRef90_P55179
RefSeq:WP_072181678.1, SO:0001217, UniParc:UPI0006A8A6D3, UniRef:UniRef100_UPI0006A8A6D3, UniRef:UniRef50_P94352, UniRef:UniRef90_P94352
RFAM:RF00162, SO:0000140
EC:2.1.1.14, GO:0003871, GO:0008270, GO:0009086, KEGG:K00549, SO:0001217, UniRef:UniRef50_P42319, UniRef:UniRef90_P42319
RFAM:RF00162, SO:0000140
PFAM:PF01717.24
SO:0001217, UniRef:UniRef50_P42317
EC:2.8.3.5, GO:0008260, GO:0008410, RefSeq:WP_014115700.1, SO:0001217, UniParc:UPI00022BB615, UniRef:UniRef100_G4NYG9, UniRef:UniRef50_P56007, UniRef:UniRef90_P42316
EC:2.8.3.5, GO:0008260, RefSeq:WP_014115701.1, SO:0001217, UniParc:UPI00022BB616, UniRef:UniRef100_G4NYH0, UniRef:UniRef50_P56006, UniRef:UniRef90_P42315
SO:0001217, UniRef:UniRef50_A0A3N5ETZ4
EC:2.1.1.187, KEGG:K00563, SO:0001217, UniRef:UniRef50_P42313, UniRef:UniRef90_A0A9Q4HJC8
RFAM:RF00167, SO:0000035
SO:0001217, UniRef:UniRef50_A0A9W3KCR1
SO:0001217, UniRef:UniRef50_UPI003000036E, UniRef:UniRef90_UPI003000036E
RefSeq:WP_014115705.1, SO:0001217, UniParc:UPI00022BB61A, UniRef:UniRef100_G4NYH4, UniRef:UniRef50_Q65D66, UniRef:UniRef90_G4NYH4
RefSeq:WP_014115706.1, SO:0001217, UniParc:UPI00022BB61B, UniRef:UniRef100_G4NYH5, UniRef:UniRef50_P42310, UniRef:UniRef90_P42310
EC:1.11.1.6, GO:0004096, GO:0005829, GO:0006979, GO:0020037, GO:0030435, GO:0042744, GO:0046872, KEGG:K03781, SO:0001217, UniRef:UniRef50_P42234, UniRef:UniRef90_P42234
BlastRules:WP_003227205, GO:0005886, GO:0006101, GO:0015137, GO:0015293, GO:0022857, GO:0055085, RefSeq:WP_080376099.1, SO:0001217, UniParc:UPI0006A829D1, UniRef:UniRef100_UPI0006A829D1, UniRef
RefSeq:WP_061186619.1, SO:0001217, UniParc:UPI0006A8C080, UniRef:UniRef100_UPI0006A8C080, UniRef:UniRef50_P96696, UniRef:UniRef90_E0TXG6
RefSeq:WP_061186601.1, SO:0001217, UniParc:UPI0006A82FC6, UniRef:UniRef100_UPI0006A82FC6, UniRef:UniRef50_G4NYH9, UniRef:UniRef90_G4NYH9
SO:0001217, UniRef:UniRef50_A0A9W5PEW5, UniRef:UniRef90_A0A9W5PEW5
EC:2.7.1.-, KEGG:K02757, RefSeq:WP_061186603.1, SO:0001217, UniParc:UPI0006A8F3C4, UniRef:UniRef100_UPI0006A8F3C4, UniRef:UniRef50_A0AA95HY55, UniRef:UniRef90_A0A9W5PES4
BlastRules:WP_003242598, GO:0003723, GO:0006355, GO:0045893, KEGG:K03488, RefSeq:WP_014115714.1, SO:0001217, UniParc:UPI00022BB623, UniRef:UniRef100_G4NYI3, UniRef:UniRef50_P11989, UniRef:UniRef
SO:0001217, UniRef:UniRef50_P42307, UniRef:UniRef90_G4NYI4
GO:0005886, GO:0022857, SO:0001217, UniRef:UniRef50_P42306, UniRef:UniRef90_P42306
BlastRules:WP_000039360, BlastRules:WP_003243023, EC:3.6.4.13, GO:0000027, GO:0003689, GO:0003724, GO:0005524, GO:0005829, GO:0009409, GO:0016887, GO:0033592, GO:0034458, GO:0061775, GO:0140584,
EC:3.1.-.-, GO:0016787, RefSeq:WP_079287603.1, SO:0001217, UniParc:UPI0009A38846, UniRef:UniRef100_UPI0009A38846, UniRef:UniRef50_P42304, UniRef:UniRef90_P42304
RefSeq:WP_014115720.1, SO:0001217, UniParc:UPI00022BB773, UniRef:UniRef100_G4NYI9, UniRef:UniRef50_G4NYI9, UniRef:UniRef90_G4NYI9
RefSeq:WP_238528865.1, SO:0001217, UniParc:UPI000A83C6AD, UniRef:UniRef100_G4NYJ0, UniRef:UniRef50_G4NYJ0, UniRef:UniRef90_G4NYJ0
RefSeq:WP_014115722.1, SO:0001217, UniParc:UPI00022BB775, UniRef:UniRef100_G4NYJ1, UniRef:UniRef50_G4NYJ1, UniRef:UniRef90_G4NYJ1
SO:0001217, UniParc:UPI00022BB777, UniRef:UniRef100_G4NYJ3, UniRef:UniRef50_G4NYJ3, UniRef:UniRef90_G4NYJ3
SO:0001217, UniRef:UniRef50_G4NYJ4, UniRef:UniRef90_A0A7G7UDY6
SO:0001217, UniRef:UniRef50_G4NYJ5
SO:0001217, UniRef:UniRef50_B7GKW2

SO:0001217, UniRef:UniRef50_B7GLY8
GO:0005886, GO:0016020, SO:0001217, UniRef:UniRef50_Q07835, UniRef:UniRef90_Q07835
SO:0001217, UniRef:UniRef50_P42297, UniRef:UniRef90_P42297
BlastRules:WP_003243232, EC:3.2.1.86, GO:0005829, GO:0008422, GO:0008706, GO:0016052, RefSeq:WP_088680086.1, SO:0001217, UniParc:UPI000B52B4A3, UniRef:UniRef100_UPI000B52B4A3, UniRef:UniRef50_P40
EC:2.7.1.-, GO:0005886, GO:0008982, GO:0009401, GO:0015771, GO:0016301, GO:0090589, KEGG:K02757, SO:0001217, UniRef:UniRef50_P40739, UniRef:UniRef90_P40739
RefSeq:WP_072181655.1, SO:0001217, UniParc:UPI0006A87E67, UniRef:UniRef100_UPI0006A87E67, UniRef:UniRef50_UPI0006A87E67, UniRef:UniRef90_UPI0006A87E67
RefSeq:WP_041521106.1, SO:0001217, UniParc:UPI0002D2602E, UniRef:UniRef100_UPI0002D2602E, UniRef:UniRef50_A0A6I7FBH2, UniRef:UniRef90_A0AAX3RRG9
EC:3.1.-.-, KEGG:K21493, SO:0001217, UniRef:UniRef50_P42296, UniRef:UniRef90_D4G3S5
RefSeq:WP_213419891.1, SO:0001217, UniParc:UPI001B949151, UniRef:UniRef100_UPI001B949151, UniRef:UniRef50_P42295, UniRef:UniRef90_P42295
RefSeq:WP_014478447.1, SO:0001217, UniParc:UPI00022BA6EA, UniRef:UniRef100_A0A8I1WFG2, UniRef:UniRef50_P42294, UniRef:UniRef90_P42294
BlastRules:WP_003244268, EC:3.2.1.99, GO:0005576, GO:0031222, GO:0046558, GO:0046872, KEGG:K06113, RefSeq:WP_072181651.1, SO:0001217, UniParc:UPI0006A855CF, UniRef:UniRef100_UPI0006A855CF, UniRef
SO:0001217, UniRef:UniRef50_UPI000A95B93A, UniRef:UniRef50_UPI00207D485C, UniRef:UniRef90_UPI000A95B93A
SO:0001217, UniRef:UniRef50_A0A2U2P3Y4
BlastRules:WP_000926516, BlastRules:WP_003243875, GO:0003729, GO:0006547, GO:0010628, KEGG:K09683, RefSeq:WP_003243875.1, SO:0001217, UniParc:UPI0000ED91F1, UniRef:UniRef100_P10943, UniRef:UniRe
EC:4.3.1.3, GO:0004397, GO:0005737, GO:0006548, GO:0019556, GO:0019557, KEGG:K01745, SO:0001217, UniRef:UniRef50_P10944, UniRef:UniRef90_P10944
EC:4.2.1.49, GO:0005737, GO:0006548, GO:0016153, GO:0019556, GO:0019557, SO:0001217, UniRef:UniRef50_P25080, UniRef:UniRef90_P25503
EC:4.2.1.49, GO:0006548, GO:0016153, KEGG:K01712, SO:0001217, UniRef:UniRef50_A0A356TQ99, UniRef:UniRef90_UPI001923AE1C
SO:0001217, UniRef:UniRef50_UPI001C4855CB
SO:0001217, UniRef:UniRef50_Q54249
PFAM:PF01979.26, PFAM:PF07969.17
EC:3.5.3.8, GO:0008783, GO:0019556, GO:0019557, GO:0030145, GO:0033389, GO:0050415, KEGG:K01479, SO:0001217, UniRef:UniRef50_P42068, UniRef:UniRef90_P42068
GO:0003333, GO:0005886, GO:0015171, GO:0016020, KEGG:K16236, SO:0001217, UniRef:UniRef50_P42087, UniRef:UniRef90_P42087
COG:COG0213, COG:F, EC:2.4.2.2, GO:0004645, GO:0004850, GO:0005829, GO:0006206, GO:0006213, GO:0009032, GO:0016154, GO:0046872, GO:0047847, RefSeq:WP_014115742.1, SO:0001217, UniParc:UPI00022B
BlastRules:WP_000376337, BlastRules:WP_003244143, GO:0005337, GO:0005886, GO:0015293, GO:1901642, KEGG:K11535, RefSeq:WP_014115743.1, SO:0001217, UniParc:UPI00022BB78A, UniRef:UniRef100_G4NYL3
EC:4.1.2.4, GO:0004139, GO:0005737, GO:0006018, GO:0009264, GO:0016052, KEGG:K01619, RefSeq:WP_072565535.1, SO:0001217, UniParc:UPI00090CAE0A, UniRef:UniRef100_UPI00090CAE0A, UniRef:UniRef50_P3
BlastRules:WP_003227124, GO:0003677, GO:0030246, GO:0042802, SO:0001217, UniRef:UniRef50_P39140, UniRef:UniRef90_P39140
SO:0001217, UniRef:UniRef50_P39139, UniRef:UniRef90_A0A6H0WPS2
SO:0001217, UniRef:UniRef50_UPI002282F86F
GO:0016829, SO:0001217, UniRef:UniRef50_P54956, UniRef:UniRef90_P54956
BlastRules:WP_003243840, EC:3.5.1.-, GO:0016787, GO:0019344, GO:0046872, KEGG:K21613, RefSeq:WP_072181640.1, SO:0001217, UniParc:UPI0006A89479, UniRef:UniRef100_UPI0006A89479, UniRef:UniRef50_P54
EC:7.4.2.-, GO:0005524, GO:0005886, GO:0015424, GO:0016887, RefSeq:WP_014115751.1, SO:0001217, UniParc:UPI00022BB8F7, UniRef:UniRef100_G4NYM1, UniRef:UniRef50_P54954, UniRef:UniRef90_P54954
GO:0005886, GO:0015184, GO:0015811, GO:0043190, RefSeq:WP_072181639.1, SO:0001217, UniParc:UPI0006A8E1C6, UniRef:UniRef100_UPI0006A8E1C6, UniRef:UniRef50_P54953, UniRef:UniRef90_P54953
GO:0005886, GO:0006865, GO:0045121, SO:0001217, UniRef:UniRef50_P54952, UniRef:UniRef90_P54952
EC:2.3.1.-, GO:0016747, GO:0019344, SO:0001217, UniRef:UniRef50_P54951, UniRef:UniRef90_P54951
EC:1.14.13.-, GO:0004497, GO:0016705, GO:0019344, KEGG:K24116, SO:0001217, UniRef:UniRef50_P54950, UniRef:UniRef90_P54950
SO:0001217, UniRef:UniRef50_P54948, UniRef:UniRef90_G4NYM6
EC:3.1.3.-, EC:3.1.3.23, GO:0000287, GO:0005829, GO:0016787, GO:0016791, KEGG:K21064, RefSeq:WP_326197680.1, SO:0001217, UniParc:UPI002DBADAAF, UniRef:UniRef100_UPI002DBADAAF, UniRef:UniRef50_P54
SO:0001217, UniRef:UniRef50_P54946, UniRef:UniRef90_UPI00227F5CA8
SO:0001217, UniRef:UniRef50_P54945, UniRef:UniRef90_G4NYM9
RefSeq:WP_014115760.1, SO:0001217, UniParc:UPI00022BB900, UniRef:UniRef100_G4NYN0, UniRef:UniRef50_P54944, UniRef:UniRef90_P54944
RefSeq:WP_014115761.1, SO:0001217, UniParc:UPI00022BB901, UniRef:UniRef100_G4NYW1, UniRef:UniRef50_P54943, UniRef:UniRef90_P54943
GO:0005886, RefSeq:WP_003243293.1, SO:0001217, UniParc:UPI000195C731, UniRef:UniRef100_P54942, UniRef:UniRef50_P54942, UniRef:UniRef90_P54942
GO:0005886, GO:0030288, GO:0045121, GO:1901678, RefSeq:WP_014115762.1, SO:0001217, UniParc:UPI00022BB902, UniRef:UniRef100_G4NYW3, UniRef:UniRef50_P54941, UniRef:UniRef90_P54941
RefSeq:WP_061186823.1, SO:0001217, UniParc:UPI0007726851, UniRef:UniRef100_A0A9Q4E8T4, UniRef:UniRef50_P54940, UniRef:UniRef90_P54940
BlastRules:WP_003244410, KEGG:K11636, SO:0001217, UniRef:UniRef50_P42424, UniRef:UniRef90_A0A9Q4DMC6
BlastRules:WP_003243557, GO:0005524, GO:0005886, GO:0016887, GO:0022857, GO:0055085, RefSeq:WP_014115765.1, SO:0001217, UniParc:UPI00022BB905, UniRef:UniRef100_G4NYW6, UniRef:UniRef50_P42423, U
BlastRules:WP_003243885, EC:2.7.13.3, GO:0000155, GO:0005524, GO:0005886, KEGG:K11633, RefSeq:WP_072181630.1, SO:0001217, UniParc:UPI0006A94091, UniRef:UniRef100_UPI0006A94091, UniRef:UniRef50_P4
GO:0000156, GO:0000976, GO:0005829, GO:0006355, GO:0032993, KEGG:K11634, SO:0001217, UniRef:UniRef50_P42421, UniRef:UniRef90_P42421
BlastRules:WP_003242766, EC:4.1.2.13, EC:4.1.2.29, GO:0004332, GO:0006096, GO:0008270, GO:0030388, GO:0047441, KEGG:K01624, SO:0001217, UniRef:UniRef50_P42420, UniRef:UniRef90_P42420
BlastRules:WP_010898471, BlastRules:WP_012118841, EC:5.3.99.11, GO:0016853, GO:0046872, RefSeq:WP_072181628.1, SO:0001217, UniParc:UPI0006A8A8BB, UniRef:UniRef100_UPI0006A8A8BB, UniRef:UniRef50_P
COG:COG1082, COG:G, SO:0001217, UniRef:UniRef50_P42418, UniRef:UniRef90_P42418
BlastRules:WP_003244482, EC:1.1.1.18, EC:1.1.1.369, GO:0000166, GO:0019310, GO:0050112, SO:0001217, UniRef:UniRef50_P26935, UniRef:UniRef90_P26935
BlastRules:WP_003244597, GO:0005886, GO:0016020, GO:0022857, GO:0055085, KEGG:K06610, SO:0001217, UniRef:UniRef50_P42417, UniRef:UniRef90_P42417
EC:4.2.1.44, GO:0019310, GO:0030145, GO:0050114, KEGG:K03335, RefSeq:WP_114151031.1, SO:0001217, UniParc:UPI000DEFA28E, UniRef:UniRef100_UPI000DEFA28E, UniRef:UniRef50_P42416, UniRef:UniRef90_P42
COG:COG3962, COG:G, EC:3.7.1.22, GO:0000287, GO:0003984, GO:0005948, GO:0009097, GO:0009099, GO:0016823, GO:0019310, GO:0030976, GO:0050660, GO:0102481, KEGG:K03336, SO:0001217, UniRef:UniRef5
COG:COG0524, COG:G, EC:2.7.1.92, GO:0005524, GO:0019310, GO:0047590, KEGG:K03338, RefSeq:WP_014115775.1, SO:0001217, UniParc:UPI00022BB90F, UniRef:UniRef100_G4NYX6, UniRef:UniRef50_P42414, UniF
COG:COG3718, COG:G, EC:5.3.1.30, GO:0008880, GO:0016861, GO:0019310, GO:0102482, KEGG:K03337, RefSeq:WP_061186814.1, SO:0001217, UniParc:UPI00077297C3, UniRef:UniRef100_UPI00077297C3, UniRef:U
BlastRules:WP_003243682, COG:COG1012, COG:I, EC:1.2.1.27, GO:0004491, GO:0006210, GO:0006574, GO:0018478, GO:0019310, SO:0001217, UniRef:UniRef50_P42412, UniRef:UniRef90_P42412
BlastRules:WP_003227050, GO:0003677, GO:0003700, KEGG:K06608, RefSeq:WP_061186813.1, SO:0001217, UniParc:UPI0006A7F815, UniRef:UniRef100_A0A9Q4E8U3, UniRef:UniRef50_P46337, UniRef:UniRef90_P4633
EC:1.1.1.-, GO:0005829, GO:0016491, KEGG:K06607, RefSeq:WP_019716280.1, SO:0001217, UniParc:UPI0002FED1BC, UniRef:UniRef100_A0A9Q4EBR0, UniRef:UniRef50_P46336, UniRef:UniRef90_P46336
GO:0005886, SO:0001217, UniRef:UniRef50_P46335, UniRef:UniRef90_P46335
RefSeq:WP_326229387.1, SO:0001217, UniParc:UPI002DBC98CA, UniRef:UniRef100_UPI002DBC98CA, UniRef:UniRef50_P46334, UniRef:UniRef90_P46334
GO:0005886, GO:0015293, GO:0016020, GO:0055085, RefSeq:WP_014115782.1, SO:0001217, UniParc:UPI00022BB916, UniRef:UniRef100_G4NYY3, UniRef:UniRef50_A0A0H2VG78, UniRef:UniRef90_P46333
GO:0005524, GO:0005829, GO:0006457, GO:0006974, GO:0009408, GO:0016887, GO:0051082, GO:0140662, SO:0001217, UniRef:UniRef50_P46208, UniRef:UniRef90_P46208
GO:0005886, RefSeq:WP_014115784.1, SO:0001217, UniParc:UPI00022BB918, UniRef:UniRef100_G4NYY5, UniRef:UniRef50_P46332, UniRef:UniRef90_P46332
EC:1.-.-.-, GO:0016491, SO:0001217, UniRef:UniRef50_P46331, UniRef:UniRef90_P46331
SO:0001217, UniRef:UniRef50_E0TYH4, UniRef:UniRef90_E0TYH4
SO:0001217, UniRef:UniRef50_P46330, UniRef:UniRef90_UPI000B45510F
EC:1.2.1.3, GO:0004029, GO:0005737, GO:0006081, RefSeq:WP_061186807.1, SO:0001217, UniParc:UPI0006A8F08B, UniRef:UniRef100_UPI0006A8F08B, UniRef:UniRef50_P46329, UniRef:UniRef90_P46329
SO:0001217, UniRef:UniRef50_A0A2M8T0B2, UniRef:UniRef90_G4NYY9
SO:0001217, UniRef:UniRef50_P42109, UniRef:UniRef90_A0A9W5LMB1
RefSeq:WP_014115790.1, SO:0001217, UniParc:UPI00022BBAAF, UniRef:UniRef100_G4NYZ1, UniRef:UniRef50_P42108, UniRef:UniRef90_A0AAP1H655
GO:0005886, KEGG:K07148, RefSeq:WP_014115791.1, SO:0001217, UniParc:UPI00022BBAB0, UniRef:UniRef100_G4NYZ2, UniRef:UniRef50_P42107, UniRef:UniRef90_P42107
EC:1.13.11.24, GO:0008127, GO:0046872, KEGG:K07155, RefSeq:WP_072565560.1, SO:0001217, UniParc:UPI00090BF246, UniRef:UniRef100_A0A9Q4E3E6, UniRef:UniRef50_P42106, UniRef:UniRef90_P42106
BlastRules:WP_003227022, GO:0003677, GO:0006355, RefSeq:WP_014115793.1, SO:0001217, UniParc:UPI00022BBAB2, UniRef:UniRef100_G4NYZ4, UniRef:UniRef50_O34619, UniRef:UniRef90_P42105
EC:1.-.-.-, GO:0005829, GO:0016491, RefSeq:WP_072182351.1, SO:0001217, UniParc:UPI0006A86B0C, UniRef:UniRef100_UPI0006A86B0C, UniRef:UniRef50_O32291, UniRef:UniRef90_O32291
GO:0003677, GO:0003700, GO:0006355, GO:0006950, SO:0001217, UniRef:UniRef50_P42103, UniRef:UniRef90_P42103
GO:0005886, RefSeq:WP_041521109.1, SO:0001217, UniParc:UPI0005A20D63, UniRef:UniRef100_A0A9Q4DZT7, UniRef:UniRef50_C0H3T7, UniRef:UniRef90_C0H3T7
GO:0005886, RefSeq:WP_072182353.1, SO:0001217, UniParc:UPI0006A86AB9, UniRef:UniRef100_UPI0006A86AB9, UniRef:UniRef50_P42102, UniRef:UniRef90_P42102
GO:0005886, GO:0006814, GO:0006885, GO:0015385, GO:0015386, GO:0016020, GO:0051453, GO:0098719, GO:1902600, SO:0001217, UniRef:UniRef50_Q9LCB5, UniRef:UniRef90_Q9LCB5
EC:2.-.-.-, GO:0016740, KEGG:K19426, SO:0001217, UniRef:UniRef50_P42101, UniRef:UniRef90_P42101
EC:2.7.1.165, KEGG:K00865, SO:0001217, UniRef:UniRef50_P23524, UniRef:UniRef90_G4NZ01
BlastRules:WP_000167476, BlastRules:WP_003243132, GO:0003677, GO:0003700, GO:0019521, KEGG:K11476, RefSeq:WP_072182357.1, SO:0001217, UniParc:UPI0006A8AF2B, UniRef:UniRef100_UPI0006A8AF2B, UniF
EC:2.7.1.12, GO:0005524, GO:0009255, GO:0019521, GO:0046177, GO:0046316, KEGG:K25031, SO:0001217, UniRef:UniRef50_P12011, UniRef:UniRef90_P12011
BlastRules:WP_001131724, BlastRules:WP_003243139, GO:0005886, GO:0015128, GO:0035429, GO:0046177, SO:0001217, UniRef:UniRef50_P12012, UniRef:UniRef90_P12012
EC:1.1.1.343, EC:1.1.1.44, GO:0004616, GO:0005737, GO:0005829, GO:0006098, GO:0009051, GO:0046177, GO:0050661, KEGG:K00033, RefSeq:WP_014115804.1, SO:0001217, UniParc:UPI00022BBABD, UniRef:UniRef
COG:COG0450, COG:V, EC:1.11.1.26, GO:0005829, GO:0006979, GO:0008379, GO:0042744, GO:0045454, GO:0102039, RefSeq:WP_014115806.1, SO:0001217, UniParc:UPI00022BBABF, UniRef:UniRef100_G4NZ07, UniF
COG:COG3634, COG:V, EC:7.1.1.2, GO:0000302, GO:0004791, GO:0005829, GO:0005886, GO:0008137, GO:0008785, GO:0045454, GO:0050660, GO:0051287, GO:0102039, SO:0001217, UniRef:UniRef50_P42974, UniRe
SO:0001217, UniRef:UniRef50_D7UUD9, UniRef:UniRef90_G4NZ11
BlastRules:WP_003226994, EC:3.2.1.86, GO:0005829, GO:0008422, GO:0008706, GO:0016052, RefSeq:WP_079287660.1, SO:0001217, UniParc:UPI0009A2CD5C, UniRef:UniRef100_UPI0009A2CD5C, UniRef:UniRef50_P4
EC:2.7.1.-, KEGG:K02757, RefSeq:WP_088680255.1, SO:0001217, UniParc:UPI000B530301, UniRef:UniRef100_UPI000B530301, UniRef:UniRef50_A0A172ZC87, UniRef:UniRef90_G4NZ13
GO:0003677, GO:0003700, GO:0045892, KEGG:K03489, RefSeq:WP_088680254.1, SO:0001217, UniParc:UPI000B533D50, UniRef:UniRef100_UPI000B533D50, UniRef:UniRef50_Q45591, UniRef:UniRef90_Q45591
GO:0005886, KEGG:K16916, RefSeq:WP_014115815.1, SO:0001217, UniParc:UPI00022BBAC8, UniRef:UniRef100_G4NZ16, UniRef:UniRef50_Q45592, UniRef:UniRef90_Q45592
GO:0005524, GO:0016887, GO:0042626, GO:0043190, KEGG:K16917, RefSeq:WP_014115816.1, SO:0001217, UniParc:UPI00022BBAC9, UniRef:UniRef100_G4NZ17, UniRef:UniRef50_Q45593, UniRef:UniRef90_Q45593
KEGG:K16922, RefSeq:WP_014115817.1, SO:0001217, UniParc:UPI00022BBACA, UniRef:UniRef100_G4NZ18, UniRef:UniRef50_Q45594, UniRef:UniRef90_G4NZ18
GO:0003824, GO:0031179, GO:0046872, GO:0051539, GO:1904047, RefSeq:WP_014115818.1, SO:0001217, UniParc:UPI00022BBACB, UniRef:UniRef100_G4NZ19, UniRef:UniRef50_Q45595, UniRef:UniRef90_Q45595
RefSeq:WP_014115819.1, SO:0001217, UniParc:UPI00022BBACC, UniRef:UniRef100_G4NZ20, UniRef:UniRef50_Q45596, UniRef:UniRef90_Q45596
BlastRules:WP_003243329, EC:3.1.3.11, GO:0006094, GO:0042132, KEGG:K04041, RefSeq:WP_326199982.1, SO:0001217, UniParc:UPI002DBDD4F5, UniRef:UniRef100_UPI002DBDD4F5, UniRef:UniRef50_Q45597, UniRe
RefSeq:WP_014115822.1, SO:0001217, UniParc:UPI00022BBC5D, UniRef:UniRef100_G4NZ23, UniRef:UniRef50_A0A1V4I6G3, UniRef:UniRef90_G4NZ23
RefSeq:WP_014115823.1, SO:0001217, UniParc:UPI00022BBC5E, UniRef:UniRef100_G4NZ24, UniRef:UniRef50_A0AAN3M8M4, UniRef:UniRef90_G4NZ24
EC:2.1.1.177, GO:0005737, GO:0006364, GO:0008168, GO:0070038, SO:0001217, UniRef:UniRef50_Q45601, UniRef:UniRef90_Q45601
RefSeq:WP_003236479.1, SO:0001217, UniParc:UPI000287C8D4, UniRef:UniRef100_A0A9Q4HEK5, UniRef:UniRef50_C0H3T9, UniRef:UniRef90_C0H3T9
EC:1.2.1.46, GO:0008270, GO:0016491, GO:0018467, GO:0046292, KEGG:K00148, SO:0001217, UniRef:UniRef50_Q45604, UniRef:UniRef90_Q45604
RefSeq:WP_019716326.1, SO:0001217, UniParc:UPI0003006A71, UniRef:UniRef100_UPI0003006A71, UniRef:UniRef50_A0AAP3CJC8, UniRef:UniRef90_UPI001F61D97C
SO:0001217, UniRef:UniRef50_Q45605, UniRef:UniRef90_G4NZ29
SO:0001217, UniRef:UniRef50_Q45606, UniRef:UniRef90_G4NZ30
SO:0001217, UniRef:UniRef50_Q45607, UniRef:UniRef90_Q45607
EC:2.3.1.-, GO:0016747, SO:0001217, UniRef:UniRef50_O32293, UniRef:UniRef90_O32293
BlastRules:WP_003243386, GO:0005737, RefSeq:WP_014115832.1, SO:0001217, UniParc:UPI00022BBC67, UniRef:UniRef100_G4NZ33, UniRef:UniRef50_O32294, UniRef:UniRef90_O32294
GO:0005576, GO:0005737, KEGG:K06356, RefSeq:WP_003219133.1, SO:0001217, UniParc:UPI0001D28B01, UniRef:UniRef100_A0A9Q4DLY2, UniRef:UniRef50_O32295, UniRef:UniRef90_O32295
EC:3.5.3.1, GO:0000050, GO:0004053, GO:0005737, GO:0006525, GO:0019547, GO:0030145, GO:0046872, KEGG:K01476, RefSeq:WP_014115833.1, SO:0001217, UniParc:UPI00022BBC68, UniRef:UniRef100_G4NZ35, U
GO:0003333, GO:0005886, GO:0006525, GO:0015171, GO:0016020, KEGG:K02205, SO:0001217, UniRef:UniRef50_P39137, UniRef:UniRef90_P39137
BlastRules:WP_000616649, BlastRules:WP_003242970, EC:2.6.1.13, GO:0004587, GO:0030170, KEGG:K00819, SO:0001217, UniRef:UniRef50_Q89RB7, UniRef:UniRef90_D4G437
BlastRules:WP_003244510, GO:0000160, GO:0005524, GO:0006355, GO:0006525, GO:0016887, GO:0043565, KEGG:K06714, RefSeq:WP_014115836.1, SO:0001217, UniParc:UPI00022BBC6B, UniRef:UniRef100_G4NZ38
SO:0001217, UniRef:UniRef50_A0A2U3KIP8
SO:0001217, UniRef:UniRef50_A0A5S9MKJ9
EC:3.4.21.-, GO:0004252, GO:0005886, GO:0006508, SO:0001217, UniRef:UniRef50_P39668, UniRef:UniRef90_P39668
EC:3.1.11.-, GO:0004527, GO:0046872, RefSeq:WP_079287674.1, SO:0001217, UniParc:UPI0009A46F33, UniRef:UniRef100_UPI0009A46F33, UniRef:UniRef50_C0SP91, UniRef:UniRef90_C0SP91
BlastRules:WP_003244037, GO:0005886, RefSeq:WP_014115843.1, SO:0001217, UniParc:UPI00022BBC72, UniRef:UniRef100_G4NZC6, UniRef:UniRef50_Q45612, UniRef:UniRef90_Q45612
BlastRules:WP_003242498, GO:0005886, GO:0046872, SO:0001217, UniRef:UniRef50_Q794W0, UniRef:UniRef90_Q794W0
SO:0001217, UniRef:UniRef50_A0A5S9MJ87
PFAM:PF00672.30, PFAM:PF23846.1
BlastRules:WP_000971872, BlastRules:WP_003244363, COG:COG0745, COG:TK, GO:0000156, GO:0000976, GO:0005829, GO:0006355, GO:0032993, SO:0001217, UniParc:UPI00019763B6, UniRef:UniRef100_P37478, Ui
SO:0000267
SO:0000256
SO:0000259
SO:0000265
COG:COG0104, COG:F, EC:6.3.4.4, GO:0000287, GO:0004019, GO:0005525, GO:0005737, GO:0009152, GO:0044208, GO:0046040, KEGG:K01939, RefSeq:WP_024713335.1, SO:0001217, UniParc:UPI00046340A3, UniRe
SO:0001217, UniRef:UniRef50_P37479, UniRef:UniRef90_E0TZB7
COG:COG0305, COG:L, EC:3.6.4.12, EC:5.6.2.3, GO:0003677, GO:0003678, GO:0003689, GO:0005524, GO:0005829, GO:0006260, GO:0006268, GO:0006269, GO:0009295, GO:0009378, GO:0016853, GO:0016887, GO:0
SO:0001217, UniParc:UPI00022BBC79, UniRef:UniRef100_G4NZD4, UniRef:UniRef50_P37480, UniRef:UniRef90_P37480
RefSeq:WP_014115851.1, SO:0001217, UniParc:UPI00022BBC7A, UniRef:UniRef100_G4NZD5, UniRef:UniRef50_O32296, UniRef:UniRef90_G4NZD5
RefSeq:WP_003242786.1, SO:0001217, UniParc:UPI0000060D0C, UniRef:UniRef100_P37481, UniRef:UniRef50_P37481, UniRef:UniRef90_P37481
SO:0001217, UniRef:UniRef50_P37482
SO:0001217, UniRef:UniRef50_A0A356PB71

BlastRules:WP_001081643, BlastRules:WP_003242517, EC:3.1.4.59, GO:0003676, GO:0005886, GO:0016787, GO:0030435, GO:0046872, GO:0106409, KEGG:K22927, RefSeq:WP_088680241.1, SO:0001217, UniParc:UPI
SO:0001217, UniRef:UniRef50_P37485, UniRef:UniRef90_P37485
GO:0005886, RefSeq:WP_014115856.1, SO:0001217, UniParc:UPI00022BA4A9, UniRef:UniRef100_G4NZE2, UniRef:UniRef50_C0H3U2, UniRef:UniRef90_C0H3U2
BlastRules:WP_000121384, BlastRules:WP_003243364, GO:0030435, KEGG:K06329, RefSeq:WP_014115857.1, SO:0001217, UniParc:UPI00022BA4AA, UniRef:UniRef100_G4NZE3, UniRef:UniRef50_P23261, UniRef:UniRe
BlastRules:WP_003242487, GO:0003677, RefSeq:WP_014115858.1, SO:0001217, UniParc:UPI00022BA4AB, UniRef:UniRef100_G4NZE4, UniRef:UniRef50_P96673, UniRef:UniRef90_P37486
EC:3.6.1.1, GO:0004427, GO:0005737, GO:0030145, KEGG:K15986, RefSeq:WP_014666106.1, SO:0001217, UniParc:UPI00025988D2, UniRef:UniRef100_A0A9Q4DZT2, UniRef:UniRef50_P37487, UniRef:UniRef90_P37487
GO:0005886, RefSeq:WP_088680240.1, SO:0001217, UniParc:UPI000B5324C0, UniRef:UniRef100_UPI000B5324C0, UniRef:UniRef50_P37488, UniRef:UniRef90_P37488
SO:0001217, UniRef:UniRef50_Q62Y65, UniRef:UniRef90_Q62Y65
GO:0005886, GO:0022857, RefSeq:WP_072183182.1, SO:0001217, UniParc:UPI0006A92360, UniRef:UniRef100_UPI0006A92360, UniRef:UniRef50_P37489, UniRef:UniRef90_P37489
SO:0001217, UniRef:UniRef50_T0N241, UniRef:UniRef90_D4G474
SO:0001217, UniRef:UniRef50_A0A0D1JCP1, UniRef:UniRef90_A0A0D1JCP1
SO:0001217, UniRef:UniRef50_UPI0027E4E691
EC:3.-.-.-, GO:0016787, GO:0046872, RefSeq:WP_014115871.1, SO:0001217, UniParc:UPI00022BA4B8, UniRef:UniRef100_G4NZF7, UniRef:UniRef50_P37502, UniRef:UniRef90_P37502
RefSeq:WP_072183180.1, SO:0001217, UniParc:UPI0006A8A9E2, UniRef:UniRef100_UPI0006A8A9E2, UniRef:UniRef50_J8DF57, UniRef:UniRef90_G4NZF8
GO:0003677, GO:0003700, RefSeq:WP_003236585.1, SO:0001217, UniParc:UPI0002ABC21C, UniRef:UniRef100_A0A9W5PF08, UniRef:UniRef50_P37503, UniRef:UniRef90_P37503
SO:0001217, UniRef:UniRef50_P37504, UniRef:UniRef90_A0A9Q4DZU2
SO:0001217, UniRef:UniRef50_P37505, UniRef:UniRef90_A0A2M8T021
RefSeq:WP_014115877.1, SO:0001217, UniParc:UPI00022BA4BE, UniRef:UniRef100_G4NZG3, UniRef:UniRef50_D5DX40, UniRef:UniRef90_E0TTV2
KEGG:K09936, SO:0001217, UniRef:UniRef50_B1YEL8, UniRef:UniRef90_UPI000EF469D8
RefSeq:WP_014115879.1, SO:0001217, UniParc:UPI00022BA4C0, UniRef:UniRef100_A0A2M8T033, UniRef:UniRef50_A0A150KUG4, UniRef:UniRef90_A0A0M2PIL5
SO:0001217, UniRef:UniRef50_A0A971Q4H8, UniRef:UniRef90_UPI0020137C31
SO:0001217, UniRef:UniRef50_A0A5S9LZB9, UniRef:UniRef90_UPI00274270AA
GO:0004222, GO:0016020, GO:0071586, RefSeq:WP_090556820.1, SO:0001217, UniParc:UPI000B850FBD, UniRef:UniRef100_UPI000B850FBD, UniRef:UniRef50_P37513, UniRef:UniRef90_P37513
GO:0005886, GO:0022857, RefSeq:WP_072183176.1, SO:0001217, UniParc:UPI0006A943C3, UniRef:UniRef100_A0A9Q4E0P1, UniRef:UniRef50_P37514, UniRef:UniRef90_P37514
EC:2.3.1.79, GO:0005829, GO:0008374, GO:0008925, KEGG:K00661, SO:0001217, UniRef:UniRef50_P37515, UniRef:UniRef90_P37515
RefSeq:WP_014115884.1, SO:0001217, UniParc:UPI00022BA4C5, UniRef:UniRef100_G4NZH0, UniRef:UniRef50_D4G492, UniRef:UniRef90_D4G492
GO:0000976, GO:0003700, GO:0006355, SO:0001217, UniRef:UniRef50_P37517, UniRef:UniRef90_P37517
EC:3.1.11.2, GO:0003677, GO:0003906, GO:0004519, GO:0005737, GO:0006281, GO:0006284, GO:0008081, GO:0008311, GO:0046872, KEGG:K01142, RefSeq:WP_014115886.1, SO:0001217, UniParc:UPI00022BA4C7, U
COG:COG0238, COG:J, GO:0000314, GO:0003735, GO:0006412, GO:0022627, GO:0070181, KEGG:K02963, RefSeq:WP_003219224.1, SO:0001217, UniParc:UPI00001B0969, UniRef:UniRef100_P21475, UniRef:UniRef50_
COG:COG0629, COG:L, GO:0003697, GO:0005737, GO:0006260, GO:0006281, GO:0006310, GO:0009295, RefSeq:WP_014115887.1, SO:0001217, UniParc:UPI00022BA4C8, UniRef:UniRef100_G4NZH3, UniRef:UniRef50_
GO:0000314, GO:0003735, GO:0005737, GO:0005840, GO:0006412, GO:0022627, GO:0070181, GO:1990904, KEGG:K02990, RefSeq:WP_014115888.1, SO:0001217, UniParc:UPI00022BA4C9, UniRef:UniRef100_G4NZH4
COG:COG0012, COG:J, GO:0005524, GO:0005525, GO:0005737, GO:0016887, GO:0043023, GO:0046872, RefSeq:WP_053407040.1, SO:0001217, UniParc:UPI0006A93CA3, UniRef:UniRef100_UPI0006A93CA3, UniRef:Ur
EC:1.-.-.-, GO:0016491, GO:0043546, GO:0046872, GO:0051536, SO:0001217, UniRef:UniRef50_P37519, UniRef:UniRef90_P37519
RefSeq:WP_003219236.1, SO:0001217, UniParc:UPI0001D28C94, UniRef:UniRef100_A0A9Q4E0M2, UniRef:UniRef50_C0H3V8, UniRef:UniRef90_C0H3V8
RefSeq:WP_014115892.1, SO:0001217, UniParc:UPI00022BA4CD, UniRef:UniRef100_G4NZH9, UniRef:UniRef50_P37520, UniRef:UniRef90_P37520
SO:0001217, UniRef:UniRef50_P37521, UniRef:UniRef90_P37521
BlastRules:WP_001051841, BlastRules:WP_003226832, GO:0003677, GO:0005694, GO:0007059, GO:0030435, GO:0043590, GO:0045881, KEGG:K03497, RefSeq:WP_014115894.1, SO:0001217, UniParc:UPI00022BA4CF,
BlastRules:WP_000516114, BlastRules:WP_003219244, COG:COG1192, COG:DN, EC:3.6.-.-, GO:0003689, GO:0005524, GO:0016887, GO:0030435, GO:0042174, GO:0061775, GO:0140584, GO:0140665, GO:0140849, KE
SO:0001217, UniParc:UPI00022BA4D0, UniRef:UniRef100_G4NZI3, UniRef:UniRef50_P37523, UniRef:UniRef90_G4NZI3
GO:0000917, GO:0003677, GO:0005694, GO:0005737, GO:0007059, GO:0009295, GO:0032466, GO:0043565, GO:0045881, KEGG:K03497, RefSeq:WP_003219249.1, SO:0001217, UniParc:UPI0001D28C99, UniRef:UniRef
EC:2.1.1.-, EC:2.1.1.170, GO:0005829, GO:0008168, GO:0031167, GO:0046118, GO:0070043, KEGG:K03501, RefSeq:WP_061187099.1, SO:0001217, UniParc:UPI0006A7FA4A, UniRef:UniRef100_A0A9Q4HEW6, UniRef:Ur
GO:0002098, GO:0005829, GO:0008033, GO:0030488, GO:0050660, KEGG:K03495, RefSeq:WP_003226825.1, SO:0001217, UniParc:UPI0000060D41, UniRef:UniRef100_P25812, UniRef:UniRef50_Q8Y3M5, UniRef:UniRef
EC:3.6.-.-, GO:0002098, GO:0003924, GO:0005525, GO:0005829, GO:0006400, GO:0030488, GO:0046872, RefSeq:WP_014115897.1, SO:0001217, UniParc:UPI00022BA62F, UniRef:UniRef100_G4NZI7, UniRef:UniRef50_P
GO:0003676, GO:0003723, GO:0005737, GO:0008360, GO:0009252, GO:0071555, RefSeq:WP_061187098.1, SO:0001217, UniParc:UPI0006A86D3B, UniRef:UniRef100_UPI0006A86D3B, UniRef:UniRef50_Q01620, UniRef:
GO:0005886, GO:0015031, GO:0032977, GO:0051205, KEGG:K03217, SO:0001217, UniRef:UniRef50_Q01625, UniRef:UniRef90_Q01625
SO:0001217, UniRef:UniRef50_A8FJG3
