# Supplementary material for: Genomic insights and evaluation of Bacillus spizizenii Endophyte from Solanum elaeagnifolium Cav against multidrug-resistant pathogens
Source: Front Cell Infect Microbiol. 2026 Sep 3;16:1929148. doi: 10.3389/fcimb.2026.1929148 (PMC13581695; doi:10.3389/fcimb.2026.1929148)
Supplement: Supplementary file 2 [file DataSheet2.docx]

| 1 | 2 |
| --- | --- |
|  |  |
| 3 | 4 |
|  |  |
| 5 | 6 |
|  |  |

**Fig. S1.** Antimicrobial activity of S1,2,3, and 4 organic extracts against **1.** MDR *K.* pneumoniae36., **2.** MDR *K.*

pneumoniae22., **3.** *P****.*** *aeruginosa45.*, **4.** MDR *P. aeruginosa47.,* **5.** s. *aureus* 9*.*, **6.** MRSA

No 5 in all pictures indicates to DMSO.

| 7 | 8 |
| --- | --- |
|  |  |
| 9 | 10 |
|  |  |

**Fig. S1.** Antimicrobial activity of S1,2,3, and organic extracts against **7.** MRSA 5 ., **8.** S. *aureus* 4., **9.** *C.albicans* ( ATCC 10231). Antimicrobial activity of P organic extract against **10.** MRSA 5.

| 11 | 12 |
| --- | --- |
|  |  |
| 13 | 14 |
|  |  |
| 15 |  |
|  |  |

**Fig. S1.** Antimicrobial activity of S1,2,3, and organic extracts against **11.** MDR *P. aeruginosa*45., **12.** MDR *P. aeruginosa* 45., **13.** S. *aureus* 4., **14.** MRSA8 **15.** S. *aureus* 9

| 16 | 17 |
| --- | --- |
|  |  |
| 18 | 19 |
|  |  |
| 20 | 21 |
|  |  |
| 22 | 23 |
|  |  |

**Fig. S1.** Antimicrobial activity of S1,2,3, and S4 organic extracts against **16.** MRSA5., **17.** MDR *P. aeruginosa47.,* **18. .** MDR *K.* pneumoniae36., **19.** *P. aeruginosa 47.,* **20**. *S.aureus* (ATCC 25923) **21.** *S.aureus* (ATCC 25923),**22.** *P. aeruginosa* (ATCC 9027) **23.** *P. aeruginosa* (ATCC 9027).

**Fig. S2:** Representative phase-contrast micrographs of untreated Vero control cells and Vero cells treated with S4 extract at the selected non-cytotoxic concentrations used in the antiviral assay. Cell morphology was comparable to the untreated control, confirming the low cytotoxicity observed in the MTT assay.

| **Genome statistics** | **MS_assembly** |  |
| --- | --- | --- |
| Genome fraction (%) | 97.829 |  |
| Duplication ratio | 0.999 |  |
| # genomic features | 4628 + 50 part |  |
| Largest alignment | 732622 |  |
| Total aligned length | 4113403 |  |
| NGA50 | 434500 |  |
| LGA50 | 4 |  |
| **Reads mapping** |  |  |
| Mapped (%) | 81.8 |  |
| Properly paired (%) | 0 |  |
| Singletons (%) | 0 |  |
| Misjoint mates (%) | 0 |  |
| Avg. coverage depth | 13 |  |
| Coverage >= 1x (%) | 95.38 |  |
| **Misassemblies** |  |  |
| # misassemblies | 19 |  |
| Misassembled contigs length | 4148609 |  |
| **Mismatches** |  |  |
| # mismatches per 100 kbp | 711.24 |  |
| # indels per 100 kbp | 68.36 |  |
| # N's per 100 kbp | 0 |  |
| **Statistics without reference** |  |  |
| # contigs | 1 |  |
| Largest contig | 4148609 |  |
| Total length | 4148609 |  |
| Total length (>= 1000 bp) | 4148609 |  |
| Total length (>= 10000 bp) | 4148609 |  |
| Total length (>= 50000 bp) | 4148609 |  |

**Fig. S3**. The Full genome assembly statistics of ***Bacillus spizizenii* CCASU-2025-89** (total contig number, genome size, GC content) and its comparison to the pan-genome sequences of *B. spizizenii* genome (Reference strain: *Bacillus spizizenii* (NCBI accession code, NC_016047.1),
